# Facilitating the transmetalation step with aryl-zincates in nickel-catalyzed enantioselective arylation of secondary benzylic halides

**DOI:** 10.1038/s41467-019-10851-4

**Published:** 2019-07-04

**Authors:** Weichen Huang, Mei Hu, Xiaolong Wan, Qilong Shen

**Affiliations:** 1 Key Laboratory of Organofluorine Chemistry, Shanghai Institute of Organic Chemistry, University of Chinese Academy Sciences, Chinese Academy of Sciences, 345 Lingling Road, 200032 Shanghai, People’s Republic of China; 2 Shandong Provincial Key Laboratory of Fluorine Chemistry and Chemical Materials, School of Chemistry and Chemical Engineering, Shandong Engineering Research Center for Fluorinated Material, University of Jinan, 250022 Jinan, China

**Keywords:** Asymmetric catalysis, Homogeneous catalysis, Stereochemistry

## Abstract

Nickel-catalyzed asymmetric cross-coupling of secondary alkyl electrophiles with different nucleophiles represents a powerful strategy for the construction of chiral tertiary carbon centers. Yet, the use of aryl Grignard reagents or aryl zinc halides in many reactions typically resulted in low enantioselectivity, mainly due to their slow transmetalation step in the catalytical cycle and consequently the requirement of relatively high temperature. Here we report that the use of lithium aryl zincate [Ph_2_ZnBr]Li facilitates the transmetalation step of the nickel-catalyzed cross-coupling reaction. Based on this discovery, a highly enantioselective construction of fluoroalkyl-substituted stereogenic center by a nickel-catalyzed asymmetric Suzuki-Miyaura coupling of α-bromobenzyl trifluoro-/difluoro-/mono- fluoromethanes with a variety of lithium aryl zincates [Ph_2_ZnBr]Li that  were in situ generated from the reaction of lithium organoboronate with 1.0 equivalent of ZnBr_2_ was described.

## Abstract (web summary)

Fluoroalkyl groups are widely found in pharmaceutical products. Here, the authors report a highly enantioselective nickel-catalyzed cross coupling method for the construction of fluoromethylated stereogenic centers via a highly reactive zincate species facilitating the transmetalation step in the nickel catalytic cycle.

## Introduction

**Fig. 1:**
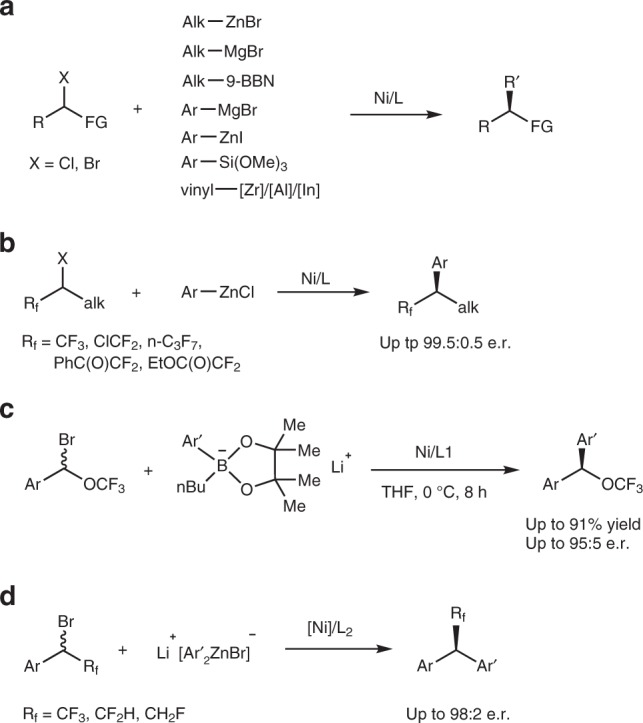
Ni-catalyzed asymmetric cross-coupling of racemic secondary alkyl halides. **a** State-of-the-art. **b** Coupling of fluoroalkylated secondary alkyl bromides with Aryl zinc chloride. **c** Coupling with lithium borates. **d** Coupling with in situ generated zincate (this work)

Over the past two decades, nickel-catalyzed asymmetric cross-coupling of secondary alkyl electrophiles with different nucleophiles has emerged as powerful methods for the construction of chiral tertiary carbon centers^1–4^. Since the seminar work by Fu and co-workers in 2005^5^, a number of activated racemic alkyl halides such as α-bromoamides^5^, α-bromoketones^6^, benzylic bromides and chlorides^7,8^, allylic chlorides^9^ or 1-bromo-1-fluoroalkane^10^ and unactivated racemic alkyl halides such as β- or γ-ether, amide or sulfonyl-substituted alkyl bromides^11,12^, and α-haloboronates^13^ were effectively employed as the coupling partners, while the choice of nucleophiles was originally mainly focused on alkyl zinc halides. Only recently, the nickel-catalyzed asymmetric couplings of racemic alkyl halides were successfully extended to other nucleophiles such as alkyl-9-BBN, aryl Grignard reagents, aryl zinc halides, aryl/vinyl silicates vinyl/alkynyl indium/zirconium/aluminum reagents (Fig. 1a)^6,14–19^.

Organoboron reagents are one of the most widely studied and applied reagents that allows for the efficient construction of carbon-carbon and carbon-heteroatom bonds^20–22^. Non-asymmetric couplings of secondary alkyl bromides with aryl boronic acids under nickel catalysis have been reported in early 2004^23^. Yet, mainly due to the slow transmetalation step of the aryl boronic acid to the active nickel intermediate, theses reactions typically required 60 °C to occur to full conversion. To facilitate the transmetalation step, Fu and co-workers^15^ turned to alkyl-9-BBN and found that the reaction could be conducted at 5 °C-room temperature to ensure high enantioselectivity. Nevertheless, alkyl boranes are generally air and moisture sensitive and should be prepared in situ by hydroboration of alkene before use, which hampered their widespread applications.

In 2017, we discovered that the transmetalation step in nickel-catalyzed asymmetric Suzuki-Miyaura coupling of CF_3_O-substituted secondary benzylic bromide was much faster when easily available, air-insensitive lithium organoboronate instead of aryl boronic acid was used as the nucleophile^24^. In this case, the reaction occurred smoothly at 0 °C to give the coupled product with a CF_3_O-stustituted stereogenic center with excellent enantioselectivity (Fig. 1c). Inspired by this discovery and considering the fact that fluoroalkyl groups, including trifluoromethyl (CF_3_-), difluoromethyl (HCF_2_-) and monofluoromethyl (CH_2_F-) group are important structural motifs in refining the lead compound’s selectivity and pharmacokinetics for new drug discovery^25–28^, we envisaged that the same strategy might work if a fluoroalkylated secondary benzylic bromide was allowed to react with lithium organoboronate. One main problem for the transition-metal-catalyzed coupling reactions of fluoroalkylated secondary benzylic bromides is the fluoride elimination from the fluoroalkylated secondary benzylic metal species if the subsequent transmetalation step is too slow^29,30^. The key for the success of such a coupling reaction, therefore, is to accelerate the transmetalation step. Herein, we report the use of lithium aryl zincate [Ph_2_ZnBr]Li facilitates the transmetalation step of the nickel-catalyzed cross-coupling reaction. Consequently, a nickel-catalyzed highly enantioselective coupling reaction for the construction of the optically active fluoroalkylated benzhydryl derivatives from easily available racemic α-bromobenzyl trifluoro-/difluoro-/monofluoromethanes and lithium organoboronates was developed (Fig. 1d).

**Fig. 2:**
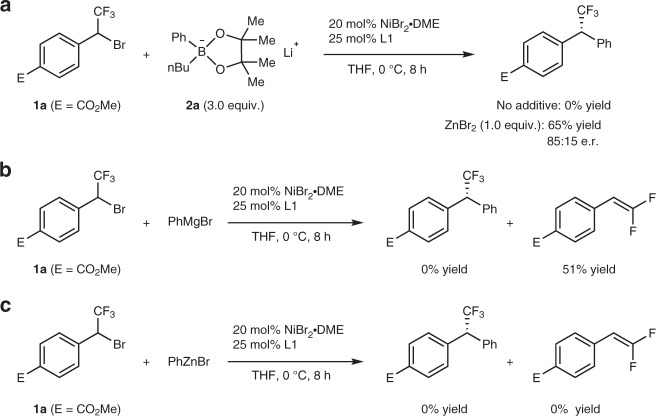
Ni-catalyzed asymmetric cross-coupling reaction with different nucleophiles. **a** Coupling with lithium phenyl boronate. **b** Coupling with Grignard reagent. **c** Coupling with aryl zinc halide

## Results

### Screening of reaction conditions

Initially, we tried the reaction of racemic trifluoromethylated benzylic bromide **1a** and lithium organoboronate **2a** as a model reaction to optimize the reaction conditions. Surprisingly, the reaction did not take place at all when it was conducted in tetrahedrofuran (THF)  at 0 °C for 8.0 h using a combination of 20 mol% NiBr_2_•DME and 25 mol% L1 as the catalyst, which is the condition for the construction of trifluoromethoxylated stereogenic center (Fig. 2a). Notably, when 1.0 equivalent of ZnBr_2_ was used as additive, the reaction occurred after 8 h at 0 °C to afford the coupled product in 65% yield with 85:15 e.r. (Fig. 2a). As a comparison, we studied the reaction of other nucleophiles such as Grignard reagent PhMgBr or PhZnBr. As summarized in (Fig. 2b, c), reaction of substrate **1a** with PhMgBr, under the identical conditions, mainly afforded the undesired defluorinated side product in 51% yield, while the reaction of substrate **1a** with PhZnBr were slow and the formation of the coupled product was not observed.

**Table 1:** Optimization of the reaction conditions


Entry	[Ni]	Ligand	Additive	Solvent	Temp (^o^C)	Yield (%)^a^		e.r.^b^
						3	3’	
1	NiBr_2_•DME	L2	ZnBr_2_	DME	−15	62	5	95.5:4.5
2	NiBr_2_•DME	L2	ZnCl_2_	DME	−15	54	8	95:5
3	NiBr_2_•DME	L2	MgBr_2_	DME	−15	0	0	-
4	NiBr_2_•DME	L2	ZnBr_2_	Diglyme	−15	78	0	95:5
5	NiBr_2_•DME	L2	ZnBr_2_	THF	−15	50	28	94.5:5.5
6	NiBr_2_•DME	L2	ZnBr_2_	Toluene	−15	-	-	-
7	NiBr_2_•DME	L2	ZnBr_2_	DMA	−15	47	6	93:7
8	NiBr_2_•DME	L2	ZnBr_2_	DMF	−15	30	6	93:7
9	NiBr_2_•DME	L2	ZnBr_2_	DME/diglyme	−15	80	0	96:4
10	NiBr_2_•DME	L2	ZnBr_2_	DME/diglyme	rt	75	15	94:6
11^c^	NiBr_2_•DME	L2	ZnBr_2_	DME/diglyme	−15	32	5	95.5:4.5
12^d^	NiBr_2_•DME	L2	ZnBr_2_	DME/diglyme	−15	75	2	95.5:4.5
13	NiCl_2_•DME	L2	ZnBr_2_	DME/diglyme	−15	70	9	95.5:4.5
14	Ni(OAc)_2_	L2	ZnBr_2_	DME/diglyme	−15	71	6	96:4
15	NiBr_2_•DME	L1	ZnBr_2_	DME/diglyme	−15	70	15	88:12
16	NiBr_2_•DME	L3	ZnBr_2_	DME/diglyme	−15	81	0	94:6
17	NiBr_2_•DME	L4	ZnBr_2_	DME/diglyme	−15	30	50	90:10
18	NiBr_2_•DME	L5	ZnBr_2_	DME/diglyme	−15	0	0	-
19	NiBr_2_•DME	L6	ZnBr_2_	DME/diglyme	−15	24	0	58:42
20^e^	-	L2	ZnBr_2_	DME/diglyme	−15	0	0	-
21^f^	NiBr_2_•DME	L2	ZnBr_2_	DME/diglyme	−15	70	9	96:4
22^g^	NiBr_2_•DME	L2	ZnBr_2_	DME/diglyme	−15	50	15	96:4

Unless otherwise noted, all reactions were carried out with compound **1a** (0.1 mmol), **2** (0.3 mmol), NiBr_2_•DME (20 mol%), ligand (25 mol%) under conditions indicated in the scheme

^a^Yields were determined by ^19^F NMR spectroscopy with trifluorotoluene as an internal standard

^b^Detemined by chiral HPLC analysis

^c^0.5 equiv. of ZnBr_2_ was used

^d^2.0 equiv. of ZnBr_2_ was used

^e^NiBr_2_•DME (0 mol%), ligand L2 (25 mol%)

^f^NiBr_2_•DME (10 mol%), ligand L2 (12.5 mol%)

^g^NiBr_2_•DME (5 mol%), ligand L2 (6.25 mol%)

A quick further survey of the reaction conditions disclosed that a combination of NiBr_2_•DME with ligand L2 was the most efficient catalyst and the desired product **3a** was obtained in 62% yield with 95.5:4.5 e.r. along with the undesired defluorinated side product **3a’** in 5% yield when the reaction was conduct at −15 °C for 12  h (Table 1, entry 1). Switching the additive to ZnCl_2_ gave slightly inferior results, while using MgBr_2_ as additive was not effective at all (Table 1, entries 2–3). Further studied showed that reactions in DME or diglyme occurred in moderate yields and good enantioselectivity, while reactions in other solvents such as THF, DMA, or DMF were less effective and reaction in toluene was completely shut down (Table 1, entries 4–8). Notably, using a combination of DME/diglyme (*v*/*v* = 1/1) as the solvent gave slightly improved yield and enantioselectivity (Table 1, entry 9). The amount of ZnBr_2_ was also important for the reaction. While the reaction with 2.0 equivalents of ZnBr_2_ gave comparative yield and enantioselectivity, reaction conducted with 0.5 equivalent of ZnBr_2_ occurred in much lower yield although the enantioselectivity was high (Table 1, entries 11–12). We next studied the effect of different nickel precursors and ligands. It was found that reaction using different nickel precursors such as NiCl_2_•DME or Ni(OAc)_2_ had little effect on the efficiency of the reaction. Yet, the choice of the ligand plays a key role in delivering the good yields and high enantioselectivity. Pyridine-oxazoline ligand with either an electron-donating group (-OMe) or an electron-withdrawing group (-CF_3_) at 5-position, as well as a methyl group at 3-position of the pyridyl moiety were less effective (Table 1, entries 15–17). Likewise, two commonly used dinitrogen ligands for nickel-catalyzed asymmetric coupling reaction were also ineffective under these conditions (Table 1, entries 18–19). Control experiment showed that reaction in the absence of nickel catalyst did not occur at all (Table 1, entry 20). Furthermore, efforts to decreasing the catalyst loadings disclosed that the amount of side products increased to 9–15% when a combination of 10 mol% NiBr_2_•DME and 12.5 mol% L2 or 5.0 mol% NiBr_2_•DME and 6.25 mol% L2 was used (Table 1, entries 21–22).

### Mechanistic investigation

**Fig. 3:**
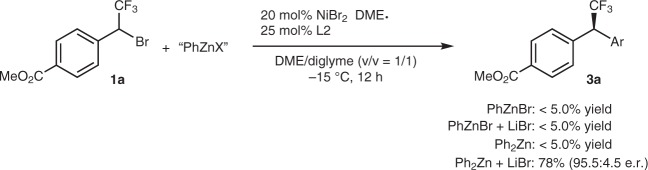
Control experiments. The effects of different nucleophiles

During the optimization of the reaction conditions, it was found that addition of 1.0 equivalent of ZnBr_2_ dramatically accelerated the reaction rate. Presumptively, mixing lithium aryl boronate with ZnBr_2_ might generate several different arylated zinc species that could accelerate the transmetalation step and the overall catalytic reaction. To probe which arylated zinc species was involved in the reaction, we did several control experiments (Fig. 3). First, reaction of compound **1a** with 3.0 equivalents of PhZnBr in the presence/absence of 3.0 equivalents of LiBr occurred under standard conditions in less than 5% yield of the coupled product. Likewise, reaction of compound **1a** with 3.0 equivalents of Ph_2_Zn, again, gave the desired product in less than 5% yield. These results clearly excluded the possibility of the involvement of PhZnBr and Ph_2_Zn in the current reaction. Interestingly, addition of 3.0 equivalent of LiBr to the reaction of compound **1a** with Ph_2_Zn led to full conversion of the starting material and gave the coupled compound **3a** in 78% yield with 95.5:4.5 e.r. These experimental results suggest that an anionic zincate [Ph_2_ZnBr]^-^ might be involved in the reaction, consistent with the observation from Ingleson and co-workers^31^ that mixing 2.0 equivalents of lithium aryl boronate with ZnBr_2_ at room temperature generated an anionic [Ph_x_ZnBr_y_]^-^ (*x* + *y* = 3).

**Fig. 4:**
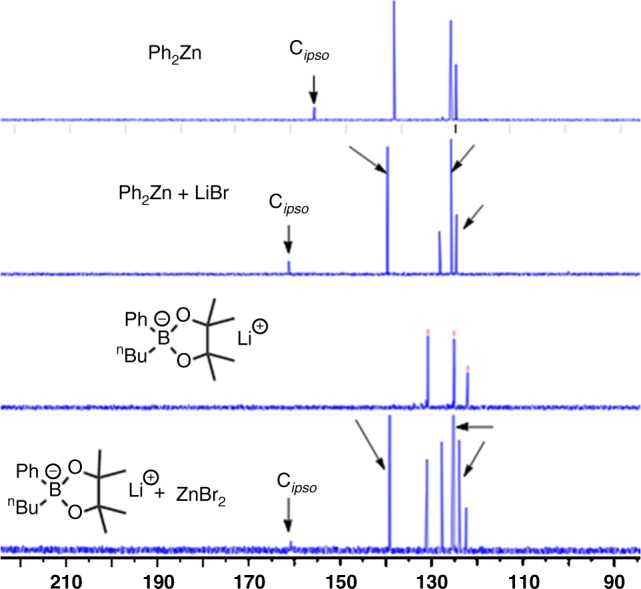
NMR detection of intermediates. ^13^C NMR spectra of Ph_2_Zn, Ph_2_Zn + LiBr, **2a** and **2a** + ZnBr_2_ in THF-d8

To gain more support about the formation of lithium zincate from lithium aryl boronate with ZnBr_2_, we studied and compared the ^13^C nuclear magnetic resonance (NMR) spectra of the species generated from mixing lithium aryl boronate with ZnBr_2_ and Ph_2_Zn with LiBr. As shown in Fig. 4, mixing equimolar amount of Ph_2_Zn with LiBr at room temperature in THF-d8 for 0.5 h cleanly generated [Ph_2_ZnBr]Li, as evidence by a peak with a chemical shift at 161.0 ppm in ^13^C NMR spectrum, which corresponds to the ipso-carbon of the phenyl group in [Ph_2_ZnBr]Li. Likewise, the same species was formed after 0.5 h at room temperature for the reaction of 3.0 equivalvents of lithium phenyl boronate **2a** with ZnBr_2_. These results suggest an anionic arylated zincate [Ph_2_ZnBr]Li could facilitate the transmetalation step, and consequently, accelerates the overall catalytic reaction. However, we could not exclude an alternative pathway in which ZnBr_2_ may abstract a bromide from nickel benzylic bromide complex to generate a cationic η^3^-benzyl nickel species^32^ that could more rapidly participate in a transmetalation reaction with lithium organoboronate **2a**.

**Fig. 5:**
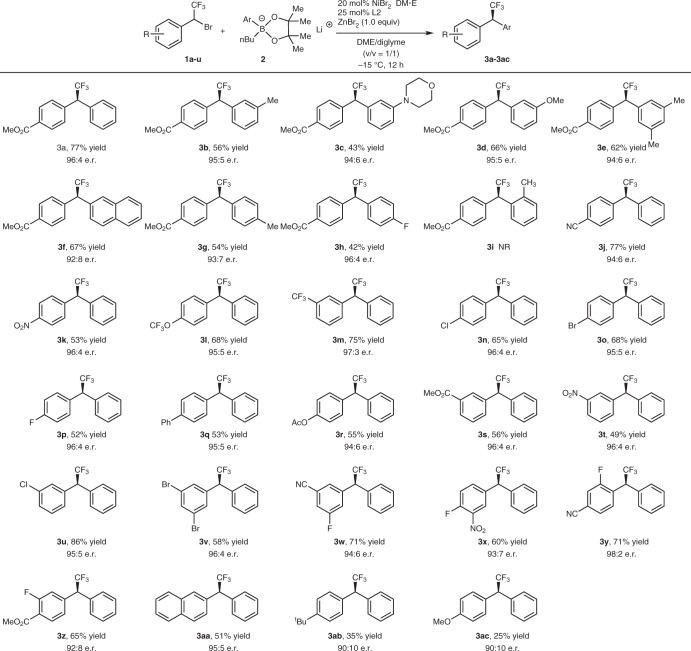
Substrate scope of asymmetric coupling of α-bromobenzyl trifluoromethane with lithium aryl boronates. All reaction were conducted with compound 1 (0.3 mmol), phenylboronic pinacol ester 2 (0.9 mmol), NiBr_2_•DME (20 mol%), ligand L2 (25 mol%) and ZnBr_2_ (0.3 mmol) in DME/diglyme (*v*/*v* = 1/1) at −15 °C for 12 h. Isolated yields and e.r. was determined by chiral HPLC analysis

### Substrate scope investigation

Having identified the optimized conditions and the likelihood role of ZnBr_2_, we next investigated the generality of the nickel-catalyzed coupling reaction for the preparation of enantio-enriched benzhydryl trifluoromethane derivatives. As summarized in Fig. 5, in general, trifluoromethylated benzylic bromides (**1a–e**) with electron-withdrawing substituted groups such as ester, cyano, nitro, trifluoromethyl or trifluoromethoxy group reacted smoothly with lithium phenyl boronates **2a–f** to afford the coupled products in moderate to good yields and enantioselectivities (Figs. 5 and **3****a–h**, **j–m**). For example, reactions of both α-bromo-4-nitrobenzyl trifluoromethane and α-bromo-3-trifluoromethyl benzyl trifluoromethane with lithium phenyl boronate **2a** gave the corresponding products **3k** and **3 m** in 53% and 75% yields with excellent enantioselectivities 96:4 and 97:3 e.r., respectively, (Figs. 5 and **3****k**, **m**). Notably, trifluoromethylated benzylic bromides with a halogen group such as chloride, bromide, and fluorine were compatible and reacted with lithium phenyl boronates **2a** to give the corresponding products **3n–p** in 65%, 68%, and 52% yields, with 96:4, 95:5 and 96:4 e.r., respectively (Figs. 5, **3n–p**). Furthermore, α-bromobenzyl trifluoromethyl with para-, meta-, and ortho-substituents are all compatible coupling partners, affording the desired products in moderate to good yields and enantioselectivities. For example, both α-bromo-3,5-dibromide benzyl trifluoromethane and α-bromo-2-fluorine-4-cyano benzyl trifluoromethane reacted to afford compounds **3v**, **3y** in 58% and 71% yield with 96:4 and 98:2 e.r., respectively (Figs. 5, **3****v**, **y**). Nevertheless, trifluoromethylated benzylic bromides with electron-donating groups occurred in much less yields and moderate enantioselectivities (Figs. 5, **3ab–ac**). In these cases, the formations of two side products including the homocoupling side products and the defluorinated side products were observed. Next, we investigated the scope of lithium aryl boronates. It was found that reactions of lithium aryl boronates with meta-substituted aryl groups occured in moderate to good yields and enantiolectivities (Figs. 5, **3b–e**), while reactions of para-methyl or fluoride substituted lithium aryl boronates occurred to generate the corresponding products in moderate yields and enantioselectivities (Figs. 5, **3g, h**). However, the formation of the desired coupling product was not observed when lithium aryl boronates with ortho-methyl group (**2i**) was subjected to the reaction conditions (Scheme 2, **3i**). Previously reported method for the preparation of enantio-enriched benzhydryl trifluoromethane derivatives typically required to use optically secondary α-(trifluoromethyl)benzyl tosylates to react with various aryl boronic acids in the presence of a palladium catalyst^19,29,33–36^, while Fu and coworker^37^ reported a highly enantioselective nickel-catalyzed coupling of fluoroalkylated secondary alkyl bromide with aryl zinc chlorides (Fig. 1c). Thus, the current method provided an alternative, more efficient method to access this family of compounds.

**Fig. 6:**
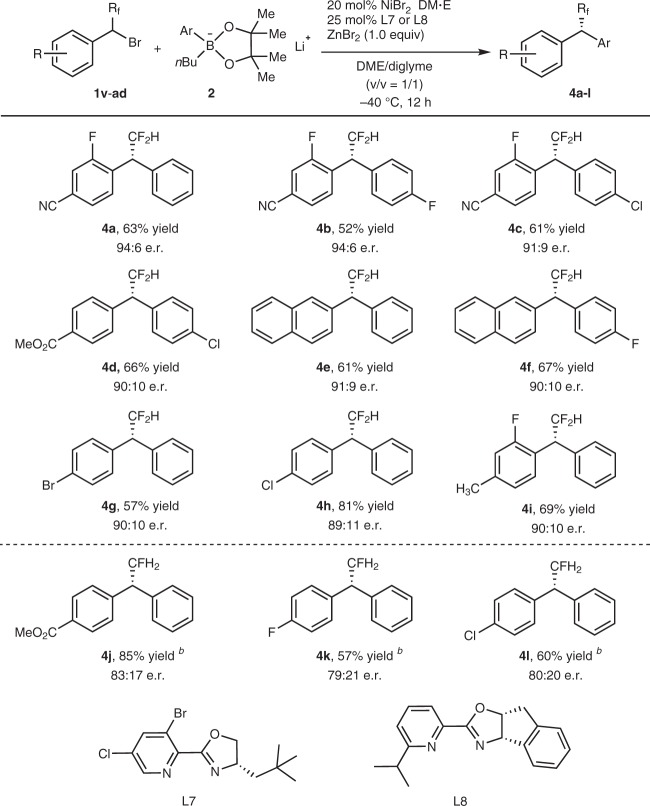
Substrate scope for reactions with α-bromobenzyl di-/mono-fluoromethane. All reaction were conducted with compound 1 (0.3 mmol), phenylboronic pinacol ester 2 (0.9 mmol), NiBr_2_•DME (20 mol%), ligand L7 or L8 (25 mol%) and ZnBr_2_ (0.3 mmol) in DME/diglyme (*v*/*v* = 1/1) at −10 or −40 °C for 12 h. Isolated yields and e.r. was determined by chiral HPLC analysis

Encouraged by the high enantioselectivity in nickel-catalyzed coupling of α-bromobenzyl trifluoromethane with lithium aryl boronates, we next tried to extend this reaction to other fluoroalkyl-substituted benzyl bromides. After a quick screen of the reaction conditions, it was found that when a more sterically hindered ligand **L7** was used as the ligand and the reaction temperature was decreased to −40 °C, good enantioselectivities could be achieved (Fig. 6). For example, reactions of 4-(1-bromo-2,2-difluoroethyl)−3-fluorobenzonitrile with lithium phenyl boronate **2a** and lithium 4-fluorophenyl boronate **2** **h** occurred smoothly after 12 h to afford the corresponding products in 94:6 e.r. (Figs. 6 and 4a, b). Since few methods for the construction of difluoromethyl-substituted stereogenic carbon center have been reported previously^38,39^, the current method represents an attractive approach for the preparation of optically active difluoromethylated benzhydryl derivatives.

On the other hand, reaction of monofluoromethylated substrates were much more challenging. After carefully screening of the combination of nickel salts and ligands, it was found that using a combination of NiBr_2_•DME with ligand **L8** could catalyze the reactions of 4-(1-bromo-2-fluoroethyl)arenes with lithium phenyl boronate **2a** to give the corresponding coupled products **4j**–**l** after 12 h at −10 °C in moderate enantioselectivities (79:21 ∼ 83:17 e.r.).

### Synthetic application

**Fig. 7:**
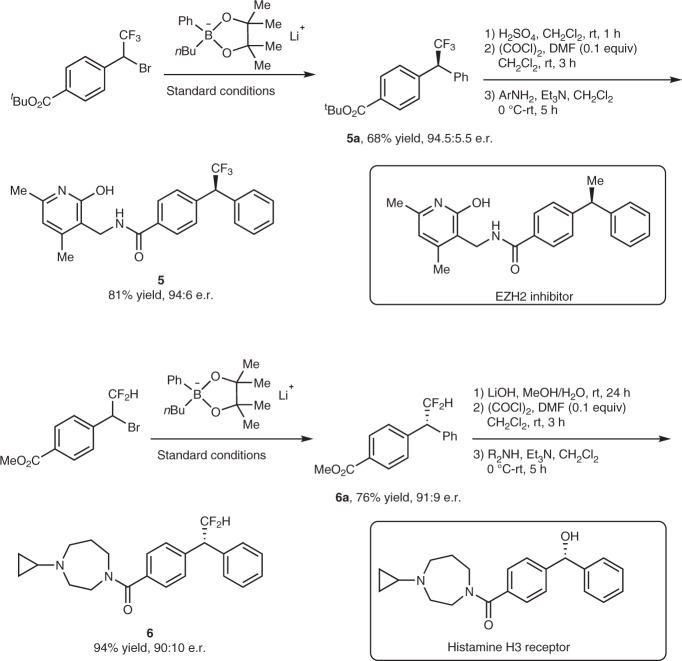
Synthetic applications. Application in the preparation of fluoroalkylated derivatives of drug candidates

To showcase the applicability of the nickel-catalyzed asymmetric coupling reaction of racemic trifluoromethylated benzylic bromide with lithium organoboronate, we applied this protocol for the synthesis of trifluoromethylated mimic of an inhibitor for the histone lysine methyltransferase enhancer of Zeste Homolog 2 (EZH2)^40^. As shown in Fig. 7, compound **5** was generated in 55% overall yield with 94:6 e.r. via a four-step transformation from easily available α-bromo-4-*tert*-butoxycarbylbenzyl trifluoromethane.

Owing to the slightly acidic proton in the difluoromethyl group, which allows it to act as a lipophilic hydrogen-bond donor, the difluoromethyl group (CHF_2_) was generally considered as a bioisostere for a hydroxy goup (-OH)^41^. Replacement of a hydroxy group of a drug molecule with a difluoromethyl group may result in a Me-too or Me-better drug molecule. Consequently, a difluoromethylated compound **6**, which is a mimic of histamine H3 receptor^42^, was synthesized in 71% overall yield and 90:10 e.r. after four steps.

## Discussion

Inspired by the mechanistic studies disclosed that a highly reactive zincate [Ph_2_ZnBr]Li would facilitate the transmetalation step of the nickel-catalyzed cross-coupling reaction, we successfully developed a highly enantioselective nickel-catalyzed coupling of easily available α-bromobenzyl fluooalkanes with a variety of lithium aryl boronates in the presence of stochiometric amount of ZnBr_2_. Thus, the protocol may serve as a versatile, efficient, and convenient approach for the rapid access of chiral benzhydryl fluoroalkane derivatives. The application of the high-reactive lithium aryl zincate [Ar_2_ZnBr]Li in other transition-metal-catalyzed cross-coupling reactions are undergoing currently in our laboratory.

## Methods

### Coupling of trifluoromethylated secondary benzyl bromides

In a glove box, phenylpinacolboronate ester (5.1 g, 25 mmol) was weighted into a 100 mL Schlenk tube, and 40 mL of anhydrous THF was added. The mixture was taken out from the glove box and cooled at −20 °C. *n*-BuLi (25 mmol, 10 mL, 2.5 M in Hexanes) was added. The mixture was stirred at −20 °C for 2 h. Then the Schlenk tube was taken into the glove box, the solvents were removed under vacuum to give lithium phenyl pinacol boronate.

In an argon-filled glove box, lithium organoborate (371 mg, 0.900 mmol, 3.00 equiv.), ligand L2 (26.8 mg, 0.0750 mmol, 0.250 equiv.), ZnBr_2_ (67.5 mg, 0.300 mmol, 1.00 equiv.), and NiBr_2_.DME (18.5 mg, 0.0600 mmol, 0.200 equiv) were placed into a 25 mL Schlenk tube. To this vial was added 5.0 mL of anhydrous DME/diglyme(*v*/*v* = 1:1). The Schlenk tube was taken out from the glove box and cooled at −15 °C. α-Bromo-4-methoxycarbonylbenzyl trifluoromethyl **1a** (89.1 mg, 0.300 mmol) was added and the mixture was stirred at -15 °C for 12 h. The mixture was quenched by addition of water (5.0 mL) and extracted with Et_2_O (10.0 mL × 3). The organic layer was combined, dried over anhydrous Na_2_SO_4_ and concentrated under vacuum. The crude product was purified by column chromatography on silica gel with pentane/ethyl acetate as the eluent to give **(S)-**methyl 4-(2,2,2-trifluoro-1-phenylethyl)benzoate **3a** as a yellow liquid (77% yield, 96:4 e.r.).

## Supplementary information

Supplementary Information

Peer Review File

## Data Availability

Experimental procedures and characterization data are available within this article and its Supplementary Information. Data are also available from the corresponding author on request. The X-ray crystallographic coordinates for structures reported in this study have been deposited at the Cambridge Crystallographic Data Center (CCDC), under deposition numbers 1868035 and 1898246. These data can be obtained free of charge from The Cambridge Crystallographic Data Center via www.ccdc.cam.ac.uk/data_request/cif.
