# Supplementary material for: Facilitating the transmetalation step with aryl-zincates in nickel-catalyzed enantioselective arylation of secondary benzylic halides
Source: Nat Commun. 2019 Jul 4;10:2963. doi: 10.1038/s41467-019-10851-4 (PMC6609707; doi:10.1038/s41467-019-10851-4)
Supplement: Supplementary file 1 — Supplementary Information [file 41467_2019_10851_MOESM1_ESM.pdf]

**Supporting Information for:**

# Facilitating the Transmetalation Step with Aryl-Zincates in Nickel-Catalyzed Enantioselective Arylation of Secondary Benzylic Halides

Shen et al.

**Supplementary Methods**

**General Information**

All solvents were purified by standard method. $^{1}$H NMR spectrawere recorded on a 500 MHz, 400 MHz or 300 MHz. $^{19}$F NMR were recorded on a 376MHz or 282 MHz spectrometer. $^{13}$C NMR spectra were recorded on a Bruker AM400spectrometer and Agilent 400 or 500 MHz spectrometer. $^{1}$H NMR and $^{13}$C NMRchemical shifts were determined relative to internal standard TMS at δ 0.0 and $^{19}$FNMR chemical shifts were determined relative to $CFCl_3$ as inter standard. Chemicalshifts (δ) are reported in ppm, and coupling constants (*J*) are in Hertz (Hz). Thefollowing abbreviations were used to explain the multiplicities: s = singlet, d = doublet,t = triplet, q = quartet, m = multiplet, br = broad. Flash column chromatograph wascarried out using 300-400 mesh silica gel at medium pressure.

All reagents were received from commercial sources. Solvents were freshlydried and degassed according to the purification handbook *Purification of LaboratoryChemicals* before using.

**Supplementary Table 1**. Effect of the equivalents of $ZnBr_2$.

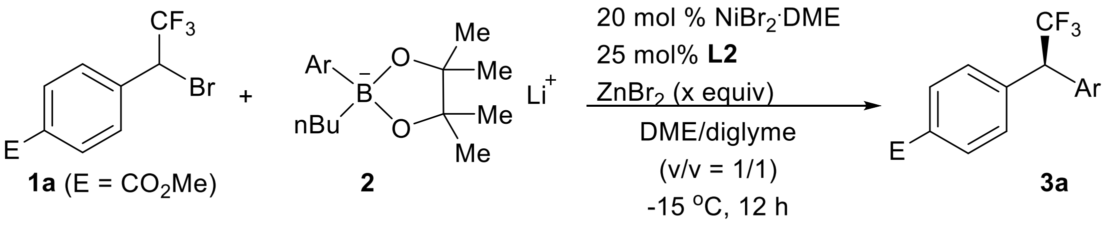


| entry | x | yield (%) 3a | yield (%) 3a' | e.r |
|---|---|---|---|---|
| 1 | 0.5 | 32 | 5 | 95.5:4.5 |
| 2 | **1** | **80** | **0** | **96:4** |
| 3 | 2 | 75 | 2 | 95:4.5 |
| 4 | 3 | 73 | 3 | 95:5 |

[a] Reaction conditions: compound **1** (0.1 mmol), phenylboronic pinacol ester **2** (0.3 mmol), $NiBr_2$•DME (20 mol%), ligand **L1** (25 mol%) and $ZnBr_2$(xequiv.) in DME/diglyme (v/v = 1/1) at -15 °C for 12 h; [b] Yields were determined by $^{19}F$ NMR spectroscopy with trifluorotoluene as an internal standard.

**Supplementary Table 2**. Effect of the ratio of DME and diglyme.

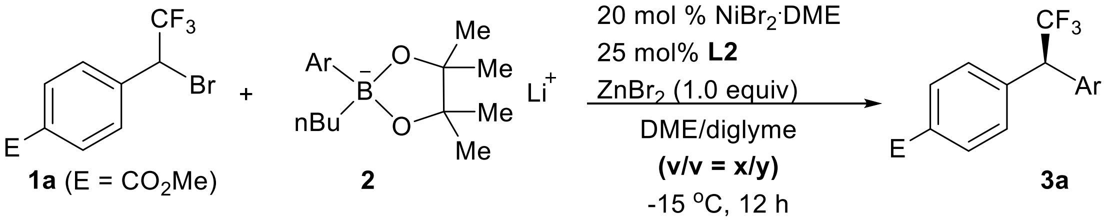


| entry | x/y | yield (%) 3a | yield (%) 3a' | e.r |
|---|---|---|---|---|
| 1 | 4/1 | 71 | 5 | 95:5 |
| 2 | 2/1 | 75 | 3 | 95:5 |
| **3** | **1/1** | **80** | **0** | **96:4** |
| 4 | 1/2 | 81 | 0 | 95.5:4.5 |
| 5 | 1/4 | 76 | 0 | 95:5 |

[a] Reaction conditions: compound **1** (0.1 mmol), phenylboronic pinacol ester **2** (0.3 mmol), $NiBr_2$•DME (20 mol%), ligand **L1** (25 mol%) and $ZnBr_2$(1.0equiv.) in DME/diglyme (v/v = x/y) at -15 °C for 12 h; [b] Yields were determined by $^{19}F$ NMR spectroscopy with trifluorotoluene as an internal standard.

**Supplementary Table 3**. Effect of the ligand.

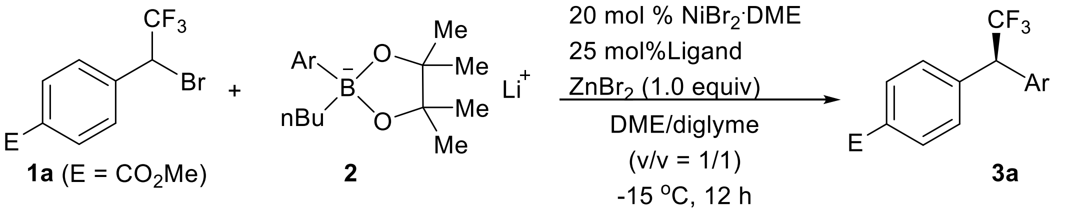


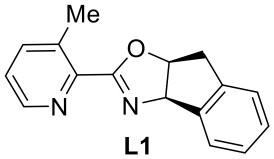


**L1**

**3a**: 70% 85:15 e.r
**3a'**: 18%

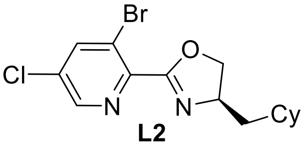


**L2**

**3a**: 80%, 96:4 e.r
**3a'**: 0%

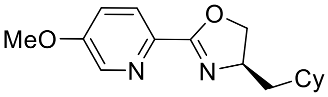


**L3**

**3a**: 81%, 94:6 e.r
**3a'**: 0%

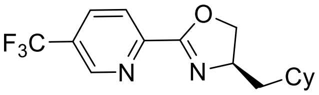


**L4**

**3a**: 30%, 90:10 e.r
**3a'**: 50%

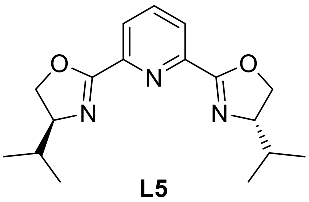


**L5**

**3a**: 0%
**3a'**: 0%

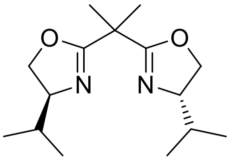


**L6**

**3a**: 24%, 58:42 e.r
**3a'**: 0%

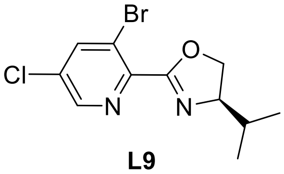


**L9**

**3a**: 63%, 91.5:8.5 e.r
**3a'**: 0%

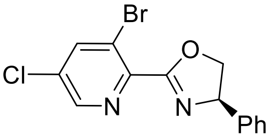


**L10**

**3a**: 40%, 88.5:11.5 e.r
**3a'**: 0%

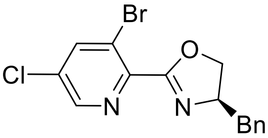


**L11**

**3a**: 33%, 86.5:13.5 e.r
**3a'**: 0%

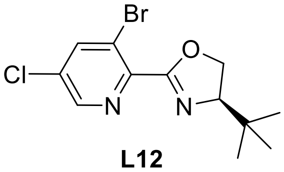


**L12**

**3a**: 80%, 75:25 e.r
**3a'**: 15%

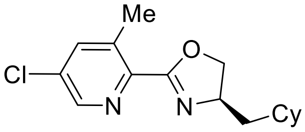


**L13**

**3a**: 69%, 93.5:6.5 e.r
**3a'**: 0%

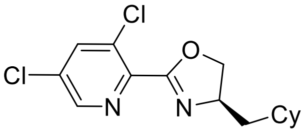


**L14**

**3a**: 63%, 94.5:5.5 e.r
**3a'**: 9%

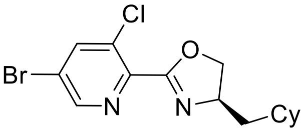


**L15**

**3a**: 68%, 95:5 e.r
**3a'**: 15%

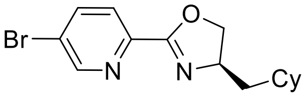


**L16**

**3a**: 88%, 94:6 e.r
**3a'**: 0%

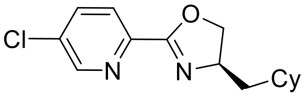


**L17**

**3a**: 81%, 93.5:6.5 e.r
**3a'**: 15%

[a] Reaction conditions: compound **1** (0.1 mmol), phenylboronic pinacol ester **2** (0.3 mmol), $NiBr_2$•DME (20 mol%), ligand **L1** (25 mol%) and $ZnBr_2$(1.0 equiv.) in DME/diglyme (v/v = 1/1) at -15 °C for 12 h; [b] Yields were determined by $^{19}F$ NMR spectroscopy with trifluorotoluene as an internal standard.

**General procedure for the preparation of $CF_3$-substituted secondary benzylic bromides**

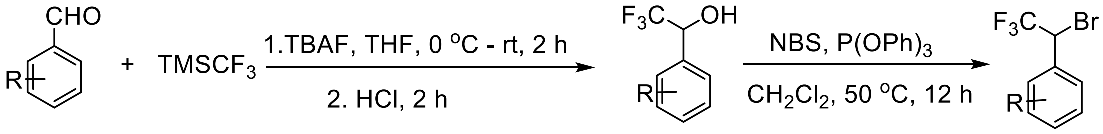


Methyl 4-formylbenzoate (5.00 g, 30.5 mmol) was weighted into a 150 mL Schlenk flask and trifluoromethyltrimethylsilane ($TMSCF_3$) (5.70mL, 36.6 mmol) and anhydrous THF (50.0 mL) were added under an argon atmosphere. The mixture was cooled at 0 °C. Then a solution of TBAF (1.0 M in THF, 0.37 mL, 0.37 mmol) was added over 20 min. The resulting mixture was stirred at 0 °C for 30 min, then was allowed to warm to room temperature. The reaction was further stirred for 2 h. An aqueous solution of HCl (1.0 N, 60 mL) was added, and the mixture was stirred for another 2 h. Then, the mixture was extracted with ethyl acetate (3 × 50 mL), and the combined organic layers were dried over anhydrous $Na_2SO_4$. The solvent was removed under vacuum and the crude product was used without further purification.

Methyl 4-(2,2,2-trifluoro-1-hydroxyethyl)benzoate obtained from the previous step was dissolved in dichloromethane (50.0 mL). To the solution was added successively NBS (7.82 g, 44.1 mmol) and triphenyl phosphite (13.5 g, 44.1 mmol). The resulting mixture was stirred at 50 °C for 12 h and was then cooled to room temperature. The solvent was evaporated in vacuo. The residue was purified by column chromatography on silica gel with a gradient eluent of petroleum ether and ethyl acetate to give compound **1a-u**.

**Methyl 4-(1-bromo-2,2,2-trifluoroethyl)benzoate 1a**

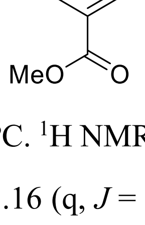


White solid (5.7 g, 57%). Mp: 45-47 °C. $^1H$ NMR (400 MHz, $CDCl_3$) δ 8.06 (d, $J$ = 8.4 Hz, 2 H), 7.59 (d, $J$ = 8.2 Hz, 2 H), 5.16 (q, $J$ = 7.3 Hz, 1 H), 3.93 (s, 3 H); $^{19}F$ NMR

(376 MHz, $CDCl_3$) δ -70.31 (d, *J* = 7.3 Hz, 3 F); $^{13}$C NMR (101 MHz, $CDCl_3$) δ 166.12, 137.28, 131.67, 130.07, 129.25, 123.21 (q, *J* = 278.1 Hz), 52.37, 46.20 (q, *J* = 34.4 Hz) ppm. MS (DART POS): 314 ($M+NH_4$); HRMS (DART POS): Calcd for $C_{10}H_{12}O_2NF_3Br$: 313.9998; Found: 313.9998. IR (KBr): $\nu_{max}$ = 3005, 1954, 2874, 1936, 1721, 1615, 1460, 1439, 1257, 1226, 1112, 1021, 865, 814, 706 $cm^{-1}$.

**4-(1-Bromo-2,2,2-trifluoroethyl)benzonitrile 1b**

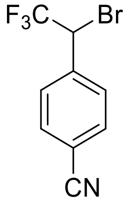


White solid (2.4 g, 57%). Mp: 95-97 °C. $^1$H NMR (400 MHz, $CDCl_3$) δ 7.71 (d, *J* = 8.2 Hz, 2 H), 7.64 (d, *J* = 8.2 Hz, 2 H), 5.15 (q, *J* = 7.1 Hz, 1 H); $^{19}$F NMR (376 MHz, $CDCl_3$) δ -70.29 (d, *J* = 7.1 Hz, 3 F); $^{13}$C NMR (101 MHz, $CDCl_3$) δ 137.55, 132.66, 130.01, 123.01 (q, *J* = 278.3 Hz), 117.81, 114.10, 45.67 (q, *J* = 34.6 Hz) ppm. MS (EI): 184 (100), 263 ($M^+$); HRMS (EI): Calcd for $C_9H_5NF_3Br$: 262.9557; Found: 262.9561. IR (KBr): $\nu_{max}$ = 3099.6, 2994, 2234, 1929, 1610, 1505, 1419, 1362, 1258, 1187, 876, 669 $cm^{-1}$.

**1-(1-Bromo-2,2,2-trifluoroethyl)-4-nitrobenzene 1c**

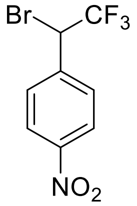


White solid (850 mg, 46%). Mp: 67-68 °C. $^1$H NMR (400 MHz, $CDCl_3$) δ 8.27 (d, *J* = 8.9 Hz, 2 H), 7.72 (d, *J* = 8.8 Hz, 2 H), 5.22 (q, *J* = 7.1 Hz, 1 H); $^{19}$F NMR (376 MHz, $CDCl_3$) δ -70.21 (d, *J* = 7.1 Hz, 3 F); $^{13}$C NMR (101 MHz, $CDCl_3$) δ 148.72, 139.31, 130.36, 124.06, 123.00 (q, *J* = 278.4 Hz), 45.25 (q, *J* = 34.7 Hz) ppm. MS (EI): 204 (100), 283 ($M^+$); HRMS (EI): Calcd for $C_8H_5NO_2F_3Br$: 282.9456; Found: 282.9454. IR (KBr): $\nu_{max}$ = 3114, 3084, 2991, 2711, 1932, 1608, 1526, 1420, 1349, 1257, 1165, 1017, 845, 704 $cm^{-1}$.

**1-(1-Bromo-2,2,2-trifluoroethyl)-4-(trifluoromethoxy)benzene 1d**

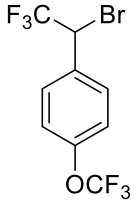


Yellow liquid (850 mg, 51%). $^{1}$H NMR (400 MHz, $CDCl_3$) δ 7.54 (d, $J$ = 8.7 Hz, 2 H), 7.23 (d, $J$ = 8.5 Hz, 2 H), 5.12 (q, $J$ = 7.3 Hz, 1 H); $^{19}$F NMR (376 MHz, $CDCl_3$) δ -57.93 (s, 3 F), -70.72 (d, $J$ = 7.2 Hz, 3 F); $^{13}$C NMR (101 MHz, $CDCl_3$) δ 150.23, 131.33, 130.82,123.21 (q, $J$ = 278.76 Hz), 121.09, 120.35（q, $J$ = 257 Hz), 45.89 (q, $J$ = 34.5 Hz) ppm. MS (EI): 243 (100), 320 ($M^+$); HRMS (EI): Calcd for $C_8H_5F_3Br_2$: 320.0637; Found: 320.0641. IR (KBr): $\nu_{max}$ = 2924, 1904, 1724, 1547, 1512, 1344, 1260, 1221, 1163, 1114, 1020, 857, 828, 678, 630 cm$^{-1}$.

**1-(1-Bromo-2,2,2-trifluoroethyl)-3-(trifluoromethyl)benzene 1e**

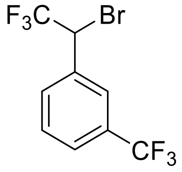


Yellow liquid (830 mg, 60%). $^{1}$H NMR (400 MHz, $CDCl_3$) δ 7.79 – 7.60 (m, 3 H), 7.55 (t, $J$ = 7.8 Hz, 1 H), 5.17 (q, $J$ = 7.2 Hz, 1 H); $^{19}$F NMR (376 MHz, $CDCl_3$) δ -62.87 (s, 3 F), -70.55 (d, $J$ = 7.2 Hz, 3 F); $^{13}$C NMR (101 MHz, $CDCl_3$) δ 133.82, 132.49, 131.50 (q, $J$ = 32.8 Hz), 129.58, 126.89 (q, $J$ = 3.7 Hz), 126.02, 123.51 (q, $J$ = 278.76 Hz), 123.14 (q, $J$ = 273.71 Hz), 45.97 (q, $J$ = 34.5 Hz) ppm. MS (EI): 227 (100), 305 ($M^+$); HRMS (EI): Calcd for $C_9H_5F_6Br$: 305.9479; Found: 305.9483. IR (KBr): $\nu_{max}$ = 2595, 2925, 2856, 1734, 1330, 1259, 1170, 1133, 1079, 849, 705, 686 cm$^{-1}$.

**1-(1-Bromo-2,2,2-trifluoroethyl)-4-chlorobenzene 1f**

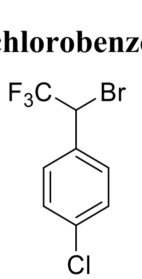


Yellow liquid (980 mg, 75%). $^{1}$H NMR (400 MHz, $CDCl_3$) δ 7.45 (d, $J$ = 8.5 Hz, 2 H), 7.38 (d, $J$ = 8.5, 2 H), 5.10 (q, $J$ = 7.3 Hz, 1 H); $^{19}$F NMR (376 MHz, $CDCl_3$) δ -70.62 (d, $J$ = 7.3 Hz, 3 F); $^{13}$C NMR (101 MHz, $CDCl_3$) δ 136.18, 131.29, 130.47, 129.18,

123.21 (q, $J$ = 278.1 Hz), 46.15 (q, $J$ = 34.4 Hz) ppm. MS (EI): 193 (100), 272 ($M^+$); HRMS (EI): Calcd for $C_8H_5F_3ClBr$: 271.9215; Found: 271.9208. IR (KBr): $\nu_{max}$ = 2927, 1905, 1597, 1492, 1412, 1342, 1304, 1258, 1218, 1162, 1113, 1017, 852, 695 $cm^{-1}$.

**1-Bromo-4-(1-bromo-2,2,2-trifluoroethyl)benzene 1g**

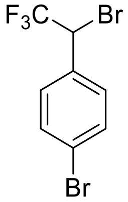


Yellow liquid (1.3g, 71%). $^1H$ NMR (400 MHz, $CDCl_3$) δ 7.54 (d, $J$ = 8.5 Hz, 2 H), 7.38 (d, $J$ = 8.4 Hz, 2 H), 5.08 (q, $J$ = 7.3 Hz, 1 H); $^{19}F$ NMR (376 MHz, $CDCl_3$) δ -70.60 (d, $J$ = 7.3 Hz, 3 F); $^{13}C$ NMR (101 MHz, $CDCl_3$) δ 132.16, 131.80, 130.71, 124.41, 123.15 (q, $J$ = 278.1 Hz), 46.20 (q, $J$ = 34.4 Hz) ppm. MS (EI): 237 (100), 316 ($M^+$); HRMS (EI): Calcd for $C_8H_5F_3Br_2$: 315.8710; Found: 315.8709. IR (KBr): $\nu_{max}$ = 2957, 2925, 1709, 1591, 1491, 1401, 1351, 1257, 1184, 1134, 1076, 1012, 872, 812, 762, 728, 678, 668 $cm^{-1}$.

**1-(1-Bromo-2,2,2-trifluoroethyl)-4-fluorobenzene 1h**

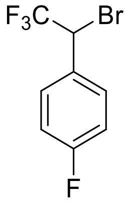


Yellow liquid (820 mg, 45%). $^1H$ NMR (400 MHz, $CDCl_3$) δ 7.50 (dd, $J$ = 8.3, 5.2 Hz, 2 H), 7.09 (t, $J$ = 8.6 Hz, 2 H), 5.12 (q, $J$ = 7.3 Hz, 1 H); $^{19}F$ NMR (376 MHz, $CDCl_3$) δ -70.52 (d, $J$ = 7.3 Hz, 3 F), -105.01 – -115.81 (m, 1 F); $^{13}C$ NMR (101 MHz, $CDCl_3$) δ 163.46 (d, $J$ = 250.6 Hz), 131.10 (d, $J$ = 7.9 Hz), 128.74, 123.27 (q, $J$ = 277.9 Hz), 116.01 (d, $J$ = 22.0 Hz), 46.20 (q, $J$ = 34.3 Hz) ppm. MS (EI): 177 (100), 256 ($M^+$); HRMS (EI): Calcd for $C_8H_5F_4Br$: 255.9511; Found: 255.9510. IR (KBr): $\nu_{max}$ = 2922, 2849, 1722, 1609, 1548, 1514, 1493, 1233, 1184, 1138, 839, 753, 689 $cm^{-1}$.

**4-(1-Bromo-2,2,2-trifluoroethyl)-1,1'-biphenyl 1i**

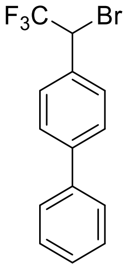


White solid (680 mg, 40%). Mp: 82-84 °C. $^1$H NMR (400 MHz, $CDCl_3$) δ 7.67 – 7.57 (m, 6 H), 7.48 (t, *J* = 7.5 Hz, 2 H), 7.40 (dd, *J* = 8.3, 6.3 Hz, 1 H), 5.20 (q, *J* = 7.4 Hz, 1 H); $^{19}$F NMR (376 MHz, $CDCl_3$) δ -70.32 (d, *J* = 7.4 Hz, 3 F); $^{13}$C NMR (101 MHz, $CDCl_3$) δ 142.95, 139.92, 131.61, 129.53, 128.89, 127.90, 127.49, 127.15, 124.42 (q, *J* = 278.7 Hz), 46.89 (q, *J* = 34.1 Hz) ppm. MS (EI): 235 (100), 314 ($M^+$); HRMS (EI): Calcd for $C_{14}H_{10}F_3Br$: 313.9918; Found: 313.9911. IR (KBr): $\nu_{max}$ = 3077, 3043, 3031, 1918, 1486, 1413,1287, 1254, 1162, 1073, 1007, 822, 697, 540, 510 cm$^{-1}$.

**4-(1-Bromo-2,2,2-trifluoroethyl)phenyl acetate 1j**

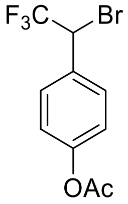


Yellow liquid (680 mg, 38%). $^1$H NMR (400 MHz, $CDCl_3$) δ 7.52 (d, *J* = 8.5 Hz, 2 H), 7.20 – 7.05 (m, 2 H), 5.13 (q, *J* = 7.3 Hz, 1 H), 2.31 (s, 3 H); $^{19}$F NMR (376 MHz, $CDCl_3$) δ -70.58 (d, *J* = 7.3 Hz, 3 F); $^{13}$C NMR (101 MHz, $CDCl_3$) δ 169.01, 151.75, 130.40, 130.21, 123.31 (q, *J* = 278.0 Hz), 122.09, 46.33 (q, *J* = 34.3 Hz), 21.11 ppm. HRMS (EI): Calcd for $C_{10}H_8O_2F_3Br$: 295.9660; Found: 295.9662. IR (KBr): $\nu_{max}$ = 3353, 1721, 1615, 1518, 1451, 1359, 1259, 1177, 1127, 1066, 1014, 826, 745, 670, 583 cm$^{-1}$.

**Methyl 3-(1-bromo-2,2,2-trifluoroethyl)benzoate 1k**

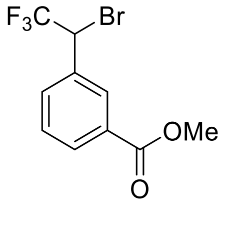

Yellow liquid (1.2 g, 71%). $^{1}$H NMR (400 MHz,$CDCl_3$) δ 8.17 (s, 1 H), 8.08 (d, *J* = 7.8 Hz, 1 H), 7.73 (d, *J* = 7.8 Hz, 1 H), 7.50 (t, *J* = 7.8 Hz, 1 H), 5.18 (q, *J* = 7.3 Hz, 1 H), 3.94 (s, 3 H); $^{19}$F NMR (376 MHz, $CDCl_3$) δ -70.50 (d, *J* = 7.3 Hz, 3 F ); $^{13}$C NMR (101 MHz, $CDCl_3$) δ 166.06, 133.41, 133.29, 131.12, 130.95, 130.31, 129.1, 123.26 (q, *J* = 278.1 Hz), 52.41, 46.33 (q, *J* = 34.3 Hz) ppm. MS (EI): 217 (100), 296 ($M^+$); HRMS (EI): Calcd for $C_{10}H_8O_2F_3Br$: 295.9660; Found: 295.9655. IR (KBr): $\nu_{max}$ = 3430, 2997, 2955, 1727, 1591, 1489, 1435, 1346, 1258, 1180, 1113, 1026, 963, 757, 709, 664 $cm^{-1}$.

**1-(1-Bromo-2,2,2-trifluoroethyl)-3-nitrobenzene 1l**

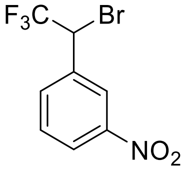


White solid (980 mg, 53%). Mp: 68-70 °C. $^{1}$H NMR (400 MHz, $CDCl_3$) δ 8.39 (s, 1 H), 8.35 – 8.21 (m, 1 H), 7.88 (d, *J* = 7.7 Hz, 1 H), 7.63 (t, *J* = 8.0 Hz, 1 H), 5.23 (q, *J* = 7.1 Hz, 1 H); $^{19}$F NMR (376 MHz, $CDCl_3$) δ -70.51 (d, *J* = 7.1 Hz, 3 F); $^{13}$C NMR (101 MHz, $CDCl_3$) δ 148.34, 135.06, 134.75, 130.16, 124.93, 124.34, 123.02 (q, *J* = 278.3 Hz), 45.32 (q, *J* = 34.7 Hz) ppm. MS (EI): 204 (100), 283 ($M^+$); HRMS (EI): Calcd for $C_8H_5NO_2F_3Br$: 282.9456; Found: 282.9452. IR (KBr): $\nu_{max}$ = 3087, 2994, 1983, 1932, 1533, 1355, 1223, 1191, 1113, 1026, 857, 700 $cm^{-1}$.

**1-(1-Bromo-2,2,2-trifluoroethyl)-3-chlorobenzene 1m**

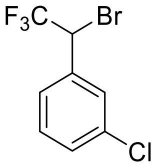


Yellow liquid (1.3 g, 68%). $^{1}$H NMR (400 MHz, $CDCl_3$) δ 7.53 (s, 1 H), 7.45 – 7.29 (m, 3 H), 5.09 (q, *J* = 7.3 Hz, 1 H); $^{19}$F NMR (376 MHz, $CDCl_3$) δ -70.46 (d, *J* = 7.2 Hz, 3 F); $^{13}$C NMR (101 MHz, $CDCl_3$) δ 134.77, 134.53, 130.23, 130.11, 129.30, 127.30, 123.15 (q, *J*= 278.76 Hz), 45.97 (q, *J* = 34.4 Hz) ppm. MS (EI): 193 (100), 272 ($M^+$); HRMS (EI): Calcd for $C_8H_5F_3ClBr$: 271.9215; Found: 271.9224. IR (KBr): $\nu_{max}$ = 2957, 2921, 2849, 1383, 1259, 1205, 1115, 1034, 850, 807, 748, 668 $cm^{-1}$.

**1,3-Dibromo-5-(1-bromo-2,2,2-trifluoroethyl)benzene 1n**

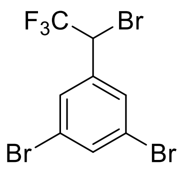

White solid (1.1 g, 68%). Mp: 34-36 ºC. $^{1}$H NMR (400 MHz, $CDCl_3$) δ 7.72 (s, 1 H), 7.59 (s, 2 H), 5.01 (q, *J* = 7.1 Hz, 1 H); $^{19}$F NMR (376 MHz, $CDCl_3$) δ -70.35 (d, *J* = 5.8 Hz, 3 F); $^{13}$C NMR (101 MHz, $CDCl_3$) δ 136.18, 135.81, 131.00, 123.29, 122.95 (q, *J* = 278.4 Hz), 44.94 (q, *J* = 34.7 Hz) ppm. MS (EI): 317 (100), 394 ($M^+$); HRMS (EI): Calcd for $C_8H_5F_3ClBr$: 393.7815; Found: 393.7826. IR (KBr): $\nu_{max}$ = 3075, 2979, 1584, 1559, 1430, 1293, 1259, 1182, 1114, 838, 730, 635 cm$^{-1}$.

**3-(1-Bromo-2,2,2-trifluoroethyl)-5-fluorobenzonitrile 1o**

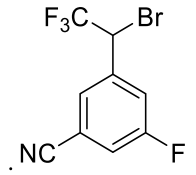

Yellow liquid (930 mg, 49%). $^{1}$H NMR (400 MHz, $CDCl_3$) δ 7.61 (s, 1 H), 7.52 (d, *J* = 8.7 Hz, 1 H), 7.43 (d, *J* = 7.6 Hz, 1 H), 5.14 (q, *J* = 7.0 Hz, 1 H); $^{19}$F NMR (376 MHz, $CDCl_3$) δ -70.50 (d, *J* = 7.0 Hz, 3 F), -107.52 (t, *J* = 8.2 Hz, 1 F); $^{13}$C NMR (101 MHz, $CDCl_3$) δ 162.11 (d, *J* = 252.8 Hz), 136.82 (d, *J* = 8.2 Hz), 128.80 (d, *J* = 3.0 Hz), 122.82 (q, *J* = 278.4 Hz), 121.44 (d, *J* = 23.2 Hz), 120.75 (d, *J* = 24.6 Hz), 116.57 (d, *J* = 3.2 Hz), 114.74 (d, *J* = 9.7 Hz), 42.94 (q, *J* = 35.0 Hz) ppm. MS (EI): 202 (100), 281 ($M^+$); HRMS (EI): Calcd for $C_9H_4F_4Br$: 280.9463; Found: 280.9456. IR (KBr): $\nu_{max}$ = 3086, 2990, 2235, 1598, 1440, 1301, 1173, 1137, 1000, 845, 723 cm$^{-1}$.

**4-(1-Bromo-2,2,2-trifluoroethyl)-1-fluoro-2-nitrobenzene 1p**

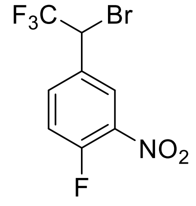

Yellow liquid (1.2 g, 70%). $^{1}$H NMR (400 MHz, $CDCl_3$) δ 8.47 – 8.09 (m, 1 H), 7.95 – 7.73 (m, 1 H), 7.38 (t, *J* = 9.5 Hz, 1 H), 5.19 (q, *J* = 7.1 Hz, 1 H); $^{19}$F NMR (376 MHz,

$CDCl_3$) δ -70.76 (d, *J* = 7.0 Hz, 3 F), -114.44 (ddd, *J* = 10.5, 6.7, 4.1 Hz, 1 F); $^{13}C$ NMR (101 MHz, $CDCl_3$) δ 156.08 (d, *J* = 269.3 Hz), 137.33, 136.08, 130.03, 127.08, 122.89 (q, *J* = 278.4 Hz), 119.41, 44.54 (q, *J* = 35.0 Hz) ppm. MS (EI): 222 (100), 301($M^+$); HRMS (EI): Calcd for $C_8H_4NO_2F_4Br$: 300.9362; Found: 300.9372. IR (KBr): $\nu_{max}$ = 3695, 3075, 2993, 1624, 1544, 1501, 1355, 1298, 1254, 1168, 940, 710, 663 $cm^{-1}$.

**4-(1-Bromo-2,2,2-trifluoroethyl)-3-fluorobenzonitrile 1q**

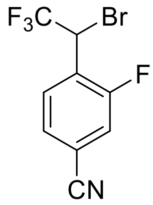


Yellow liquid (1.3 g, 75%). $^1H$ NMR (400 MHz, $CDCl_3$) δ 7.84 (t, *J* = 7.6 Hz, 1 H), 7.56 (d, *J* = 8.2 Hz, 1 H), 7.44 (d, *J* = 9.2 Hz, 1 H), 5.57 (q, *J* = 7.1 Hz, 1 H); $^{19}F$ NMR (376 MHz, $CDCl_3$) δ -70.33 (dd, *J* = 7.0, 2.5 Hz, 3 F), -113.04 – -113.28 (m, 1 F); $^{13}C$ NMR (101 MHz, $CDCl_3$) δ 159.12 (d, *J* = 254.4 Hz), 132.21, 128.77 (d, *J* = 4.1 Hz), 125.91 (d, *J* = 12.5 Hz), 122.88 (q, *J* = 278.4 Hz), 119.57 (d, *J* = 25.8 Hz), 116.63 (d, *J* = 2.8 Hz), 115.54 (d, *J* = 9.9 Hz), 37.08 (qd, *J* = 35.9, 4.8 Hz) ppm. MS (EI): 202 (100), 281 ($M^+$); HRMS (EI): Calcd for $C_9H_4NF_4Br$: 280.9463; Found: 280.9470. IR (KBr): $\nu_{max}$ = 3451, 3098, 2998, 2919, 2845, 2238, 1640, 1573, 1511, 1461, 1418, 1353, 1222, 1118, 951, 668 $cm^{-1}$.

**Methyl 4-(1-bromo-2,2,2-trifluoroethyl)-2-fluorobenzoate 1r**

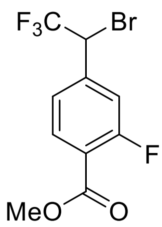


White solid (5.7 g, 57%). Mp: 48-49 °C. $^1H$ NMR (400 MHz, $CDCl_3$) δ 7.95 (t, *J* = 7.8 Hz, 1 H), 7.30 (dd, *J* = 19.1, 16.3 Hz, 2 H), 5.11 (q, *J* = 7.1 Hz, 1 H), 3.93 (s, 3 H); $^{19}F$ NMR (376 MHz, $CDCl_3$) δ -70.32 (d, *J* = 7.1 Hz, 3 F), -107.74 (dd, *J* = 10.8, 7.3 Hz, 1 F); $^{13}C$ NMR (101 MHz, $CDCl_3$) δ 164.00 (d, *J* = 3.8 Hz), 161.52 (d, *J* = 261.9 Hz), 139.17 (d, *J* = 8.6 Hz), 132.70 (d, *J* = 1.2 Hz), 124.70 (d, *J* = 3.4 Hz), 122.99 (q, *J* = 278.4 Hz), 120.15 (d, *J* = 10.3 Hz), 118.06 (d, *J* = 24.9 Hz), 52.59, 45.20 (q, *J* = 34.7

Hz) ppm. MS (EI): 235 (100), 314($M^+$); HRMS (EI): Calcd for $C_{10}H_7O_2F_4Br$: 313.9566; Found: 313.9565. IR (KBr): $\nu_{max}$ = 3444, 3002, 2956, 2847, 2396, 1732, 1625, 1504, 1439, 1254, 1170, 1113, 959, 773, 739 $cm^{-1}$.

**2-(1-Bromo-2,2,2-trifluoroethyl)naphthalene 1s**

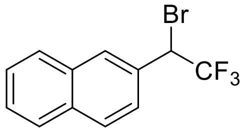


White solid (2.5 g, 63%). Mp: 70-72 °C. $^1$H NMR (400 MHz, $CDCl_3$) δ 7.94 (s, 1 H), 7.88 (dd, $J$ = 12.5, 6.9 Hz, 3 H), 7.63 (d, $J$ = 8.5 Hz, 1 H), 7.60 – 7.50 (m, 2 H), 5.31 (tt, $J$ = 7.4, 3.7 Hz, 1 H); $^{19}$F NMR (376 MHz, $CDCl_3$) δ -70.09 (t, $J$ = 6.6 Hz, 3 F); $^{13}$C NMR (101 MHz, $CDCl_3$) δ 133.76, 132.75, 130.03, 129.10, 129.02, 128.28, 127.77, 127.44, 126.92, 125.63, 123.52 (q, $J$ = 279.77 Hz), 47.52 (q, $J$ = 34.1Hz) ppm. MS (EI): 209 (100), 288 ($M^+$); HRMS (EI): Calcd for $C_{12}H_8F_3Br$: 287.9761; Found: 287.9767. IR (KBr): $\nu_{max}$ = 3063, 2974, 1827, 1598, 1508, 1370, 1319, 1261, 1176, 1107, 1015, 870, 820, 752 $cm^{-1}$.

**1-(1-Bromo-2,2,2-trifluoroethyl)-4-(tert-butyl)benzene 1t**

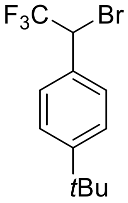


Yellow liquid (980 mg, 65%). $^1$H NMR (400 MHz, $CDCl_3$) δ 7.43 (d, $J$ = 8.8 Hz, 2 H), 7.40 (d, $J$ = 8.8 Hz, 2 H), 5.12 (q, $J$ = 7.5 Hz, 1 H), 1.33 (s, 9 H); $^{19}$F NMR (376 MHz, $CDCl_3$) δ -70.46 (d, $J$ = 7.5 Hz); $^{13}$C NMR (101 MHz, $CDCl_3$) δ 153.27, 129.73, 128.80, 125.87, 123.48 (q, $J$ = 278.0 Hz), 47.07 (q, $J$ = 34.1 Hz), 34.76, 31.17 ppm. MS (EI): 265 (100), 294 ($M^+$); HRMS (EI): Calcd for $C_{12}H_{14}F_3Br$: 294.0231; Found: 294.0221. IR (KBr):$\nu_{max}$ = 2964, 1612, 1256, 1158, 1108, 818, 668, 557 $cm^{-1}$.

**1-(1-Bromo-2,2,2-trifluoroethyl)-4-methoxybenzene 1u**

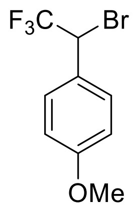


$^{1}$H NMR (400 MHz, $CDCl_3$) δ 7.44 (d, *J* = 8.6 Hz, 2 H), 6.91 (d, *J* = 8.8 Hz, 2 H), 5.12 (q, *J* = 7.4 Hz, 1 H), 3.83 (s, 3 H); $^{19}$F NMR (376 MHz, $CDCl_3$) δ -70.68 (d, *J* = 7.4 Hz); $^{13}$C NMR (101 MHz, $CDCl_3$) δ 160.77, 130.48, 124.84, 123.46 (q, *J* = 277.8 Hz), 114.27, 55.3, 47.05 (q, *J* = 34.1 Hz) ppm. MS (EI): 189 (100), 268 ($M^+$); HRMS (EI): Calcd for $C_9H_8OF_3Br$: 267.9711; Found: 267.9717. IR (KBr): $\nu_{max}$ = 2962, 2840, 1611, 1514, 1251, 1157, 1106, 1031, 821, 670 $cm^{-1}$.

**General procedure for the preparation of $CF_2H$-substituted secondary benzylic bromides**[1]

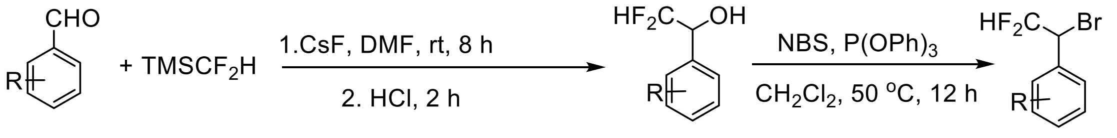


In a Schlenk flask was added methyl 4-formylbenzoate (5.00 g, 30.5 mmol), CsF (595 mg, 4.00 mmol) and anhydrous DMF (50.0 mL) were added under an argon atmosphere. Difluoromethyltrimethylsilane ($TMSCF_2H$) (9.5 mL, 61 mmol) was then added dropwise over 20 min. The mixture was stirred at room temperature for 8 h. An aqueous solution of HCl (60.0 mL, 1.0 N) was added, and the mixture was stirred for another 2 h. The mixture was then extracted with ethyl acetate (3 × 50 mL), and the combined organic layers were dried over anhydrous $Na_2SO_4$. The solvent was removed under vacuum and the crude product was used without further purification.

Difluoromethylated alcohol obtained from the previous step was dissolved in dichloromethane (50.0 mL), and NBS (7.82 g, 44.1 mmol) was added. To the solution was added triphenyl phosphite (13.5 g, 44.1 mmol) and the resulting mixture was stirred at 50 °C for 12 h. The mixture was then cooled to room temperature and the solvent was evaporated in vacuo. The residue was purified by column chromatography on silica gel with a gradient eluent of petroleum ether and ethyl acetate to give compound **1v-1aa**.

**4-(1-Bromo-2,2-difluoroethyl)-3-fluorobenzonitrile 1v**

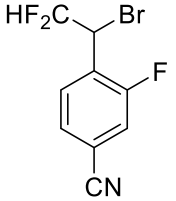


Yellow liquid (2.05 g, 25% yield). $^1$H NMR (400 MHz, $CDCl_3$) δ 7.72 (t, $J$ = 7.5 Hz, 1 H), 7.53 (d, $J$ = 8.0 Hz, 1 H), 7.42 (d, $J$ = 9.4 Hz, 1 H), 6.12 (td, $J$ = 55.4, 4.1 Hz, 1 H), 5.31 (td, $J$ = 11.6, 4.0 Hz, 1 H); $^{19}$F NMR (376 MHz, $CDCl_3$) δ -112.56 (dd, $J$ = 9.1, 7.1 Hz), -116.89 (dddd, $J$ = 279.1, 55.3, 10.9, 3.3 Hz), -118.88 (dddd, $J$ = 279.2, 55.8, 12.3,

1.6 Hz); $^{13}C$ NMR (101 MHz, $CDCl_3$) δ 159.41 (d, *J* = 253.7 Hz), 132.39, 128.64 (d, *J* = 4.1 Hz), 127.13 (d, *J* = 13.0 Hz), 119.61 (d, *J* = 25.7 Hz), 116.78 (d, *J* = 2.7 Hz), 115.01 (d, *J* = 9.9 Hz), 113.30 (t, *J* = 248.9 Hz), 40.29 (t, *J* = 26.5 Hz) ppm. MS (EI): 184 (100), 263 ($M^+$); HRMS (EI): Calcd for $C_9H_5NF_3Br$: 262.9557; Found: 262.9561. IR(KBr): $\nu_{max}$ = 3077, 2237, 1750, 1619, 1590, 1457, 1417, 1196, 1105, 950 $cm^{-1}$.

**Methyl 4-(1-bromo-2,2-difluoroethyl)benzoate 1w**

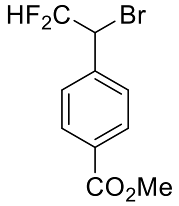


Yellow liquid (3.8 g, 45% yield). $^1H$ NMR (400 MHz, $CDCl_3$) δ 8.03 (d, *J* = 8.2 Hz, 2 H), 7.51 (d, *J* = 8.3 Hz, 2 H), 6.03 (td, *J* = 55.6, 4.2 Hz, 1 H), 4.98 (td, *J* = 11.7, 4.2 Hz, 1 H), 3.91 (s, 3 H); $^{19}F$ NMR (376 MHz, $CDCl_3$) δ -117.20 (ddd, *J* = 276.8, 55.5, 11.5 Hz), -118.44 (ddd, *J* = 276.8, 55.6, 11.8 Hz); $^{13}C$ NMR (101 MHz, $CDCl_3$) δ 166.25, 138.61, 131.21, 130.07, 129.14, 113.92 (t, *J* = 247.3 Hz), 52.34, 48.85 (t, *J* = 25.1 Hz) ppm. MS (EI): 199 (100), 278 ($M^+$); HRMS (EI): Calcd for $C_{10}H_9O_2F_2Br$: 277.9754; Found: 277.9760. IR (KBr): $\nu_{max}$ = 2997, 2954, 1719, 1612, 1436,1283, 1108, 1066, 1020, 727 $cm^{-1}$.

**2-(1-Bromo-2,2-difluoroethyl)naphthalene 1x**

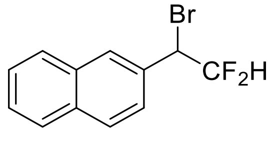


Yellow liquid (1.2 g, 75% yield). $^1H$ NMR (400 MHz, $CDCl_3$) δ 7.98 – 7.72 (m, 4 H), 7.62 – 7.41 (m, 3 H), 6.14 (td, *J* = 55.7, 4.3 Hz, 1 H), 5.16 (td, *J* = 11.6, 4.3 Hz, 1 H); $^{19}F$ NMR (376 MHz, $CDCl_3$) δ -116.97 (ddd, *J* = 275.6, 55.7, 11.3 Hz), -117.90 (ddd, *J* = 275.6, 55.6, 11.9 Hz); $^{13}C$ NMR (101 MHz, $CDCl_3$) δ 133.58, 132.90, 131.35, 128.99, 128.67, 128.19, 127.77, 127.21, 126.84, 125.73, 114.24 (t, *J* = 247.0 Hz), 50.37 (t, *J* = 24.9 Hz) ppm. MS (EI): 191 (100), 270 ($M^+$); HRMS (EI): Calcd for $C_{12}H_2F_2Br$: 269.9856; Found: 296.9860. IR (KBr): $\nu_{max}$ = 3444, 3062, 2969, 1660, 1598, 1363, 1109, 1071, 1041, 966, 792 $cm^{-1}$.

**1-(1-Bromo-2,2-difluoroethyl)-4-chlorobenzene 1y**

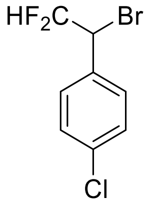


Yellow liquid (642 mg, 42% yield). $^{1}$H NMR (400 MHz, $CDCl_3$) δ 7.46 – 7.30 (m, 4 H), 6.01 (td, $J$ = 55.6, 4.1 Hz, 1 H), 4.94 (td, $J$ = 11.8, 4.1 Hz, 1 H); $^{19}$F NMR (376 MHz, $CDCl_3$) δ -117.14 (ddd, $J$ = 276.2, 55.6, 11.5 Hz), -118.75 (ddd, $J$ = 276.3, 55.7, 12.2 Hz); $^{13}$C NMR (101 MHz, $CDCl_3$) δ 135.64, 132.45, 130.38, 129.14, 113.93 (t, $J$ = 247.3 Hz), 48.84 (t, $J$ = 25.0 Hz) ppm. MS (EI): 175 (100), 256 ($M^+$); HRMS (EI): Calcd for $C_8H_6F_2ClBr$: 253.9309; Found: 253.9312. IR (KBr): $\nu_{max}$ = 2977, 1902, 1597, 1493, 1411, 1134, 1092, 1016, 849 $cm^{-1}$.

**1-Bromo-4-(1-bromo-2,2-difluoroethyl)benzene 1z**

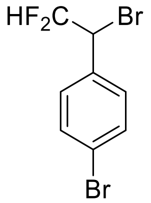


Yellow liquid (1.2g, 67% yield). $^{1}$H NMR (400 MHz, $CDCl_3$) δ 7.53 (d, $J$ = 8.5 Hz, 2 H), 7.33 (d, $J$ = 8.4 Hz, 2 H), 6.00 (td, $J$ = 55.6, 4.1 Hz, 1 H), 4.92 (td, $J$ = 11.8, 4.1 Hz, 1 H); $^{19}$F NMR (376 MHz, $CDCl_3$) δ -117.13 (ddd, $J$ = 276.4, 55.6, 11.5 Hz), -118.76 (ddd, $J$ = 276.3, 55.7, 12.2 Hz); $^{13}$C NMR (101 MHz, $CDCl_3$) δ 132.95, 132.11, 130.64, 123.85, 113.86 (t, $J$ = 247.3 Hz), 48.87 (t, $J$ = 25.0 Hz) ppm. MS (EI): 140 (100), 298 ($M^+$); HRMS (EI): Calcd for $C_8H_6F_2Br_2$: 297.8804; Found: 297.8806. IR (KBr): $\nu_{max}$ = 3502, 3075, 2977, 1772, 1591, 1490, 1456, 1407, 1074, 1012, 848, 745, 560 $cm^{-1}$.

**1-(1-Bromo-2,2-difluoroethyl)-2-fluoro-4-methylbenzene 1aa**

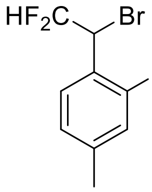


Yellow liquid (590 mg, 33% yield). $^1$H NMR (400 MHz, $CDCl_3$) δ 7.41 (t, *J* = 7.8 Hz, 1 H), 7.01 (d, *J* = 8.0 Hz, 1 H), 6.91 (d, *J* = 11.2 Hz, 1 H), 6.11 (tdd, *J* = 55.8, 4.7, 0.6 Hz, 1 H), 5.29 (ddd, *J* = 12.3, 10.4, 4.7 Hz, 1 H), 2.36 (s, 3 H); $^{19}$F NMR (376 MHz, $CDCl_3$) δ -116.72 (dddd, *J* = 276.1, 55.8, 12.4, 2.6 Hz), -116.98 (d, *J* = 10.8 Hz), -118.39 (dddd, *J* = 276.1, 55.8, 12.4, 2.6 Hz); $^{13}$C NMR (101 MHz, $CDCl_3$) δ 159.83 (d, *J* = 249.4 Hz), 142.42 (d, *J* = 8.3 Hz), 130.43, 125.62 (d, *J* = 3.1 Hz), 118.65 (d, *J* = 13.7 Hz), 116.36 (d, *J* = 21.5 Hz), 113.83 (dt, *J* = 246.5, 3.2 Hz), 42.45 (td, *J* = 26.3, 3.0 Hz), 21.22 (d, *J* = 1.6 Hz) ppm. MS (EI): 173 (100), 252 ($M^+$); HRMS (EI): Calcd for $C_9H_8F_3Br$: 251.9761; Found: 251.9768. IR (KBr): $\nu_{max}$ = 2980, 2926, 1627, 1578, 1423, 1268, 1142, 1104, 950 $cm^{-1}$.

**General procedure for the preparation of $CFH_2$-substituted secondary benzylic bromides[2]**

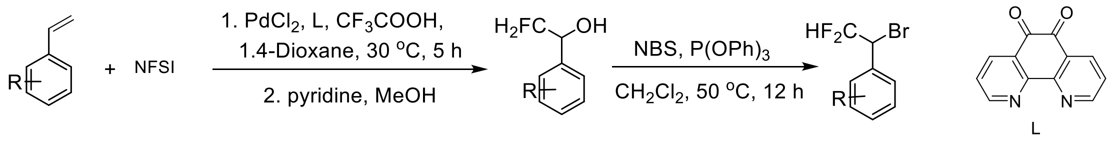


In a dried tube was added $PdCl_2$ (27.0 mg, 0.150 mmol), 1,10-phenanthroline-5,6-quinone (46 mg, 0.23 mmol, 7.5 mol %), NFSI (285 mg, 0.900 mmol) and styrene (486 mg, 3.00 mmol) and 1,4-dioxane (8.0 mL). To the mixture was added $CF_3CO_2H$ (1.1 mL,15.0 mmol) was added. The reaction mixture was stirred at 30 °C for 3-5 h. Methanol (5.0 mL) and 5 drops of wet pyridine were added. The mixture was further stirred for another 1.0 h. The mixture was extracted with ethyl acetate (3 × 50 mL), and the combined organic layers were dried over anhydrous $Na_2SO_4$. The solvent was removed under vacuum and the crude product was used without further purification.

Monofluoromethylated alcohol obtained from the previous step was dissolved in dichloromethane (10.0 mL) and NBS (782 mg, 4.41 mmol) was added under an argon atmosphere. Triphenyl phosphite (1.35 g, 4.41 mmol) was added and the resulting mixture was stirred at 50 °C for 12 h. The mixture was then cooled to room temperature and the solvent was evaporated in vacuo. The residue was purified by column chromatography on silica gel with a gradient eluent of petroleum ether and ethyl acetate to give compound **1ab-ad**.

**Methyl 4-(1-bromo-2-fluoroethyl)benzoate 1ab**

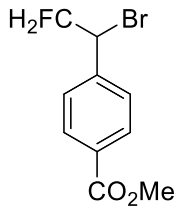


Yellow liquid (335 mg, 43% yield). $^1$H NMR (400 MHz, $CDCl_3$) δ 8.04 (d, *J* = 8.4 Hz, 2 H), 7.51 (d, *J* = 8.3 Hz, 2 H), 5.12 (dt, *J* = 11.1, 6.9 Hz, 1 H), 4.85 (ddt, *J* = 17.3, 11.6, 8.6 Hz, 1 H), 4.73 (ddt, *J* = 17.3, 11.6, 8.7 Hz, 1 H), 3.92 (s, 3 H); $^{19}$F NMR (282 MHz, $CDCl_3$) δ -205.10 (td, *J* = 47.3, 11.9 Hz); $^{13}$C NMR (101 MHz, $CDCl_3$) δ 166.36, 142.01 (d, *J* = 2.8 Hz), 130.80, 130.17, 128.11, 84.33 (d, *J* = 180.5 Hz), 52.32, 48.42 (d, *J* =

22.3 Hz) ppm. MS (EI): 181 (100), 300 ($M^+$); HRMS (EI): Calcd for $C_{10}H_{10}O_2FBr$: 259.9848; Found: 259.9853. IR (KBr): $\nu_{max}$ = 2958, 1942, 1717, 1610, 1434, 1315, 1284, 1111, 1005, 714, 684 $cm^{-1}$

**1-(1-Bromo-2-fluoroethyl)-4-fluorobenzene 1ac**

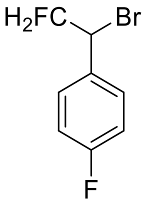


Yellow liquid (400 mg, 60% yield). $^1$H NMR (400 MHz, $CDCl_3$) δ 7.43 (dd, $J$ = 8.5, 5.3 Hz, 2 H), 7.07 (t, $J$ = 8.6 Hz, 2 H), 5.11 (dt, $J$ = 11.1, 6.9 Hz, 1 H), 4.82 (ddd, $J$ = 17.2, 9.8, 7.0 Hz, 1 H), 4.71 (ddd, $J$ = 17.2, 9.8, 7.0 Hz, 1 H); $^{19}$F NMR (376 MHz, $CDCl_3$) δ -111.86 – -111.95, -204.19 (td, $J$ = 46.8, 11.1 Hz); $^{13}$C NMR (101 MHz, $CDCl_3$) δ 162.90 (d, $J$ = 248.9 Hz), 133.20 (t, $J$ = 3.2 Hz), 129.87 (d, $J$ = 8.5 Hz), 115.97 (d, $J$ = 21.9 Hz), 84.57 (d, $J$ = 180.1 Hz), 48.81 (d, $J$ = 22.2 Hz) ppm. MS (EI): 141 (100), 220 ($M^+$); HRMS (EI): Calcd for $C_8H_7F_2Br$: 219.9699; Found: 219.9692. IR (KBr): $\nu_{max}$ = 3046, 2968, 1893, 1605, 1510, 1470, 1233, 1160, 1015, 857, 783 $cm^{-1}$.

**1-(1-Bromo-2-fluoroethyl)-4-chlorobenzene 1ad**

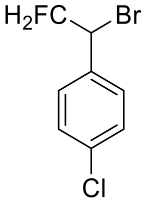


Yellow liquid (390 mg, 55% yield). $^1$H NMR (300 MHz, $CDCl_3$) δ 7.42 – 7.31 (m, 4 H), 5.08 (dt, $J$ = 11.0, 7.2 Hz, 1 H), 4.84 (qd, $J$ = 9.7, 7.1 Hz, 1 H), 4.68 (qd, $J$ = 9.8, 7.2 Hz, 1 H); $^{19}$F NMR (282 MHz, $CDCl_3$) δ -204.69 (td, $J$ = 47.1, 11.5 Hz); $^{13}$C NMR (101 MHz, $CDCl_3$) δ 135.82, 135.03, 129.30 (d, $J$ = 23.5 Hz), 129.18, 84.44 (d, $J$ = 180.3 Hz), 48.64 (d, $J$ = 21.4 Hz) ppm. MS (EI): 157 (100), 236 ($M^+$); HRMS (EI): Calcd for $C_8H_7FClBr$: 235.9404; Found: 235.9407. IR (KBr): $\nu_{max}$ = 2974, 1900, 1493, 1411, 1093, 1014, 830, 732, 684 $cm^{-1}$.

**General procedure for the preparation of ligands**

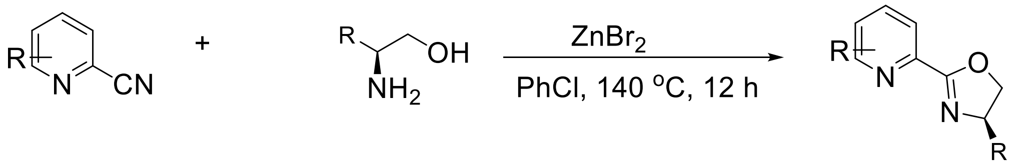


In a 100 mL flask, $ZnBr_2$ (281 mg, 1.25 mmol) was heated at 200 °C under vacuum for 30 min and then cooled to room temperature under an argon atmosphere. Chlorobenzene (30.0 mL) was added, followed by 2-cyanopydine (1.45 g, 6.75 mmol) and (S)-2-amino-3-cyclohexyl-1-propanol (1.59 g, 10.1 mmol). The resulting mixture was heated at 140 °C for 24 h. The mixture was allowed to cool to room temperature, and concentrated under reduced pressure. The residue was dissolved in $CH_2Cl_2$ (100 mL), and the resulting solution was transferred to a separatory funnel and washed with water (200 mL). The aqueous layer was extracted with $CH_2Cl_2$ (3 × 100 mL), and the combined organic layers were washed with brine, dried over anhydrous $Na_2SO_4$, and concentrated under reduced pressure. The residue was purified by column chromatography on silica gel using a mixture of pentane/ethyl acetate as the eluent to give the ligand.

**(R)-2-(3-Bromo-5-chloropyridin-2-yl)-4-(cyclohexylmethyl)-4,5-dihydrooxazole L2**

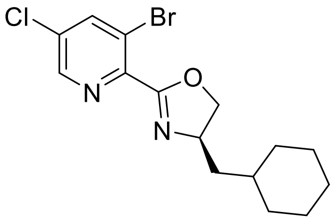

White solid (1.6 g, 54%). Mp: 36-38 °C. $^1$H NMR (400 MHz, $CDCl_3$) δ 8.57 (dd, *J* = 2.1, 0.8 Hz, 1 H), 8.01 (dd, *J* = 2.1, 0.8 Hz, 1 H), 4.60 – 4.53 (m, 1 H), 4.52 – 4.42 (m, 1 H), 4.07 (t, *J* = 7.7 Hz, 1 H), 1.84 – 1.62 (m, 6 H), 1.53 (dtd, *J* = 10.7, 7.1, 3.5 Hz, 1H), 1.44 (dt, *J* = 13.7, 7.0 Hz, 1 H), 1.30 – 1.13 (m, 3 H), 0.97 (q, *J* = 12.1 Hz, 2 H); $^{13}$C NMR (101 MHz, $CDCl_3$) δ 160.35, 146.87, 145.13, 140.95, 133.26, 120.21, 73.66, 65.48, 44.00, 34.89, 33.45, 26.50, 26.20 ppm. MS (EI): 138 (100), 356 ($M^+$); HRMS (EI): Calcd for $C_{11}H_{12}N_2OClBr$: 356.0291; Found: 356.0302. IR (KBr): $\nu_{max}$ =2921, 2849, 1675, 1560, 1444, 1351, 1112, 1032, 956, 803cm$^{-1}$.

**(R)-4-(Cyclohexylmethyl)-2-(5-methoxypyridin-2-yl)-4,5-dihydrooxazole L3**

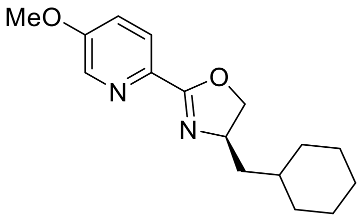


White solid (360 mg, 36%). Mp: 66-68 °C. $^1$H NMR (400 MHz, $CDCl_3$) δ 8.36 (d, *J* = 2.4 Hz, 1 H), 7.97 (d, *J* = 8.7 Hz, 1 H), 7.21 (dd, *J* = 8.7, 2.9 Hz, 1 H), 4.56 (t, *J* = 8.7 Hz, 1 H), 4.40 (dd, *J* = 16.0, 7.9 Hz, 1 H), 4.02 (t, *J* = 8.2 Hz, 1 H), 3.90 (s, 3 H), 1.56-1.73 (m, 6 H), 1.56 – 1.43 (m, 1 H), 1.43 – 1.30 (m, 1 H), 1.23 (m, 3 H), 0.95 (m, 2 H); $^{13}$C NMR (101 MHz, $CDCl_3$) δ 162.19, 157.06, 139.35, 137.72, 124.75, 120.10, 73.69, 64.76, 55.72, 44.25, 33.49, 26.54, 26.22, 26.20 ppm. MS (EI): 177 (100), 274 ($M^+$); HRMS (EI): Calcd for $C_{16}H_{22}N_2O_2$: 274.1681; Found: 274.1689. IR (KBr): $\nu_{max}$ = 3385, 2921, 2849, 1641, 1588, 1518, 1494, 1362, 1272, 1232, 1091, 1025, 960, 845, 683 $cm^{-1}$.

**(R)-4-(Cyclohexylmethyl)-2-(5-(trifluoromethyl)pyridin-2-yl)-4,5-dihydrooxazole L4**

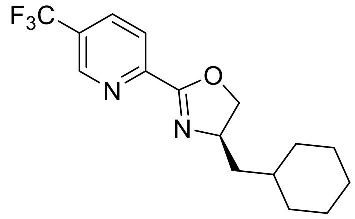


White solid (471 mg, 52%). Mp: 75-77 °C. $^1$H NMR (400 MHz, $CDCl_3$) δ 8.94 (s, 1 H), 8.16 (d, *J* = 8.2 Hz, 1 H), 8.01 (d, *J* = 8.3, 1 H), 4.62 (dd, *J* = 9.4, 8.3 Hz, 1 H), 4.55 – 4.38 (m, 1 H), 4.09 (t, *J* = 8.3 Hz, 1 H), 1.85 – 1.64 (m, 6 H), 1.45-1.55 (m, 1 H), 1.46 – 1.35 (m, 1 H), 1.31 – 1.13 (m, 3 H), 0.9 (qd, *J* = 12.3, 3.0 Hz, 2 H); $^{19}$F NMR (376 MHz, $CDCl_3$) δ -62.60 (s, 3 F); $^{13}$C NMR (101 MHz, $CDCl_3$) δ 161.46, 150.04, 146.60 (q, *J* = 4.0 Hz), 133.90 (q, *J* = 3.4 Hz), 127.96 (q, *J* = 33.3 Hz) 123.59, 123.14 (q, *J* = 273.7 Hz), 74.08 , 65.11, 44.08, 34.81, 33.45, 26.49, 26.17 ppm. MS (EI): 138 (100), 312 ($M^+$); HRMS (EI): Calcd for $C_{16}H_{19}N_2OF$: 312.1449; Found: 312.1448. IR (KBr):

$\nu_{max}$ = 3258, 3043, 2925, 2662, 1878, 1637,1604, 1446, 1329, 1127, 1014, 971, 687 cm$^{-1}$.

**(S)-2-(3-Bromo-5-chloropyridin-2-yl)-4-neopentyl-4,5-dihydrooxazole L7**

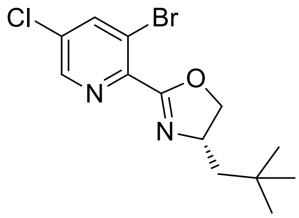


Yellow liquid (750 mg, 45% yield). $^1$H NMR (400 MHz, CDCl$_3$) δ 8.67 – 8.42 (s, 1 H), 8.12 – 7.84 (s, 1 H), 4.67 – 4.46 (m, 1 H), 4.51 – 4.34 (m, 1 H), 4.13 – 3.85 (m, 1 H), 2.00 – 1.81 (m, 1 H), 1.59 – 1.34 (m, 1 H), 0.98 (s, 9 H); $^{13}$C NMR (101 MHz, CDCl$_3$) δ 159.99, 146.82, 144.94, 141.03, 133.22, 120.23, 74.74, 65.00, 50.37, 30.35, 29.96 ppm. MS (EI): 112.1 (100), 330 (M$^+$); HRMS (EI): Calcd for $C_{13}H_{16}N_2OClBr$: 330.0135; Found: 330.0140. IR (KBr): $\nu_{max}$ = 3046, 2954, 2866, 1674, 1560, 1473, 1394, 1365, 1113, 1031, 936, 804 cm$^{-1}$.

**(3aS,8aR)-2-(6-*iso*propylpyridin-2-yl)-8,8a-dihydro-3aH-indeno[1,2-d]oxazole L8**

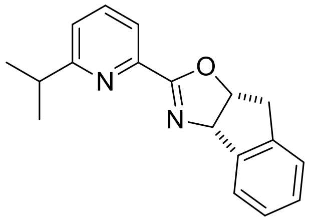


Yellow liquid (430 mg, 31% yield). $^1$H NMR (400 MHz, CDCl$_3$) δ 7.82 (d, *J* = 7.7 Hz, 1 H), 7.63 (t, *J* = 7.8 Hz, 1 H), 7.57 (d, *J* = 4.5 Hz, 1 H), 7.14 (dd, *J* = 80.4, 5.8 Hz, 4 H), 5.77 (d, *J* = 8.0 Hz, 1 H), 5.61 – 5.44 (m, 1 H), 3.53 – 3.36 (m, 2 H), 3.19 (dt, *J* = 13.9, 6.9 Hz, 1 H), 1.41 – 1.07 (m, 6 H); $^{13}$C NMR (101 MHz, CDCl$_3$) δ 167.93, 163.55, 146.06, 141.73, 139.92, 136.86, 128.53, 127.44, 125.75, 125.30, 121.90, 121.80, 83.87, 39.88, 36.59, 22.88, 22.78 ppm. MS (EI): 104 (100), 278 (M$^+$); HRMS (EI): Calcd for $C_{18}H_{18}N_2FO$: 278.1419; Found: 278.1418. IR (KBr): $\nu_{max}$ = 3068, 2962, 1633, 1588, 1470, 1384, 1363, 1118, 1021, 746 cm$^{-1}$.

**(R)-2-(3-Bromo-5-chloropyridin-2-yl)-4-isopropyl-4,5-dihydrooxazole L9**

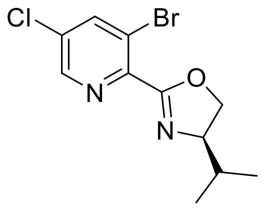


Yellow liquid (280 mg, 40%). $^{1}$H NMR (400 MHz, $CDCl_3$) δ 8.56 (d, $J$ = 2.1 Hz, 1 H), 8.01 (d, $J$ = 2.0 Hz, 1 H), 4.61 – 4.35 (m, 1 H), 4.35 – 4.15 (m, 2 H), 1.97 – 1.85 (m, 1 H), 1.05 (d, $J$ = 6.8 Hz, 3 H), 0.97 (d, $J$ = 6.8 Hz, 3 H); $^{13}$C NMR (101 MHz, $CDCl_3$) δ 160.41, 146.83, 145.07, 140.98, 133.23, 120.20, 73.52, 70.70, 32.69, 18.88, 18.36 ppm. MS (EI): 261 (100), 302 ($M^+$); HRMS (EI): Calcd for $C_{11}H_{12}N_2OClBr$: 301.9822; Found: 301.9824. IR (KBr): $\nu_{max}$ = 3046, 2958, 2871, 1675, 1560, 1534, 1467, 1440, 1363, 1348, 1204, 1116, 1032, 912, 806 cm$^{-1}$.

**(R)-2-(3-Bromo-5-chloropyridin-2-yl)-4-phenyl-4,5-dihydrooxazole L10**

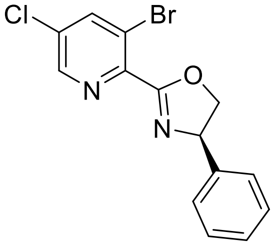


White solid (420 mg, 53%). Mp: 67-69 $^{o}$C. $^{1}$H NMR (400 MHz, $CDCl_3$) δ 8.61 (d, $J$ = 2.1 Hz, 1 H), 8.06 (d, $J$ = 2.1 Hz, 1 H), 7.45 – 7.25 (m, 5 H), 5.54 (dd, $J$ = 10.3, 8.7 Hz, 1 H), 4.89 (dd, $J$ = 10.3, 8.5 Hz, 1 H), 4.34 (t, $J$ = 8.6 Hz, 1 H); $^{13}$C NMR (101 MHz, $CDCl_3$) δ 161.77, 146.94, 144.72, 141.48, 141.14, 133.55, 128.82, 127.80, 126.78, 120.44, 75.15, 70.94 ppm. MS (EI): 308 (100), 336 ($M^+$); HRMS (EI): Calcd for $C_{14}H_{10}N_2OClBr$: 335.9665; Found: 335.9666. IR (KBr): $\nu_{max}$ = 3058, 3030, 2963, 2898, 1668, 1559, 1472, 1439, 1351, 1115, 1031, 949, 806, 699, 535 cm$^{-1}$.

**(R)-4-Benzyl-2-(3-bromo-5-chloropyridin-2-yl)-4,5-dihydrooxazole L11**

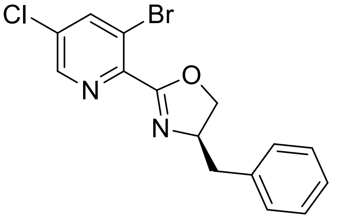


.

White solid (320 mg, 38%). Mp: 94-96 °C. $^1$H NMR (400 MHz, CDCl$_3$) δ 8.60 (d, *J* = 2.1 Hz, 1 H), 8.05 (d, *J* = 2.1 Hz, 1 H), 7.39 – 7.22 (m, 5 H), 4.74 (dddd, *J* = 13.1, 8.8, 7.7, 5.4 Hz, 1 H), 4.46 (dd, *J* = 9.4, 8.7 Hz, 1 H), 4.25 (dd, *J* = 8.5, 7.7 Hz, 1 H), 3.32 (dd, *J* = 13.8, 5.4 Hz, 1 H), 2.83 (dd, *J* = 13.8, 8.7 Hz, 1 H); $^{13}$C NMR (101 MHz, CDCl$_3$) δ 161.02, 146.87, 144.73, 141.09, 137.58, 133.41, 129.29, 128.65, 126.67, 120.27, 72.28, 68.74, 41.50 ppm. MS (EI): 261 (100), 350 (M$^+$); HRMS (EI): Calcd for $C_{15}H_{12}N_2OClBr$: 349.9822; Found: 349.9824. IR (KBr): $\nu_{max}$ = 3059, 3027, 2921, 1672, 1582, 1473, 1350, 1204, 1111, 1072, 1030, 957, 701, 525 cm$^{-1}$.

**(R)-2-(3-Bromo-5-chloropyridin-2-yl)-4-(tert-butyl)-4,5-dihydrooxazole L12**

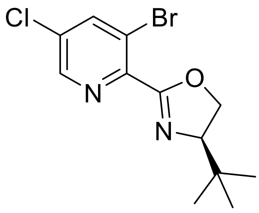


White solid (510 mg, 69%). Mp: 56-58 °C. $^1$H NMR (400 MHz, CDCl$_3$) δ 8.56 (d, *J* = 2.1 Hz, 1 H), 8.01 (d, *J* = 2.1 Hz, 1 H), 4.44 (dd, *J* = 10.2, 8.6 Hz, 1 H), 4.28 (t, *J* = 8.5 Hz, 1 H), 4.18 (dd, *J* = 10.2, 8.4 Hz, 1 H), 1.00 (s, 9 H); $^{13}$C NMR (101 MHz, CDCl$_3$) δ 160.34, 146.83, 145.13, 140.97, 133.21, 120.25, 77.25, 69.23, 33.97, 25.99 ppm. MS (EI): 262 (100), 318 (M$^+$); HRMS (EI): Calcd for $C_{12}H_{14}N_2OClBr$: 315.9978; Found: 301.9972. IR (KBr): $\nu_{max}$ = 3064, 2987, 2903, 1683, 1559, 1467, 1351, 1205, 1107, 1032, 957, 880, 804, 648 cm$^{-1}$.

**(R)-2-(5-Chloro-3-methylpyridin-2-yl)-4-(cyclohexylmethyl)-4,5-dihydrooxazole L13**

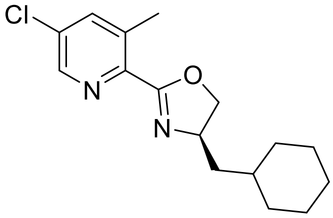


Yellow liquid (260 mg, 33%). $^1$H NMR (400 MHz, CDCl$_3$) δ 8.47 (d, *J* = 2.0 Hz, 1 H), 7.59 (d, *J* = 1.8 Hz, 1 H), 4.50 (t, *J* = 7.0 Hz, 1 H), 4.48– 4.39 (m, 1 H), 4.00 (t, *J* = 7.7 Hz, 1 H), 2.60 (s, 3 H), 1.82 – 1.66 (m, 6 H), 1.45– 1.55 (m, *J* = 14.3, 1 H), 1.45 – 1.36 (m, 1 H), 1.28 – 1.14 (m, 3 H), 1.02 – 0.90 (m, 2 H); $^{13}$C NMR (101 MHz, CDCl$_3$) δ

161.48, 145.64 , 143.95, 138.60, 136.47, 132.90, 72.76, 65.56, 44.27, 35.07, 33.49, 26.51, 26.23, 20.44 ppm. MS (EI): 138 (100), 292 ($M^+$); HRMS (EI): Calcd for $C_{16}H_{21}N_2OCl$: 292.1342; Found: 292.1339. IR (KBr): $\nu_{max}$ = 3280, 2922, 2850, 1640, 1575, 1448, 1425, 1354, 1222, 1067, 917, 684 $cm^{-1}$.

**(R)-4-(Cyclohexylmethyl)-2-(3,5-dichloropyridin-2-yl)-4,5-dihydrooxazole L14**

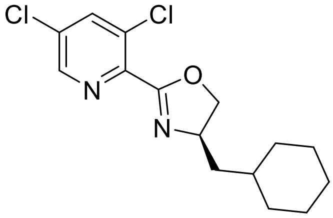


Yellow liquid (290 mg, 53%). $^1H$ NMR (400 MHz, $CDCl_3$) δ 8.51 (s, 1 H), 7.80 (s, 1 H), 4.53 (qd, $J$ = 9.4, 1.6 Hz, 1 H), 4.50 – 4.40 (m, 1 H), 4.04 (td, $J$ = 7.8, 1.6 Hz, 1 H), 1.84 – 1.62 (m, 6 H), 1.51 (ddd, $J$ = 10.0,8.8, 3.3 Hz, 1 H), 1.41 (ddd, $J$ = 14.6, 7.7, 1.3 Hz, 1 H), 1.30 – 1.08 (m, 3 H), 0.94 (q, $J$ = 12.2 Hz, 2 H); $^{13}C$ NMR (101 MHz, $CDCl_3$) δ 159.73, 146.42, 143.33, 137.97, 133.37, 132.17, 73.44, 65.50, 44.00, 34.86, 33.43, 26.48, 26.18 ppm. MS (EI): 138 (100), 312 ($M^+$); HRMS (EI): Calcd for $C_{15}H_{18}N_2OCl_2$: 312.0796; Found: 312.0791. IR (KBr): $\nu_{max}$ = 3280, 3048, 2921, 2849, 2675, 1652, 1563, 1445, 1351, 1111, 1042, 957, 616 $cm^{-1}$.

**(R)-2-(5-Bromo-3-chloropyridin-2-yl)-4-(cyclohexylmethyl)-4,5-dihydrooxazole L15**

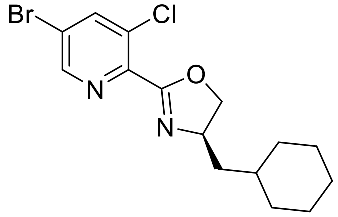


White solid (730 mg, 44%). Mp: 37-39 °C. $^1H$ NMR (400 MHz, $CDCl_3$) δ 8.63 (d, $J$ = 1.9 Hz, 1 H), 7.98 (d, $J$ = 1.9 Hz, 1 H), 4.55 (d, $J$ = 7.8 Hz, 1 H), 4.51 – 4.42 (m, 1 H), 4.06 (t, $J$ = 7.8 Hz, 1 H), 1.84 – 1.64 (m, 6 H), 1.57 – 1.47 (m, 1 H), 1.47 – 1.38 (m, 1 H), 1.24 (ddd, $J$ = 32.3, 17.7, 10.2 Hz, 3 H), 1.04 – 0.89 (m, 2 H); $^{13}C$ NMR (101 MHz, $CDCl_3$) δ 159.83, 148.57, 143.70, 140.74, 132.35, 121.88, 73.46, 65.55, 44.00, 34.88, 33.45, 26.49, 26.20 ppm. MS (EI): 138 (100), 356 ($M^+$); HRMS (EI): Calcd for

$C_{15}H_{18}N_2OClBr$: 356.0291; Found: 356.0300. IR (KBr): $\nu_{max}$ = 2921, 2849, 1647, 1557, 1445, 1372, 1113, 1040, 956, 890, 803 $cm^{-1}$.

**(R)-2-(5-Bromopyridin-2-yl)-4-(cyclohexylmethyl)-4,5-dihydrooxazole L16**

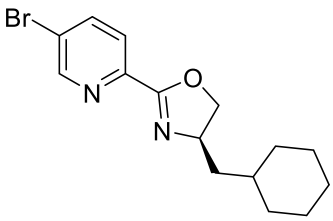


White solid (360 mg, 45%). Mp: 56-58 ºC. $^1H$ NMR (400 MHz, $CDCl_3$) δ 8.74 (dd, $J$ = 1.9, 0.8 Hz, 1 H), 8.01 – 7.79 (m, 2 H), 4.58 (dd, $J$ = 9.4, 8.2 Hz, 1 H), 4.40 (dt, $J$ = 9.3, 7.4 Hz, 1 H), 4.05 (t, $J$ = 8.3 Hz, 1 H), 1.88 – 1.61 (m, 6 H), 1.59 – 1.45 (m, 1 H), 1.40 (dd, $J$ = 13.6, 7.0 Hz, 1 H), 1.35 – 1.11 (m, 3 H), 0.96 (qd, $J$ = 12.1, 2.7 Hz, 2 H); $^{13}C$ NMR (101 MHz, $CDCl_3$) δ 161.79, 150.87, 145.37, 139.27, 125.01, 123.09, 73.95, 65.01, 44.10, 34.81, 33.46, 26.50, 26.18 ppm. MS (EI): 138 (100), 322 ($M^+$); HRMS (EI): Calcd for $C_{15}H_{19}N_2OBr$: 322.0681; Found: 322.0674. IR (KBr): $\nu_{max}$ = 3109, 3042, 2924, 2848, 1637, 1462, 1384, 1233, 1126, 1093, 1005, 862, 678, 630 $cm^{-1}$.

**(R)-2-(5-Chloropyridin-2-yl)-4-(cyclohexylmethyl)-4,5-dihydrooxazole L17**

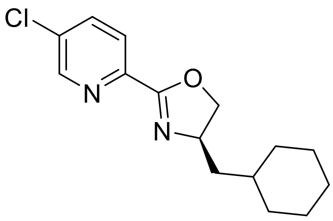


White solid (268 mg, 44%). Mp: 86-88 ºC. $^1H$ NMR (400 MHz, $CDCl_3$) δ 8.64 (d, $J$ = 2.0 Hz, 1 H), 7.98 (d, $J$= 8.4 Hz, 1 H), 7.74 (dd, $J$ = 8.4, 2.4 Hz, 1 H), 4.59 (dd, $J$= 9.4, 8.2 Hz, 1 H), 4.49 – 4.37 (m, 1 H), 4.05 (t, $J$ = 8.3 Hz, 1 H), 1.85 – 1.66 (m, 6 H), 1.53 (dddd, $J$ = 14.2, 10.7, 7.1, 3.4 Hz, 1 H), 1.44 – 1.35 (m, 1 H), 1.29 – 1.13 (m, 3 H), 1.01 – 0.89 (m, 2 H); $^{13}C$ NMR (101 MHz, $CDCl_3$) δ 161.67, 148.68, 145.04, 136.33, 134.11, 124.63, 73.94, 65.00, 44.11, 34.82, 33.46, 26.50, 26.18 ppm. MS (EI): 138 (100), 278 ($M^+$); HRMS (EI): Calcd for $C_{15}H_{19}N_2OCl$: 278.1186;Found: 278.1194. IR (KBr): $\nu_{max}$ = 3117, 3045, 2922, 2849, 1640, 1465, 1447, 1387, 1357, 1125, 1090, 1053, 901, 863, 680 $cm^{-1}$.

**General procedure for Nickel-Catalyzed Asymmetric Suzuki-Miyaura Coupling of trifluoromethylated Secondary Benzyl Bromides**

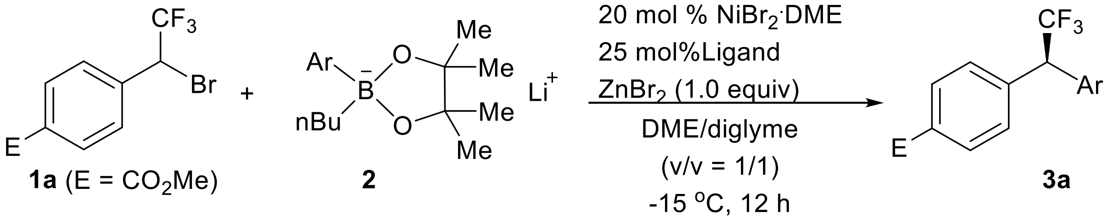


In a glove box, phenyl pinacol boronate ester (5.1 g, 25 mmol) was weighted into a 100 mL Schlenk tube, and 40 mL of dry THF was added. The mixture was taken out from the glove box and cooled at -20 °C. *n*-BuLi (25 mmol, 10 mL, 2.5 M in Hexanes) was added. The mixture was stirred at -20 °C for 2 h. Then the Schlenk tube was taken into the glove box, the solvents were removed under vacuum to give lithium phenyl pinacol boronate.

In an argon-filled glove box, lithium organoboronate (371 mg, 0.900 mmol, 3.00 equiv.), ligand **L2** (26.8 mg, 0.0750 mmol, 0.250 equiv.), $ZnBr_2$ (67.5 mg, 0.300 mmol, 1.00 equiv.) and $NiBr_2$·DME (18.5 mg, 0.0600 mmol, 0.200 equiv.) were placed into a 25 mL Schlenk tube. To this vial was added 5.0 mL of anhydrous DME/diglyme(v/v = 1:1). The Schlenk tube was taken out from the glove box and cooled at -15 °C. α-Bromo-4-methoxycarbonylbenzyl trifluoromethyl **1a** (89.1 mg, 0.300 mmol) was added and the mixture was stirred at-15 °C for 12 h. The mixture was quenched by addition of water (5.0 mL and extracted with $Et_2O$ (10.0 mL × 3). The organic layer was combined, dried over anhydrous $Na_2SO_4$ and concentrated under vacuum. The crude product was purified by column chromatography on silica gel with pentane/ethyl acetate as the eluent to give (S)-methyl 4-(2,2,2-trifluoro-1-phenylethyl)benzoate **3a** as a yellow liquid.

**(S)-Methyl 4-(2,2,2-trifluoro-1-phenylethyl)benzoate 3a**

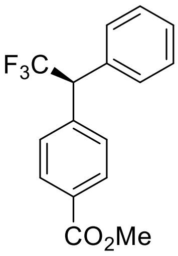

Yellow liquid (68 mg, 77% yield). $^1$H NMR (400 MHz, $CDCl_3$) δ 8.03 (d, $J$ = 8.4 Hz, 2 H), 7.46 (d, $J$ = 8.2 Hz, 2 H), 7.42 – 7.27 (m, 5 H), 4.75 (q, $J$ = 9.8 Hz, 1 H), 3.92 (s, 3 H); $^{19}$F NMR (376 MHz, $CDCl_3$) δ -65.72 (d, $J$ = 9.8 Hz, 3 F); $^{13}$C NMR (101 MHz, $CDCl_3$) δ 166.57, 140.31, 134.67, 129.97, 129.82, 129.14, 129.12, 128.87, 128.23, 125.89 (q, $J$ = 281.79 Hz), 55.46 (q, $J$ = 27.8 Hz), 52.19 ppm. MS (EI): 225 (100), 294 ($M^+$); HRMS (EI): Calcd for $C_{16}H_{13}O_2F_3$: 294.0868; Found: 294.0869. IR (KBr): $\nu_{max}$ = 3034, 2953, 1724, 1615, 1436, 1282, 1261, 1110, 1021, 769 $cm^{-1}$.

HPLC (OJ-H, 0.46 × 25 cm, 5 μm, hexane/isopropanol = 99/1 (v/v %), flow 0.7 mL/min, UV detection at 214 nm), retention time = 15.37 min (minor) and 17.00 min (major). $[\alpha]_D^{25}$ = 22.9650 (c = 0.1800, $CHCl_3$, 96:4 e.r.).

**(R)-Methyl 4-(2,2,2-trifluoro-1-(m-tolyl)ethyl)benzoate 3b**

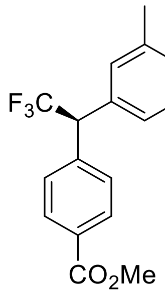


Yellow liquid (52 mg, 56% yield). $^1$H NMR (400 MHz, $CDCl_3$) δ 8.02 (d, $J$ = 8.4 Hz, 2 H), 7.45 (d, $J$ = 8.2 Hz, 2 H), 7.23 (dd, $J$ = 6.7, 3.2 Hz, 1 H), 7.15 (d, $J$ = 8.5 Hz, 3 H), 4.69 (q, $J$ = 9.8 Hz, 1 H), 3.91 (s, 3 H), 2.33 (s, 3 H); $^{19}$F NMR (376 MHz, $CDCl_3$) δ -65.66 (d, $J$ = 9.8 Hz, 3 F); $^{13}$C NMR (101 MHz, $CDCl_3$) δ 166.61, 140.46, 138.62, 134.57, 129.95, 129.85, 129.76, 129.14, 129.00, 128.73, 126.10, 125.93 (q, $J$ = 281.79 Hz), 55.43 (q, $J$ = 27.7 Hz), 52.19, 21.43 ppm. MS (EI): 239 (100), 308 ($M^+$); HRMS (EI): Calcd for $C_{17}H_{15}O_2F_3$: 308.1024; Found: 308.1020. IR (KBr): $\nu_{max}$ = 2953, 2924, 1726, 1608, 1507, 1436, 1319, 1281, 1319, 1110, 1021, 781, 717 $cm^{-1}$.

HPLC (AD-H, 0.46 × 25 cm, 5 μm, hexane/isopropanol = 95/5 (v/v %), flow 0.7 mL/min, UV detection at 214 nm), retention time = 9.02 min (minor) and 10.99 min (major). $[\alpha]_D^{25}$ = 22.9650 (c = 0.1800, $CHCl_3$, 95:5 e.r.).

**(R)-Methyl 4-(2,2,2-trifluoro-1-(3-morpholinophenyl)ethyl)benzoate 3c**

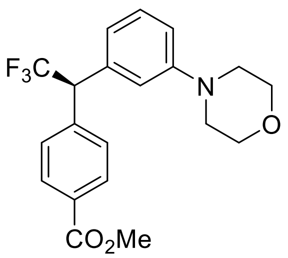

Yellow liquid (49 mg, 43% yield). $^1$H NMR (400 MHz, $CDCl_3$) δ 8.00 (d, $J$ = 8.3 Hz, 2 H), 7.43 (d, $J$ = 8.2 Hz, 2 H), 7.23 (d, $J$ = 8.4 Hz, 1 H), 6.84 (dd, $J$ = 10.9, 2.8 Hz, 3 H), 4.67 (d, $J$ = 9.8 Hz, 1 H), 3.89 (s, 3 H), 3.86 – 3.78 (m, 4 H), 3.18 – 3.04 (m, 4 H); $^{19}$F NMR (376 MHz, $CDCl_3$) δ -65.53 (d, $J$ = 9.8 Hz, 3 F); $^{13}$C NMR (101 MHz, $CDCl_3$) δ 166.61, 151.59, 140.36, 135.57, 129.93, 129.76, 129.63, 129.10, 125.89 (q, $J$ = 281.79), 120.53, 116.51, 115.23, 66.83, 55.65 (q, $J$ = 27.6 Hz), 52.23, 49.08 ppm. MS (EI): 379 (100), 379 ($M^+$); HRMS (EI): Calcd for $C_{20}H_{20}NO_3F_3$: 379.1395; Found: 379.1399. IR (KBr): $\nu_{max}$ = 2957, 2855, 1724, 1602, 1448, 1282, 1261, 1155, 1110, 1021, 745 $cm^{-1}$.

HPLC (IC, 0.46 × 25 cm, 5 μm, hexane/isopropanol = 95/5 (v/v %), flow 0.7 mL/min, UV detection at 214 nm), retention time = 20.33 min (minor) and 22.22 min (major). $[\alpha]_D^{25}$ = 62.823 (c = 0.0500, $CHCl_3$, 94:6 e.r.).

**(R)-Methyl 4-(2,2,2-trifluoro-1-(3-methoxyphenyl)ethyl)benzoate 3d**

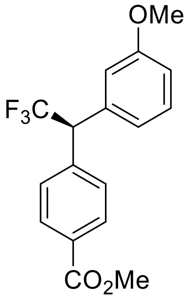

Yellow liquid (68 mg, 77% yield). $^1$H NMR (400 MHz, $CDCl_3$) δ 8.02 (d, $J$ = 8.3 Hz, 2 H), 7.45 (d, $J$ = 8.2 Hz, 2 H), 7.32 – 7.26 (m, 1 H), 6.99 – 6.77 (m, 3 H), 4.71 (q, $J$ = 9.7 Hz, 1 H), 3.91 (s, 3 H), 3.78 (s, 3 H); $^{19}$F NMR (376 MHz, $CDCl_3$) δ -65.64 (d, $J$ = 9.7 Hz, 3 F); $^{13}$C NMR (101 MHz, $CDCl_3$) δ 166.57, 159.81, 140.19, 136.02, 129.96, 129.86, 129.84, 129.10, 125.82 (q, $J$ = 281.79 Hz), 121.41, 115.40, 113.17, 55.39 (q, $J$ = 28.28 Hz), 55.24, 52.19 ppm. MS (EI): 324 (100), 324 ($M^+$); HRMS (EI): Calcd for

$C_{17}H_{15}O_3F_3$: 324.0973; Found: 324.0965. IR (KBr): $\nu_{max}$ = 3002, 2838, 1728, 1603, 1435, 1282, 1259, 1157, 1110, 1050, 1021, 717 $cm^{-1}$.

HPLC (IG, 0.46 × 25 cm, 5 μm, hexane/isopropanol = 99/1(v/v %), flow 0.7 mL/min, UV detection at 214 nm), retention time = 13.38 min (minor) and 14.15 min (major). $[\alpha]_D^{25}$ = 19.1278 (c = 0.1000, $CHCl_3$, 95:5 e.r.).

**(R)-Methyl 4-(1-(3,5-dimethylphenyl)-2,2,2-trifluoroethyl)benzoate 3e**

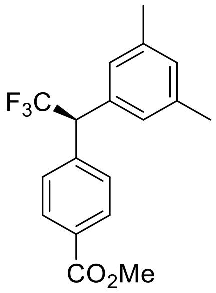


Yellow liquid (60 mg, 62% yield). $^1$H NMR (400 MHz, $CDCl_3$) δ 8.02 (d, *J* = 8.3 Hz, 2 H), 7.45 (d, *J* = 8.2 Hz, 2 H), 6.95 (d, *J* = 5.3 Hz, 3 H), 4.65 (q, *J* = 9.8 Hz, 1 H), 3.91 (s, 3 H), 2.30 (s, 6 H); $^{19}$F NMR (376 MHz, $CDCl_3$) δ -65.61 (d, *J* = 9.8 Hz, 3 F); $^{13}$C NMR (101 MHz, $CDCl_3$) δ 166.63, 140.56, 138.43, 134.47, 129.91, 129.88, 129.70, 129.10, 126.86, 125.94 (q, *J* = 280.6 Hz), 55.40 (q, *J* = 27.8 Hz), 52.16, 21.30 ppm. MS (EI): 253 (100), 322 ($M^+$); HRMS (EI): Calcd for $C_{18}H_{17}O_2F_3$: 322.1181; Found: 322.1186. IR (KBr): $\nu_{max}$ = 2953, 2921, 1727, 1604, 1436, 1281, 1259, 1156, 1110, 1021, 846, 714 $cm^{-1}$.

HPLC (AD-H, 0.46 × 25 cm, 5 μm, hexane/isopropanol = 95/5 (v/v %), flow 0.7 mL/min, UV detection at 214 nm), retention time = 7.48 min (minor) and 9.45 min (major). $[\alpha]_D^{25}$ = 29.9726 (c = 0.0800, $CHCl_3$, 94:6 e.r.).

**(R)-Methyl 4-(2,2,2-trifluoro-1-(naphthalen-2-yl)ethyl)benzoate 3f**

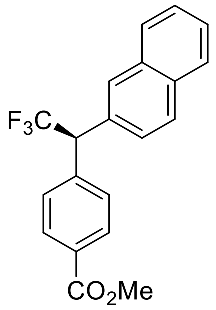

Yellow liquid (69 mg, 67% yield). $^1$H NMR (400 MHz, $CDCl_3$) δ 8.04 (d, $J$ = 8.3 Hz, 2 H), 7.91 – 7.73 (m, 4 H), 7.51 (dd, $J$ = 7.4, 1.6 Hz, 4 H), 7.41 (d, $J$ = 8.5 Hz, 1 H), 4.93 (q, $J$ = 9.7 Hz, 1 H), 3.92 (s, 3 H); $^{19}$F NMR (376 MHz, $CDCl_3$) δ -65.42 (d, $J$ = 9.7 Hz, 3 F); $^{13}$C NMR (101 MHz, $CDCl_3$) δ 166.58, 140.26, 133.21, 132.82, 132.03, 130.01, 129.89, 129.28, 128.70, 128.40, 128.09, 127.65, 126.66, 126.62, 126.53, 126.00 (q, $J$ = 281.79 Hz),55.53 (q, $J$ = 27.8 Hz), 52.22 ppm. MS (EI): 344 (100), 344 ($M^+$); HRMS (EI): Calcd for $C_{20}H_{15}O_2F_3$: 344.1024; Found: 344.1017. IR (KBr): $\nu_{max}$ = 3059, 2952, 1933, 1724, 1614, 1436, 1319, 1282, 1188, 1109, 812, 715 $cm^{-1}$.

SFC (OJ-H, 0.46 × 25 cm, 5 μm, $CO_2$/MeOH = 98/2, flow 2.0 mL/min, column temperature: 40 °C, background press: 2000 psi, UV detection at 214 nm), retention time = 17.17 min (major) and 25.88 min (minor). $[\alpha]_D^{25}$ = 4.9725 (c = 0.1450, $CHCl_3$, 92:8 e.r.).

**(S)-Methyl 4-(2,2,2-trifluoro-1-(p-tolyl)ethyl)benzoate 3g**

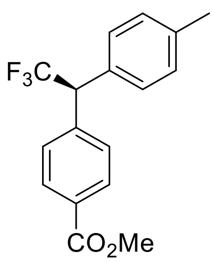


Yellow liquid (49 mg, 54% yield). $^1$H NMR (400 MHz, $CDCl_3$) δ 8.02 (d, $J$ = 8.4 Hz, 2 H), 7.45 (d, $J$ = 8.2 Hz, 2 H), 7.24 (d, $J$ = 8.0 Hz, 2 H), 7.17 (d, $J$ = 8.0 Hz, 2 H), 4.71 (q, $J$ = 9.8 Hz, 1 H), 3.91 (s, 3 H), 2.34 (s, 3 H); $^{19}$F NMR (376 MHz,$CDCl_3$) δ -65.84 (d, $J$ = 9.8 Hz); $^{13}$C NMR (101 MHz, $CDCl_3$) δ 166.61, 140.55, 138.09, 131.68, 129.9, 129.72, 129.57, 129.09, 128.98, 125.96 (q, $J$ = 280.4 Hz), 55.10 (q, $J$ = 27.8 Hz), 52.19, 21.05 ppm. MS (EI): 187 (100), 308 ($M^+$); HRMS (EI): Calcd for $C_{17}H_{15}O_2F_3$: 308.1024; Found: 308.1026. IR (KBr): $\nu_{max}$ = 2953, 1724, 1436, 1280, 1261, 1155, 1106, 1021, 803, 717 $cm^{-1}$.

HPLC (IG, 0.46 × 25 cm, 5 μm, hexane/isopropanol = 90/10 (v/v %), flow 0.7 mL/min, UV detection at 214 nm), retention time = 8.99 min (minor) and 9.46 min (major). $[\alpha]_D^{25}$ = 11.7500 (c = 0.2400, $CHCl_3$, 93:7 e.r.).

**(R)-Methyl 4-(2,2,2-trifluoro-1-(4-fluorophenyl)ethyl)benzoate 3h**

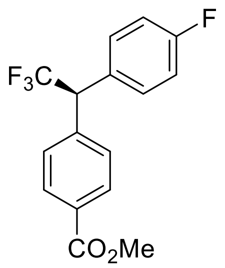

Yellow liquid (39 mg, 42% yield). $^{1}$H NMR (400 MHz, $CDCl_3$) δ 8.03 (d, *J* = 8.4 Hz, 2 H), 7.43 (d, *J* = 8.2 Hz, 2 H), 7.31 (dd, *J* = 8.3, 5.4 Hz, 2 H), 7.05 (t, *J* = 8.6 Hz, 2 H), 4.73 (q, *J* = 9.7 Hz, 1 H), 3.91 (s, 3H); $^{19}$F NMR (376 MHz, $CDCl_3$) δ -66.02 (d, *J* = 9.7 Hz), -113.63; $^{13}$C NMR (101 MHz, $CDCl_3$) δ 166.51, 162.51 (d, *J* = 247.9 Hz), 140.02, 130.85 (d, *J* = 8.2 Hz), 130.48, 130.05, 129.94, 129.0, 125.7(q, *J* = 280.7 Hz), 115.87 (d, *J* = 21.6 Hz), 54.67 (q, *J* = 28.0 Hz), 52.24 ppm. MS (EI): 243 (100), 312 ($M^+$); HRMS (EI): Calcd for $C_{16}H_{12}O_2F_4$: 312.0773; Found: 312.0767. IR (KBr): $\nu_{max}$ = 2959, 1723, 1511, 1436, 1281, 1261, 1157, 1109, 1021 $cm^{-1}$.

HPLC (IF3, 0.46 × 25 cm, 5 μm, hexane/isopropanol = 98/2 (v/v %), flow 0.7 mL/min, UV detection at 214 nm), retention time = 11.49 min (minor) and 12.26 min (major). $[\alpha]_D^{25}$ = 22.2000 (c = 0.4000, $CHCl_3$, 96:4 e.r.).

**(S)-4-(2,2,2-Trifluoro-1-phenylethyl)benzonitrile 3j**

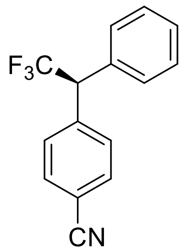

Yellow liquid (60 mg, 77% yield). $^{1}$H NMR (400 MHz, $CDCl_3$) δ 7.65 (d, *J* = 8.3 Hz, 2 H), 7.49 (d, *J* = 8.2 Hz, 2 H), 7.42 – 7.29 (m, 5 H), 4.75 (q, *J* = 9.6 Hz, 1 H); $^{19}$F NMR (376 MHz, $CDCl_3$) δ -65.70 (d, *J* = 9.6 Hz, 3 F); $^{13}$C NMR (101 MHz, $CDCl_3$) δ 140.55, 134.04, 132.50, 129.91, 129.90, 129.05, 128.52, 125.65 (q, *J* = 281.79 Hz), 118.31, 112.12, 55.43 (q, *J* = 28.0 Hz) ppm. MS (EI): 192 (100), 261 ($M^+$); HRMS (EI): Calcd for $C_{15}H_{10}NF_3$: 261.0765; Found: 261.0770. IR (KBr): $\nu_{max}$ = 3065, 3035, 2230, 1611, 1504, 1359, 1261, 1189, 1158, 1107, 1033, 817, 702, 644, 631 $cm^{-1}$. HPLC (OJ-H, 0.46 × 25 cm, 5 μm, hexane/isopropanol = 98/2 (v/v %), flow 0.7 mL/min, UV detection at

214 nm), retention time = 33.32 min (minor) and 50.62 min (major). $[\alpha]_D^{25}$ = 11.1247 (c = 0.3000, $CHCl_3$, 94:6 e.r.).

**(S)-1-Nitro-4-(2,2,2-trifluoro-1-phenylethyl)benzene 3k**

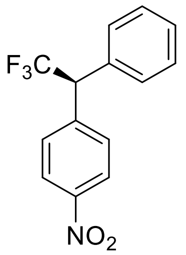

Yellow liquid (45 mg, 53% yield). $^1$H NMR (400 MHz, $CDCl_3$) δ 8.22 (d, *J* = 8.8 Hz, 2 H), 7.56 (d, *J* = 8.6 Hz, 2 H), 7.43 – 7.30 (m, 5 H), 4.80 (q, *J* = 9.6 Hz, 1 H); $^{19}$F NMR (376 MHz, $CDCl_3$) δ -65.71 (d, *J* = 9.6 Hz, 3 F); $^{13}$C NMR (101 MHz, $CDCl_3$) δ 147.57, 142.44, 133.92, 130.10, 129.10, 129.05, 128.60, 125.58 (q, *J* = 280.6 Hz), 123.91, 55.24 (q, *J* = 28.1 Hz) ppm. MS (EI): 212 (100), 281 ($M^+$); HRMS (EI): Calcd for $C_{14}H_{10}O_2F_3$: 281.0664; Found: 281.0659. IR (KBr): $\nu_{max}$ = 3068, 2929, 2859, 1731, 1598, 1522, 1349, 1260, 1161, 1108, 848, 715 cm$^{-1}$.

HPLC (IG, 0.46 × 25 cm, 5 μm, hexane/isopropanol = 99/1 (v/v %), flow 0.7 mL/min, UV detection at 214 nm), retention time = 13.89 min (minor) and 14.80 min (major). $[\alpha]_D^{25}$ = 35.1192 (c = 0.0700, $CHCl_3$, 96:4 e.r.).

**(S)-1-(2,2,2-Trifluoro-1-phenylethyl)-4-(trifluoromethoxy)benzene 3l**

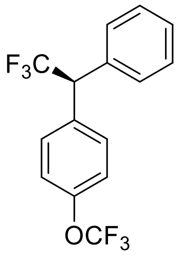

Yellow liquid (65 mg, 68% yield). $^1$H NMR (400 MHz, $CDCl_3$) δ 7.43 – 7.30 (m, 6 H), 7.20 (d, *J* = 8.3 Hz, 2 H), 4.70 (q, *J* = 9.8 Hz, 1 H); $^{19}$F NMR (376 MHz, $CDCl_3$) δ -57.90 (s, 3 F), -66.04 (d, *J* = 9.8 Hz, 3 F); $^{13}$C NMR (101 MHz, $CDCl_3$) δ 148.85, 134.83, 134.04, 130.59, 129.00, 128.89, 128.19, 125.93 (q, *J* = 281.79 HZ), 121.07, 120.41 (q, *J* = 257.6 Hz), 54.87 (q, *J* = 27.8 Hz) ppm. MS (EI): 251 (100), 320 ($M^+$); HRMS (EI):

Calcd for $C_{14}H_{10}BrF_3$: 320.0637; Found: 320.0641. IR (KBr): $\nu_{max}$ = 3036, 2927, 1511, 1359, 1261, 1216, 1161, 1106, 1034, 922, 803 $cm^{-1}$.

HPLC (OJ-H, 0.46 × 25 cm, 5 μm, hexane/isopropanol = 98/2 (v/v %), flow 0.7 mL/min, UV detection at 214 nm), retention time = 7.61 min (minor) and 8.93 min (major). $[\alpha]_D^{25}$ = 2.6503 (c = 0.0600, $CHCl_3$, 95:5 e.r.).

**(S)-1-(2,2,2-Trifluoro-1-phenylethyl)-3-(trifluoromethyl)benzene 3m**

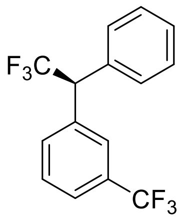


Yellow liquid (68 mg, 75% yield). $^1$H NMR (400 MHz, $CDCl_3$) δ 7.64 (s, 1 H), 7.62 – 7.55 (m, 2 H), 7.49 (t, $J$ = 7.8 Hz, 1 H), 7.42 – 7.32 (m, 5 H), 4.76 (q, $J$ = 9.7 Hz, 1 H); $^{19}$F NMR (376 MHz, $CDCl_3$) δ -62.72 (s, 3 F), -65.92 (d, $J$ = 9.7 Hz, 3 F); $^{13}$C NMR (101 MHz, $CDCl_3$) δ 136.40, 134.46, 132.41, 131.20 (q, $J$ = 32.4 Hz), 129.29, 129.04, 128.97, 128.34, 125.91, 125.84 (q, $J$ = 280.6 Hz), 124.92 (q, $J$ = 3.7 Hz), 123.85 (q, $J$ = 272.5 Hz), 55.33 (q, $J$ = 27.9 Hz) ppm. MS (EI): 235 (100), 304 ($M^+$); HRMS (EI): Calcd for $C_{15}H_{10}F_6$: 304.0687; Found: 304.0684. IR (KBr): $\nu_{max}$ = 3035, 2916, 1684, 1600, 1452, 1331, 1162, 1129, 1077, 799, 699 $cm^{-1}$.

SFC (OJ-H, 0.46 × 25 cm, 5 μm, $CO_2$/MeOH = 99.5/0.5, flow 2.0 mL/min, column temperature: 40 °C, background press: 2000 psi, UV detection at 214 nm), retention time = 2.24 min (major) and 2.37 min (minor). $[\alpha]_D^{25}$ = -7.9503 (c = 0.0750, $CHCl_3$, 97:3 e.r.).

**(S)-1-Chloro-4-(2,2,2-trifluoro-1-phenylethyl)benzene 3n**

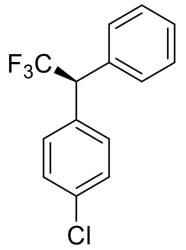


Yellow liquid (58 mg, 72% yield). $^1$H NMR (400 MHz, $CDCl_3$) δ 7.42 – 7.28 (m, 9 H), 4.67 (q, $J$ = 9.8 Hz, 1 H); $^{19}$F NMR (376 MHz, $CDCl_3$) δ -66.00 (d, $J$ = 9.8 Hz, 3 F);

$^{13}C$ NMR (101 MHz, $CDCl_3$) δ 134.92, 134.04, 133.93, 130.47, 129.01, 128.93, 128.85, 128.14, 125.95 (q, $J$ = 281.79 Hz), 54.91 (q, $J$ = 27.8 Hz) ppm. MS (EI): 201 (100), 270 ($M^+$); HRMS (EI): Calcd for $C_{14}H_{10}F_3Cl$: 270.0423; Found: 270.0420. IR (KBr): $\nu_{max}$ = 3066, 3034, 2854, 1604, 1493, 1455, 1358, 1261, 1185, 1156, 1114, 1033, 1016, 730 $cm^{-1}$.

HPLC (OJ-H, 0.46 × 25 cm, 5 μm, hexane/isopropanol = 98/2 (v/v %), flow 0.7 mL/min, UV detection at 214 nm), retention time = 10.74 min (minor) and 14.58 min (major). $[\alpha]_D^{25}$ = 4.0199 (c = 0.1050, $CHCl_3$, 96:4 e.r.).

**(S)-1-Bromo-4-(2,2,2-trifluoro-1-phenylethyl)benzene 3o**

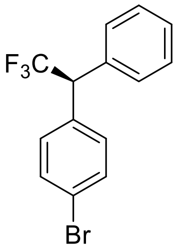


Yellow liquid (63 mg, 67% yield). $^1H$ NMR (400 MHz, $CDCl_3$) δ 7.48 (d, $J$ = 8.5 Hz, 2 H), 7.40 – 7.29 (m, 5 H), 7.24 (d, $J$ = 8.3 Hz, 2 H), 4.65 (q, $J$ = 9.8 Hz, 1 H); $^{19}F$ NMR (376 MHz, $CDCl_3$) δ -65.99 (d, $J$ = 9.8 Hz, 3 F); $^{13}C$ NMR (101 MHz, $CDCl_3$) δ 134.83, 134.43, 131.90, 130.78, 129.00, 128.85, 128.15, 125.88 (q, $J$ = 280.5 Hz), 122.18, 54.97 (q, $J$ = 27.8 Hz) ppm. MS (EI): 165 (100), 314 ($M^+$); HRMS (EI): Calcd for $C_{14}H_{10}BrF_3$: 313.9918; Found: 313.9924. IR (KBr): $\nu_{max}$ = 3065, 3033, 1491, 1357, 1258, 1155, 1033, 1012, 885, 698 $cm^{-1}$.

HPLC (IG, 0.46 × 25 cm, 5 μm, hexane/isopropanol = 95/5 (v/v %), flow 0.7 mL/min, UV detection at 214 nm), retention time = 5.87 min (minor) and 6.13 min (major). $[\alpha]_D^{25}$ = 8.5027 (c = 0.2000, $CHCl_3$, 95:5 e.r.).

**(S)-1-Fluoro-4-(2,2,2-trifluoro-1-phenylethyl)benzene 3p**

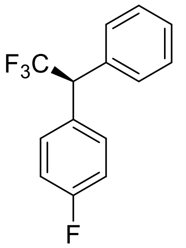


Yellow liquid (40 mg, 52% yield). $^1H$ NMR (400 MHz, $CDCl_3$) δ 7.48 – 7.29 (m, 6 H), 7.04 (t, $J$ = 8.7 Hz, 2 H), 4.67 (q, $J$ = 9.9 Hz, 1 H); $^{19}F$ NMR (376 MHz, $CDCl_3$) δ -

66.15 (d, $J$ = 9.8 Hz, 3 F), -114.28 (dq, $J$ = 8.6, 5.2 Hz, 1 F); $^{13}$C NMR (101 MHz, $CDCl_3$) δ 162.36 (d, $J$ = 247.3 Hz), 135.18, 131.22, 130.81 (d, $J$ = 8.2 Hz), 128.97, 128.81, 128.04, 126.04 (q, $J$ = 280.3 Hz), 115.66 (d, $J$ = 21.5 Hz,), 54.78 (q, $J$ = 27.7 Hz) ppm. MS (EI): 185 (100), 254 ($M^+$); HRMS (EI): Calcd for $C_{14}H_{10}F_4$: 254.0719; Found: 254.0722. IR (KBr): $\nu_{max}$ = 3066, 2926, 1608, 1511, 1497, 1359, 1262, 1186, 1111, 822 $cm^{-1}$.

HPLC (AD-H, 0.46 × 25 cm, 5 µm, hexane = 100%, flow 0.7 mL/min, UV detection at 214 nm), retention time = 9.53 min (minor) and 10.89 min (major). $[\alpha]_D^{25}$ = -3.0174 (c = 0.1500, $CHCl_3$, 96:4 e.r.).

**(S)-4-(2,2,2-Trifluoro-1-phenylethyl)-1,1'-biphenyl 3q**

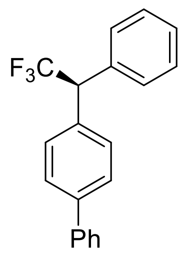


White solid (50 mg, 53% yield). Mp: 87-88 °C. $^1$H NMR (400 MHz, $CDCl_3$) δ 7.58 (d, $J$ = 8.1 Hz, 4 H), 7.49 – 7.29 (m, 10 H), 4.74 (q, $J$ = 9.9 Hz, 1 H); $^{19}$F NMR (376 MHz, $CDCl_3$) δ -65.82 (d, $J$ = 9.9 Hz, 3 F); $^{13}$C NMR (101 MHz, $CDCl_3$) δ 140.85, 140.41, 135.38, 134.39, 129.50, 129.13, 128.82, 128.78, 127.99, 127.51, 127.43, 127.10, 126.21 (q, $J$ = 280.8 Hz), 55.26 (q, $J$ = 27.5 Hz) ppm. MS (EI): 243 (100), 312 ($M^+$); HRMS (EI): Calcd for $C_{20}H_{15}F_3$: 312.1126; Found: 312.1119. IR (KBr): $\nu_{max}$ = 3032, 2922, 1602, 1497, 1410, 1257, 1190, 1122, 1097, 762, 724, 702 $cm^{-1}$.

HPLC (IG, 0.46 × 25 cm, 5 µm, hexane/isopropanol = 99/1 (v/v %), flow 0.7 mL/min, UV detection at 214 nm), retention time = 7.82 min (minor) and 8.78 min (major). $[\alpha]_D^{25}$ = 26.1836 (c = 0.1200, $CHCl_3$, 95:5 e.r.).

**(S)-4-(2,2,2-Trifluoro-1-phenylethyl)phenyl acetate 3r**

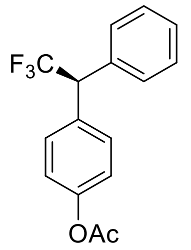

Yellow liquid (49 mg, 55% yield). $^1$H NMR (400 MHz, $CDCl_3$) δ 7.43 – 7.29 (m, 7 H), 7.08 (d, *J* = 8.6 Hz, 2 H), 4.69 (q, *J* = 9.9 Hz, 1 H), 2.29 (s, 3 H); $^{19}$F NMR (376 MHz, $CDCl_3$) δ -65.97 (d, *J* = 9.9 Hz, 3 F); $^{13}$C NMR (101 MHz, $CDCl_3$) δ 169.26, 150.29, 135.11, 132.89, 130.22, 129.08, 128.79, 128.04, 126.10 (q, *J* = 281.79 Hz),121.81, 54.96 (q, *J* = 27.7 Hz), 21.12 ppm. MS (DART POS): 295 (M+H); HRMS (DART POS): Calcd for $C_{16}H_{14}O_2F_3$: 295.0940; Found: 295.0943. IR (KBr): $\nu_{max}$ = 3492, 3065, 2921, 1759, 1608, 1548, 1508, 1369, 1262, 1152, 1102, 1033, 913, 699 cm$^{-1}$.

HPLC (IG, 0.46 × 25 cm, 5 μm, hexane/isopropanol = 99/1 (v/v %), flow 0.7 mL/min, UV detection at 214 nm), retention time = 12.49 min (minor) and 13.57 min (major). $[\alpha]_D^{25}$ = -10.1055 (c = 0.095, $CHCl_3$, 94:6 e.r.).

**(S)-methyl 3-(2,2,2-Trifluoro-1-phenylethyl)benzoate 3s**

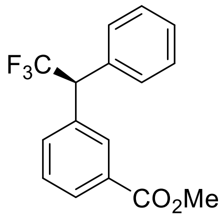


Yellow liquid (49 mg, 56% yield). $^1$H NMR (400 MHz, $CDCl_3$) δ 8.04 (d, *J* = 20.4 Hz, 1 H), 8.02 (s, 1 H), 7.58 (d, *J* = 7.8 Hz, 1 H), 7.44 (dd, *J* = 9.7, 5.8 Hz, 1 H), 7.39 – 7.30 (m, 5 H), 4.74 (q, *J* = 9.8 Hz, 1 H), 3.91 (s, 3 H); $^{19}$F NMR (376 MHz, $CDCl_3$) δ -65.87 (d, *J* = 9.8 Hz, 3 F); $^{13}$C NMR (101 MHz, $CDCl_3$) δ 166.61, 135.86, 134.80, 133.40, 130.74, 130.34, 129.18, 129.07, 128.89, 128.87, 128.16, 125.96 (q, *J* = 280.5 Hz), 55.37 (q, *J* = 27.7 Hz), 52.27 ppm. MS (EI): 225 (100), 294 ($M^+$); HRMS (EI): Calcd for $C_{16}H_{13}O_2F_3$: 294.0868; Found: 294.0872. IR (KBr): $\nu_{max}$ = 3066, 3034, 1724, 1604, 1498, 1448, 1358, 1291, 1155, 1108, 717, 678 cm$^{-1}$.

HPLC (IG, 0.46 × 25 cm, 5 μm, hexane/isopropanol = 99/1 (v/v %), flow 0.7 mL/min, UV detection at 214 nm), retention time = 13.38 min (minor) and 14.15 min (major). $[\alpha]_D^{25}$ = 19.1278 (c = 0.1000, $CHCl_3$, 96:4 e.r.).

**(S)-1-Nitro-3-(2,2,2-trifluoro-1-phenylethyl)benzene 3t**

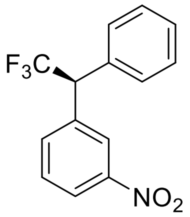


Yellow liquid (41 mg, 49% yield). $^1H$ NMR (400 MHz, $CDCl_3$) δ 8.26 (s, 1 H), 8.22 – 8.15 (m, 1 H), 7.73 (d, $J$ = 7.5 Hz, 1 H), 7.56 (t, $J$ = 8.0 Hz, 1 H), 7.44 – 7.30 (m, 5 H), 4.81 (q, $J$ = 9.6 Hz, 1 H); $^{19}F$ NMR (376 MHz, $CDCl_3$) δ -65.91 (d, $J$ = 9.6 Hz, 3 F); $^{13}C$ NMR (101 MHz, $CDCl_3$) δ 148.44, 137.42, 135.10, 133.93, 129.80, 129.14, 129.02, 128.60, 124.19, 123.11, 123.11, 55.12 (q, $J$ = 28.1 Hz) ppm. MS (EI): 165 (100), 281 ($M^+$); HRMS (EI): Calcd for $C_{14}H_{10}NO_2F_3$: 281.0664; Found: 281.0658. IR (KBr): $\nu_{max}$ = 3093, 2932, 1603, 1498, 1533, 1355, 1261, 1161, 1112, 714 $cm^{-1}$.

HPLC (IG, 0.46 × 25 cm, 5 μm, hexane/isopropanol = 95/5 (v/v %), flow 0.7 mL/min, UV detection at 214 nm), retention time = 13.14 min (minor) and 17.64 min (major). $[\alpha]_D^{25}$ = 17.1305 (c = 0.1300, $CHCl_3$, 96:4 e.r.).

**(S)-1-Chloro-3-(2,2,2-trifluoro-1-phenylethyl)benzene 3u**

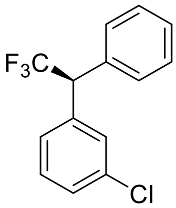


Yellow liquid (69 mg, 86% yield). $^1H$ NMR (400 MHz, $CDCl_3$) δ 7.39 – 7.24 (m, 9 H), 4.63 (q, $J$ = 9.8 Hz, 1 H); $^{19}F$ NMR (376 MHz, $CDCl_3$) δ -65.86 (d, $J$ = 9.8 Hz, 3 F); $^{13}C$ NMR (101 MHz, $CDCl_3$) δ 137.29, 134.65, 134.61, 129.96, 129.28, 129.28, 129.05, 128.88, 128.22, 127.28, 125.86 (q, $J$ = 281.79 Hz), 55.19 (q, $J$ = 27.8 Hz) ppm. MS (EI): 165 (100), 270 ($M^+$); HRMS (EI): Calcd for $C_{14}H_{10}F_3Cl$: 270.0423; Found: 270.0422. IR (KBr): $\nu_{max}$ = 2959, 2927, 1717, 1596, 1573, 1477, 1260, 1189, 1156, 722 $cm^{-1}$.

HPLC (OJ-H, 0.46 × 25 cm, 5 μm, hexane/isopropanol = 98/2 (v/v %), flow 0.7 mL/min, UV detection at 214 nm), retention time = 10.74 min (minor) and 14.58 min (major). $[\alpha]_D^{25}$ = 4.0199 (c = 0.1050, $CHCl_3$, 95:5 e.r.).

**(S)-1,3-Dibromo-5-(2,2,2-trifluoro-1-phenylethyl)benzene 3v**

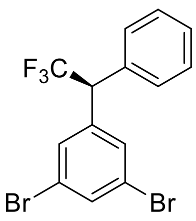


Yellow liquid (68 mg, 58% yield). $^1$H NMR (400 MHz, $CDCl_3$) δ 7.63 (s, 1 H), 7.45 (s, 2 H), 7.42 – 7.29 (m, 5 H), 4.60 (q, *J* = 9.5 Hz, 1 H); $^{19}$F NMR (376 MHz, $CDCl_3$) δ -65.80 (d, *J* = 9.6 Hz, 3 F); $^{13}$C NMR (101 MHz, $CDCl_3$) δ 139.03, 133.89, 133.83, 130.92, 129.06, 128.99, 128.53, 125.55 (q, *J* = 280.7 Hz), 123.23, 54.83 (q, *J* = 28.2 Hz) ppm. MS (EI): 165 (100), 392 ($M^+$); HRMS (EI): Calcd for $C_{14}H_9F_3Br_2$: 391.9023; Found: 391.9027. IR (KBr): $\nu_{max}$ = 3068, 3033, 1584, 1557, 1427, 1356, 1257, 1162, 1113, 853, 759, 722 cm$^{-1}$.

HPLC (OJ-H, 0.46 × 25 cm, 5 μm, hexane/isopropanol = 98/2 (v/v %), flow 0.7 mL/min, UV detection at 214 nm), retention time = 8.72 min (minor) and 10.10 min (major). $[\alpha]_D^{25}$ = 27.8440 (c = 0.1050, $CHCl_3$, 96:4 e.r.).

**(S)-3-Fluoro-5-(2,2,2-trifluoro-1-phenylethyl)benzonitrile 3w**

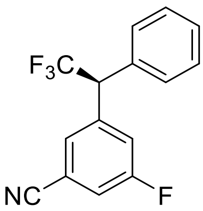


Yellow liquid (59 mg, 71% yield). $^1$H NMR (400 MHz, $CDCl_3$) δ 7.49 – 7.27 (m, 7 H), 4.72 (q, *J* = 9.4 Hz, 1 H); $^{19}$F NMR (376 MHz, $CDCl_3$) δ -65.86 (d, *J* = 9.4 Hz, 3 F), -108.47 (dd, *J* = 8.9, 8.0 Hz, 1 F); $^{13}$C NMR (101 MHz, $CDCl_3$) δ 162.21 (d, *J* = 251.5 Hz), 139.55, 133.34, 129.26, 128.93, 128.94, 128.83, 125.36 (q, *J* = 281.79 Hz), 121.31 (d, *J* = 23.1 Hz), 118.77 (d, *J* = 24.6 Hz), 117.09, 114.37 (d, *J* = 9.7 Hz), 54.82 (q, *J* = 28.28 Hz) ppm. MS (DART POS): 280 (M+H); HRMS (DART POS): Calcd for $C_{15}H_{10}NF_4$: 280.0744; Found: 280.0743. IR (KBr): $\nu_{max}$ = 3086, 3037, 2929, 2234, 1614, 15967, 1456, 1358, 1258, 1135, 1112, 997, 866, 702 cm$^{-1}$.

SFC (OJ-H, 0.46 × 25 cm, 5 μm, $CO_2$/MeOH = 98/2, flow 2.0 mL/min, column temperature: 40 °C, background press: 2000 psi, UV detection at 214 nm) retention time

= 3.84 min (major) and 3.99 min (minor). $[\alpha]_D^{25}$ = 25.1938 (c = 0.0650, $CHCl_3$, 94:6 e.r.).

**(S)-1-Fluoro-2-nitro-4-(2,2,2-trifluoro-1-phenylethyl)benzene 3x**

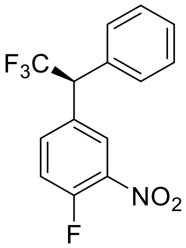


Yellow liquid (54 mg, 60% yield). $^1H$ NMR (400 MHz, $CDCl_3$) δ 8.08 (dd, $J$ = 6.9, 2.3 Hz, 1 H), 7.67 – 7.62 (m, 1 H), 7.43 – 7.27 (m, 6 H), 4.76 (q, $J$ = 9.5 Hz, 1 H); $^{19}F$ NMR (376 MHz, $CDCl_3$) δ -66.11 (d, $J$ = 9.5 Hz, 3 F), -117.99 (ddd, $J$ = 10.7, 6.9, 4.1 Hz, 1 F); $^{13}C$ NMR (101 MHz, $CDCl_3$) δ 155.09 (d, $J$ = 266.6 Hz), 137.31, 136.02 (d, $J$ = 8.7 Hz), 133.61, 132.60, 129.24, 128.89, 128.74, 126.81, 125.28 (q, $J$ = 281.79 Hz). 118.93 (d, $J$ = 21.1 Hz), 54.45 (q, $J$ = 28.3 Hz) ppm. MS (EI): 183 (100), 299 ($M^+$); HRMS (EI): Calcd for $C_{14}H_9NO_2F_4$: 299.0569; Found: 299.0561. IR (KBr): $\nu_{max}$ = 3069, 3036, 1624, 1593, 1541, 1498, 1456, 1354, 1258, 1165, 1110, 1090, 835, 702 $cm^{-1}$.

HPLC (IF3, 0.46 × 25 cm, 3 μm, hexane/isopropanol = 98/2 (v/v %), flow 0.7 mL/min, UV detection at 214 nm), retention time = 8.33 min (minor) and 8.65 min (major). $[\alpha]_D^{25}$ = -12.3927 (c = 0.0800, $CHCl_3$, 93:7 e.r.).

**(S)-3-Fluoro-4-(2,2,2-trifluoro-1-phenylethyl)benzonitrile 3y**

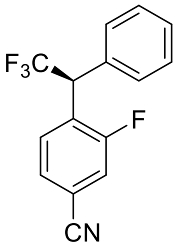


Yellow liquid (63 mg, 75% yield). $^1H$ NMR (400 MHz, $CDCl_3$) δ 7.68 (t, $J$ = 7.6 Hz, 1 H), 7.50 (d, $J$ = 8.2 Hz, 1 H), 7.42 – 7.30 (m, 6 H), 5.12 (q, $J$ = 9.5 Hz, 1 H); $^{19}F$ NMR (376 MHz, $CDCl_3$) δ -65.77 (dd, $J$ = 9.5, 2.7 Hz, 3 F), -113.08 (ddd, $J$ = 9.7, 6.9, 3.1 Hz, 1 F); $^{13}C$ NMR (101 MHz, $CDCl_3$) δ 160.04 (d, $J$ = 252.0 Hz), 132.89, 130.62, 129.07, 128.83, 128.71, 128.40, 128.36, 125.40 (q, $J$ = 280.7 Hz), 119.56 (d, $J$ = 26.2 Hz), 117.07 (d, $J$ = 2.9 Hz), 113.61 (d, $J$ = 9.9 Hz), 47.51 (qd, $J$ = 29.1, 3.9 Hz) ppm. MS (EI): 210 (100), 279 ($M^+$); HRMS (EI): Calcd for $C_{15}H_9NF_4$: 279.0671; Found:

279.0676. IR (KBr): $\nu_{max}$ = 3066, 3036, 2237, 1570, 1504, 1417, 1363, 1250, 1166, 1121, 1094, 952, 732 $cm^{-1}$.

HPLC (IF3, 0.46 × 25 cm, 5 μm, hexane/isopropanol = 98/2 (v/v %), flow 0.7 mL/min, UV detection at 214 nm), retention time = 7.89 min (minor) and 8.30 min (major). $[\alpha]_D^{25}$ = 22.8189 (c = 0.1500, $CHCl_3$, 98:2 e.r.).

**(S)-Methyl 2-fluoro-4-(2,2,2-trifluoro-1-phenylethyl)benzoate 3z**

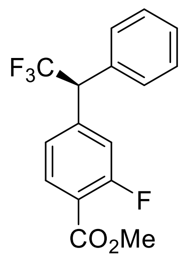


Yellow liquid (61 mg, 65% yield). $^{1}H$ NMR (400 MHz, $CDCl_3$) δ 7.92 (t, *J* = 7.8 Hz, 1 H), 7.42 – 7.28 (m, 5 H), 7.20 (dd, *J* = 17.4, 10.0 Hz, 2 H), 4.71 (q, *J* = 9.6 Hz, 1 H), 3.92 (s, 3 H); $^{19}F$ NMR (376 MHz, $CDCl_3$) δ -65.79 (d, *J* = 9.6 Hz, 3 F), -108.36 (dd, *J* = 11.6, 7.6 Hz, 1 F); $^{13}C$ NMR (101 MHz, $CDCl_3$) δ 164.41 (d, *J* = 3.8 Hz), 161.75 (d, *J* = 260.8 Hz), 142.41, 133.93, 132.47, 129.06, 129.01, 128.51, 125.58 (q, *J* = 280.6 Hz), 124.75, 118.19 (d, *J* = 10.1 Hz), 117.77 (d, *J* = 24.1 Hz), 55.11 (q, *J* = 28.28 Hz), 52.43 ppm. MS (EI): 243 (100), 312 ($M^+$); HRMS (EI): Calcd for $C_{16}H_{12}O_2F_4$: 312.0773; Found: 312.0782. IR (KBr): $\nu_{max}$ = 3035, 2955, 1728, 1625, 1456, 1439, 1357, 1259, 1163, 1111, 817, 711 $cm^{-1}$.

SFC (OJ-H, 0.46 × 25 cm, 5 μm, $CO_2$/MeOH = 98/2, flow 2.0 mL/min, column temperature: 40 °C, background press: 2000 psi, UV detection at 214 nm), retention time = 3.78 min (major) and 4.56 min (minor). $[\alpha]_D^{25}$ = 63.6251 (c = 0.0750, $CHCl_3$, 92:8 e.r.).

**(S)-2-(2,2,2-Trifluoro-1-phenylethyl)naphthalene 3aa**

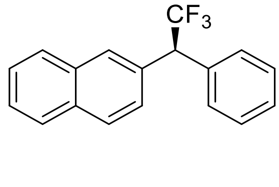

Yellow liquid (44 mg, 51% yield). $^{1}$H NMR (400 MHz, $CDCl_3$) δ 7.84 (dd, $J$ = 18.8, 10.1 Hz, 4 H), 7.56 – 7.28 (m, 8 H), 4.87 (q, $J$ = 9.8 Hz, 1 H); $^{19}$F NMR (376 MHz, $CDCl_3$) δ -65.59 (d, $J$ = 9.9 Hz, 3 F); $^{13}$C NMR (101 MHz, $CDCl_3$) δ 135.37, 133.21, 132.80, 132.71, 129.23, 129.22, 128.74, 128.47, 128.18, 128.08, 127.98, 127.60, 126.73, 126.43, 126.27 (q, $J$ = 280.7 Hz), 55.62 (q, $J$ = 27.5 Hz) ppm. MS (EI): 217 (100), 286 ($M^+$); HRMS (EI): Calcd for $C_{18}H_{13}F_3$: 286.0969; Found: 286.0972. IR (KBr): $\nu_{max}$ = 3059, 2923, 1600, 1507, 1369, 1259, 1207, 1151, 1163, 1099, 1010, 899, 820, 751, 706 $cm^{-1}$.

HPLC (OJ-H, 0.46 × 25 cm, 5 μm, hexane/isopropanol = 95/5 (v/v %), flow 0.7 mL/min, UV detection at 214 nm), retention time = 21.66 min (minor) and 24.82 min (major). $[\alpha]_D^{25}$ = 23.6295 (c = 0.0600, $CHCl_3$, 95:5 e.r.).

**1-(1-Bromo-2,2,2-trifluoroethyl)-4-(tert-butyl)benzene 3ab**

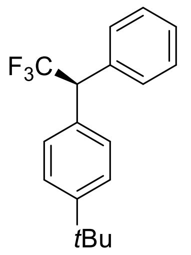


Yellow liquid (30 mg, 35% yield). $^{1}$H NMR (400 MHz, $CDCl_3$) δ 7.46 – 7.28 (m, 9 H), 4.66 (q, $J$ = 10.0 Hz, 1 H), 1.31 (s, 9 H); $^{19}$F NMR (376 MHz, $CDCl_3$) δ -65.91 (d, $J$ = 10.0 Hz); $^{13}$C NMR (101 MHz, $CDCl_3$) δ 150.78, 135.63, 132.32, 129.11, 128.70, 128.68, 127.84, 126.30 (q, $J$ = 280.7 Hz), 125.64, 55.18 (q, $J$ = 27.4 Hz), 34.49, 31.27. MS (EI): 215 (100), 292 ($M^+$); HRMS (EI): Calcd for $C_{18}H_{19}F_3$: 292.1439; Found: 292.1440. IR (KBr): $\nu_{max}$ = 2963, 1518, 1362, 1256, 1152, 1103, 632 $cm^{-1}$.

HPLC (OJ-H, 0.46 × 25 cm, 5 μm, hexane/isopropanol = 98/2 (v/v %), flow 0.7 mL/min, UV detection at 214 nm), retention time = 11.49 min (minor) and 12.26 min (major). $[\alpha]_D^{25}$ = 1.0500 (c = 0.4000, $CHCl_3$, 90:10 e.r.).

**1-(1-Bromo-2,2,2-trifluoroethyl)-4-methoxybenzene 3ac**

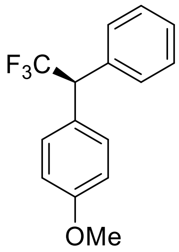

Yellow liquid (20 mg, 25% yield). $^1$H NMR (400 MHz, $CDCl_3$) δ 7.41 – 7.27 (m, 7 H), 6.89 (d, $J$ = 8.8 Hz, 2 H), 4.65 (q, $J$ = 10.0 Hz, 1 H), 3.80 (s, 3 H); $^{19}$F NMR (376 MHz,$CDCl_3$) δ -66.14 (d, $J$ = 10.0 Hz); $^{13}$C NMR (101 MHz, $CDCl_3$) δ 159.21, 135.74, 130.25, 129.00, 128.70, 127.82, 127.52, 126.30 (q, $J$ = 280.7 Hz), 114.10, 55.25, 54.77 (q, $J$ = 27.5 Hz) ppm. MS (EI): 197 (100), 266 ($M^+$); HRMS (EI): Calcd for $C_{15}H_{13}OF_3$: 266.0918; Found: 266.0921. IR (KBr): $\nu_{max}$ = 2935, 2838, 1611, 1512, 1245, 1147, 1099, 1032, 700, 586 $cm^{-1}$.

HPLC (AD-H, 0.46 × 25 cm, 5 μm, hexane/isopropanol = 92/8 (v/v %), flow 0.7 mL/min, UV detection at 214 nm), retention time = 6.46 min (minor) and 7.09 min (major). $[\alpha]_D^{25}$ = -2.740 (c = 0.1900, $CHCl_3$, 90:10 e.r.).

**General procedure for Nickel-Catalyzed Asymmetric Suzuki-Miyaura Coupling of difluoromethylated Secondary Benzyl Bromides**

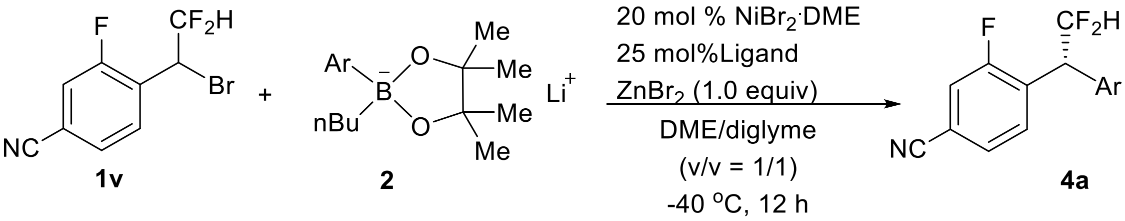


In a glove box, phenyl pinacol boronate ester (5.1 g, 25 mmol) was weighted into a 100 mL Schlenk tube, and 40 mL of dry THF was added. The mixture was taken out from the glove box and then cooled at -20 °C. *n*-BuLi (25 mmol, 10 mL, 2.5 M in Hexanes) was added. The mixture was stirred at -20 °C for 2 h. Then the Schlenk tube was taken into the glove box, the solvents were removed under vacuum to give lithium phenyl pinacol boronate.

In an argon-filled glove box, lithium organoboronate (371 mg, 0.900 mmol, 3.00 equiv.), ligand **L7** (24.8 mg, 0.0750 mmol, 0.250 equiv.), $ZnBr_2$ (67.5 mg, 0.300 mmol, 1.00 equiv.) and $NiBr_2$·DME (18.5 mg, 0.0600 mmol, 0.200 equiv.) were placed into a 25 mL Schlenk tube. To this vial was added 5.0 mL of anhydrous DME/diglyme(v/v = 1:1). The Schlenk tube was taken out from the glove box and cooled at -40°C. 4-(1-Bromo-2,2-difluoroethyl)-3-fluorobenzonitrile (78.9 mg, 0.300 mmol) was added and the mixture was stirred at -40 °C for 12 h. The mixture was quenched by addition of water (5.0 mL and extracted with $Et_2O$ (10.0 mL × 3). The organic layer was combined, dried over anhydrous $Na_2SO_4$ and concentrated under vacuum. The crude product was purified by column chromatography on silica gel using a mixture of pentane/ethyl acetate as the eluent to give (R)-4-(2,2-difluoro-1-phenylethyl)-3-fluorobenzonitrile **4a** as a yellow liquid.

**(R)-4-(2,2-Difluoro-1-phenylethyl)-3-fluorobenzonitrile 4a**

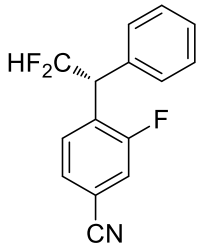

Yellow liquid (50 mg, 63% yield). $^1$H NMR (400 MHz, $CDCl_3$) δ 7.53 (t, *J* = 7.5 Hz, 1 H), 7.46 (dd, *J* = 8.1, 1.3 Hz, 1 H),7.29 -7.40(m, 6 H) 6.37 (td, *J* = 55.5, 4.3 Hz, 1 H), 4.79 (td, *J* = 15.5, 4.3 Hz, 1 H); $^{19}$F NMR (376 MHz, $CDCl_3$) δ -112.80 (ddd, *J* = 12.9, 6.8, 3.4 Hz), -117.55 (dddd, *J* = 282.0, 55.4, 14.6, 4.1 Hz), -118.90 (dddd, *J* = 282.1, 55.5, 16.1, 2.6 Hz); $^{13}$C NMR (101 MHz, $CDCl_3$) δ 160.24 (d, *J* = 250.8 Hz), 134.64, 131.44, 130.38 (d, *J* = 14.5 Hz), 129.09, 128.87, 128.33 (d, *J* = 3.8 Hz), 128.27, 119.53 (d, *J* = 26.3 Hz), 117.28, 115.82 (t, *J* = 245.2 Hz), 113.05 (d, *J* = 9.8 Hz), 48.31 (t, *J* = 22.6 Hz) ppm. MS (EI): 210 (100), 261 ($M^+$); HRMS (EI): Calcd for $C_{15}H_{10}NF_3$: 261.0765; Found: 261.0757. IR (KBr): $\nu_{max}$ = 3064, 2965, 2853, 1603, 1489, 1454, 1381, 1127, 1060, 1031, 699 $cm^{-1}$.

HPLC (IE3, 0.46 × 25 cm, 5 μm, hexane/isopropanol = 99/1 (v/v %), flow 0.7 mL/min, UV detection at 214 nm), retention time = 16.10 min (minor) and 16.60 min (major). $[\alpha]_D^{25}$ = -3.90 (c = 0.2050, $CHCl_3$, 94:6 e.r.).

**(R)-Methyl 4-(2,2-difluoro-1-(4-fluorophenyl)ethyl)-3-fluorobenzoate 4b**

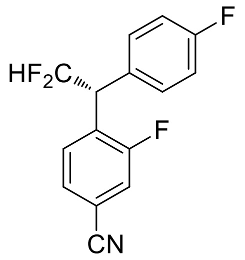


Yellow liquid (44 mg, 52% yield). $^1$H NMR (400 MHz, $CDCl_3$) δ 7.53 – 7.42 (m, 2 H), 7.37 (dd, *J* = 9.5, 1.2 Hz, 1 H), 7.24 (dd, *J* = 8.7, 5.1 Hz, 2 H), 7.03 (t, *J* = 8.6 Hz, 2 H), 6.30 (td, *J* = 55.4, 4.1 Hz, 1 H), 4.73 (td, *J* = 15.5, 4.1 Hz, 1 H); $^{19}$F NMR (376 MHz, $CDCl_3$) δ -112.75 (dd, *J* = 6.2, 3.1 Hz), -113.47, -117.94 (dddd, *J* = 55.5, 18.3, 3.2 Hz), -118.84 (dddd, *J* = 55.5, 18.3, 15.5, 3.2 Hz); $^{13}$C NMR (101 MHz, $CDCl_3$) δ 162.49 (d, *J* = 248.0 Hz), 160.17 (d, *J* = 251.0 Hz), 131.18, 130.65 (d, *J* = 8.3 Hz), 130.24 (d, *J* = 3.5 Hz), 130.07, 128.43 (d, *J* = 3.9 Hz), 119.61 (d, *J* = 26.3 Hz), 117.16 (d, *J* = 2.8 Hz), 116.06 (d, *J* = 21.6 Hz), 115.57 (t, *J* = 244.9 Hz), 113.26 (d, *J* = 9.9 Hz), 47.60 (t, *J* = 22.8 Hz) ppm. MS (EI): 228 (100), 279 ($M^+$); HRMS (EI): Calcd for $C_{15}H_9NF_4$: 279.0671; Found: 279.0674. IR (KBr): $\nu_{max}$ = 3078, 2236, 1606, 1570, 1511, 1417, 1233, 830, 583 $cm^{-1}$.

HPLC (IA, 0.46 × 25 cm, 5 μm, hexane/ isopropanol = 95/5 (v/v %), flow 0.7 mL/min, UV detection at 214 nm), retention time = 12.98 min (minor) and 13.88 min (major). $[\alpha]_D^{25}$ = -13.63 (c = 0.1350, $CHCl_3$, 94:6 e.r.).

**(R)-Methyl 4-(1-(4-chlorophenyl)-2,2-difluoroethyl)-3-fluorobenzoate 4c**

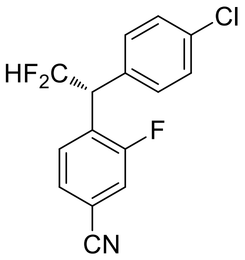


Yellow liquid (54 mg, 61% yield). $^1H$ NMR (400 MHz, $CDCl_3$) δ 7.54 – 7.45 (m, 2 H), 7.40 (dd, *J* = 9.5, 1.1 Hz, 1 H), 7.36 – 7.32 (m, 2 H), 7.22 (d, *J* = 8.5 Hz, 2 H), 6.32 (td, *J* = 55.4, 4.1 Hz, 1 H), 4.75 (td, *J* = 15.4, 4.1 Hz, 1 H); $^{19}F$ NMR (376 MHz, $CDCl_3$) δ -112.66 (ddt, *J* = 9.4, 6.2, 3.1 Hz), -117.93 (dddd, *J* = 73.9, 55.8, 15.4, 3.2 Hz), -118.78 (dddd, *J* = 73.9, 55.5, 15.5, 3.3 Hz); $^{13}C$ NMR (101 MHz, $CDCl_3$) δ 160.17 (d, *J* = 251.2 Hz), 134.41, 132.93, 131.17 (d, *J* = 4.1 Hz), 130.27, 129.85 (d, *J* = 14.4 Hz), 129.27, 128.45 (d, *J* = 3.9 Hz), 119.62 (d, *J* = 26.2 Hz), 117.12 (d, *J* = 2.8 Hz), 115.44 (t, *J* = 245.5 Hz), 113.36 (d, *J* = 9.8 Hz), 47.70 (t, *J* = 22.2 Hz) ppm. MS (EI): 244 (100), 295 ($M^+$); HRMS (EI): Calcd for $C_{15}H_9NF_4Cl$: 295.0376; Found: 295.0374. IR (KBr): $\nu_{max}$ = 3077, 2977, 2236, 1570, 1495, 1416, 1385, 1260, 1129, 1093, 824, 544, 536 $cm^{-1}$.

HPLC (PA2, 0.46 × 25 cm, 5 μm, hexane/ isopropanol = 98/2 (v/v %), flow 0.7 mL/min, UV detection at 214 nm), retention time = 19.60 min (minor) and 20.92 min (major). $[\alpha]_D^{25}$ = 3.24 (c = 0.1050, $CHCl_3$, 91:9 e.r.).

**(S)-Methyl 4-(1-(4-chlorophenyl)-2,2-difluoroethyl)benzoate 4d**

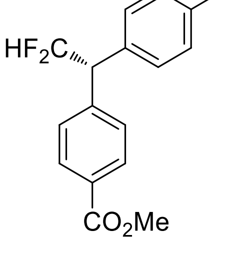

Yellow liquid (61 mg, 66% yield). $^{1}$H NMR (400 MHz,$CDCl_3$) δ 8.02 (d, *J* = 8.4 Hz, 2 H), 7.37 – 7.26 (m, 4 H), 7.21 (d, *J* = 8.4 Hz, 2 H), 6.29 (td, *J* = 55.5, 4.0 Hz, 1 H), 4.45 (td, *J* = 15.8, 3.9 Hz, 1 H), 3.91 (s, 3 H); $^{19}$F NMR (376 MHz, $CDCl_3$) δ -118.27 (dd, *J* = 18.0, 16.1 Hz), -118.42 (dd, *J* = 18.0, 16.1 Hz); $^{13}$C NMR (101 MHz, $CDCl_3$) δ 166.61, 141.54, 134.72, 133.87, 130.44, 130.04, 129.62, 129.04, 129.02, 116.17 (t, *J* = 245.0 Hz), 54.26 (t, *J* = 21.1 Hz), 52.20 ppm. MS (EI): 259 (100), 310 ($M^+$); HRMS (EI): Calcd for $C_{16}H_{13}F_2Cl$: 310.0572; Found: 310.0574. IR (KBr): $\nu_{max}$ = 2962, 2853, 1718, 1612, 1507, 1435, 1411, 1262, 1093, 1058, 1019, 801, 769 $cm^{-1}$.

HPLC (IB, 0.46 × 25 cm, 5 μm, hexane/ isopropanol = 95/5 (v/v %), flow 0.7 mL/min, UV detection at 214 nm), retention time = 10.62 min (minor) and 11.51 min (major). $[\alpha]_D^{25}$ = 4.00 (c = 0.1000, $CHCl_3$, 90:10 e.r.).

**(R)-2-(2,2-Difluoro-1-phenylethyl)naphthalene 4e**

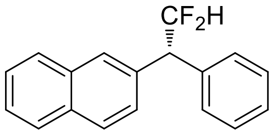


White solid (49 mg, 61% yield). Mp: 67-68 °C. $^{1}$H NMR (400 MHz, $CDCl_3$) δ 7.82 (ddd, *J* = 7.9, 5.1, 2.8 Hz, 4 H), 7.55 – 7.44 (m, 2 H), 7.42 – 7.26 (m, 6 H), 6.44 (td, *J* = 55.8, 4.4 Hz, 1 H), 4.59 (dt, *J* = 15.8, 7.9 Hz, 1 H); $^{19}$F NMR (376 MHz, $CDCl_3$) δ -117.45 (ddd, *J* = 71.4, 39.9, 16.6 Hz), -118.39 (ddd, *J* = 71.4, 39.9, 15.6 Hz); $^{13}$C NMR (101 MHz, $CDCl_3$) δ 137.02, 134.58, 133.32, 132.58, 129.20, 128.73, 128.41, 127.97, 127.77, 127.63, 127.56, 127.09, 126.34, 126.18, 116.95 (t, *J* = 244.4 Hz), 55.08 (t, *J* = 20.8 Hz) ppm. MS (EI): 217 (100), 268 ($M^+$); HRMS (EI): Calcd for $C_{18}H_{14}F_2$: 268.1064; Found: 268.1063. IR (KBr): $\nu_{max}$ = 3052, 2998, 1954, 1632, 1507, 1455, 1118, 1080, 1043, 842, 769 $cm^{-1}$.

HPLC (IC, 0.46 × 25 cm, 5 μm, hexane/ isopropanol = 99/1 (v/v %), flow 0.7 mL/min, UV detection at 214 nm), retention time = 8.63 min (minor) and 9.21 min (major). $[\alpha]_D^{25}$ = -32 (c = 0.1200, $CHCl_3$, 91:9 e.r.).

**(R)-2-(2,2-Difluoro-1-(4-fluorophenyl)ethyl)naphthalene 4f**

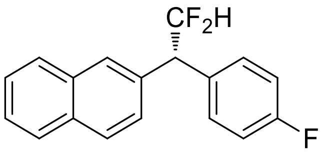


Yellow liquid (57 mg, 67% yield). $^1$H NMR (400 MHz, $CDCl_3$) δ 7.88 – 7.77 (m, 4 H), 7.56 – 7.45 (m, 2 H), 7.38 – 7.28 (m, 3 H), 7.09 – 6.96 (m, 2 H), 6.40 (td, *J* = 55.7, 4.1 Hz, 1 H), 4.57 (td, *J* = 16.1, 4.2 Hz, 1 H); $^{19}$F NMR (376 MHz, $CDCl_3$) δ -114.91 (tt, *J* = 8.5, 5.3 Hz), -117.35 (ddd, *J* = 280.2, 55.6, 14.9 Hz), -118.94 (ddd, *J* = 280.1, 55.8, 16.9 Hz); $^{13}$C NMR (101 MHz, $CDCl_3$) δ 162.19 (d, *J* = 246.6 Hz), 134.35, 133.31, 132.67, 132.60, 130.89 (d, *J* = 8.1 Hz), 128.56, 127.96, 127.65, 127.64, 126.91, 126.47, 126.31, 116.73 (t, *J* = 244.5 Hz), 115.61 (d, *J* = 21.4 Hz), 54.26 (t, *J* = 20.9 Hz) ppm. MS (EI): 235 (100), 268 ($M^+$); HRMS (EI): Calcd for $C_{18}H_{13}F_3$: 286.0969; Found: 286.0977. IR (KBr): $\nu_{max}$ = 3058, 2968, 2926, 1603, 1509, 1466, 1370, 1231, 1311, 1062, 894 $cm^{-1}$.

HPLC (IG, 0.46 × 25 cm, 5 µm, hexane/ isopropanol = 97/3 (v/v %), flow 0.7 mL/min, UV detection at 214 nm), retention time = 7.55 min (minor) and 7.89 min (major). $[\alpha]_D^{25}$ = -49.20 (c = 0.1500, $CHCl_3$, 90:10 e.r.).

**(R)-1-Bromo-4-(2,2-difluoro-1-phenylethyl)benzene 4g**

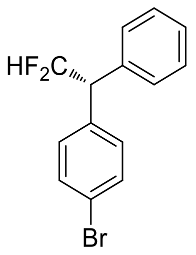


Yellow liquid (51 mg, 57% yield). $^1$H NMR (400 MHz, $CDCl_3$) δ 7.47 (d, *J* = 8.5 Hz, 2 H), 7.39 – 7.27 (m, 5 H), 7.18 (d, *J* = 8.4 Hz, 2 H), 6.28 (td, *J* = 55.7, 4.2 Hz, 1 H), 4.37 (td, *J* = 16.1, 4.2 Hz, 1 H);$^{19}$F NMR (376 MHz, $CDCl_3$) δ -117.59 (ddd, *J* = 280.2, 55.6, 15.0 Hz), -118.98 (ddd, *J* = 280.2, 55.8, 16.8 Hz); $^{13}$C NMR (101 MHz, $CDCl_3$) δ 136.53, 135.99, 131.79, 130.83, 128.92, 128.84, 127.71, 121.65, 116.49 (t, *J* = 244.8 Hz), 54.42 (t, *J* = 20.9 Hz) ppm. MS (EI): 245 (100), 296 ($M^+$); HRMS (EI): Calcd for $C_{14}H_{11}F_2Br$: 296.0012; Found: 296.0022. IR (KBr): $\nu_{max}$ = 3064, 2965, 1603, 1454, 1381, 1127, 1060, 1031, 1011, 972, 699 $cm^{-1}$.

HPLC (IB, 0.46 × 25 cm, 5 μm, hexane/ isopropanol = 98/2 (v/v %), flow 0.7 mL/min, UV detection at 214 nm), retention time = 7.90 min (minor) and 8.46 min (major). $[\alpha]_D^{25}$ = 16.92 (c = 0.0650, $CHCl_3$, 90:10 e.r.).

**(R)-1-Chloro-4-(2,2-difluoro-1-phenylethyl)benzene 4h**

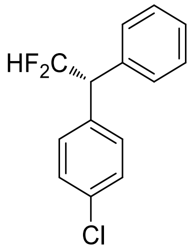

Yellow liquid (61 mg, 81% yield). $^1$H NMR (400 MHz, $CDCl_3$) δ 7.40 – 7.21 (m, 8 H), 6.29 (td, *J* = 55.7, 4.2 Hz, 1 H), 4.4 (td, *J* = 15.5, 4.1 Hz, 1 H); $^{19}$F NMR (376 MHz, $CDCl_3$) δ -117.58 (ddd, *J* = 280.1, 55.6, 15.1 Hz), -118.96 (ddd, *J* = 280.1, 55.7, 16.7 Hz); $^{13}$C NMR (101 MHz, $CDCl_3$) δ 136.63, 135.48, 133.52, 130.49, 128.94, 128.84, 127.71, 116.58 (t, *J* = 244.8 Hz), 54.36 (t, *J* = 20.9 Hz) ppm. MS (EI): 165 (100), 252 ($M^+$); HRMS (EI): Calcd for $C_{14}H_{11}F_2Cl$: 252.0517; Found: 252.0523. IR (KBr): $\nu_{max}$ = 3064, 3032, 2919, 1492, 1128, 1093, 1015, 972, 733, 701 $cm^{-1}$.

HPLC (IB, 0.46 × 25 cm, 5 μm, hexane/isopropanol = 98/2 (v/v %), flow 0.7 mL/min, UV detection at 214 nm), retention time = 7.34 min (minor) and 7.84 min (major). $[\alpha]_D^{25}$ = 4.47 (c = 0.2150, $CHCl_3$, 89:11 e.r.).

**(R)-1-(2,2-Difluoro-1-phenylethyl)-2-fluoro-4-methylbenzene 4i**

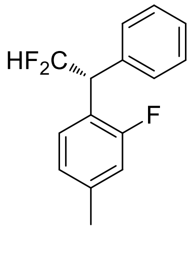

Yellow liquid (52 mg, 69% yield). $^1$H NMR (400 MHz, $CDCl_3$) δ 7.32 -7.36 (m, 4 H), 7.31 – 7.21 (m, 2 H), 6.93 (dd, *J* = 17.9, 9.6 Hz, 2 H), 6.36 (td, *J* = 56.0, 4.8 Hz, 1 H), 4.7 (td, *J* = 16.2, 4.2 Hz, 1 H), 2.34 (s, 3 H); $^{19}$F NMR (376 MHz, $CDCl_3$) δ -117.49 (td, *J* = 8.0, 4.0 Hz), -117.55 (dddd, *J* = 279.2, 55.9, 14.4, 2.6 Hz), -118.58 (dddd, *J* = 279.3, 56.1, 16.3, 3.4 Hz); $^{13}$C NMR (101 MHz, $CDCl_3$) δ 160.56 (d, *J* = 245.9 Hz), 139.83 (d,

*J* = 8.2 Hz), 136.29, 129.76 (d, *J* = 4.4 Hz), 128.96, 128.70, 127.58, 125.09 (d, *J* = 3.1 Hz), 121.39 (d, *J* = 14.7 Hz), 116.54 (t, *J* = 244.2 Hz), 116.37 (d, *J* = 22.3 Hz), 48.33 (t, *J* = 21.9 Hz), 20.98 ppm. MS (EI): 199 (100), 250 ($M^+$); HRMS (EI): Calcd for $C_{15}H_{13}F_3$: 250.0969; Found: 250.0974. IR (KBr): $\nu_{max}$ = 3064, 3033, 2925, 1628, 1508, 1129, 1061, 1003, 806, 698 $cm^{-1}$.

HPLC (IA, 0.46 × 25 cm, 5 μm, hexane/ isopropanol = 100/0 (v/v %), flow 0.7 mL/min, UV detection at 214 nm), retention time = 11.67 min (minor) and 12.44 min (major). $[\alpha]_D^{25}$ = -9.67 (c = 0.2750, $CHCl_3$, 90:10 e.r.).

**General procedure for Nickel-Catalyzed Asymmetric Suzuki-Miyaura Coupling of difluoromethylated Secondary Benzyl Bromides**

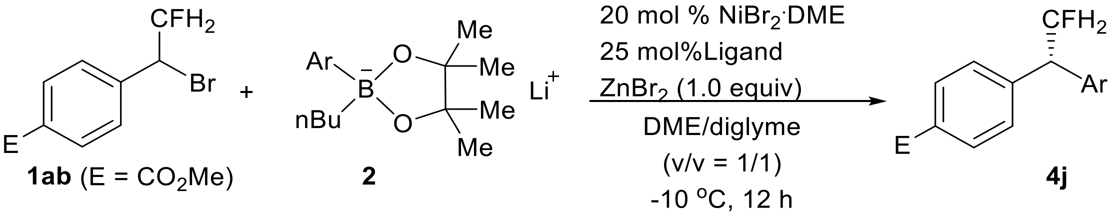


In a glove box, phenyl pinacol boronate ester (5.1 g, 25 mmol) was weighted into a 100 mL Schlenk tube, and 40 mL of dry THF was added. The mixture was taken out from the glove box and cooled at -20 °C. *n*-BuLi (25 mmol, 10 mL, 2.5 M in Hexanes) was added. The mixture was stirred at -20 °C for 2 h. Then the Schlenk tube was taken into the glove box, the solvents were removed under vaccum to give lithium phenyl pinacol boronate.

In an argon-filled glove box, lithium organoboronate (371 mg, 0.900 mmol, 3.00 equiv.), ligand **L8** (20.8 mg, 0.0750 mmol, 0.250 equiv.), $ZnBr_2$ (67.5 mg, 0.300 mmol, 1.00 equiv.) and $NiBr_2$·DME (18.5 mg, 0.0600 mmol, 0.200 equiv.) were placed into a 25 mL Schlenk tube. To this vial was added 5.0 mL of anhydrous DME/diglyme(v/v = 1:1). The Schlenk tube was taken out from the glove box and cooled at -10 °C. Methyl 4-(1-bromo-2-fluoroethyl)benzoate **1z** (80.0 mg, 0.300 mmol) was added and the mixture was stirred at -10 °C for 12 h. The mixture was quenched by addition of water (5.0 mL and extracted with $Et_2O$ (10.0 mL × 3). The organic layer was combined, dried over anhydrous $Na_2SO_4$ and concentrated under vacuum. The crude product was purified by column chromatography on silica gel using a mixture of pentane/ethyl acetate as the eluent to give (R)-methyl 4-(2-fluoro-1-phenylethyl)benzoate **4j** as a yellow liquid.

**(R)-Methyl 4-(2-fluoro-1-phenylethyl)benzoate 4j**

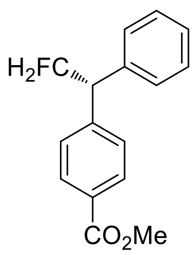

Yellow liquid (66 mg, 85% yield). $^1$H NMR (400 MHz,$CDCl_3$) δ 8.00 (d, $J$ = 8.2 Hz, 2 H), 7.42 – 7.13 (m, 7 H), 4.94 (dd, $J$ = 47.0, 6.7, 2 H), 4.45 (dt, $J$ = 16.9, 6.7 Hz, 1 H), 3.90 (s, 3 H); $^{19}$F NMR (376 MHz, $CDCl_3$) δ -215.42 (td, $J$ = 46.9, 17.1 Hz); $^{13}$C NMR (101 MHz, $CDCl_3$) δ 166.85, 145.60, 139.46, 129.92, 128.90, 128.80, 128.43, 128.34, 127.26, 84.94 (d, $J$ = 175.8 Hz), 52.08, 51.28 (d, $J$ = 19.7 Hz) ppm. MS (EI): 225 (100), 258 ($M^+$); HRMS (EI): Calcd for $C_{16}H_{15}O_2F$: 258.1056; Found: 258.1063. IR (KBr): $\nu_{max}$ = 3061, 2593, 1934, 1719, 1611, 1453, 1280, 1185, 1019, 700 $cm^{-1}$.

HPLC (IG, 0.46 × 25 cm, 5 μm, hexane/ isopropanol = 95/5 (v/v %), flow 0.7 mL/min, UV detection at 214 nm), retention time = 16.50 min (minor) and 17.90 min (major). $[\alpha]_D^{25}$ = -6.33 (c = 0.2400, $CHCl_3$, 83:17 e.r.).

**(R)-1-Fluoro-4-(2-fluoro-1-phenylethyl)benzene 4k**

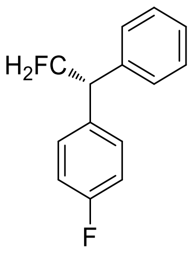


Yellow liquid (37 mg, 57% yield). $^1$H NMR (400 MHz,$CDCl_3$) δ 7.32 (t, $J$ = 7.3 Hz, 2 H), 7.27 – 7.16 (m, 5 H), 7.00 (t, $J$ = 8.6 Hz, 2 H), 4.88 (dd, $J$ = 47.1, 6.8 Hz, 2 H), 4.37 (dt, $J$ = 16.8, 6.8 Hz, 1 H); $^{19}$F NMR (376 MHz,$CDCl_3$) δ -115.98 (ddd, $J$ = 14.6, 9.0, 5.5 Hz, 1 F), -215.01 (td, $J$ = 47.3, 17.3 Hz, 1 F); $^{13}$C NMR (101 MHz, $CDCl_3$) δ 161.80 (d, $J$ = 245.4 Hz), 140.04, 136.12, 129.87 (d, $J$ = 7.9 Hz), 128.73, 128.26, 127.10, 115.46 (d, $J$ = 21.2 Hz), 85.27 (d, $J$ = 175.6 Hz), 50.53 (d, $J$ = 19.6 Hz) ppm. MS (EI): 185 (100), 218 ($M^+$); HRMS (EI): Calcd for $C_{14}H_{12}F_2$: 218.0907; Found: 218.0902. IR (KBr): $\nu_{max}$ = 3030, 2962, 2904, 1508, 1261, 1099, 1017, 800 $cm^{-1}$.

HPLC (OD-H, 0.46 × 25 cm, 5 μm, hexane/ isopropanol = 95/5 (v/v %), flow 0.7 mL/min, UV detection at 214 nm), retention time = 7.62 min (minor) and 7.95 min (major). $[\alpha]_D^{25}$ = 11.67 (c = 0.0600, $CHCl_3$, 79:21 e.r.).

**(R)-1-Chloro-4-(2-fluoro-1-phenylethyl)benzene 4l**

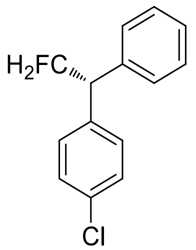

Yellow liquid (42 mg, 60% yield). $^{1}H$ NMR (400 MHz, $CDCl_3$) δ 7.36 – 7.09 (m, 9 H), 4.88 (dd, $J$ = 48.1, 6.8 Hz, 2 H), 4.36 (dt, $J$ = 17.0, 6.7 Hz, 1 H); $^{19}F$ NMR (376 MHz,$CDCl_3$) δ -215.68 (td, $J$ = 48.3, 18.2 Hz); $^{13}C$ NMR (101 MHz, $CDCl_3$) δ 139.79, 138.89, 132.83, 129.73, 128.77, 128.27, 127.18, 85.09 (d, $J$ = 175.8 Hz), 50.66 (d, $J$ = 19.7 Hz) ppm. MS (EI): 201 (100), 234 ($M^+$); HRMS (EI): Calcd for $C_{14}H_{12}ClF$: 234.0612; Found: 234.0620. IR (KBr): $\nu_{max}$ = 3029, 2962, 1601, 1490, 1260, 1092, 1017, 799 $cm^{-1}$.

HPLC (OD-H, 0.46 × 25 cm, 5 μm, hexane/ isopropanol = 95/5 (v/v %), flow 0.7 mL/min, UV detection at 214 nm), retention time = 8.03 min (minor) and 8.58 min (major). $[\alpha]_D^{25}$ = 4.89 (c = 0.0900, $CHCl_3$, 80:20 e.r.).

**Procedure for the synthesis of compound 5**

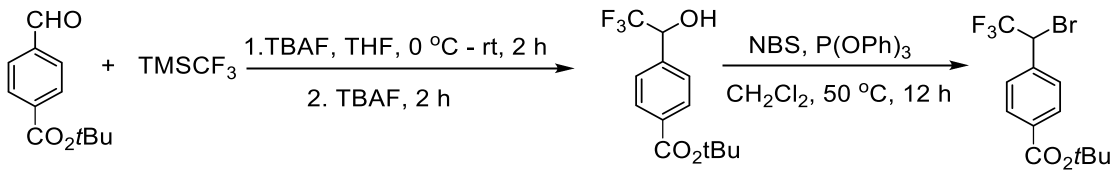


*tert*-Butyl 4-Formylbenzoate (2.0 g, 9.7 mmol) was weighted into a 50 mL Schlenk tube. Trifluoromethyltrimethylsilane ($TMSCF_3$) (2.10 mL, 13.5 mmol) andanhydrous THF (30.0 mL) were added under an argon atmosphere. The mixture was cooled at 0 °C. Then a solution of TBAF (1.0 M in THF, 0.10 mL, 0.10 mmol) was added over 20 min. The resulting mixture was stirred at 0 °C for 30 min, then was allowed to warm to room temperature. The reaction was stirred for 2 h. Then a solution of TBAF (1.0 M in THF, 9.7 mL, 9.7 mmol) was added, and the mixture was stirred for another 2 h. Then, the mixture was extracted with ethyl acetate (3 × 50 mL), and the combined organic layers were dried over anhydrous $Na_2SO_4$. The solvent was removed under vacuum and the crude product was used without further purification.

*tert*-Butyl 4-(1-bromo-2,2,2-trifluoroethyl)benzoate obtained from the previous step was dissolved in dichloromethane (10.0 mL). To the solution was added successively NBS (7.82 g, 14.5 mmol) and triphenyl phosphite (4.50 g, 14.5 mmol). The resulting mixture was stirred at 50 °C for 12 h. The reaction mixture was then cooled to room temperature and the solvent was evaporated in vacuo. The residue was purified by column chromatography on silica gel with a gradient eluent of petroleum ether and ethyl acetate to give *tert*-butyl 4-(1-bromo-2,2,2-trifluoroethyl)benzoate **1ae**.

***tert*-Butyl 4-(1-bromo-2,2,2-trifluoroethyl)benzoate 1ae**

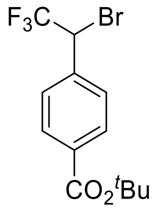


Yellow liquid (1.87 g, 57%). $^1$H NMR (400 MHz, $CDCl_3$) δ 8.01 (d, $J$ = 8.5 Hz, 2 H), 7.56 (d, $J$ = 8.3 Hz, 2 H), 5.15 (q, $J$ = 7.3 Hz, 1 H), 1.59 (s, 9 H); $^{19}$F NMR (376 MHz, $CDCl_3$) δ -70.33 (d, $J$ = 7.3 Hz, 3 F); $^{13}$C NMR (101 MHz, $CDCl_3$) δ 164.75, 136.76, 133.57, 129.91, 129.07, 123.42 (q, $J$ = 278.2 Hz), 81.61, 46.30 (q, $J$ = 34.2 Hz), 28.14 ppm. MS (EI): 175 (100), 282 ($M^+$). HRMS (EI): Calcd for $C_9H_6O_2F_3Br$: 281.9498; Found: 281.9503.IR (KBr): $\nu_{max}$ = 3342, 2921, 2849, 1675, 1560, 1473, 1445, 1371, 1351, 1313, 1112, 1032, 956, 803, 649 cm$^{-1}$.

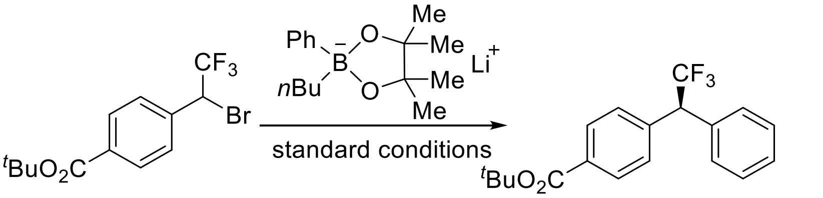


**1ae** **5a** 68% yield, 94.5:5.5 e.r

**(S)-*tert*-Butyl 4-(2,2,2-trifluoro-1-phenylethyl)benzoate 5a**

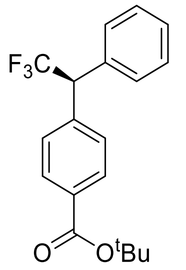

Yellow liquid (178 mg, 53% yield). $^1$H NMR (400 MHz, $CDCl_3$) δ 7.97 (d, $J$ = 8.1 Hz, 2 H), 7.43 (d, $J$ = 8.1 Hz, 2 H), 7.35 – 7.20 (m, 5 H), 4.73 (q, $J$ = 9.8 Hz, 1 H), 1.58 (s, 9 H); $^{19}$F NMR (376 MHz, $CDCl_3$) δ -65.70 (d, $J$ = 9.8 Hz, 3 F); $^{13}$C NMR (101 MHz, $CDCl_3$) δ 165.23, 139.77, 134.80, 131.68, 129.80, 129.09, 128.98, 128.83, 128.16, 125.92 (q, $J$ = 281.1 Hz), 81.22, 55.41 (q, $J$ = 27.6 Hz), 28.16 ppm. MS (DART POS): 337 (M+H); HRMS (DART POS): Calcd for $C_{19}H_{20}O_2F_3$: 337.1410; Found: 337.1410. IR (KBr): $\nu_{max}$ = 2977, 2931, 1712, 1604, 1368, 1294, 1161, 1112, 1035, 1020, 849, 716 cm$^{-1}$.

SFC (OJ-H, 0.46 × 25 cm, 5 μm, $CO_2$/MeOH = 98/2, flow 2.0 mL/min, column temperature: 40 °C, background press: 2000 psi, UV detection at 214 nm), retention time = 2.93 min (major) and 3.38 min (minor). $[\alpha]_D^{25}$ = 37.9622 (c = 0.0600, $CHCl_3$, 94.5:5.5 e.r.).

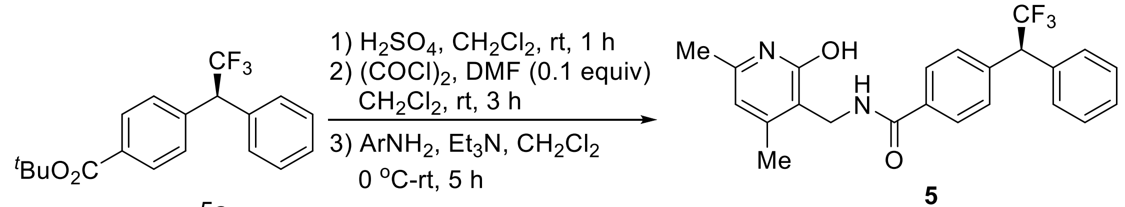


**5**

81% yield, 94:6 e.r.

In a 100 mL flask, compound **5a** (178 mg, 0.520 mmol) was dissolved in $CH_2Cl_2$ (20.0 mL). A solution of $H_2SO_4$ (26 mg, 0.26 mmol) in $CH_2Cl_2$ (5.0 mL) was added. The mixture was stirred at room temperature for 1 h and quenched by addition of water (20 mL). The mixture was extracted with $CH_2Cl_2$ (20 mL × 3) and the organic layer was combined, dried over anhydrous $Na_2SO_4$ and concentrated under vacuum. The crude product was used in the next step without further purification.

To a stirred solution of benzoic acid derivative obtained from previous step (140 mg, 0.470 mmol) and DMF (5.5 μL, 0.10 equiv.) in $CH_2Cl_2$ (10.0 mL) was added oxalyl chloride (140 μL, 3.00 equiv.) at 0 °C. The mixture was stirred at ambient temperature for 3 h and extra oxalyl chloride and the solvent were removed by evaporation under vacuum. The residue was dissolved in $CH_2Cl_2$ (5.0 mL) and a solution of 3-(aminomethyl)-4,6-dimethylpyridin-2-ol (87.0 mg, 1.20 equiv.) and $Et_3N$ (280 μL, 4.00

equiv.) in $CH_2Cl_2$ (5.0 mL) was added dropwise at 0 °C. The mixture was further stirred at room temperature for 5 h before it was quenched by adding water (1.0 mL). The mixture was extracted with $CH_2Cl_2$ (20 mL × 3) and the organic layer was dried over anhydrous $Na_2SO_4$, filtered and concentrated under vacuum. The residue was purified by column chromatography on silica gel with a gradient eluent of petroleum ether and ethyl acetate to give (S)-*N*-((2-hydroxy-4,6-dimethylpyridin-3-yl)methyl)-4-(2,2,2-trifluoro-1-phenylethyl)benzamide **5**.

**(S)-*N*-((2-Hydroxy-4,6-dimethylpyridin-3-yl)methyl)-4-(2,2,2-trifluoro-1-phenylethyl)benzamide 5**

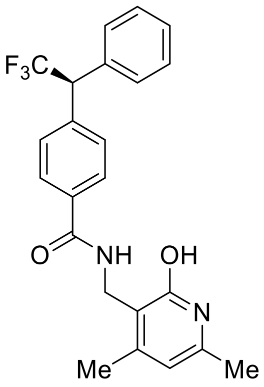


Yellow solid (178 mg, 81% yield). Mp: 130-132 °C. $^1$H NMR (400 MHz, $CDCl_3$) δ 12.84 (s, 1 H), 7.88 (t, $J$ = 5.3 Hz, 1 H), 7.73 (d, $J$ = 8.2 Hz, 2 H), 7.40 – 7.25 (m, 7 H), 6.16 – 5.95 (m, 1 H), 4.68 (q, $J$ = 9.8 Hz, 1 H), 4.53 (d, $J$ = 5.6 Hz, 2 H), 2.35 (s, 3 H), 2.19 (s, 3 H); $^{19}$F NMR (376 MHz, $CDCl_3$) δ -65.77 (d, $J$ = 9.8 Hz, 3 F); $^{13}$C NMR (101 MHz, $CDCl_3$) δ 166.32, 165.54, 150.74, 142.78, 138.56, 134.79, 134.43, 129.18, 129.06, 128.81, 128.14, 127.42, 125.93 (q, $J$ = 280.5 Hz), 121.80, 110.21, 55.26 (q, $J$ = 27.7 Hz), 36.54, 19.64, 18.63 ppm. MS (DART POS): 415 (M+H); HRMS (DART POS): Calcd for $C_{23}H_{22}O_2N_2F_3$: 415.1628; Found: 415.1628. IR (KBr): $\nu_{max}$ = 3288, 2926, 1635, 1540, 1500, 1261, 1154, 1154, 1106, 1021, 849, 701 cm$^{-1}$.

HPLC (IG, 0.46 × 25 cm, 5 μm, hexane/isopropanol = 80/20 (v/v %), flow 0.7 mL/min, UV detection at 214 nm), retention time = 22.45 min (minor) and 28.47 min (major). $[\alpha]_D^{25}$ = 6.9710 (c = 0.2000, $CHCl_3$, 94:6 e.r.).

**Procedure for the synthesis of compound 6**

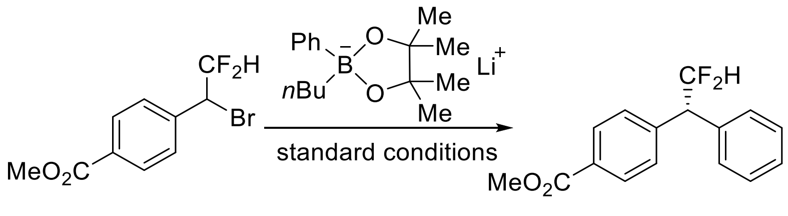


**1w** **6a** 76% yield, 91:9 e.r

In a glove box, phenyl pinacol boronate ester (5.1 g, 25 mmol) was weighted into a 100 mL Schlenk tube, and 40 mL of dry THF was added. The mixture was taken out from the glove box and cooled at -20 °C. *n*-BuLi (25 mmol, 10 mL, 2.5 M in Hexanes) was added. The mixture was stirred at -20 °C for 2 h. Then the Schlenk tube was taken into the glove box, the solvents were removed by oil pump to give lithium phenyl pinacol boronate.

In an argon-filled glove box, lithium organoboronate (371 mg, 0.900 mmol, 3.00 equiv.), ligand **L7** (24.8 mg, 0.0750 mmol, 0.250 equiv.), $ZnBr_2$ (67.5 mg, 0.300 mmol, 1.00 equiv.) and $NiBr_2$·DME (18.5 mg, 0.0600 mmol, 0.200 equiv.) were placed into a 25 mL Schlenk tube. To this vial was added 5.0 mL of anhydrous DME/diglyme(v/v = 1:1). The Schlenk tube was taken out from the glove box and cooled at -40 °C. α-bromo-4-methoxycarbonylbenzyl difluoromethyl **1u** (89.1 mg, 0.300 mmol) was added and the mixture was stirred at -40 °C for 12 h. The mixture was quenched by addition of water (5.0 mL and extracted with $Et_2O$ (10.0 mL × 3). The organic layer was combined, dried over anhydrous $Na_2SO_4$ and concentrated under vacuum. The crude product was purified by column chromatography on silica gel with pentane/ethyl acetate as the eluent to give (R)-methyl 4-(2,2-difluoro-1-phenylethyl)benzoate **6a** as a yellow liquid.

**(R)-Methyl 4-(2,2-difluoro-1-phenylethyl)benzoate 6a**

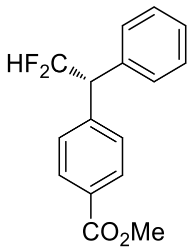


Yellow liquid (210 mg, 76% yield). $^1$H NMR (400 MHz, $CDCl_3$) δ 8.02 (d, *J* = 8.4 Hz, 2 H), 7.55 – 7.18 (m, 7 H), 6.34 (td, *J* = 55.6, 4.3 Hz, 1 H), 4.48 (td, *J* = 15.7, 4.2 Hz, 1 H), 3.91 (s, 3 H); $^{19}$F NMR (376 MHz, $CDCl_3$) δ -117.64 (ddd, *J* = 280.5, 55.6, 15.2

Hz), -118.70 (ddd, $J$ = 280.5, 55.6, 16.2 Hz); $^{13}$C NMR (101 MHz, $CDCl_3$) δ 166.73, 142.12, 136.38, 129.92, 129.39, 129.17, 129.02, 128.87, 127.79, 116.53 (t, $J$ = 244.8 Hz), 54.96 (t, $J$ = 20.9 Hz), 52.15 ppm. MS (EI): 225 (100), 276 ($M^+$); HRMS (EI): Calcd for $C_{16}H_{14}O_2F_2$: 276.0962; Found: 276.0967. IR (KBr): $\nu_{max}$ = 3032, 2953, 1723, 1454, 1284, 1112, 733, 566 cm$^{-1}$.

HPLC (IB, 0.46 × 25 cm, 5 μm, hexane/ isopropanol = 95/5 (v/v %), flow 0.7 mL/min, UV detection at 214 nm), retention time = 9.41 min (minor) and 9.91 min (major). $[\alpha]_D^{25}$ = -6.25 (c = 0.128, $CHCl_3$, 91:9 e.r.).

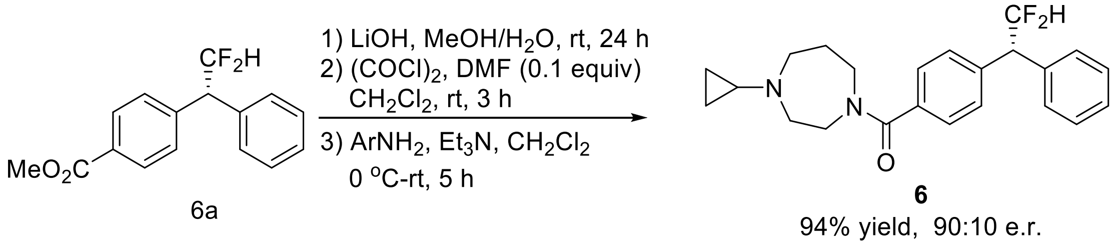


**6**

94% yield, 90:10 e.r.

In a 25 mL flask, compound **6a** (210 mg 0.760 mmol) was dissolved in MeOH (5.0 mL). A solution of LiOH (27.0 mg, 1.14 mmol) in $H_2O$ (5.0 mL) was added. The mixture was stirred at room temperature for 24 h and quenched by an aqueous solution of HCl (2.0 mL, 3.0 N). The mixture was extracted with $CH_2Cl_2$ (20 mL × 3) and the organic layer was combined, dried over anhydrous $Na_2SO_4$ and concentrated under vacuum. The crude product was used in the next step without further purification.

To a stirred solution of benzoic acid derivative obtained from previous step (140 mg, 0.470 mmol) and DMF (8.9 μL, 0.10 equiv.) in $CH_2Cl_2$ (10.0 mL) was added oxalyl chloride (224 μL, 3.00 equiv.) at 0 °C. The mixture was stirred at ambient temperature for 3 h and extra oxalyl chloride and the solvent were removed by evaporation under vacuum. The residue was dissolved in $CH_2Cl_2$ (5.0 mL) and a solution of 1-cyclopropyl-1,4-diazepane (128.0 mg, 1.20 equiv.) and $Et_3N$ (450 μL, 4.00 equiv.) in $CH_2Cl_2$ (5.0 mL) was added dropwise at 0 °C. The mixture was further stirred at room temperature for 5 h before it was quenched by adding water (1.0 mL). The mixture was extracted with $CH_2Cl_2$ (20 mL × 3) and the organic layer was dried over anhydrous $Na_2SO_4$, filtered and concentrated under vacuum. The residue was purified by column chromatography on silica gel with a gradient eluent of petroleum ether and ethyl acetate

to give (R)-(4-cyclopropyl-1,4-diazepan-1-yl)(4- (2,2-difluoro-1-phenylethyl)phenyl)methanone **6**.

**(R)-(4-cyclopropyl-1,4-diazepan-1-yl)(4-(2,2-difluoro-1-phenylethyl)phenyl)methanone 6**

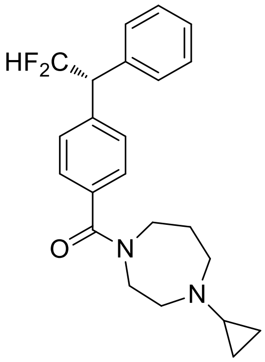


Yellow liquid (274 mg, 94% yield). $^1$H NMR (400 MHz, $CDCl_3$) δ 7.51 – 7.01 (m, 9 H), 6.29 (td, $J$ = 55.7, 4.2 Hz, 1 H), 4.41 (td, $J$ = 15.9, 3.8 Hz, 1 H), 3.73 (d, $J$ = 5.6 Hz, 2 H), 3.59 – 3.28 (m, 2 H), 2.95 (s, 1 H), 2.83 (d, $J$ = 5.3 Hz, 1 H), 2.76 (s, 2 H), 1.97 – 1.86 (m, 2 H), 1.82 – 1.73 (m, 1 H), 0.35-0.49 (m, 4 H); $^{19}$F NMR (376 MHz,$CDCl_3$) δ -117.67 (ddd, $J$ = 71.1, 55.7, 14.9 Hz), -118.66 (dddd, $J$ = 279.7, 55.6, 16.2, 9.7 Hz); $^{13}$C NMR (101 MHz, $CDCl_3$) δ 171.08, 138.23, 136.60, 136.21, 129.20, 129.04, 128.78, 127.68, 127.07, 116.66 (t, $J$ = 244.7 Hz), 56.83, 55.90, 54.83 (t, $J$ = 20.8 Hz), 48.69, 45.56, 38.01, 26.84, 7.29 ppm. MS (EI): 245 (100), 384 ($M^+$); HRMS (EI): Calcd for $C_{23}H_{26}N_2OF_2$: 384.2013; Found: 384.2018. IR (KBr): $\nu_{max}$ = 3030, 2940, 2814, 1625, 1532, 1496, 1185, 1126, 1107, 1079, 1057, 736, 700, 615 cm$^{-1}$. HPLC (AD-H, 0.46 × 25 cm, 5 μm, hexane/ isopropanol = 8/2 (v/v %), flow 0.7 mL/min, UV detection at 214 nm), retention time = 20.70 min (minor) and 23.46 min (major). $[\alpha]_D^{25}$ = 3.23 (c = 0.1550, $CHCl_3$, 90:10 e.r.).

## Supplementary Note 1

## Mechanism studies

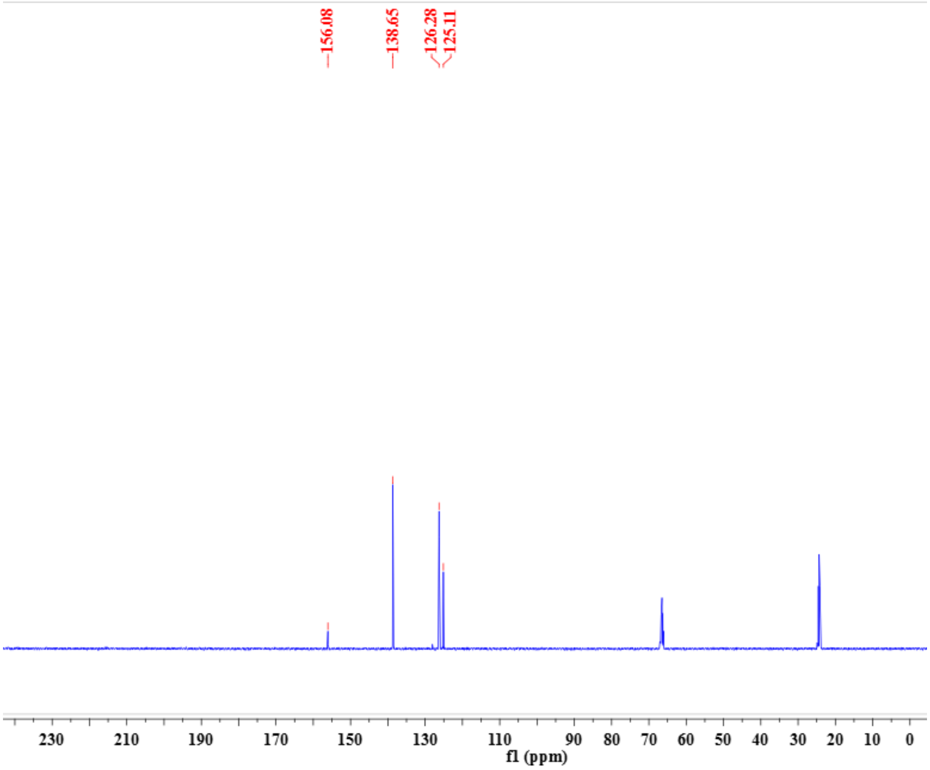


**Supplementary Figure 1**. $^{13}C$ NMR spectra of $Ph_2Zn$ in THF-*d8*.

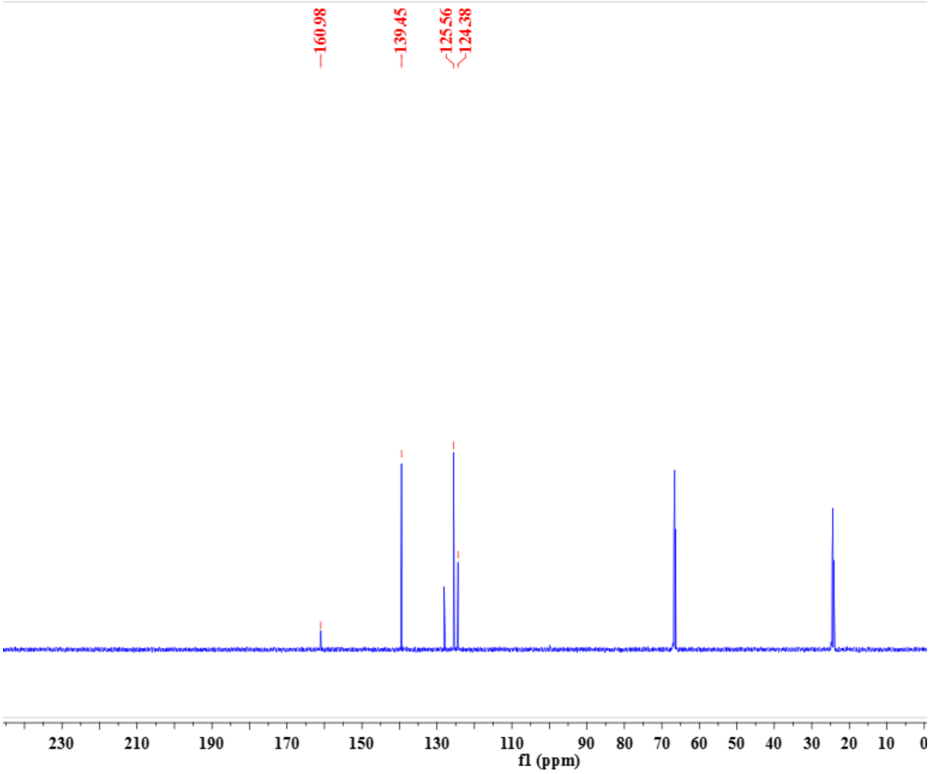


**Supplementary Figure 2**. $^{13}C$ NMR spectra of $Ph_2Zn$ + LiBr in THF-*d8*.

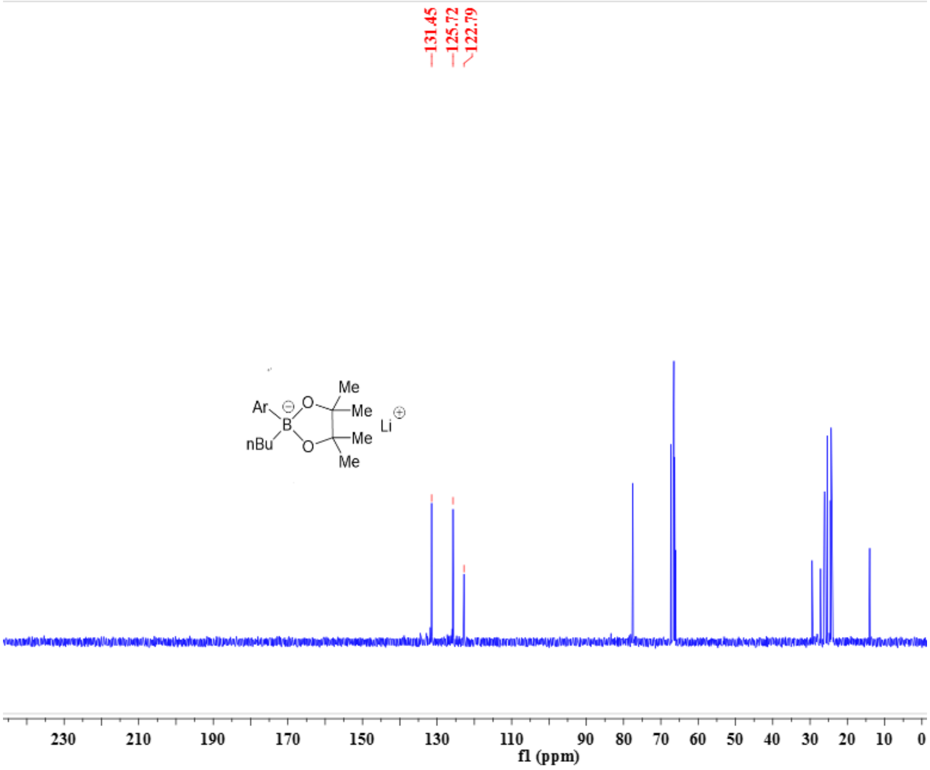


**Supplementary Figure 3**. $^{13}C$ NMR spectra of **2a** in THF-*d8*.

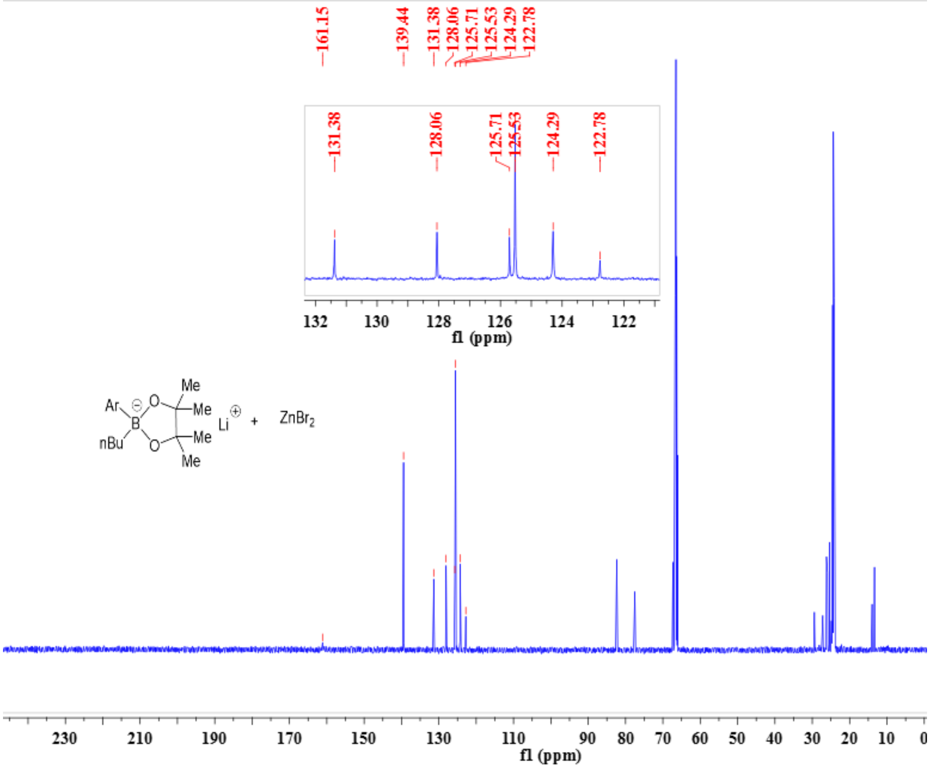


**Supplementary Figure 4**. $^{13}C$ NMR spectra of **2a**+$ZnBr_2$ in THF-*d8*.

In addition to these $^{13}C$ NMR spectra, we also studied the $^{1}H$ NMR spectra of these species. Mixing an equimolar amount of $Ph_2Zn$ with LiBr at room temperature in THF-d8 for 0.5 h generated the desired species (δ 7.82 (d, *J* = 6.2 Hz, 2 H), 7.07 (t, *J* = 7.2 Hz, 2 H), 6.98 (t, *J* = 7.3 Hz, 1 H) in $^{1}H$ NMR spectrum) (**Supplementary Figure 5**). Likewise, the same species was formed after 0.5 h at room temperature for the reaction of 3.0 equivalvents of lithium phenyl borate **2a** with $ZnBr_2$ (δ 7.83 (d, *J* = 6.3 Hz, 2H), 7.06 (t, *J* = 7.3 Hz, 2 H), 6.97 (t, *J* = 7.3 Hz, 1 H) in $^{1}H$ NMR spectrum ) (**Supplementary Figure 6**).

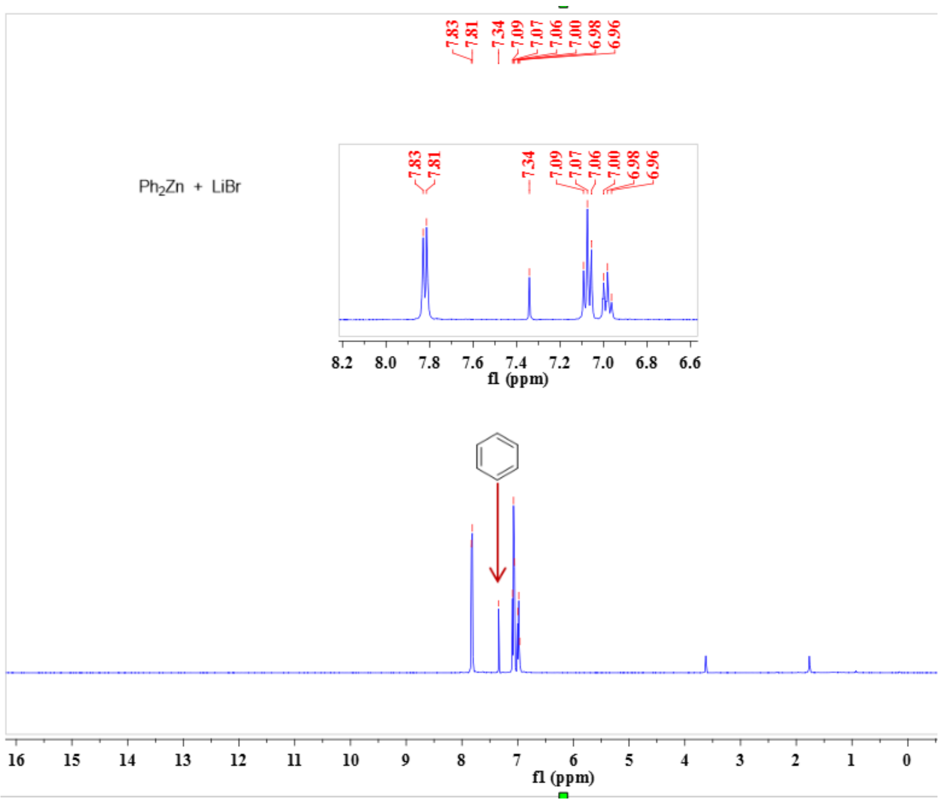


**Supplementary Figure 5**. $^{1}H$ NMR spectra of $Ph_2Zn$ + LiBr in THF-*d8*.

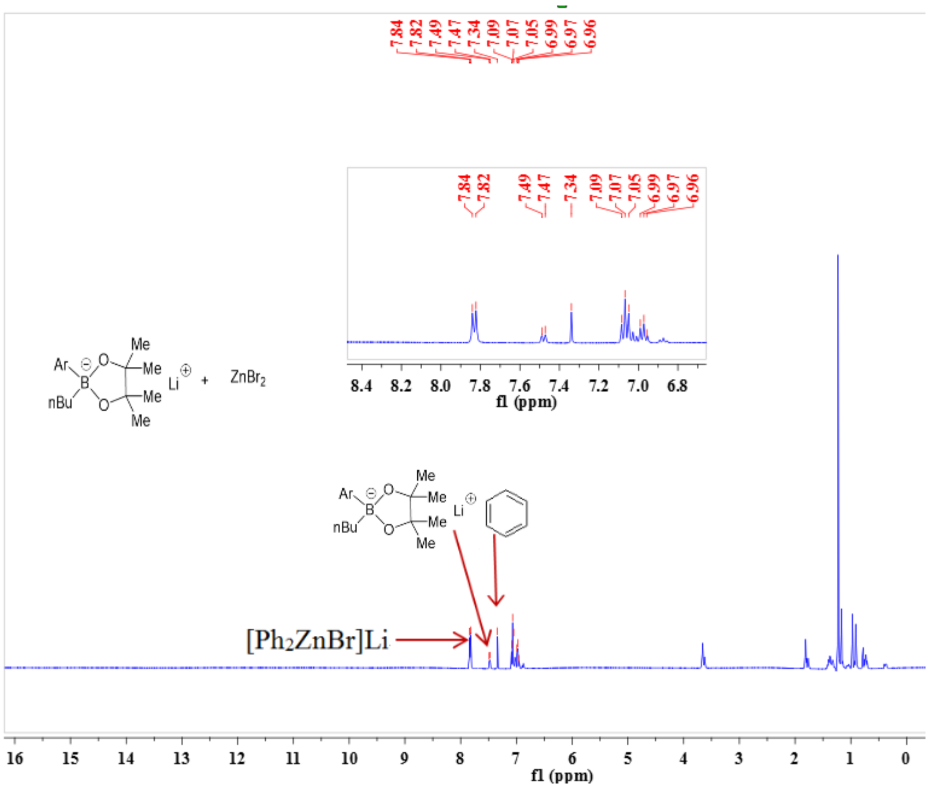


**Supplementary Figure 6**. $^{1}H$ NMR spectra of **2a**+$ZnBr_2$ in THF-*d8*.

Furthermore, we also studied the $^{11}B$ NMR spectrum for the reaction of **2a** and $ZnBr_2$. It was found that reaction of **2a** and $ZnBr_2$ generated BuBpin (δ 34.12 ppm in $^{11}B$ NMR spectrum), and unreacted **3a** with a chemical shift at 7.49 ppm (**Supplementary Figure 7**).

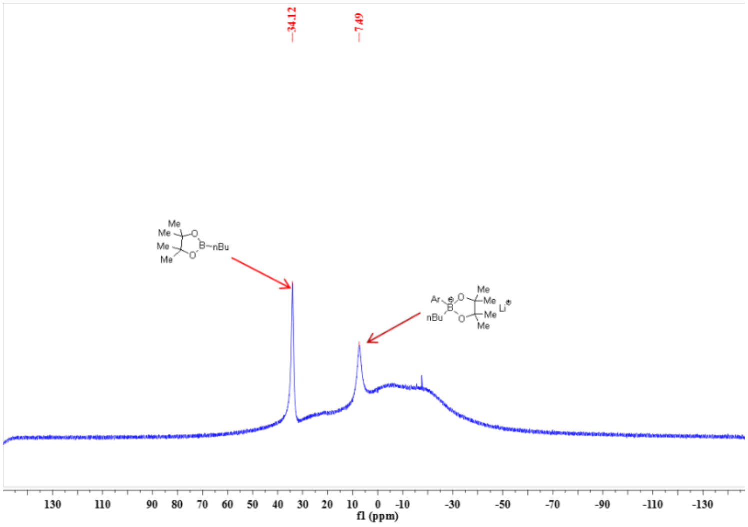


**Supplementary Figure 7.** $^{11}B$ NMR spectra of **2a**+$ZnBr_2$ in THF-*d8*.

**Supplementary Note 2**

**Determination of the absolute configuration of compound 3j**

**ECD experimental**

ECD and UV spectra of **3j** at a concentration of 0.1 mg/mL in acetonitrile, were recorded in a 1 mm pathlength quartz cuvette, using a Chirascan circular dichroism spectrometer (Applied Photophycis Ltd., Leatherhead, UK). The experimental conditions were shown as follows: bandwidth, 1 nm; wavelength range, 190-400 nm; wavelength step size, 1 nm; time-per-point, 0.5 s; temperature, 25 °C. Acetonitrile was measured under the same conditions to obtain baseline.

**ECD computations**

Molecular model of compound **3j** was built and subjected to a conformational analysis using the Monte Carlo protocol at the molecular mechanic force field MMFF94 level with Compute VOA (BioTools Inc., Jupiter, FL). Within a 20 kcal/mol window, 7 conformers were predicted. Geometry optimization of the conformers were then carried out in the framework of density functional theory (DFT) using the B3LYP hybrid density functional and 6-31G(d) basis set with Gaussian 09 (Gaussian Inc., Wallingford, CT). Frequency calculations were also carried out to confirm the geometries obtained were true minima of the potential energy surface by exhibiting no imaginary frequencies. Structures and population percentages based onDFT//B3LYP/6-31G(d) relative energies of the seven conformers in the initial 0.1 kcal/mol range, accounting for 99.9% of the total population, are shown in Fig. S1.

Rotatory strengths in velocity form ($R_{vel}$) and length form ($R_{len}$), oscillator strengths and excitation energies ofthe 50 lowest electronic transitions were calculated for each conformer employing time-dependent density functional theory (TDDFT) at B3LYP/6-31G(d).$R_{vel}$, oscillator strengths and ó=0.28 eV（UV peak half-width at half height）were adopted to simulate ECD andUV curves with GaussView 5. Boltzmann-population-weighted composite ECD and UV spectra were then generated.

The observed and calculated ECD and UV spectra of **3j** over the range of 200-350 nm were shown in Fig. S2 and Fig. S3. The experimental ECD spectrum has a positive

Cotton effect at 205 nm, which can be assigned to the calculated band of compound **3j** at 216 nm. The observed negative Cotton effects at 220 nm and 190 nm can be assigned to the calculated bands of compound **3j** at 240 nm and 207 nm respectively. The calculated bands are blue-shifted by 10-30 nm to the measured ones. Because the observed spectrum is consistentwith the calculated spectrum, compound**3g**is unambiguouslyassigned to have(*S*) configuration.

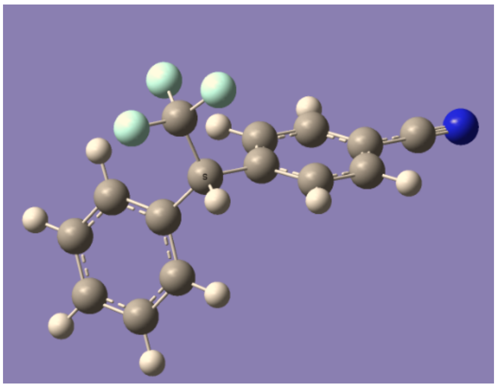

C1 15.3%

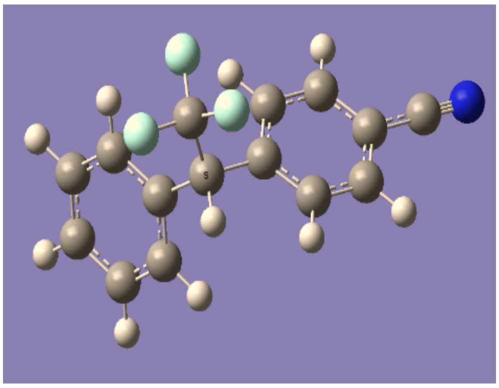

C2 15.3%

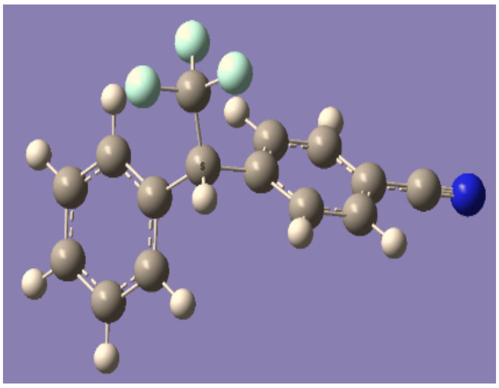

C3 15.3%

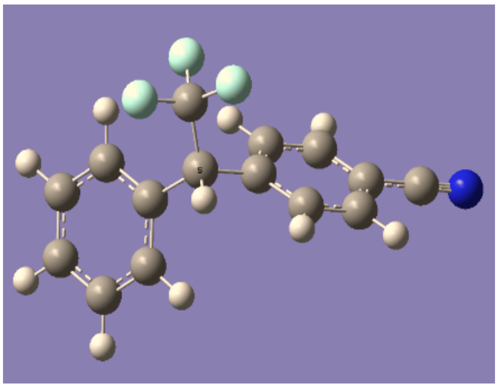

C4 15.3%

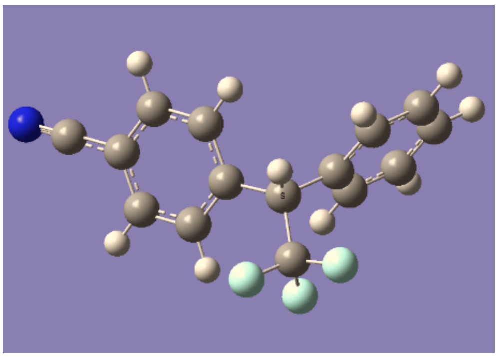

C5 12.9%

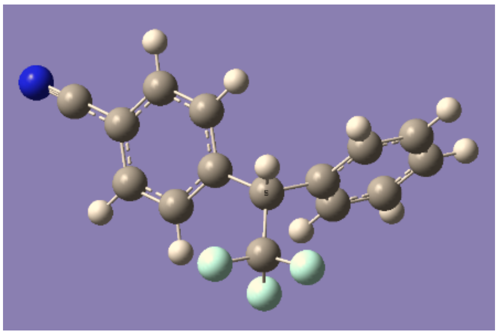

C6 12.9%

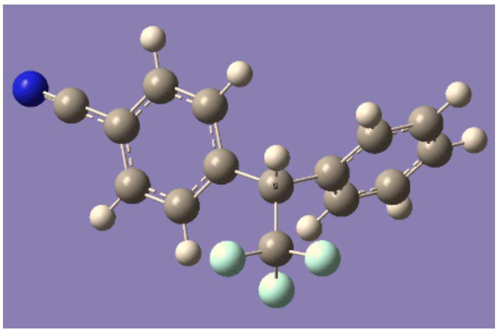


C7 12.9%

**Supplementary Figure 8.** DFT//B3LYP/6-31G(d) minimum energy structures of compound **3j**.

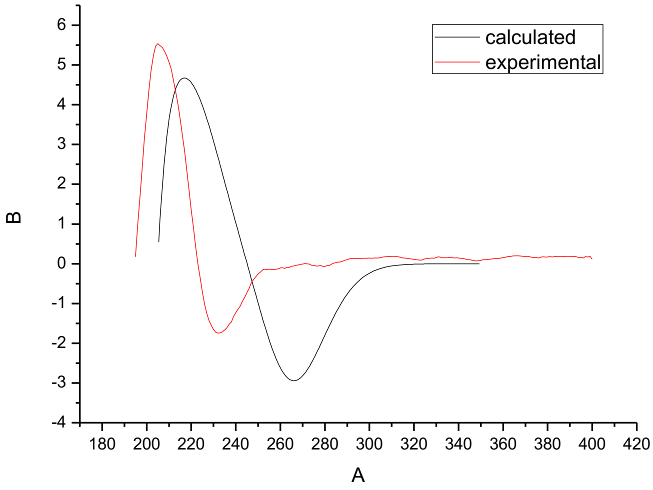


**Supplementary Figure 9.** Comparison of theexperimental(top) and calculated (bottom) ECD spectra of compound **3j**.

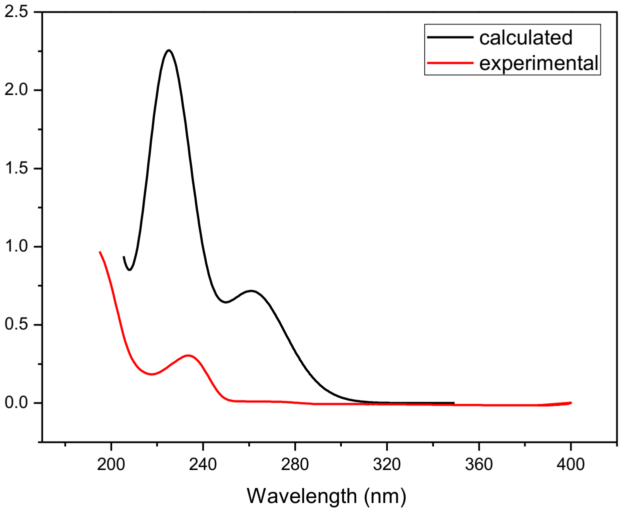


**Supplementary Figure 10.** Comparison of theexperimental (top) and calculated(bottom) UV spectra of compound **3j**.

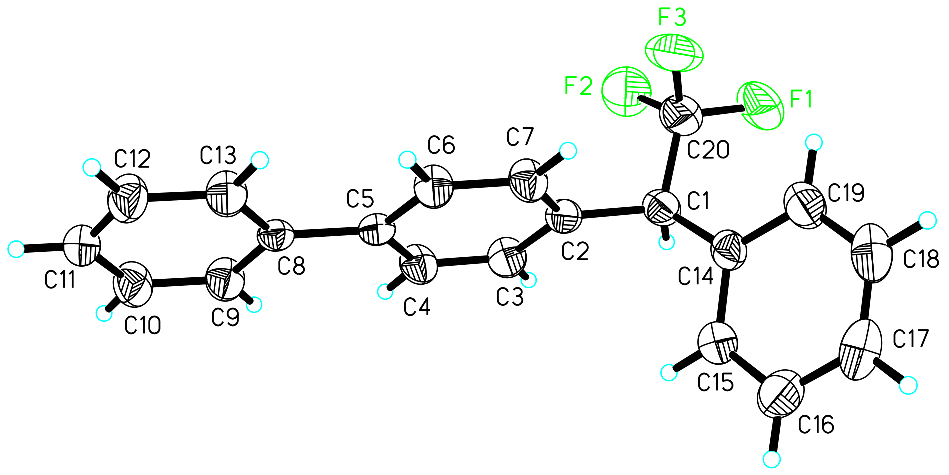


**Supplementary Figure 11.** X-ray structure of compound **3q**.

**Supplementary Table 4**. Crystal data and structure refinement for compound 3n (9-50).

| | |
|---|---|
| Identification code | 9-50 |
| Empirical formula | C20 H15 F3 |
| Formula weight | 312.32 |
| Temperature | 297(2) K |
| Wavelength | 1.54178 Å |
| Crystal system, space group | Monoclinic, P 21 |
| Unit cell dimensions | a = 7.6216(2) A  α = 90 deg. |
| | b = 5.7428(2) A  β = 90.588(2) deg. |
| | c = 17.7537(5) A  γ = 90 deg. |
| Volume | 777.03(4) $Å^3$ |
| Z, Calculated density | 2, 1.335 $Mg/m^3$ |
| Absorption coefficient | 0.850 $mm^{-1}$ |
| F(000) | 324 |
| Crystal size | 0.22 x 0.18 x 0.08 mm |
| Theta range for data collection | 2.49 to 68.21 deg. |
| Limiting indices | -9<=h<=9, -6<=k<=6, -21<=l<=21 |
| Reflections collected / unique | 7597 / 2747 [R(int) = 0.0382] |
| Completeness to theta = 68.21 | 97.0 % |
| Absorption correction | Semi-empirical from equivalents |
| Max. and min. transmission | 0.7531 and 0.6068 |
| Refinement method | Full-matrix least-squares on $F^2$ |
| Data / restraints / parameters | 2747 / 1 / 208 |
| Goodness-of-fit on $F^2$ | 1.032 |
| Final R indices [I>2sigma(I)] | R1 = 0.0381, wR2 = 0.0906 |
| R indices (all data) | R1 = 0.0501, wR2 = 0.0985 |
| Absolute structure parameter | -0.19(19) |
| Largest diff. peak and hole | 0.111 and -0.147 e. $Å^{-3}$ |

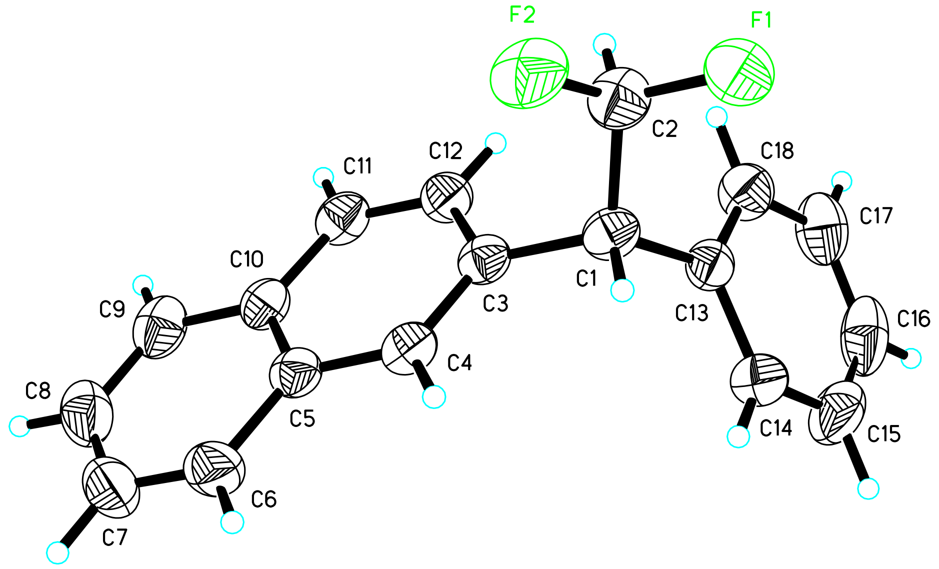


**Supplementary Figure 12**. X-ray structure of compound **4e**.

**Supplementary Table 5.** Crystal data and structure refinement for cu_d8v181061_0m.

| | | |
|---|---|---|
| Identification code | cu_d8v181061_0m | |
| Empirical formula | C18 H14 F2 | |
| Formula weight | 268.29 | |
| Temperature | 293(2) K | |
| Wavelength | 1.54178 Å | |
| Crystal system | Monoclinic | |
| Space group | P 21 | |
| Unit cell dimensions | a = 8.2132(2) Å | α= 90°. |
| | b = 5.86600(10) Å | β= 103.5680(10)°. |
| | c = 14.7733(3) Å | γ = 90°. |
| Volume | 691.89(3) $Å^3$ | |
| Z | 2 | |
| Density (calculated) | 1.288 $Mg/m^3$ | |
| Absorption coefficient | 0.758 $mm^{-1}$ | |
| F(000) | 280 | |
| Crystal size | 0.180 x 0.160 x 0.100 $mm^3$ | |
| Theta range for data collection | 3.077 to 66.991°. | |
| Index ranges | -9<=h<=9, -6<=k<=6, -17<=l<=17 | |
| Reflections collected | 8908 | |
| Independent reflections | 2383 [R(int) = 0.0660] | |
| Completeness to theta = 67.679° | 96.4 % | |
| Absorption correction | Semi-empirical from equivalents | |
| Max. and min. transmission | 0.7456 and 0.4898 | |
| Refinement method | Full-matrix least-squares on $F^2$ | |
| Data / restraints / parameters | 2383 / 1 / 182 | |
| Goodness-of-fit on $F^2$ | 1.045 | |
| Final R indices [I>2sigma(I)] | R1 = 0.0464, wR2 = 0.1246 | |
| R indices (all data) | R1 = 0.0495, wR2 = 0.1286 | |
| Absolute structure parameter | 0.12(13) | |
| Extinction coefficient | 0.042(14) | |
| Largest diff. peak and hole | 0.243 and -0.116 e.$Å^{-3}$ | |

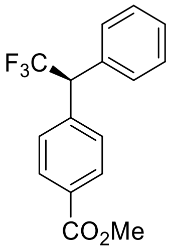


Compound **3a**. HPLC (OJ-H, 0.46 × 25 cm, 5 μm, hexane/isopropanol = 99/1 (v/v %), flow 0.7 mL/min, UV detection at 214 nm), retention time = 15.37 min (minor) and 17.00 min (major). $[\alpha]_D^{25}$ = 22.9650 (c = 0.1800, $CHCl_3$, 96:4 e.r.).

2018-5-26 10:1[illegible]

**1549 HWC-9-88-3+- IF3 991 214 0.7**

| | | | |
|---|---|---|---|
| *Sample Name:* | HWC-9-88-3+- IF3 991 214 0.7 | *Injection Volume:* | 3.0 |
| *Vial Number:* | RA4 | *Channel:* | UV_VIS_2 |
| *Sample Type:* | unknown | *Wavelength:* | 214.0 |
| *Control Program:* | test-dad6 | *Bandwidth:* | 4 |
| *Quantif. Method:* | 20170608 | *Dilution Factor:* | 1.0000 |
| *Recording Time:* | 2018-4-9 10:53 | *Sample Weight:* | 1.0000 |
| *Run Time (min):* | 35.00 | *Sample Amount:* | 1.0000 |

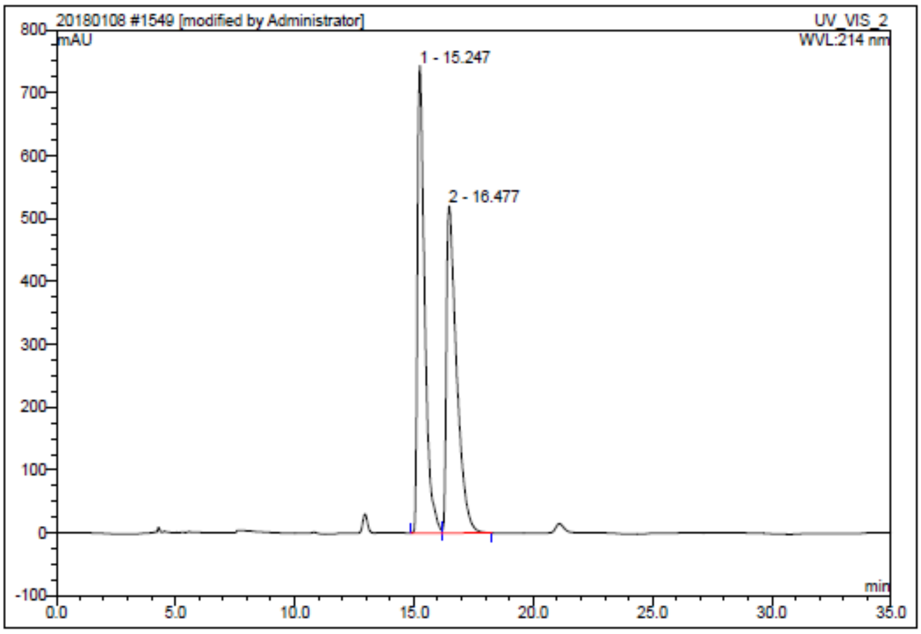


| No. | Ret.Time min | Peak Name | Height mAU | Area mAU*min | Rel.Area % | Amount | Type |
|---|---|---|---|---|---|---|---|
| 1 | 15.25 | n.a. | 743.200 | 263.238 | 50.51 | n.a. | BM |
| 2 | 16.48 | n.a. | 519.756 | 257.958 | 49.49 | n.a. | MB |
| Total: | | | 1262.955 | 521.196 | 100.00 | 0.000 | |

**Supplementary Figure 13**. HPLC chromatogram for compound **3a**, racemic

**1551 HWC-9-85-3 IF3 991 214 0.7**

| | | | |
|---|---|---|---|
| *Sample Name:* | **HWC-9-85-3 IF3 991 214 0.7** | *Injection Volume:* | **3.0** |
| *Vial Number:* | **RC4** | *Channel:* | **UV_VIS_2** |
| *Sample Type:* | **unknown** | *Wavelength:* | **214.0** |
| *Control Program:* | **test-dad6** | *Bandwidth:* | **4** |
| *Quantif. Method:* | **20170608** | *Dilution Factor:* | **1.0000** |
| *Recording Time:* | **2018-4-9 12:05** | *Sample Weight:* | **1.0000** |
| *Run Time (min):* | **30.00** | *Sample Amount:* | **1.0000** |

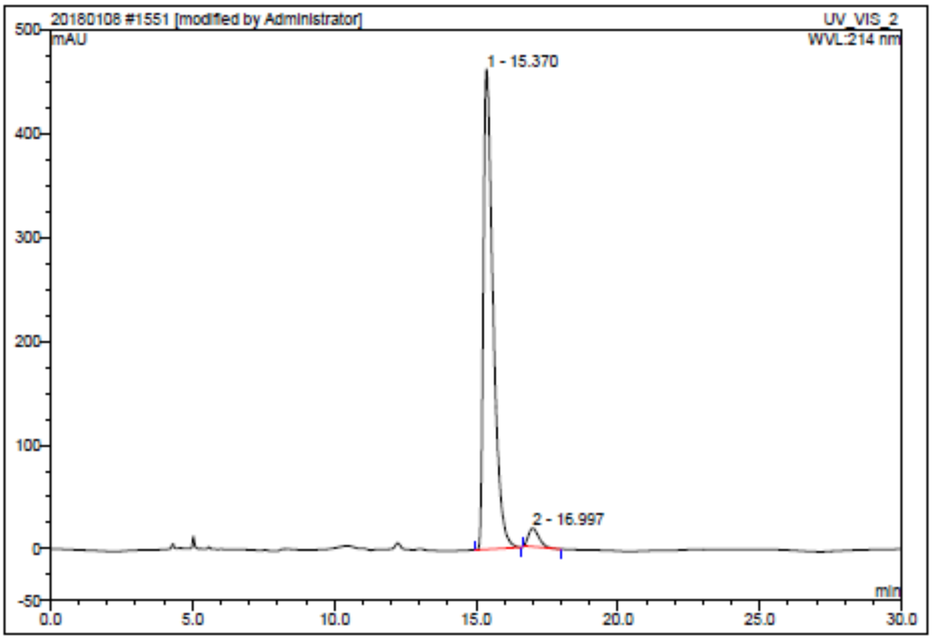


| No. | Ret.Time min | Peak Name | Height mAU | Area mAU*min | Rel.Area % | Amount | Type |
|---|---|---|---|---|---|---|---|
| 1 | 15.37 | n.a. | 462.495 | 183.199 | 96.09 | n.a. | BMB* |
| 2 | 17.00 | n.a. | 17.546 | 7.457 | 3.91 | n.a. | BMB* |
| Total: | | | 480.041 | 190.655 | 100.00 | 0.000 | |

**Supplementary Figure 14**. HPLC chromatogram for compound **3a**

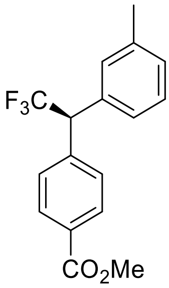


Compound **3b**. HPLC (AD-H, 0.46 × 25 cm, 5 μm, hexane/isopropanol = 95/5 (v/v %), flow 0.7 mL/min, UV detection at 214 nm), retention time = 9.02 min (minor) and 10.99 min (major). $[\alpha]_D^{25}$ = 22.9650 (c = 0.1800, $CHCl_3$, 95:5 e.r.).

Operator:Administrator Timebase:HPLC Sequence:20180108 Page 1-1
2018-5-28 3:11 下午

**2084 HWC-9-29-3+- ADH 955 214 0.7**

| | | | |
|---|---|---|---|
| *Sample Name:* | **HWC-9-29-3+- ADH 955 214 0.7** | *Injection Volume:* | **3.0** |
| *Vial Number:* | **BA5** | *Channel:* | **UV_VIS_2** |
| *Sample Type:* | **unknown** | *Wavelength:* | **214.0** |
| *Control Program:* | **test-dad6** | *Bandwidth:* | **4** |
| *Quantif. Method:* | **20170608** | *Dilution Factor:* | **1.0000** |
| *Recording Time:* | **2018-5-28 12:10** | *Sample Weight:* | **1.0000** |
| *Run Time (min):* | **15.00** | *Sample Amount:* | **1.0000** |

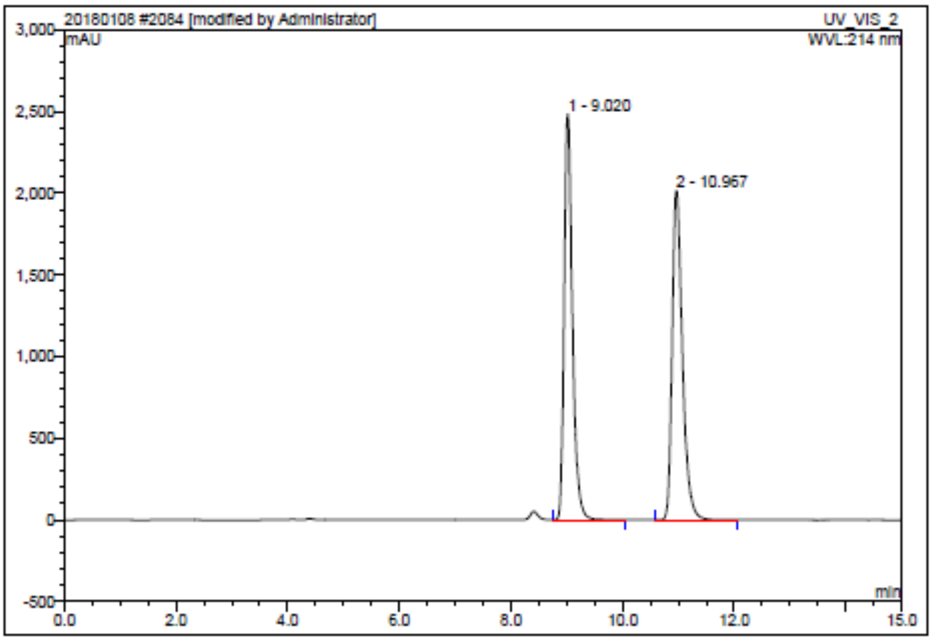


| No. | Ret.Time min | Peak Name | Height mAU | Area mAU*min | Rel.Area % | Amount | Type |
|---|---|---|---|---|---|---|---|
| 1 | 9.02 | n.a. | 2486.107 | 426.531 | 49.86 | n.a. | BMB* |
| 2 | 10.97 | n.a. | 2014.958 | 428.926 | 50.14 | n.a. | BMB* |
| **Total:** | | | 4501.065 | 855.457 | 100.00 | 0.000 | |

**Supplementary Figure 15**. HPLC chromatogram for compound **3b**, racemic

**2085 HWC-932-3C ADH 955 214 0.7**

| | | | |
|---|---|---|---|
| *Sample Name:* | **HWC-932-3C ADH 955 214 0.7** | *Injection Volume:* | **3.0** |
| *Vial Number:* | **BB5** | *Channel:* | **UV_VIS_2** |
| *Sample Type:* | **unknown** | *Wavelength:* | **214.0** |
| *Control Program:* | **test-dad6** | *Bandwidth:* | **4** |
| *Quantif. Method:* | **20170608** | *Dilution Factor:* | **1.0000** |
| *Recording Time:* | **2018-5-28 12:26** | *Sample Weight:* | **1.0000** |
| *Run Time (min):* | **15.00** | *Sample Amount:* | **1.0000** |

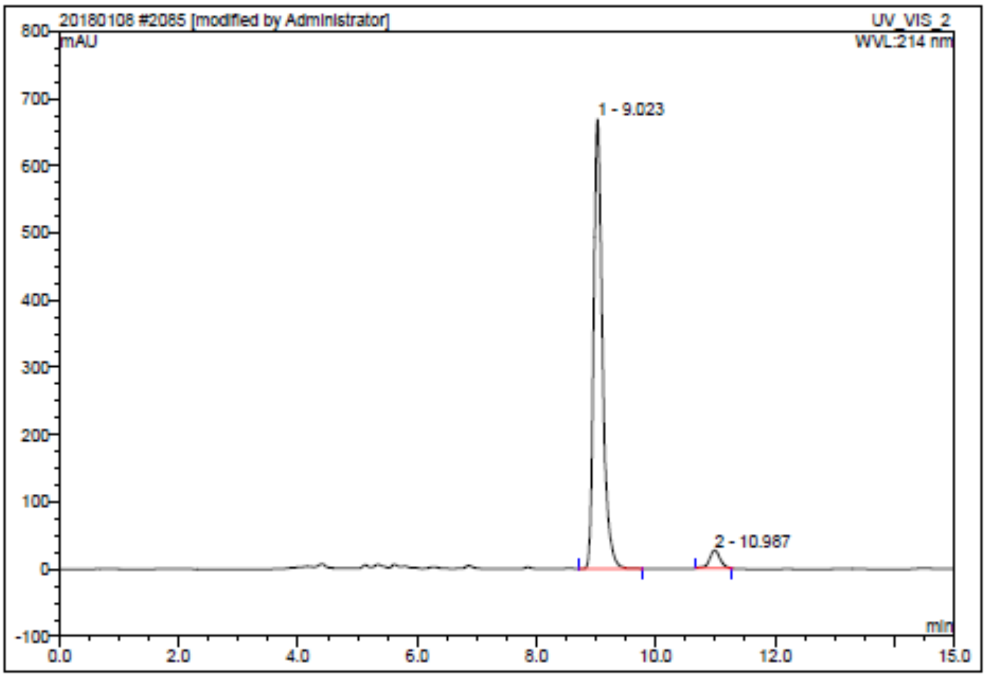


| No. | Ret.Time min | Peak Name | Height mAU | Area mAU*min | Rel.Area % | Amount | Type |
|---|---|---|---|---|---|---|---|
| 1 | 9.02 | n.a. | 668.735 | 114.709 | 95.42 | n.a. | BMB* |
| 2 | 10.99 | n.a. | 26.976 | 5.506 | 4.58 | n.a. | BMB* |
| Total: | | | 695.711 | 120.215 | 100.00 | 0.000 | |

**Supplementary Figure 16**. HPLC chromatogram for compound **3b**

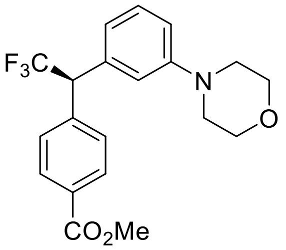


Compound **3c**. HPLC (IC, 0.46 × 25 cm, 5 μm, hexane/isopropanol = 95/5 (v/v %), flow 0.7 mL/min, UV detection at 214 nm), retention time = 20.33 min (minor) and 22.22 min (major). $[\alpha]_D^{25}$ = 62.823 (c = 0.0500, $CHCl_3$, 94:6 e.r.).

2018/5/26 9:56

**6188 HWC-9-41-3+- IC 955 214 0.7**

| | | | |
|---|---|---|---|
| *Sample Name:* | **HWC-9-41-3+- IC 955 214 0.7** | *Injection Volume:* | **3.0** |
| *Vial Number:* | **GC3** | *Channel:* | **UV_VIS_1** |
| *Sample Type:* | **unknown** | *Wavelength:* | **214** |
| *Control Program:* | **201701-6** | *Bandwidth:* | **n.a.** |
| *Quantif. Method:* | **201701** | *Dilution Factor:* | **1.0000** |
| *Recording Time:* | **2018/3/30 18:36** | *Sample Weight:* | **1.0000** |
| *Run Time (min):* | **35.00** | *Sample Amount:* | **1.0000** |

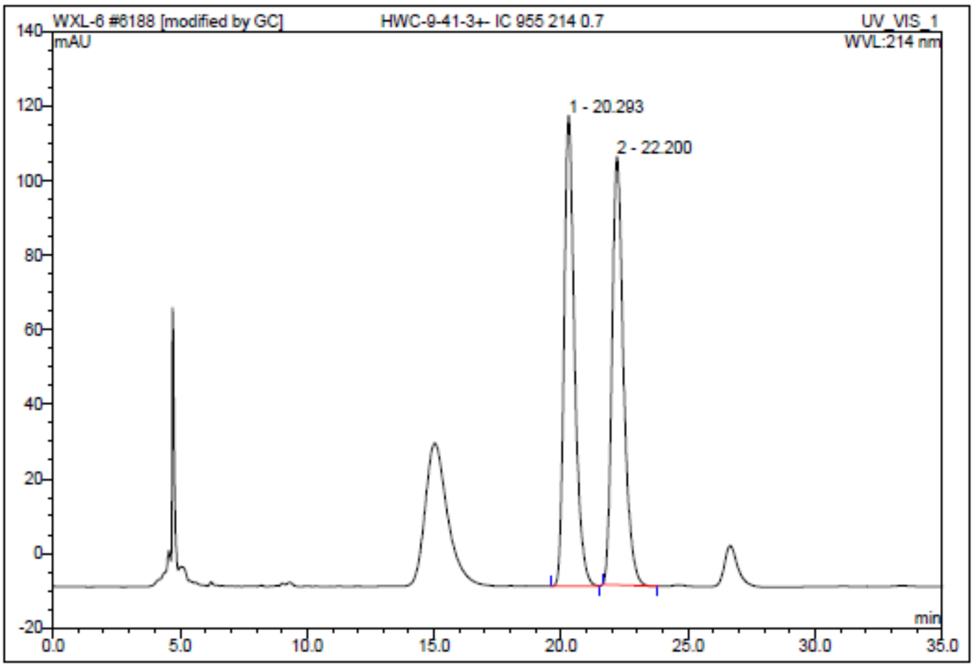


| No. | Ret.Time min | Peak Name | Height mAU | Area mAU*min | Rel.Area % | Amount | Type |
|---|---|---|---|---|---|---|---|
| 1 | 20.29 | n.a. | 126.181 | 60.519 | 50.05 | n.a. | BMB* |
| 2 | 22.20 | n.a. | 114.830 | 60.391 | 49.95 | n.a. | BMB* |
| **Total:** | | | 241.011 | 120.910 | 100.00 | 0.000 | |

**Supplementary Figure 17**. HPLC chromatogram for compound **3c**, racemic

**6189 HWC-9-37-3 IC 955 214 0.7**

| | | | |
|---|---|---|---|
| *Sample Name:* | HWC-9-37-3 IC 955 214 0.7 | *Injection Volume:* | 2.0 |
| *Vial Number:* | GC7 | *Channel:* | UV_VIS_1 |
| *Sample Type:* | unknown | *Wavelength:* | 214 |
| *Control Program:* | 201701-6 | *Bandwidth:* | n.a. |
| *Quantif. Method:* | 201701 | *Dilution Factor:* | 1.0000 |
| *Recording Time:* | 2018/3/30 19:12 | *Sample Weight:* | 1.0000 |
| *Run Time (min):* | 35.00 | *Sample Amount:* | 1.0000 |

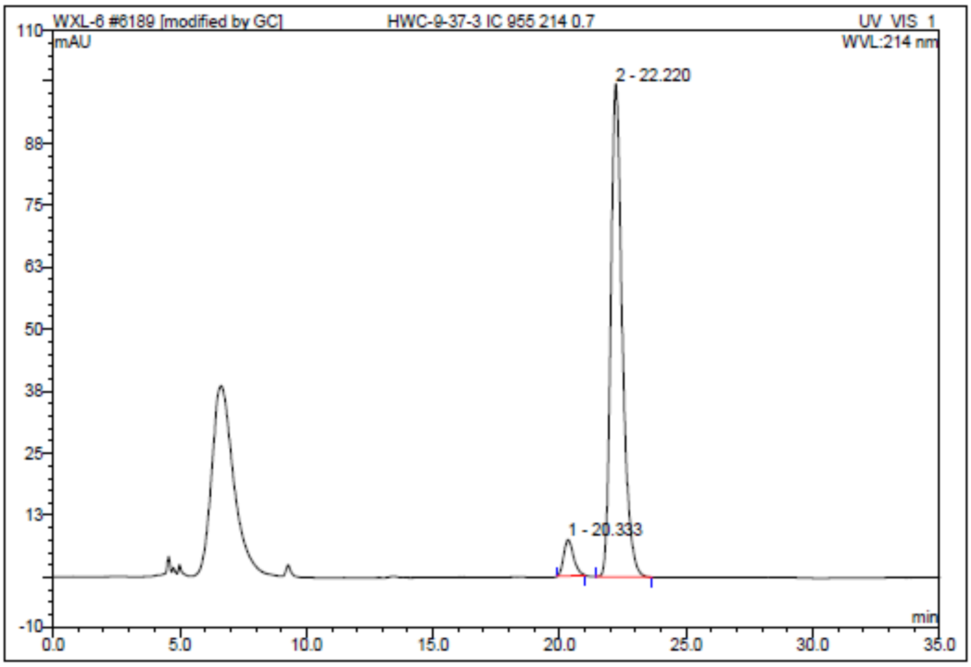


| No. | Ret.Time min | Peak Name | Height mAU | Area mAU*min | Rel.Area % | Amount | Type |
|---|---|---|---|---|---|---|---|
| 1 | 20.33 | n.a. | 7.309 | 3.303 | 5.94 | n.a. | BMB* |
| 2 | 22.22 | n.a. | 99.325 | 52.270 | 94.06 | n.a. | BMB* |
| Total: | | | 106.634 | 55.572 | 100.00 | 0.000 | |

**Supplementary Figure 18**. HPLC chromatogram for compound **3c**

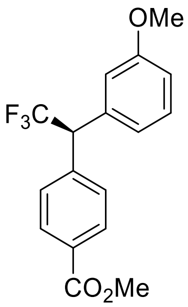


Compound **3d**. HPLC (OD-H, 0.46 × 25 cm, 5 µm, hexane/isopropanol = 95/5 (v/v %), flow 0.7 mL/min, UV detection at 214 nm) retention time = 10.56 min (minor) and 13.03 min (major). $[\alpha]_D^{25}$ = 15.9011 (c = 0.1750, $CHCl_3$, 95:5 e.r.).

Operator:Administrator Timebase:HPLC Sequence:20180108 Page

2018-5-26 10:51

**872 HWC-9-18-3B ODH 955 214 0.7**

| | | | |
|---|---|---|---|
| *Sample Name:* | HWC-9-18-3B ODH 955 214 0.7 | *Injection Volume:* | 3.0 |
| *Vial Number:* | RD7 | *Channel:* | UV_VIS_2 |
| *Sample Type:* | unknown | *Wavelength:* | 214.0 |
| *Control Program:* | test-dad4 | *Bandwidth:* | 4 |
| *Quantif. Method:* | 20170608 | *Dilution Factor:* | 1.0000 |
| *Recording Time:* | 2018-3-1 21:04 | *Sample Weight:* | 1.0000 |
| *Run Time (min):* | 19.00 | *Sample Amount:* | 1.0000 |

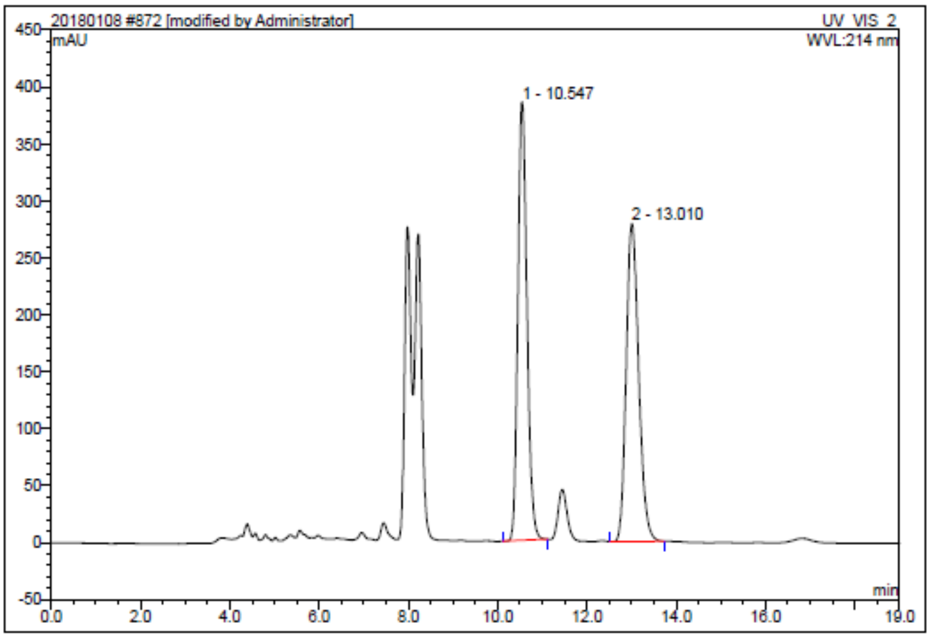


| No. | Ret.Time min | Peak Name | Height mAU | Area mAU*min | Rel.Area % | Amount | Type |
|---|---|---|---|---|---|---|---|
| 1 | 10.55 | n.a. | 384.960 | 90.470 | 50.03 | n.a. | BMB* |
| 2 | 13.01 | n.a. | 279.173 | 90.364 | 49.97 | n.a. | BMB* |
| **Total:** | | | 664.133 | 180.834 | 100.00 | 0.000 | |

**Supplementary Figure 19**. HPLC chromatogram for compound **3d**, racemic

**873 HWC-9-27-3B ODH 955 214 0.7**

| | | | |
|---|---|---|---|
| *Sample Name:* | HWC-9-27-3B ODH 955 214 0.7 | *Injection Volume:* | 2.0 |
| *Vial Number:* | RE7 | *Channel:* | UV_VIS_2 |
| *Sample Type:* | unknown | *Wavelength:* | 214.0 |
| *Control Program:* | test-dad4 | *Bandwidth:* | 4 |
| *Quantif. Method:* | 20170608 | *Dilution Factor:* | 1.0000 |
| *Recording Time:* | 2018-3-1 21:23 | *Sample Weight:* | 1.0000 |
| *Run Time (min):* | 19.00 | *Sample Amount:* | 1.0000 |

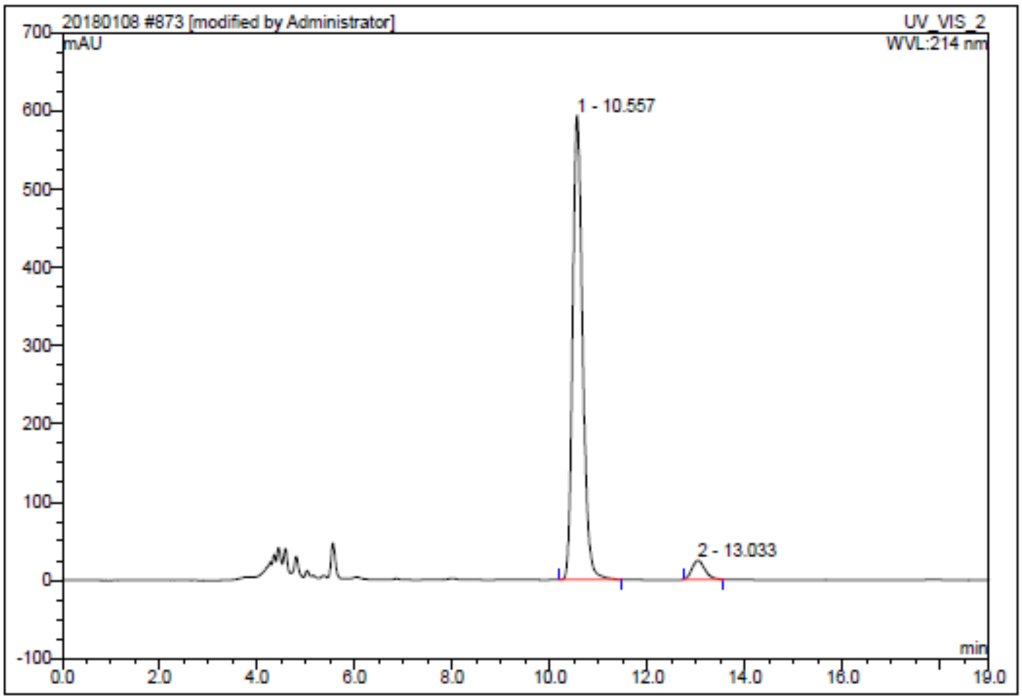


| No. | Ret.Time min | Peak Name | Height mAU | Area mAU*min | Rel.Area % | Amount | Type |
|---|---|---|---|---|---|---|---|
| 1 | 10.56 | n.a. | 593.139 | 140.324 | 94.95 | n.a. | BMB* |
| 2 | 13.03 | n.a. | 24.203 | 7.463 | 5.05 | n.a. | BMB* |
| Total: | | | 617.342 | 147.787 | 100.00 | 0.000 | |

**Supplementary Figure 20**. HPLC chromatogram for compound **3d**

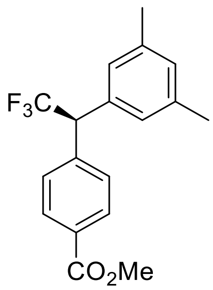

Compound **3e**. HPLC (AD-H, 0.46 × 25 cm, 5 µm, hexane/isopropanol = 95/5 (v/v %), flow 0.7 mL/min, UV detection at 214 nm) retention time = 7.48 min (minor) and 9.45 min (major). $[\alpha]_D^{25}$ = 29.9726 (c = 0.0800, $CHCl_3$, 94:6 e.r.).

**954 HWC-9-13-3C ADH 955 214 0.7**

| | | | |
|---|---|---|---|
| *Sample Name:* | HWC-9-13-3C ADH 955 214 0.7 | *Injection Volume:* | 1.0 |
| *Vial Number:* | RC5 | *Channel:* | UV_VIS_2 |
| *Sample Type:* | unknown | *Wavelength:* | 214.0 |
| *Control Program:* | test-dad4 | *Bandwidth:* | 4 |
| *Quantif. Method:* | 20170608 | *Dilution Factor:* | 1.0000 |
| *Recording Time:* | 2018-3-6 17:37 | *Sample Weight:* | 1.0000 |
| *Run Time (min):* | 17.00 | *Sample Amount:* | 1.0000 |

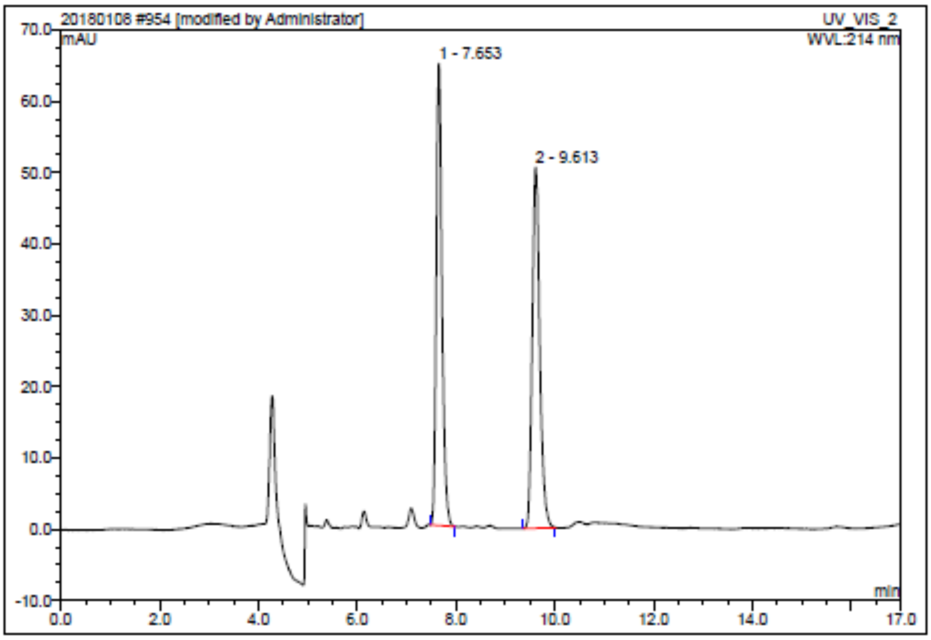


| No. | Ret.Time min | Peak Name | Height mAU | Area mAU*min | Rel.Area % | Amount | Type |
|---|---|---|---|---|---|---|---|
| 1 | 7.65 | n.a. | 64.760 | 8.786 | 49.67 | n.a. | BMB |
| 2 | 9.61 | n.a. | 50.613 | 8.904 | 50.33 | n.a. | BMB |
| Total: | | | 115.373 | 17.690 | 100.00 | 0.000 | |

**Supplementary Figure 21**. HPLC chromatogram for compound **3e**, racemic

Operator:Administrator Timebase:HPLC Sequence:20180108 Page 1-
2018-5-26 10:27 [illegible]

| 955 HWC-9-38-3 ADH 955 214 0.7 |
|---|

| | | | |
|---|---|---|---|
| *Sample Name:* | **HWC-9-38-3 ADH 955 214 0.7** | *Injection Volume:* | **1.0** |
| *Vial Number:* | **RB5** | *Channel:* | **UV_VIS_2** |
| *Sample Type:* | **unknown** | *Wavelength:* | **214.0** |
| *Control Program:* | **test-dad4** | *Bandwidth:* | **4** |
| *Quantif. Method:* | **20170608** | *Dilution Factor:* | **1.0000** |
| *Recording Time:* | **2018-3-6 17:55** | *Sample Weight:* | **1.0000** |
| *Run Time (min):* | **17.00** | *Sample Amount:* | **1.0000** |

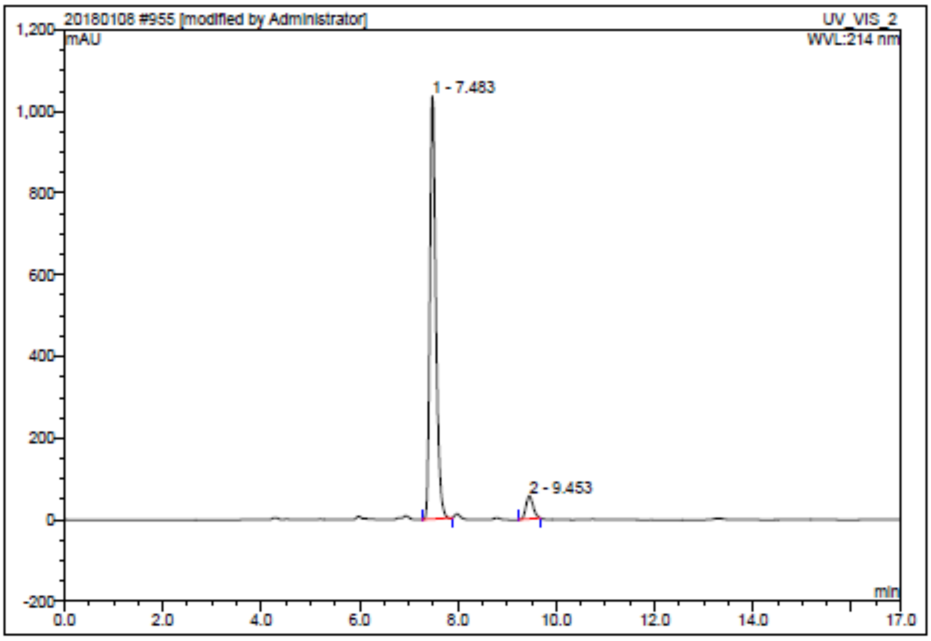


| No. | Ret.Time min | Peak Name | Height mAU | Area mAU*min | Rel.Area % | Amount | Type |
|---|---|---|---|---|---|---|---|
| 1 | 7.48 | n.a. | 1035.403 | 145.807 | 93.99 | n.a. | BMB* |
| 2 | 9.45 | n.a. | 55.983 | 9.325 | 6.01 | n.a. | BMB* |
| Total: | | | 1091.386 | 155.132 | 100.00 | 0.000 | |

**Supplementary Figure 22**. HPLC chromatogram for compound **3e**

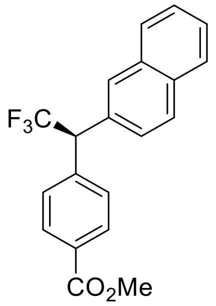


Compound **3f**. SFC (OJ-H, 0.46 × 25 cm, 5 μm, $CO_2$/MeOH = 98/2, flow 2.0 mL/min, column temperature: 40 °C, background press: 2000 psi,UV detection at 214 nm) retention time = 17.17 min (major) and 25.88 min (minor). $[\alpha]_D^{25}$ = 4.9725 (c = 0.1450, $CHCl_3$, 92:8 e.r.).

Empower™ 3 SOFTWARE

SAMPLE INFORMATION

| | | | |
|---|---|---|---|
| Sample Name: | HWC-9-72-3B+- OJH9822141200040 | Acquired By: | System |
| Sample Type: | | Date Acquired: | 2018/3/22 9:53:41 CST |
| Vial: | 1:A,7 | Acq. Method Set: | chiral_isocratic |
| Injection: | 1 | Date Processed: | 2018/5/28 9:15:46 CST |
| Injection Volume: | 2.00 ul | Processing Method | 1 |
| Run Time: | 36.0 Minutes | Channel Name: | PDA Ch1 214 nm@1.2 nm |
| Sample Set Name | 20180306 | Proc. Chnl. Descr: | PDA Ch1 214 nm@1.2 nm |

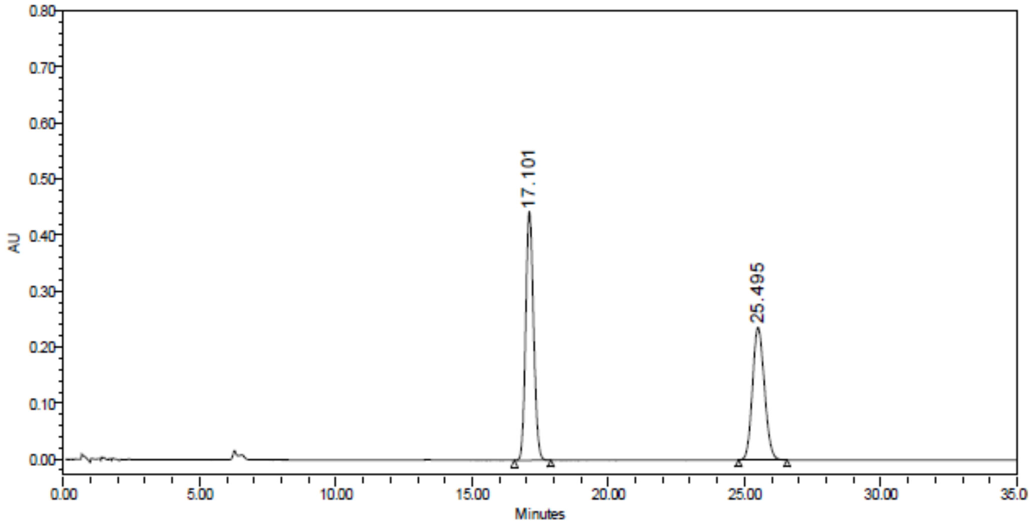


Peak Results

| | RT | Area | Height | % Area |
|---|---|---|---|---|
| 1 | 17.101 | 8931775 | 443665 | 55.30 |
| 2 | 25.495 | 7219697 | 236783 | 44.70 |

**Supplementary Figure 23**. HPLC chromatogram for compound **3f**, racemic

SAMPLE INFORMATION

| | | | |
|---|---|---|---|
| Sample Name: | HWC-9-72-3A OJH9822141200040 | Acquired By: | System |
| Sample Type: | | Date Acquired: | 2018/3/22 11:07:55 CST |
| Vial: | 1:A,6 | Acq. Method Set: | chiral_isocratic |
| Injection: | 1 | Date Processed: | 2018/5/28 9:16:22 CST |
| Injection Volume: | 1.00 ul | Processing Method | 1 |
| Run Time: | 36.0 Minutes | Channel Name: | PDA Ch1 214 nm@1.2 nm |
| Sample Set Name | 20180306 | Proc. Chnl. Descr. | PDA Ch1 214 nm@1.2 nm |

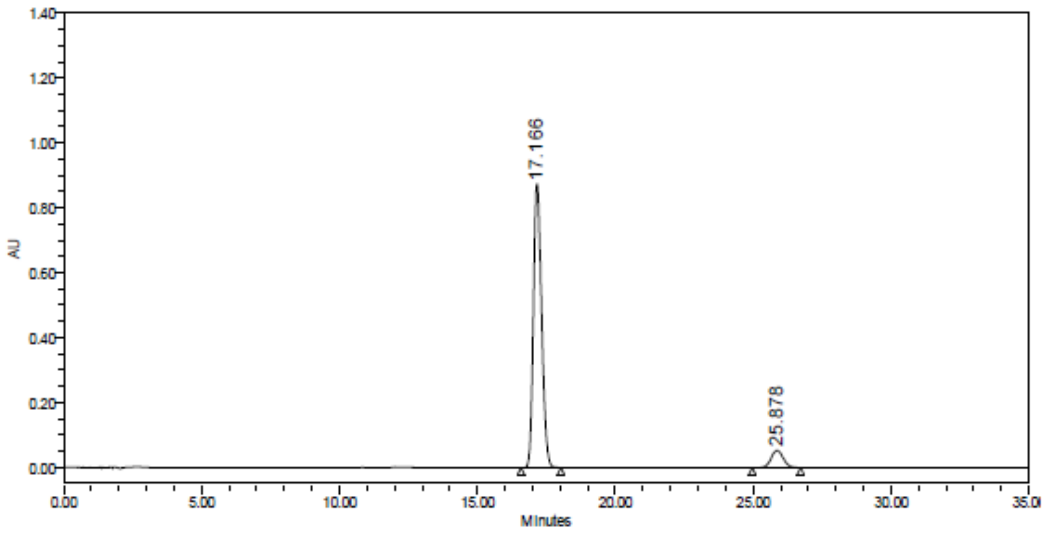


Peak Results

| | RT | Area | Height | % Area |
|---|---|---|---|---|
| 1 | 17.166 | 18125312 | 875384 | 91.66 |
| 2 | 25.878 | 1649581 | 53436 | 8.34 |

**Supplementary Figure 24**. HPLC chromatogram for compound **3f**

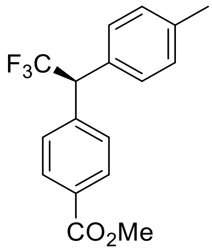


Compound **3g**. HPLC (IG, 0.46 × 25 cm, 5 µm, hexane/isopropanol = 90/10 (v/v %), flow 0.7 mL/min, UV detection at 214 nm), retention time = 8.99 min (minor) and 9.46 min (major). $[\alpha]_D^{25}$ = 11.7500 (c = 0.2400, $CHCl_3$, 93:7 e.r.).

Empower 3 SOFTWARE 1

## SAMPLE INFORMATION

| | | | |
|---|---|---|---|
| Sample Name: | HWC-14-19-3+- IG 91 214 0.7 | Acquired By: | System |
| Sample Type: | Unknown | Sample Set Name: | 1 |
| Vial: | 1:E,5 | Acq. Method Set: | 1017 |
| Injection #: | 1 | Processing Method: | 1 |
| Injection Volume: | 4.00 ul | Channel Name: | PDA Ch1 214nm@4.8nm |
| Run Time: | 135.0 Minutes | Proc. Chnl. Descr.: | PDA Ch1 214nm@4.8nm |
| Date Acquired: | 3/29/2019 3:29:11 PM CST | | |
| Date Processed: | 3/29/2019 4:56:35 PM CST | | |

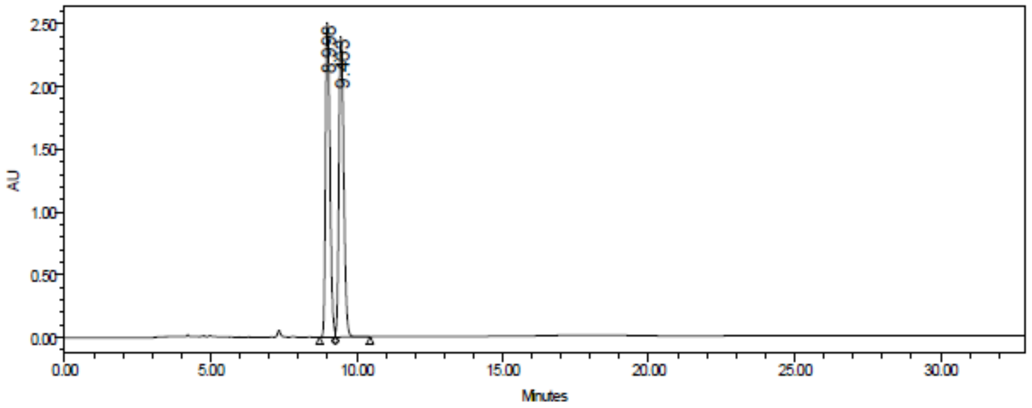


| | RT | Area | % Area | Height |
|---|---|---|---|---|
| 1 | 8.998 | 26796010 | 49.42 | 2501819 |
| 2 | 9.463 | 27426815 | 50.58 | 2389724 |

**Supplementary Figure 25**. HPLC chromatogram for compound **3g**, racemic

Empower 3 SOFTWARE



## SAMPLE INFORMATION

| | | | |
|---|---|---|---|
| Sample Name: | HWC-14-20-3B IG91 214 0.7 | Acquired By: | System |
| Sample Type: | Unknown | Sample Set Name: | 1 |
| Vial: | 1:F,5 | Acq. Method Set: | 1017 |
| Injection #: | 1 | Processing Method: | 1 |
| Injection Volume: | 4.00 ul | Channel Name: | PDA Ch1 214nm@4.8nm |
| Run Time: | 135.0 Minutes | Proc. Chnl. Descr.: | PDA Ch1 214nm@4.8nm |
| Date Acquired: | 3/29/2019 4:04:18 PM CST | | |
| Date Processed: | 3/29/2019 4:56:04 PM CST | | |

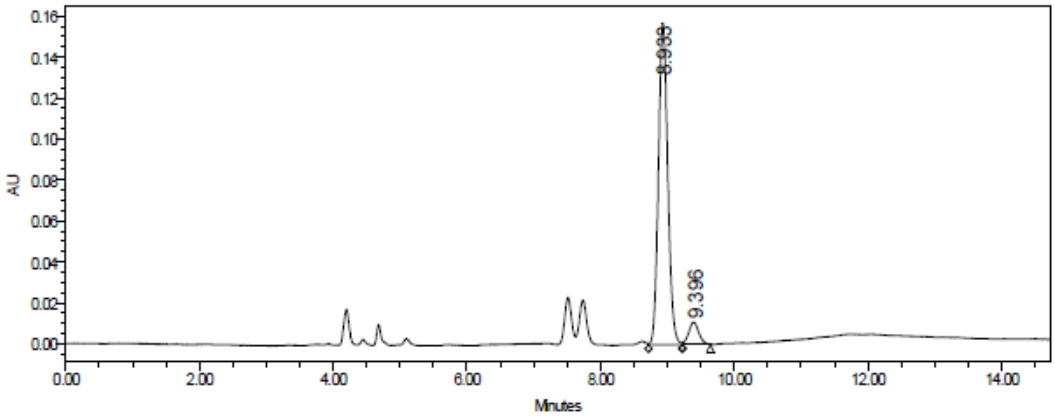


| | RT | Area | % Area | Height |
|---|---|---|---|---|
| 1 | 8.933 | 1520114 | 92.86 | 157188 |
| 2 | 9.396 | 116844 | 7.14 | 10981 |

**Supplementary Figure 26**. HPLC chromatogram for compound **3g**

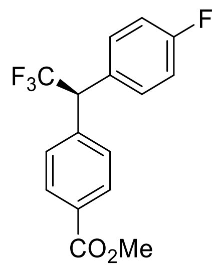

Compound **3h**. HPLC (IF3, 0.46 × 25 cm, 5 µm, hexane/isopropanol = 98/2 (v/v %), flow 0.7 mL/min, UV detection at 214 nm), retention time = 11.49 min (minor) and 12.26 min (major). $[\alpha]_D^{25}$ = 22.2000 (c = 0.4000, $CHCl_3$, 96:4 e.r.).

**5277 HWC-14-13-3B IF3 982 214 0.7**

| | | | |
|---|---|---|---|
| *Sample Name:* | HWC-14-13-3B IF3 982 214 0.7 | *Injection Volume:* | 5.0 |
| *Vial Number:* | RC4 | *Channel:* | UV_VIS_2 |
| *Sample Type:* | unknown | *Wavelength:* | 214.0 |
| *Control Program:* | test-dad4 | *Bandwidth:* | 4 |
| *Quantif. Method:* | 20170608 | *Dilution Factor:* | 1.0000 |
| *Recording Time:* | 2019-3-27 19:23 | *Sample Weight:* | 1.0000 |
| *Run Time (min):* | 20.22 | *Sample Amount:* | 1.0000 |

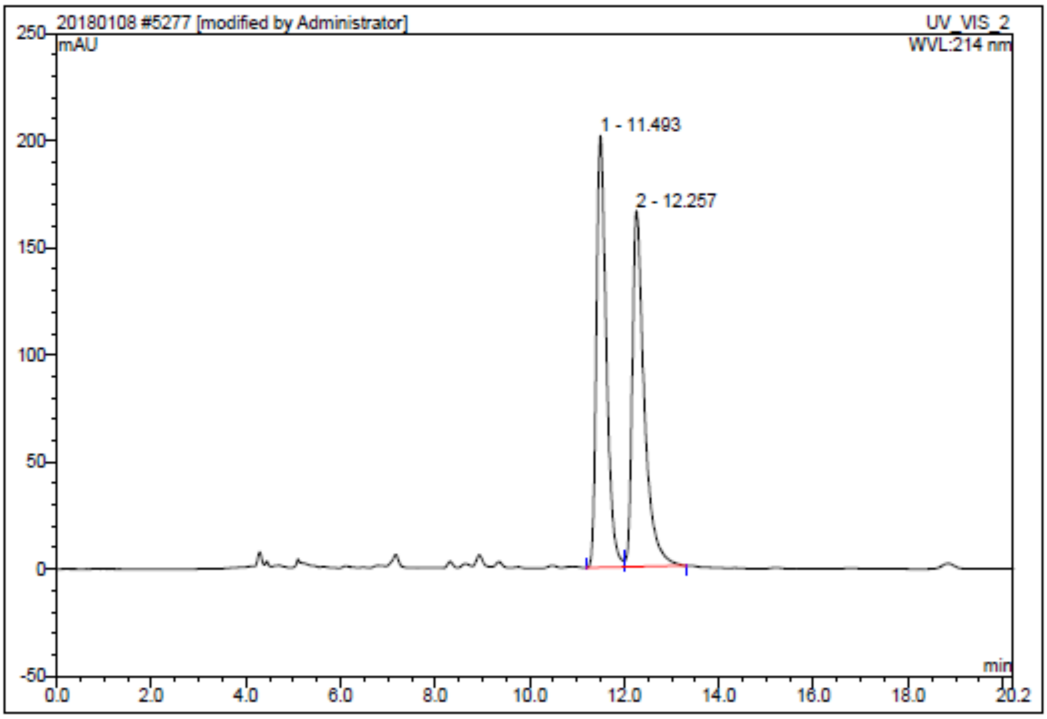


| No. | Ret.Time min | Peak Name | Height mAU | Area mAU*min | Rel.Area % | Amount | Type |
|---|---|---|---|---|---|---|---|
| 1 | 11.49 | n.a. | 201.503 | 48.996 | 50.06 | n.a. | BM |
| 2 | 12.26 | n.a. | 165.997 | 48.874 | 49.94 | n.a. | MB |
| **Total:** | | | 367.500 | 97.870 | 100.00 | 0.000 | |

**Supplementary Figure 27**. HPLC chromatogram for compound **3h**, racemic

## 5278 HWC-14-13-3A IF3 982 214 0.7

| | | | |
|---|---|---|---|
| *Sample Name:* | **HWC-14-13-3A IF3 982 214 0.7** | *Injection Volume:* | **5.0** |
| *Vial Number:* | **RC5** | *Channel:* | **UV_VIS_2** |
| *Sample Type:* | **unknown** | *Wavelength:* | **214.0** |
| *Control Program:* | **test-dad4** | *Bandwidth:* | **4** |
| *Quantif. Method:* | **20170608** | *Dilution Factor:* | **1.0000** |
| *Recording Time:* | **2019-3-28 8:43** | *Sample Weight:* | **1.0000** |
| *Run Time (min):* | **15.00** | *Sample Amount:* | **1.0000** |

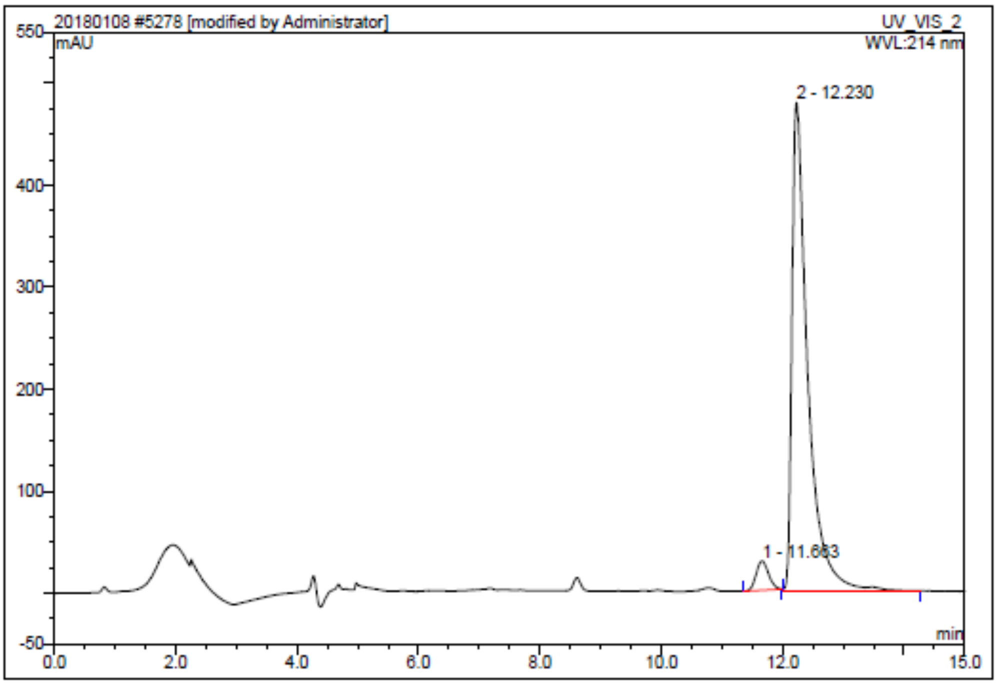


| No. | Ret.Time min | Peak Name | Height mAU | Area mAU*min | Rel.Area % | Amount | Type |
|---|---|---|---|---|---|---|---|
| 1 | 11.66 | n.a. | 28.473 | 6.729 | 4.46 | n.a. | BMB* |
| 2 | 12.23 | n.a. | 478.688 | 144.113 | 95.54 | n.a. | MB* |
| **Total:** | | | 507.162 | 150.841 | 100.00 | 0.000 | |

**Supplementary Figure 28**. HPLC chromatogram for compound **3h**

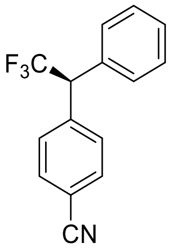


Compound **3j**. HPLC (OJ-H, 0.46 × 25 cm, 5 μm, hexane/isopropanol = 98/2 (v/v %), flow 0.7 mL/min, UV detection at 214 nm), retention time = 33.32 min (minor) and 50.62 min (major). $[\alpha]_D^{25}$ = 11.1247 (c = 0.3000, $CHCl_3$, 94:6 e.r.).

**486 HWC-8-32-3A+- OJH 982 214 0.7**

| | | | |
|---|---|---|---|
| *Sample Name:* | HWC-8-32-3A+- OJH 982 214 0.7 | *Injection Volume:* | 3.0 |
| *Vial Number:* | GA3 | *Channel:* | UV_VIS_2 |
| *Sample Type:* | unknown | *Wavelength:* | 214.0 |
| *Control Program:* | test-dad6 | *Bandwidth:* | 4 |
| *Quantif. Method:* | 20170608 | *Dilution Factor:* | 1.0000 |
| *Recording Time:* | 2018-1-23 12:46 | *Sample Weight:* | 1.0000 |
| *Run Time (min):* | 74.30 | *Sample Amount:* | 1.0000 |

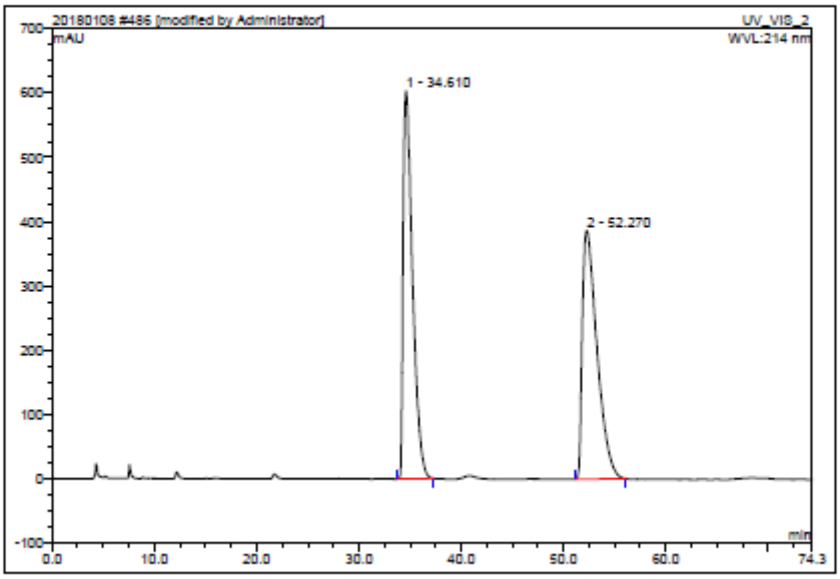


| No. | Ret.Time min | Peak Name | Height mAU | Area mAU*min | Rel.Area % | Amount | Type |
|---|---|---|---|---|---|---|---|
| 1 | 34.61 | n.a. | 604.009 | 642.469 | 49.95 | n.a. | BMB |
| 2 | 52.27 | n.a. | 386.549 | 643.643 | 50.05 | n.a. | BMB |
| Total: | | | 990.559 | 1286.112 | 100.00 | 0.000 | |

**Supplementary Figure 29**. HPLC chromatogram for compound **3j**, racemic

**487 HWC-9-1-3C OJH 982 214 0.7**

| | | | |
|---|---|---|---|
| *Sample Name:* | **HWC-9-1-3C OJH 982 214 0.7** | *Injection Volume:* | **1.0** |
| *Vial Number:* | **GB3** | *Channel:* | **UV_VIS_2** |
| *Sample Type:* | **unknown** | *Wavelength:* | **214.0** |
| *Control Program:* | **test-dad6** | *Bandwidth:* | **4** |
| *Quantif. Method:* | **20170608** | *Dilution Factor:* | **1.0000** |
| *Recording Time:* | **2018-1-23 16:22** | *Sample Weight:* | **1.0000** |
| *Run Time (min):* | **69.80** | *Sample Amount:* | **1.0000** |

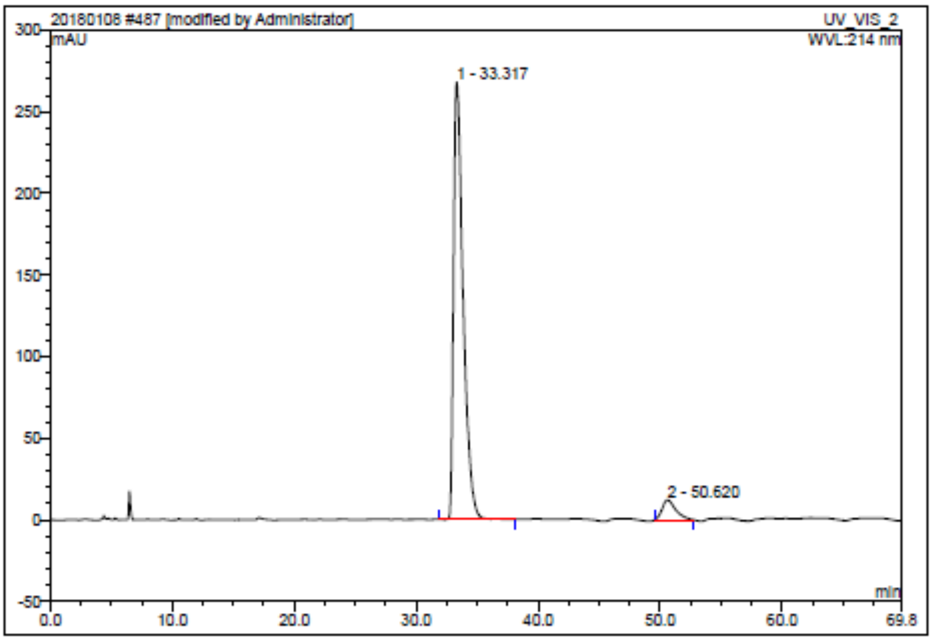


| No. | Ret.Time min | Peak Name | Height mAU | Area mAU*min | Rel.Area % | Amount | Type |
|---|---|---|---|---|---|---|---|
| 1 | 33.32 | n.a. | 267.697 | 242.864 | 93.90 | n.a. | BMB* |
| 2 | 50.62 | n.a. | 12.147 | 15.787 | 6.10 | n.a. | BMB* |
| Total: | | | 279.844 | 258.652 | 100.00 | 0.000 | |

**Supplementary Figure 30**. HPLC chromatogram for compound **3j**, racemic

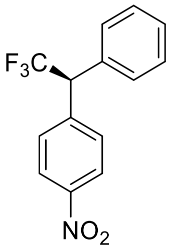


Compound **3k**. HPLC (IG, 0.46 × 25 cm, 5 µm, hexane/isopropanol = 99/1 (v/v %), flow 0.7 mL/min, UV detection at 214 nm), retention time = 13.89 min (minor) and 14.80 min (major). $[\alpha]_D^{25}$ = 35.1192 (c = 0.0700, $CHCl_3$, 96:4 e.r.).

**5263 HWC-8-102-3D IG 991 214 0.7**

| | | | |
|---|---|---|---|
| *Sample Name:* | **HWC-8-102-3D IG 991 214 0.7** | *Injection Volume:* | **0.5** |
| *Vial Number:* | **BA5** | *Channel:* | **UV_VIS_1** |
| *Sample Type:* | **unknown** | *Wavelength:* | **214** |
| *Control Program:* | **201701-4** | *Bandwidth:* | **n.a.** |
| *Quantif. Method:* | **201701** | *Dilution Factor:* | **1.0000** |
| *Recording Time:* | **2018/1/18 23:24** | *Sample Weight:* | **1.0000** |
| *Run Time (min):* | **18.00** | *Sample Amount:* | **1.0000** |

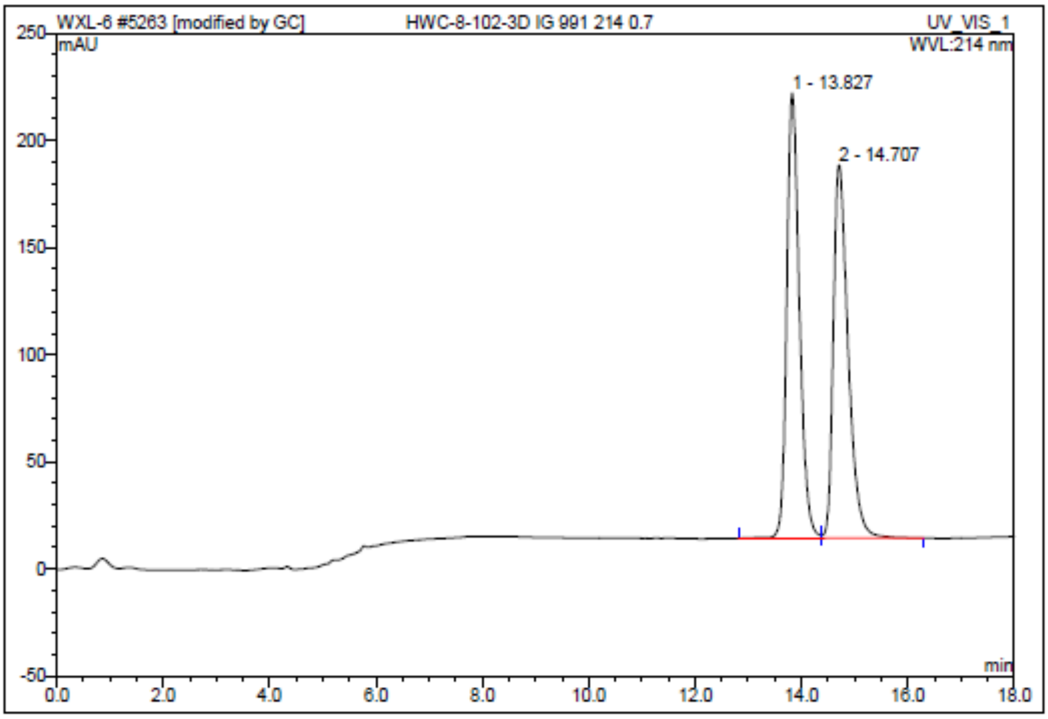


| No. | Ret.Time min | Peak Name | Height mAU | Area mAU*min | Rel.Area % | Amount | Type |
|---|---|---|---|---|---|---|---|
| 1 | 13.83 | n.a. | 207.601 | 57.268 | 50.50 | n.a. | BM |
| 2 | 14.71 | n.a. | 174.040 | 56.130 | 49.50 | n.a. | MB |
| Total: | | | 381.641 | 113.397 | 100.00 | 0.000 | |

**Supplementary Figure 31**. HPLC chromatogram for compound **3k**, racemic

**5264 HWC-8-103-3A IG 991 214 0.7**

| | | | |
|---|---|---|---|
| *Sample Name:* | HWC-8-103-3A IG 991 214 0.7 | *Injection Volume:* | 0.5 |
| *Vial Number:* | BA6 | *Channel:* | UV_VIS_1 |
| *Sample Type:* | unknown | *Wavelength:* | 214 |
| *Control Program:* | 201701-4 | *Bandwidth:* | n.a. |
| *Quantif. Method:* | 201701 | *Dilution Factor:* | 1.0000 |
| *Recording Time:* | 2018/1/18 23:44 | *Sample Weight:* | 1.0000 |
| *Run Time (min):* | 18.00 | *Sample Amount:* | 1.0000 |

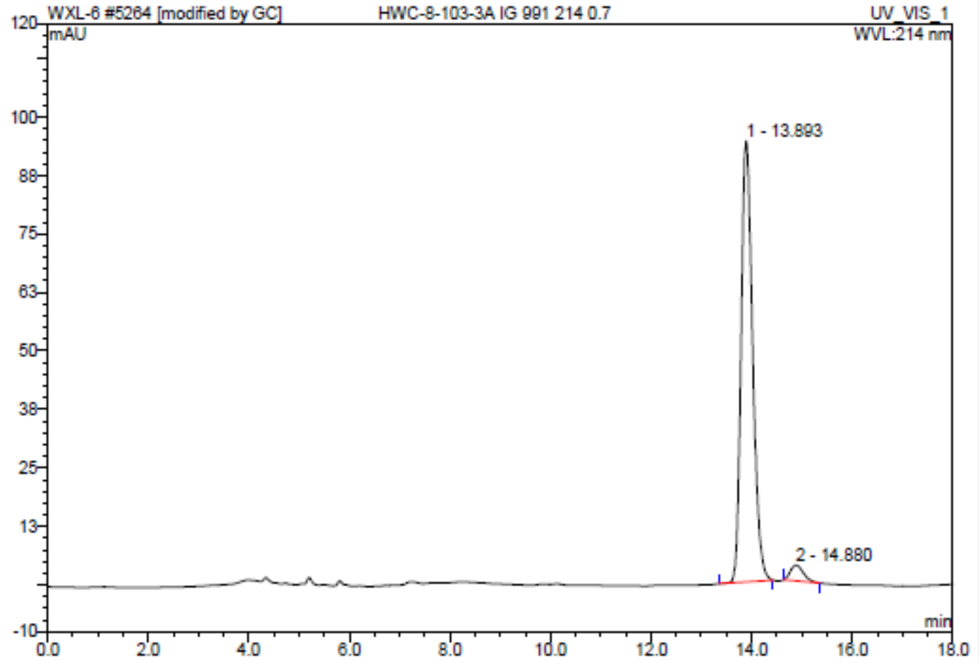


| No. | Ret.Time min | Peak Name | Height mAU | Area mAU*min | Rel.Area % | Amount | Type |
|---|---|---|---|---|---|---|---|
| 1 | 13.89 | n.a. | 94.287 | 24.922 | 96.17 | n.a. | BMB* |
| 2 | 14.88 | n.a. | 3.345 | 0.992 | 3.83 | n.a. | BMB* |
| Total: | | | 97.632 | 25.914 | 100.00 | 0.000 | |

**Supplementary Figure 32**. HPLC chromatogram for compound **3k**

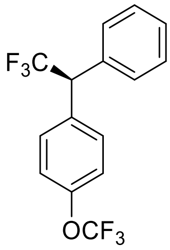


Compound **3l**. HPLC (OJ-H, 0.46 × 25 cm, 5 μm, hexane/isopropanol = 98/2 (v/v %), flow 0.7 mL/min,UV detection at 214 nm), retention time = 7.61 min (minor) and 8.93 min (major). $[\alpha]_D^{25}$ = 2.6503 (c = 0.0600, $CHCl_3$, 95:5 e.r.).

**461 HWC-8-49-3C OJH 982 214 0.7**

| | | | |
|---|---|---|---|
| *Sample Name:* | HWC-8-49-3C OJH 982 214 0.7 | *Injection Volume:* | 2.0 |
| *Vial Number:* | GE6 | *Channel:* | UV_VIS_2 |
| *Sample Type:* | unknown | *Wavelength:* | 214.0 |
| *Control Program:* | test-dad6 | *Bandwidth:* | 4 |
| *Quantif. Method:* | 20170608 | *Dilution Factor:* | 1.0000 |
| *Recording Time:* | 2018-1-22 16:39 | *Sample Weight:* | 1.0000 |
| *Run Time (min):* | 11.92 | *Sample Amount:* | 1.0000 |

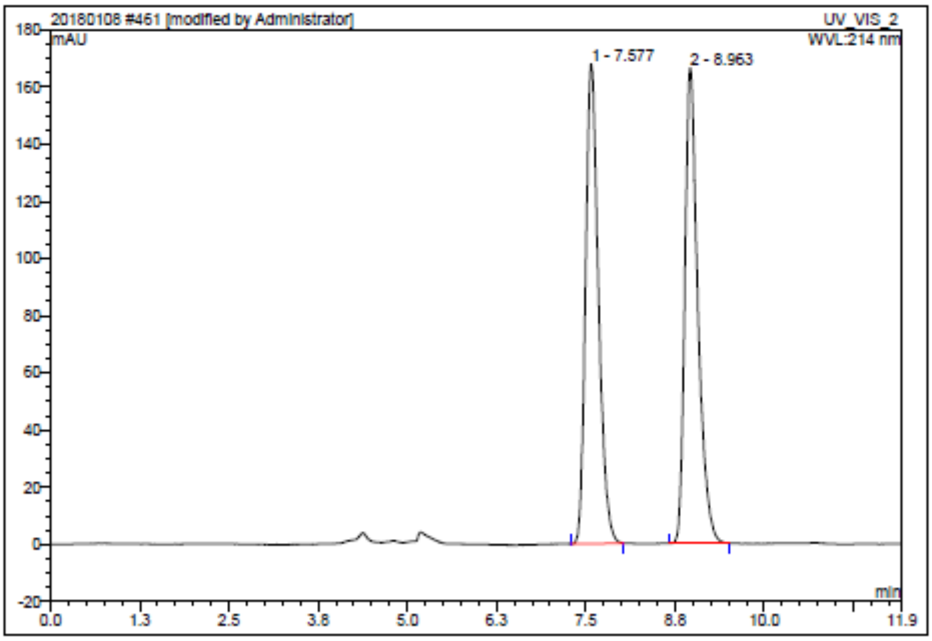


| No. | Ret.Time min | Peak Name | Height mAU | Area mAU*min | Rel.Area % | Amount | Type |
|---|---|---|---|---|---|---|---|
| 1 | 7.58 | n.a. | 167.815 | 35.096 | 49.96 | n.a. | BMB |
| 2 | 8.96 | n.a. | 166.321 | 35.156 | 50.04 | n.a. | BMB |
| Total: | | | 334.136 | 70.252 | 100.00 | 0.000 | |

**Supplementary Figure 33**. HPLC chromatogram for compound **3l**, racemic

| 462 HWC-9-1-3D OJH 982 214 0.7 | | | |
|---|---|---|---|
| *Sample Name:* | HWC-9-1-3D OJH 982 214 0.7 | *Injection Volume:* | 2.0 |
| *Vial Number:* | GC4 | *Channel:* | UV_VIS_2 |
| *Sample Type:* | unknown | *Wavelength:* | 214.0 |
| *Control Program:* | test-dad6 | *Bandwidth:* | 4 |
| *Quantif. Method:* | 20170608 | *Dilution Factor:* | 1.0000 |
| *Recording Time:* | 2018-1-22 18:26 | *Sample Weight:* | 1.0000 |
| *Run Time (min):* | 19.44 | *Sample Amount:* | 1.0000 |

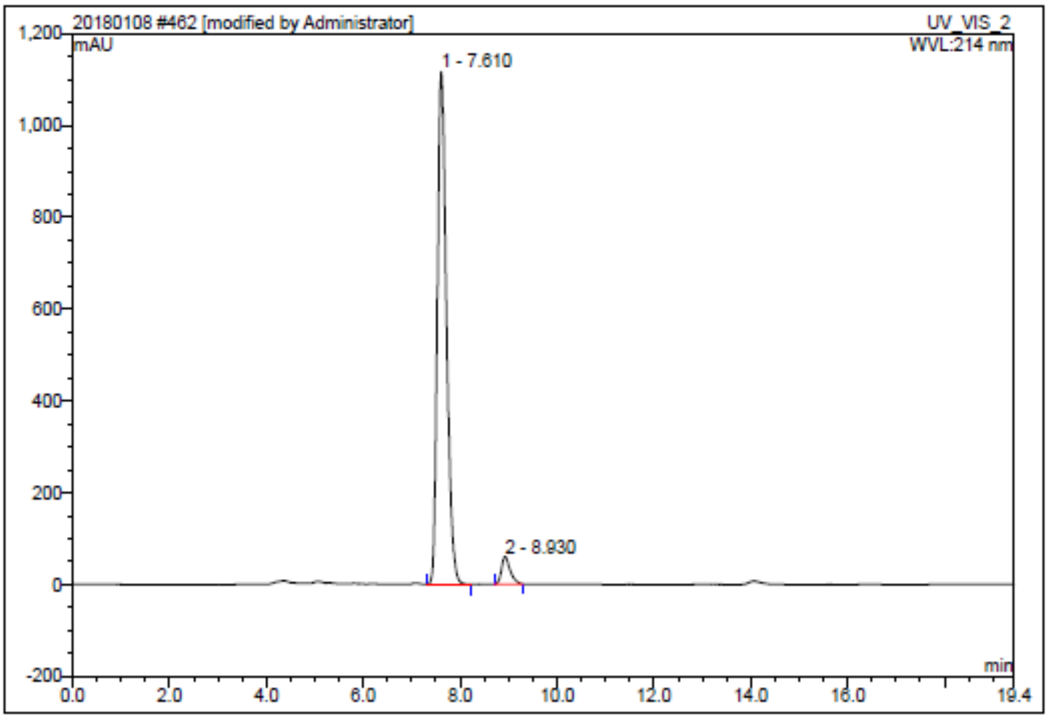


| No. | Ret.Time min | Peak Name | Height mAU | Area mAU*min | Rel.Area % | Amount | Type |
|---|---|---|---|---|---|---|---|
| 1 | 7.61 | n.a. | 1115.766 | 242.540 | 95.19 | n.a. | BMB* |
| 2 | 8.93 | n.a. | 59.599 | 12.258 | 4.81 | n.a. | BMB* |
| Total: | | | 1175.365 | 254.798 | 100.00 | 0.000 | |

**Supplementary Figure 34**. HPLC chromatogram for compound **3l**

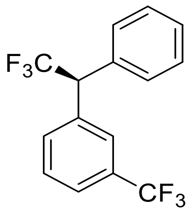


Compound **3m**. SFC (OJ-H, 0.46 × 25 cm, 5 μm, $CO_2$/MeOH = 99.5/0.5, flow 2.0 mL/min, column temperature: 40 ºC, background press: 2000 psi, UV detection at 214 nm), retention time = 2.24 min (major) and 2.37 min (minor). $[\alpha]_D^{25}$ = -7.9503 (c = 0.0750, $CHCl_3$, 97:3 e.r.).

Empower 3 SOFTWARE

SAMPLE INFORMATION

| | | | |
|---|---|---|---|
| Sample Name: | hwc-9-74-3 ojh 99552142200040 | Acquired By: | System |
| Sample Type: | | Date Acquired: | 2018/3/23 14:56:47 CST |
| Vial: | 1:A,1 | Acq. Method Set: | chiral_isocratic |
| Injection: | 1 | Date Processed: | 2018/5/28 9:14:03 CST |
| Injection Volume: | 2.00 ul | Processing Method | 1 |
| Run Time: | 10.0 Minutes | Channel Name: | PDA Ch1 214 nm@1.2 nm |
| Sample Set Name | 20180323 | Proc. Chnl. Descr: | PDA Ch1 214 nm@1.2 nm |

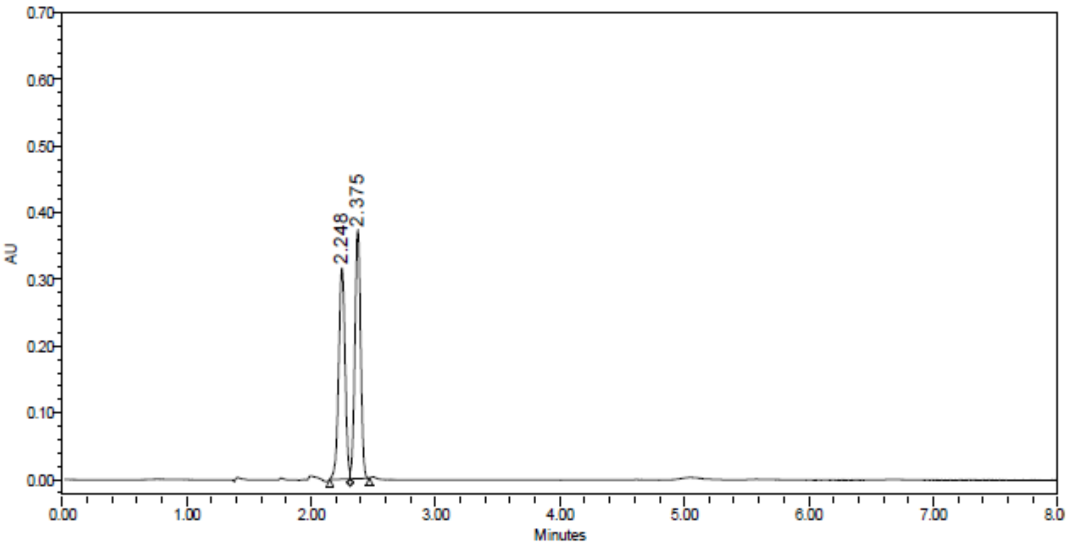


Peak Results

| | RT | Area | Height | % Area |
|---|---|---|---|---|
| 1 | 2.248 | 1129521 | 316678 | 48.51 |
| 2 | 2.375 | 1198870 | 373289 | 51.49 |

**Supplementary Figure 35**. HPLC chromatogram for compound **3m**, racemic

SAMPLE INFORMATION

| | | | |
|---|---|---|---|
| Sample Name: | hwc-9-56-3 ojh 99552142200040 | Acquired By: | System |
| Sample Type: | | Date Acquired: | 2018/3/23 15:07:43 CST |
| Vial: | 1:A,2 | Acq. Method Set: | chiral_isocratic |
| Injection: | 1 | Date Processed: | 2018/5/28 9:14:47 CST |
| Injection Volume: | 2.00 ul | Processing Method | 1 |
| Run Time: | 10.0 Minutes | Channel Name: | PDA Ch1 214 nm@1.2 nm |
| Sample Set Name | 20180323 | Proc. Chnl. Descr: | PDA Ch1 214 nm@1.2 nm |

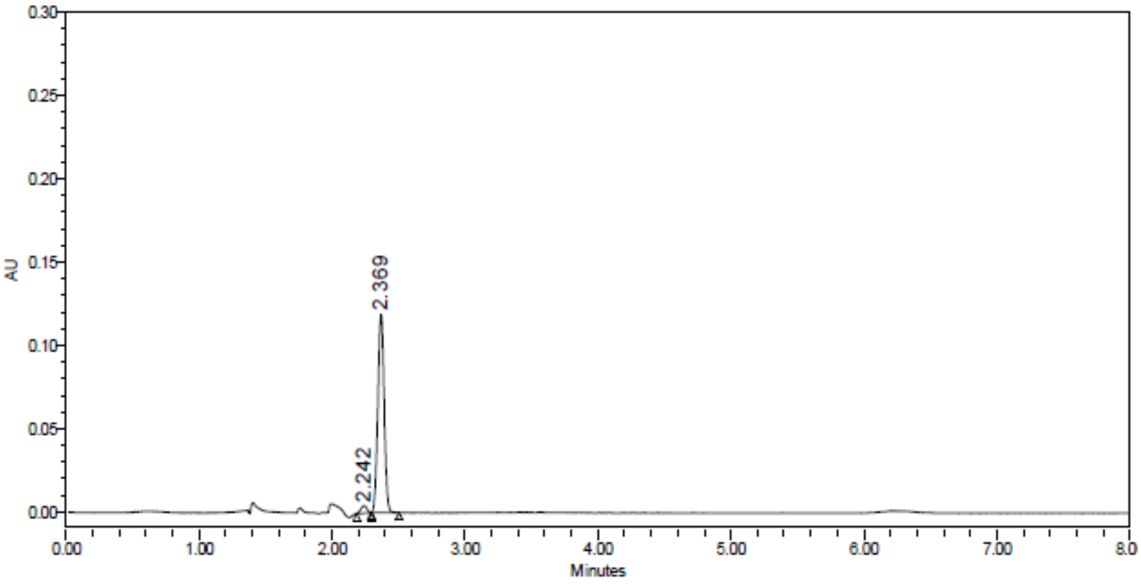


Peak Results

| | RT | Area | Height | % Area |
|---|---|---|---|---|
| 1 | 2.242 | 13804 | 4549 | 3.42 |
| 2 | 2.369 | 390072 | 118907 | 96.58 |

**Supplementary Figure 36**. HPLC chromatogram for compound **3m**

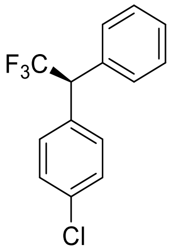

Compound **3n**. HPLC (OJ-H, 0.46 × 25 cm, 5 μm, hexane/isopropanol = 98/2 (v/v %), flow 0.7 mL/min, UV detection at 214 nm), retention time = 10.74 min (minor) and 14.58 min (major). $[\alpha]_D^{25}$ = 4.0199 (c = 0.1050, $CHCl_3$, 96:4 e.r).

**512 HWC-8-33-3B OJH 982 214 0.7**

| | | | |
|---|---|---|---|
| *Sample Name:* | **HWC-8-33-3B OJH 982 214 0.7** | *Injection Volume:* | **3.0** |
| *Vial Number:* | **GA3** | *Channel:* | **UV_VIS_2** |
| *Sample Type:* | **unknown** | *Wavelength:* | **214.0** |
| *Control Program:* | **test-dad2** | *Bandwidth:* | **4** |
| *Quantif. Method:* | **20170608** | *Dilution Factor:* | **1.0000** |
| *Recording Time:* | **2018-1-24 19:10** | *Sample Weight:* | **1.0000** |
| *Run Time (min):* | **25.00** | *Sample Amount:* | **1.0000** |

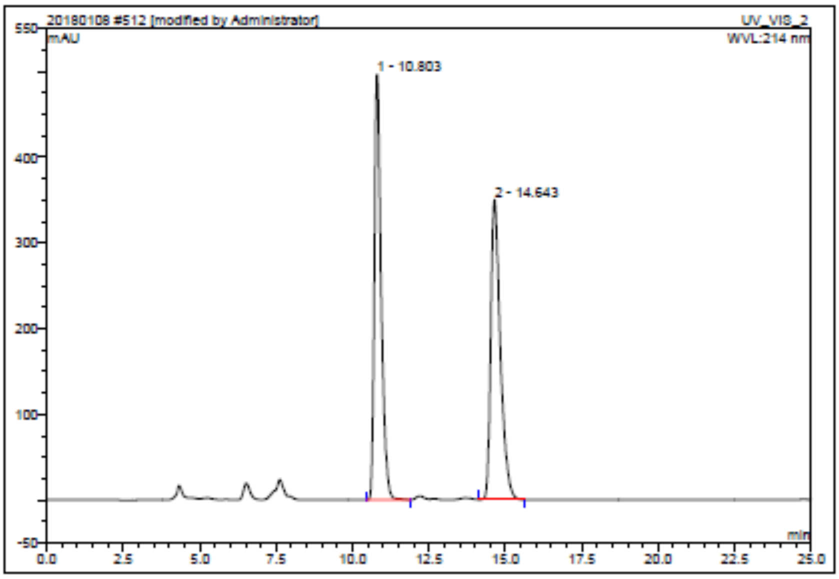


| No. | Ret.Time min | Peak Name | Height mAU | Area mAU*min | Rel.Area % | Amount | Type |
|---|---|---|---|---|---|---|---|
| 1 | 10.80 | n.a. | 496.744 | 127.826 | 50.19 | n.a. | BMB |
| 2 | 14.64 | n.a. | 350.150 | 126.873 | 49.81 | n.a. | BMB* |
| Total: | | | 846.894 | 254.699 | 100.00 | 0.000 | |

**Supplementary Figure 37**. HPLC chromatogram for compound **3n**, racemic

**513 HWC-9-2-3B OJH 982 214 0.7**

| | | | |
|---|---|---|---|
| *Sample Name:* | HWC-9-2-3B OJH 982 214 0.7 | *Injection Volume:* | 3.0 |
| *Vial Number:* | GA2 | *Channel:* | UV_VIS_2 |
| *Sample Type:* | unknown | *Wavelength:* | 214.0 |
| *Control Program:* | test-dad2 | *Bandwidth:* | 4 |
| *Quantif. Method:* | 20170608 | *Dilution Factor:* | 1.0000 |
| *Recording Time:* | 2018-1-24 19:36 | *Sample Weight:* | 1.0000 |
| *Run Time (min):* | 25.00 | *Sample Amount:* | 1.0000 |

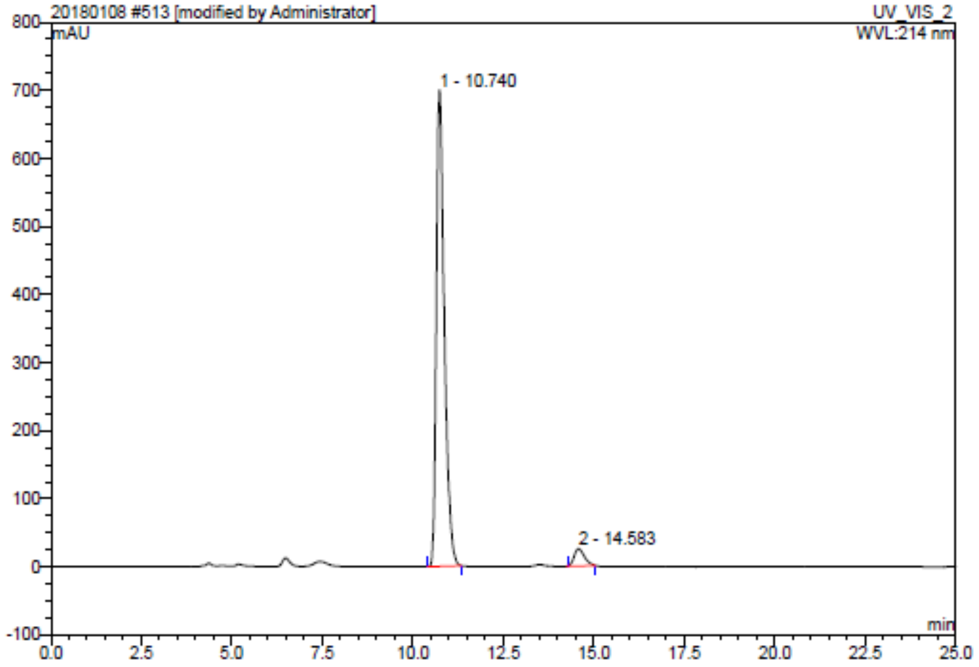


| No. | Ret.Time min | Peak Name | Height mAU | Area mAU*min | Rel.Area % | Amount | Type |
|---|---|---|---|---|---|---|---|
| 1 | 10.74 | n.a. | 700.080 | 179.862 | 95.57 | n.a. | BMB* |
| 2 | 14.58 | n.a. | 25.397 | 8.341 | 4.43 | n.a. | BMB* |
| Total: | | | 725.477 | 188.203 | 100.00 | 0.000 | |

**Supplementary Figure 38**. HPLC chromatogram for compound **3n**

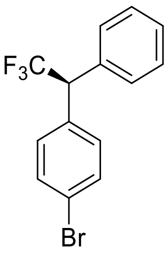


Compound **3o**. HPLC (IG, 0.46 × 25 cm, 5 μm, hexane/isopropanol = 95/5 (v/v %), flow 0.7 mL/min, UV detection at 214 nm), retention time = 5.87 min (minor) and 6.13 min (major). $[\alpha]_D^{25}$ = 8.5027 (c = 0.2000, $CHCl_3$, 95:5 e.r.).

**984 HWC-8-102-3F IG 991 214 0.7**

| | | | |
|---|---|---|---|
| *Sample Name:* | HWC-8-102-3F IG 991 214 0.7 | *Injection Volume:* | 1.0 |
| *Vial Number:* | RE5 | *Channel:* | UV_VIS_2 |
| *Sample Type:* | unknown | *Wavelength:* | 214.0 |
| *Control Program:* | test-dad6 | *Bandwidth:* | 4 |
| *Quantif. Method:* | 20170608 | *Dilution Factor:* | 1.0000 |
| *Recording Time:* | 2018-3-8 11:25 | *Sample Weight:* | 1.0000 |
| *Run Time (min):* | 9.95 | *Sample Amount:* | 1.0000 |

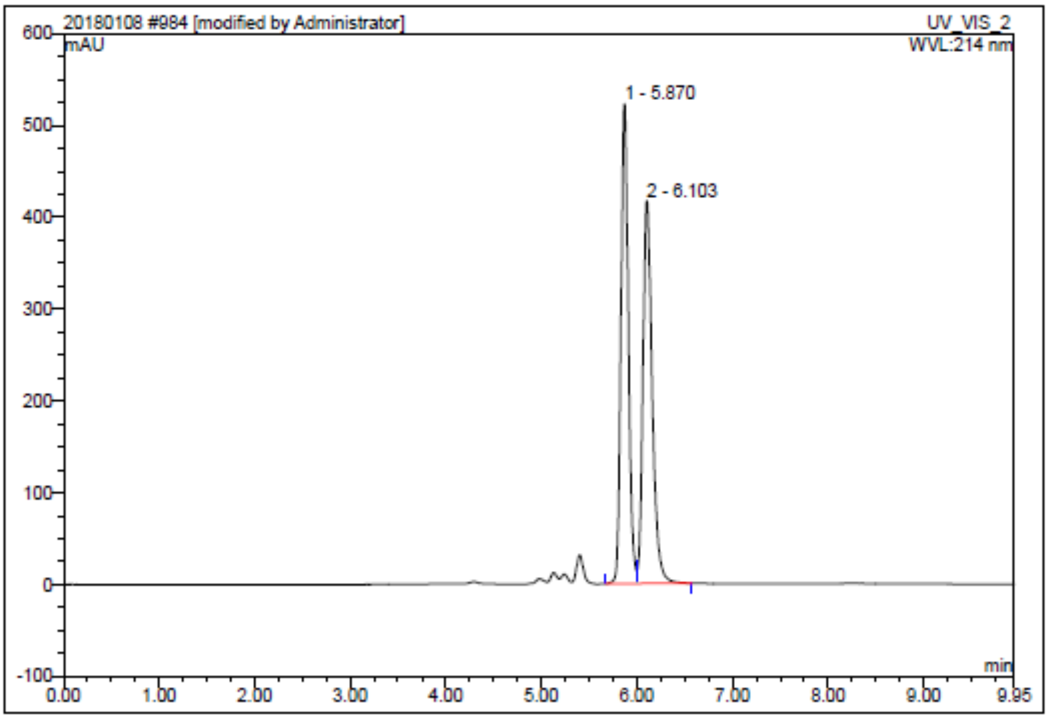


| No. | Ret.Time min | Peak Name | Height mAU | Area mAU*min | Rel.Area % | Amount | Type |
|---|---|---|---|---|---|---|---|
| 1 | 5.87 | n.a. | 522.677 | 47.093 | 50.00 | n.a. | BM |
| 2 | 6.10 | n.a. | 416.655 | 47.095 | 50.00 | n.a. | MB |
| Total: | | | 939.333 | 94.188 | 100.00 | 0.000 | |

**Supplementary Figure 39**. HPLC chromatogram for compound **3o**, racemic

**985 HWC-9-46-3 IG 991 214 0.7**

| | | | |
|---|---|---|---|
| *Sample Name:* | HWC-9-46-3 IG 991 214 0.7 | *Injection Volume:* | 1.0 |
| *Vial Number:* | RD5 | *Channel:* | UV_VIS_2 |
| *Sample Type:* | unknown | *Wavelength:* | 214.0 |
| *Control Program:* | test-dad6 | *Bandwidth:* | 4 |
| *Quantif. Method:* | 20170608 | *Dilution Factor:* | 1.0000 |
| *Recording Time:* | 2018-3-8 11:36 | *Sample Weight:* | 1.0000 |
| *Run Time (min):* | 16.60 | *Sample Amount:* | 1.0000 |

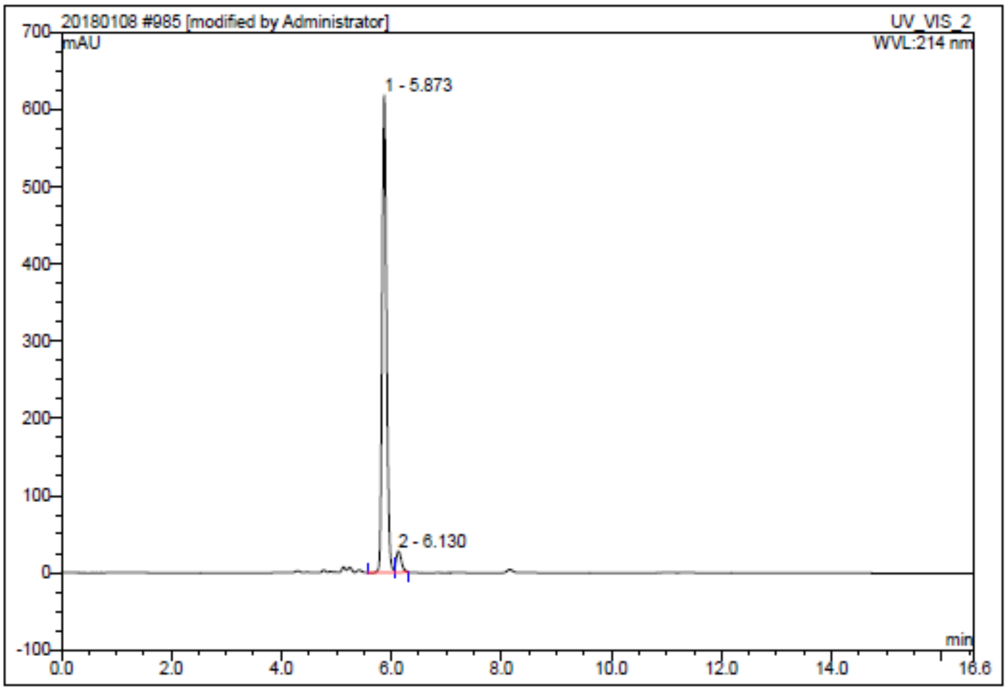


| No. | Ret.Time min | Peak Name | Height mAU | Area mAU*min | Rel.Area % | Amount | Type |
|---|---|---|---|---|---|---|---|
| 1 | 5.87 | n.a. | 618.078 | 56.171 | 94.85 | n.a. | BM * |
| 2 | 6.13 | n.a. | 27.625 | 3.048 | 5.15 | n.a. | MB* |
| Total: | | | 645.703 | 59.219 | 100.00 | 0.000 | |

**Supplementary Figure 40**. HPLC chromatogram for compound **3o**

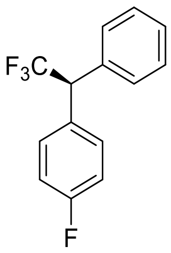


Compound **3p**. HPLC (AD-H, 0.46 × 25 cm, 5 μm, hexane = 100%, flow 0.7 mL/min, UV detection at 214 nm), retention time = 9.53 min (minor) and 10.89 min (major). $[\alpha]_D^{25}$ = -3.0174 (c = 0.1500, $CHCl_3$, 96:4 e.r.).

**455 HWC-8-34-3A ADH 1 214 0.7**

| | | | |
|---|---|---|---|
| *Sample Name:* | **HWC-8-34-3A ADH 1 214 0.7** | *Injection Volume:* | **1.0** |
| *Vial Number:* | **GC5** | *Channel:* | **UV_VIS_2** |
| *Sample Type:* | **unknown** | *Wavelength:* | **214.0** |
| *Control Program:* | **test-dad2** | *Bandwidth:* | **4** |
| *Quantif. Method:* | **20170608** | *Dilution Factor:* | **1.0000** |
| *Recording Time:* | **2018-1-22 12:24** | *Sample Weight:* | **1.0000** |
| *Run Time (min):* | **20.00** | *Sample Amount:* | **1.0000** |

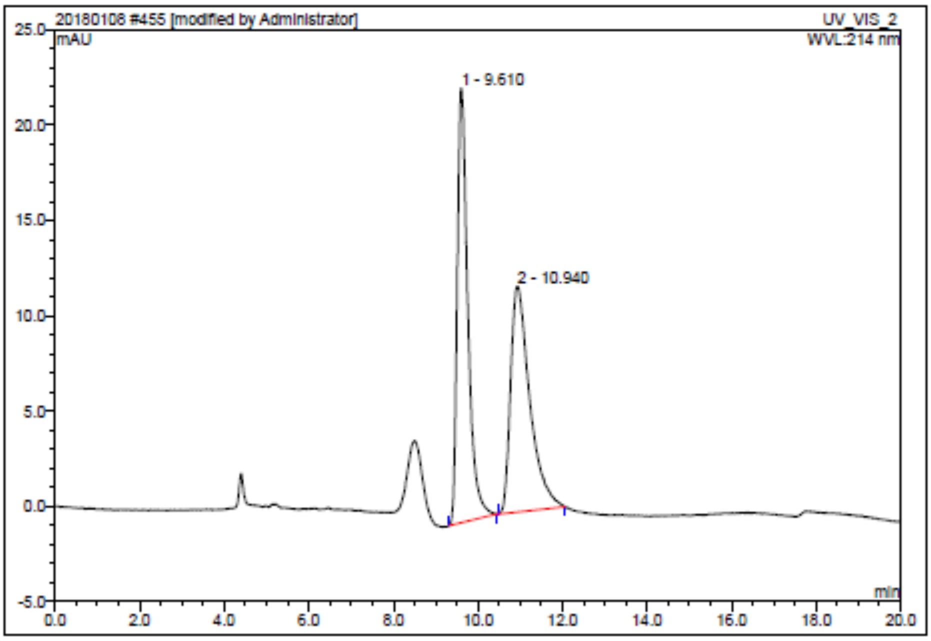


| No. | Ret.Time min | Peak Name | Height mAU | Area mAU*min | Rel.Area % | Amount | Type |
|---|---|---|---|---|---|---|---|
| 1 | 9.61 | n.a. | 22.819 | 6.638 | 51.61 | n.a. | BMB |
| 2 | 10.94 | n.a. | 11.844 | 6.224 | 48.39 | n.a. | BMB |
| **Total:** | | | 34.663 | 12.862 | 100.00 | 0.000 | |

**Supplementary Figure 41**. HPLC chromatogram for compound **3p**, racemic

**456 HWC-8-104-3E ADH 1 214 0.7**

| | | | |
|---|---|---|---|
| *Sample Name:* | HWC-8-104-3E ADH 1 214 0.7 | *Injection Volume:* | 1.0 |
| *Vial Number:* | GC6 | *Channel:* | UV_VIS_2 |
| *Sample Type:* | unknown | *Wavelength:* | 214.0 |
| *Control Program:* | test-dad2 | *Bandwidth:* | 4 |
| *Quantif. Method:* | 20170608 | *Dilution Factor:* | 1.0000 |
| *Recording Time:* | 2018-1-22 12:45 | *Sample Weight:* | 1.0000 |
| *Run Time (min):* | 20.00 | *Sample Amount:* | 1.0000 |

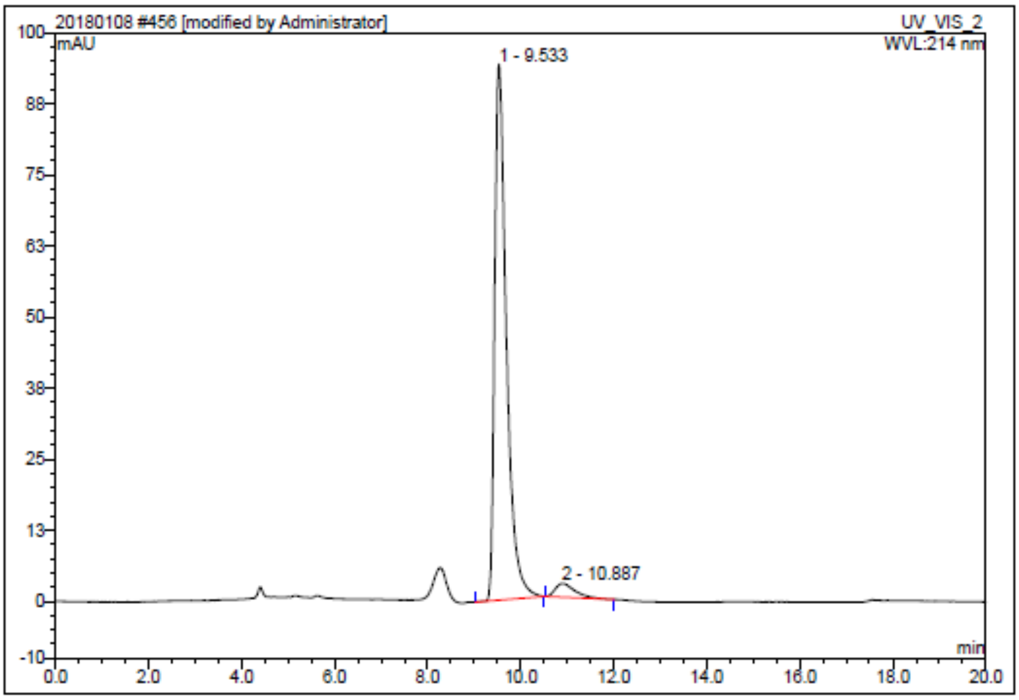


| No. | Ret.Time min | Peak Name | Height mAU | Area mAU*min | Rel.Area % | Amount | Type |
|---|---|---|---|---|---|---|---|
| 1 | 9.53 | n.a. | 94.225 | 27.543 | 95.83 | n.a. | BMB* |
| 2 | 10.89 | n.a. | 2.395 | 1.197 | 4.17 | n.a. | BMB* |
| Total: | | | 96.620 | 28.741 | 100.00 | 0.000 | |

**Supplementary Figure 42**. HPLC chromatogram for compound **3p**

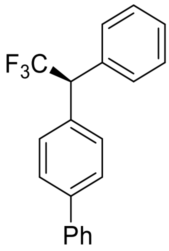

Compound **3q**. HPLC (IG, 0.46 × 25 cm, 5 μm, hexane/isopropanol = 99/1 (v/v %), flow 0.7 mL/min, UV detection at 214 nm), retention time = 7.82 min (minor) and 8.78 min (major). $[\alpha]_D^{25}$ = 26.1836 (c = 0.1200, $CHCl_3$, 95:5 e.r.).

**5352 HWC-8-41-3A+- IG 991 214 0.7**

| | | | |
|---|---|---|---|
| *Sample Name:* | HWC-8-41-3A+- IG 991 214 0.7 | *Injection Volume:* | 6.0 |
| *Vial Number:* | RC5 | *Channel:* | UV_VIS_1 |
| *Sample Type:* | unknown | *Wavelength:* | 214 |
| *Control Program:* | 201701-6 | *Bandwidth:* | n.a. |
| *Quantif. Method:* | 201701 | *Dilution Factor:* | 1.0000 |
| *Recording Time:* | 2018/1/22 14:02 | *Sample Weight:* | 1.0000 |
| *Run Time (min):* | 15.25 | *Sample Amount:* | 1.0000 |

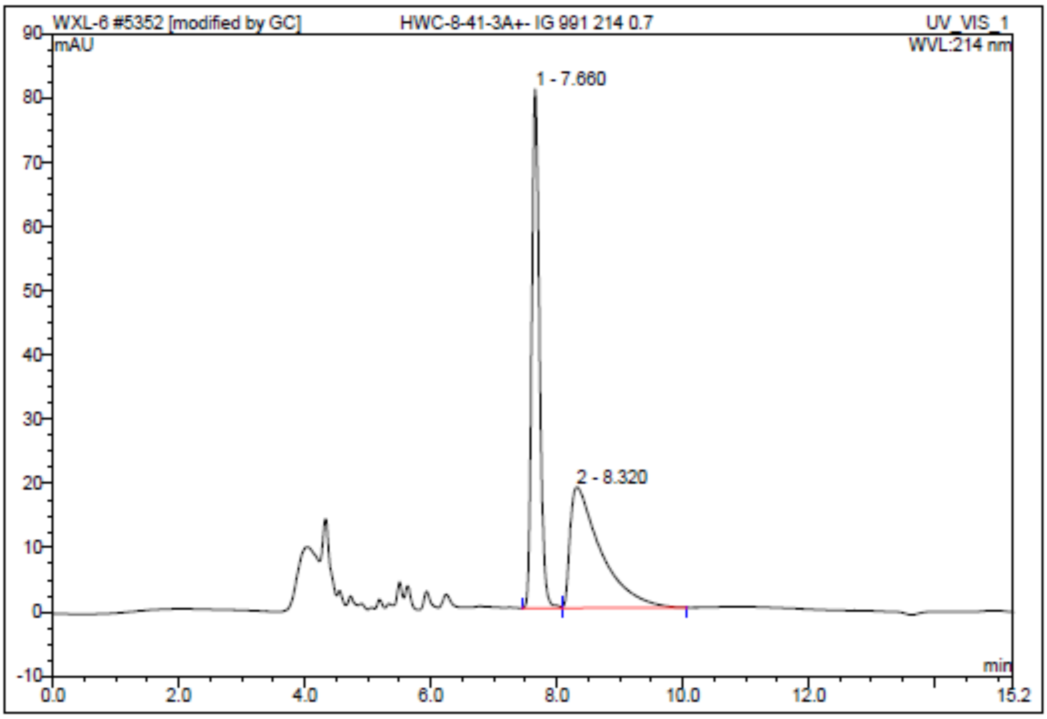


| No. | Ret.Time min | Peak Name | Height mAU | Area mAU*min | Rel.Area % | Amount | Type |
|---|---|---|---|---|---|---|---|
| 1 | 7.66 | n.a. | 80.747 | 11.450 | 51.76 | n.a. | BM |
| 2 | 8.32 | n.a. | 18.807 | 10.670 | 48.24 | n.a. | MB |
| Total: | | | 99.554 | 22.120 | 100.00 | 0.000 | |

**Supplementary Figure 43**. HPLC chromatogram for compound **3q**, racemic

## 5353 HWC-8-104-3G IG 991 214 0.7

| | | | |
|---|---|---|---|
| *Sample Name:* | **HWC-8-104-3G IG 991 214 0.7** | *Injection Volume:* | **1.0** |
| *Vial Number:* | **RC6** | *Channel:* | **UV_VIS_1** |
| *Sample Type:* | **unknown** | *Wavelength:* | **214** |
| *Control Program:* | **201701-6** | *Bandwidth:* | **n.a.** |
| *Quantif. Method:* | **201701** | *Dilution Factor:* | **1.0000** |
| *Recording Time:* | **2018/1/22 13:38** | *Sample Weight:* | **1.0000** |
| *Run Time (min):* | **19.01** | *Sample Amount:* | **1.0000** |

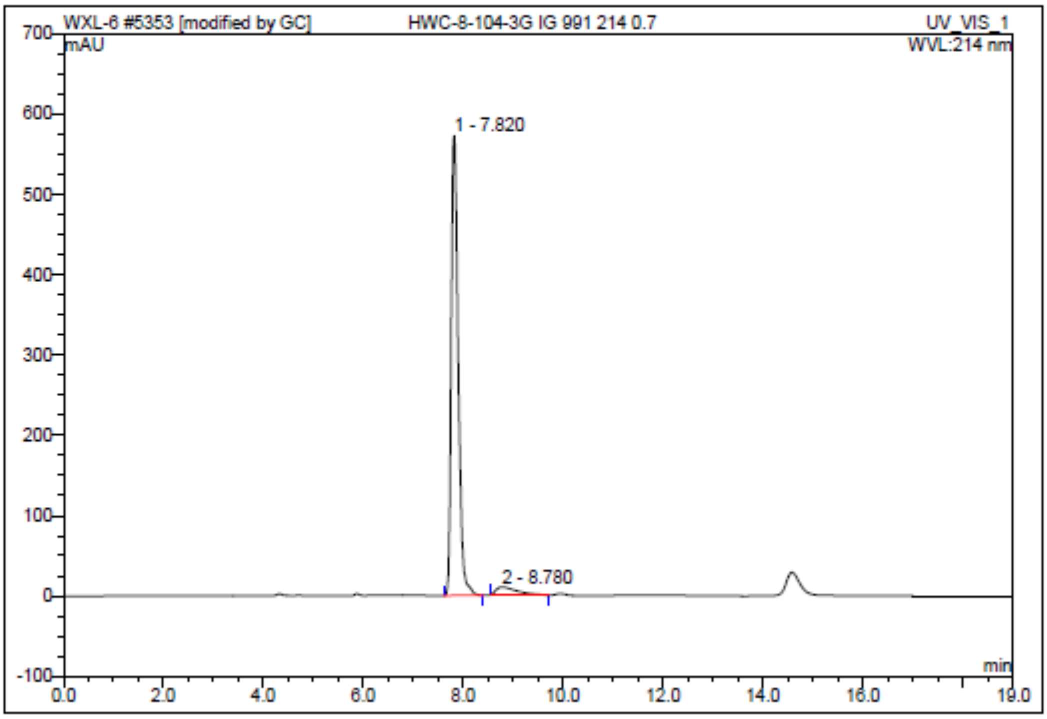


| No. | Ret.Time min | Peak Name | Height mAU | Area mAU*min | Rel.Area % | Amount | Type |
|---|---|---|---|---|---|---|---|
| 1 | 7.82 | n.a. | 572.103 | 92.601 | 95.11 | n.a. | BMB* |
| 2 | 8.78 | n.a. | 9.962 | 4.762 | 4.89 | n.a. | BMB* |
| **Total:** | | | 582.065 | 97.362 | 100.00 | 0.000 | |

**Supplementary Figure 44**. HPLC chromatogram for compound **3q**

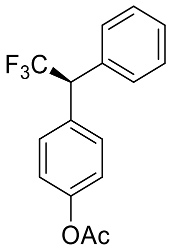


Compound **3r**. HPLC (IG, 0.46 × 25 cm, 5 µm, hexane/isopropanol = 99/1 (v/v %), flow 0.7 mL/min, UV detection at 214 nm), retention time = 12.49 min (minor) and 13.57 min (major). $[\alpha]_D^{25}$ = -10.1055 (c = 0.095, $CHCl_3$, 94:6 e.r.).

Operator:GC Timebase:U3000 Sequence:WXL-6 Pag[illegible]
2018/5/26 11:06

**6326 HWC-9-31-3B IG 991 214 0.7**

| | | | |
|---|---|---|---|
| *Sample Name:* | **HWC-9-31-3B IG 991 214 0.7** | *Injection Volume:* | **2.0** |
| *Vial Number:* | **GB6** | *Channel:* | **UV_VIS_1** |
| *Sample Type:* | **unknown** | *Wavelength:* | **214** |
| *Control Program:* | **201701-4** | *Bandwidth:* | **n.a.** |
| *Quantif. Method:* | **201701** | *Dilution Factor:* | **1.0000** |
| *Recording Time:* | **2018/4/12 9:47** | *Sample Weight:* | **1.0000** |
| *Run Time (min):* | **20.01** | *Sample Amount:* | **1.0000** |

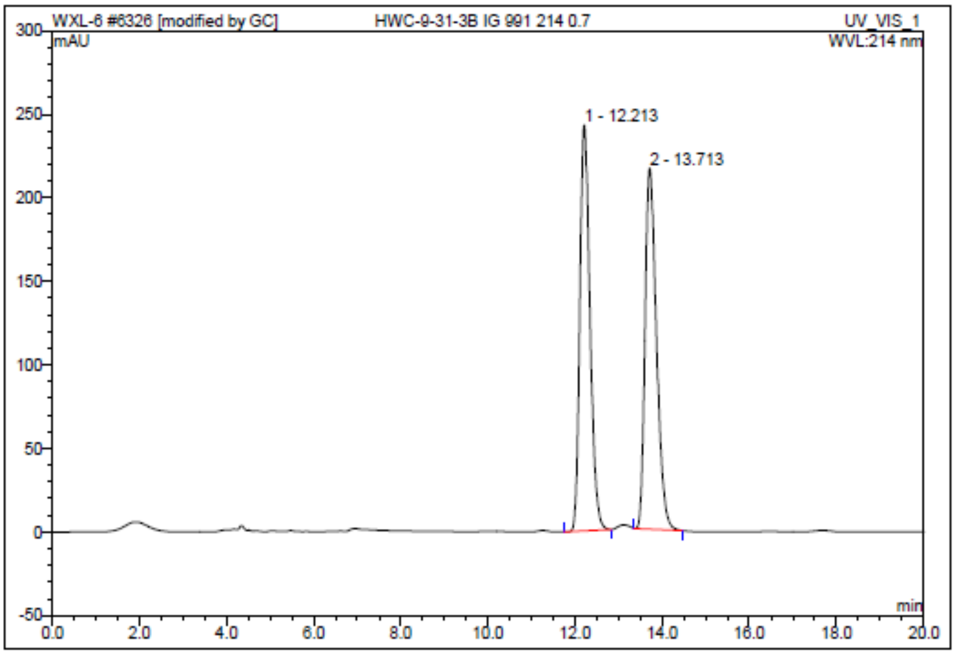


| No. | Ret.Time min | Peak Name | Height mAU | Area mAU*min | Rel.Area % | Amount | Type |
|---|---|---|---|---|---|---|---|
| 1 | 12.21 | n.a. | 242.791 | 65.456 | 49.93 | n.a. | BMB* |
| 2 | 13.71 | n.a. | 216.050 | 65.648 | 50.07 | n.a. | BMB* |
| **Total:** | | | 458.841 | 131.103 | 100.00 | 0.000 | |

**Supplementary Figure 45**. HPLC chromatogram for compound **3r**, racemic

**6328 HWC-9-31-3A IG 991 214 0.7**

| | | | |
|---|---|---|---|
| *Sample Name:* | **HWC-9-31-3A IG 991 214 0.7** | *Injection Volume:* | **2.0** |
| *Vial Number:* | **GD6** | *Channel:* | **UV_VIS_1** |
| *Sample Type:* | **unknown** | *Wavelength:* | **214** |
| *Control Program:* | **201701-4** | *Bandwidth:* | **n.a.** |
| *Quantif. Method:* | **201701** | *Dilution Factor:* | **1.0000** |
| *Recording Time:* | **2018/4/12 9:15** | *Sample Weight:* | **1.0000** |
| *Run Time (min):* | **20.01** | *Sample Amount:* | **1.0000** |

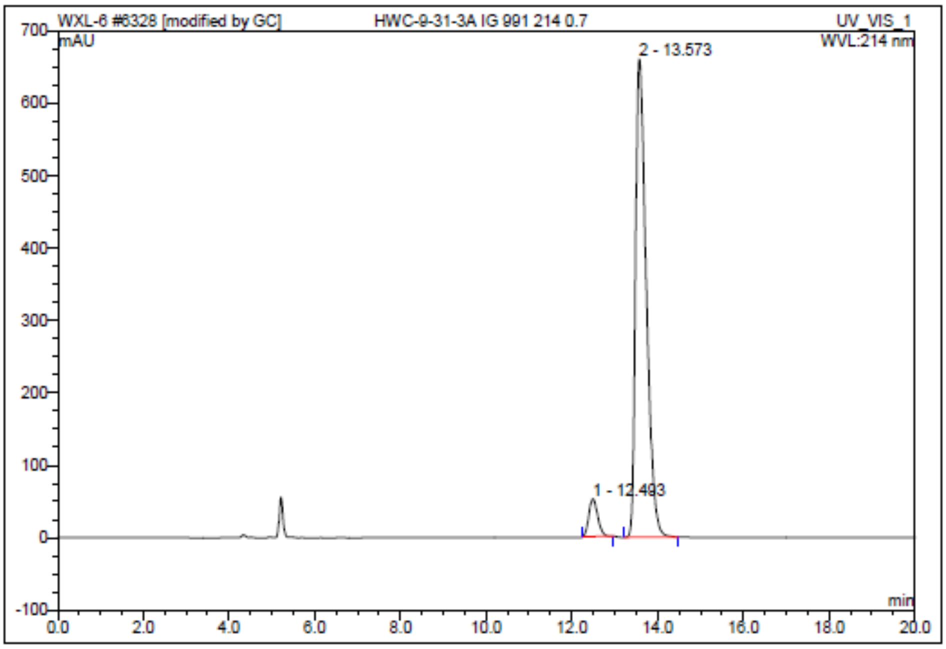


| No. | Ret.Time min | Peak Name | Height mAU | Area mAU*min | Rel.Area % | Amount | Type |
|---|---|---|---|---|---|---|---|
| 1 | 12.49 | n.a. | 51.798 | 12.401 | 6.09 | n.a. | BMB* |
| 2 | 13.57 | n.a. | 659.668 | 191.142 | 93.91 | n.a. | BMB* |
| Total: | | | 711.466 | 203.543 | 100.00 | 0.000 | |

**Supplementary Figure 46**. HPLC chromatogram for compound **3r**

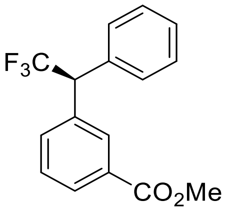


Compound **3s**. HPLC (IG, 0.46 × 25 cm, 5 μm, hexane/isopropanol = 99/1 (v/v %), flow 0.7 mL/min, UV detection at 214 nm), retention time = 13.38 min (minor) and 14.15 min (major). $[\alpha]_D^{25}$ = 19.1278 (c = 0.1000, $CHCl_3$, 96:4 e.r.).

**5349 HWC-8-102-3B+- IG 991 214 0.7**

| | | | |
|---|---|---|---|
| *Sample Name:* | **HWC-8-102-3B+- IG 991 214 0.7** | *Injection Volume:* | **1.0** |
| *Vial Number:* | **RC3** | *Channel:* | **UV_VIS_1** |
| *Sample Type:* | **unknown** | *Wavelength:* | **214** |
| *Control Program:* | **201701-6** | *Bandwidth:* | **n.a.** |
| *Quantif. Method:* | **201701** | *Dilution Factor:* | **1.0000** |
| *Recording Time:* | **2018/1/22 12:37** | *Sample Weight:* | **1.0000** |
| *Run Time (min):* | **19.01** | *Sample Amount:* | **1.0000** |

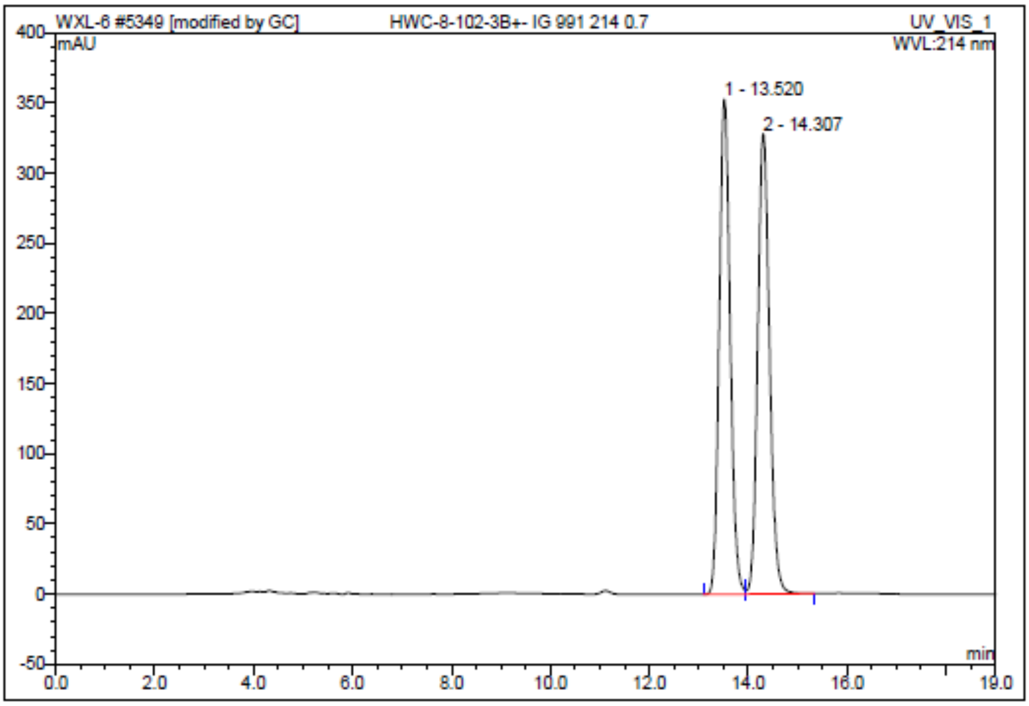


| No. | Ret.Time min | Peak Name | Height mAU | Area mAU*min | Rel.Area % | Amount | Type |
|---|---|---|---|---|---|---|---|
| 1 | 13.52 | n.a. | 352.903 | 90.716 | 49.90 | n.a. | BM |
| 2 | 14.31 | n.a. | 327.847 | 91.064 | 50.10 | n.a. | MB |
| **Total:** | | | 680.750 | 181.780 | 100.00 | 0.000 | |

**Supplementary Figure 47**. HPLC chromatogram for compound **3s**, racemic

## 5350 HWC-8-104-3B IG 991 214 0.7

| | | | |
|---|---|---|---|
| *Sample Name:* | **HWC-8-104-3B IG 991 214 0.7** | *Injection Volume:* | **1.0** |
| *Vial Number:* | **RC4** | *Channel:* | **UV_VIS_1** |
| *Sample Type:* | **unknown** | *Wavelength:* | **214** |
| *Control Program:* | **201701-6** | *Bandwidth:* | **n.a.** |
| *Quantif. Method:* | **201701** | *Dilution Factor:* | **1.0000** |
| *Recording Time:* | **2018/1/22 12:57** | *Sample Weight:* | **1.0000** |
| *Run Time (min):* | **19.01** | *Sample Amount:* | **1.0000** |

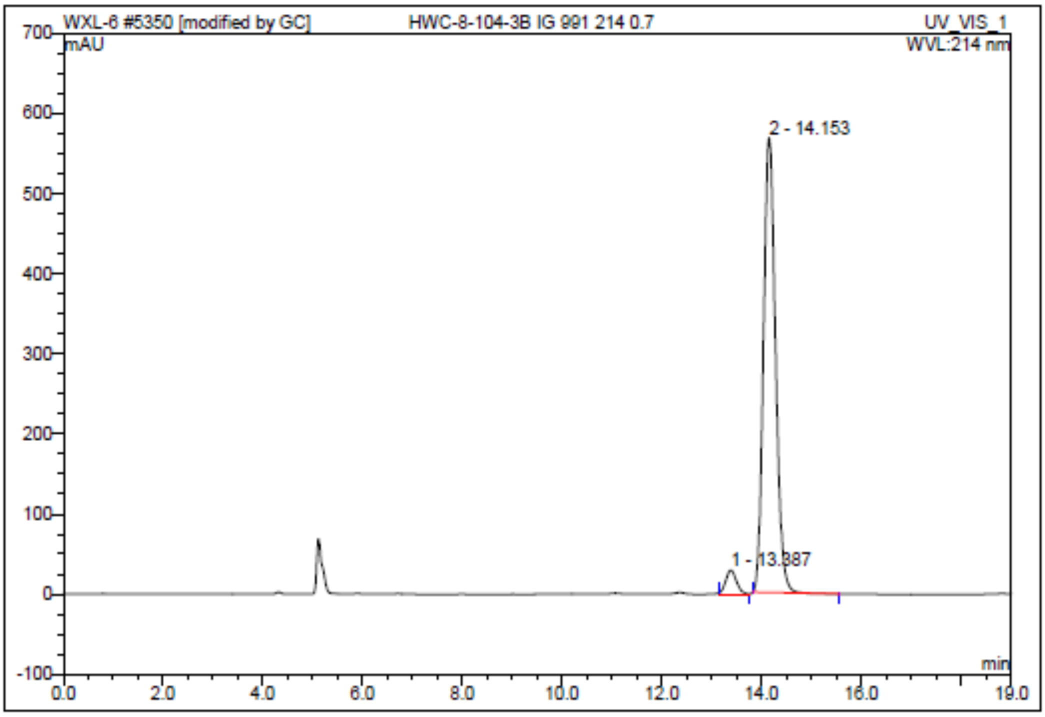


| No. | Ret.Time min | Peak Name | Height mAU | Area mAU*min | Rel.Area % | Amount | Type |
|---|---|---|---|---|---|---|---|
| 1 | 13.39 | n.a. | 29.400 | 7.284 | 4.41 | n.a. | MB* |
| 2 | 14.15 | n.a. | 567.198 | 158.016 | 95.59 | n.a. | BMB* |
| Total: | | | 596.598 | 165.300 | 100.00 | 0.000 | |

**Supplementary Figure 48**. HPLC chromatogram for compound **3s**

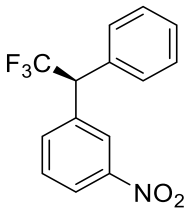


Compound **3t**. HPLC (IG, 0.46 × 25 cm, 5 µm, hexane/isopropanol = 95/5 (v/v %), flow 0.7 mL/min, UV detection at 214 nm), retention time = 13.14 min (minor) and 17.64 min (major). $[\alpha]_D^{25}$ = 17.1305 (c = 0.1300, $CHCl_3$, 96:4 e.r.).

**5951 HWC-8-49-3A+- ODH 991 214 0.7**

| | | | |
|---|---|---|---|
| *Sample Name:* | HWC-8-49-3A+- ODH 991 214 0.7 | *Injection Volume:* | 1.0 |
| *Vial Number:* | RC7 | *Channel:* | UV_VIS_1 |
| *Sample Type:* | unknown | *Wavelength:* | 214 |
| *Control Program:* | 201701-3 | *Bandwidth:* | n.a. |
| *Quantif. Method:* | 201701 | *Dilution Factor:* | 1.0000 |
| *Recording Time:* | 2018/3/12 18:52 | *Sample Weight:* | 1.0000 |
| *Run Time (min):* | 61.94 | *Sample Amount:* | 1.0000 |

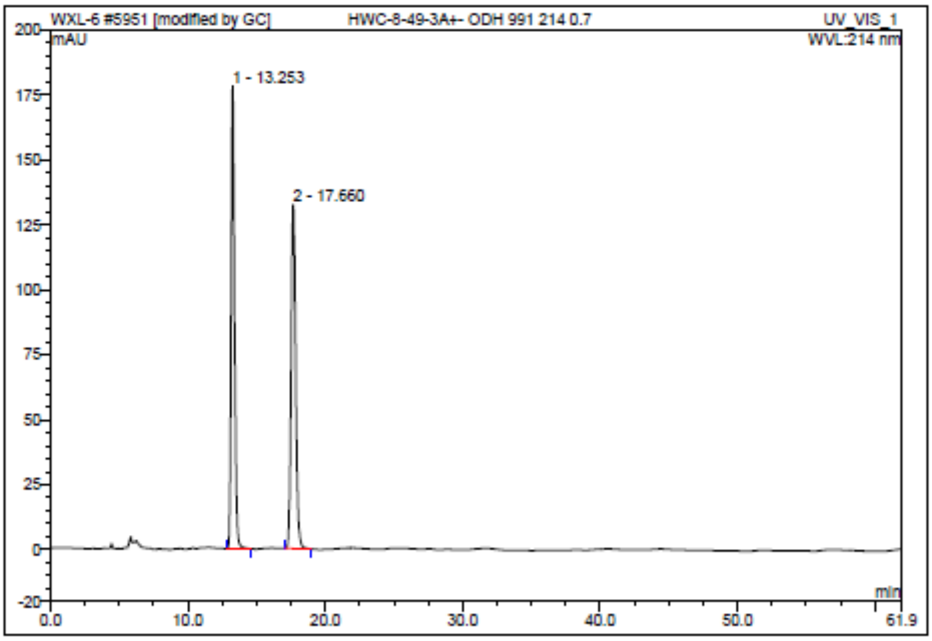


| No. | Ret.Time min | Peak Name | Height mAU | Area mAU*min | Rel.Area % | Amount | Type |
|---|---|---|---|---|---|---|---|
| 1 | 13.25 | n.a. | 177.966 | 51.104 | 49.95 | n.a. | BMB |
| 2 | 17.66 | n.a. | 132.370 | 51.208 | 50.05 | n.a. | BMB |
| Total: | | | 310.337 | 102.312 | 100.00 | 0.000 | |

**Supplementary Figure 49**. HPLC chromatogram for compound **3t**, racemic

‘

# 5952 HWC-9-49-3A ODH 991 214 0.7

| | | | |
|---|---|---|---|
| *Sample Name:* | **HWC-9-49-3A ODH 991 214 0.7** | *Injection Volume:* | **1.0** |
| *Vial Number:* | **RC8** | *Channel:* | **UV_VIS_1** |
| *Sample Type:* | **unknown** | *Wavelength:* | **214** |
| *Control Program:* | **201701-3** | *Bandwidth:* | **n.a.** |
| *Quantif. Method:* | **201701** | *Dilution Factor:* | **1.0000** |
| *Recording Time:* | **2018/3/12 17:14** | *Sample Weight:* | **1.0000** |
| *Run Time (min):* | **41.93** | *Sample Amount:* | **1.0000** |

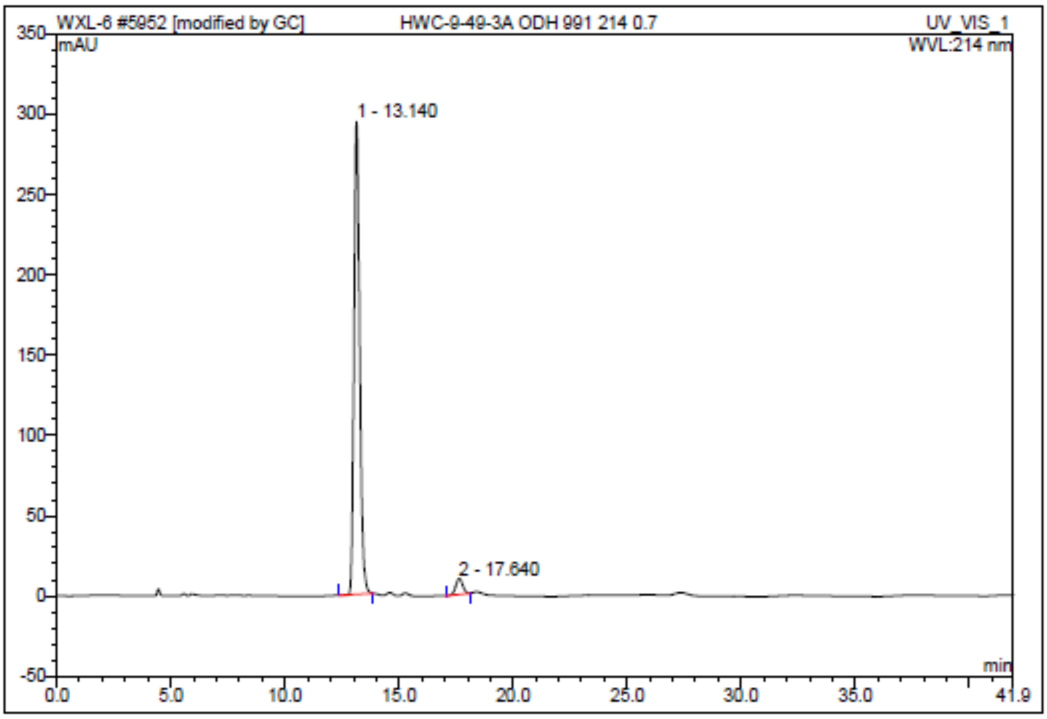


| No. | Ret.Time min | Peak Name | Height mAU | Area mAU*min | Rel.Area % | Amount | Type |
|---|---|---|---|---|---|---|---|
| 1 | 13.14 | n.a. | 293.915 | 84.449 | 96.09 | n.a. | BMB* |
| 2 | 17.64 | n.a. | 9.802 | 3.434 | 3.91 | n.a. | BMB* |
| Total: | | | 303.718 | 87.883 | 100.00 | 0.000 | |

**Supplementary Figure 50**. HPLC chromatogram for compound **3t**

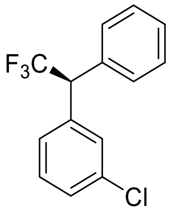


Compound **3u**. HPLC (AD-H, 0.46 × 25 cm, 5 µm, hexane = 100%, flow 0.7 mL/min, UV detection at 214 nm), retention time = 8.69 min (minor) and 9.54 min (major). $[\alpha]_D^{25}$ = 2.3955 (c = 0.1000, $CHCl_3$, 95:5 e.r.).

**952 HWC-8-34-3B ADH 1 214 0.7**

| | | | |
|---|---|---|---|
| *Sample Name:* | HWC-8-34-3B ADH 1 214 0.7 | *Injection Volume:* | 1.0 |
| *Vial Number:* | RE5 | *Channel:* | UV_VIS_2 |
| *Sample Type:* | unknown | *Wavelength:* | 214.0 |
| *Control Program:* | test-dad4 | *Bandwidth:* | 4 |
| *Quantif. Method:* | 20170608 | *Dilution Factor:* | 1.0000 |
| *Recording Time:* | 2018-3-6 15:40 | *Sample Weight:* | 1.0000 |
| *Run Time (min):* | 17.00 | *Sample Amount:* | 1.0000 |

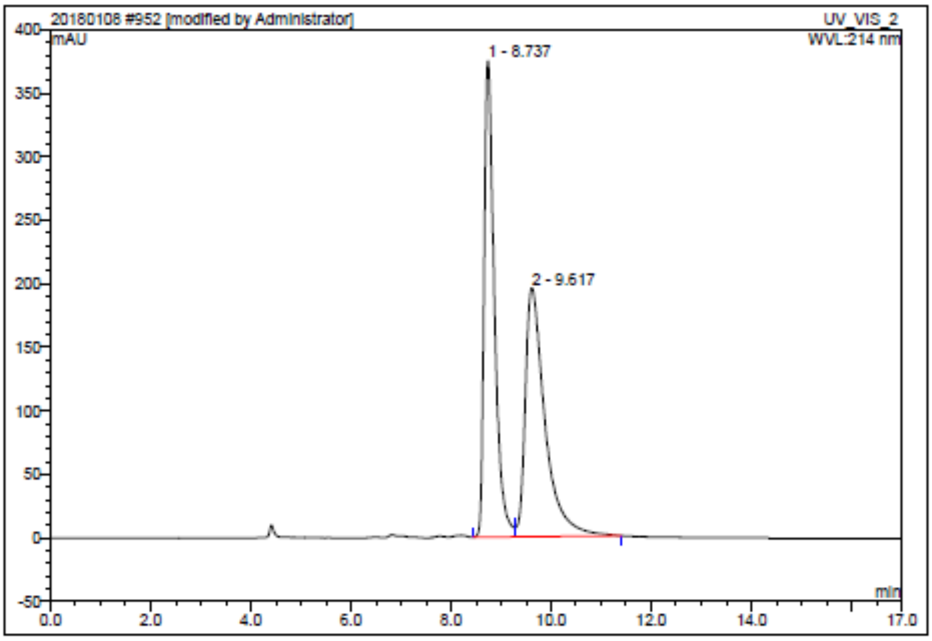


| No. | Ret.Time min | Peak Name | Height mAU | Area mAU*min | Rel.Area % | Amount | Type |
|---|---|---|---|---|---|---|---|
| 1 | 8.74 | n.a. | 374.765 | 89.167 | 49.93 | n.a. | BM |
| 2 | 9.62 | n.a. | 195.595 | 89.424 | 50.07 | n.a. | MB |
| Total: | | | 570.360 | 178.591 | 100.00 | 0.000 | |

**Supplementary Figure 51**. HPLC chromatogram for compound **3u**, racemic

## 994 HWC-9-44-3 ADH 1 214 0.7

| | | | |
|---|---|---|---|
| *Sample Name:* | HWC-9-44-3 ADH 1 214 0.7 | *Injection Volume:* | 2.0 |
| *Vial Number:* | RB7 | *Channel:* | UV_VIS_2 |
| *Sample Type:* | unknown | *Wavelength:* | 214.0 |
| *Control Program:* | test-dad6 | *Bandwidth:* | 4 |
| *Quantif. Method:* | 20170608 | *Dilution Factor:* | 1.0000 |
| *Recording Time:* | 2018-3-8 15:42 | *Sample Weight:* | 1.0000 |
| *Run Time (min):* | 15.00 | *Sample Amount:* | 1.0000 |

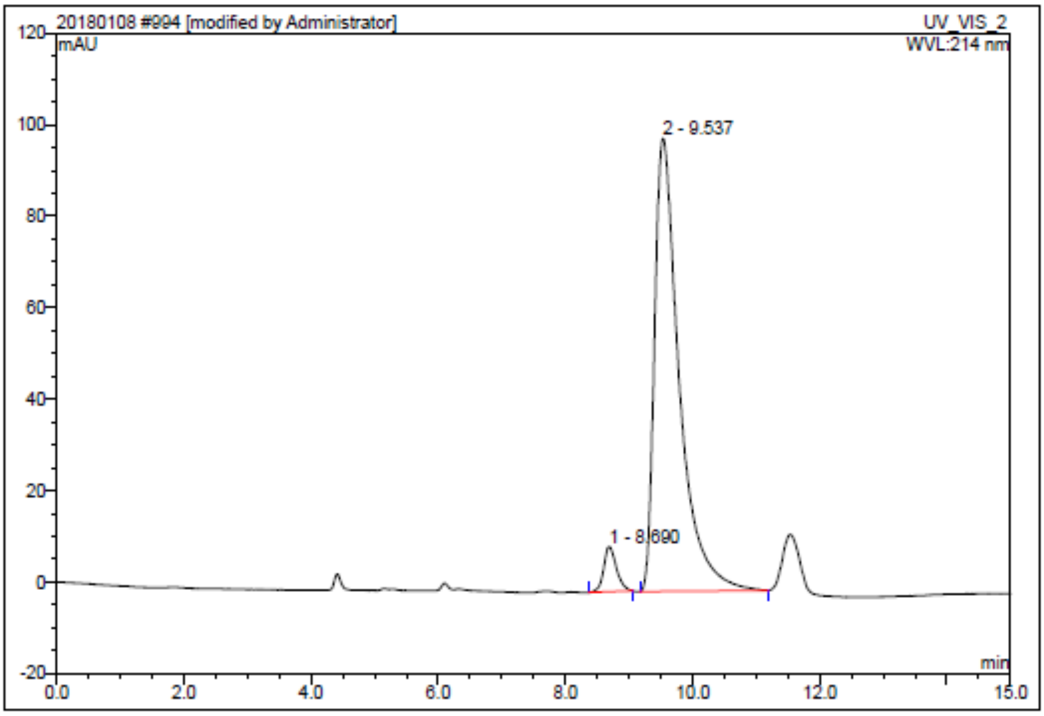


| No. | Ret.Time min | Peak Name | Height mAU | Area mAU*min | Rel.Area % | Amount | Type |
|---|---|---|---|---|---|---|---|
| 1 | 8.69 | n.a. | 9.860 | 2.288 | 4.89 | n.a. | BMB* |
| 2 | 9.54 | n.a. | 98.988 | 44.478 | 95.11 | n.a. | BMB* |
| Total: | | | 108.848 | 46.767 | 100.00 | 0.000 | |

**Supplementary Figure 52**. HPLC chromatogram for compound **3u**

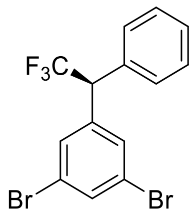


Compound **3v**. HPLC (OJ-H, 0.46 × 25 cm, 5 μm, hexane/isopropanol = 98/2 (v/v %), flow 0.7 mL/min, UV detection at 214 nm), retention time = 8.72 min (minor) and 10.10 min (major). $[\alpha]_D^{25}$ = 27.8440 (c = 0.1050, $CHCl_3$, 96:4 e.r.).

**465 HWC-8-33-3A+- OJH 982 214 0.7**

| | | | |
|---|---|---|---|
| *Sample Name:* | **HWC-8-33-3A+- OJH 982 214 0.7** | *Injection Volume:* | **4.0** |
| *Vial Number:* | **GE4** | *Channel:* | **UV_VIS_2** |
| *Sample Type:* | **unknown** | *Wavelength:* | **214.0** |
| *Control Program:* | **test-dad6** | *Bandwidth:* | **4** |
| *Quantif. Method:* | **20170608** | *Dilution Factor:* | **1.0000** |
| *Recording Time:* | **2018-1-22 17:45** | *Sample Weight:* | **1.0000** |
| *Run Time (min):* | **19.28** | *Sample Amount:* | **1.0000** |

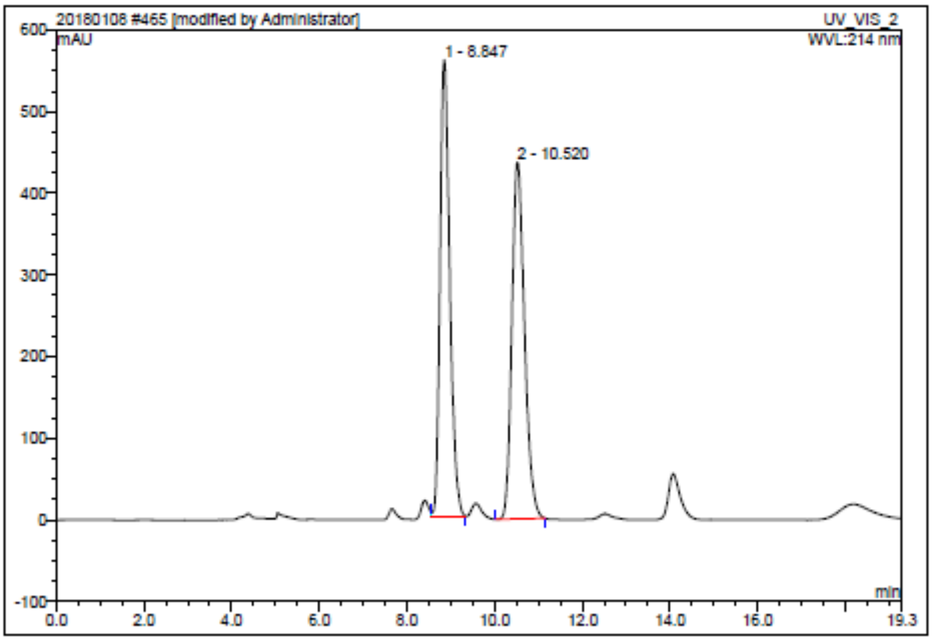


| No. | Ret.Time min | Peak Name | Height mAU | Area mAU*min | Rel.Area % | Amount | Type |
|---|---|---|---|---|---|---|---|
| 1 | 8.85 | n.a. | 558.712 | 143.397 | 49.62 | n.a. | MB* |
| 2 | 10.52 | n.a. | 436.761 | 145.589 | 50.38 | n.a. | BMB* |
| Total: | | | 995.473 | 288.986 | 100.00 | 0.000 | |

**Supplementary Figure 53**. HPLC chromatogram for compound **3v**, racemic

**466 HWC-9-2-3A OJH 982 214 0.7**

| | | | |
|---|---|---|---|
| *Sample Name:* | **HWC-9-2-3A OJH 982 214 0.7** | *Injection Volume:* | **2.0** |
| *Vial Number:* | **GC4** | *Channel:* | **UV_VIS_2** |
| *Sample Type:* | **unknown** | *Wavelength:* | **214.0** |
| *Control Program:* | **test-dad6** | *Bandwidth:* | **4** |
| *Quantif. Method:* | **20170608** | *Dilution Factor:* | **1.0000** |
| *Recording Time:* | **2018-1-23 14:53** | *Sample Weight:* | **1.0000** |
| *Run Time (min):* | **31.65** | *Sample Amount:* | **1.0000** |

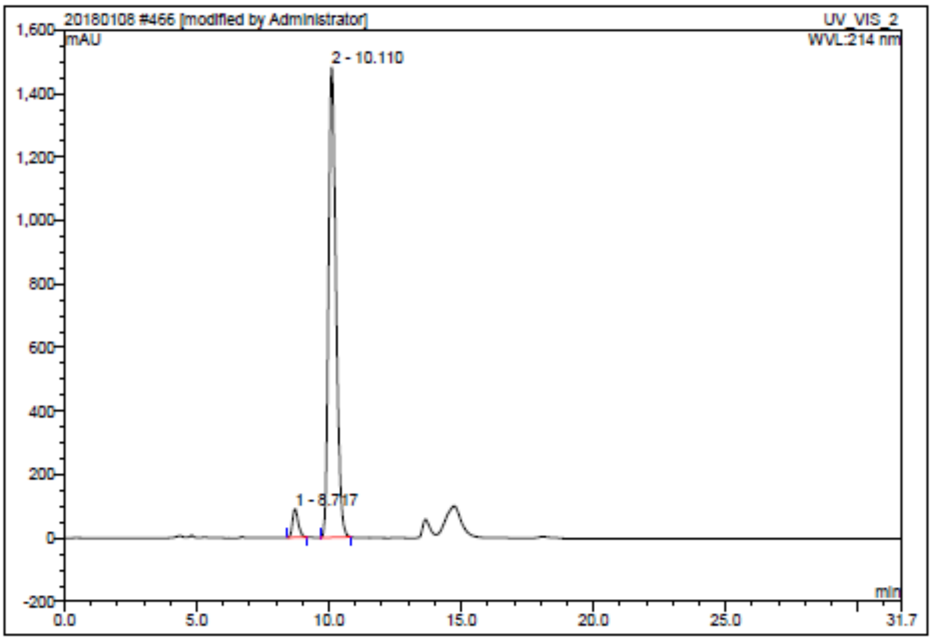


| No. | Ret.Time min | Peak Name | Height mAU | Area mAU*min | Rel.Area % | Amount | Type |
|---|---|---|---|---|---|---|---|
| 1 | 8.72 | n.a. | 90.015 | 22.658 | 4.44 | n.a. | BMB* |
| 2 | 10.11 | n.a. | 1480.532 | 487.329 | 95.56 | n.a. | BMB* |
| Total: | | | 1570.546 | 509.987 | 100.00 | 0.000 | |

**Supplementary Figure 54**. HPLC chromatogram for compound **3v**

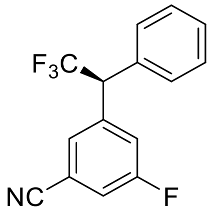


Compound **3w**. SFC (OJ-H, 0.46 × 25 cm, 5 μm, $CO_2$/MeOH = 98/2, flow 2.0 mL/min, column temperature: 40 °C, background press: 2000 psi, UV detection at 214 nm) retention time = 3.84 min (major) and 3.99 min (minor). $[\alpha]_D^{25}$ = 25.1938 (c = 0.0650, $CHCl_3$, 94:6 e.r.).

Empower 3 SOFTWARE

SAMPLE INFORMATION

| | | | |
|---|---|---|---|
| Sample Name: | HWC-9-69-3B+- OJH9822141200040 | Acquired By: | System |
| Sample Type: | | Date Acquired: | 2018/3/21 16:27:00 CST |
| Vial: | 1:A,3 | Acq. Method Set: | chiral_isocratic |
| Injection: | 1 | Date Processed: | 2018/5/28 9:17:04 CST |
| Injection Volume: | 3.00 ul | Processing Method | 1 |
| Run Time: | 60.0 Minutes | Channel Name: | PDA Ch1 214 nm@1.2 nm |
| Sample Set Name | 20180306 | Proc. Chnl. Descr. | PDA Ch1 214 nm@1.2 nm |

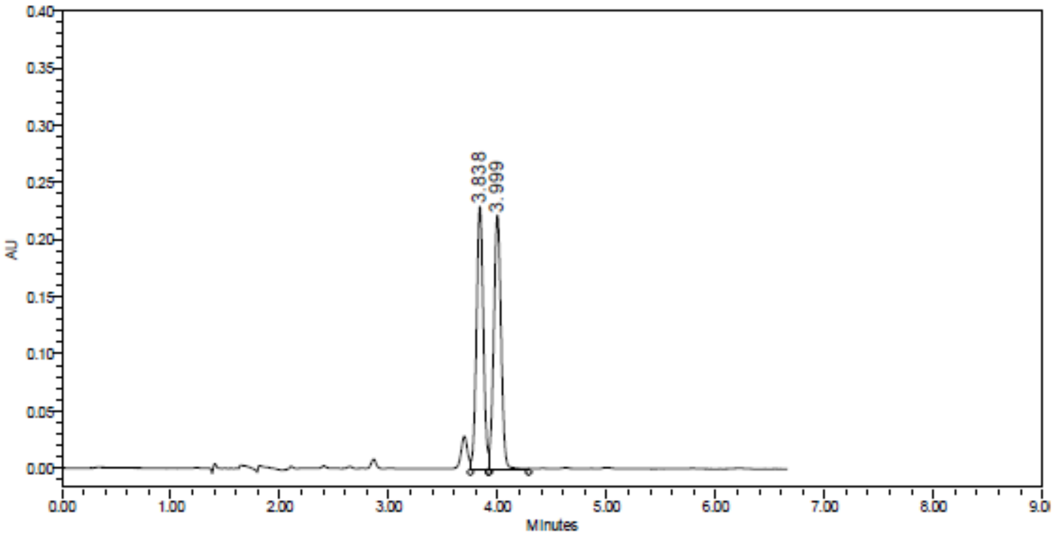


Peak Results

| | RT | Area | Height | % Area |
|---|---|---|---|---|
| 1 | 3.838 | 997793 | 229516 | 49.21 |
| 2 | 3.999 | 1029991 | 221941 | 50.79 |

**Supplementary Figure 55**. HPLC chromatogram for compound **3w**, racemic

## SAMPLE INFORMATION

| | | | |
|---|---|---|---|
| Sample Name: | HWC-9-69-3A OJH9822141200040 | Acquired By: | System |
| Sample Type: | | Date Acquired: | 2018/3/21 16:35:17 CST |
| Vial: | 1:A,4 | Acq. Method Set: | chiral_isocratic |
| Injection: | 1 | Date Processed: | 2018/5/28 9:17:29 CST |
| Injection Volume: | 3.00 ul | Processing Method | 1 |
| Run Time: | 10.0 Minutes | Channel Name: | PDA Ch1 214 nm@1.2 nm |
| Sample Set Name | 20180306 | Proc. Chnl. Descr: | PDA Ch1 214 nm@1.2 nm |

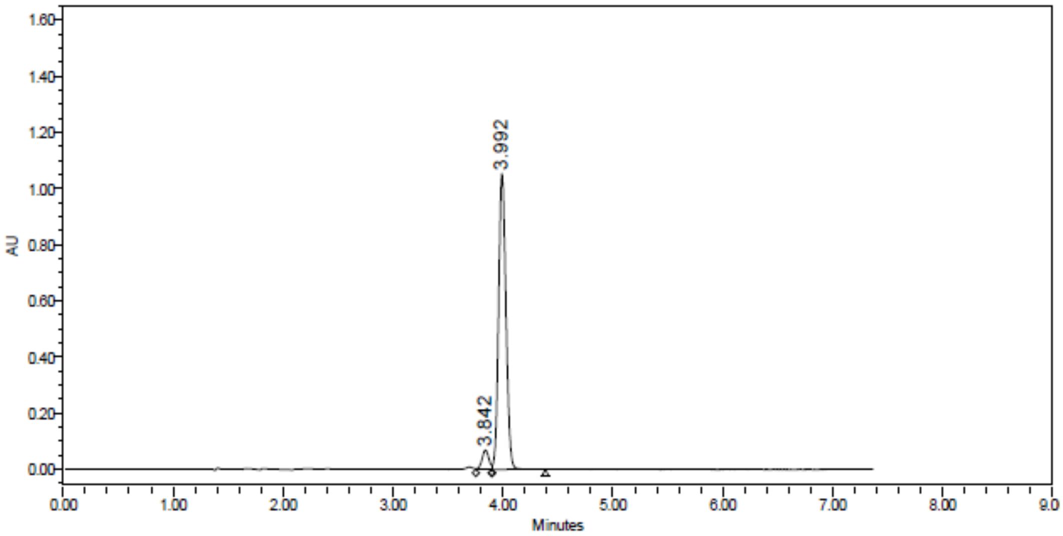


Peak Results

| | RT | Area | Height | % Area |
|---|---|---|---|---|
| 1 | 3.842 | 293756 | 69009 | 5.65 |
| 2 | 3.992 | 4908684 | 1053902 | 94.35 |

**Supplementary Figure 56**. HPLC chromatogram for compound **3w**

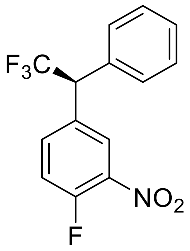


Compound **3x**. HPLC (IF3, 0.46 × 25 cm, 3 μm, hexane/isopropanol = 98/2 (v/v %), flow 0.7 mL/min, UV detection at 214 nm), retention time = 8.33 min (minor) and 8.65 min (major). $[\alpha]_D^{25}$ = -12.3927 (c = 0.0800, $CHCl_3$, 93:7 e.r).

**6019 HWC-9-66-3B+- IF3 982 214 0.7**

| | | | |
|---|---|---|---|
| *Sample Name:* | HWC-9-66-3B+- IF3 982 214 0.7 | *Injection Volume:* | 3.0 |
| *Vial Number:* | RE3 | *Channel:* | UV_VIS_1 |
| *Sample Type:* | unknown | *Wavelength:* | 214 |
| *Control Program:* | 201701-3 | *Bandwidth:* | n.a. |
| *Quantif. Method:* | 201701 | *Dilution Factor:* | 1.0000 |
| *Recording Time:* | 2018/3/19 10:53 | *Sample Weight:* | 1.0000 |
| *Run Time (min):* | 25.61 | *Sample Amount:* | 1.0000 |

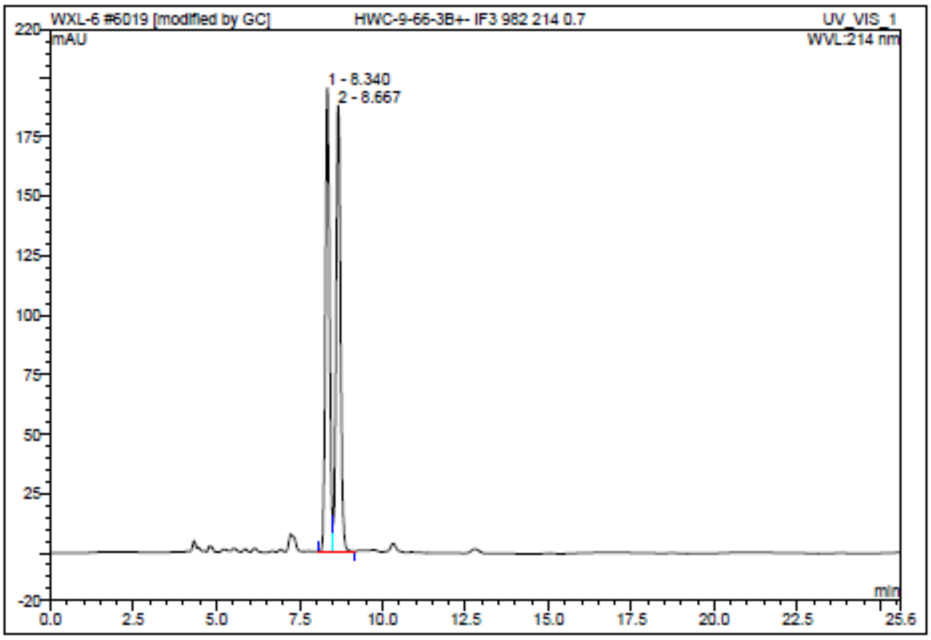


| No. | Ret.Time min | Peak Name | Height mAU | Area mAU*min | Rel.Area % | Amount | Type |
|---|---|---|---|---|---|---|---|
| 1 | 8.34 | n.a. | 194.823 | 28.609 | 49.67 | n.a. | BM |
| 2 | 8.67 | n.a. | 187.484 | 28.994 | 50.33 | n.a. | MB |
| Total: | | | 382.307 | 57.603 | 100.00 | 0.000 | |

**Supplementary Figure 57**. HPLC chromatogram for compound **3x**, racemic

Operator:GC Timebase:U3000 Sequence:WXL-6 Page
2018/5/26 9:48

## 6020 HWC-9-66-3A IF3 982 214 0.7

| | | | |
|---|---|---|---|
| Sample Name: | HWC-9-66-3A IF3 982 214 0.7 | Injection Volume: | 2.0 |
| Vial Number: | RE4 | Channel: | UV_VIS_1 |
| Sample Type: | unknown | Wavelength: | 214 |
| Control Program: | 201701-3 | Bandwidth: | n.a. |
| Quantif. Method: | 201701 | Dilution Factor: | 1.0000 |
| Recording Time: | 2018/3/19 11:21 | Sample Weight: | 1.0000 |
| Run Time (min): | 18.56 | Sample Amount: | 1.0000 |

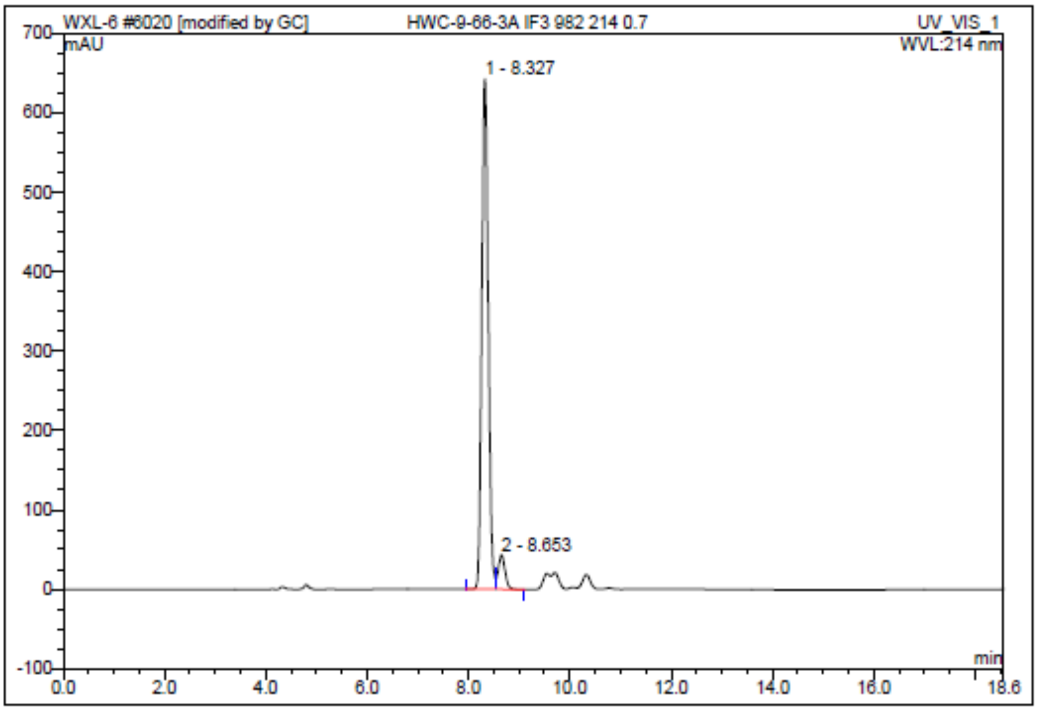


| No. | Ret.Time min | Peak Name | Height mAU | Area mAU*min | Rel.Area % | Amount | Type |
|---|---|---|---|---|---|---|---|
| 1 | 8.33 | n.a. | 642.514 | 95.394 | 93.40 | n.a. | BM |
| 2 | 8.65 | n.a. | 43.207 | 6.745 | 6.60 | n.a. | MB |
| Total: | | | 685.721 | 102.139 | 100.00 | 0.000 | |

**Supplementary Figure 58**. HPLC chromatogram for compound **3x**, racemic

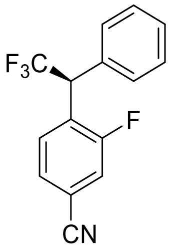


Compound **3y**. HPLC (IF3, 0.46 × 25 cm, 5 μm, hexane/isopropanol = 98/2 (v/v %), flow 0.7 mL/min, UV detection at 214 nm), retention time = 7.89 min (minor) and 8.30 min (major). $[\alpha]_D^{25}$ = 22.8189 (c = 0.1500, $CHCl_3$, 98:2 e.r.).

Operator:Administrator Timebase:HPLC Sequence:20180108 Page
2018-5-26 10:29

**1223 HWC-9-70-3B+- IF3 982 214 0.7**

| | | | |
|---|---|---|---|
| *Sample Name:* | **HWC-9-70-3B+- IF3 982 214 0.7** | *Injection Volume:* | **2.0** |
| *Vial Number:* | **RD7** | *Channel:* | **UV_VIS_2** |
| *Sample Type:* | **unknown** | *Wavelength:* | **214.0** |
| *Control Program:* | **test-dad4** | *Bandwidth:* | **4** |
| *Quantif. Method:* | **20170608** | *Dilution Factor:* | **1.0000** |
| *Recording Time:* | **2018-3-21 14:08** | *Sample Weight:* | **1.0000** |
| *Run Time (min):* | **10.98** | *Sample Amount:* | **1.0000** |

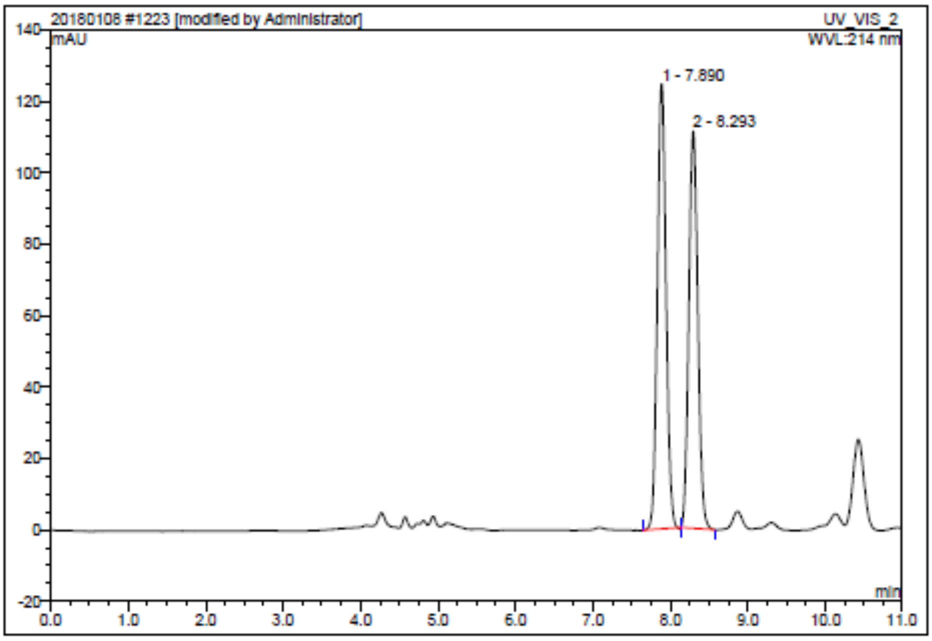


| No. | Ret.Time min | Peak Name | Height mAU | Area mAU*min | Rel.Area % | Amount | Type |
|---|---|---|---|---|---|---|---|
| 1 | 7.89 | n.a. | 124.531 | 16.180 | 51.84 | n.a. | BMb* |
| 2 | 8.29 | n.a. | 111.166 | 15.029 | 48.16 | n.a. | bMB* |
| Total: | | | 235.697 | 31.209 | 100.00 | 0.000 | |

**Supplementary Figure 59**. HPLC chromatogram for compound **3y**, racemic

**1224 HWC-9-70-3A IF3 982 214 0.7**

| | | | |
|---|---|---|---|
| *Sample Name:* | HWC-9-70-3A IF3 982 214 0.7 | *Injection Volume:* | 2.0 |
| *Vial Number:* | RD7 | *Channel:* | UV_VIS_2 |
| *Sample Type:* | unknown | *Wavelength:* | 214.0 |
| *Control Program:* | test-dad4 | *Bandwidth:* | 4 |
| *Quantif. Method:* | 20170608 | *Dilution Factor:* | 1.0000 |
| *Recording Time:* | 2018-3-21 14:21 | *Sample Weight:* | 1.0000 |
| *Run Time (min):* | 34.09 | *Sample Amount:* | 1.0000 |

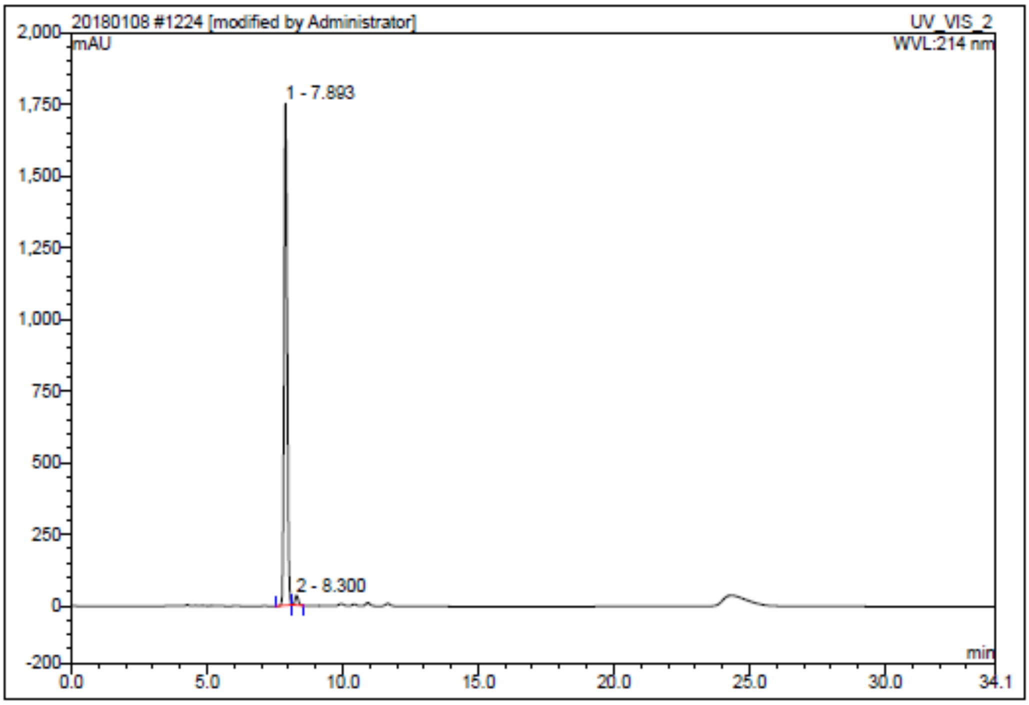


| No. | Ret.Time min | Peak Name | Height mAU | Area mAU*min | Rel.Area % | Amount | Type |
|---|---|---|---|---|---|---|---|
| 1 | 7.89 | n.a. | 1750.791 | 228.216 | 98.01 | n.a. | BMb* |
| 2 | 8.30 | n.a. | 34.305 | 4.627 | 1.99 | n.a. | bMB* |
| Total: | | | 1785.096 | 232.843 | 100.00 | 0.000 | |

**Supplementary Figure 60**. HPLC chromatogram for compound **3y**

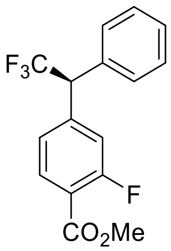


Compound **3z**. SFC (OJ-H, 0.46 × 25 cm, 5 μm, $CO_2$/MeOH = 98/2, flow 2.0 mL/min, column temperature: 40 °C, background press: 2000 psi, UV detection at 214 nm), retention time = 3.78 min (major) and 4.56 min (minor). $[\alpha]_D^{25}$ = 63.6251 (c = 0.0750, $CHCl_3$, 92:8 e.r.).

## SAMPLE INFORMATION

| | | | |
|---|---|---|---|
| Sample Name: | HWC-9-65-3B+- OJH9822141200040 | Acquired By: | System |
| Sample Type: | | Date Acquired: | 2018/3/21 16:02:12 CST |
| Vial: | 1:A,1 | Acq. Method Set: | chiral_isocratic |
| Injection: | 1 | Date Processed: | 2018/5/28 9:18:09 CST |
| Injection Volume: | 3.00 ul | Processing Method | 1 |
| Run Time: | 60.0 Minutes | Channel Name: | PDA Ch1 214 nm@1.2 nm |
| Sample Set Name | 20180306 | Proc. Chnl. Descr: | PDA Ch1 214 nm@1.2 nm |

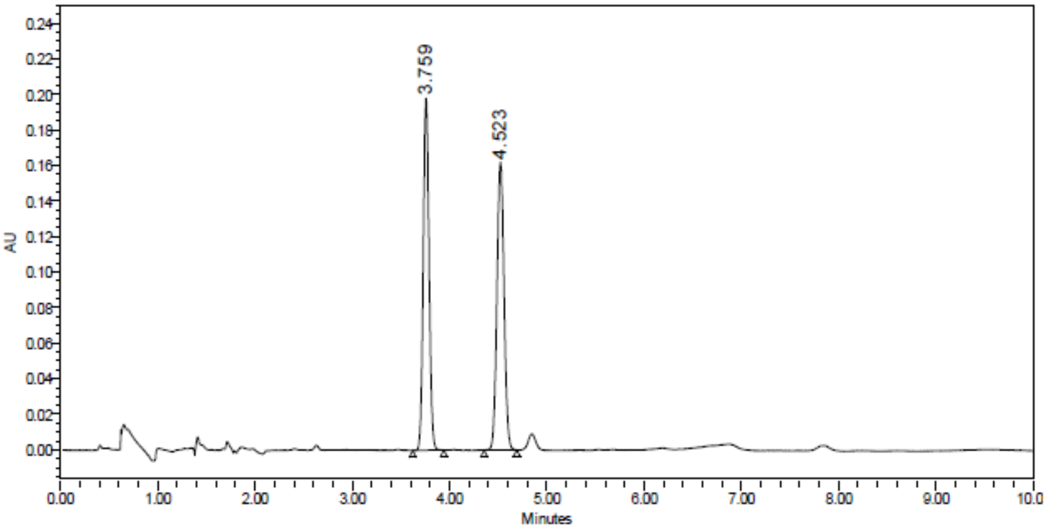


Peak Results

| | RT | Area | Height | % Area |
|---|---|---|---|---|
| 1 | 3.759 | 818494 | 198128 | 49.97 |
| 2 | 4.523 | 819400 | 161900 | 50.03 |

**Supplementary Figure 61**. HPLC chromatogram for compound **3z**, racemic

## SAMPLE INFORMATION

| | | | |
|---|---|---|---|
| Sample Name: | HWC-9-65-3A OJH9822141200040 | Acquired By: | System |
| Sample Type: | | Date Acquired: | 2018/3/21 16:16:31 CST |
| Vial: | 1:A,2 | Acq. Method Set: | chiral_isocratic |
| Injection: | 1 | Date Processed: | 2018/5/28 9:18:51 CST |
| Injection Volume: | 2.00 ul | Processing Method | 1 |
| Run Time: | 10.0 Minutes | Channel Name: | PDA Ch1 214 nm@1.2 nm |
| Sample Set Name | 20180306 | Proc. Chnl. Descr. | PDA Ch1 214 nm@1.2 nm |

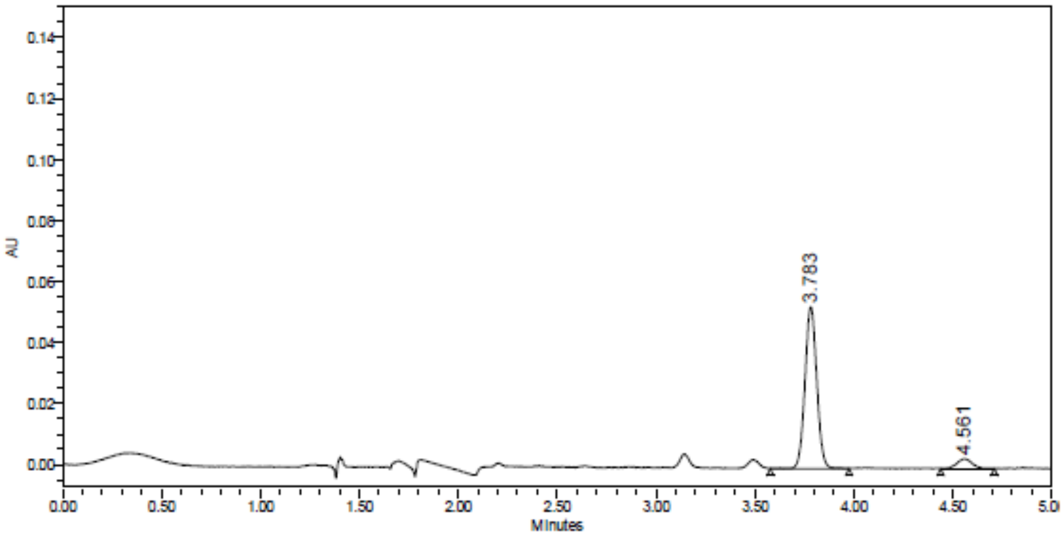


**Peak Results**

| | RT | Area | Height | % Area |
|---|---|---|---|---|
| 1 | 3.783 | 220438 | 52956 | 92.33 |
| 2 | 4.561 | 18304 | 3341 | 7.67 |

**Supplementary Figure 62**. HPLC chromatogram for compound **3z**

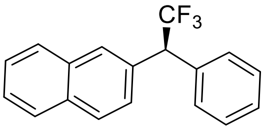


Compound **3aa**. HPLC (OJ-H, 0.46 × 25 cm, 5 µm, hexane/isopropanol = 95/5 (v/v %), flow 0.7 mL/min, UV detection at 214 nm), retention time = 21.66 min (minor) and 24.82 min (major). $[\alpha]_D^{25}$ = 23.6295 (c = 0.0600, $CHCl_3$, 95:5 e.r.).

**516 HWC-9-3-3B+- OJH 955 214 0.7**

| | | | |
|---|---|---|---|
| *Sample Name:* | HWC-9-3-3B+- OJH 955 214 0.7 | *Injection Volume:* | 3.0 |
| *Vial Number:* | GB2 | *Channel:* | UV_VIS_2 |
| *Sample Type:* | unknown | *Wavelength:* | 214.0 |
| *Control Program:* | test-dad6 | *Bandwidth:* | 4 |
| *Quantif. Method:* | 20170608 | *Dilution Factor:* | 1.0000 |
| *Recording Time:* | 2018-1-25 9:01 | *Sample Weight:* | 1.0000 |
| *Run Time (min):* | 33.74 | *Sample Amount:* | 1.0000 |

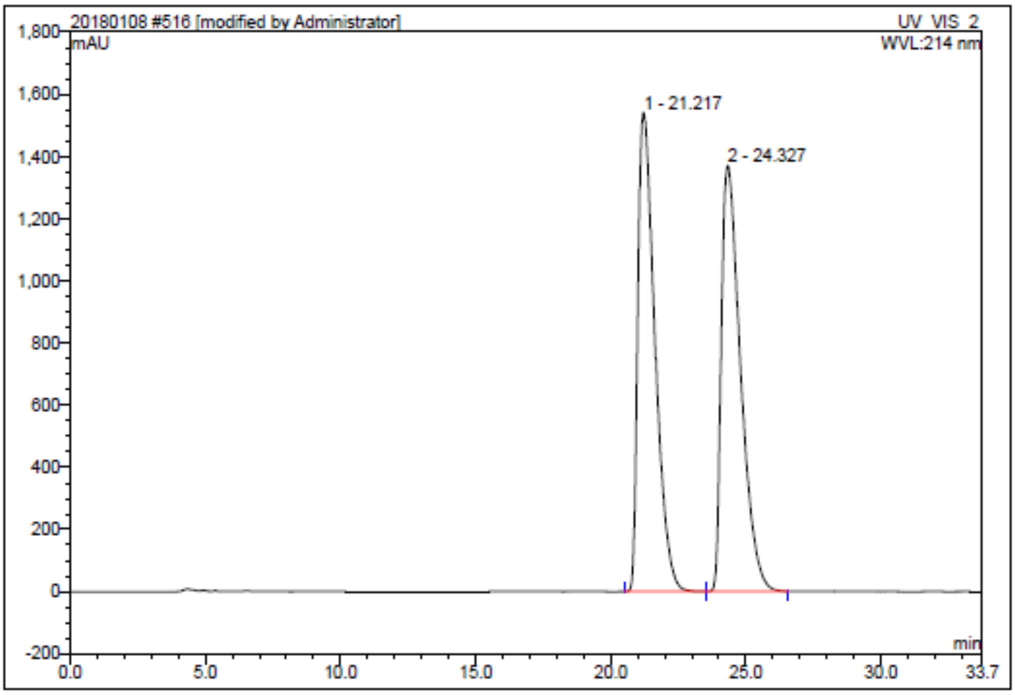


| No. | Ret.Time min | Peak Name | Height mAU | Area mAU*min | Rel.Area % | Amount | Type |
|---|---|---|---|---|---|---|---|
| 1 | 21.22 | n.a. | 1541.310 | 1128.982 | 49.96 | n.a. | BM |
| 2 | 24.33 | n.a. | 1369.784 | 1130.665 | 50.04 | n.a. | MB |
| Total: | | | 2911.094 | 2259.647 | 100.00 | 0.000 | |

**Supplementary Figure 63**. HPLC chromatogram for compound **3aa**, racemic

| 518 HWC-9-4-3F OJH 955 214 0.7 | | | |
|---|---|---|---|
| *Sample Name:* | **HWC-9-4-3F OJH 955 214 0.7** | *Injection Volume:* | **1.0** |
| *Vial Number:* | **GB3** | *Channel:* | **UV_VIS_2** |
| *Sample Type:* | **unknown** | *Wavelength:* | **214.0** |
| *Control Program:* | **test-dad6** | *Bandwidth:* | **4** |
| *Quantif. Method:* | **20170608** | *Dilution Factor:* | **1.0000** |
| *Recording Time:* | **2018-1-25 9:37** | *Sample Weight:* | **1.0000** |
| *Run Time (min):* | **36.74** | *Sample Amount:* | **1.0000** |

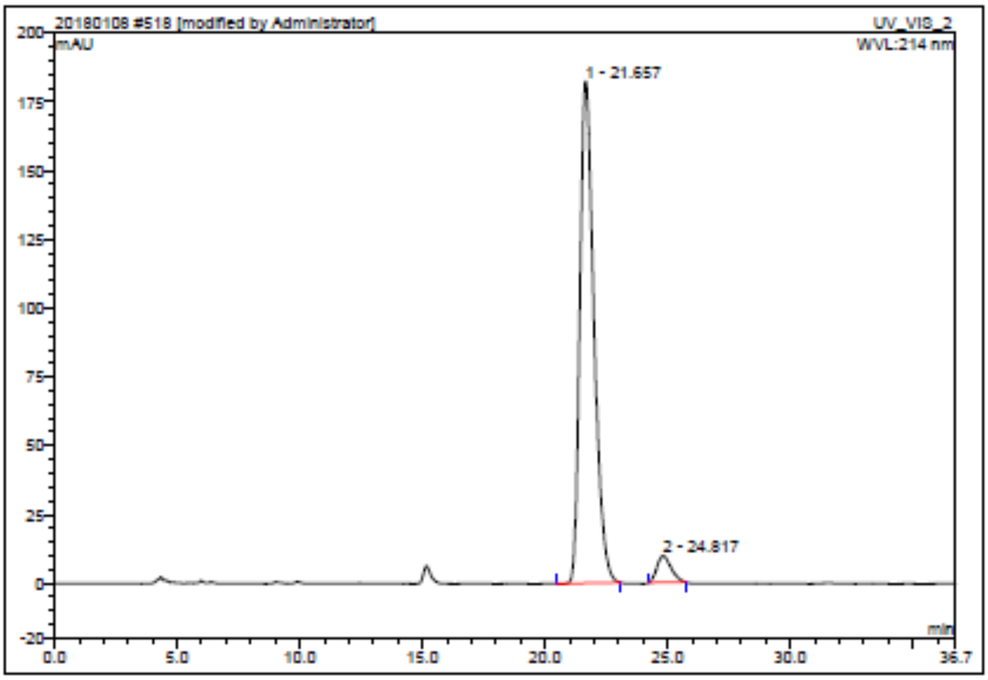


| No. | Ret.Time min | Peak Name | Height mAU | Area mAU*min | Rel.Area % | Amount | Type |
|---|---|---|---|---|---|---|---|
| 1 | 21.66 | n.a. | 182.515 | 121.140 | 94.91 | n.a. | BMB* |
| 2 | 24.82 | n.a. | 9.669 | 6.492 | 5.09 | n.a. | BMB* |
| Total: | | | 192.184 | 127.632 | 100.00 | 0.000 | |

**Supplementary Figure 64**. HPLC chromatogram for compound **3aa**

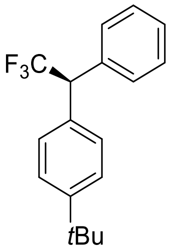


Compound **3ab**. HPLC (OJ-H, 0.46 × 25 cm, 5 µm, hexane/isopropanol = 98/2 (v/v %), flow 0.7 mL/min, UV detection at 214 nm), retention time = 11.49 min (minor) and 12.26 min (major). $[\alpha]_D^{25}$ = 1.0500 (c = 0.4000, $CHCl_3$, 90:10 e.r.).

**484 HWC-8-32-3B+- OJH 982 214 0.7**

| | | | |
|---|---|---|---|
| *Sample Name:* | HWC-8-32-3B+- OJH 982 214 0.7 | *Injection Volume:* | 3.0 |
| *Vial Number:* | GA4 | *Channel:* | UV_VIS_2 |
| *Sample Type:* | unknown | *Wavelength:* | 214.0 |
| *Control Program:* | test-dad2 | *Bandwidth:* | 4 |
| *Quantif. Method:* | 20170608 | *Dilution Factor:* | 1.0000 |
| *Recording Time:* | 2018-1-23 12:15 | *Sample Weight:* | 1.0000 |
| *Run Time (min):* | 30.00 | *Sample Amount:* | 1.0000 |

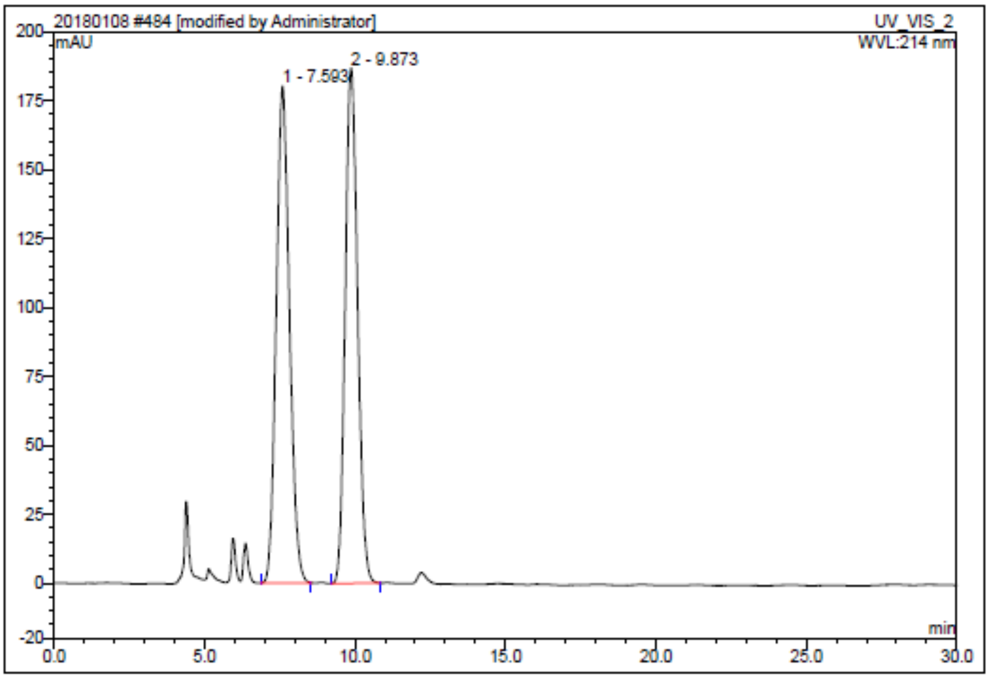


| No. | Ret.Time min | Peak Name | Height mAU | Area mAU*min | Rel.Area % | Amount | Type |
|---|---|---|---|---|---|---|---|
| 1 | 7.59 | n.a. | 180.177 | 91.613 | 50.71 | n.a. | BMB |
| 2 | 9.87 | n.a. | 186.837 | 89.032 | 49.29 | n.a. | BMB |
| Total: | | | 367.013 | 180.645 | 100.00 | 0.000 | |

**Supplementary Figure 65**. HPLC chromatogram for compound **3ab**, racemic

Operator:Administrator Timebase:HPLC Sequence:20180108 Page 1
2019-4-1 2:01

**485 HWC-9-1-3F OJH 982 214 0.7**

| | | | |
|---|---|---|---|
| *Sample Name:* | HWC-9-1-3F OJH 982 214 0.7 | *Injection Volume:* | 2.0 |
| *Vial Number:* | GC3 | *Channel:* | UV_VIS_2 |
| *Sample Type:* | unknown | *Wavelength:* | 214.0 |
| *Control Program:* | test-dad2 | *Bandwidth:* | 4 |
| *Quantif. Method:* | 20170608 | *Dilution Factor:* | 1.0000 |
| *Recording Time:* | 2018-1-23 14:33 | *Sample Weight:* | 1.0000 |
| *Run Time (min):* | 18.20 | *Sample Amount:* | 1.0000 |

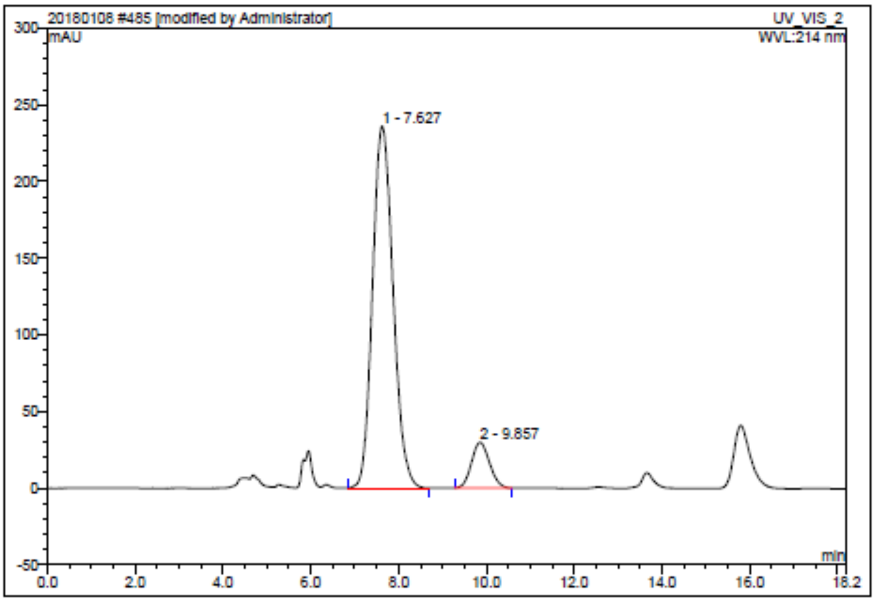


| No. | Ret.Time min | Peak Name | Height mAU | Area mAU*min | Rel.Area % | Amount | Type |
|---|---|---|---|---|---|---|---|
| 1 | 7.63 | n.a. | 236.024 | 127.995 | 90.02 | n.a. | BMB |
| 2 | 9.86 | n.a. | 29.801 | 14.191 | 9.98 | n.a. | BMB |
| Total: | | | 265.824 | 142.186 | 100.00 | 0.000 | |

**Supplementary Figure 66**. HPLC chromatogram for compound **3ab**

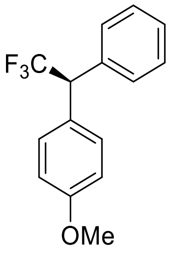

Compound **3ac**. HPLC (AD-H, 0.46 × 25 cm, 5 μm, hexane/isopropanol = 92/8 (v/v %), flow 0.7 mL/min, UV detection at 214 nm), retention time = 6.46 min (minor) and 7.09 min (major). $[\alpha]_D^{25}$ = -2.740 (c = 0.1900, $CHCl_3$, 90:10 e.r.).

Empower 3 SOFTWARE 1

## SAMPLE INFORMATION

| | | | |
|---|---|---|---|
| Sample Name: | HWC-14-25-3+- ADH 928 214 0.7 | Acquired By: | System |
| Sample Type: | Unknown | Sample Set Name: | 1 |
| Vial: | 1:E,6 | Acq. Method Set: | 1017 |
| Injection #: | 1 | Processing Method: | 1 |
| Injection Volume: | 2.00 ul | Channel Name: | PDA Ch1 214nm@4.8nm |
| Run Time: | 12.0 Minutes | Proc. Chnl. Descr.: | PDA Ch1 214nm@4.8nm |
| Date Acquired: | 3/29/2019 7:11:26 PM CST | | |
| Date Processed: | 4/1/2019 1:52:09 PM CST | | |

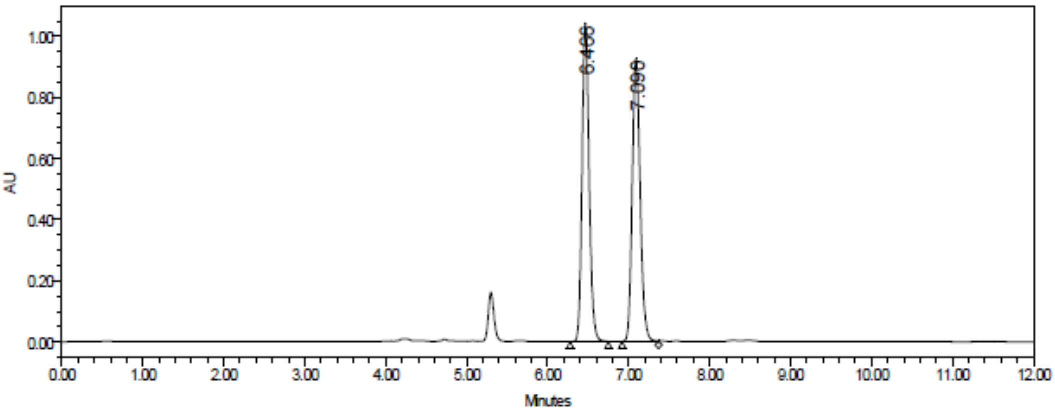


| | RT | Area | % Area | Height |
|---|---|---|---|---|
| 1 | 6.466 | 6306752 | 50.08 | 104111 |
| 2 | 7.090 | 6285922 | 49.92 | 924037 |

**Supplementary Figure 67**. HPLC chromatogram for compound **3ac**, racemic

## SAMPLE INFORMATION

| | | | |
|---|---|---|---|
| Sample Name: | HWC-14-26-3 ADH 928 214 0.7 | Acquired By: | System |
| Sample Type: | Unknown | Sample Set Name: | 1 |
| Vial: | 1:E,7 | Acq. Method Set: | 1017 |
| Injection #: | 1 | Processing Method: | 1 |
| Injection Volume: | 2.00 ul | Channel Name: | PDA Ch1 214nm@4.8nm |
| Run Time: | 12.0 Minutes | Proc. Chnl. Descr.: | PDA Ch1 214nm@4.8nm |
| Date Acquired: | 3/29/2019 7:24:02 PM CST | | |
| Date Processed: | 4/1/2019 1:51:42 PM CST | | |

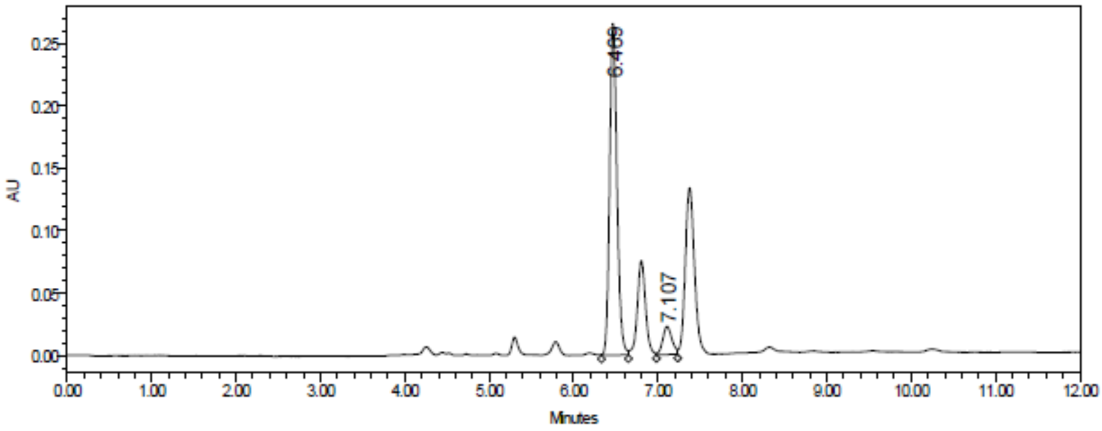


| | RT | Area | % Area | Height |
|---|---|---|---|---|
| 1 | 6.469 | 1603286 | 90.38 | 265292 |
| 2 | 7.107 | 170614 | 9.62 | 22483 |

**Supplementary Figure 68**. HPLC chromatogram for compound **3ac**

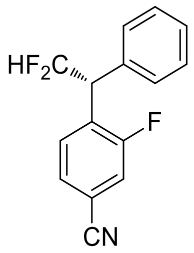


Compound **4a**. HPLC (IE3, 0.46 × 25 cm, 5 μm, hexane/ isopropanol = 99/1 (v/v %), flow 0.7 mL/min, UV detection at 214 nm), retention time = 16.10 min (minor) and 16.60 min (major). $[\alpha]_D^{25}$ = -3.90 (c = 0.2050, $CHCl_3$, 94:6 e.r.).

**2957 HWC-11-53-3B IE3 991 214 0.7**

| | | | |
|---|---|---|---|
| *Sample Name:* | HWC-11-53-3B IE3 991 214 0.7 | *Injection Volume:* | 2.0 |
| *Vial Number:* | GB7 | *Channel:* | UV_VIS_2 |
| *Sample Type:* | unknown | *Wavelength:* | 214.0 |
| *Control Program:* | test-dad3 | *Bandwidth:* | 4 |
| *Quantif. Method:* | 20170608 | *Dilution Factor:* | 1.0000 |
| *Recording Time:* | 2018-9-3 14:34 | *Sample Weight:* | 1.0000 |
| *Run Time (min):* | 23.64 | *Sample Amount:* | 1.0000 |

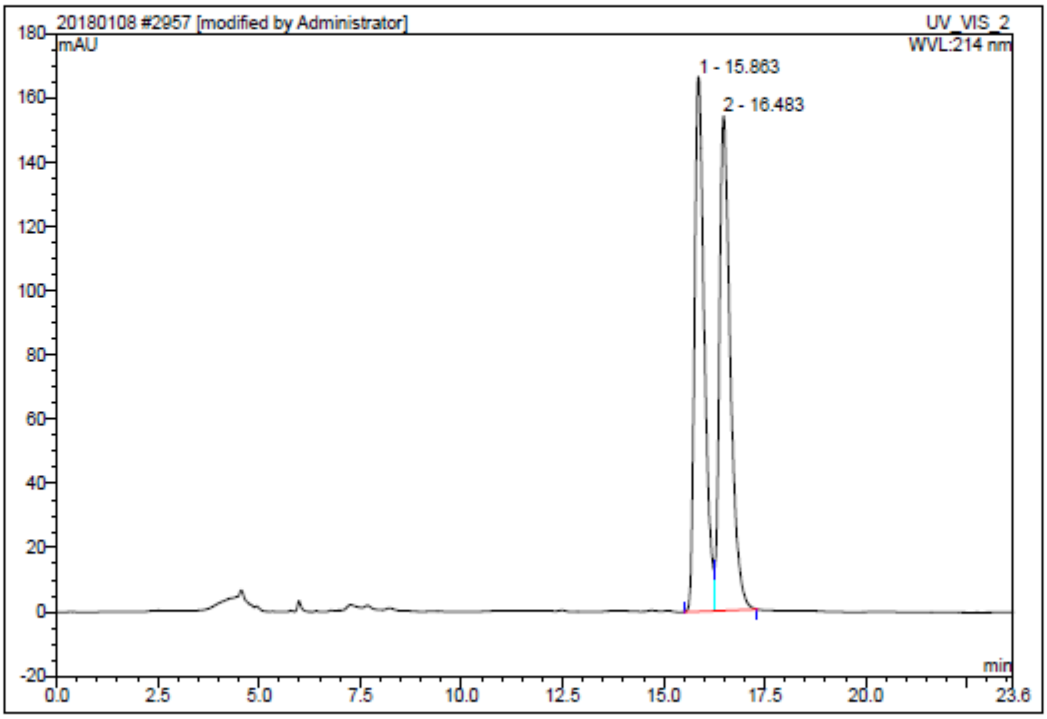


| No. | Ret.Time min | Peak Name | Height mAU | Area mAU*min | Rel.Area % | Amount | Type |
|---|---|---|---|---|---|---|---|
| 1 | 15.86 | n.a. | 166.343 | 46.715 | 49.11 | n.a. | BM |
| 2 | 16.48 | n.a. | 153.858 | 48.407 | 50.89 | n.a. | MB |
| Total: | | | 320.201 | 95.122 | 100.00 | 0.000 | |

**Supplementary Figure 69**. HPLC chromatogram for compound **4a**, racemic

Operator:Administrator Timebase:HPLC Sequence:20180108 Page 1-1
2018-12-25 9:33 下午

**2956 HWC-11-53-3A+- IE3 991 214 0.7**

| | | | |
|---|---|---|---|
| *Sample Name:* | **HWC-11-53-3A+- IE3 991 214 0.7** | *Injection Volume:* | **2.0** |
| *Vial Number:* | **GA7** | *Channel:* | **UV_VIS_2** |
| *Sample Type:* | **unknown** | *Wavelength:* | **214.0** |
| *Control Program:* | **test-dad3** | *Bandwidth:* | **4** |
| *Quantif. Method:* | **20170608** | *Dilution Factor:* | **1.0000** |
| *Recording Time:* | **2018-9-3 14:07** | *Sample Weight:* | **1.0000** |
| *Run Time (min):* | **25.76** | *Sample Amount:* | **1.0000** |

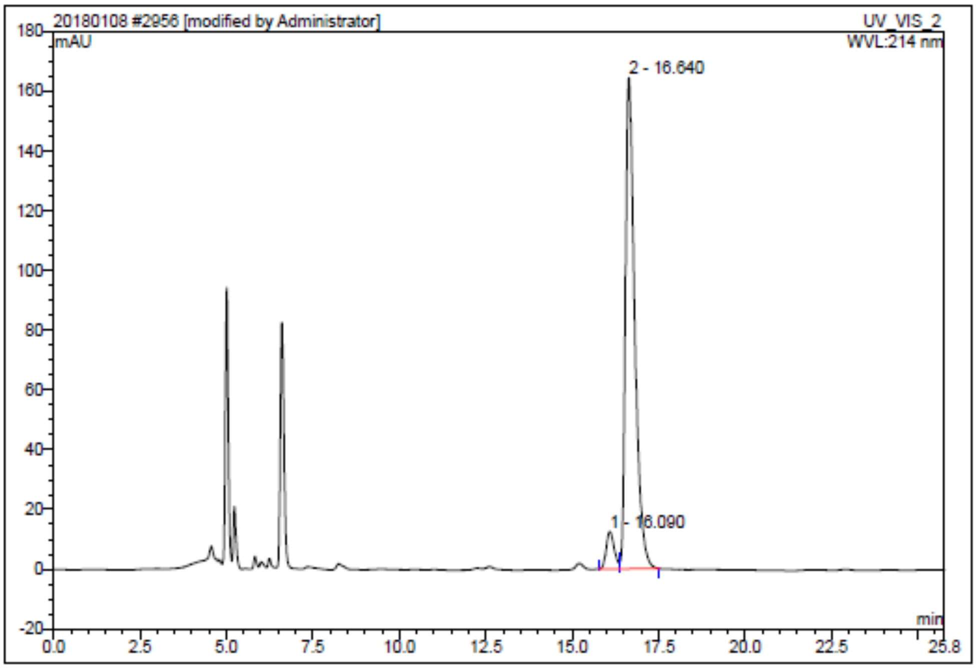


| No. | Ret.Time min | Peak Name | Height mAU | Area mAU*min | Rel.Area % | Amount | Type |
|---|---|---|---|---|---|---|---|
| 1 | 16.09 | n.a. | 12.464 | 3.342 | 6.12 | n.a. | BM |
| 2 | 16.64 | n.a. | 164.205 | 51.303 | 93.88 | n.a. | MB |
| **Total:** | | | 176.669 | 54.645 | 100.00 | 0.000 | |

**Supplementary Figure 70**. HPLC chromatogram for compound **4a**

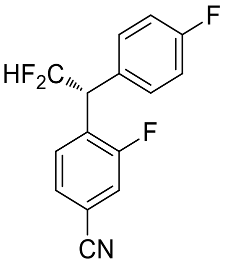


Compound **4b**. HPLC (IA, 0.46 × 25 cm, 5 μm, hexane/ isopropanol = 95/5 (v/v %), flow 0.7 mL/min, UV detection at 214 nm), retention time = 12.98 min (minor) and 13.88 min (major). $[\alpha]_D^{25}$ = -13.63 (c = 0.1350, $CHCl_3$, 94:6 e.r.).

Operator:GC Timebase:U3000 Sequence:WXL-6 — Page 1-1
2018/12/24 11:18 下午

**7619 HWC-11-104-3A+- IA 955 214 0.7**

| | | | |
|---|---|---|---|
| *Sample Name:* | **HWC-11-104-3A+- IA 955 214 0.7** | *Injection Volume:* | **3.0** |
| *Vial Number:* | **GE2** | *Channel:* | **UV_VIS_1** |
| *Sample Type:* | **unknown** | *Wavelength:* | **214** |
| *Control Program:* | **201701-4** | *Bandwidth:* | **n.a.** |
| *Quantif. Method:* | **201701** | *Dilution Factor:* | **1.0000** |
| *Recording Time:* | **2018/10/15 18:33** | *Sample Weight:* | **1.0000** |
| *Run Time (min):* | **25.00** | *Sample Amount:* | **1.0000** |

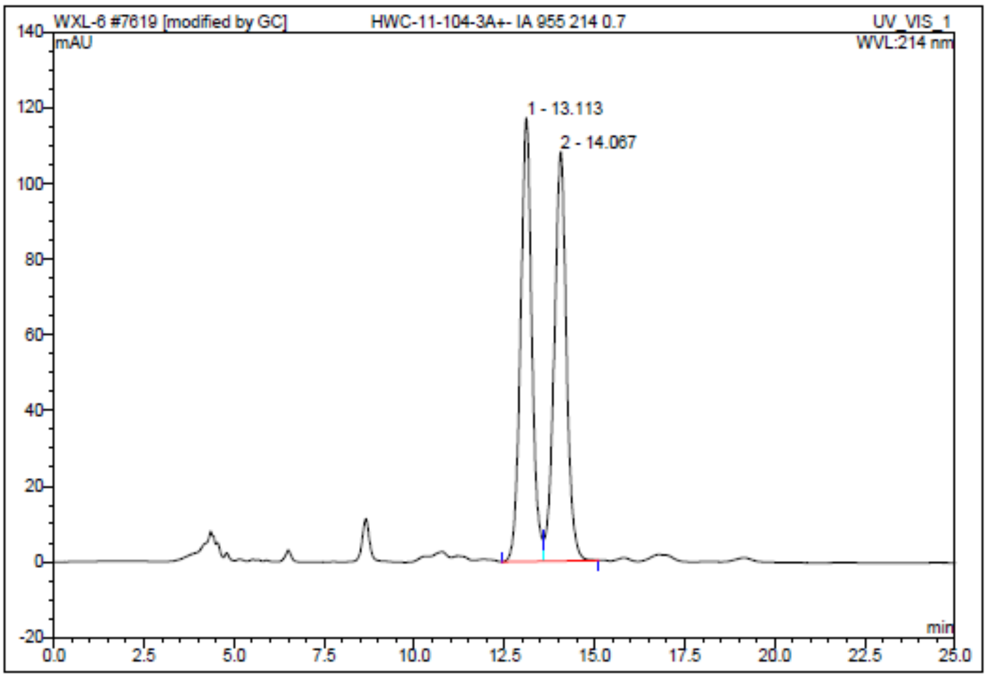


| No. | Ret.Time min | Peak Name | Height mAU | Area mAU*min | Rel.Area % | Amount | Type |
|---|---|---|---|---|---|---|---|
| 1 | 13.11 | n.a. | 117.194 | 42.623 | 49.99 | n.a. | BM |
| 2 | 14.07 | n.a. | 108.096 | 42.647 | 50.01 | n.a. | MB |
| **Total:** | | | 225.290 | 85.270 | 100.00 | 0.000 | |

**Supplementary Figure 71**. HPLC chromatogram for compound **4b**, racemic

Operator:GC Timebase:U3000 Sequence:WXL-6 Page 1-1

2018/12/24 11:18 下午

**7649 HWC-12-2-3 IA 955 214 0.7**

| | | | |
|---|---|---|---|
| *Sample Name:* | **HWC-12-2-3 IA 955 214 0.7** | *Injection Volume:* | **20.0** |
| *Vial Number:* | **GA5** | *Channel:* | **UV_VIS_1** |
| *Sample Type:* | **unknown** | *Wavelength:* | **214** |
| *Control Program:* | **201701-4** | *Bandwidth:* | **n.a.** |
| *Quantif. Method:* | **201701** | *Dilution Factor:* | **1.0000** |
| *Recording Time:* | **2018/10/22 9:41** | *Sample Weight:* | **1.0000** |
| *Run Time (min):* | **40.00** | *Sample Amount:* | **1.0000** |

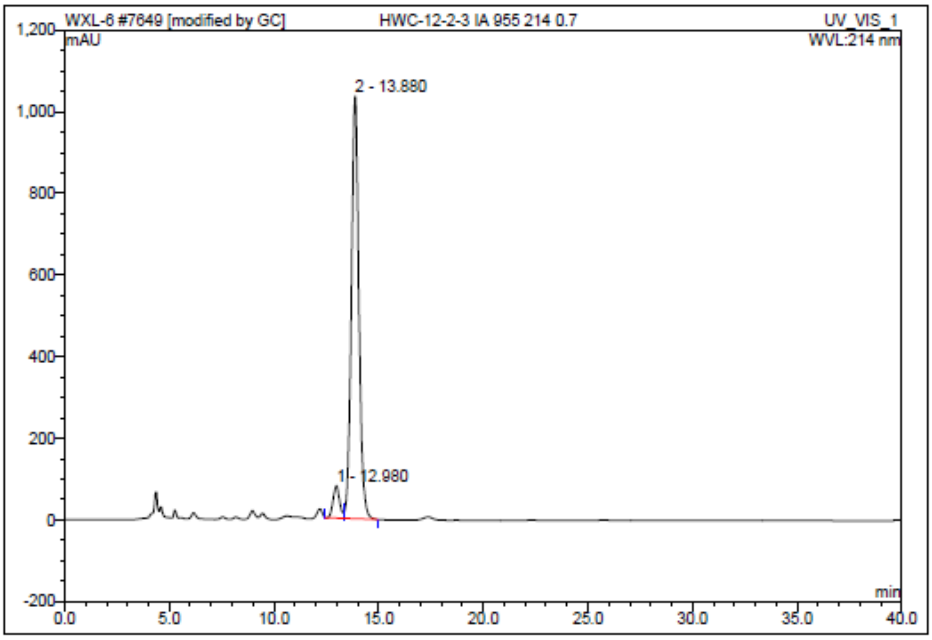


| No. | Ret.Time min | Peak Name | Height mAU | Area mAU*min | Rel.Area % | Amount | Type |
|---|---|---|---|---|---|---|---|
| 1 | 12.98 | n.a. | 79.210 | 28.248 | 6.14 | n.a. | BM * |
| 2 | 13.88 | n.a. | 1035.785 | 431.896 | 93.86 | n.a. | MB* |
| Total: | | | 1114.995 | 460.144 | 100.00 | 0.000 | |

**Supplementary Figure 72**. HPLC chromatogram for compound **4b**

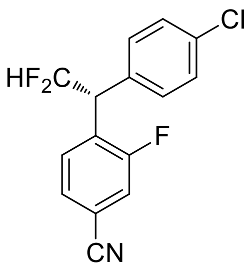


Compound **4c**. HPLC (PA2, 0.46 × 25 cm, 5 μm, hexane/isopropanol = 98/2 (v/v %), flow 0.7 mL/min, UV detection at 214 nm), retention time = 19.60 min (minor) and 20.92 min (major). $[\alpha]_D^{25}$ = 3.24 (c = 0.1050, $CHCl_3$, 91:9 e.r.).

**3320 HWC-11-101-3B PA2 982 214 0.7**

| | | | |
|---|---|---|---|
| *Sample Name:* | **HWC-11-101-3B PA2 982 214 0.7** | *Injection Volume:* | **2.0** |
| *Vial Number:* | **GE7** | *Channel:* | **UV_VIS_2** |
| *Sample Type:* | **unknown** | *Wavelength:* | **214.0** |
| *Control Program:* | **test-dad2** | *Bandwidth:* | **4** |
| *Quantif. Method:* | **20170608** | *Dilution Factor:* | **1.0000** |
| *Recording Time:* | **2018-10-12 15:00** | *Sample Weight:* | **1.0000** |
| *Run Time (min):* | **68.10** | *Sample Amount:* | **1.0000** |

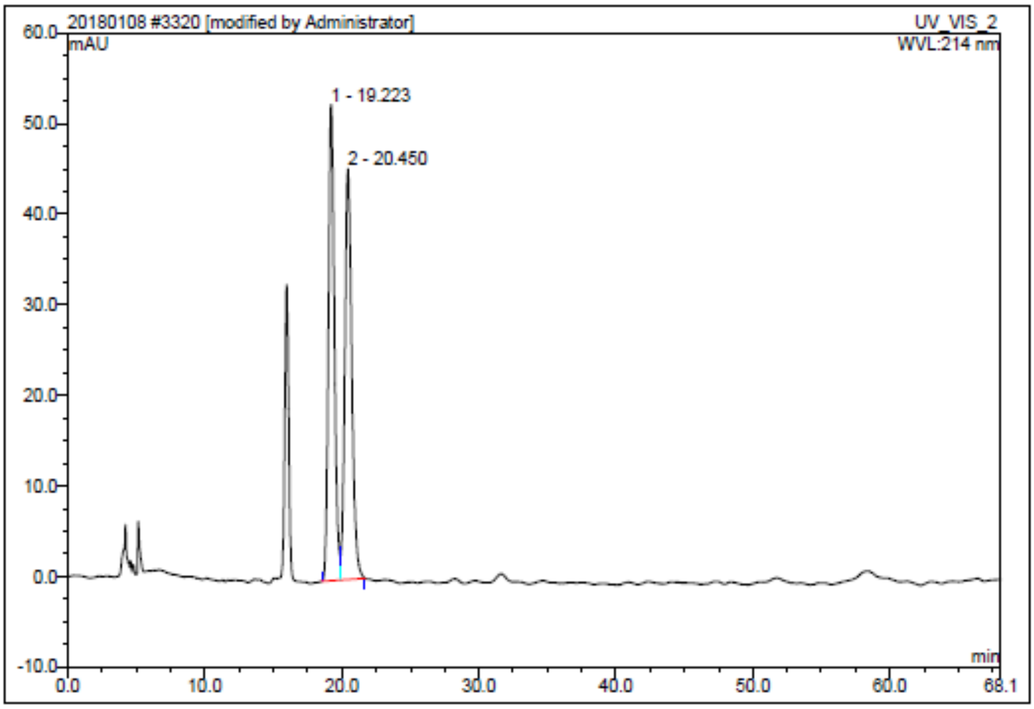


| No. | Ret.Time min | Peak Name | Height mAU | Area mAU*min | Rel.Area % | Amount | Type |
|---|---|---|---|---|---|---|---|
| 1 | 19.22 | n.a. | 52.540 | 26.620 | 49.84 | n.a. | BM |
| 2 | 20.45 | n.a. | 45.363 | 26.786 | 50.16 | n.a. | MB |
| **Total:** | | | 97.902 | 53.407 | 100.00 | 0.000 | |

**Supplementary Figure 73**. HPLC chromatogram for compound **4c**, racemic

**3321 HWC-11-100-3B PA2 982 214 0.7**

| | | | |
|---|---|---|---|
| *Sample Name:* | HWC-11-100-3B PA2 982 214 0.7 | *Injection Volume:* | 2.0 |
| *Vial Number:* | GD6 | *Channel:* | UV_VIS_2 |
| *Sample Type:* | unknown | *Wavelength:* | 214.0 |
| *Control Program:* | test-dad2 | *Bandwidth:* | 4 |
| *Quantif. Method:* | 20170608 | *Dilution Factor:* | 1.0000 |
| *Recording Time:* | 2018-10-12 16:14 | *Sample Weight:* | 1.0000 |
| *Run Time (min):* | 25.00 | *Sample Amount:* | 1.0000 |

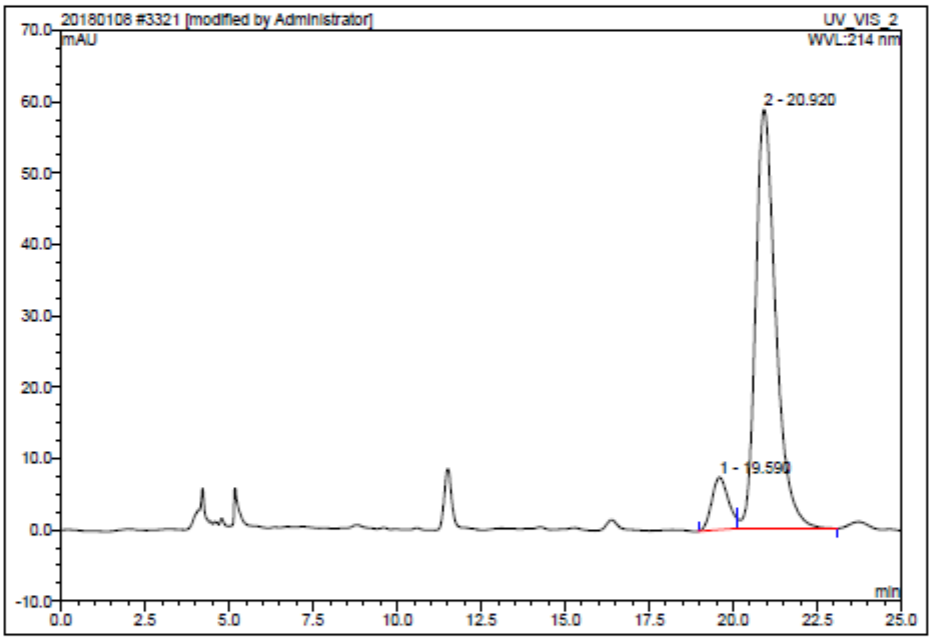


| No. | Ret.Time min | Peak Name | Height mAU | Area mAU*min | Rel.Area % | Amount | Type |
|---|---|---|---|---|---|---|---|
| 1 | 19.59 | n.a. | 7.370 | 4.162 | 9.24 | n.a. | BM * |
| 2 | 20.92 | n.a. | 58.706 | 40.889 | 90.76 | n.a. | MB* |
| Total: | | | 66.077 | 45.051 | 100.00 | 0.000 | |

**Supplementary Figure 74**. HPLC chromatogram for compound **4c**

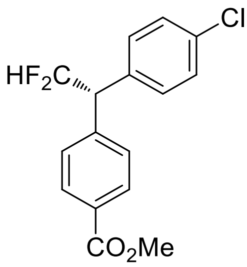


Compound **4d**. HPLC (IB, 0.46 × 25 cm, 5 μm, hexane/isopropanol = 95/5 (v/v %), flow 0.7 mL/min, UV detection at 214 nm), retention time = 10.62 min (minor) and 11.51 min (major). $[\alpha]_D^{25}$ = 4.00 (c = 0.1000, $CHCl_3$, 90:10 e.r.).

**3367 HWC-12-7-3 IG 973 214 0.7**

| | | | |
|---|---|---|---|
| *Sample Name:* | HWC-12-7-3 IG 973 214 0.7 | *Injection Volume:* | 1.0 |
| *Vial Number:* | GD6 | *Channel:* | UV_VIS_2 |
| *Sample Type:* | unknown | *Wavelength:* | 214.0 |
| *Control Program:* | test-dad2 | *Bandwidth:* | 4 |
| *Quantif. Method:* | 20170608 | *Dilution Factor:* | 1.0000 |
| *Recording Time:* | 2018-10-19 14:04 | *Sample Weight:* | 1.0000 |
| *Run Time (min):* | 30.00 | *Sample Amount:* | 1.0000 |

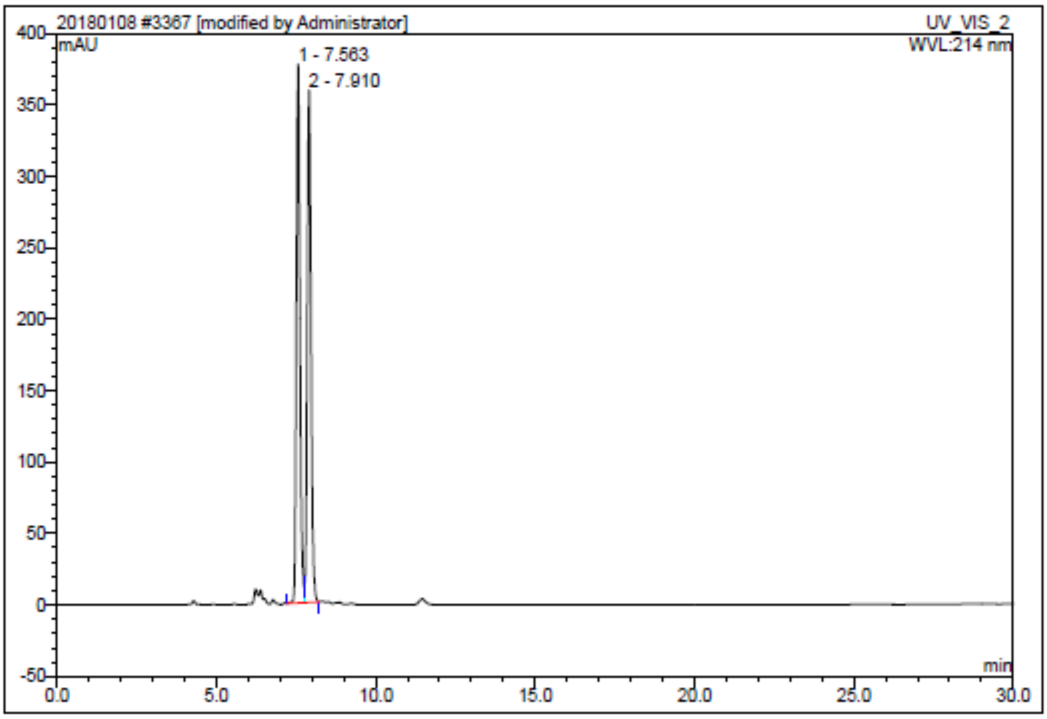


| No. | Ret.Time min | Peak Name | Height mAU | Area mAU*min | Rel.Area % | Amount | Type |
|---|---|---|---|---|---|---|---|
| 1 | 7.56 | n.a. | 377.133 | 48.699 | 49.87 | n.a. | BM |
| 2 | 7.91 | n.a. | 358.575 | 48.955 | 50.13 | n.a. | MB |
| Total: | | | 735.708 | 97.654 | 100.00 | 0.000 | |

**Supplementary Figure 75.** HPLC chromatogram for compound **4d**, racemic

**3355 HWC-12-1-3B IB 955 214 0.7**

| | | | |
|---|---|---|---|
| *Sample Name:* | HWC-12-1-3B IB 955 214 0.7 | *Injection Volume:* | 3.0 |
| *Vial Number:* | GD6 | *Channel:* | UV_VIS_2 |
| *Sample Type:* | unknown | *Wavelength:* | 214.0 |
| *Control Program:* | test-dad2 | *Bandwidth:* | 4 |
| *Quantif. Method:* | 20170608 | *Dilution Factor:* | 1.0000 |
| *Recording Time:* | 2018-10-18 9:27 | *Sample Weight:* | 1.0000 |
| *Run Time (min):* | 19.73 | *Sample Amount:* | 1.0000 |

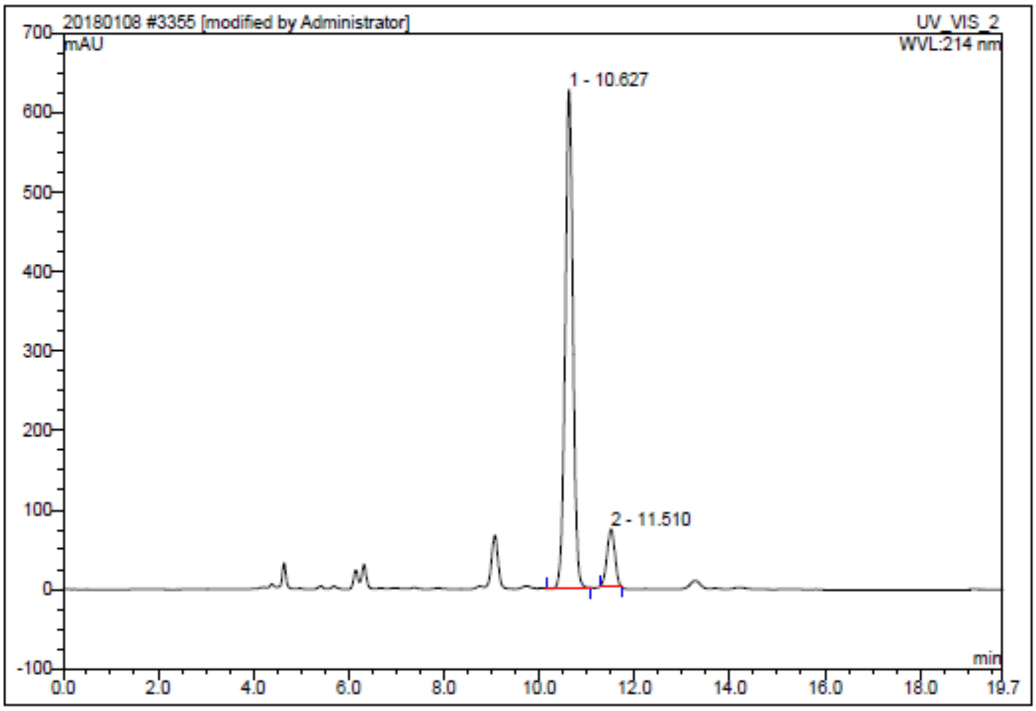


| No. | Ret.Time min | Peak Name | Height mAU | Area mAU*min | Rel.Area % | Amount | Type |
|---|---|---|---|---|---|---|---|
| 1 | 10.63 | n.a. | 627.221 | 118.677 | 89.57 | n.a. | BMB* |
| 2 | 11.51 | n.a. | 72.508 | 13.817 | 10.43 | n.a. | MB* |
| Total: | | | 699.729 | 132.493 | 100.00 | 0.000 | |

**Supplementary Figure 76.** HPLC chromatogram for compound **4d**

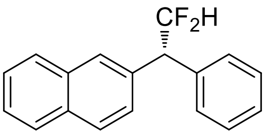


Compound **4e**. HPLC (IC, 0.46 × 25 cm, 5 µm, hexane/isopropanol = 99/1 (v/v %), flow 0.7 mL/min, UV detection at 214 nm), retention time = 8.63 min (minor) and 9.21 min (major). $[\alpha]_D^{25}$ = -32 (c = 0.1200, $CHCl_3$, 91:9 e.r.).

**7314 HWC-11-37-3B IC 991 214 0.7**

| | | | |
|---|---|---|---|
| *Sample Name:* | **HWC-11-37-3B IC 991 214 0.7** | *Injection Volume:* | **2.0** |
| *Vial Number:* | **GD2** | *Channel:* | **UV_VIS_1** |
| *Sample Type:* | **unknown** | *Wavelength:* | **214** |
| *Control Program:* | **201701-3** | *Bandwidth:* | **n.a.** |
| *Quantif. Method:* | **201701** | *Dilution Factor:* | **1.0000** |
| *Recording Time:* | **2018/8/30 14:00** | *Sample Weight:* | **1.0000** |
| *Run Time (min):* | **16.77** | *Sample Amount:* | **1.0000** |

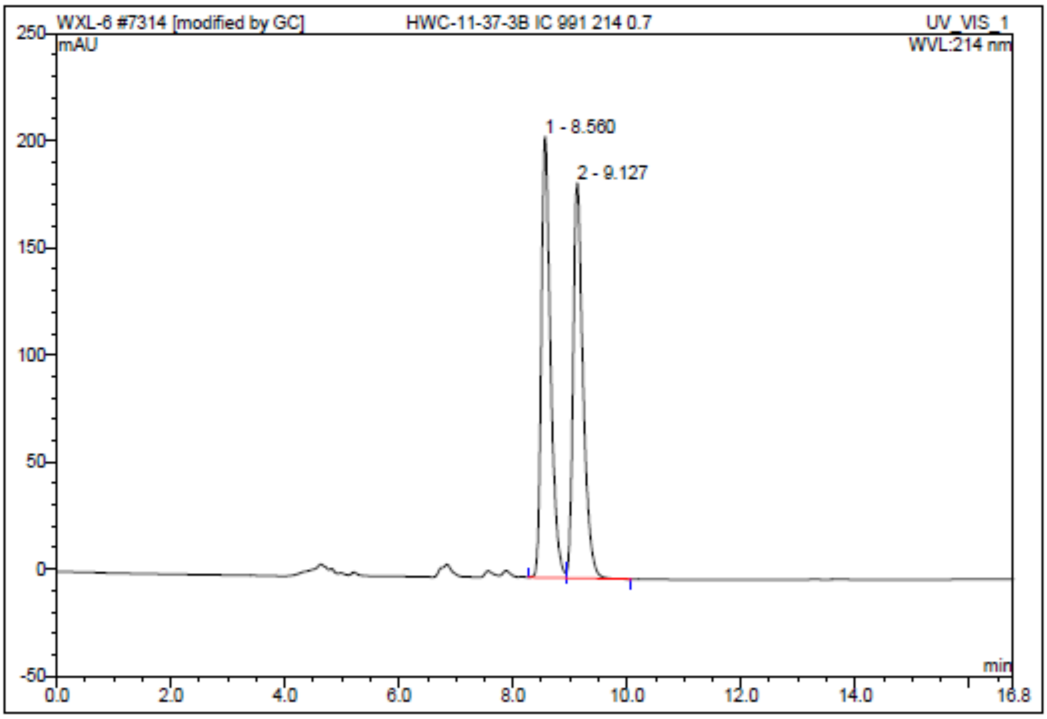


| No. | Ret.Time min | Peak Name | Height mAU | Area mAU*min | Rel.Area % | Amount | Type |
|---|---|---|---|---|---|---|---|
| 1 | 8.56 | n.a. | 205.813 | 38.654 | 50.76 | n.a. | BM |
| 2 | 9.13 | n.a. | 184.560 | 37.492 | 49.24 | n.a. | MB |
| **Total:** | | | 390.373 | 76.146 | 100.00 | 0.000 | |

**Supplementary Figure 77.** HPLC chromatogram for compound **4e**, racemic

**7315 HWC-11-37-3A IC 991 214 0.7**

| | | | |
|---|---|---|---|
| *Sample Name:* | **HWC-11-37-3A IC 991 214 0.7** | *Injection Volume:* | **2.0** |
| *Vial Number:* | **GD3** | *Channel:* | **UV_VIS_1** |
| *Sample Type:* | **unknown** | *Wavelength:* | **214** |
| *Control Program:* | **201701-3** | *Bandwidth:* | **n.a.** |
| *Quantif. Method:* | **201701** | *Dilution Factor:* | **1.0000** |
| *Recording Time:* | **2018/8/30 12:28** | *Sample Weight:* | **1.0000** |
| *Run Time (min):* | **16.00** | *Sample Amount:* | **1.0000** |

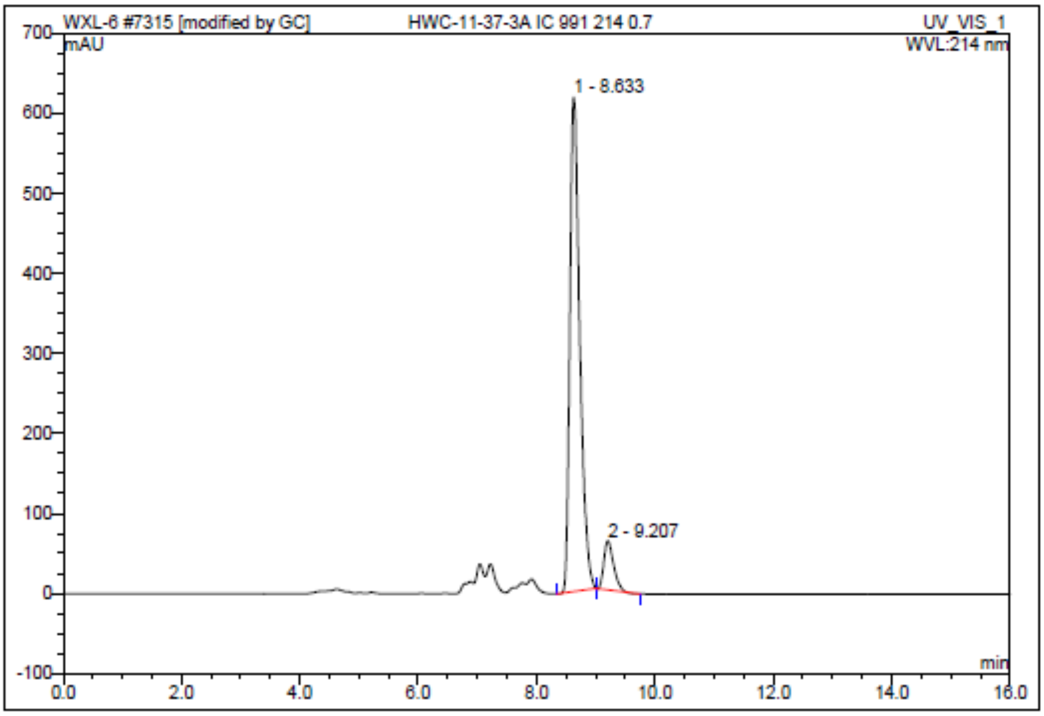


| No. | Ret.Time min | Peak Name | Height mAU | Area mAU*min | Rel.Area % | Amount | Type |
|---|---|---|---|---|---|---|---|
| 1 | 8.63 | n.a. | 617.579 | 120.077 | 91.20 | n.a. | BMb* |
| 2 | 9.21 | n.a. | 61.335 | 11.588 | 8.80 | n.a. | bMB* |
| Total: | | | 678.913 | 131.665 | 100.00 | 0.000 | |

**Supplementary Figure 78.** HPLC chromatogram for compound **4e**

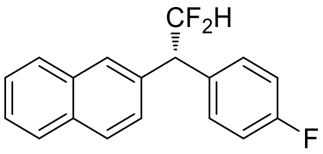

Compound **4f**. HPLC (IG, 0.46 × 25 cm, 5 μm, hexane/isopropanol = 97/3 (v/v %), flow 0.7 mL/min, UV detection at 214 nm), retention time = 7.55 min (minor) and 7.89 min (major). $[\alpha]_D^{25}$ = -49.20 (c = 0.1500, $CHCl_3$, 90:10 e.r.).

**3367 HWC-12-7-3 IG 973 214 0.7**

| | | | |
|---|---|---|---|
| *Sample Name:* | HWC-12-7-3 IG 973 214 0.7 | *Injection Volume:* | 1.0 |
| *Vial Number:* | GD6 | *Channel:* | UV_VIS_2 |
| *Sample Type:* | unknown | *Wavelength:* | 214.0 |
| *Control Program:* | test-dad2 | *Bandwidth:* | 4 |
| *Quantif. Method:* | 20170608 | *Dilution Factor:* | 1.0000 |
| *Recording Time:* | 2018-10-19 14:04 | *Sample Weight:* | 1.0000 |
| *Run Time (min):* | 30.00 | *Sample Amount:* | 1.0000 |

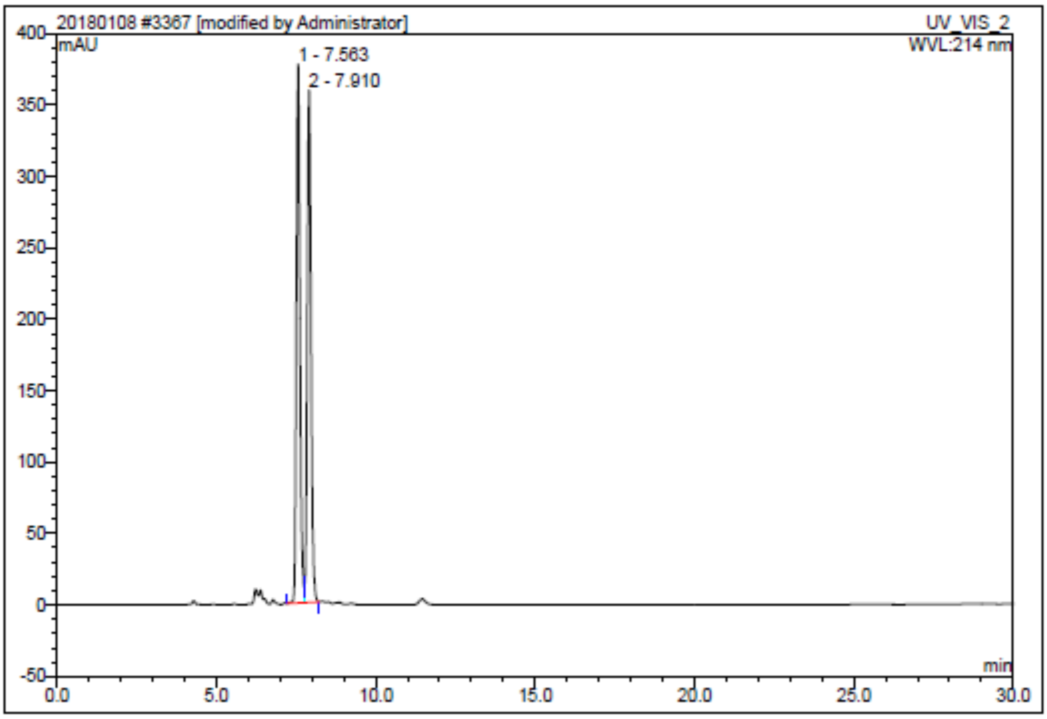


| No. | Ret.Time min | Peak Name | Height mAU | Area mAU*min | Rel.Area % | Amount | Type |
|---|---|---|---|---|---|---|---|
| 1 | 7.56 | n.a. | 377.133 | 48.699 | 49.87 | n.a. | BM |
| 2 | 7.91 | n.a. | 358.575 | 48.955 | 50.13 | n.a. | MB |
| Total: | | | 735.708 | 97.654 | 100.00 | 0.000 | |

**Supplementary Figure 79.** HPLC chromatogram for compound **4f**, racemic

**3368 HWC-12-3-3A IG 973 214 0.7**

| | | | |
|---|---|---|---|
| *Sample Name:* | **HWC-12-3-3A IG 973 214 0.7** | *Injection Volume:* | **1.0** |
| *Vial Number:* | **GC6** | *Channel:* | **UV_VIS_2** |
| *Sample Type:* | **unknown** | *Wavelength:* | **214.0** |
| *Control Program:* | **test-dad2** | *Bandwidth:* | **4** |
| *Quantif. Method:* | **20170608** | *Dilution Factor:* | **1.0000** |
| *Recording Time:* | **2018-10-19 14:34** | *Sample Weight:* | **1.0000** |
| *Run Time (min):* | **20.50** | *Sample Amount:* | **1.0000** |

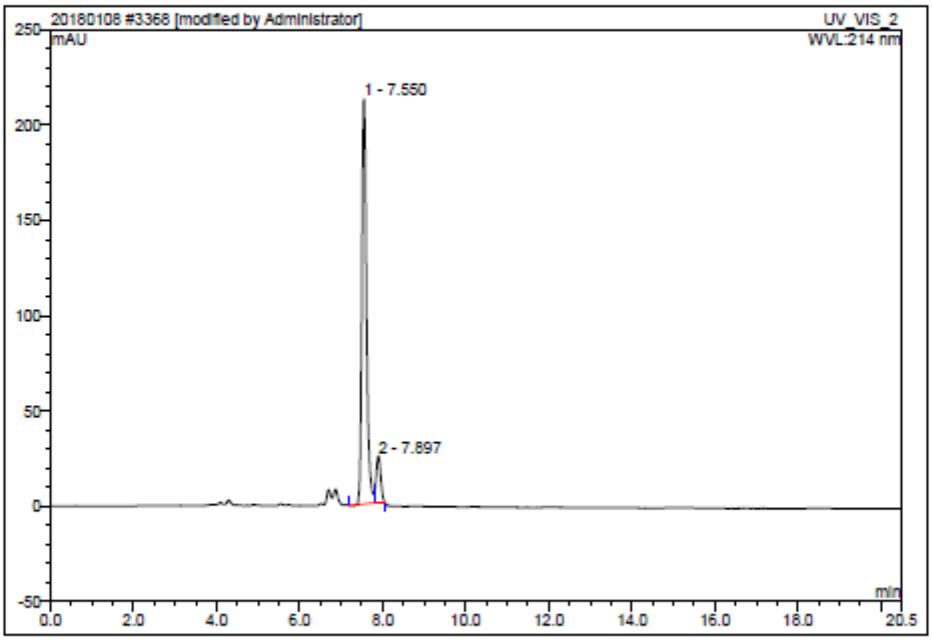


| No. | Ret.Time min | Peak Name | Height mAU | Area mAU*min | Rel.Area % | Amount | Type |
|---|---|---|---|---|---|---|---|
| 1 | 7.55 | n.a. | 212.561 | 27.640 | 89.74 | n.a. | BM * |
| 2 | 7.90 | n.a. | 24.716 | 3.161 | 10.26 | n.a. | MB* |
| Total: | | | 237.277 | 30.801 | 100.00 | 0.000 | |

**Supplementary Figure 80.** HPLC chromatogram for compound **4f**

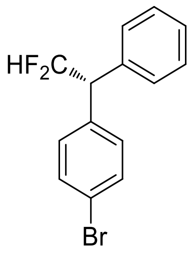


Compound **4g**. HPLC (IB, 0.46 × 25 cm, 5 μm, hexane/isopropanol = 98/2 (v/v %), flow 0.7 mL/min, UV detection at 214 nm), retention time = 7.90 min (minor) and 8.46 min (major). $[\alpha]_D^{25}$ = 16.92 (c = 0.0650, $CHCl_3$, 90:10 e.r.).

**2900 HWC-11-40-3C+- IB 982 214 0.7**

| | | | |
|---|---|---|---|
| *Sample Name:* | HWC-11-40-3C+- IB 982 214 0.7 | *Injection Volume:* | 2.0 |
| *Vial Number:* | GC7 | *Channel:* | UV_VIS_2 |
| *Sample Type:* | unknown | *Wavelength:* | 214.0 |
| *Control Program:* | test-dad3 | *Bandwidth:* | 4 |
| *Quantif. Method:* | 20170608 | *Dilution Factor:* | 1.0000 |
| *Recording Time:* | 2018-8-28 16:45 | *Sample Weight:* | 1.0000 |
| *Run Time (min):* | 9.54 | *Sample Amount:* | 1.0000 |

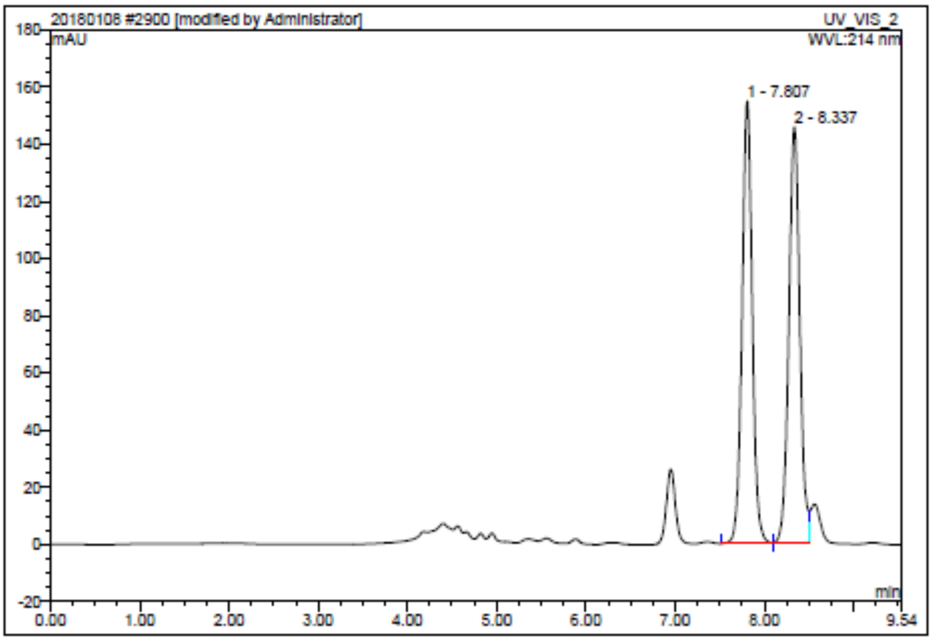


| No. | Ret.Time min | Peak Name | Height mAU | Area mAU*min | Rel.Area % | Amount | Type |
|---|---|---|---|---|---|---|---|
| 1 | 7.81 | n.a. | 154.916 | 20.559 | 49.64 | n.a. | BM * |
| 2 | 8.34 | n.a. | 145.801 | 20.858 | 50.36 | n.a. | M * |
| Total: | | | 300.716 | 41.417 | 100.00 | 0.000 | |

**Supplementary Figure 81.** HPLC chromatogram for compound **4g**, racemic

## 2901 HWC-11-39-3C IB 982 214 0.7

| | | | |
|---|---|---|---|
| *Sample Name:* | HWC-11-39-3C IB 982 214 0.7 | *Injection Volume:* | 2.0 |
| *Vial Number:* | GC8 | *Channel:* | UV_VIS_2 |
| *Sample Type:* | unknown | *Wavelength:* | 214.0 |
| *Control Program:* | test-dad3 | *Bandwidth:* | 4 |
| *Quantif. Method:* | 20170608 | *Dilution Factor:* | 1.0000 |
| *Recording Time:* | 2018-8-28 16:56 | *Sample Weight:* | 1.0000 |
| *Run Time (min):* | 12.06 | *Sample Amount:* | 1.0000 |

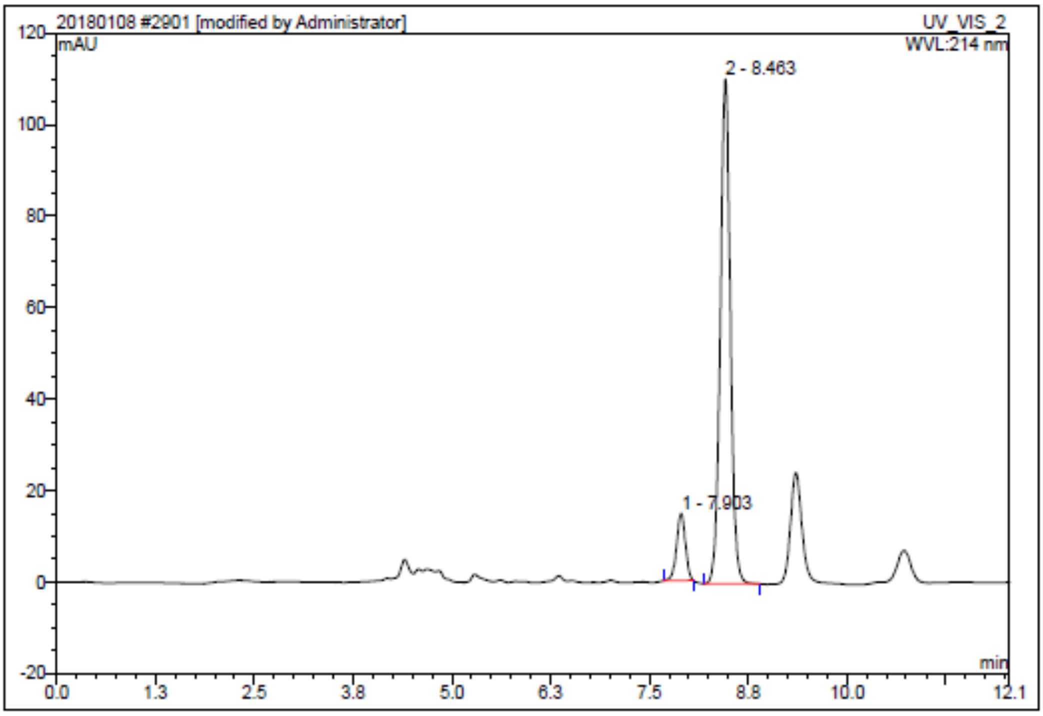


| No. | Ret.Time min | Peak Name | Height mAU | Area mAU*min | Rel.Area % | Amount | Type |
|---|---|---|---|---|---|---|---|
| 1 | 7.90 | n.a. | 14.717 | 1.907 | 10.49 | n.a. | BMB* |
| 2 | 8.46 | n.a. | 110.338 | 16.279 | 89.51 | n.a. | BMB* |
| Total: | | | 125.054 | 18.185 | 100.00 | 0.000 | |

**Supplementary Figure 82.** HPLC chromatogram for compound **4g**

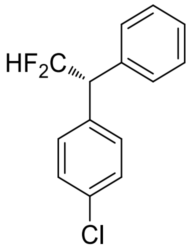


Compound **4h**. HPLC (IB, 0.46 × 25 cm, 5 μm, hexane/isopropanol = 98/2 (v/v %), flow 0.7 mL/min, UV detection at 214 nm), retention time = 7.34 min (minor) and 7.84 min (major). $[\alpha]_D^{25}$ = 4.47 (c = 0.2150, $CHCl_3$, 89:11 e.r.).

**2898 HWC-11-40-3B IB 982 214 0.7**

| | | | |
|---|---|---|---|
| *Sample Name:* | HWC-11-40-3B IB 982 214 0.7 | *Injection Volume:* | 2.0 |
| *Vial Number:* | GB7 | *Channel:* | UV_VIS_2 |
| *Sample Type:* | unknown | *Wavelength:* | 214.0 |
| *Control Program:* | test-dad3 | *Bandwidth:* | 4 |
| *Quantif. Method:* | 20170608 | *Dilution Factor:* | 1.0000 |
| *Recording Time:* | 2018-8-28 16:17 | *Sample Weight:* | 1.0000 |
| *Run Time (min):* | 10.78 | *Sample Amount:* | 1.0000 |

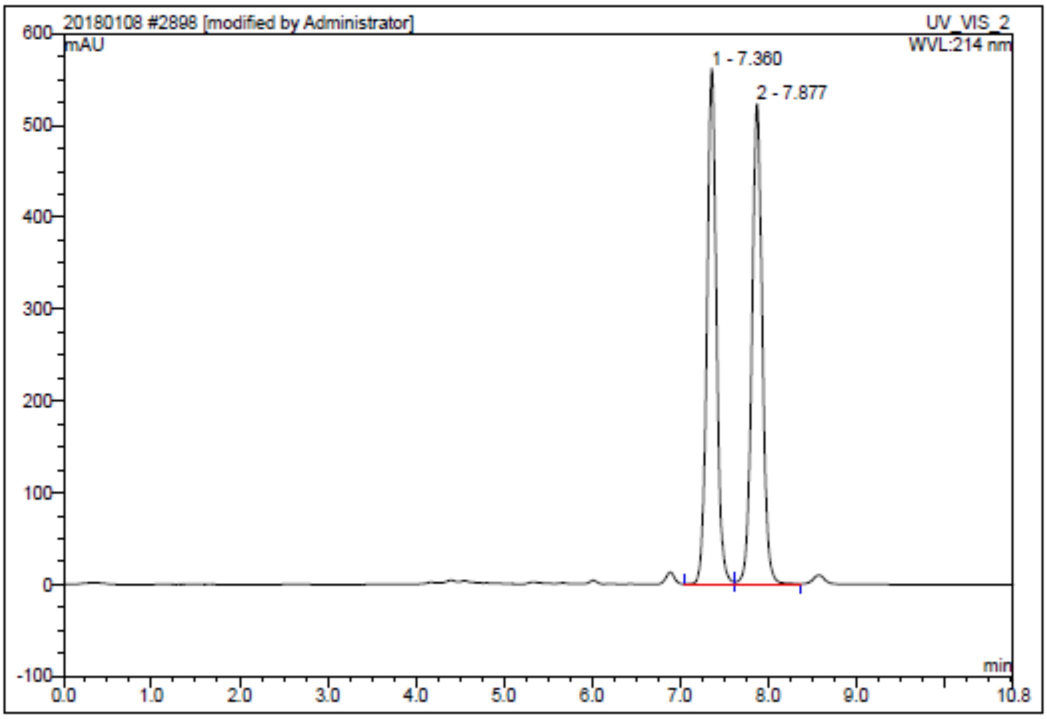


| No. | Ret.Time min | Peak Name | Height mAU | Area mAU*min | Rel.Area % | Amount | Type |
|---|---|---|---|---|---|---|---|
| 1 | 7.36 | n.a. | 561.013 | 70.750 | 49.80 | n.a. | BM * |
| 2 | 7.88 | n.a. | 522.786 | 71.305 | 50.20 | n.a. | MB* |
| Total: | | | 1083.799 | 142.054 | 100.00 | 0.000 | |

**Supplementary Figure 83.** HPLC chromatogram for compound **4h**, racemic

**2899 HWC-11-39-3B IB 982 214 0.7**

| | | | |
|---|---|---|---|
| *Sample Name:* | HWC-11-39-3B IB 982 214 0.7 | *Injection Volume:* | 2.0 |
| *Vial Number:* | GB8 | *Channel:* | UV_VIS_2 |
| *Sample Type:* | unknown | *Wavelength:* | 214.0 |
| *Control Program:* | test-dad3 | *Bandwidth:* | 4 |
| *Quantif. Method:* | 20170608 | *Dilution Factor:* | 1.0000 |
| *Recording Time:* | 2018-8-28 16:29 | *Sample Weight:* | 1.0000 |
| *Run Time (min):* | 13.71 | *Sample Amount:* | 1.0000 |

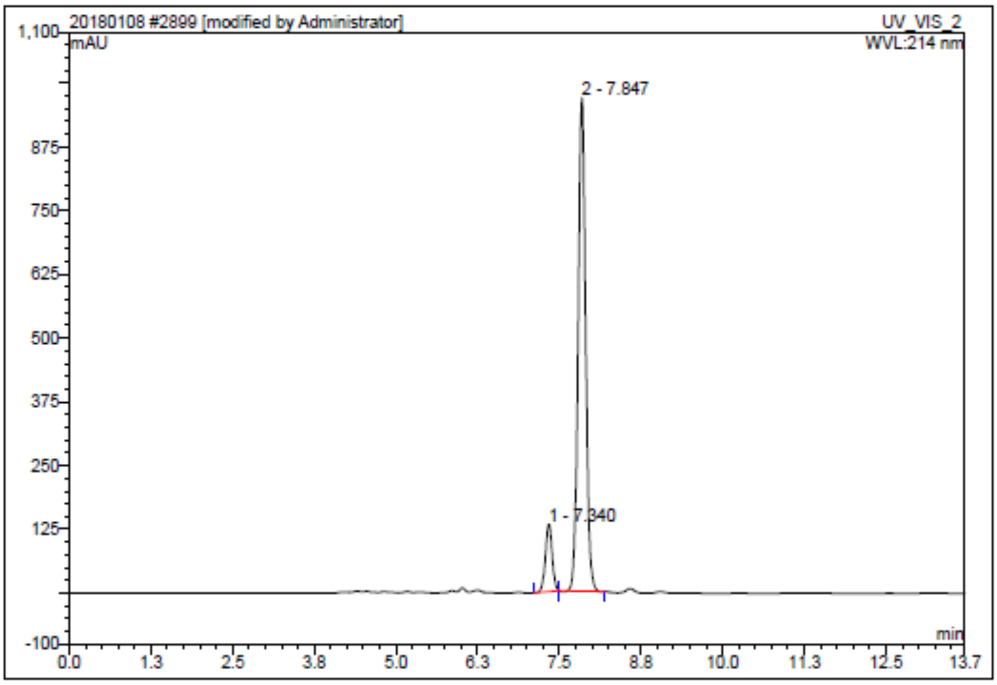


| No. | Ret.Time min | Peak Name | Height mAU | Area mAU*min | Rel.Area % | Amount | Type |
|---|---|---|---|---|---|---|---|
| 1 | 7.34 | n.a. | 131.543 | 15.788 | 10.95 | n.a. | BMb* |
| 2 | 7.85 | n.a. | 967.052 | 128.407 | 89.05 | n.a. | bMB* |
| Total: | | | 1098.595 | 144.195 | 100.00 | 0.000 | |

**Supplementary Figure 84.** HPLC chromatogram for compound **4h**

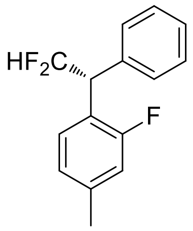


Compound **4i**. HPLC (IA, 0.46 × 25 cm, 5 µm, hexane/isopropanol = 100/0 (v/v %), flow 0.7 mL/min, UV detection at 214 nm), retention time = 11.67 min (minor) and 12.44 min (major). $[\alpha]_D^{25}$ = -9.67 (c = 0.2750, $CHCl_3$, 90:10 e.r.).

**3044 HWC-11-69-3B+- IA 1 214 0.7**

| | | | |
|---|---|---|---|
| *Sample Name:* | **HWC-11-69-3B+- IA 1 214 0.7** | *Injection Volume:* | **2.0** |
| *Vial Number:* | **GE6** | *Channel:* | **UV_VIS_2** |
| *Sample Type:* | **unknown** | *Wavelength:* | **214.0** |
| *Control Program:* | **test-dad3** | *Bandwidth:* | **4** |
| *Quantif. Method:* | **20170608** | *Dilution Factor:* | **1.0000** |
| *Recording Time:* | **2018-9-11 18:38** | *Sample Weight:* | **1.0000** |
| *Run Time (min):* | **40.00** | *Sample Amount:* | **1.0000** |

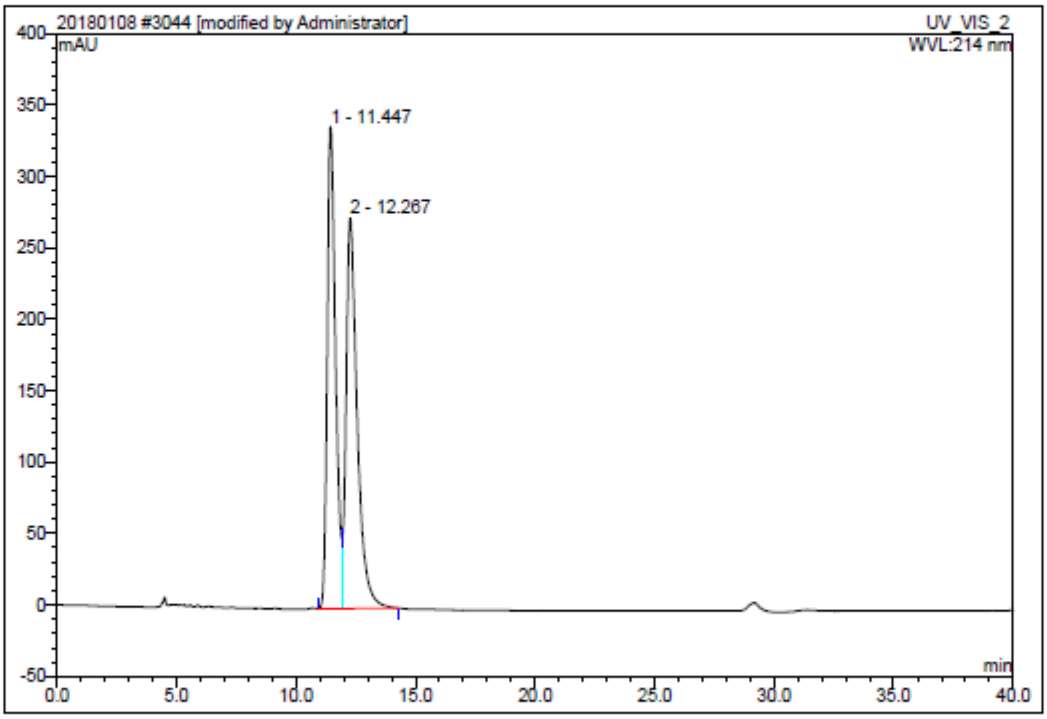


| No. | Ret.Time min | Peak Name | Height mAU | Area mAU*min | Rel.Area % | Amount | Type |
|---|---|---|---|---|---|---|---|
| 1 | 11.45 | n.a. | 337.518 | 135.207 | 47.76 | n.a. | BM |
| 2 | 12.27 | n.a. | 273.186 | 147.875 | 52.24 | n.a. | MB |
| **Total:** | | | 610.704 | 283.082 | 100.00 | 0.000 | |

**Supplementary Figure 85.** HPLC chromatogram for compound **4i**, racemic

## 3045 HWC-11-69-3A IA 1 214 0.7

| | | | |
|---|---|---|---|
| *Sample Name:* | HWC-11-69-3A IA 1 214 0.7 | *Injection Volume:* | 2.0 |
| *Vial Number:* | GE7 | *Channel:* | UV_VIS_2 |
| *Sample Type:* | unknown | *Wavelength:* | 214.0 |
| *Control Program:* | test-dad3 | *Bandwidth:* | 4 |
| *Quantif. Method:* | 20170608 | *Dilution Factor:* | 1.0000 |
| *Recording Time:* | 2018-9-11 19:19 | *Sample Weight:* | 1.0000 |
| *Run Time (min):* | 40.00 | *Sample Amount:* | 1.0000 |

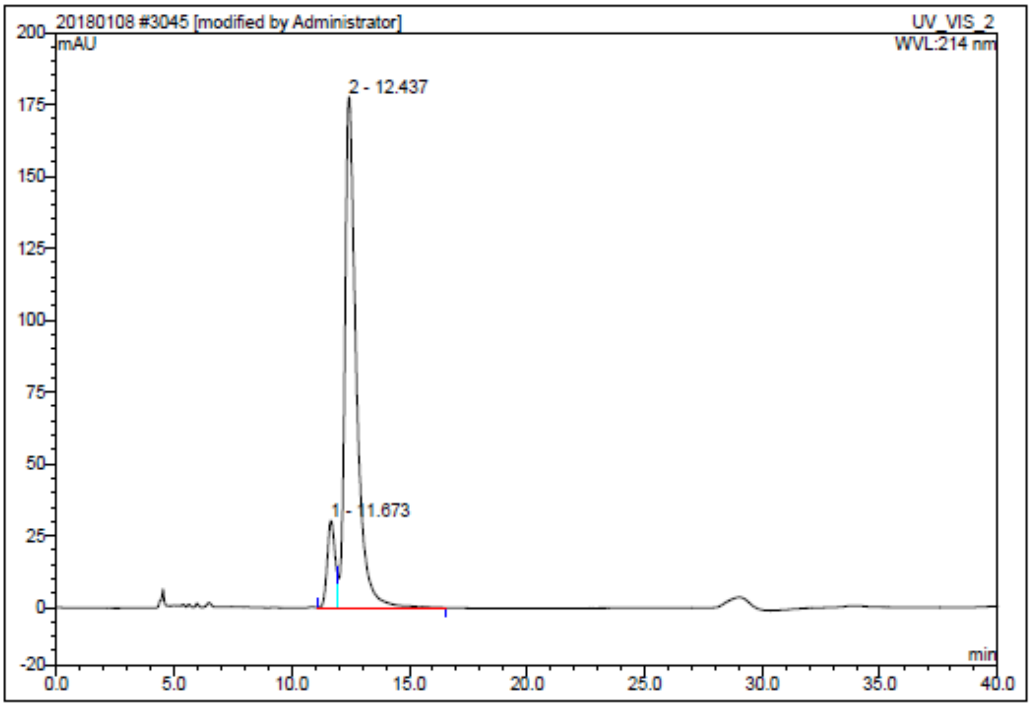


| No. | Ret.Time min | Peak Name | Height mAU | Area mAU*min | Rel.Area % | Amount | Type |
|---|---|---|---|---|---|---|---|
| 1 | 11.67 | n.a. | 30.208 | 11.268 | 10.09 | n.a. | BM * |
| 2 | 12.44 | n.a. | 177.690 | 100.369 | 89.91 | n.a. | MB* |
| Total: | | | 207.898 | 111.637 | 100.00 | 0.000 | |

**Supplementary Figure 86.** HPLC chromatogram for compound **4i**

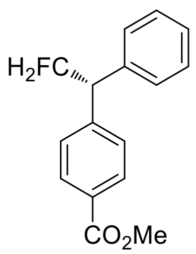


Compound **4j**. HPLC (IG, 0.46 × 25 cm, 5 μm, hexane/isopropanol = 95/5 (v/v %), flow 0.7 mL/min, UV detection at 214 nm), retention time = 16.50 min (minor) and 17.90 min (major). $[\alpha]_D^{25}$ = -6.33 (c = 0.2400, $CHCl_3$, 83:17 e.r.).

**491 HM-1-59-2E IG 955 214 0.7**

| | | | |
|---|---|---|---|
| *Sample Name:* | HM-1-59-2E IG 955 214 0.7 | *Injection Volume:* | 1.0 |
| *Vial Number:* | GE2 | *Channel:* | UV_VIS_2 |
| *Sample Type:* | unknown | *Wavelength:* | 214.0 |
| *Control Program:* | test-dad6 | *Bandwidth:* | 4 |
| *Quantif. Method:* | 20170608 | *Dilution Factor:* | 1.0000 |
| *Recording Time:* | 2018-1-23 18:53 | *Sample Weight:* | 1.0000 |
| *Run Time (min):* | 23.00 | *Sample Amount:* | 1.0000 |

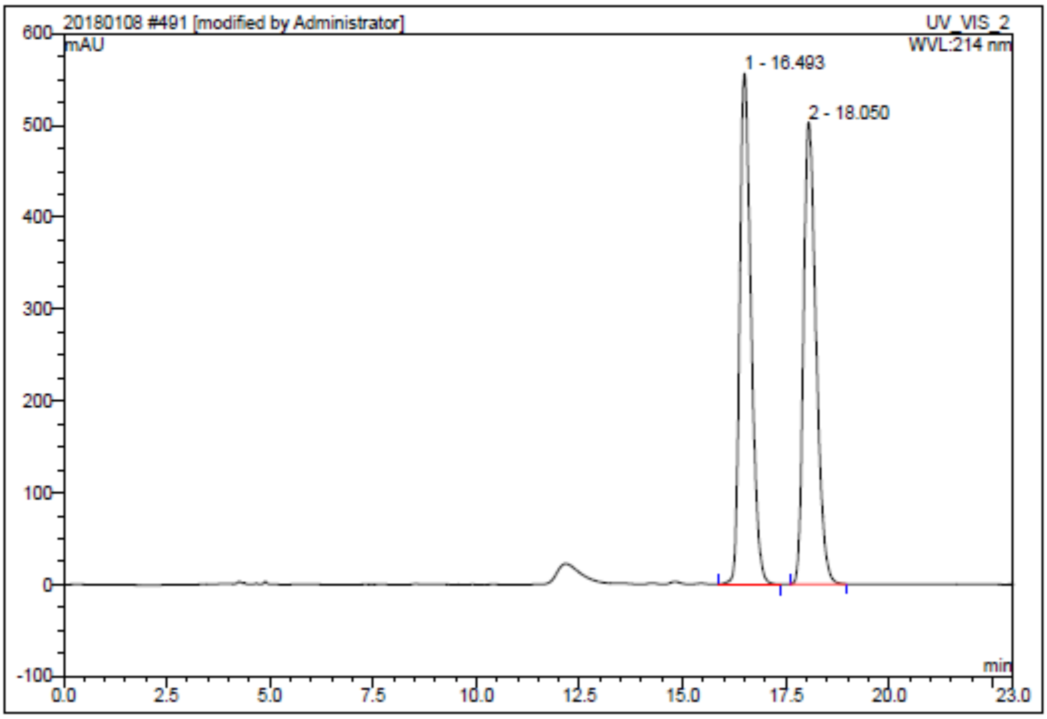


| No. | Ret.Time min | Peak Name | Height mAU | Area mAU*min | Rel.Area % | Amount | Type |
|---|---|---|---|---|---|---|---|
| 1 | 16.49 | n.a. | 555.914 | 177.482 | 50.01 | n.a. | BMB |
| 2 | 18.05 | n.a. | 503.001 | 177.439 | 49.99 | n.a. | BMB |
| Total: | | | 1058.915 | 354.921 | 100.00 | 0.000 | |

**Supplementary Figure 87.** HPLC chromatogram for compound **4j**, racemic

## 2827 HM-2-35-C IG 955 214 0.7

| | | | |
|---|---|---|---|
| *Sample Name:* | HM-2-35-C IG 955 214 0.7 | *Injection Volume:* | 2.0 |
| *Vial Number:* | GC7 | *Channel:* | UV_VIS_2 |
| *Sample Type:* | unknown | *Wavelength:* | 214.0 |
| *Control Program:* | test-dad2 | *Bandwidth:* | 4 |
| *Quantif. Method:* | 20170608 | *Dilution Factor:* | 1.0000 |
| *Recording Time:* | 2018-8-21 14:33 | *Sample Weight:* | 1.0000 |
| *Run Time (min):* | 20.00 | *Sample Amount:* | 1.0000 |

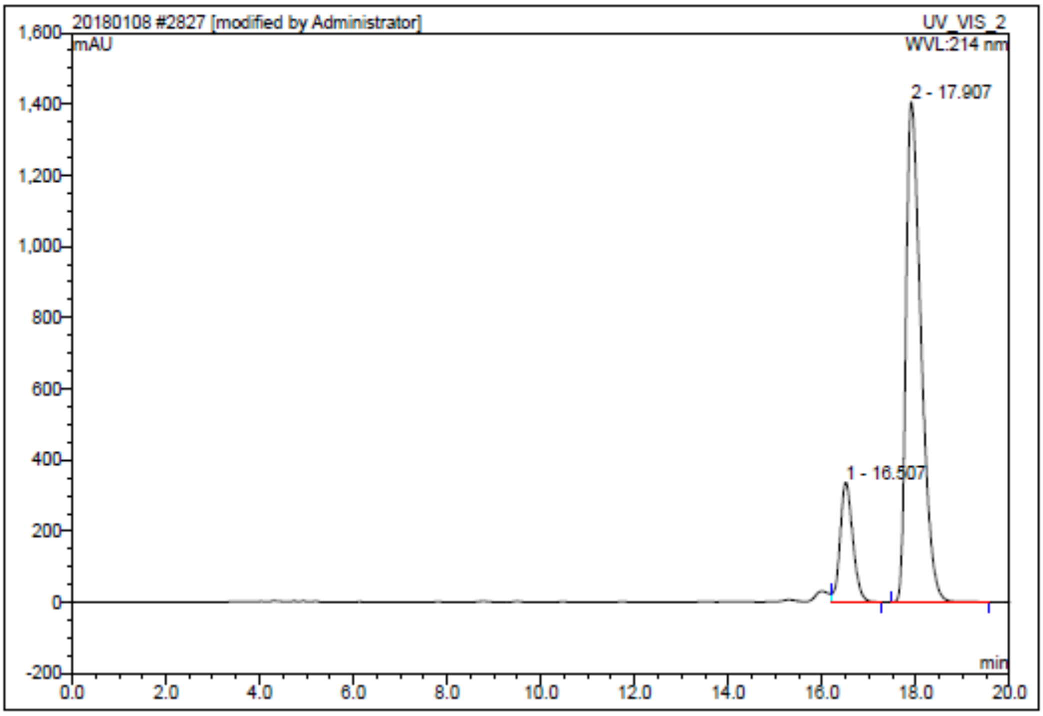


| No. | Ret.Time min | Peak Name | Height mAU | Area mAU*min | Rel.Area % | Amount | Type |
|---|---|---|---|---|---|---|---|
| 1 | 16.51 | n.a. | 336.188 | 105.803 | 16.81 | n.a. | MB* |
| 2 | 17.91 | n.a. | 1405.047 | 523.718 | 83.19 | n.a. | BMB |
| Total: | | | 1741.235 | 629.521 | 100.00 | 0.000 | |

**Supplementary Figure 88.** HPLC chromatogram for compound **4j**

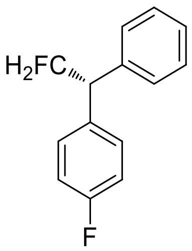


Compound **4k**. HPLC (OD-H, 0.46 × 25 cm, 5 µm, hexane/isopropanol = 95/5 (v/v %), flow 0.7 mL/min, UV detection at 214 nm), retention time = 7.62 min (minor) and 7.95 min (major). $[\alpha]_D^{25}$ = 11.67 (c = 0.0600, $CHCl_3$, 79:21 e.r.).

**7384 HM-2-29-A+- ODH 955 214 0.7**

| | | | |
|---|---|---|---|
| *Sample Name:* | **HM-2-29-A+- ODH 955 214 0.7** | *Injection Volume:* | **1.0** |
| *Vial Number:* | **GE1** | *Channel:* | **UV_VIS_1** |
| *Sample Type:* | **unknown** | *Wavelength:* | **214** |
| *Control Program:* | **201701-5** | *Bandwidth:* | **n.a.** |
| *Quantif. Method:* | **201701** | *Dilution Factor:* | **1.0000** |
| *Recording Time:* | **2018/9/11 9:14** | *Sample Weight:* | **1.0000** |
| *Run Time (min):* | **9.53** | *Sample Amount:* | **1.0000** |

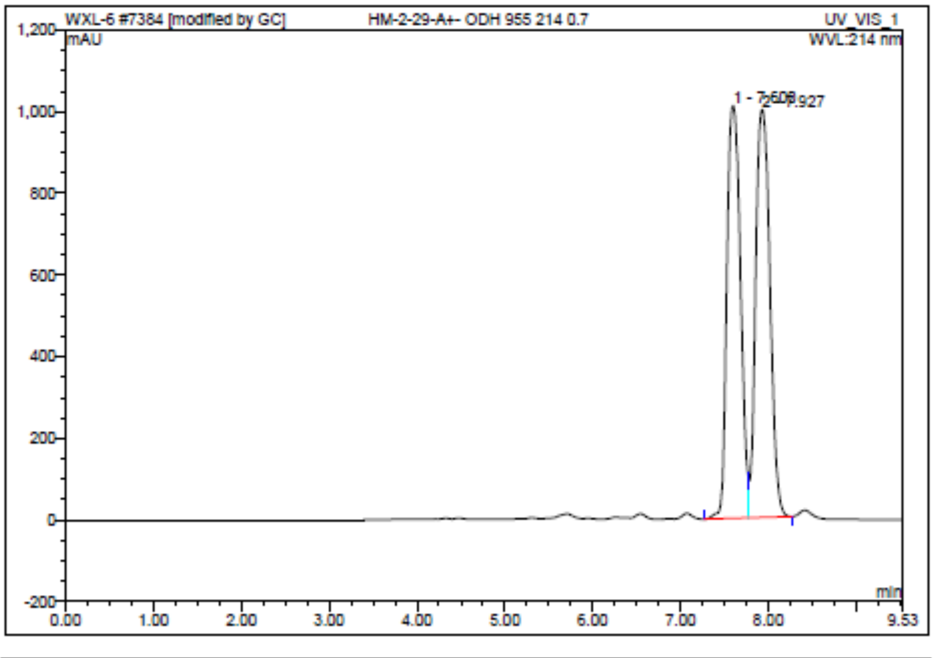


| No. | Ret.Time min | Peak Name | Height mAU | Area mAU*min | Rel.Area % | Amount | Type |
|---|---|---|---|---|---|---|---|
| 1 | 7.60 | n.a. | 1010.847 | 181.480 | 49.09 | n.a. | BM |
| 2 | 7.93 | n.a. | 998.948 | 188.181 | 50.91 | n.a. | MB* |
| Total: | | | 2009.794 | 369.662 | 100.00 | 0.000 | |

**Supplementary Figure 89.** HPLC chromatogram for compound **4k**, racemic

Operator:GC Timebase:U3000 Sequence:WXL-6 Pag
2018/12/25 10:23

**7383 HM-2-30-A ODH 955 214 0.7**

| | | | |
|---|---|---|---|
| *Sample Name:* | **HM-2-30-A ODH 955 214 0.7** | *Injection Volume:* | **1.0** |
| *Vial Number:* | **GC1** | *Channel:* | **UV_VIS_1** |
| *Sample Type:* | **unknown** | *Wavelength:* | **214** |
| *Control Program:* | **201701-5** | *Bandwidth:* | **n.a.** |
| *Quantif. Method:* | **201701** | *Dilution Factor:* | **1.0000** |
| *Recording Time:* | **2018/9/10 19:37** | *Sample Weight:* | **1.0000** |
| *Run Time (min):* | **20.00** | *Sample Amount:* | **1.0000** |

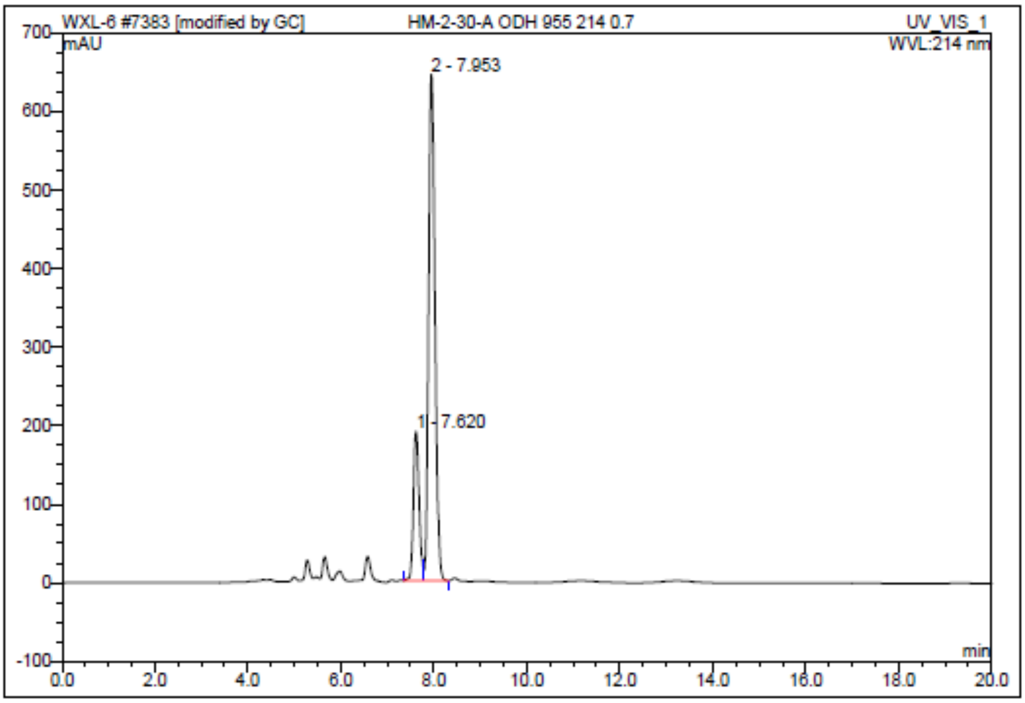


| No. | Ret.Time min | Peak Name | Height mAU | Area mAU*min | Rel.Area % | Amount | Type |
|---|---|---|---|---|---|---|---|
| 1 | 7.62 | n.a. | 189.879 | 27.119 | 20.94 | n.a. | BM |
| 2 | 7.95 | n.a. | 644.083 | 102.385 | 79.06 | n.a. | MB* |
| **Total:** | | | 833.963 | 129.504 | 100.00 | 0.000 | |

**Supplementary Figure 89.** HPLC chromatogram for compound **4k**

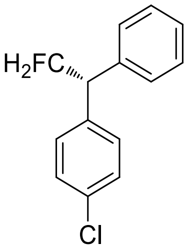


Compound **4l**. HPLC (OD-H, 0.46 × 25 cm, 5 μm, hexane/isopropanol = 95/5 (v/v %), flow 0.7 mL/min, UV detection at 214 nm), retention time = 8.03 min (minor) and 8.58 min (major). $[\alpha]_D^{25}$ = 4.89 (c = 0.0900, $CHCl_3$, 80:20 e.r.).

**7386 HM-2-29-B+- ODH 955 214 0.7**

| | | | |
|---|---|---|---|
| *Sample Name:* | HM-2-29-B+- ODH 955 214 0.7 | *Injection Volume:* | 0.8 |
| *Vial Number:* | GE2 | *Channel:* | UV_VIS_1 |
| *Sample Type:* | unknown | *Wavelength:* | 214 |
| *Control Program:* | 201701-5 | *Bandwidth:* | n.a. |
| *Quantif. Method:* | 201701 | *Dilution Factor:* | 1.0000 |
| *Recording Time:* | 2018/9/11 9:44 | *Sample Weight:* | 1.0000 |
| *Run Time (min):* | 11.30 | *Sample Amount:* | 1.0000 |

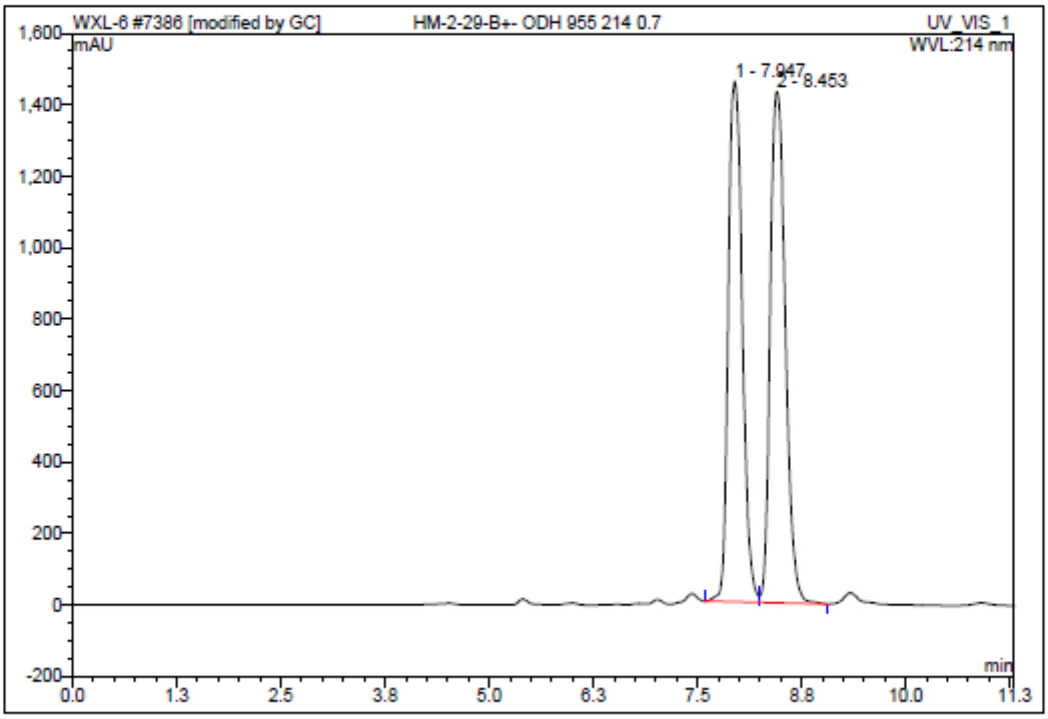


| No. | Ret.Time min | Peak Name | Height mAU | Area mAU*min | Rel.Area % | Amount | Type |
|---|---|---|---|---|---|---|---|
| 1 | 7.95 | n.a. | 1457.002 | 285.483 | 48.67 | n.a. | BM * |
| 2 | 8.45 | n.a. | 1431.912 | 301.067 | 51.33 | n.a. | MB* |
| Total: | | | 2888.914 | 586.550 | 100.00 | 0.000 | |

**Supplementary Figure 91.** HPLC chromatogram for compound **4l**, racemic

Operator:GC Timebase:U3000 Sequence:WXL-6 Page 1
2018/12/25 10:25 下

## 7385 HM-2-30-B ODH 955 214 0.7

| | | | |
|---|---|---|---|
| *Sample Name:* | **HM-2-30-B ODH 955 214 0.7** | *Injection Volume:* | **1.0** |
| *Vial Number:* | **GC2** | *Channel:* | **UV_VIS_1** |
| *Sample Type:* | **unknown** | *Wavelength:* | **214** |
| *Control Program:* | **201701-5** | *Bandwidth:* | **n.a.** |
| *Quantif. Method:* | **201701** | *Dilution Factor:* | **1.0000** |
| *Recording Time:* | **2018/9/10 19:58** | *Sample Weight:* | **1.0000** |
| *Run Time (min):* | **20.00** | *Sample Amount:* | **1.0000** |

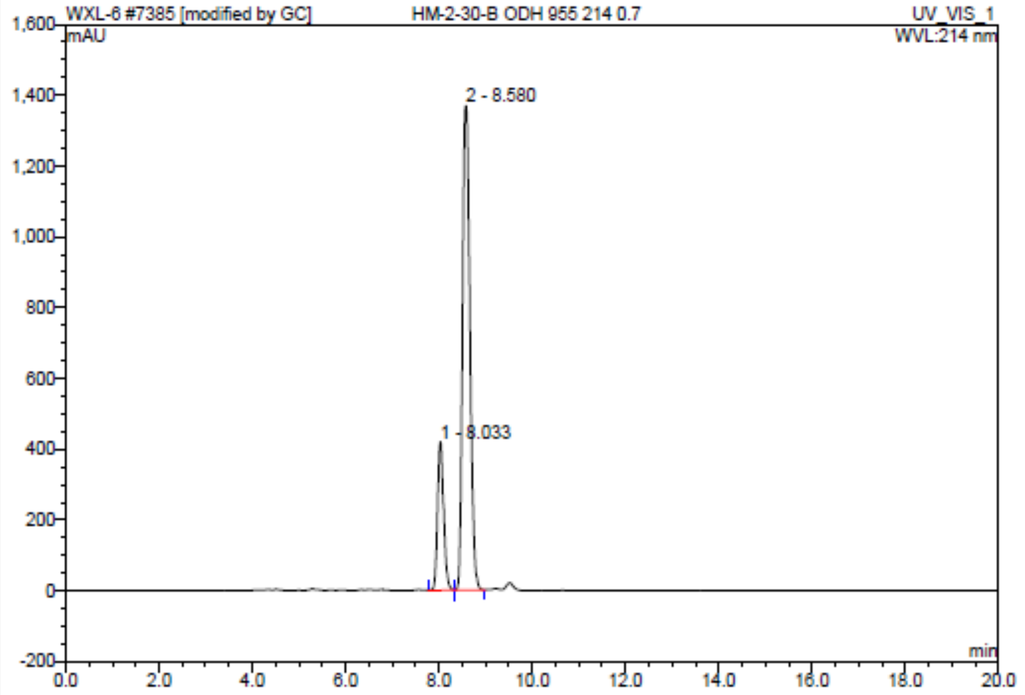


| No. | Ret.Time min | Peak Name | Height mAU | Area mAU*min | Rel.Area % | Amount | Type |
|---|---|---|---|---|---|---|---|
| 1 | 8.03 | n.a. | 419.676 | 63.647 | 20.10 | n.a. | BM * |
| 2 | 8.58 | n.a. | 1368.098 | 253.046 | 79.90 | n.a. | MB* |
| Total: | | | 1787.774 | 316.693 | 100.00 | 0.000 | |

**Supplementary Figure 92.** HPLC chromatogram for compound **4l**

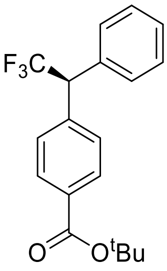


Compound **5a**. SFC (OJ-H, 0.46 × 25 cm, 5 μm, $CO_2$/MeOH = 98/2, flow 2.0 mL/min, column Temperature: 40 °C, background press: 2000 psi, UV detection at 214 nm), retention time = 2.93 min (major) and 3.38 min (minor). $[\alpha]_D^{25}$ = 37.9622 (c = 0.0600, $CHCl_3$, 95.5 :4.5 e.r.).

Empower 3 SOFTWARE

## SAMPLE INFORMATION

| | | | |
|---|---|---|---|
| Sample Name: | HWC-9-106-3B OJH 9822142200040 | Acquired By: | System |
| Sample Type: | | Date Acquired: | 2018/4/20 14:03:04 CST |
| Vial: | 1:A,2 | Acq. Method Set: | chiral_isocratic |
| Injection: | 1 | Date Processed: | 2018/5/28 9:10:02 CST |
| Injection Volume: | 1.00 ul | Processing Method | 1 |
| Run Time: | 6.0 Minutes | Channel Name: | PDA Ch1 214 nm@1.2 nm |
| Sample Set Name | 20180323 | Proc. Chnl. Descr: | PDA Ch1 214 nm@1.2 nm |

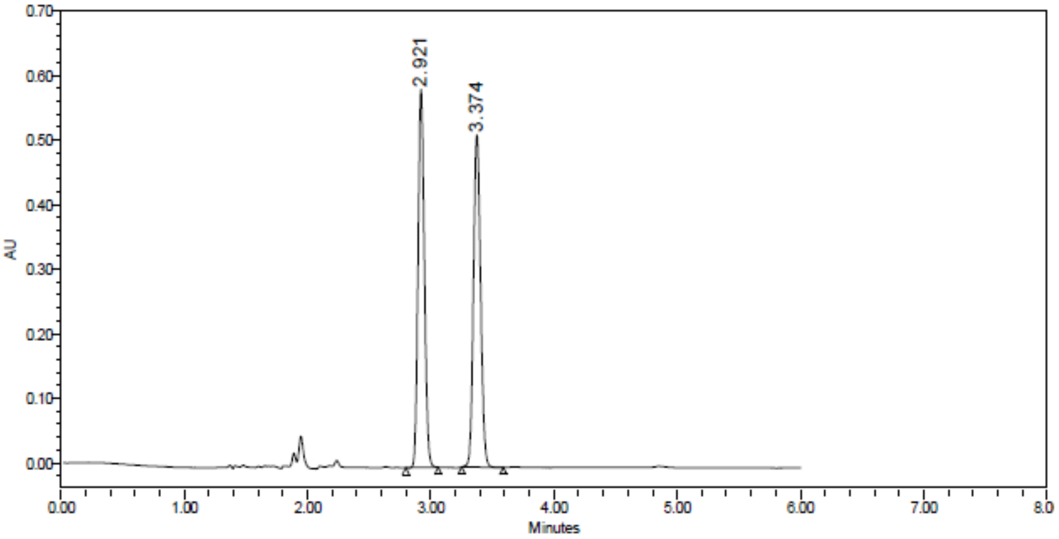


Peak Results

| | RT | Area | Height | % Area |
|---|---|---|---|---|
| 1 | 2.921 | 2086072 | 584924 | 50.29 |
| 2 | 3.374 | 2061820 | 513959 | 49.71 |

**Supplementary Figure 93.** HPLC chromatogram for compound **5a**, racemic

SAMPLE INFORMATION

| | | | |
|---|---|---|---|
| Sample Name: | HWC-9-106-3A OJH 9822142200040 | Acquired By: | System |
| Sample Type: | | Date Acquired: | 2018/4/20 13:54:35 CST |
| Vial: | 1:A,1 | Acq. Method Set: | chiral_isocratic |
| Injection: | 1 | Date Processed: | 2018/5/28 9:11:42 CST |
| Injection Volume: | 3.00 ul | Processing Method | 1 |
| Run Time: | 6.0 Minutes | Channel Name: | PDA Ch1 214 nm@1.2 nm |
| Sample Set Name | 20180323 | Proc. Chnl. Descr: | PDA Ch1 214 nm@1.2 nm |

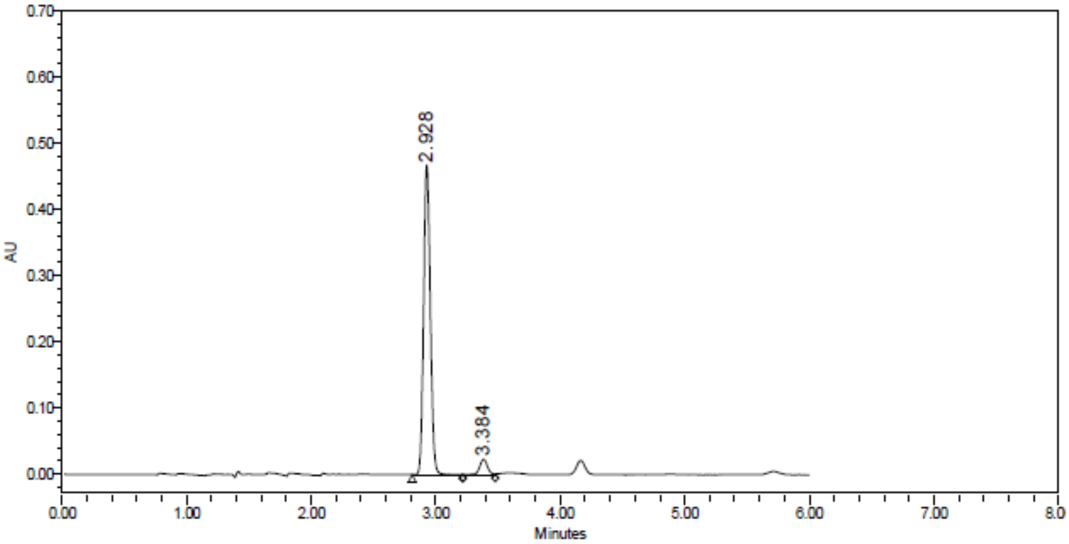


Peak Results

| | RT | Area | Height | % Area |
|---|---|---|---|---|
| 1 | 2.928 | 1740048 | 467241 | 94.57 |
| 2 | 3.384 | 99923 | 23182 | 5.43 |

**Supplementary Figure 94.** HPLC chromatogram for compound **5a**

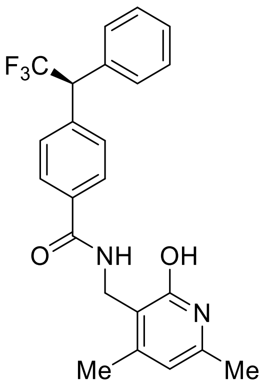


Compound **5**. HPLC (IG, 0.46 × 25 cm, 5 μm, hexane/isopropanol = 80/20 (v/v %), flow 0.7 mL/min, UV detection at 214 nm), retention time = 22.45 min (minor) and 28.47 min (major). $[\alpha]_D^{25}$ = 6.9710 (c = 0.2000, $CHCl_3$, 94:6 e.r.).

**1892 HWC-9-92-3 IG 82 214 0.7**

| | | | |
|---|---|---|---|
| *Sample Name:* | HWC-9-92-3 IG 82 214 0.7 | *Injection Volume:* | 5.0 |
| *Vial Number:* | BE7 | *Channel:* | UV_VIS_2 |
| *Sample Type:* | unknown | *Wavelength:* | 214.0 |
| *Control Program:* | test-dad2 | *Bandwidth:* | 4 |
| *Quantif. Method:* | 20170608 | *Dilution Factor:* | 1.0000 |
| *Recording Time:* | 2018-5-7 14:27 | *Sample Weight:* | 1.0000 |
| *Run Time (min):* | 50.00 | *Sample Amount:* | 1.0000 |

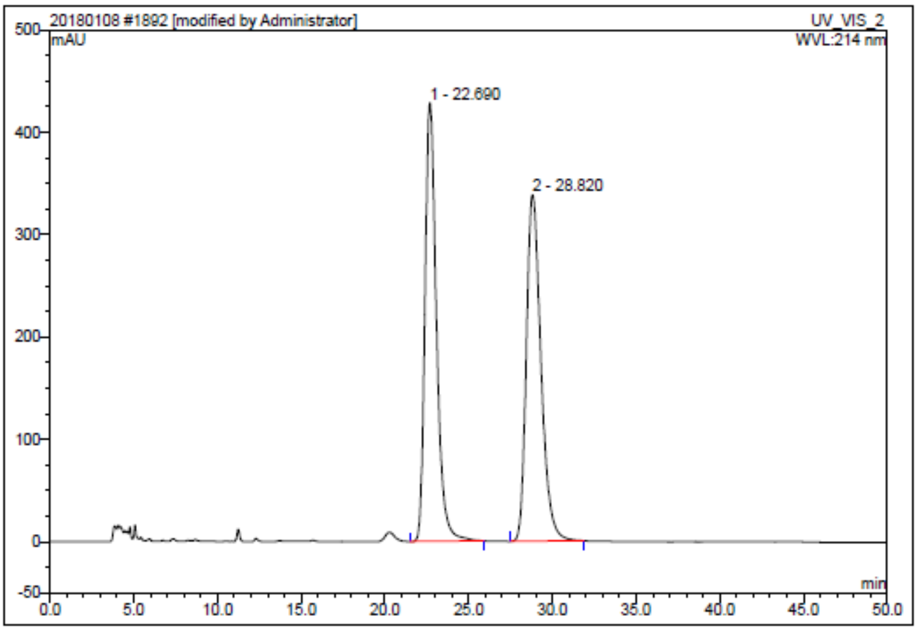


| No. | Ret.Time min | Peak Name | Height mAU | Area mAU*min | Rel.Area % | Amount | Type |
|---|---|---|---|---|---|---|---|
| 1 | 22.69 | n.a. | 428.557 | 339.951 | 49.88 | n.a. | BMB* |
| 2 | 28.82 | n.a. | 338.284 | 341.652 | 50.12 | n.a. | BMB |
| Total: | | | 766.840 | 681.603 | 100.00 | 0.000 | |

**Supplementary Figure 95.** HPLC chromatogram for compound **5**, racemic

**1889 HWC-10-14-31 IG 82 214 0.7**

| | | | |
|---|---|---|---|
| *Sample Name:* | **HWC-10-14-31 IG 82 214 0.7** | *Injection Volume:* | **5.0** |
| *Vial Number:* | **BC6** | *Channel:* | **UV_VIS_2** |
| *Sample Type:* | **unknown** | *Wavelength:* | **214.0** |
| *Control Program:* | **test-dad2** | *Bandwidth:* | **4** |
| *Quantif. Method:* | **20170608** | *Dilution Factor:* | **1.0000** |
| *Recording Time:* | **2018-5-7 11:54** | *Sample Weight:* | **1.0000** |
| *Run Time (min):* | **50.00** | *Sample Amount:* | **1.0000** |

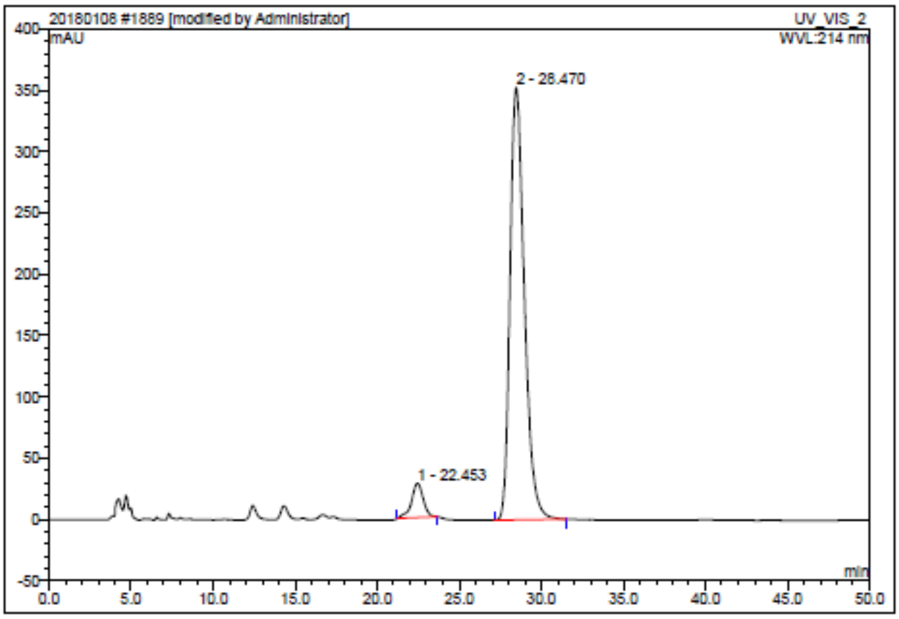


| No. | Ret.Time min | Peak Name | Height mAU | Area mAU*min | Rel.Area % | Amount | Type |
|---|---|---|---|---|---|---|---|
| 1 | 22.45 | n.a. | 27.932 | 23.138 | 6.09 | n.a. | BMB* |
| 2 | 28.47 | n.a. | 352.528 | 357.096 | 93.91 | n.a. | BMB |
| **Total:** | | | 380.460 | 380.235 | 100.00 | 0.000 | |

**Supplementary Figure 96.** HPLC chromatogram for compound **5**

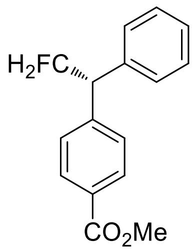


Compound **6a**. HPLC (IB, 0.46 × 25 cm, 5 μm, hexane/isopropanol = 95/5 (v/v %), flow 0.7 mL/min, UV detection at 214 nm), retention time = 9.41 min (minor) and 9.91 min (major). $[\alpha]_D^{25}$ = -6.25 (c = 0.128, $CHCl_3$, 91:9 e.r.).

**7702 HWC-12-19-3+- IB 955 214 0.7**

| | | | |
|---|---|---|---|
| *Sample Name:* | HWC-12-19-3+- IB 955 214 0.7 | *Injection Volume:* | 3.0 |
| *Vial Number:* | GB1 | *Channel:* | UV_VIS_1 |
| *Sample Type:* | unknown | *Wavelength:* | 214 |
| *Control Program:* | 201701-4 | *Bandwidth:* | n.a. |
| *Quantif. Method:* | 201701 | *Dilution Factor:* | 1.0000 |
| *Recording Time:* | 2018/10/29 10:08 | *Sample Weight:* | 1.0000 |
| *Run Time (min):* | 12.31 | *Sample Amount:* | 1.0000 |

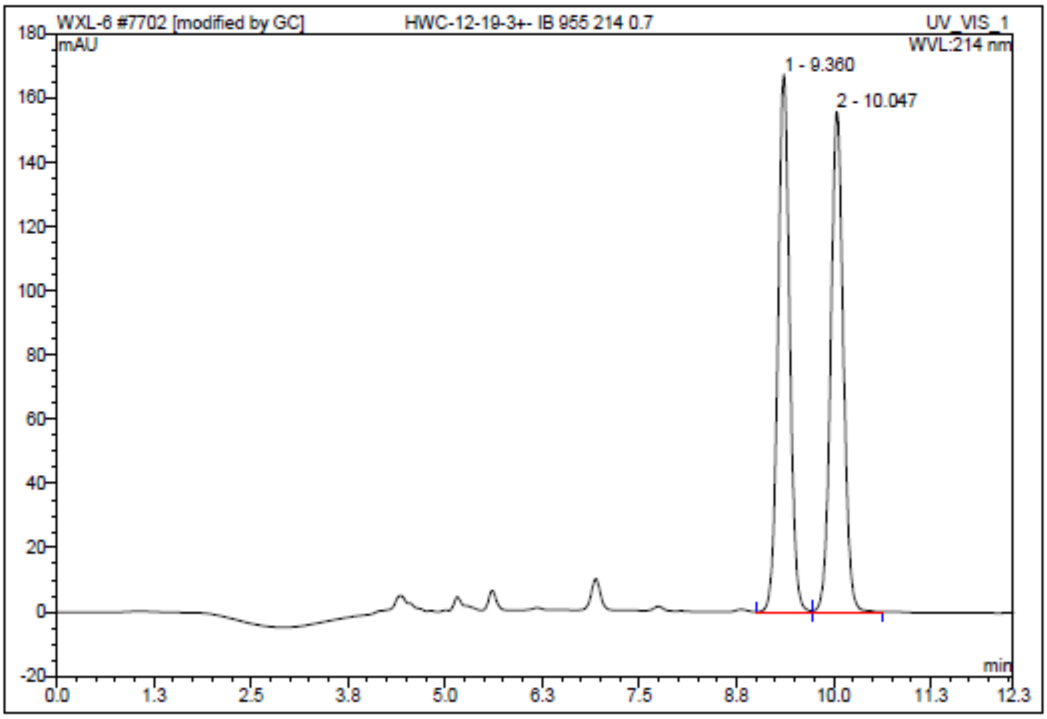


| No. | Ret.Time min | Peak Name | Height mAU | Area mAU*min | Rel.Area % | Amount | Type |
|---|---|---|---|---|---|---|---|
| 1 | 9.36 | n.a. | 167.153 | 29.303 | 49.94 | n.a. | BM |
| 2 | 10.05 | n.a. | 155.548 | 29.374 | 50.06 | n.a. | MB |
| Total: | | | 322.701 | 58.677 | 100.00 | 0.000 | |

**Supplementary Figure 97.** HPLC chromatogram for compound **6a**, racemic

**6911 HWC-10-75-3C IB 955 214 0.7**

| | | | |
|---|---|---|---|
| *Sample Name:* | **HWC-10-75-3C IB 955 214 0.7** | *Injection Volume:* | **2.0** |
| *Vial Number:* | **GE2** | *Channel:* | **UV_VIS_1** |
| *Sample Type:* | **unknown** | *Wavelength:* | **214** |
| *Control Program:* | **201701-3** | *Bandwidth:* | **n.a.** |
| *Quantif. Method:* | **201701** | *Dilution Factor:* | **1.0000** |
| *Recording Time:* | **2018/6/20 17:59** | *Sample Weight:* | **1.0000** |
| *Run Time (min):* | **15.01** | *Sample Amount:* | **1.0000** |

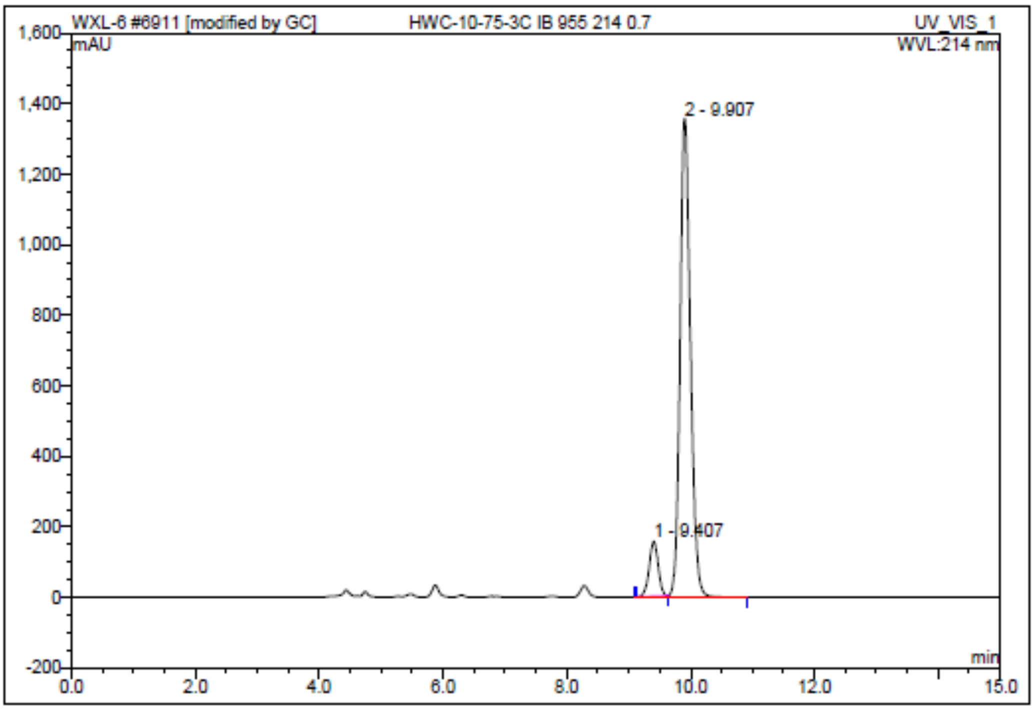


| No. | Ret.Time min | Peak Name | Height mAU | Area mAU*min | Rel.Area % | Amount | Type |
|---|---|---|---|---|---|---|---|
| 1 | 9.41 | n.a. | 155.999 | 26.356 | 9.04 | n.a. | Ru |
| 2 | 9.91 | n.a. | 1357.543 | 265.342 | 90.96 | n.a. | BMB |
| Total: | | | 1513.542 | 291.697 | 100.00 | 0.000 | |

**Supplementary Figure 98.** HPLC chromatogram for compound **6a**

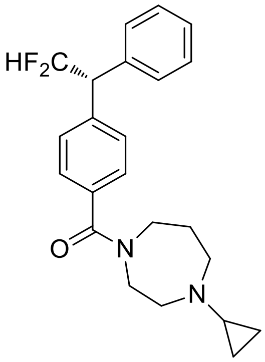


Compound **6**. HPLC (AD-H, 0.46 × 25 cm, 5 μm, hexane/isopropanol = 8/2 (v/v %), flow 0.7 mL/min, UV detection at 214 nm), retention time = 20.70 min (minor) and 23.46 min (major). $[\alpha]_D^{25}$ = 3.23 (c = 0.1550, $CHCl_3$, 90:10 e.r.).

**8148 HWC-12-92-3+- ADH 82 214 0.7**

| | | | |
|---|---|---|---|
| *Sample Name:* | HWC-12-92-3+- ADH 82 214 0.7 | *Injection Volume:* | 2.0 |
| *Vial Number:* | GC1 | *Channel:* | UV_VIS_1 |
| *Sample Type:* | unknown | *Wavelength:* | 214 |
| *Control Program:* | 201701-5 | *Bandwidth:* | n.a. |
| *Quantif. Method:* | 201701 | *Dilution Factor:* | 1.0000 |
| *Recording Time:* | 2018/12/13 14:10 | *Sample Weight:* | 1.0000 |
| *Run Time (min):* | 39.73 | *Sample Amount:* | 1.0000 |

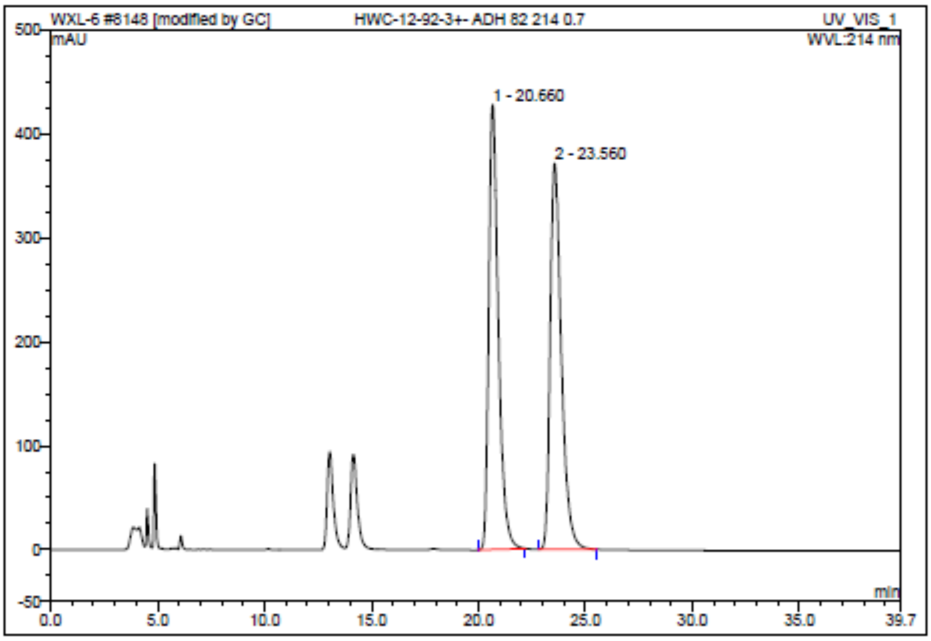


| No. | Ret.Time min | Peak Name | Height mAU | Area mAU*min | Rel.Area % | Amount | Type |
|---|---|---|---|---|---|---|---|
| 1 | 20.66 | n.a. | 428.027 | 223.281 | 49.86 | n.a. | BMB* |
| 2 | 23.56 | n.a. | 371.222 | 224.548 | 50.14 | n.a. | BMB* |
| Total: | | | 799.249 | 447.829 | 100.00 | 0.000 | |

**Supplementary Figure 99.** HPLC chromatogram for compound **6**, racemic

| 8149 HWC-12-94-3 ADH 82 214 0.7 | | | |
|---|---|---|---|
| *Sample Name:* | HWC-12-94-3 ADH 82 214 0.7 | *Injection Volume:* | 2.0 |
| *Vial Number:* | GC2 | *Channel:* | UV_VIS_1 |
| *Sample Type:* | unknown | *Wavelength:* | 214 |
| *Control Program:* | 201701-5 | *Bandwidth:* | n.a. |
| *Quantif. Method:* | 201701 | *Dilution Factor:* | 1.0000 |
| *Recording Time:* | 2018/12/13 14:52 | *Sample Weight:* | 1.0000 |
| *Run Time (min):* | 27.50 | *Sample Amount:* | 1.0000 |

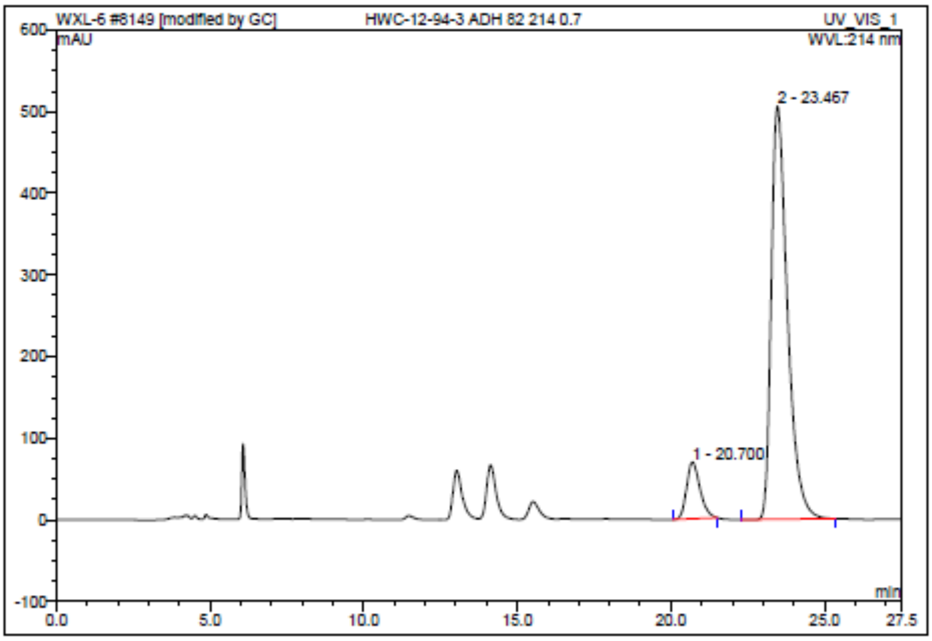


| No. | Ret.Time min | Peak Name | Height mAU | Area mAU*min | Rel.Area % | Amount | Type |
|---|---|---|---|---|---|---|---|
| 1 | 20.70 | n.a. | 69.665 | 35.105 | 10.20 | n.a. | BMB* |
| 2 | 23.47 | n.a. | 505.981 | 308.985 | 89.80 | n.a. | BMB* |
| Total: | | | 575.646 | 344.090 | 100.00 | 0.000 | |

**Supplementary Figure 100.** HPLC chromatogram for compound **6**

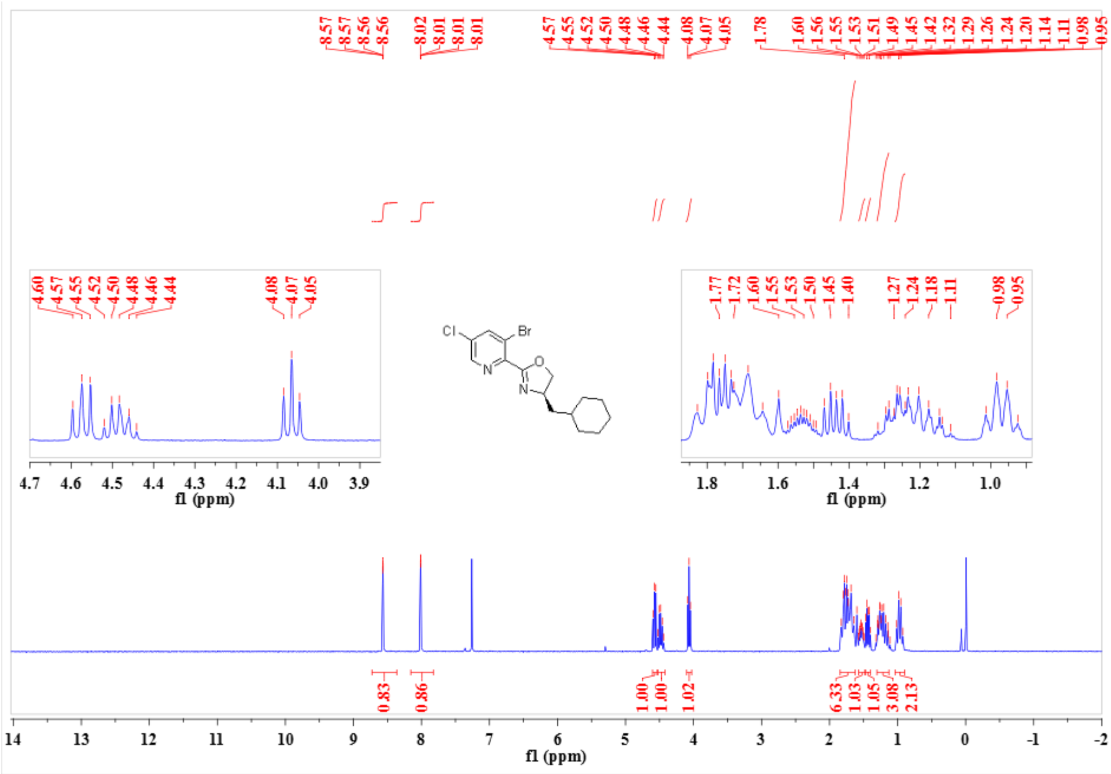

Supplementary Figure 101. $^{1}$H NMR (400 MHz, $CDCl_3$) spectrum of L2

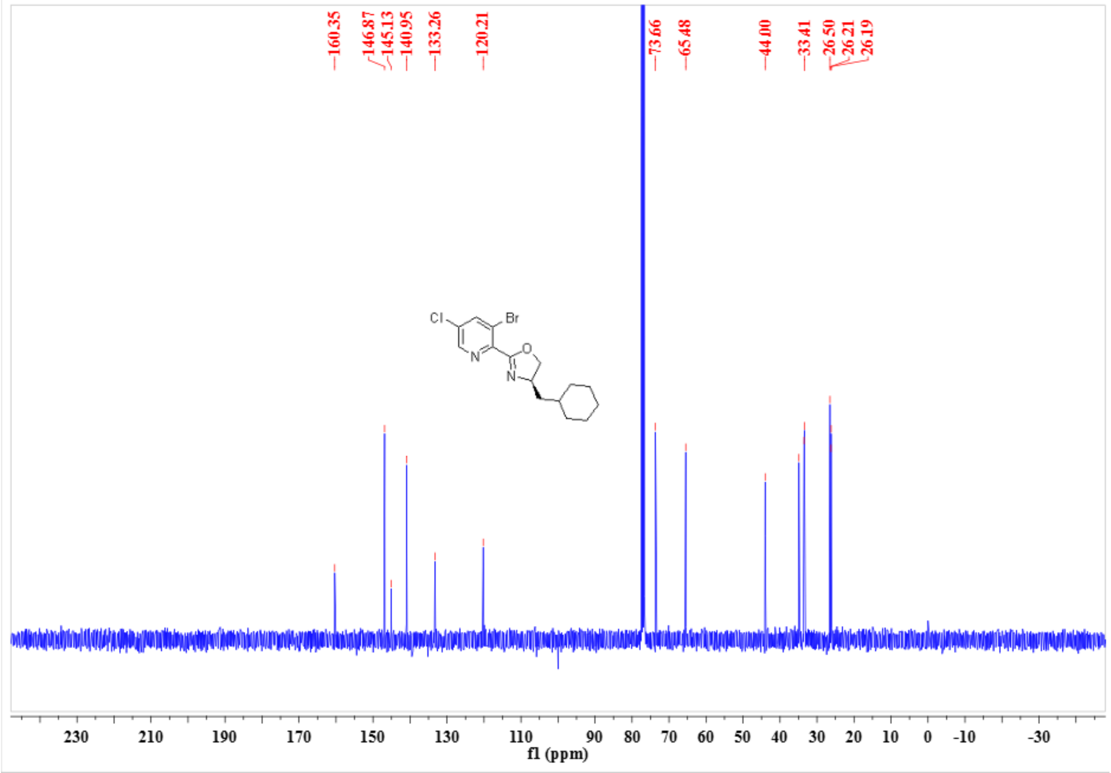

Supplementary Figure 102. $^{13}$C NMR (101 MHz, $CDCl_3$) spectrum of L2

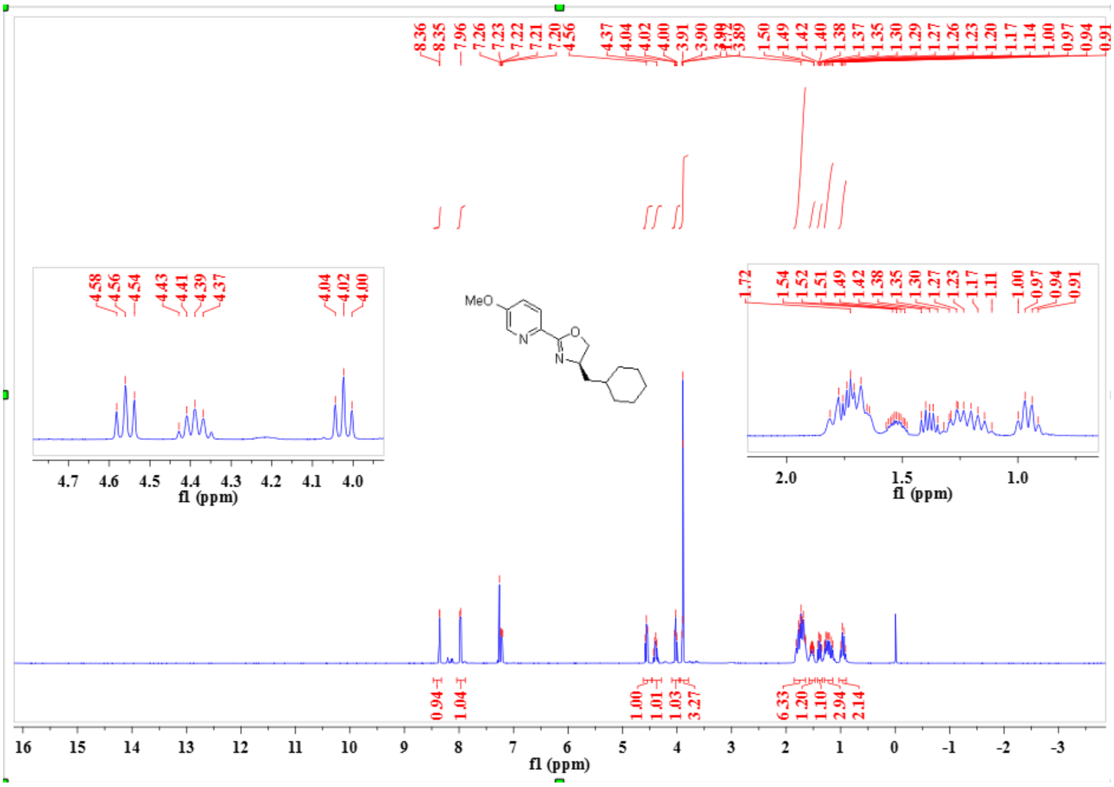

**Supplementary Figure 103.** $^{1}$**H NMR (400 MHz,** $CDCl_3$**) spectrum of L3**

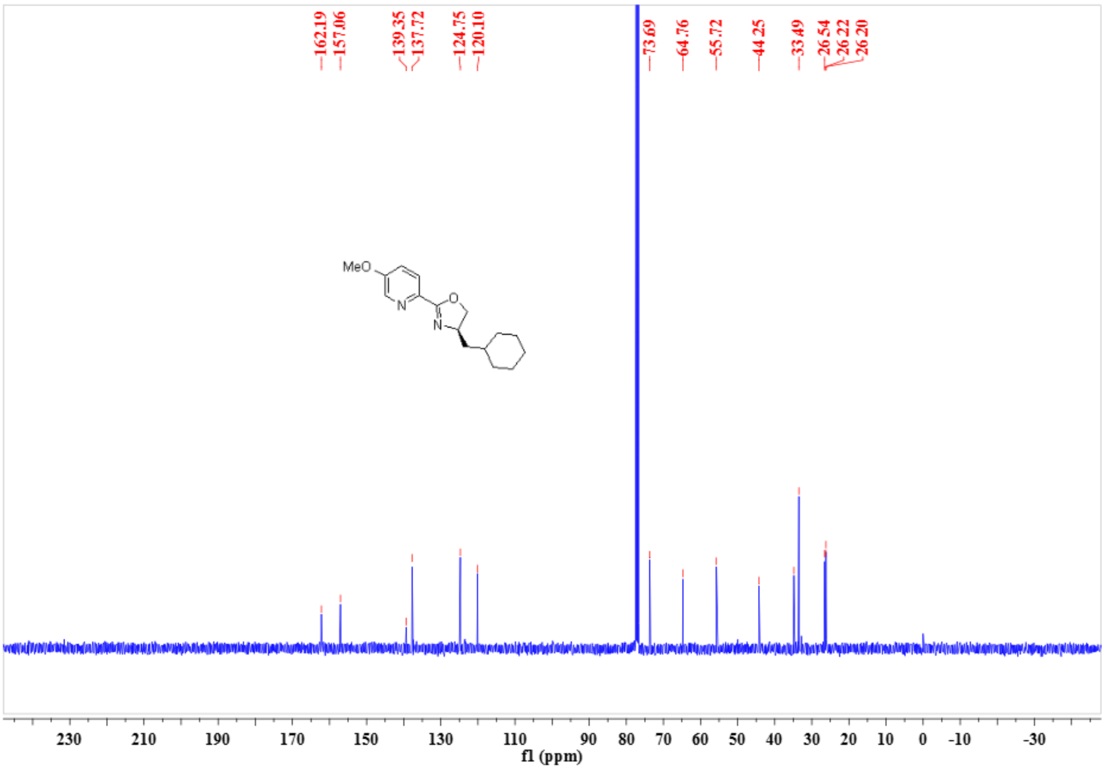

**Supplementary Figure 104.** $^{13}$**C NMR (101 MHz,** $CDCl_3$**) spectrum of L3**

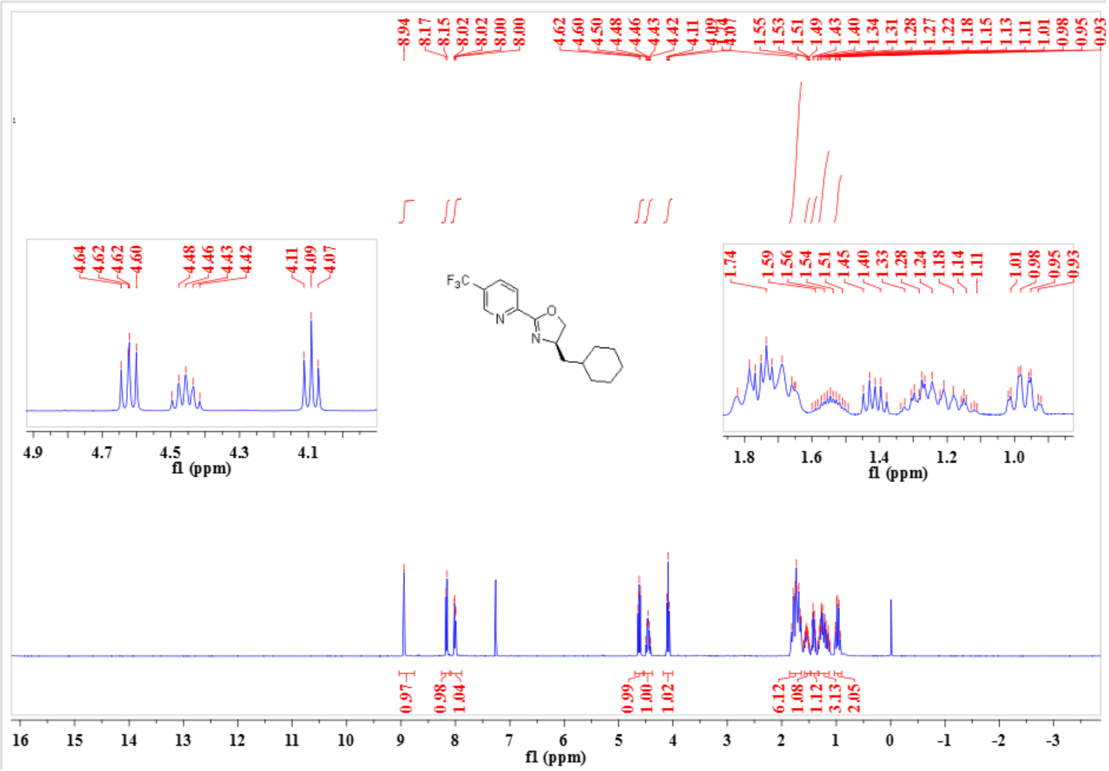


**Supplementary Figure 105.** **$^{1}H$ NMR (400 MHz, $CDCl_3$) spectrum of L4**

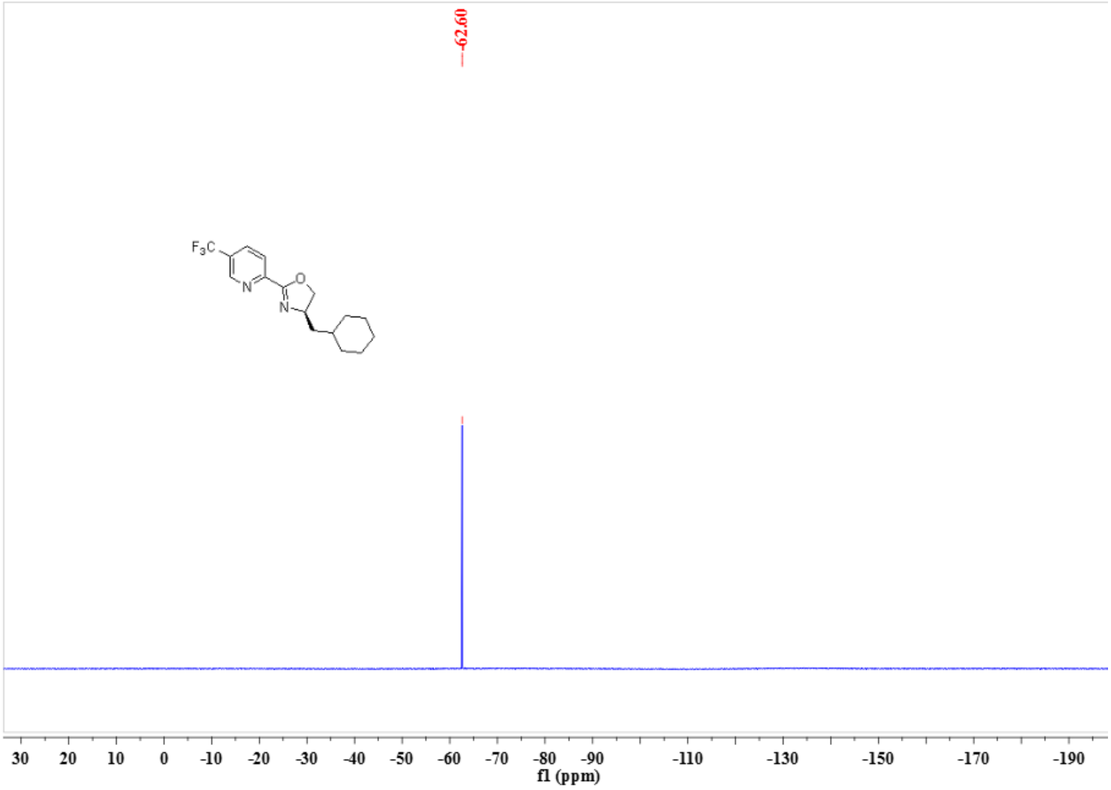


**Supplementary Figure 106.** **$^{19}F$ NMR (376 MHz, $CDCl_3$) spectrum of L4**

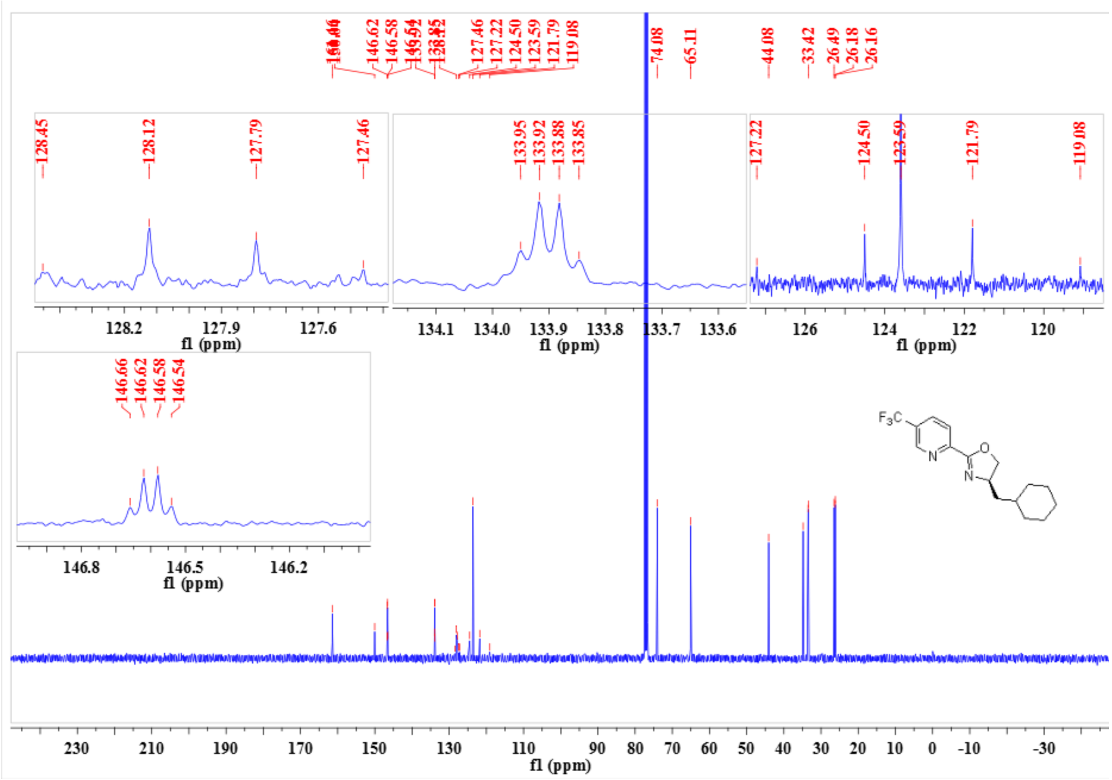

Supplementary Figure 107. $^{13}C$ NMR (101 MHz, $CDCl_3$) spectrum of L4

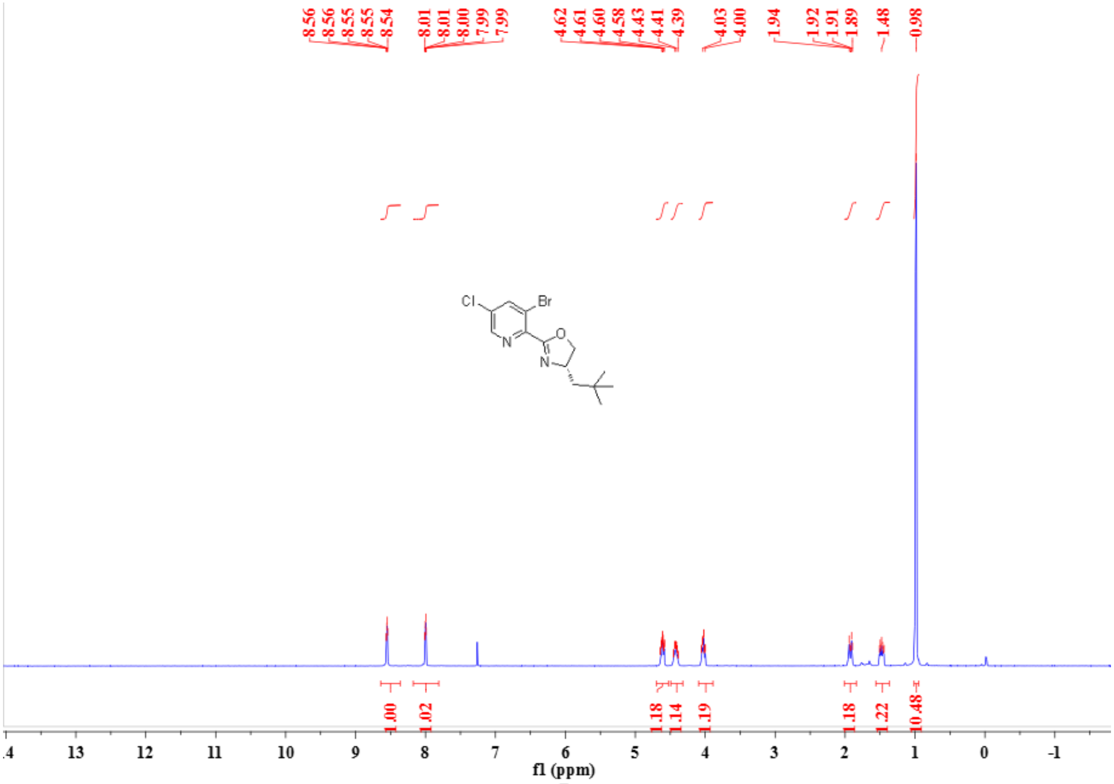

Supplementary Figure 108. $^{1}H$ NMR (400 MHz, $CDCl_3$) spectrum of L7

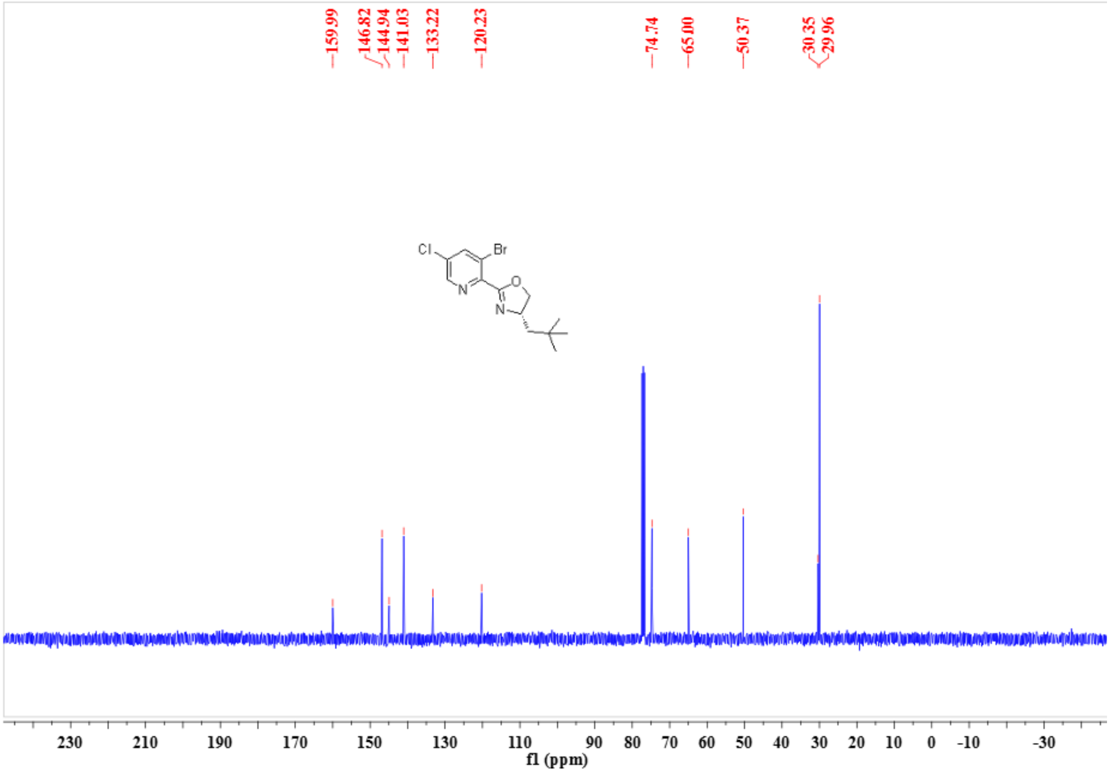


**Supplementary Figure 109.** $^{13}C$ **NMR (101 MHz,** $CDCl_3$**) spectrum of L7**

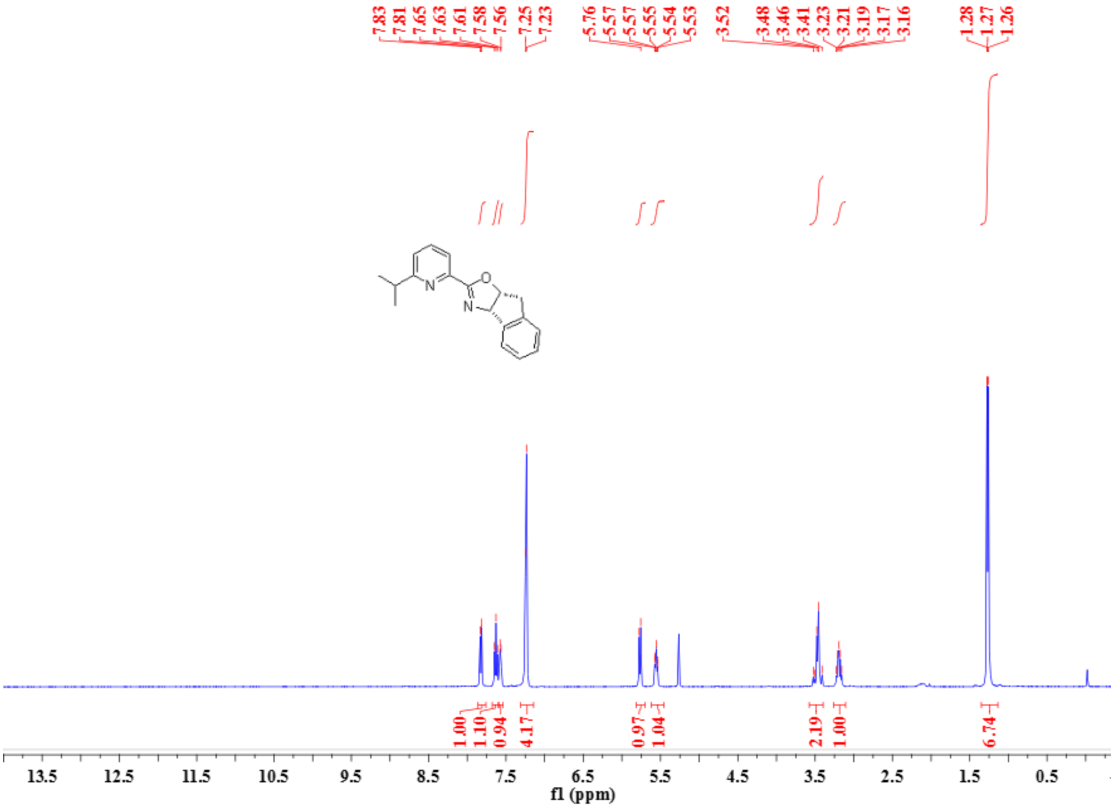


**Supplementary Figure 110.** $^{1}H$ **NMR (400 MHz,** $CDCl_3$**) spectrum of L8**

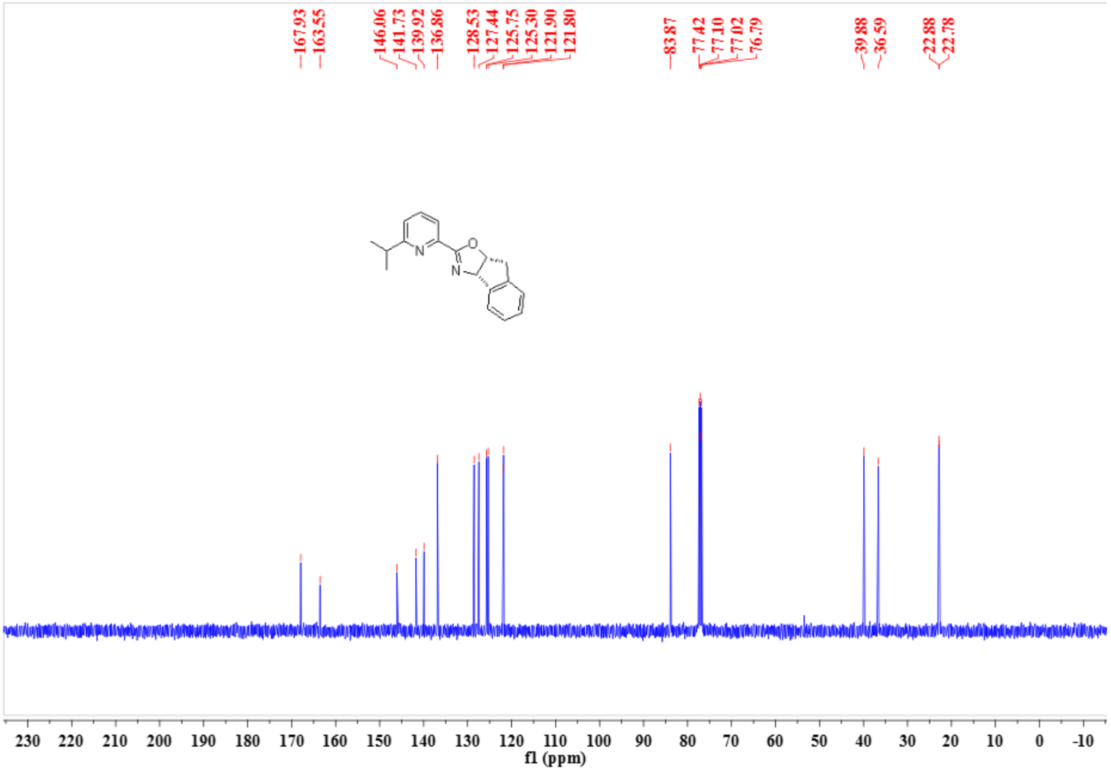


**Supplementary Figure 111.** $^{13}$**C NMR (101 MHz, CDCl$_3$) spectrum of L8**

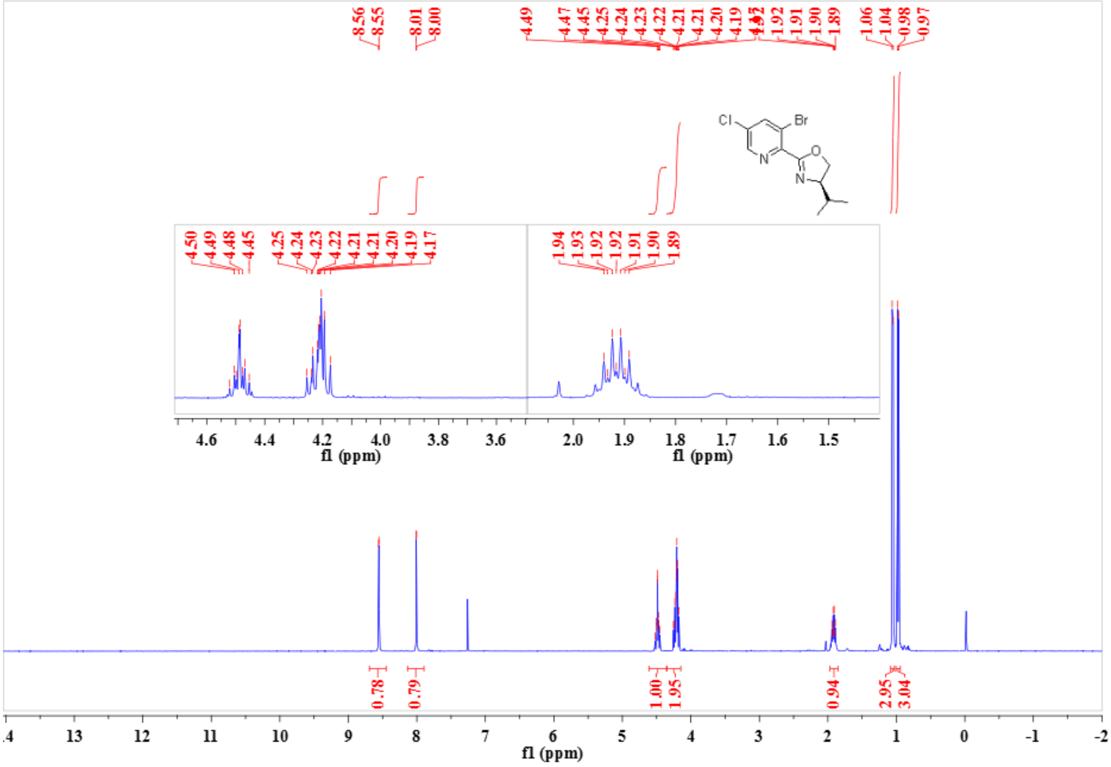


**Supplementary Figure 112.** $^{1}$**H NMR (400 MHz, CDCl$_3$) spectrum of L9**

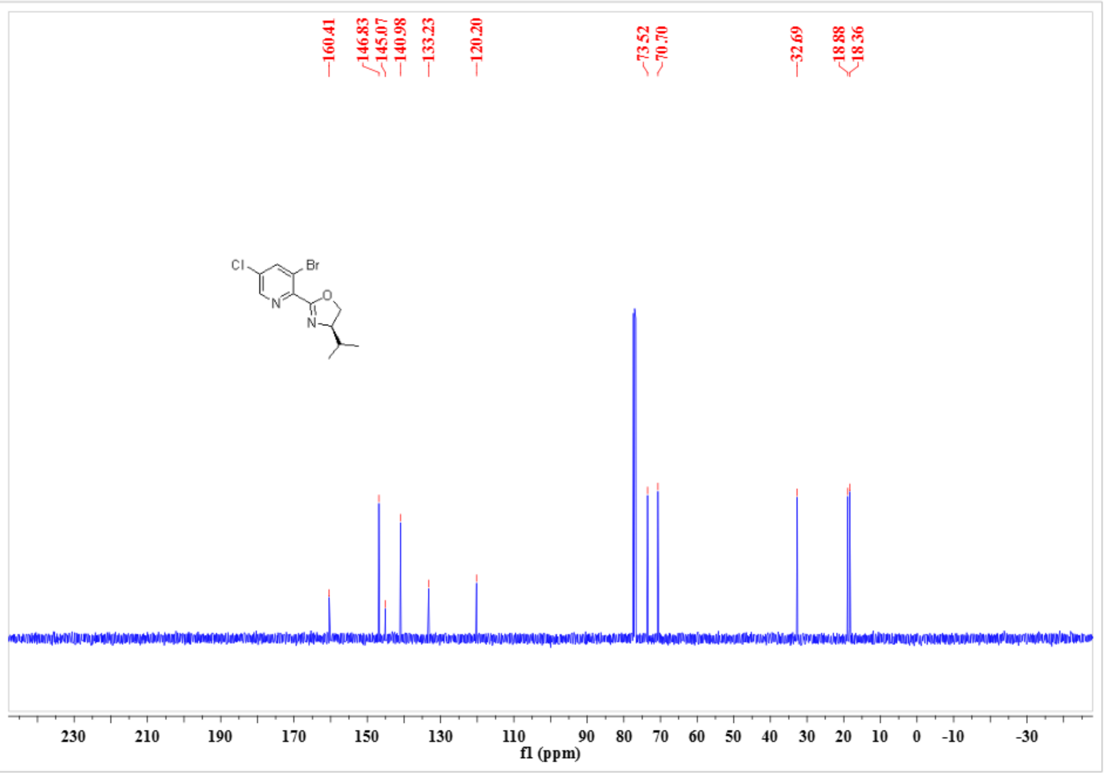

Supplementary Figure 113. $^{13}C$ NMR (101 MHz, $CDCl_3$) spectrum of L9

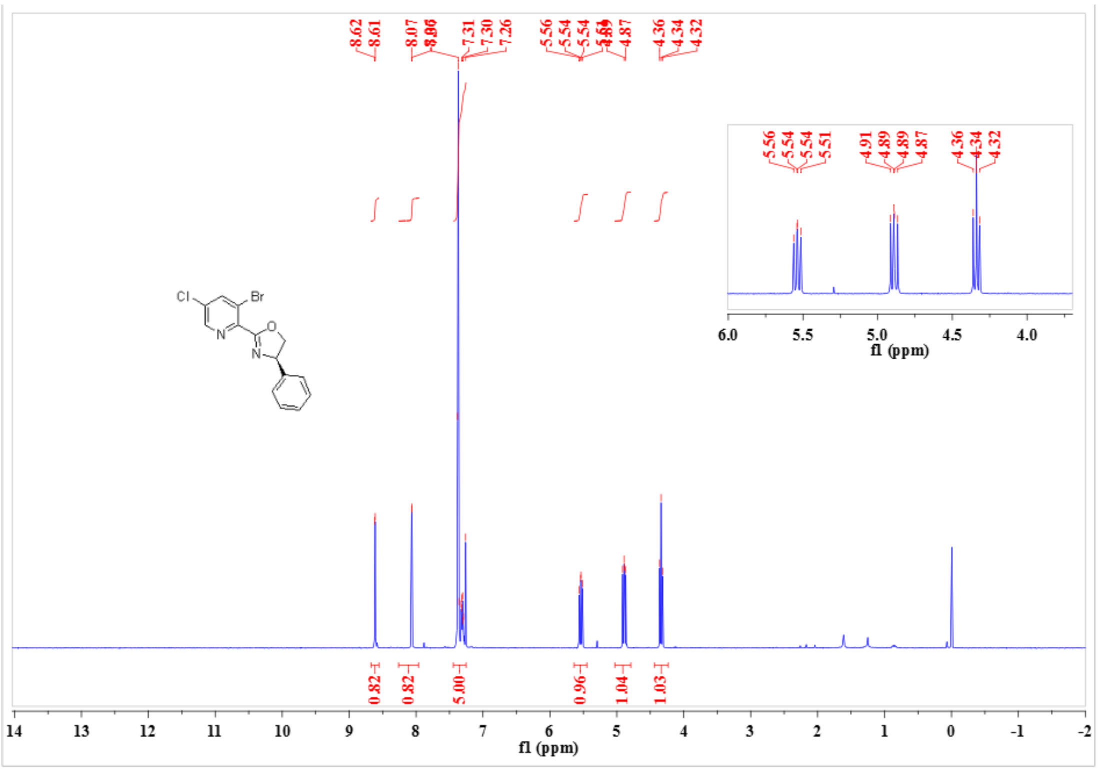

Supplementary Figure 114. $^{1}H$ NMR (400 MHz, $CDCl_3$) spectrum of L10

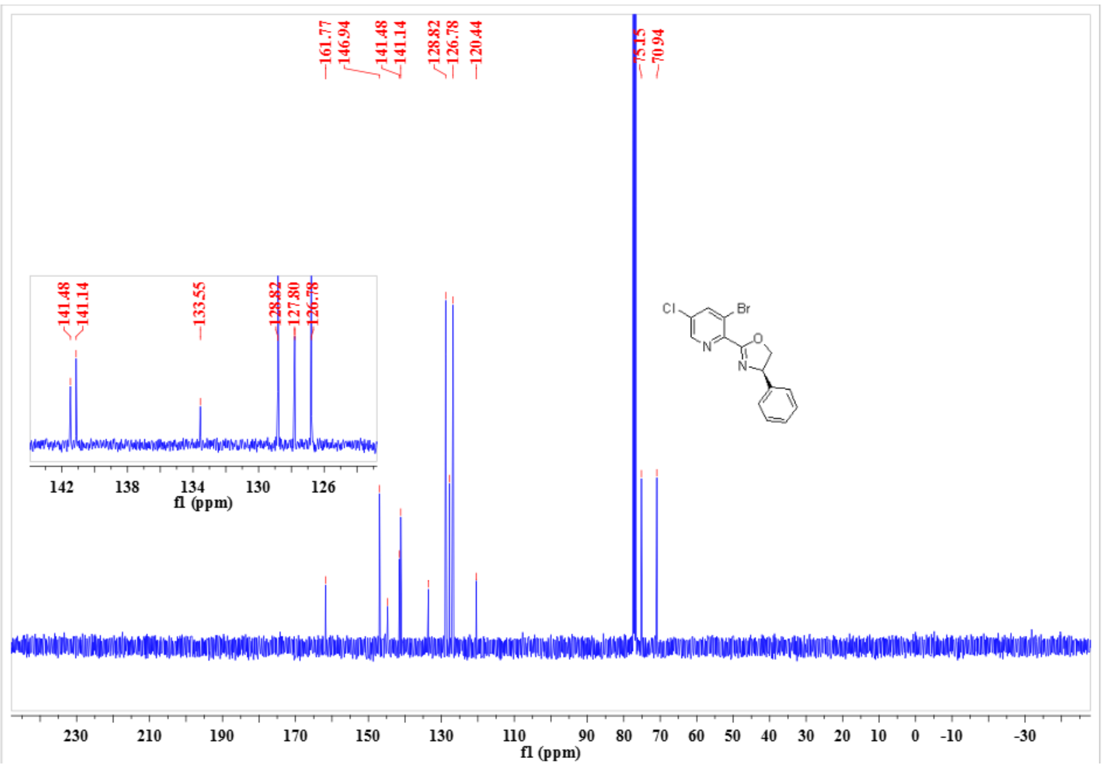


**Supplementary Figure 115. $^{13}$C NMR (101 MHz, $CDCl_3$) spectrum of L10**

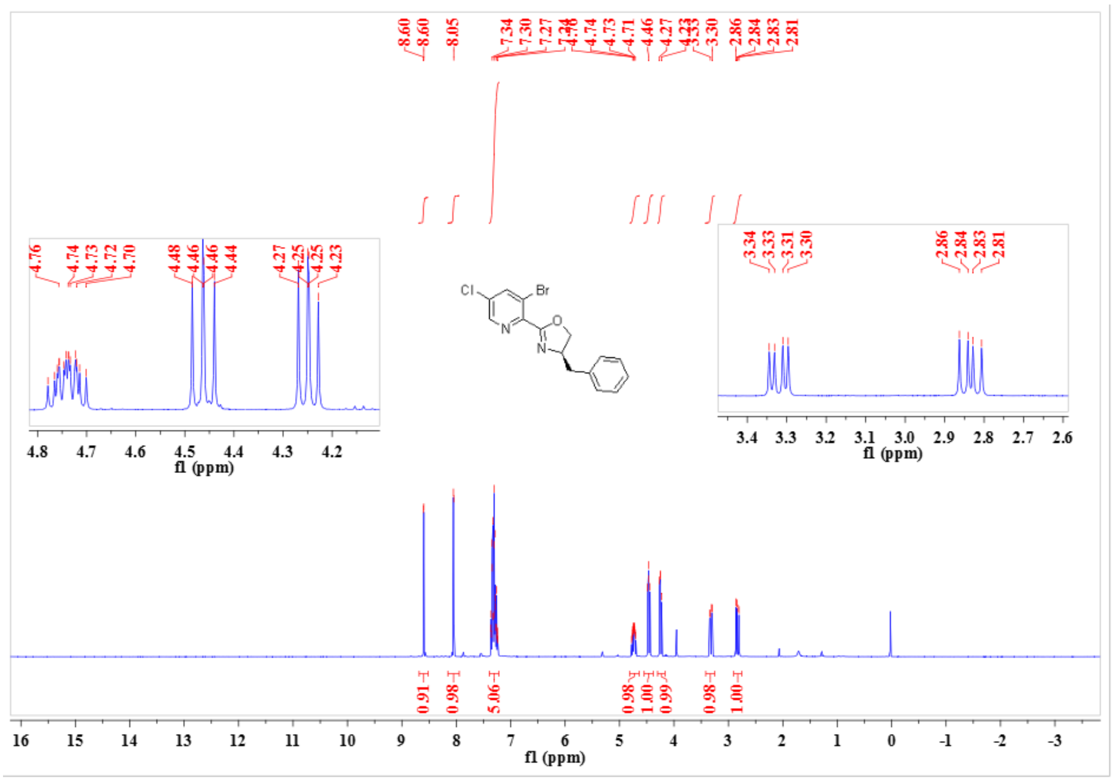


**Supplementary Figure 116. $^{1}$H NMR (400 MHz, $CDCl_3$) spectrum of L11**

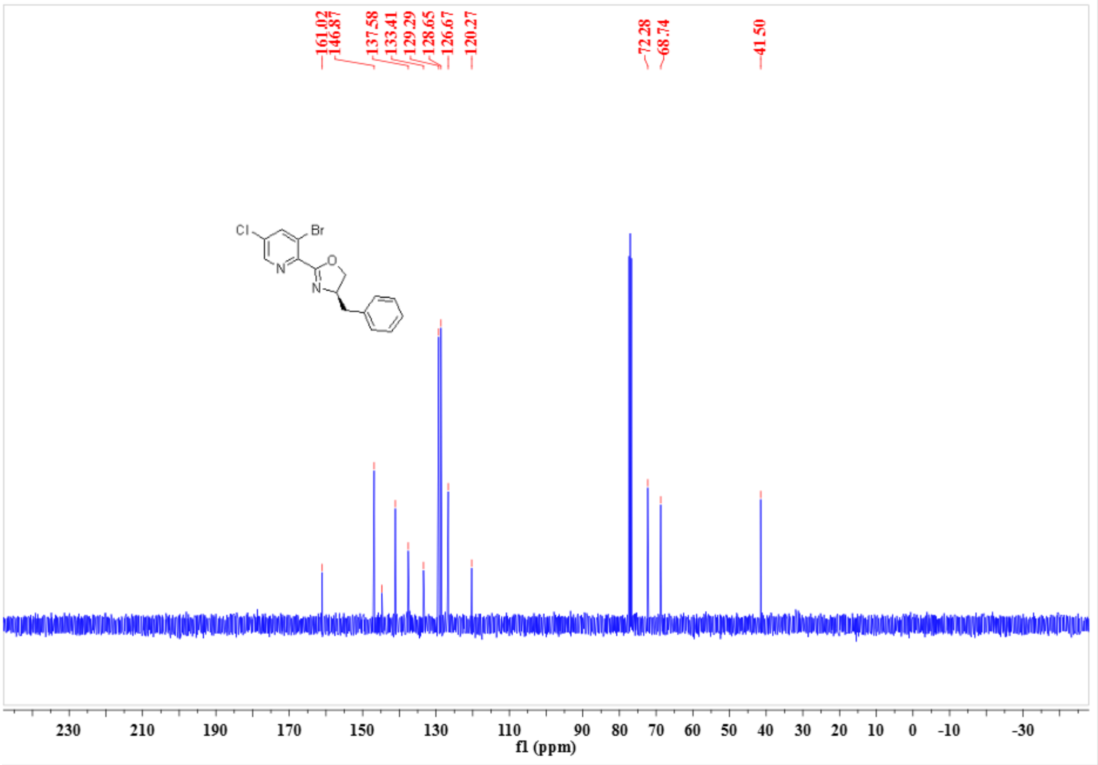


**Supplementary Figure 117.** $^{13}$**C NMR (101 MHz, CDCl**$_3$**) spectrum of L11**

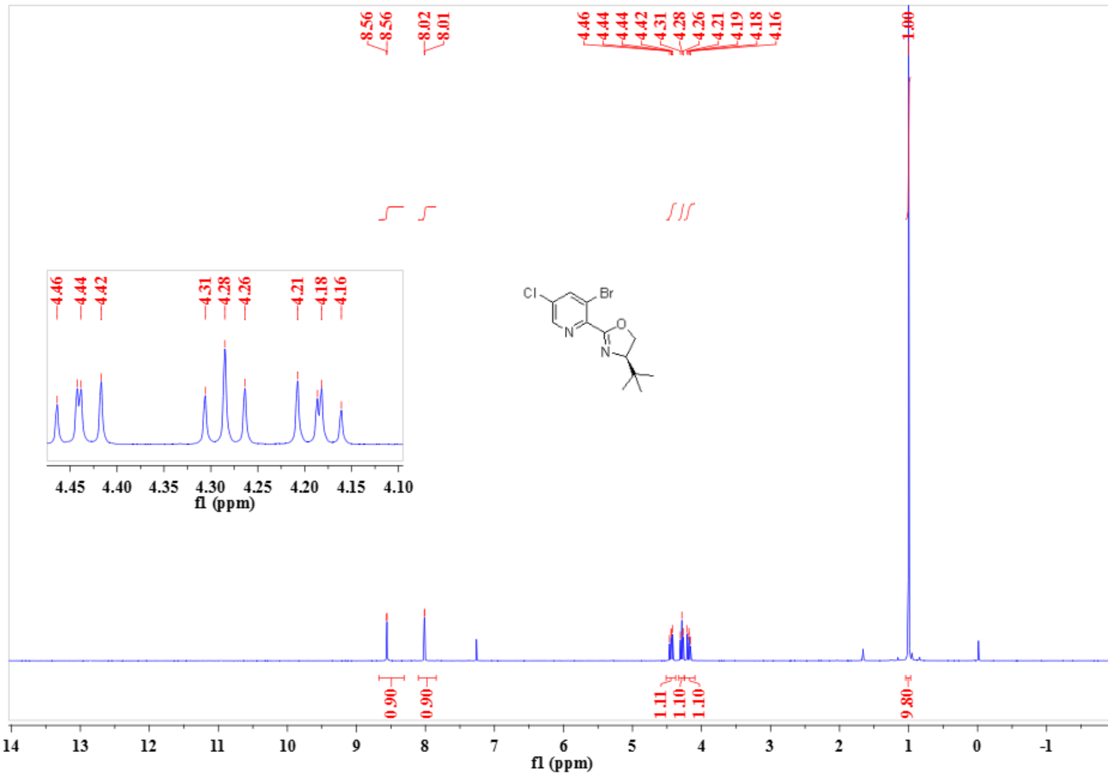


**Supplementary Figure 118.** $^{1}$**H NMR (400 MHz, CDCl**$_3$**) spectrum of L12**

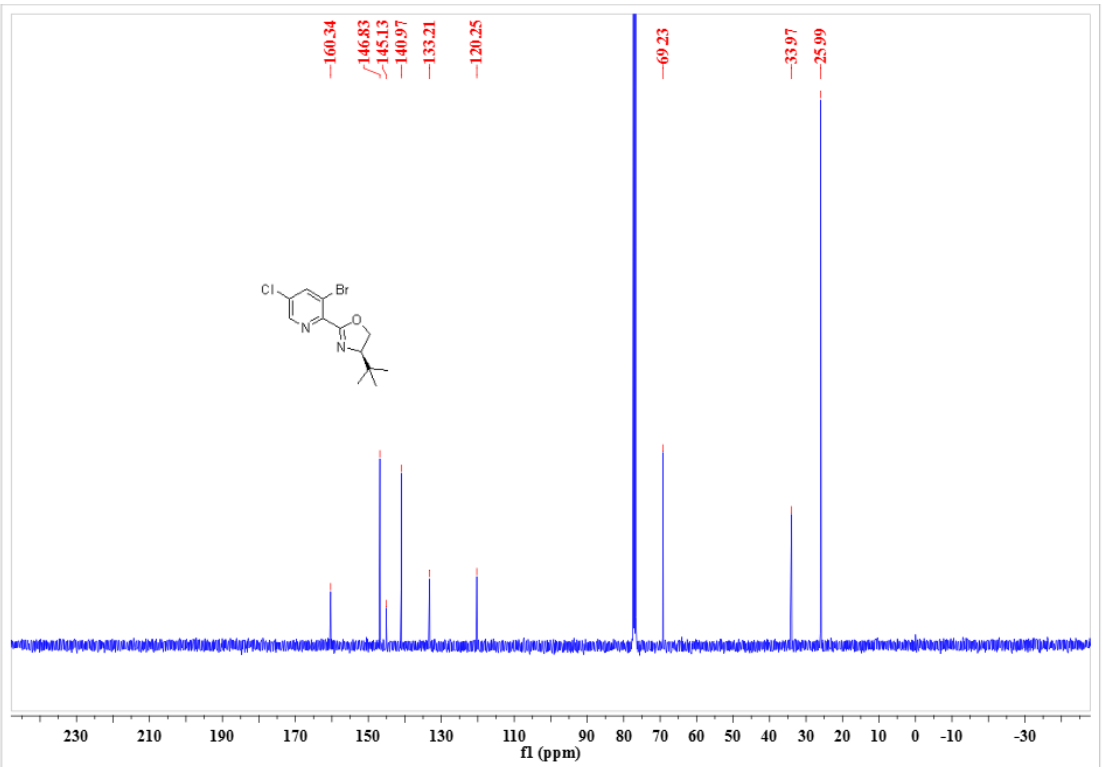

**Supplementary Figure 119.** $^{13}$**C NMR (101 MHz, CDCl$_3$) spectrum of L12**

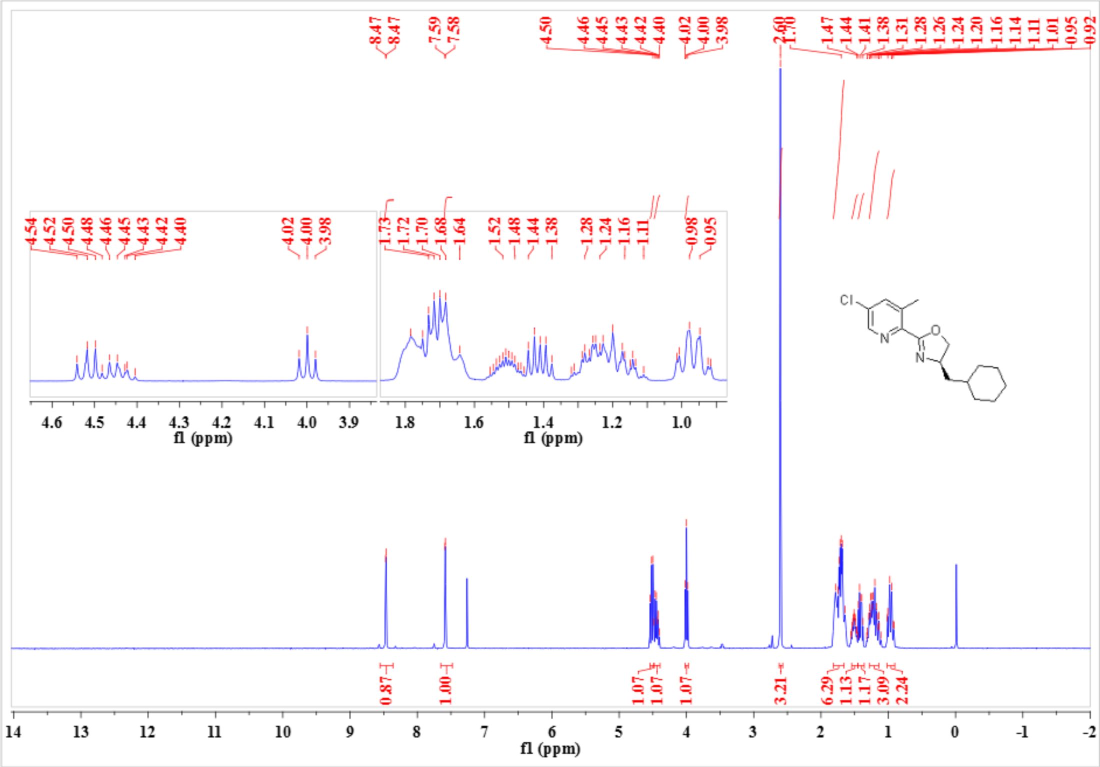

**Supplementary Figure 120.** $^{1}$**H NMR (400 MHz, CDCl$_3$) spectrum of L13**

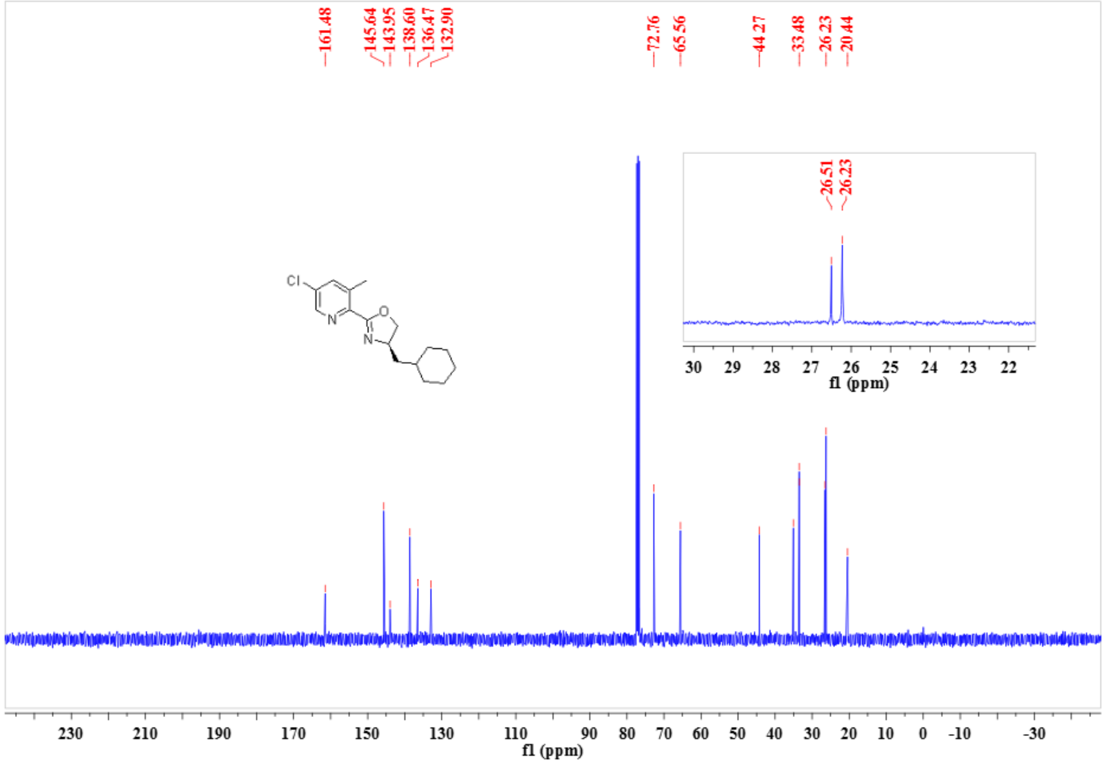

**Supplementary Figure 121.** $^{13}$C NMR (101 MHz, $CDCl_3$) spectrum of L13

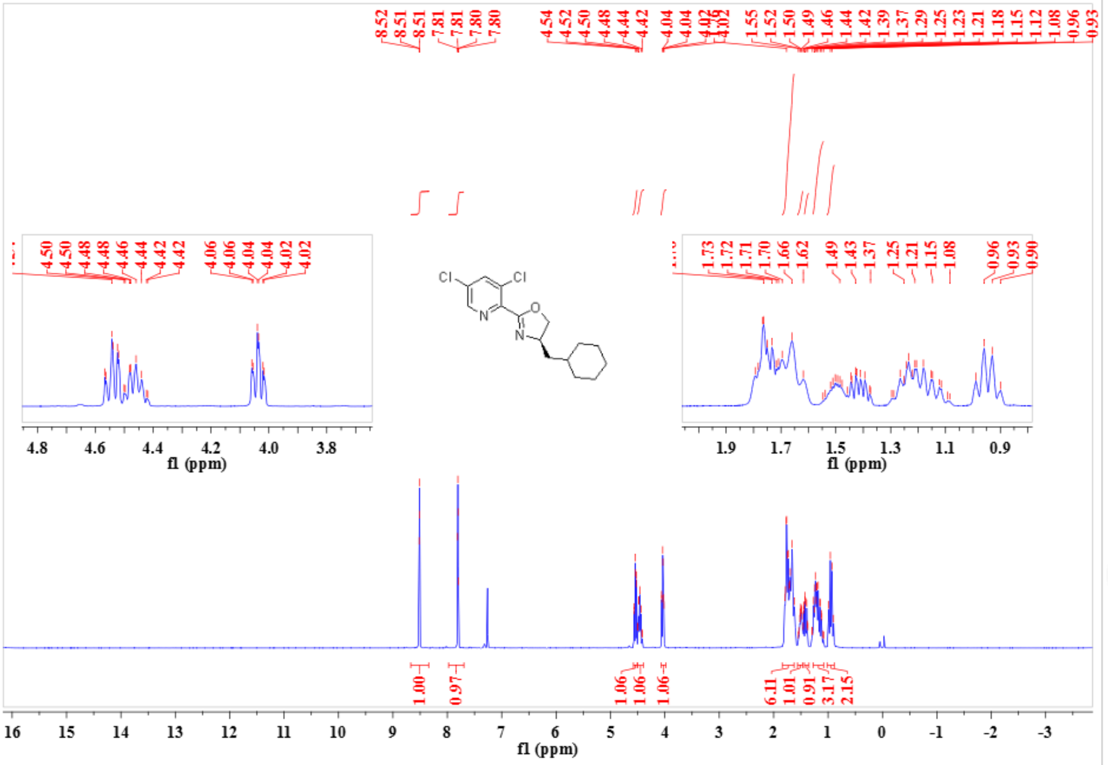

**Supplementary Figure 122.** $^{1}$H NMR (400 MHz, $CDCl_3$) spectrum of L14

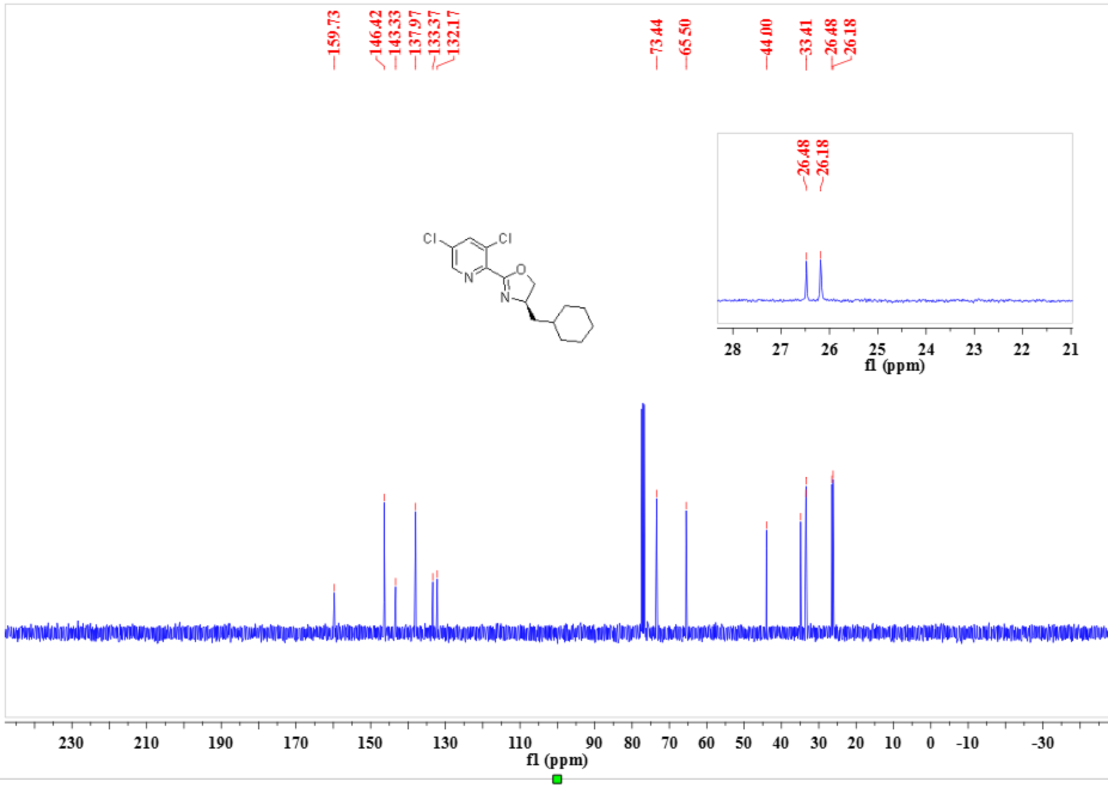

**Supplementary Figure 123. $^{13}$C NMR (101 MHz, $CDCl_3$) spectrum of L14**

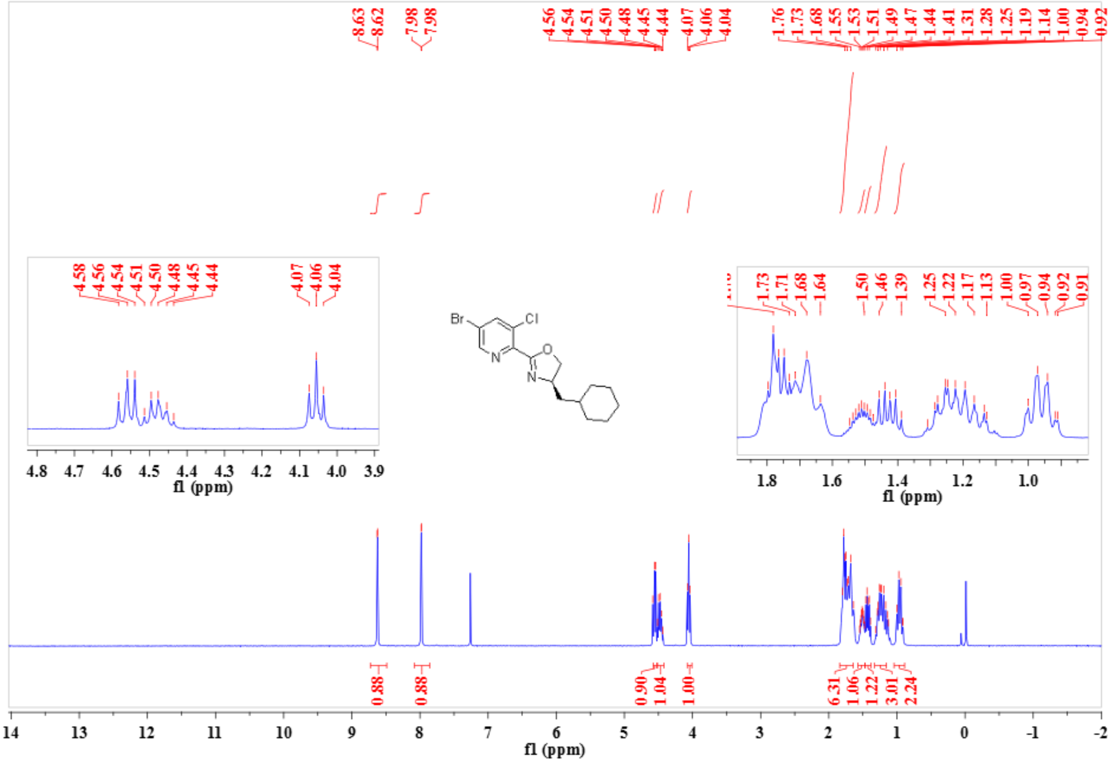

**Supplementary Figure 124. $^{1}$H NMR (400 MHz, $CDCl_3$) spectrum of L15**

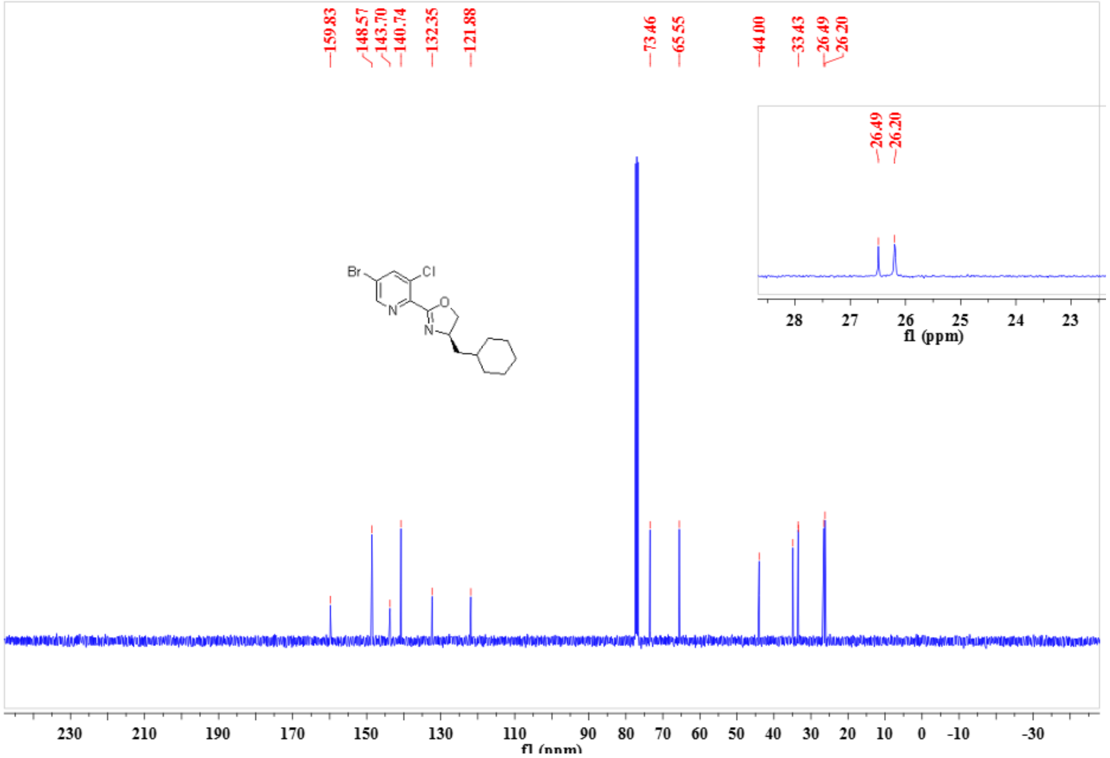

**Supplementary Figure 125.** $^{13}C$ **NMR (101 MHz,** $CDCl_3$**) spectrum of L15**

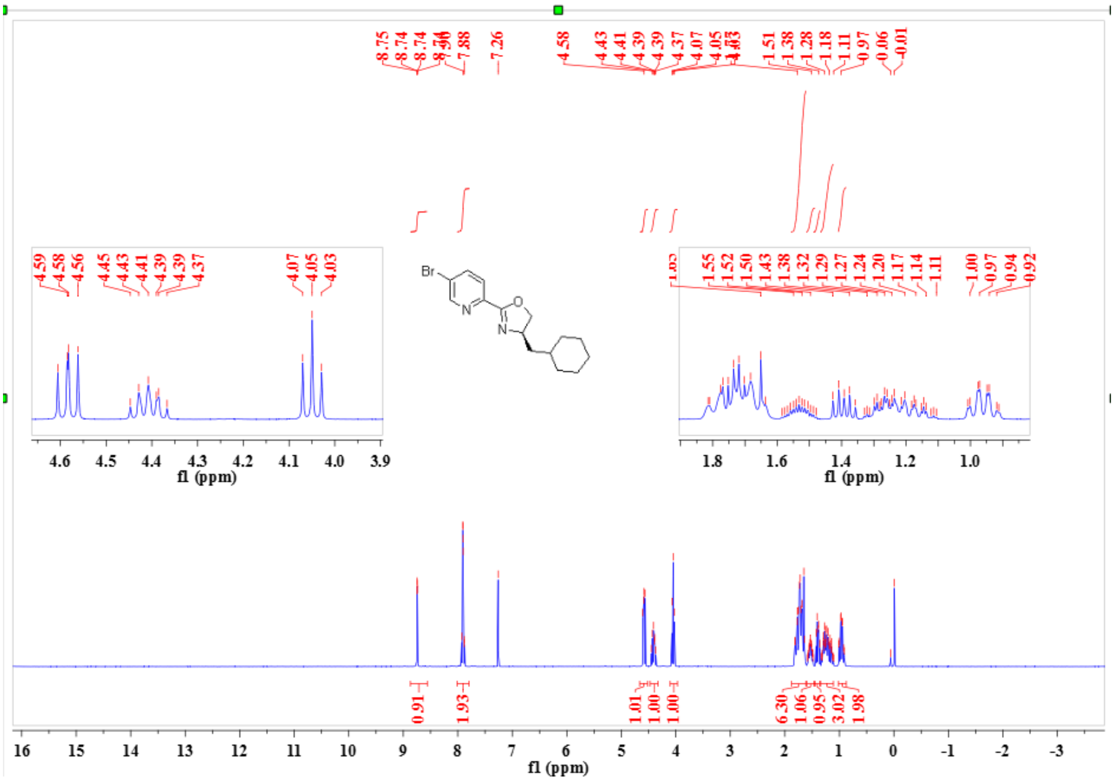

**Supplementary Figure 126.** $^{1}H$ **NMR (400 MHz,** $CDCl_3$**) spectrum of L16**

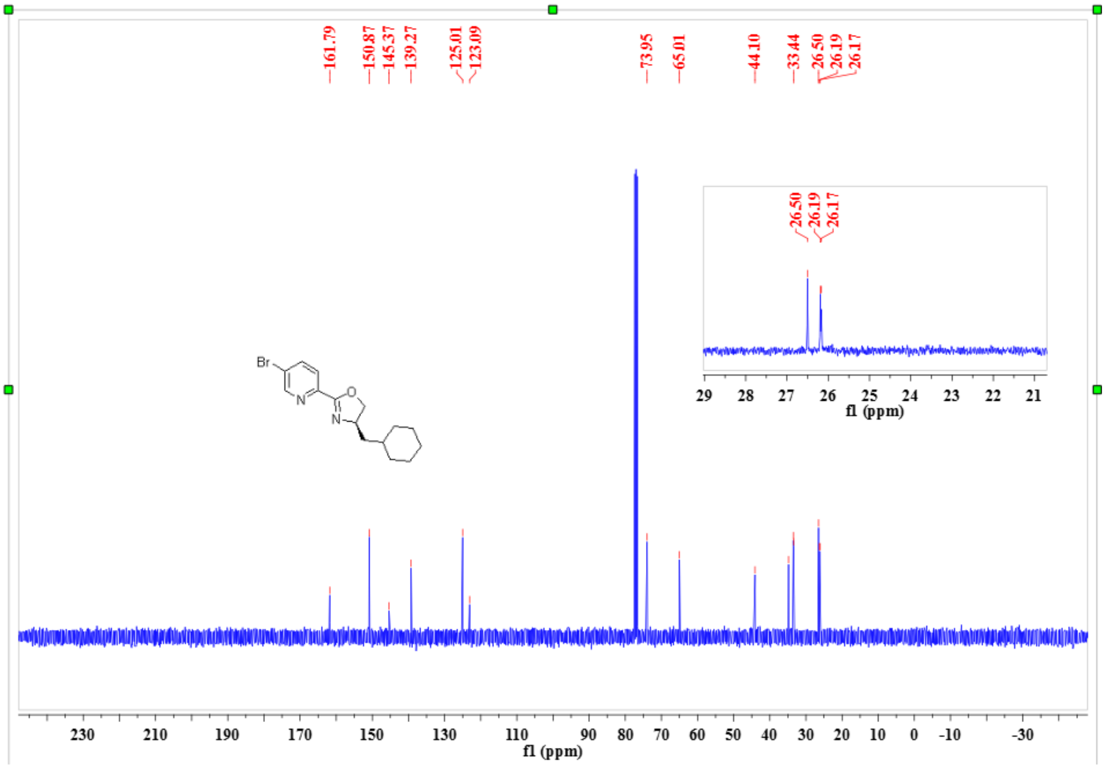


**Supplementary Figure 127.** $^{13}$C **NMR (101 MHz, CDCl$_3$) spectrum of L16**

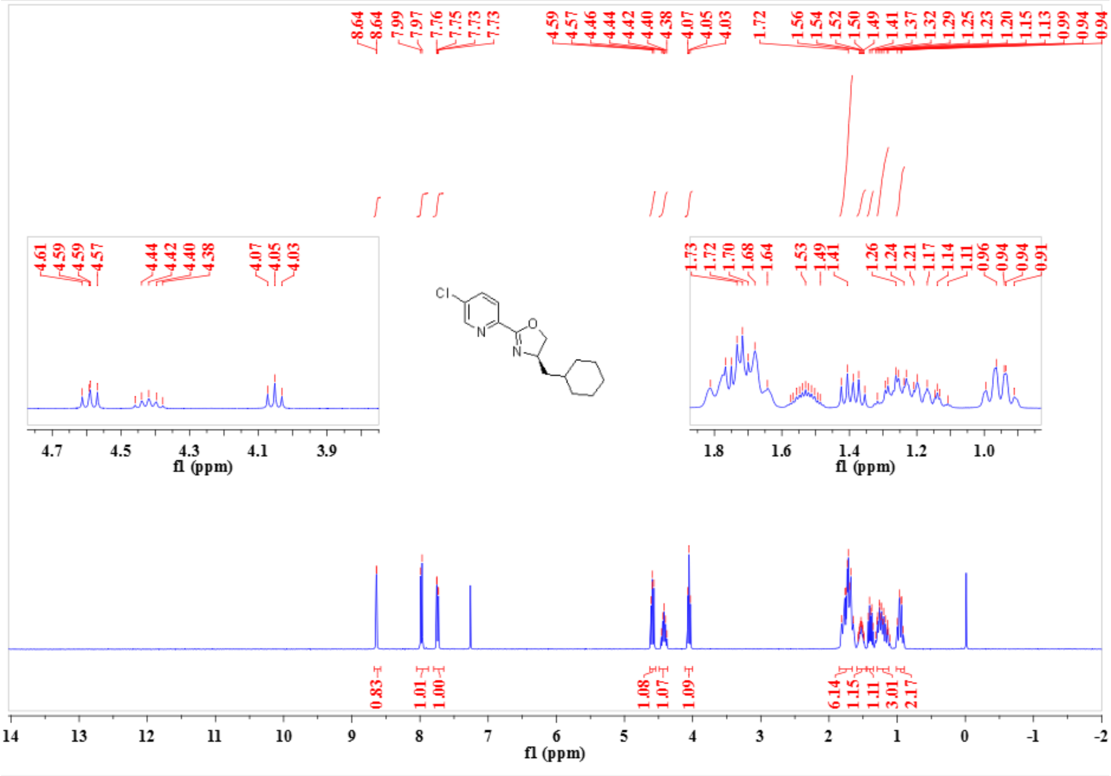


**Supplementary Figure 128.** $^{1}$H **NMR (400 MHz, CDCl$_3$) spectrum of L17**

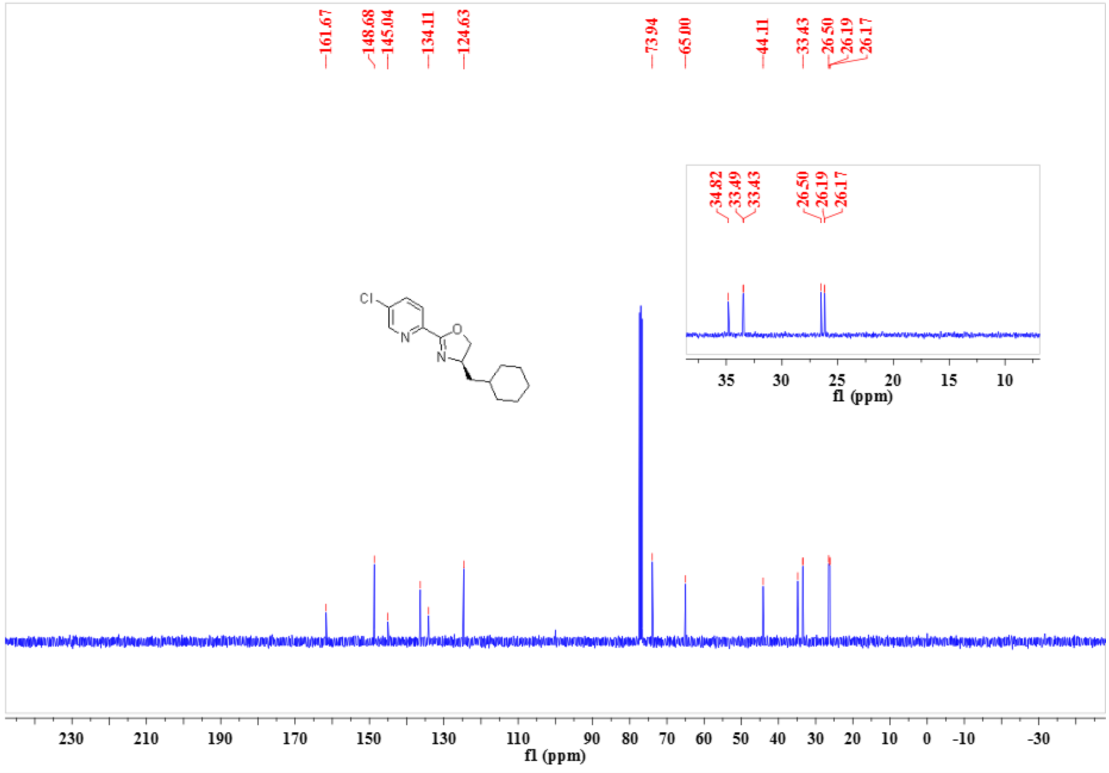

**Supplementary Figure 129. $^{13}C$ NMR (101 MHz, $CDCl_3$) spectrum of L17**

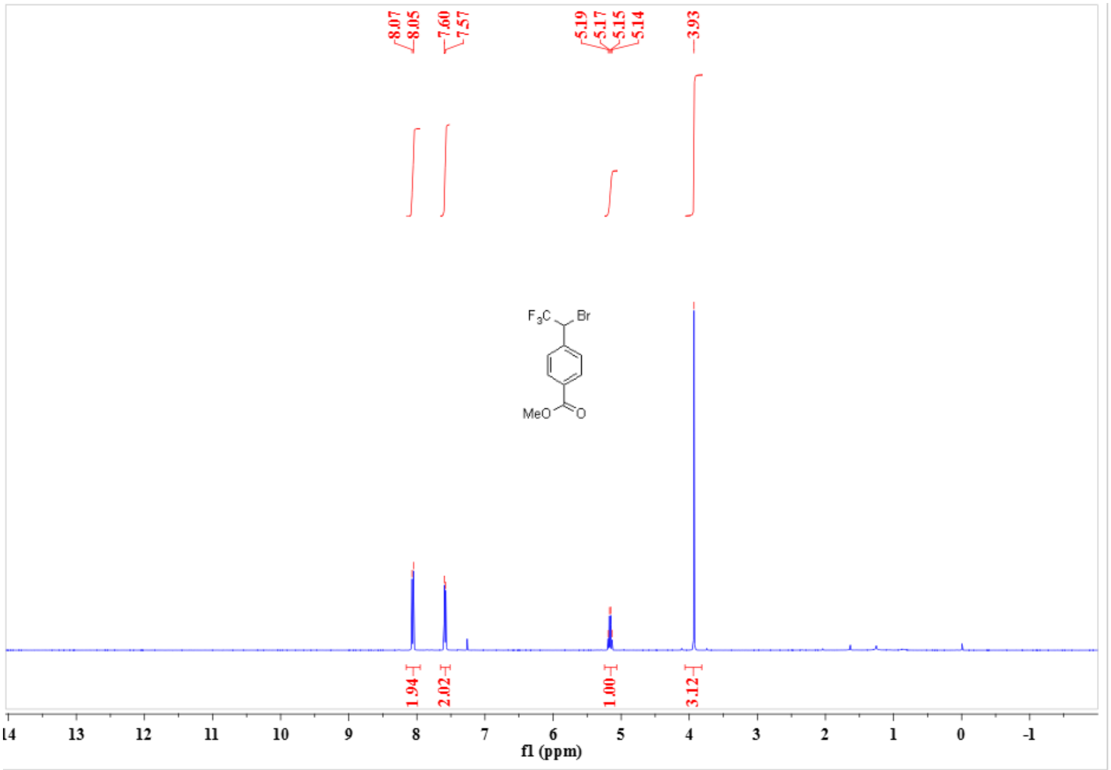

**Supplementary Figure 130. $^{1}H$ NMR (400 MHz, $CDCl_3$) spectrum of 1a**

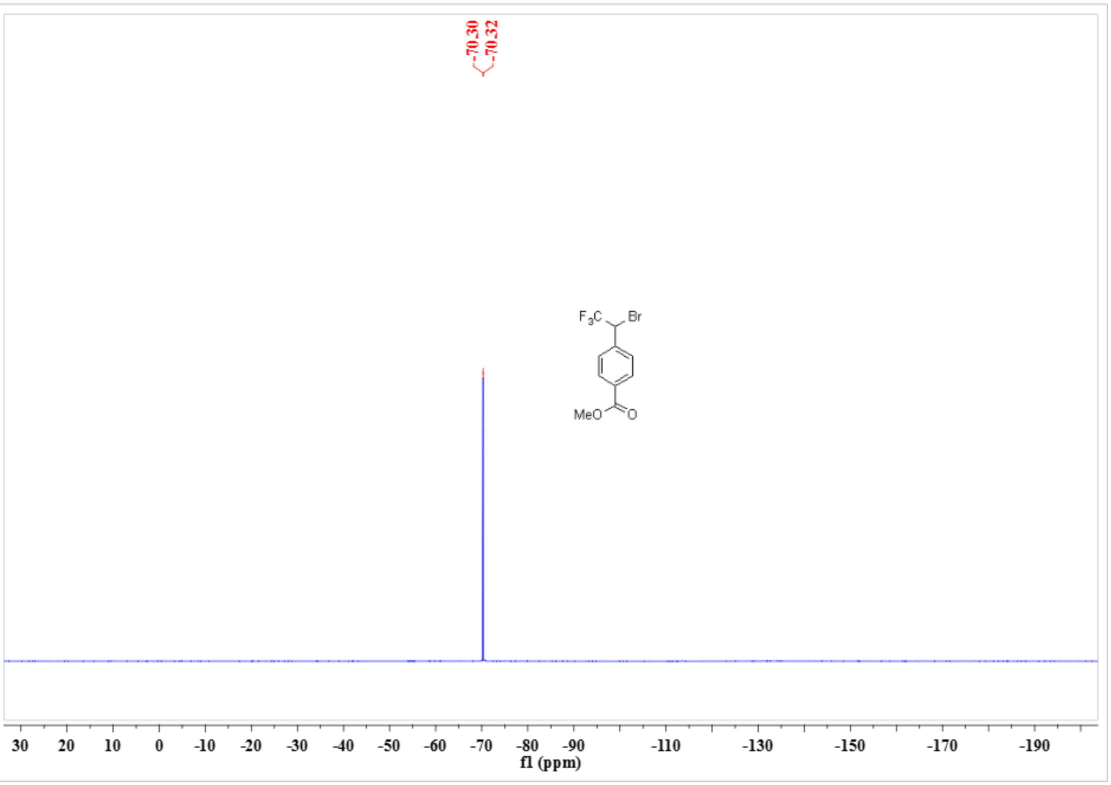


**Supplementary Figure 131.** $^{19}$**F NMR (376 MHz, CDCl$_3$) spectrum of 1a**

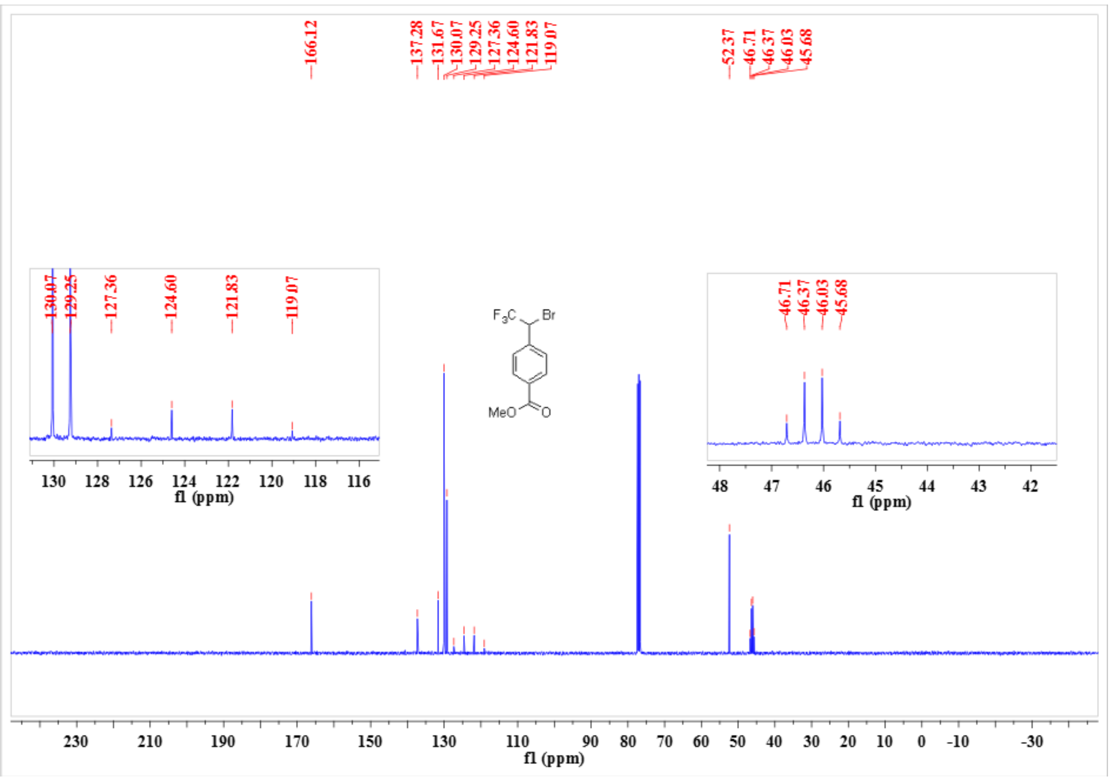


**Supplementary Figure 132.** $^{13}$**C NMR (101 MHz, CDCl$_3$) spectrum of 1a**

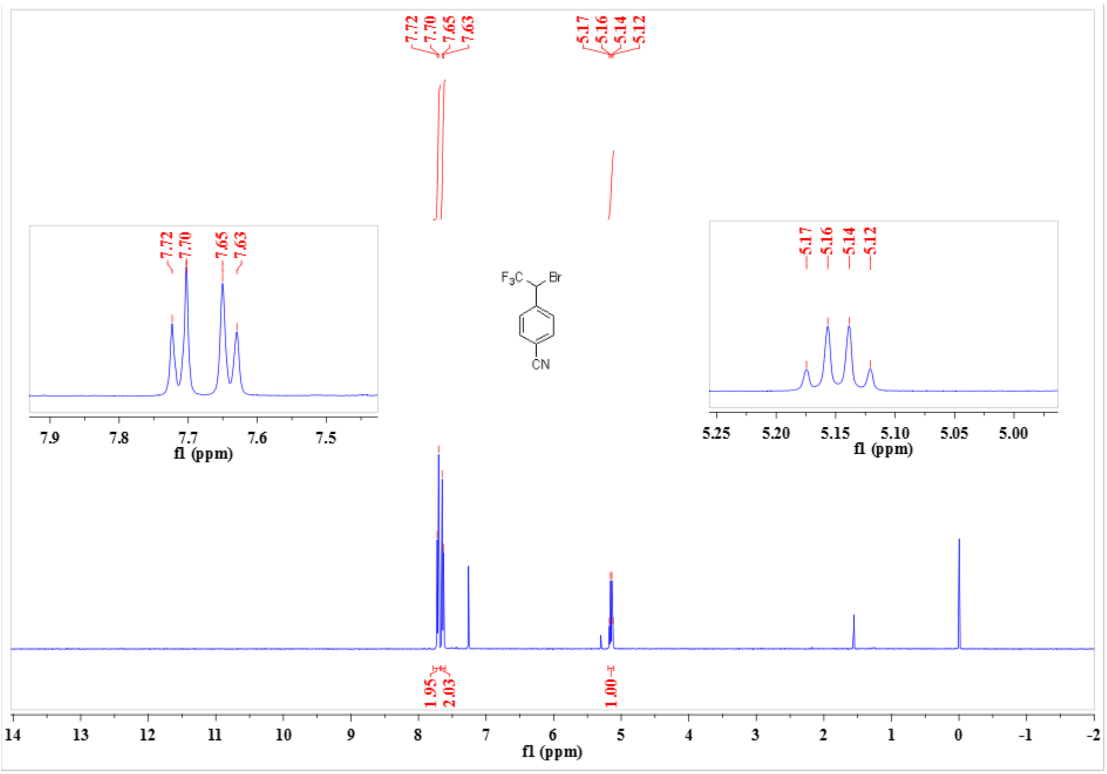

**Supplementary Figure 133.** $^{1}$**H NMR (400 MHz, CDCl$_3$) spectrum of 1b**

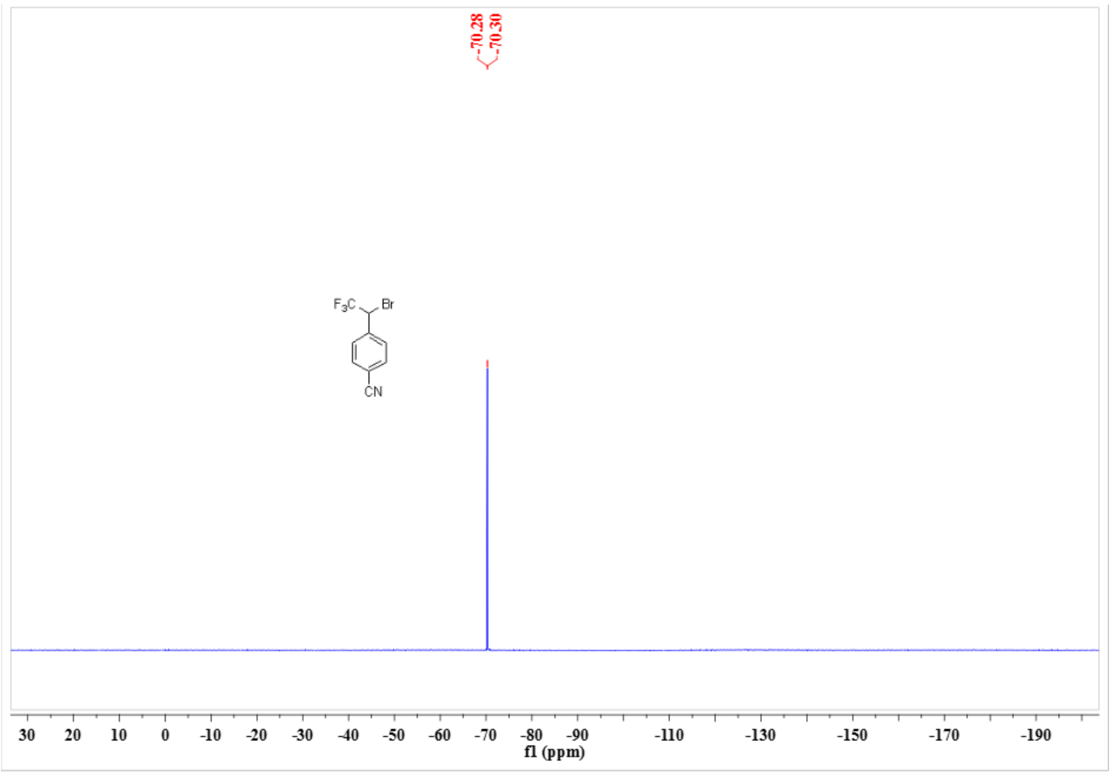

**Supplementary Figure 134.** $^{19}$**F NMR (376 MHz, CDCl$_3$) spectrum of 1b**

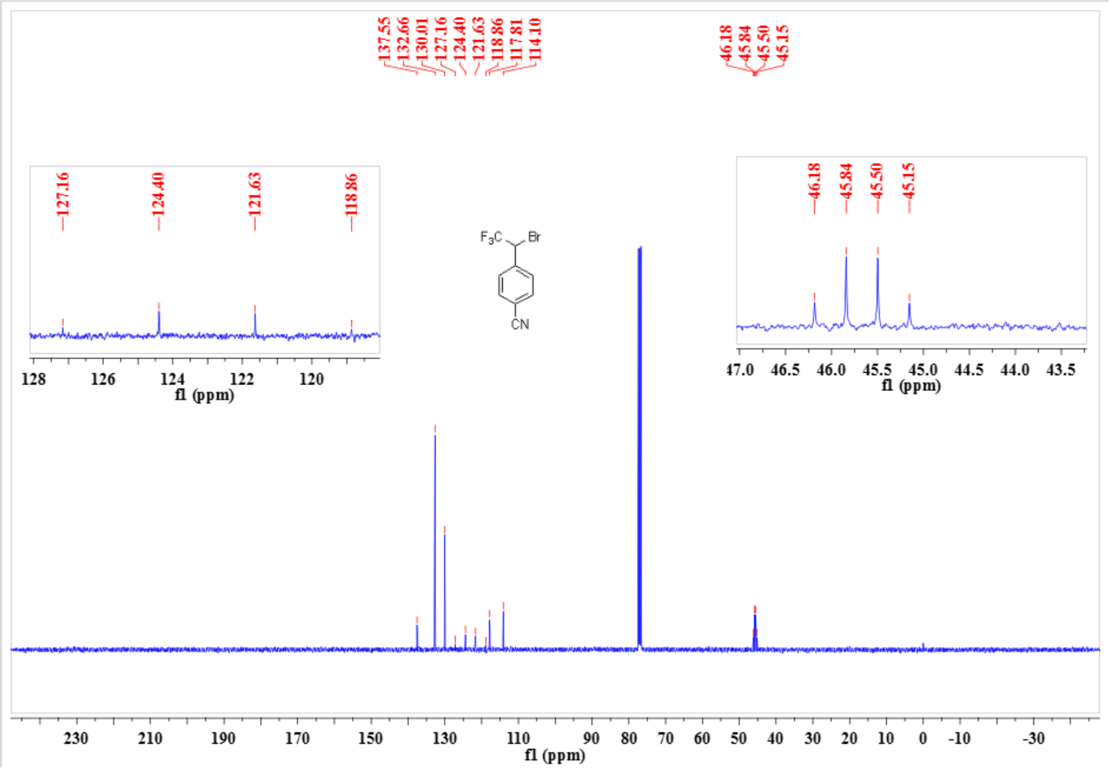


**Supplementary Figure 135.** $^{13}$**C NMR (101 MHz, $CDCl_3$) spectrum of 1b**

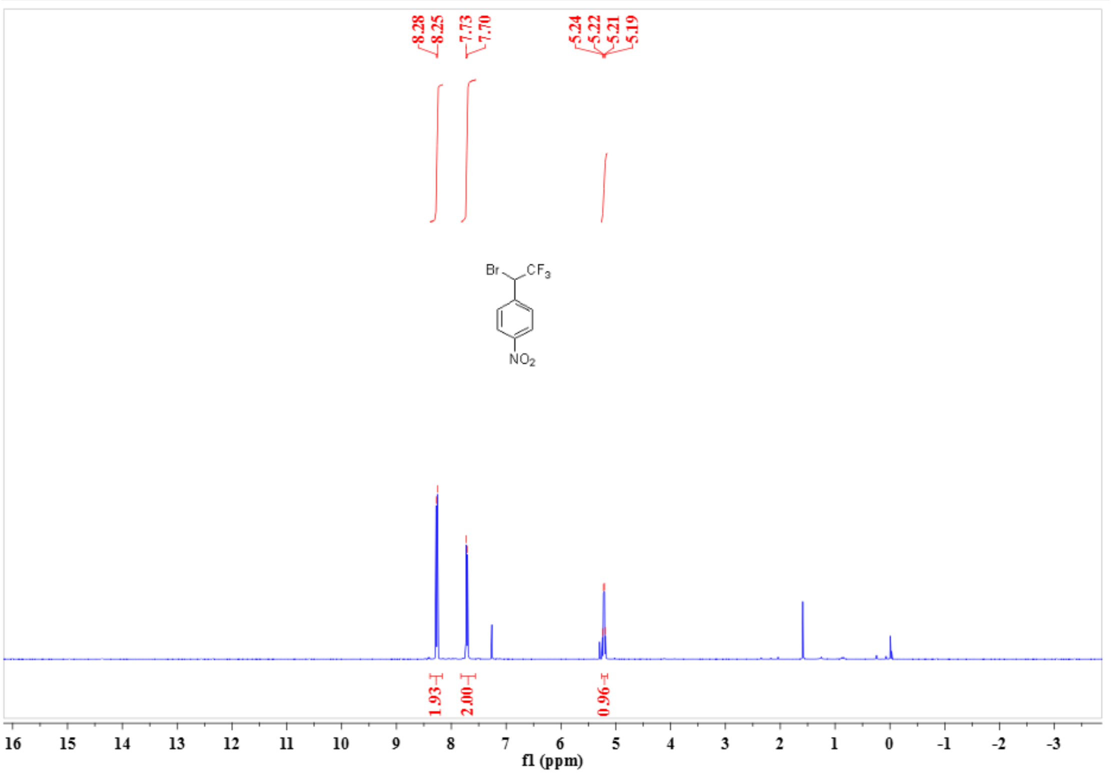


**Supplementary Figure 136.** $^{1}$**H NMR (400 MHz, $CDCl_3$) spectrum of 1c**

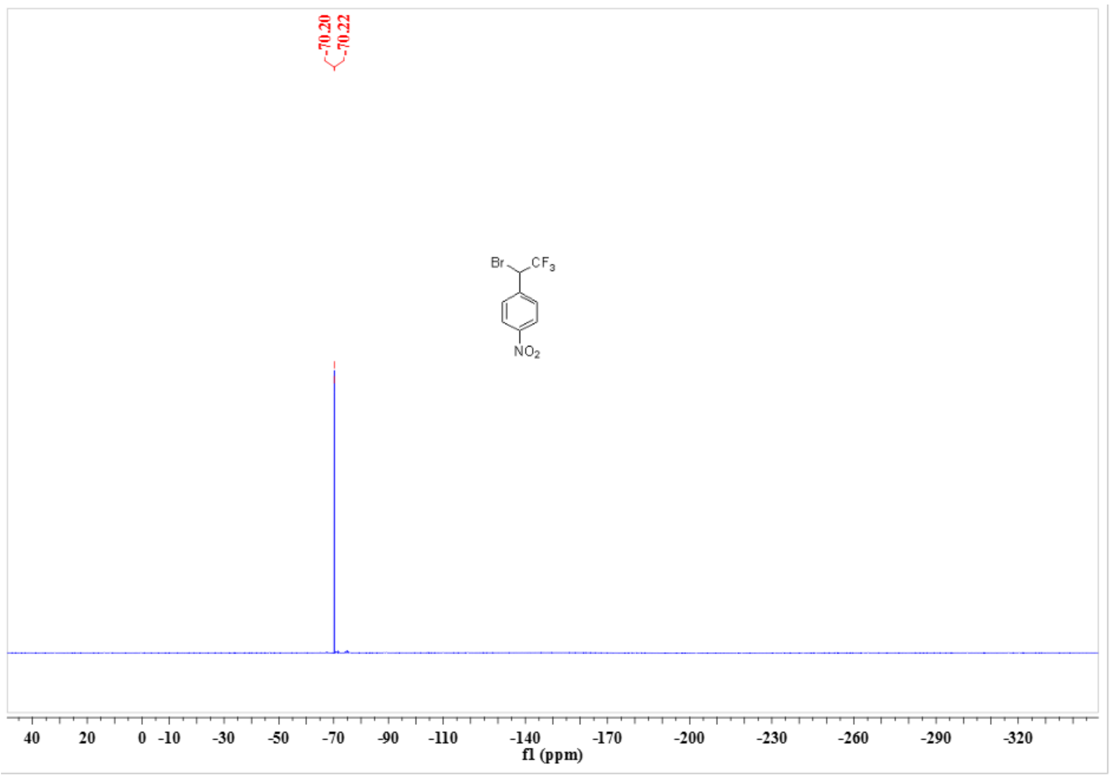


**Supplementary Figure 137.** $^{19}F$ **NMR (376 MHz, $CDCl_3$) spectrum of 1c**

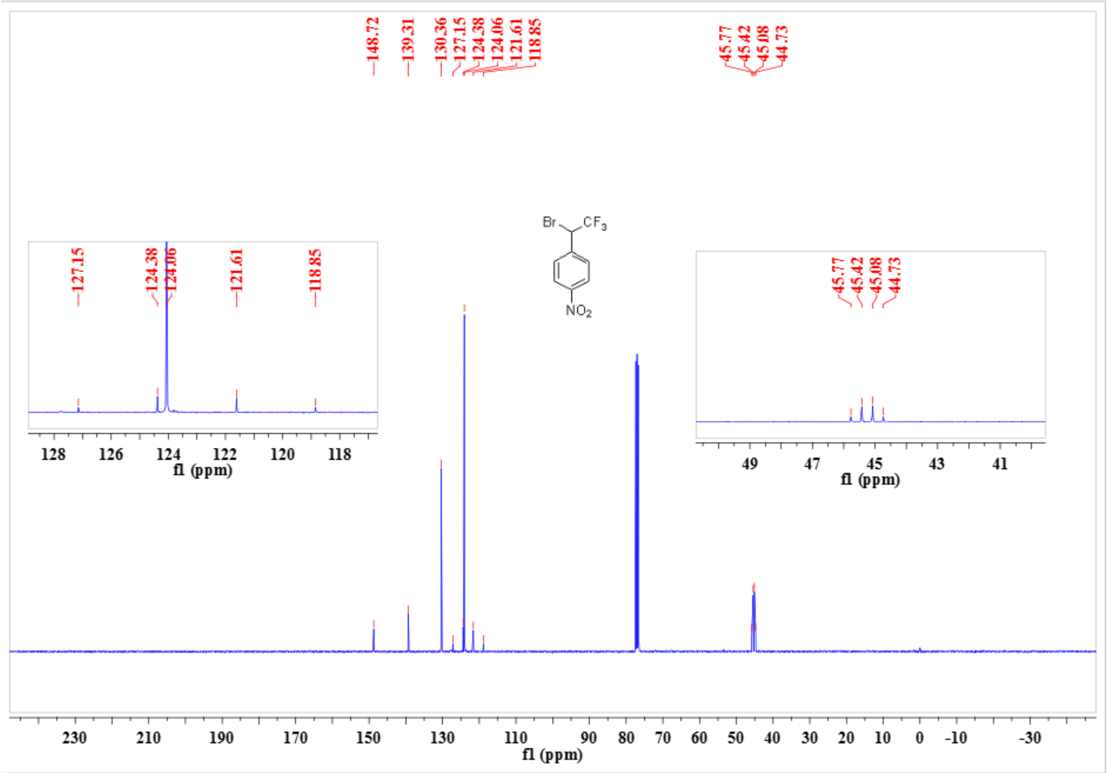


**Supplementary Figure 138.** $^{13}C$ **NMR (101 MHz, $CDCl_3$) spectrum of 1c**

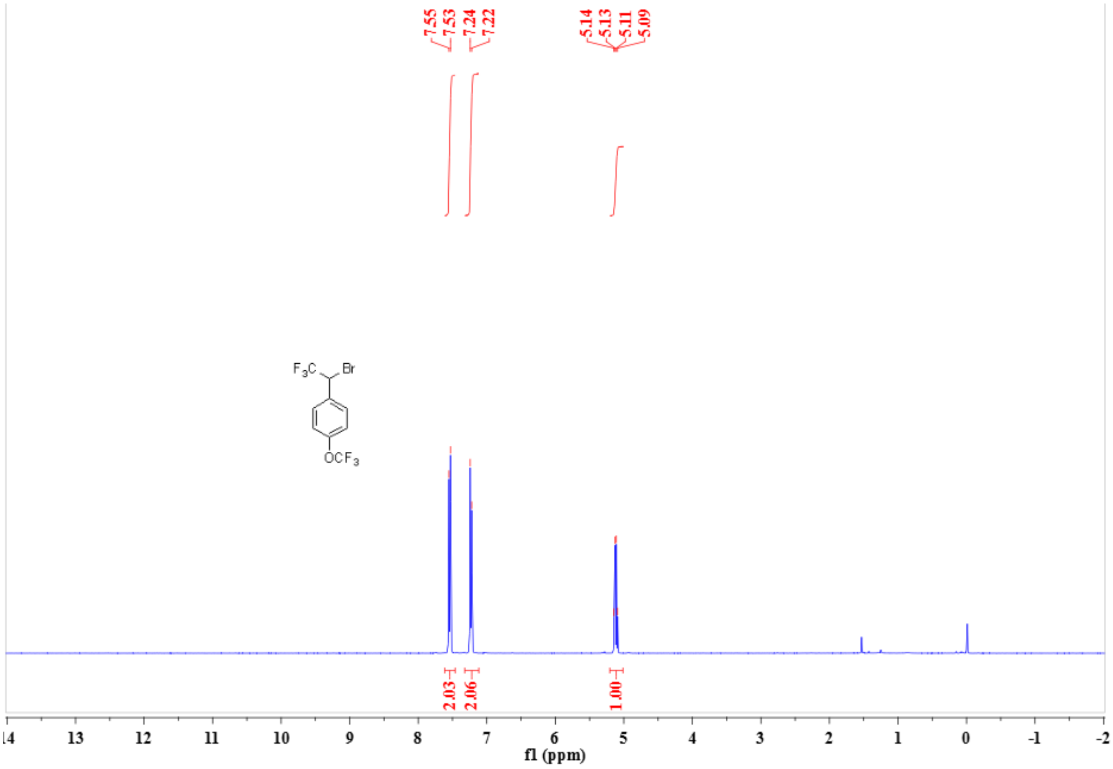


**Supplementary Figure 139.** **$^{1}$H NMR (400 MHz, $CDCl_3$) spectrum of 1d**

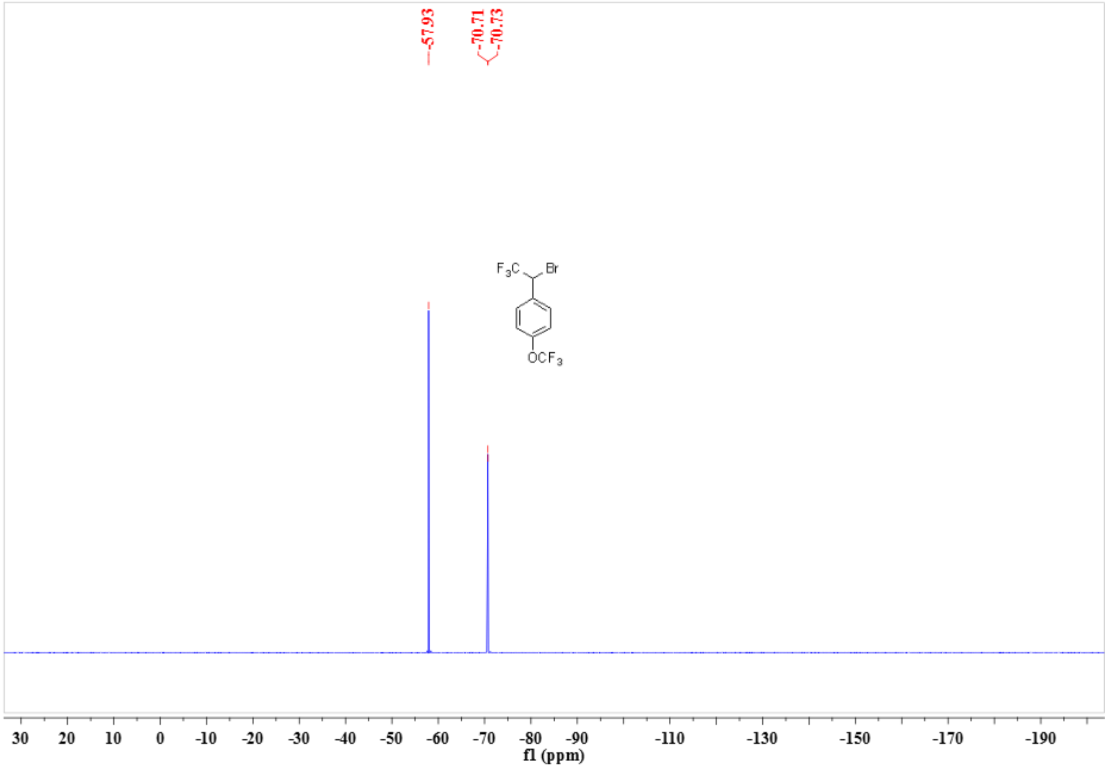


**Supplementary Figure 140.** **$^{19}$F NMR (376 MHz, $CDCl_3$) spectrum of 1d**

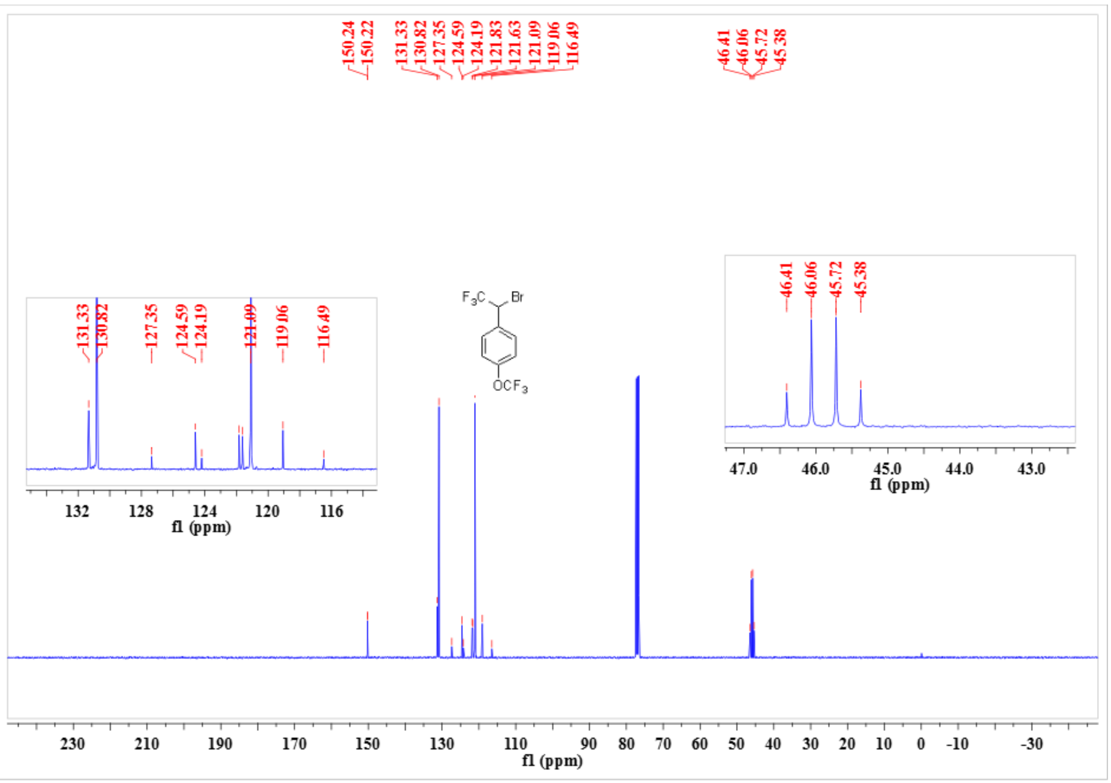

Supplementary Figure 141. $^{13}$C NMR (101 MHz, $CDCl_3$) spectrum of 1d

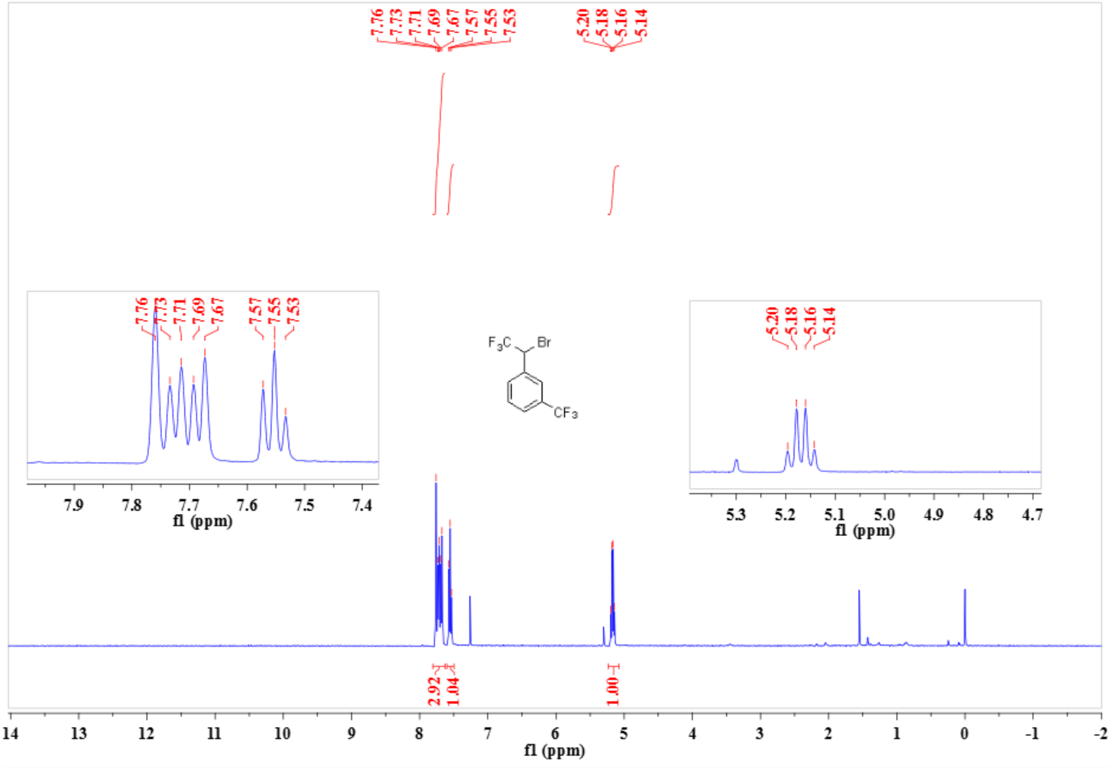

Supplementary Figure 142. $^{1}$H NMR (400 MHz, $CDCl_3$) spectrum of 1e

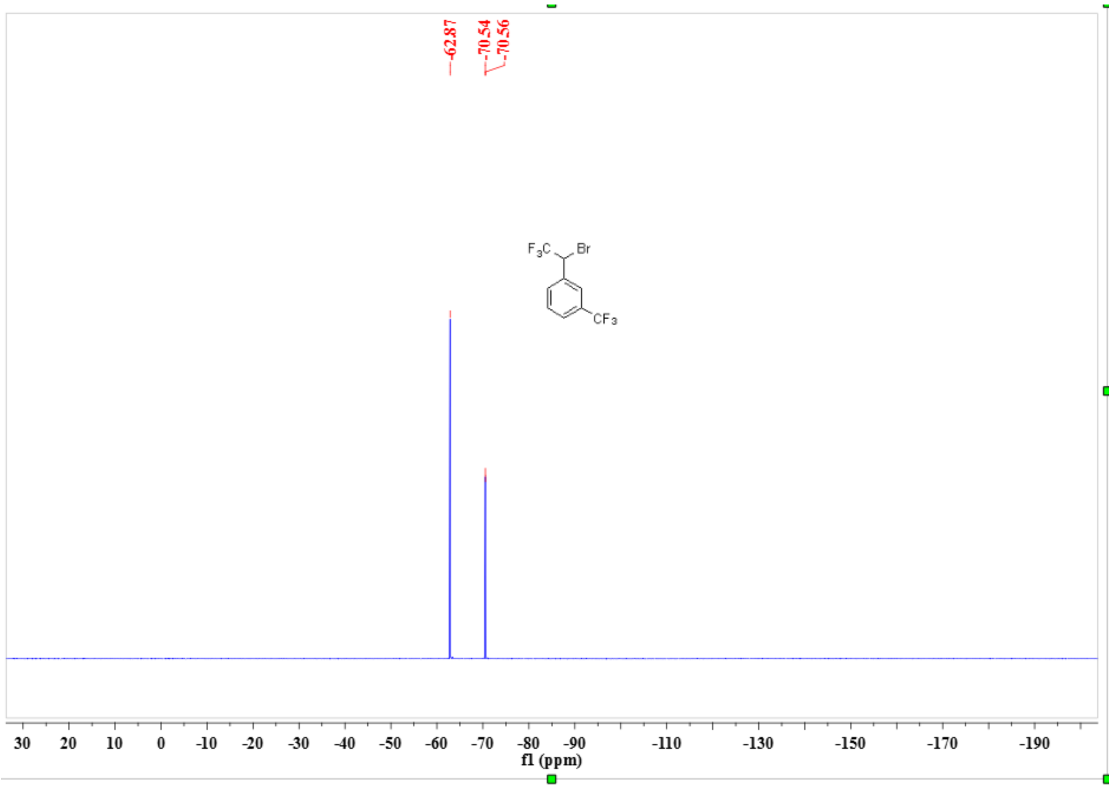


**Supplementary Figure 143.** $^{19}$F NMR (376 MHz, $CDCl_3$) spectrum of **1e**

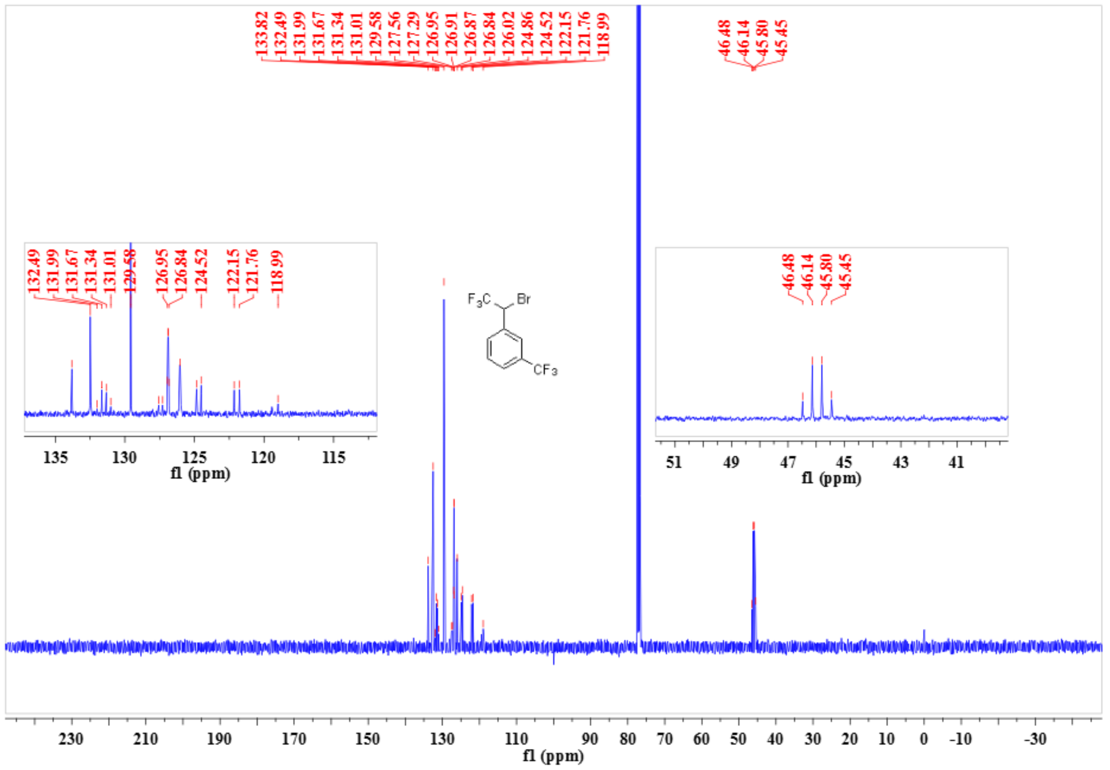


**Supplementary Figure 144.** $^{13}$C NMR (101 MHz, $CDCl_3$) spectrum of **1e**

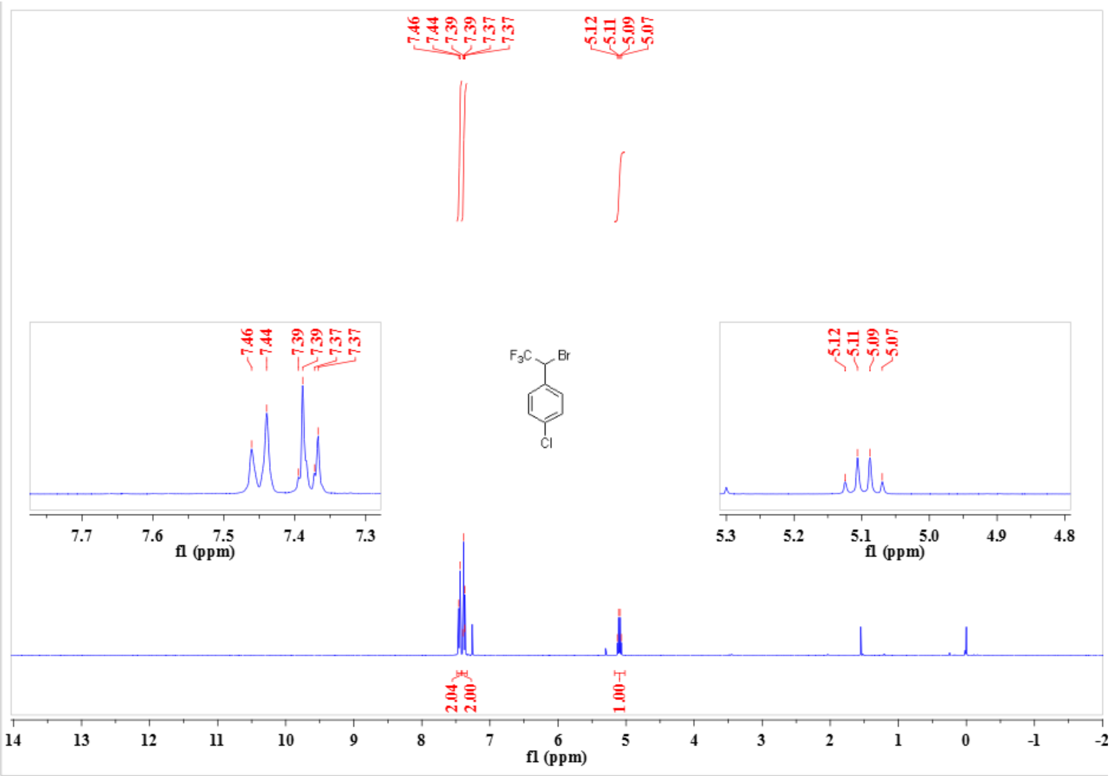


**Supplementary Figure 145.** $^{1}H$ **NMR (400 MHz, $CDCl_3$) spectrum of 1f**

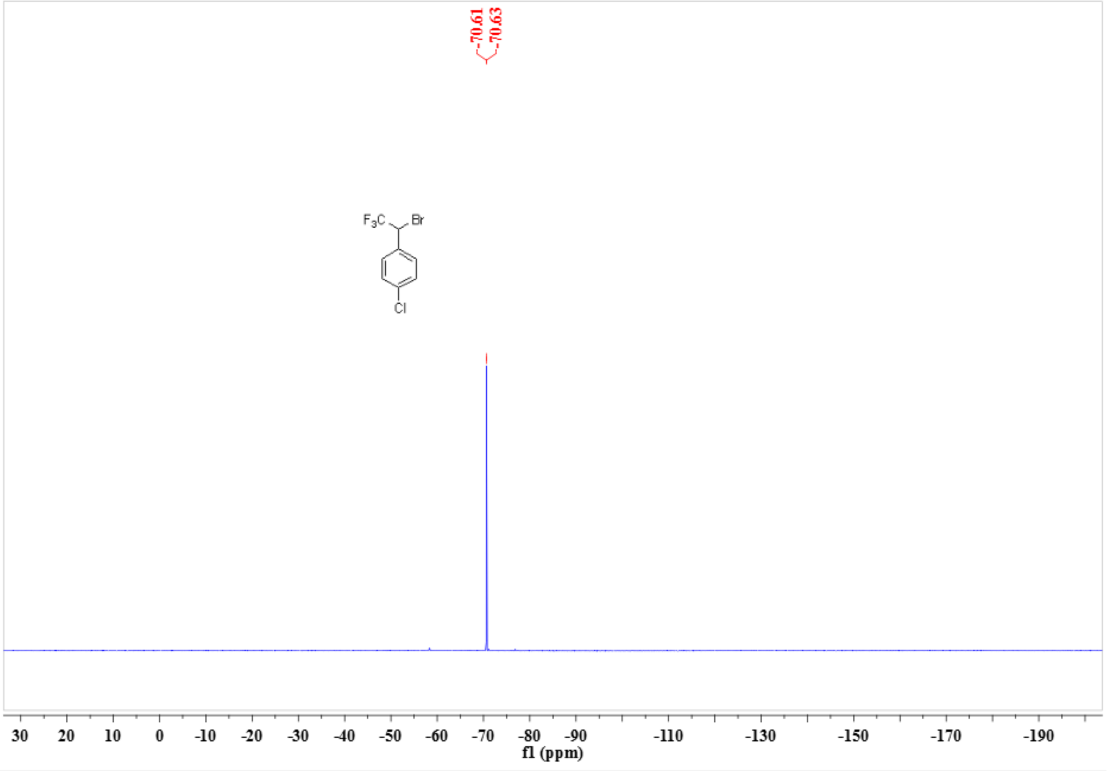


**Supplementary Figure 146.** $^{19}F$ **NMR (376 MHz, $CDCl_3$) spectrum of 1f**

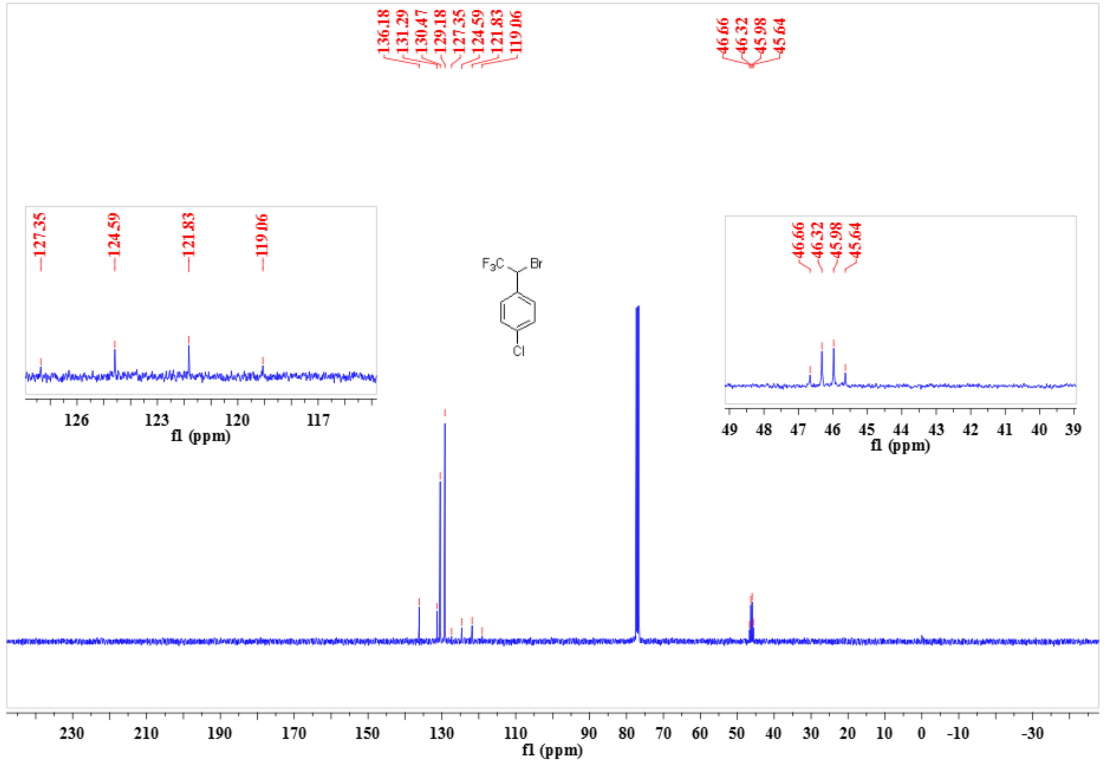


**Supplementary Figure 147.** $^{13}$**C NMR (101 MHz, CDCl$_3$) spectrum of 1f**

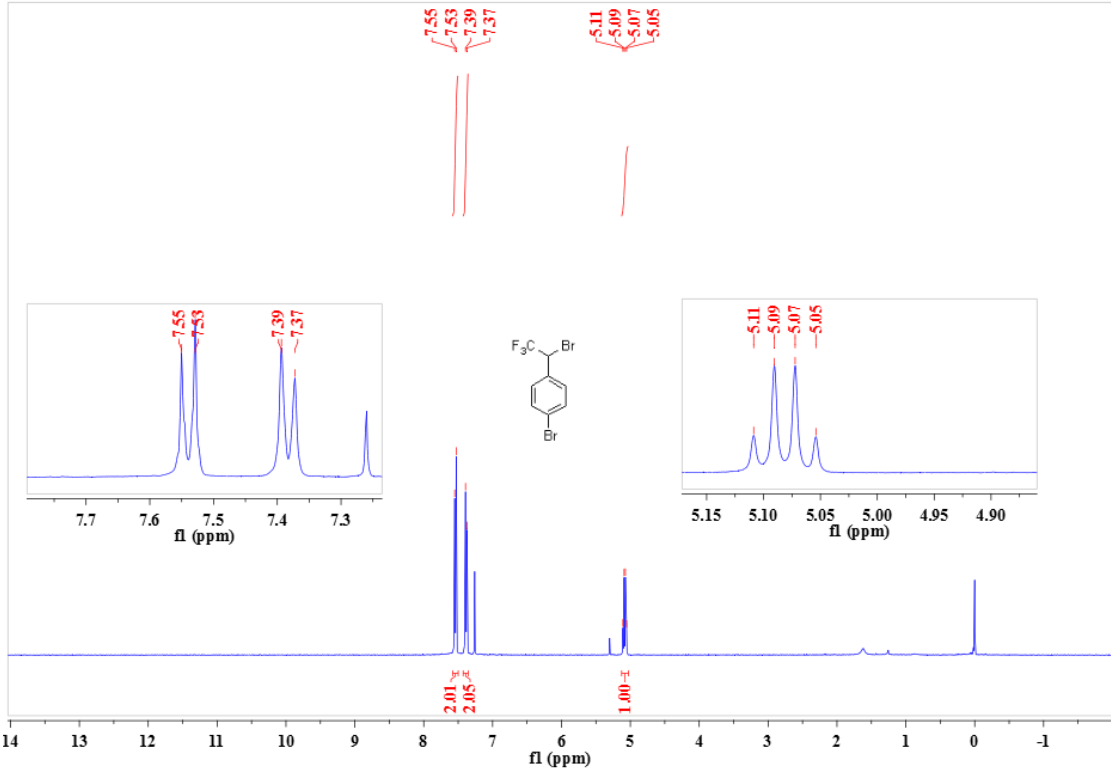


**Supplementary Figure 148.** $^{1}$**H NMR (400 MHz, CDCl$_3$) spectrum of 1g**

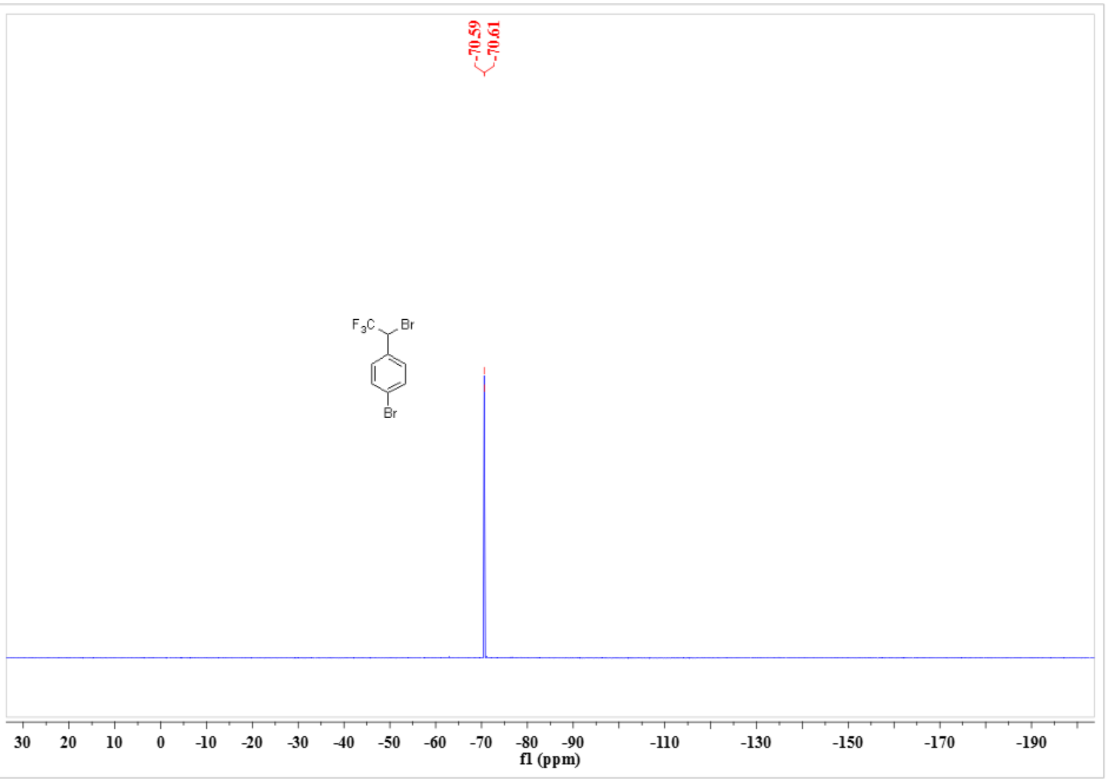


**Supplementary Figure 149.** $^{19}$**F NMR (376 MHz, CDCl**$_3$**) spectrum of 1g**

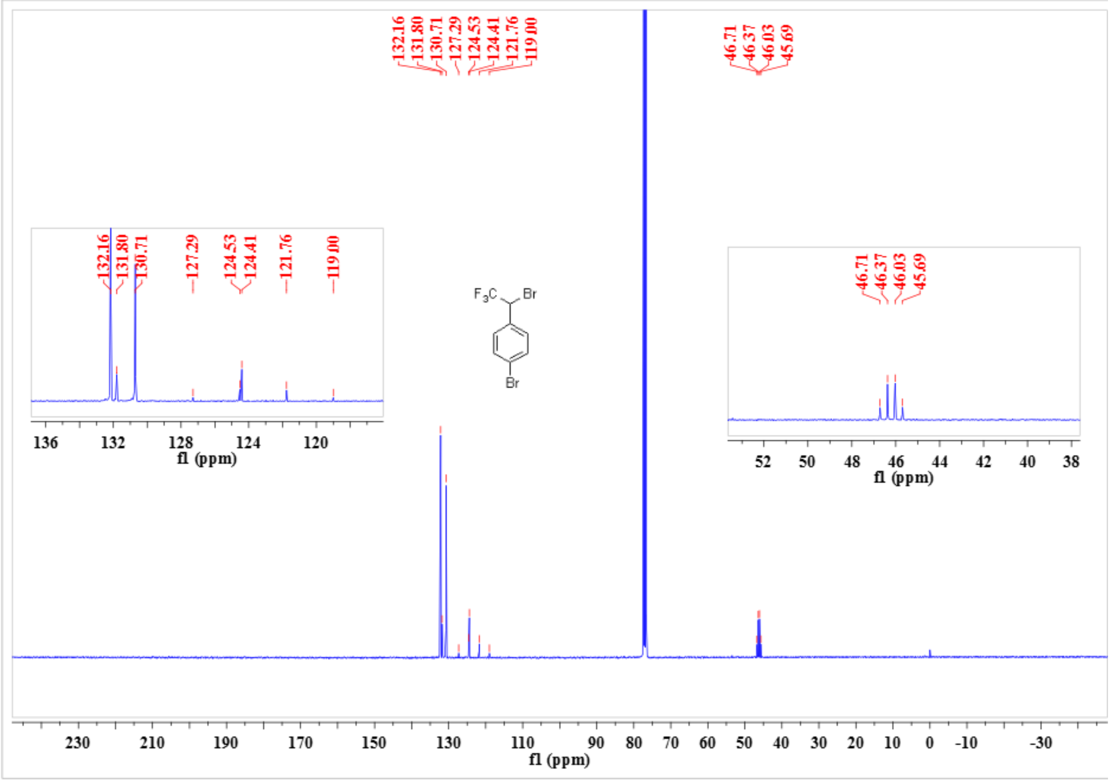


**Supplementary Figure 150.** $^{13}$**C NMR (101 MHz, CDCl**$_3$**) spectrum of 1g**

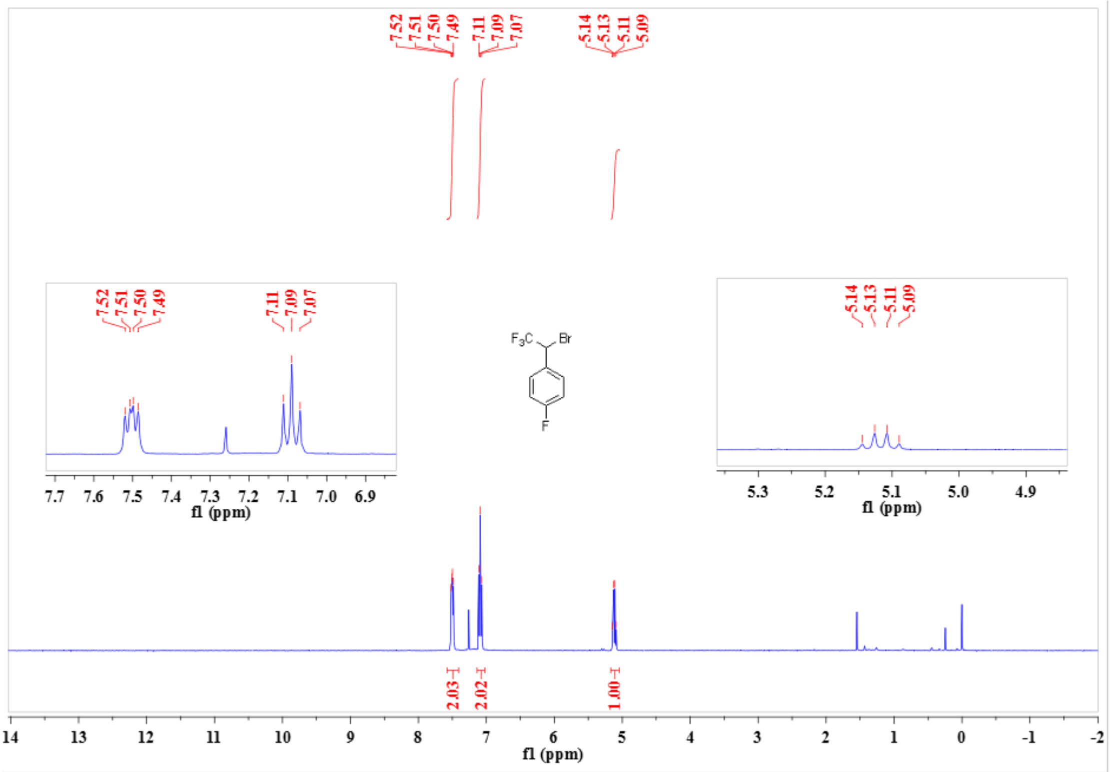


Supplementary Figure 151. $^{1}$H NMR (400 MHz, $CDCl_3$) spectrum of 1h

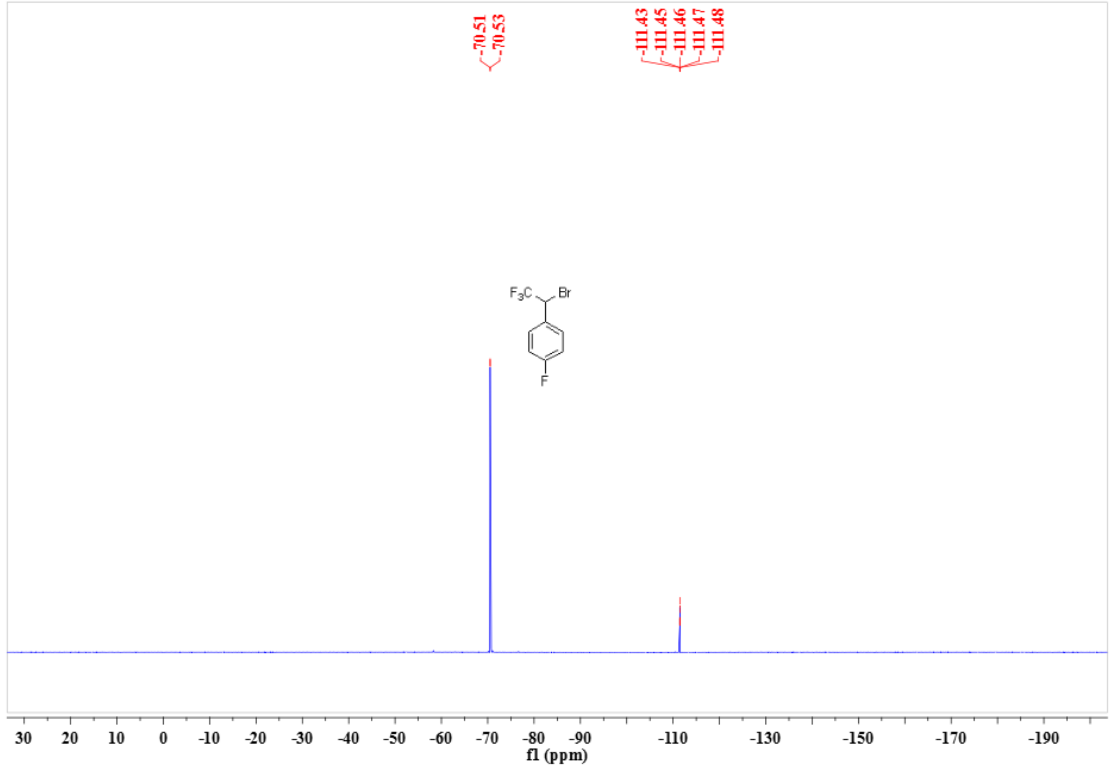


Supplementary Figure 152. $^{19}$F NMR (376 MHz, $CDCl_3$) spectrum of 1h

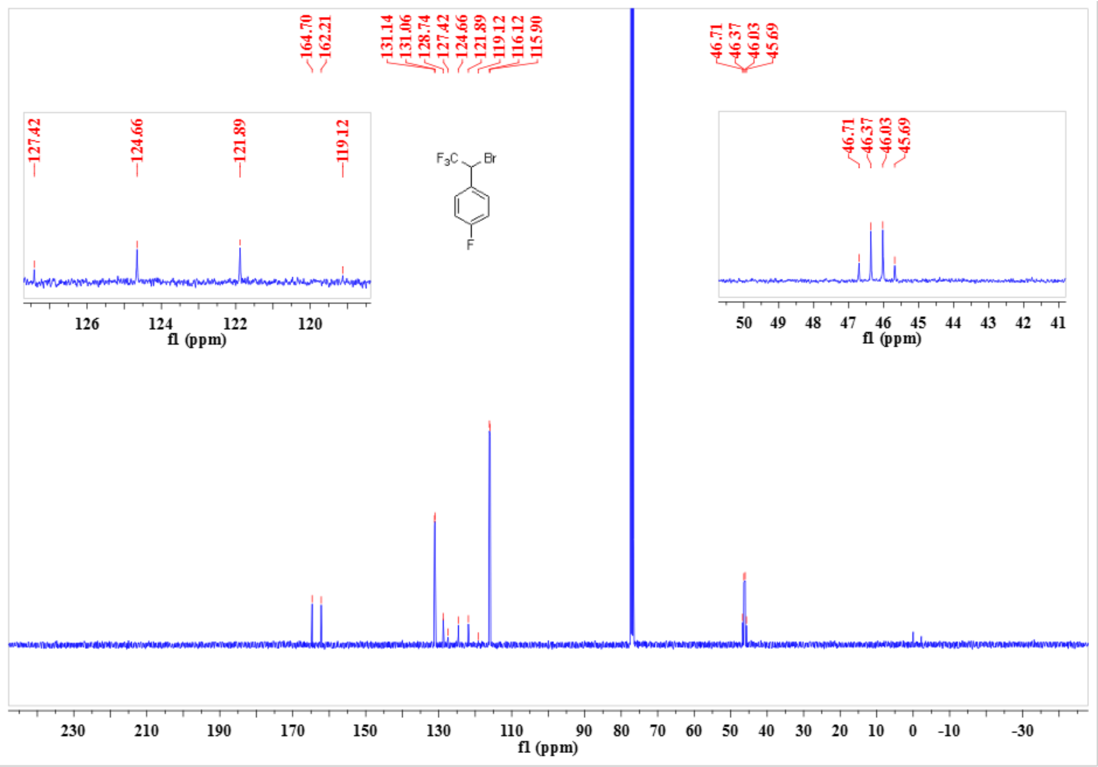

Supplementary Figure 153. $^{13}$C NMR (101 MHz, $CDCl_3$) spectrum of 1h

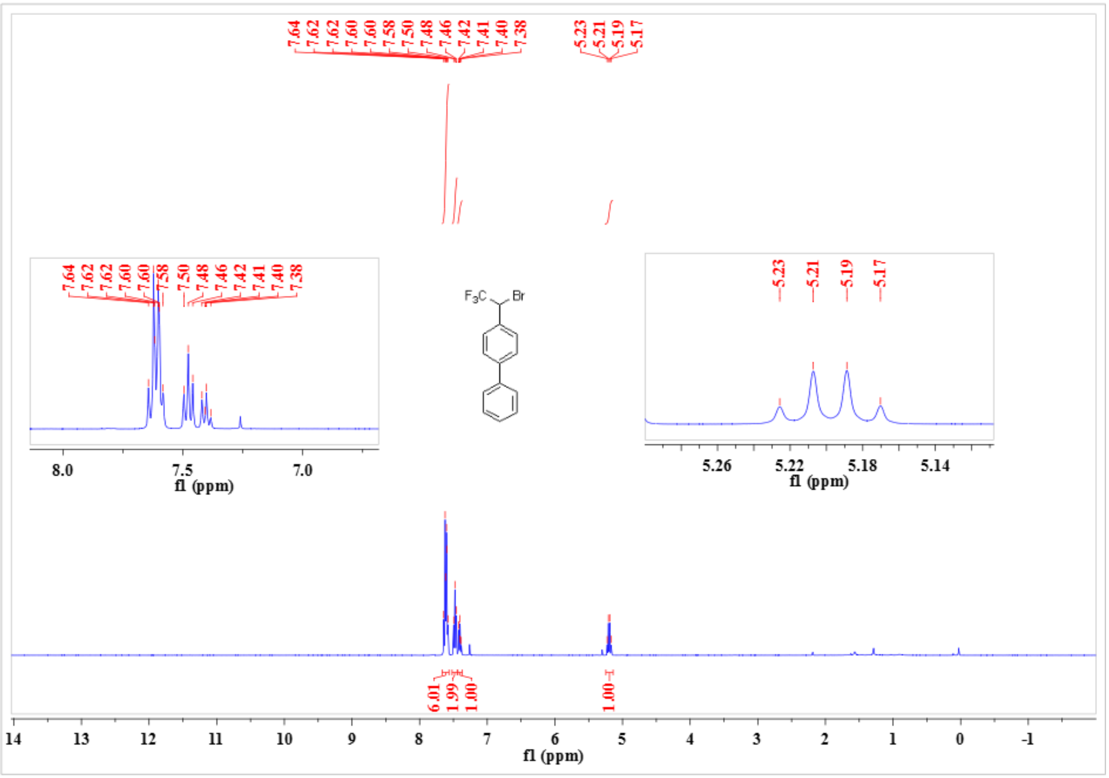

Supplementary Figure 154. $^{1}$H NMR (400 MHz, $CDCl_3$) spectrum of 1i

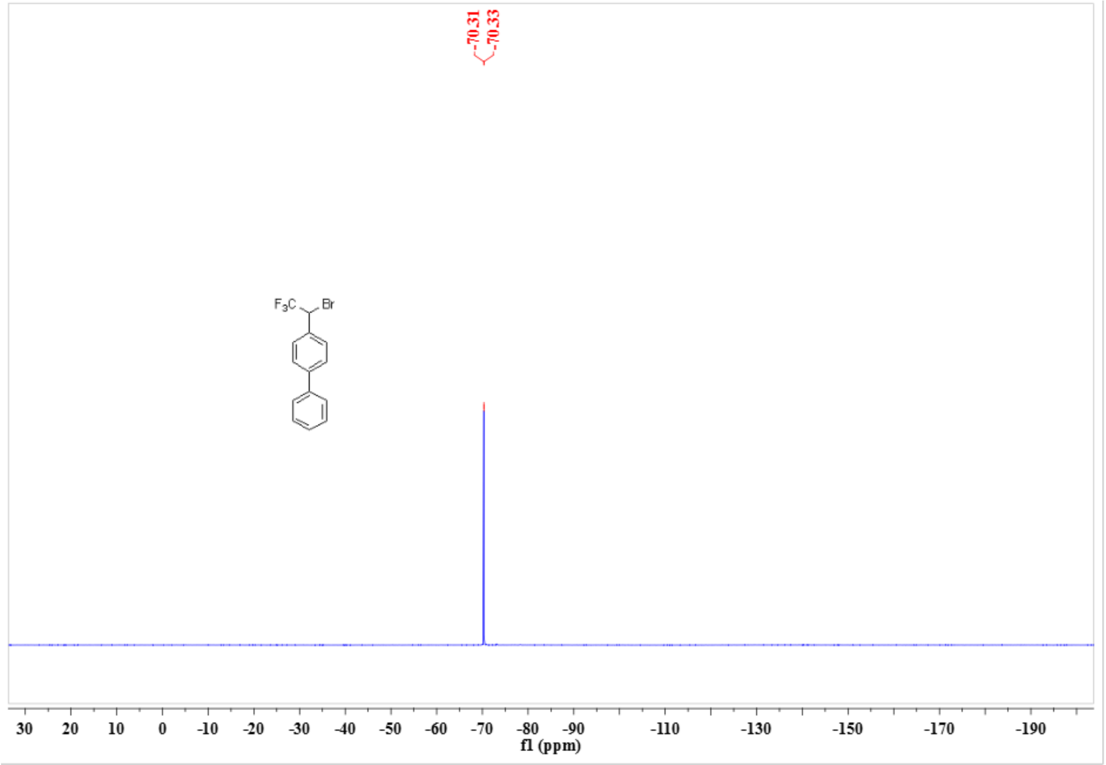

**Supplementary Figure 155. $^{19}$F NMR (376 MHz, $CDCl_3$) spectrum of 1i**

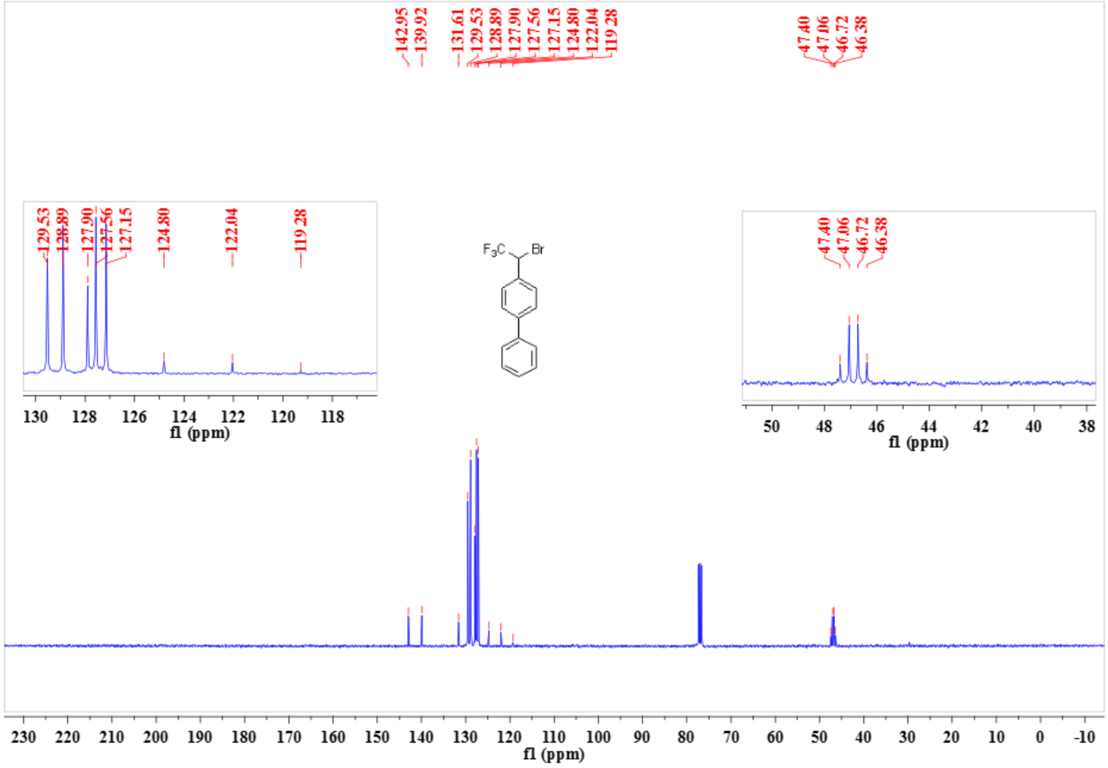

**Supplementary Figure 156. $^{13}$C NMR (101 MHz, $CDCl_3$) spectrum of 1i**

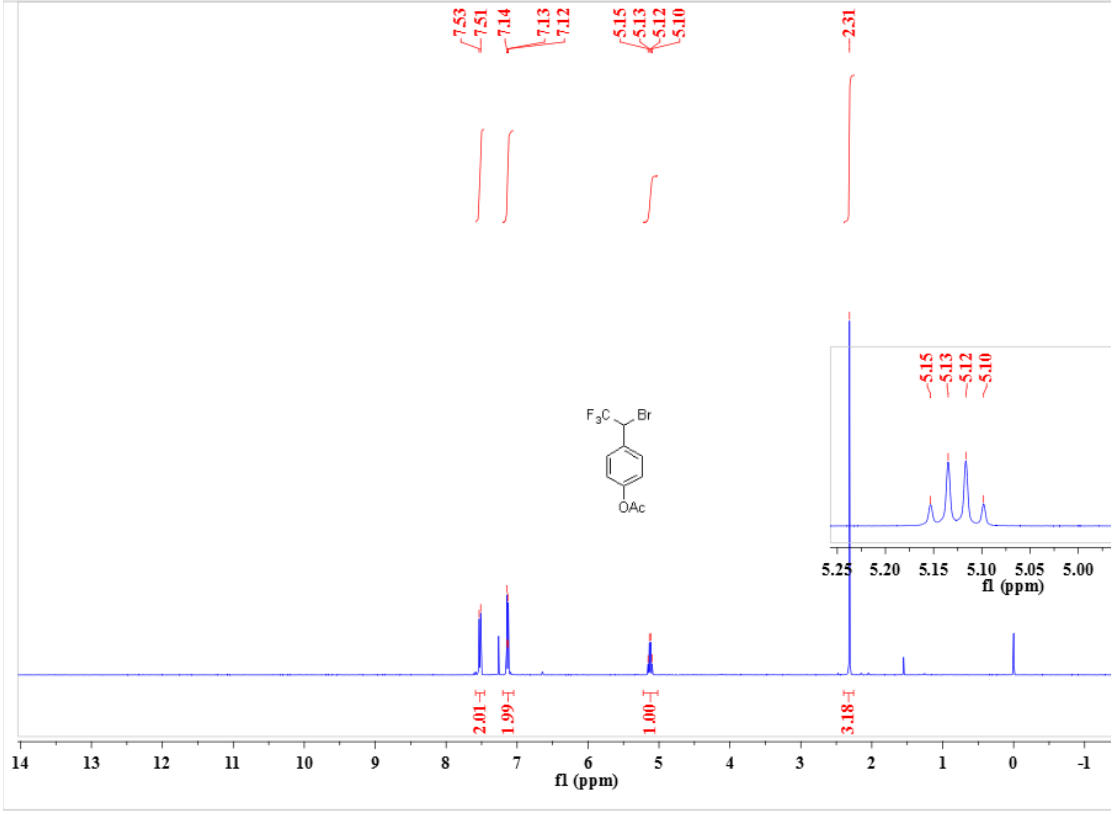


**Supplementary Figure 157. $^{1}$H NMR (400 MHz, CDCl$_3$) spectrum of 1j**

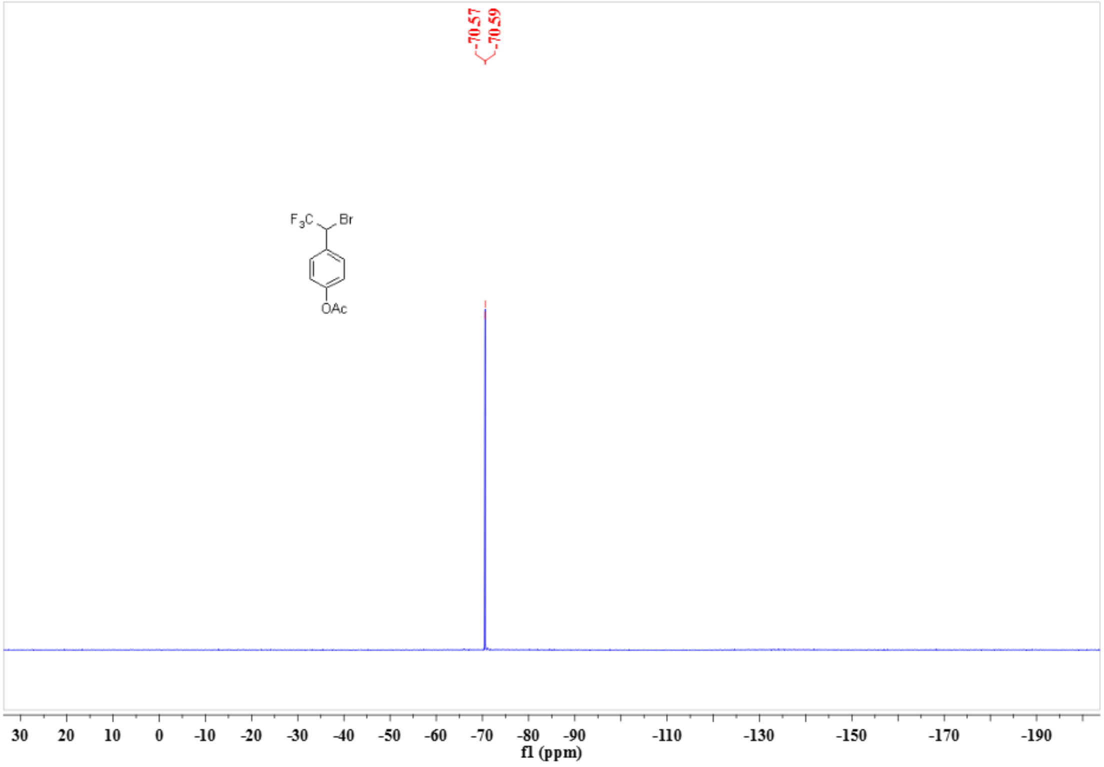


**Supplementary Figure 158. $^{19}$F NMR (376 MHz, CDCl$_3$) spectrum of 1j**

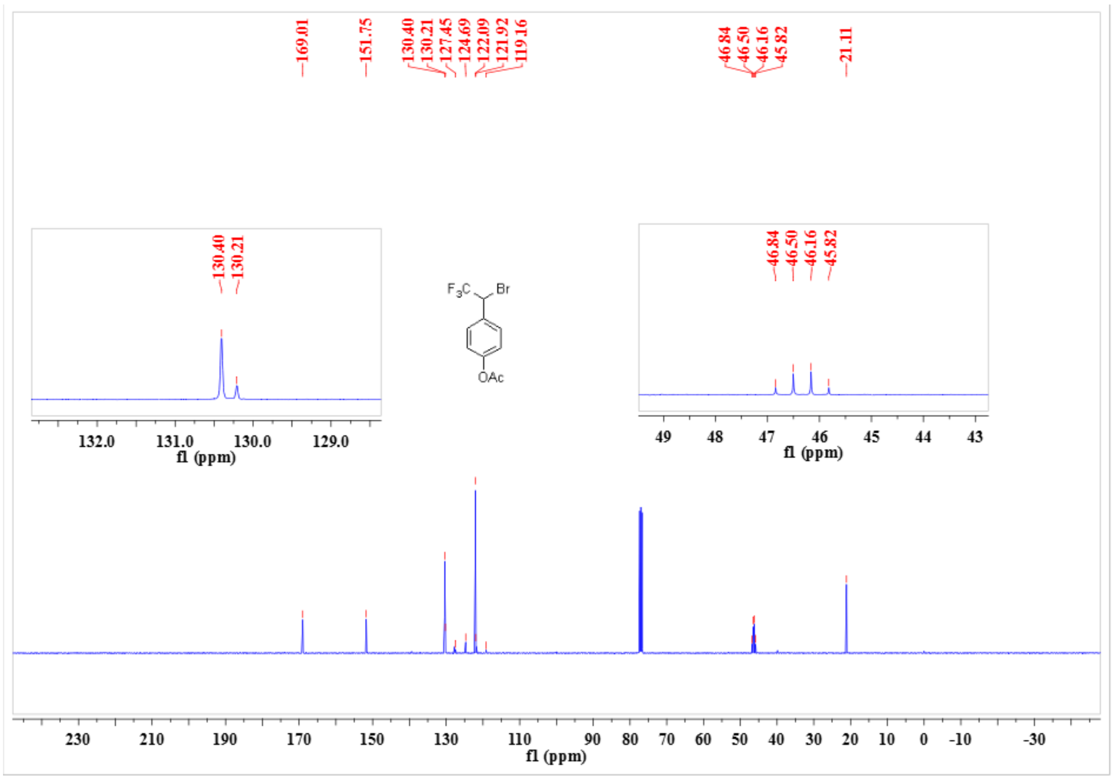


**Supplementary Figure 159.** $^{13}C$ **NMR (101 MHz,** $CDCl_3$**) spectrum of 1j**

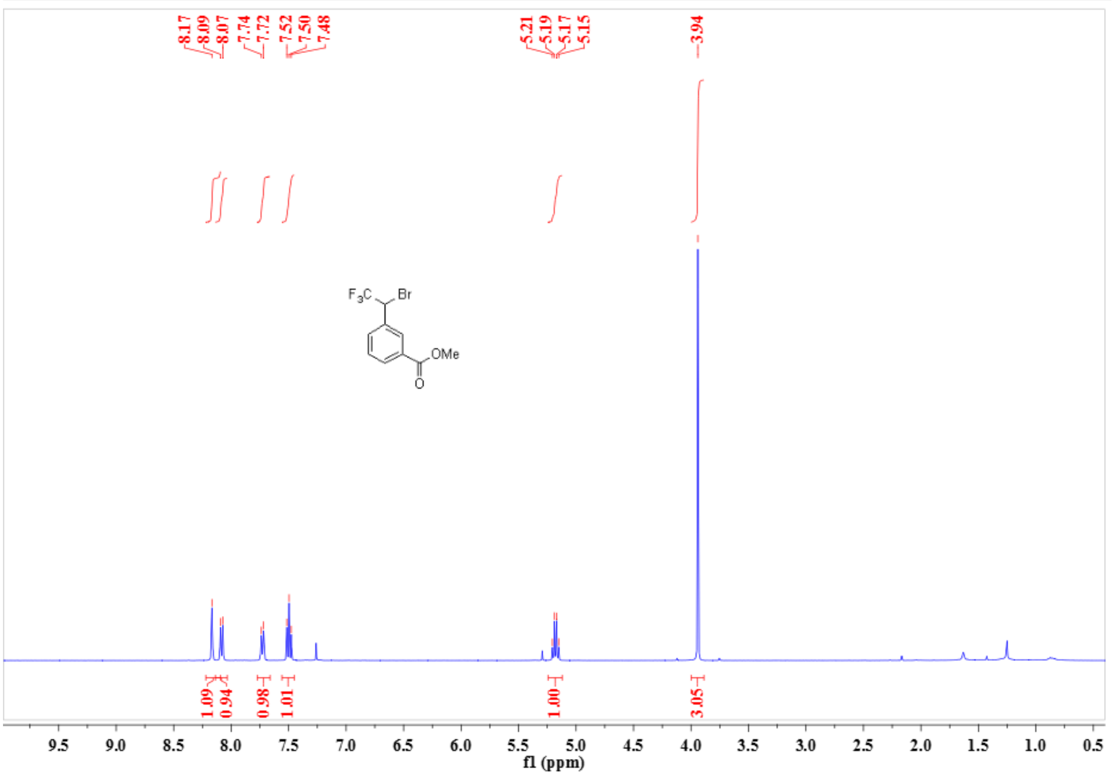


**Supplementary Figure 160.** $^{1}H$ **NMR (400 MHz,** $CDCl_3$**) spectrum of 1k**

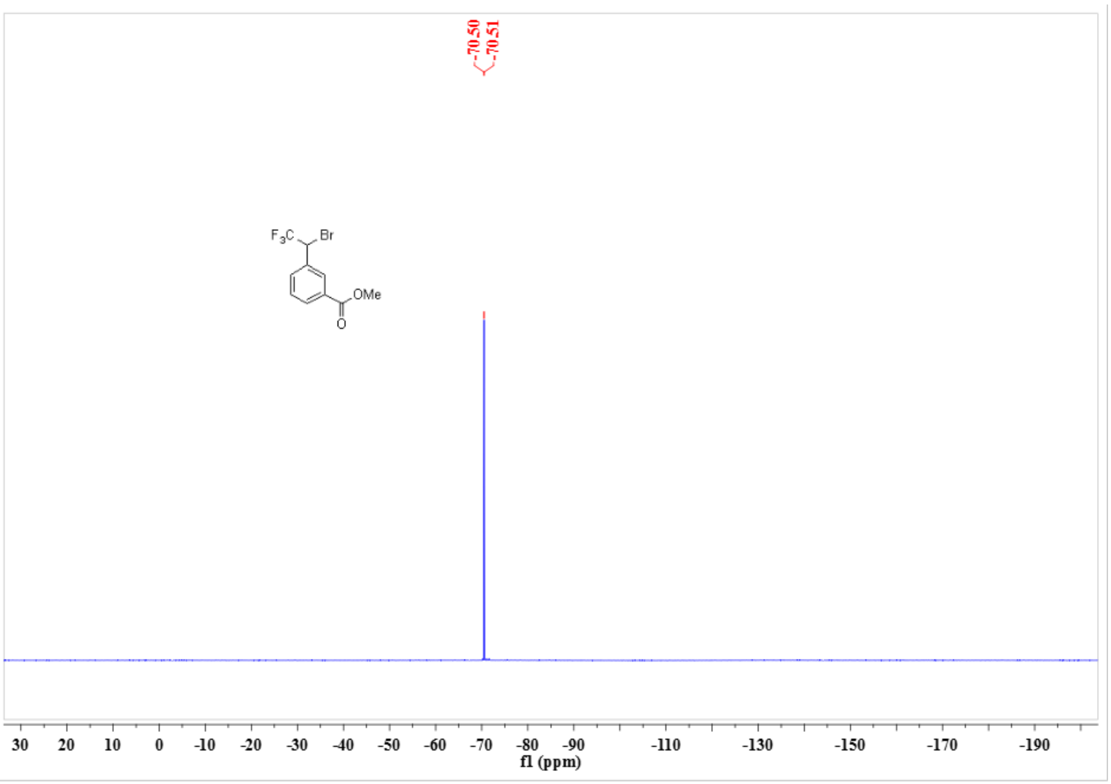


**Supplementary Figure 161. $^{19}$F NMR (376 MHz, $CDCl_3$) spectrum of 1k**

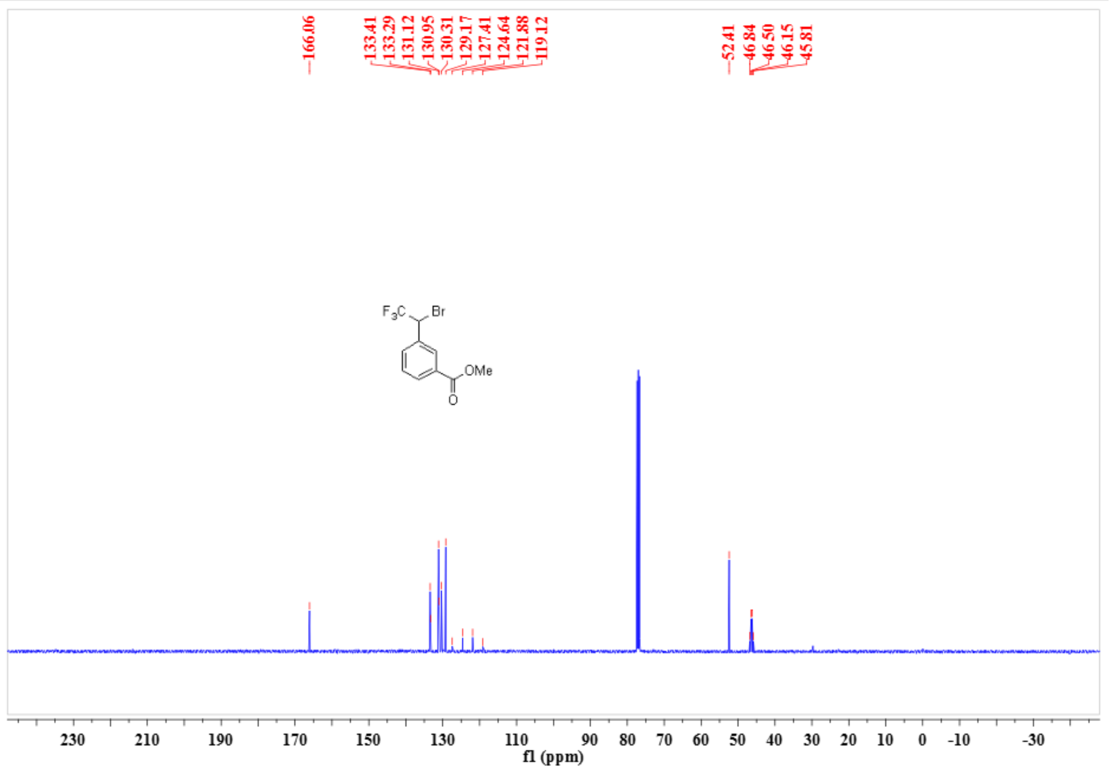


**Supplementary Figure 162. $^{13}$C NMR (101 MHz, $CDCl_3$) spectrum of 1k**

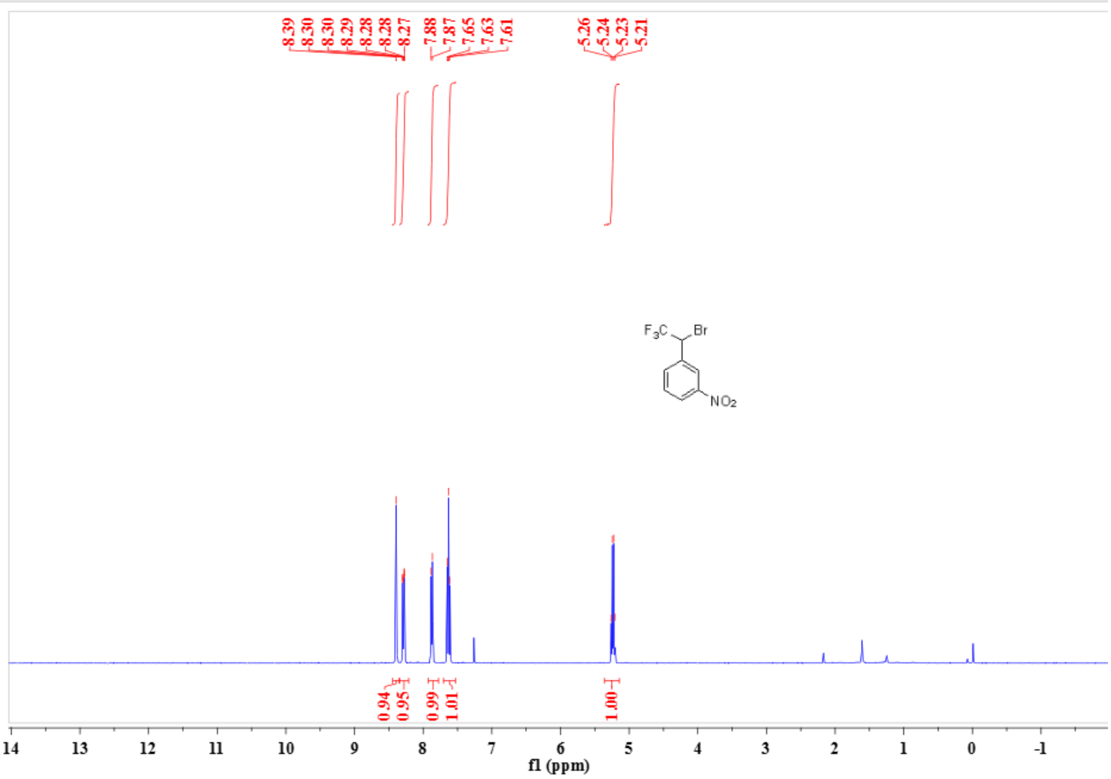


Supplementary Figure 163. $^{1}$H NMR (400 MHz, $CDCl_3$) spectrum of 1l

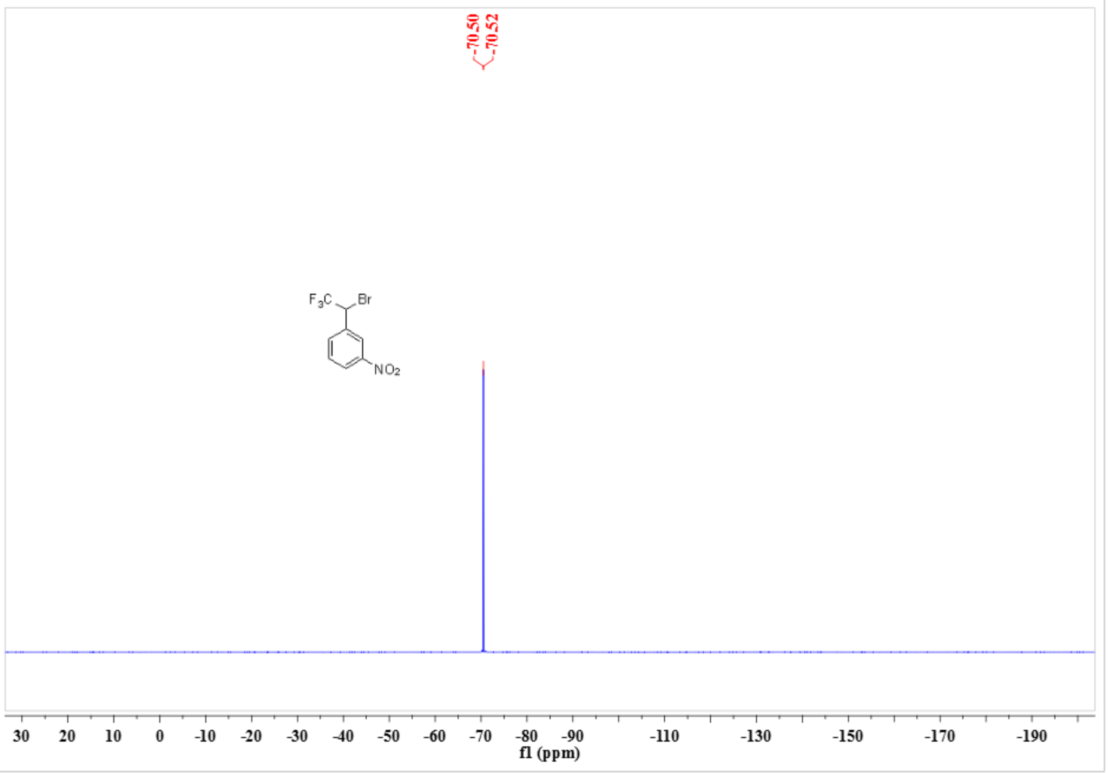


Supplementary Figure 164. $^{19}$F NMR (376 MHz, $CDCl_3$) spectrum of 1l

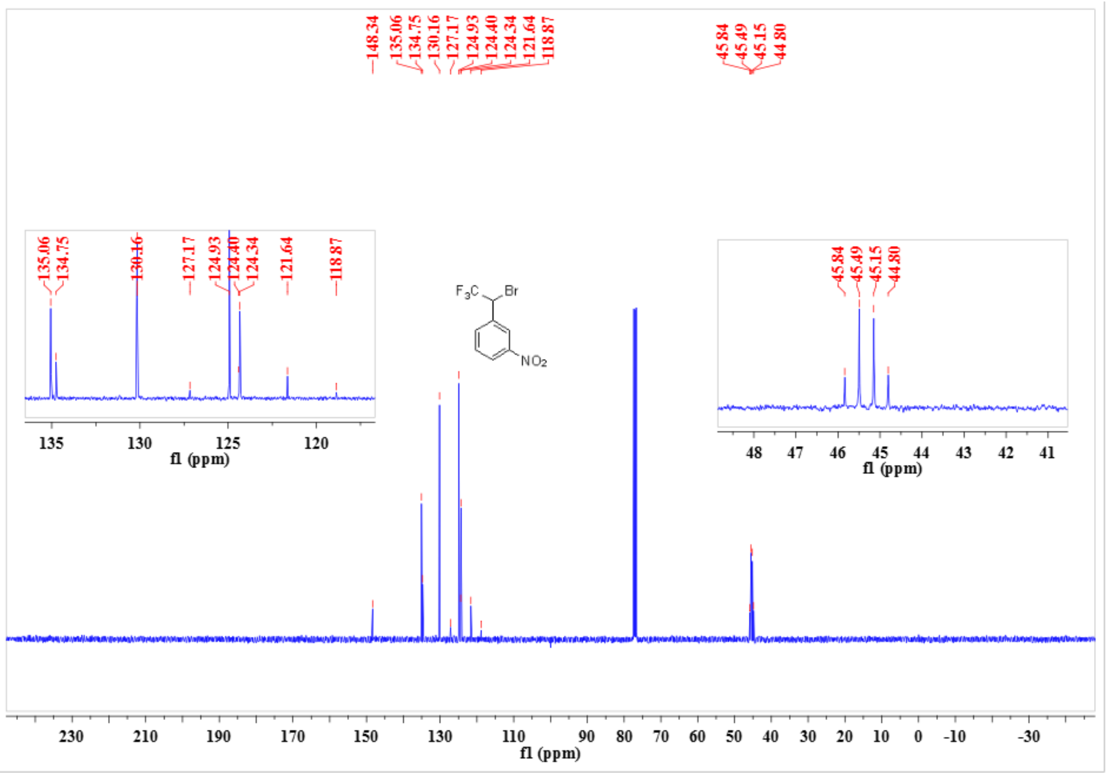

Supplementary Figure 165. $^{13}$C NMR (101 MHz, $CDCl_3$) spectrum of 1l

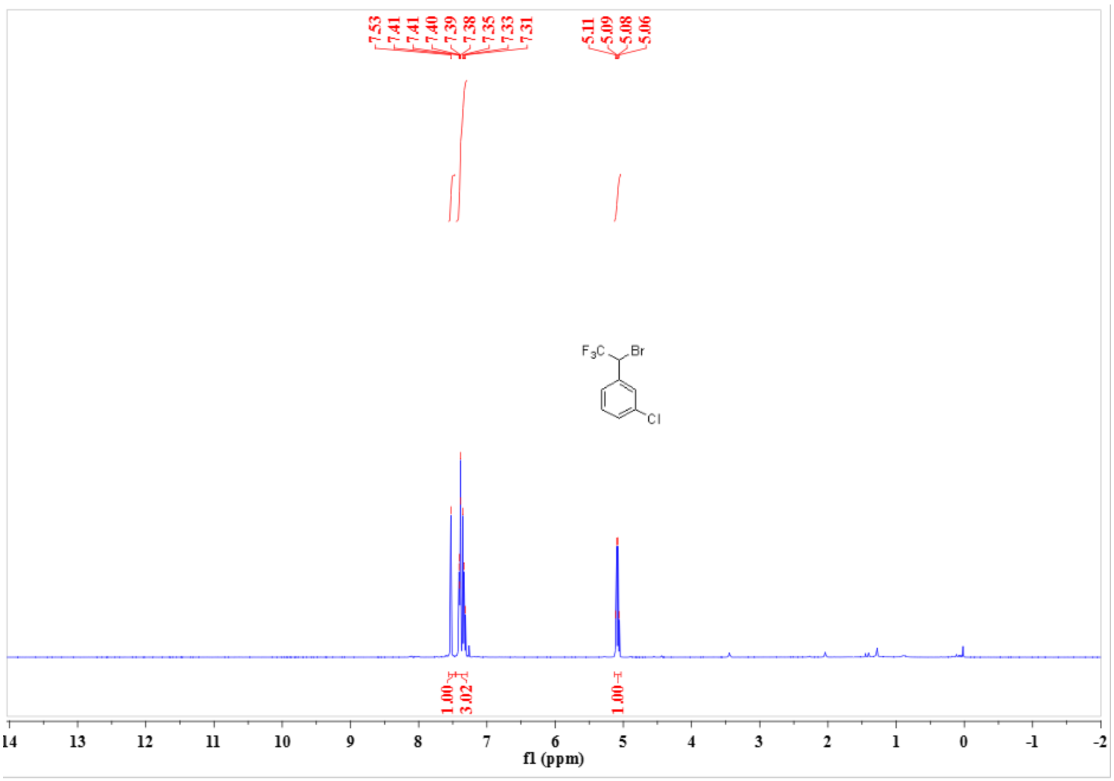

Supplementary Figure 166. $^{1}$H NMR (400 MHz, $CDCl_3$) spectrum of 1m

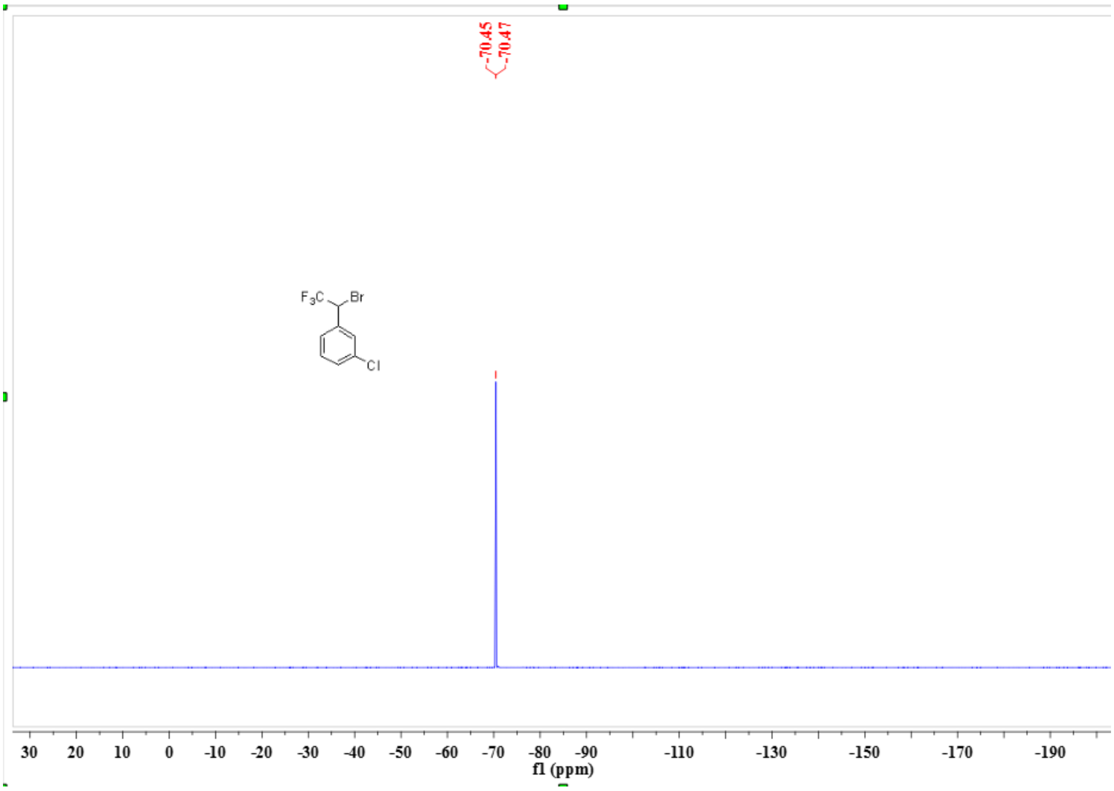


Supplementary Figure 167. $^{19}$F NMR (376 MHz, $CDCl_3$) spectrum of 1m

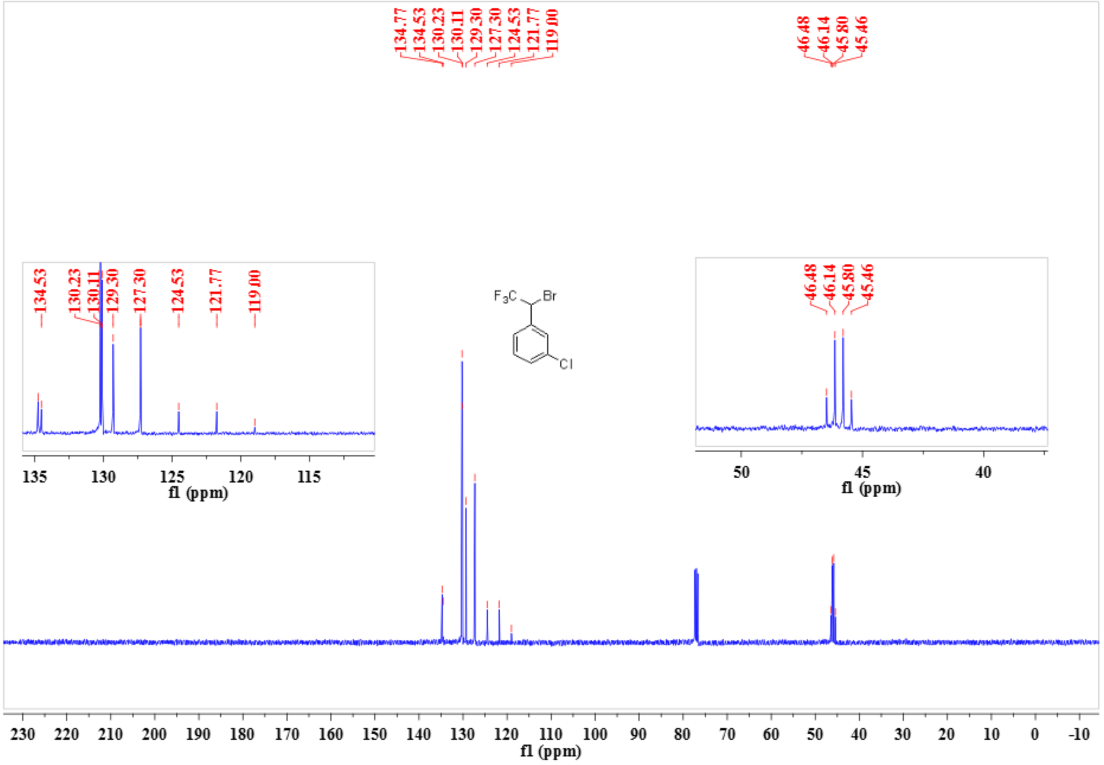


Supplementary Figure 168. $^{13}$C NMR (101 MHz, $CDCl_3$) spectrum of 1m

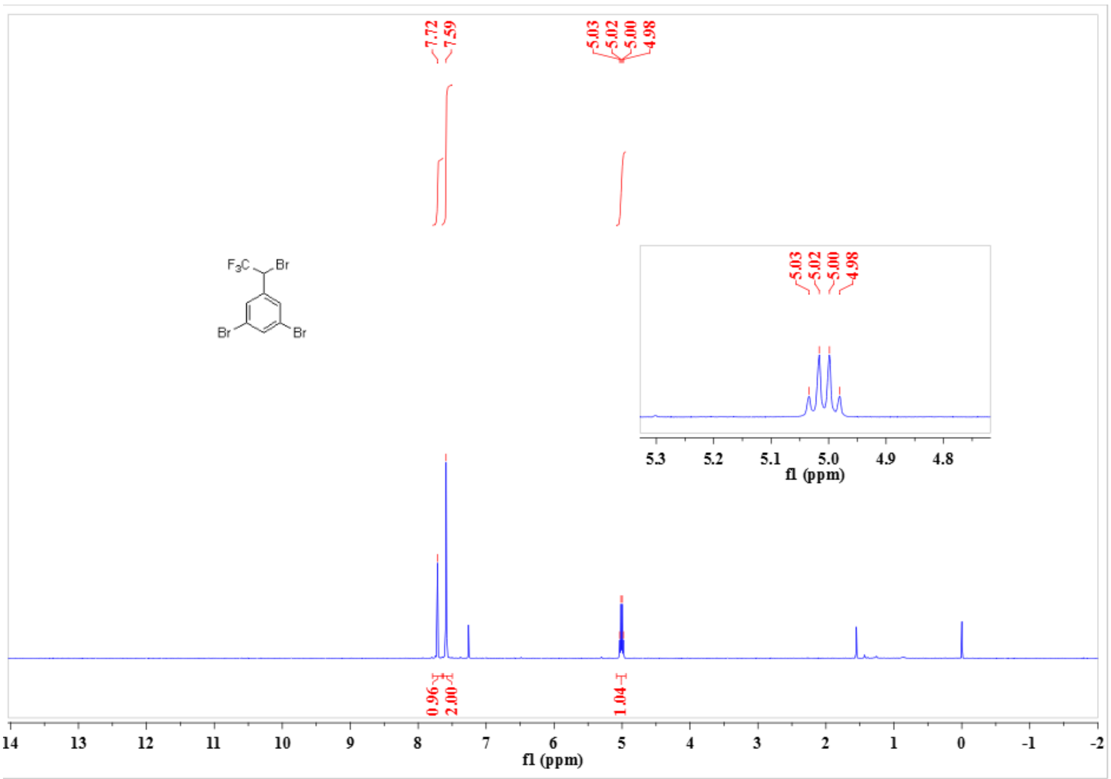


**Supplementary Figure 169. $^{1}$H NMR (400 MHz, $CDCl_3$) spectrum of 1n**

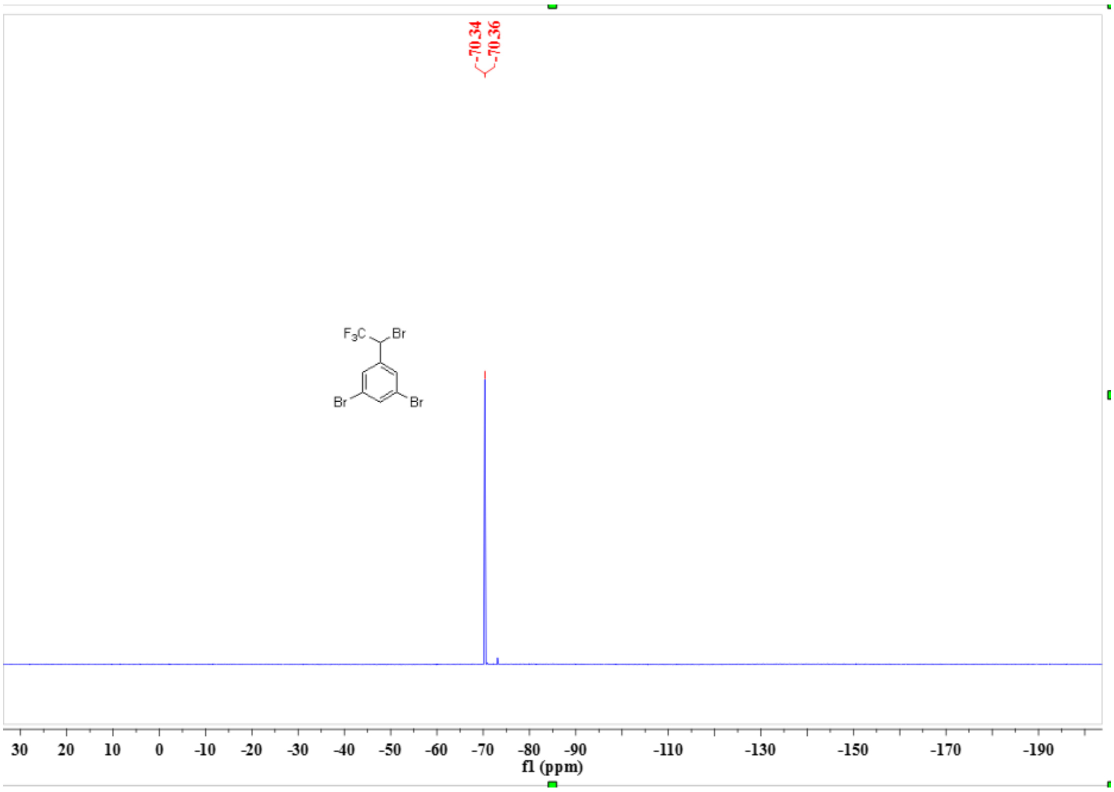


**Supplementary Figure 170. $^{19}$F NMR (376 MHz, $CDCl_3$) spectrum of 1n**

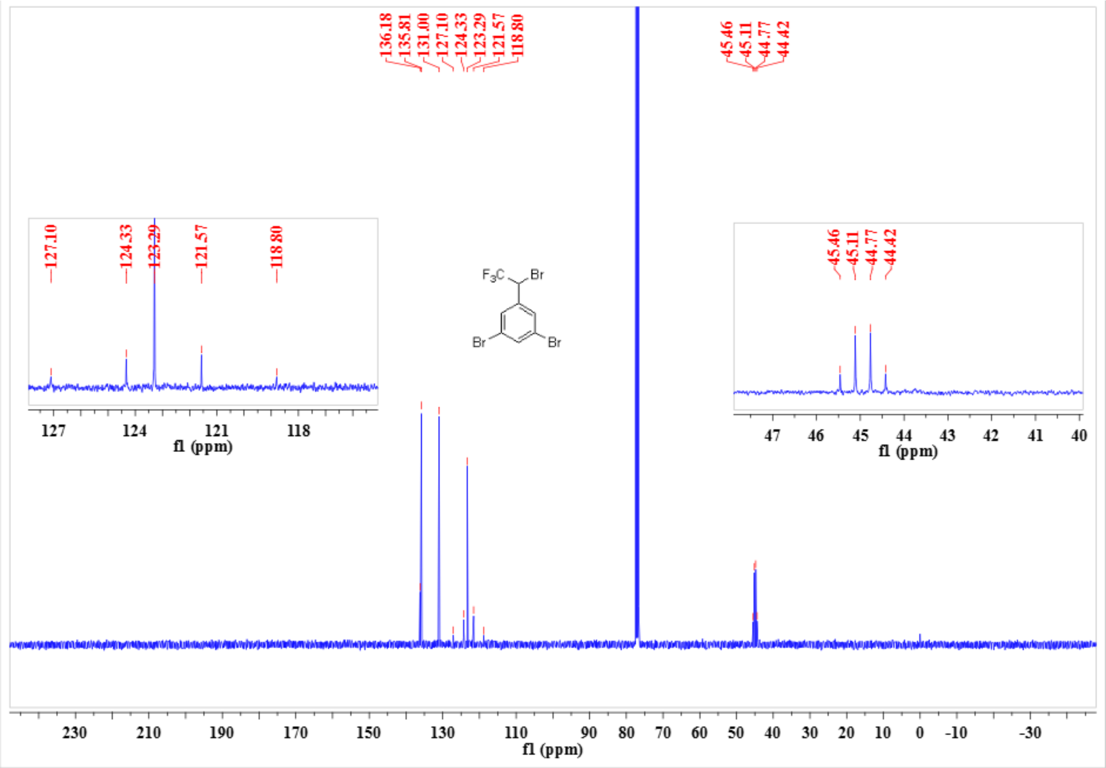

**Supplementary Figure 171.** $^{13}$C NMR (101 MHz, $CDCl_3$) spectrum of 1n

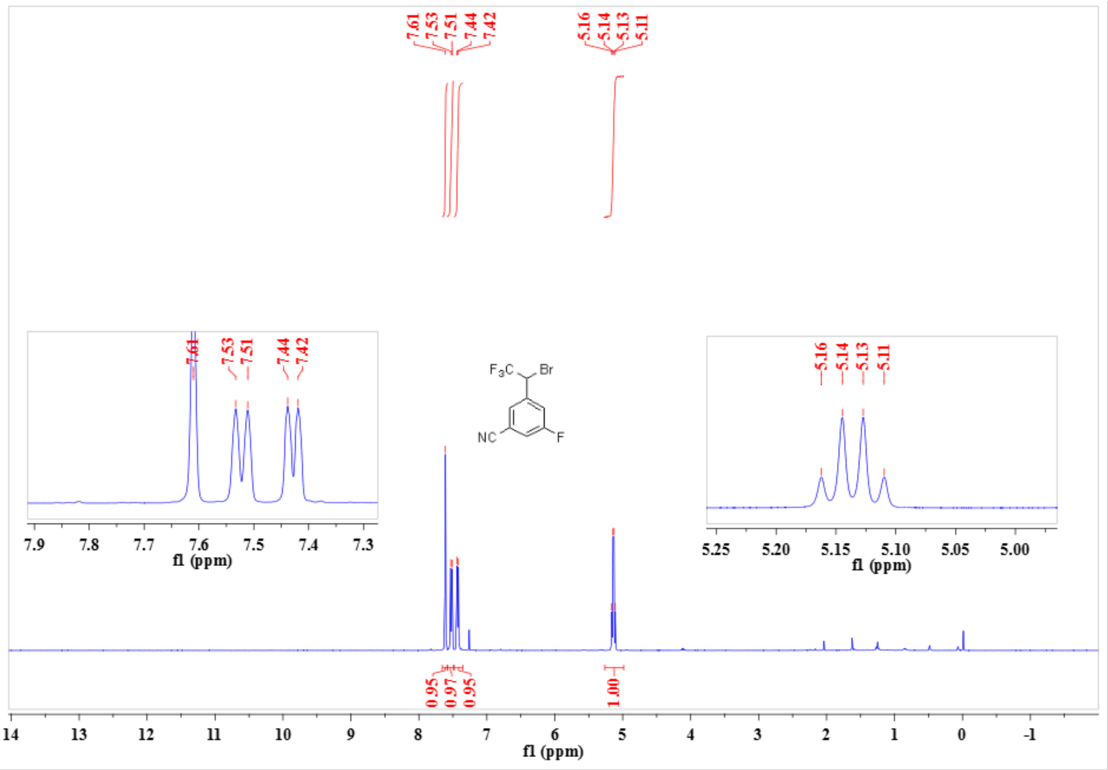

**Supplementary Figure 172.** $^{1}$H NMR (400 MHz, $CDCl_3$) spectrum of 1o

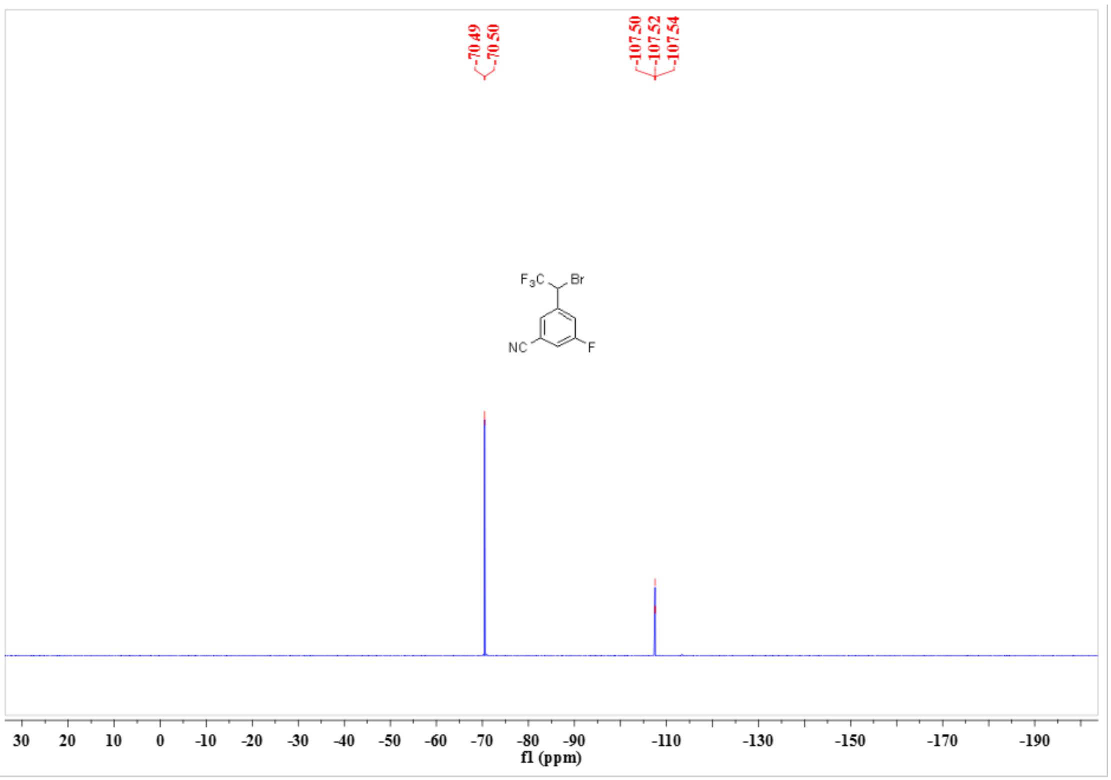


**Supplementary Figure 173.** $^{19}$**F NMR (376 MHz, CDCl$_3$) spectrum of 1o**

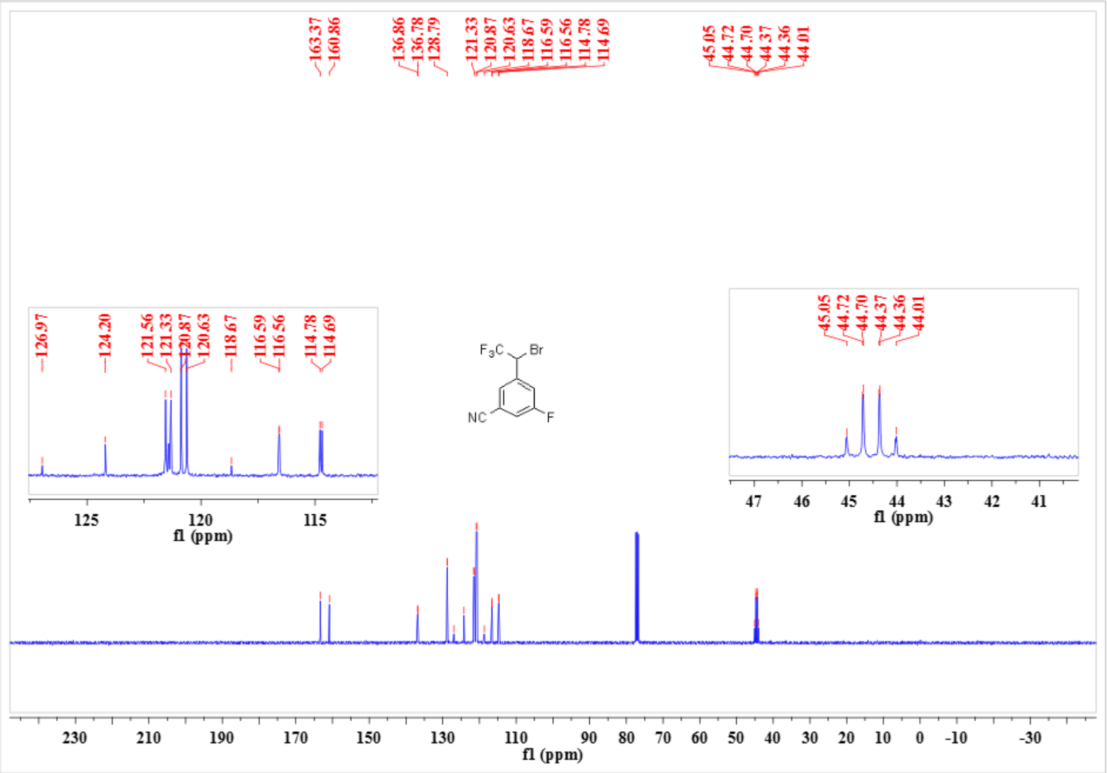


**Supplementary Figure 174.** $^{13}$**C NMR (101 MHz, CDCl$_3$) spectrum of 1o**

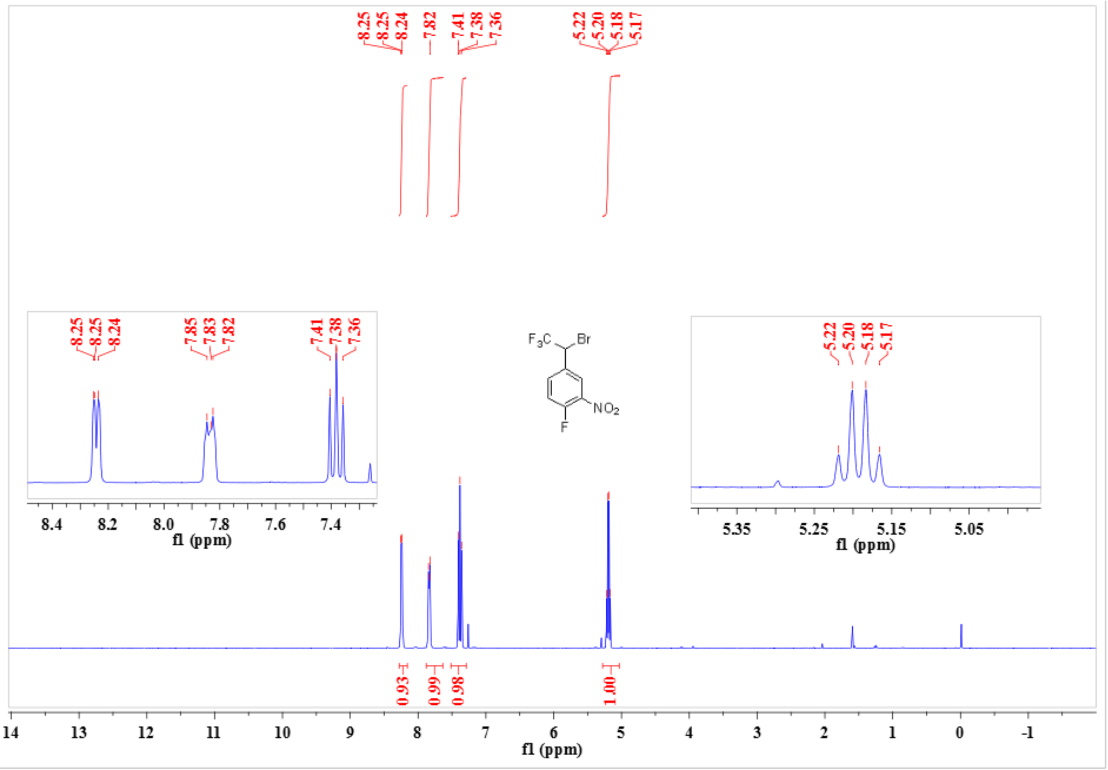

**Supplementary Figure 175.** $^{1}$**H NMR (400 MHz, CDCl$_3$) spectrum of 1p**

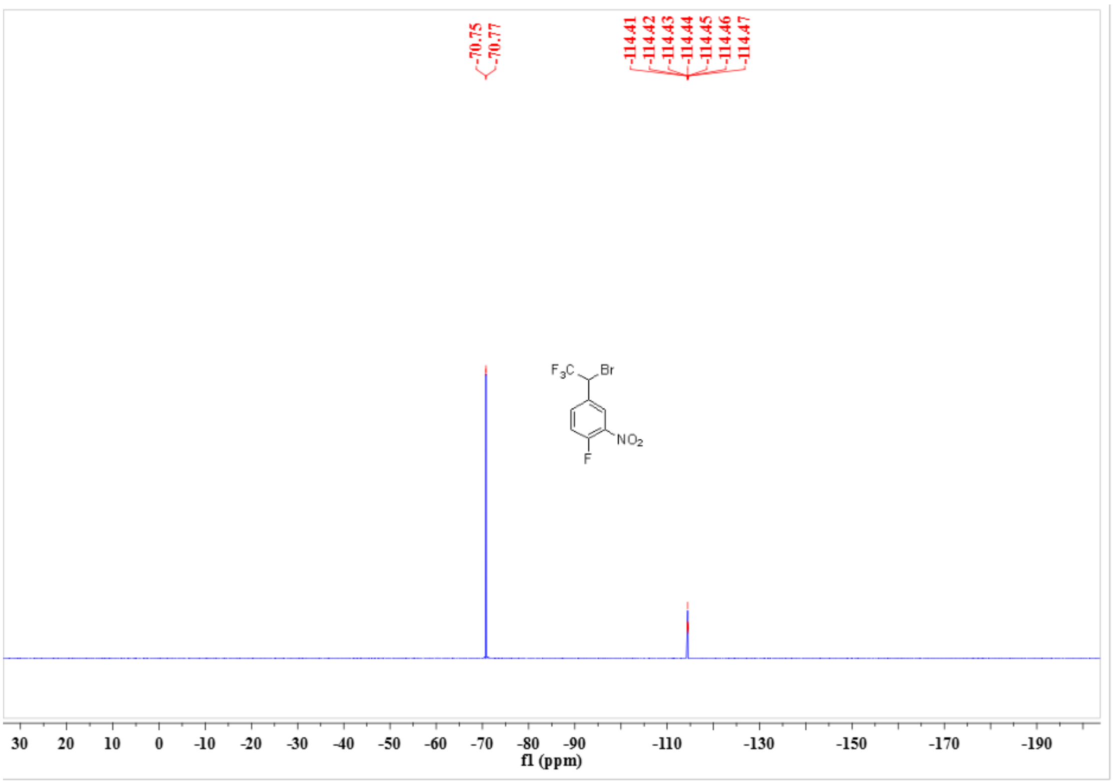

**Supplementary Figure 176.** $^{19}$**F NMR (376 MHz, CDCl$_3$) spectrum of 1p**

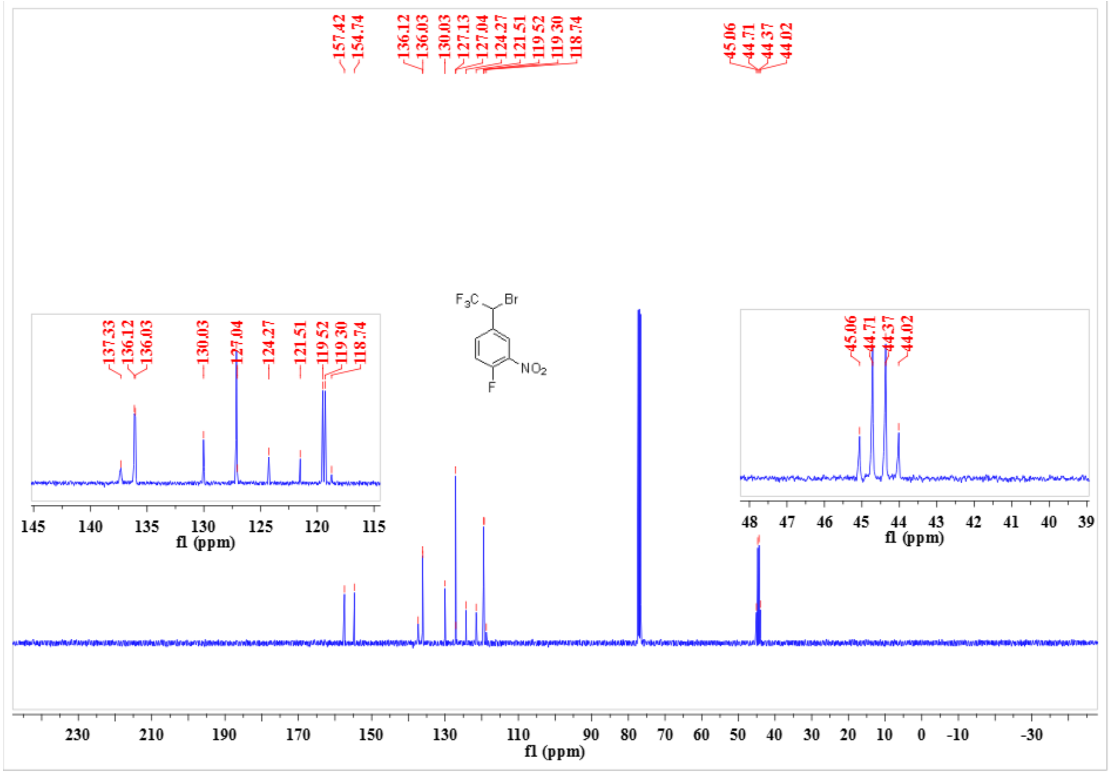


**Supplementary Figure 177.** **$^{13}$C NMR (101 MHz, $CDCl_3$) spectrum of 1p**

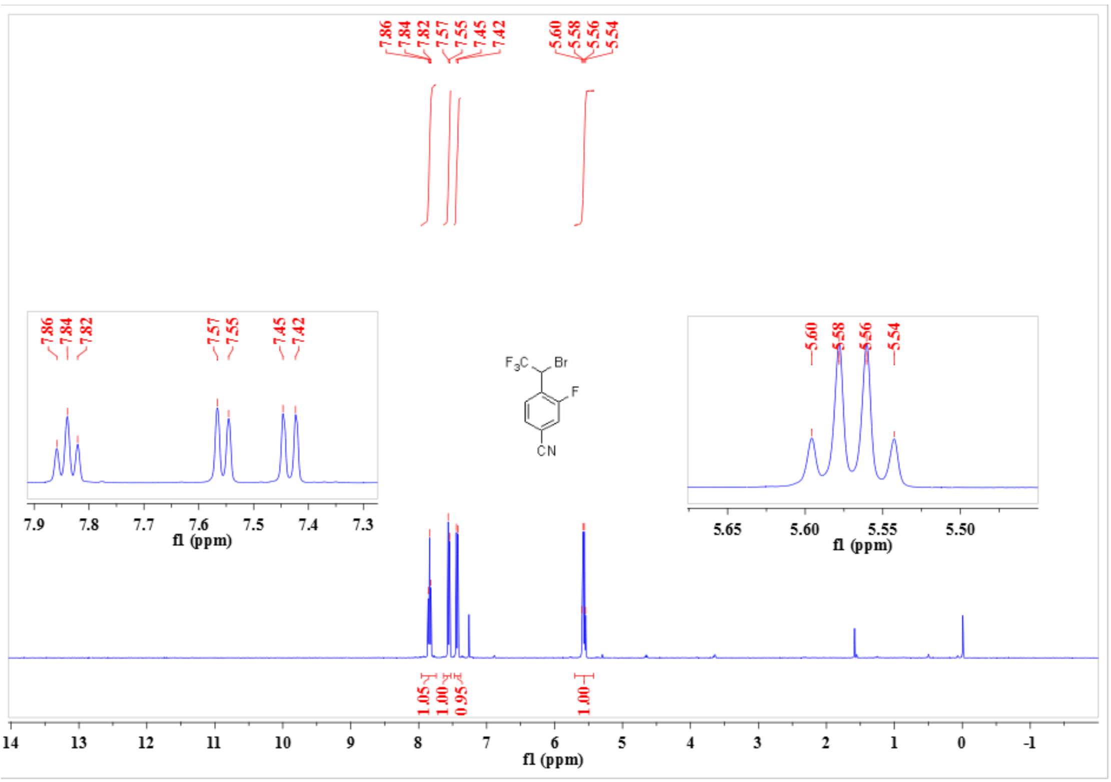


**Supplementary Figure 178.** **$^1$H NMR (400 MHz, $CDCl_3$) spectrum of 1q**

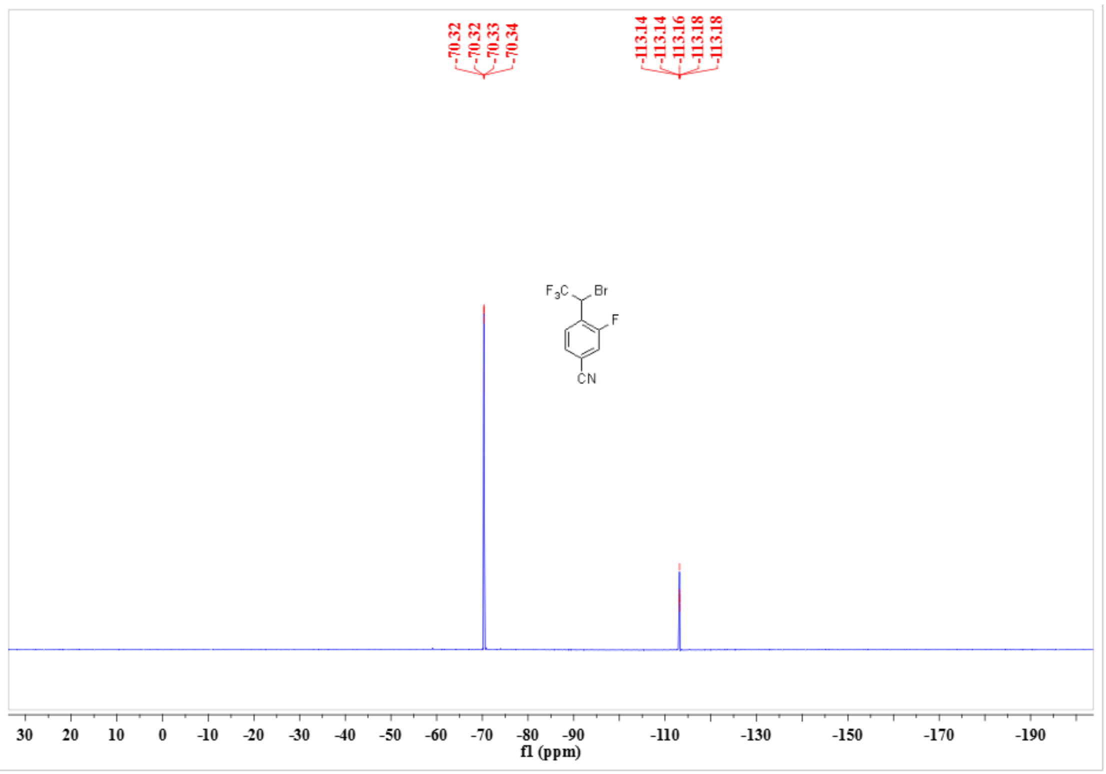

**Supplementary Figure 179.** $^{19}$**F NMR (376 MHz, CDCl$_3$) spectrum of 1q**

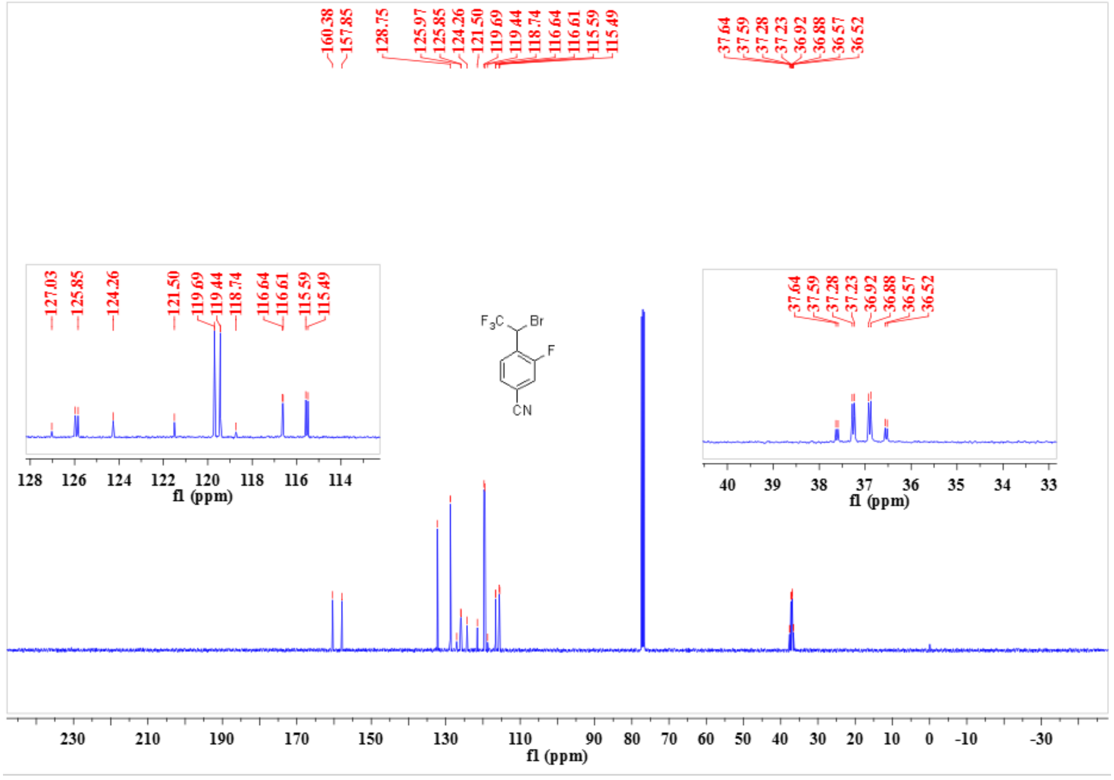

**Supplementary Figure 180.** $^{13}$**C NMR (101 MHz, CDCl$_3$) spectrum of 1q**

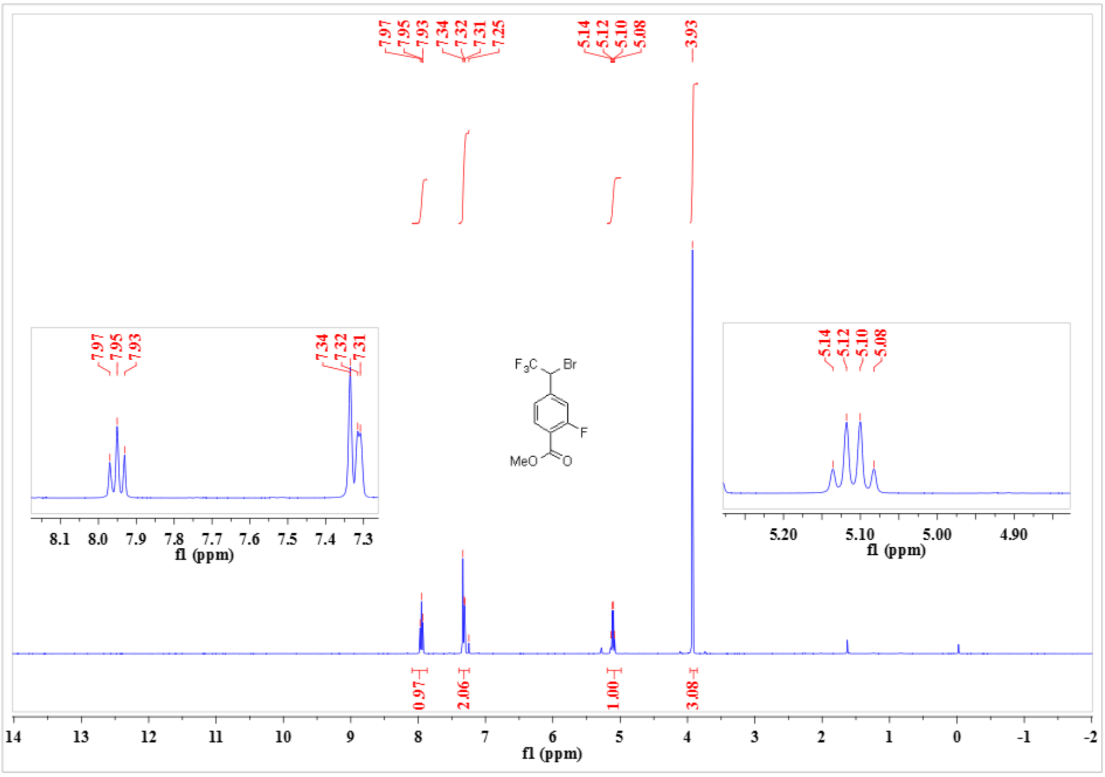


**Supplementary Figure 181.** $^{1}$**H NMR (400 MHz, CDCl$_3$) spectrum of 1r**

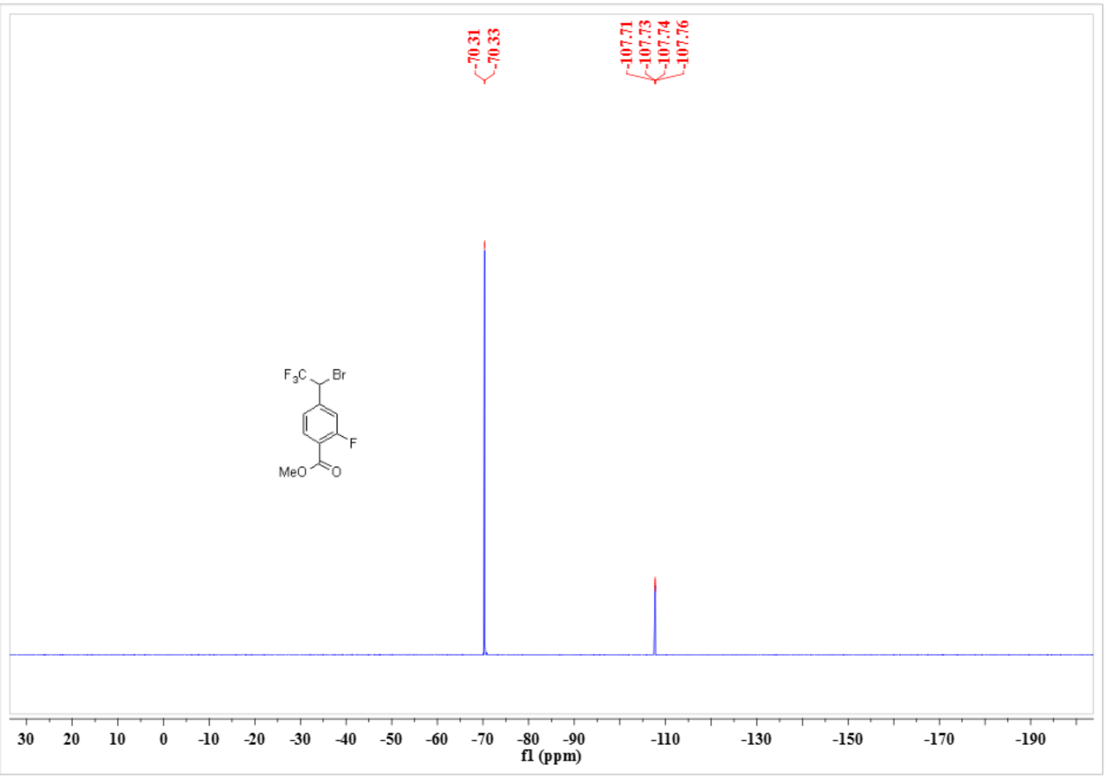


**Supplementary Figure 182.** $^{19}$**F NMR (376 MHz, CDCl$_3$) spectrum of 1r**

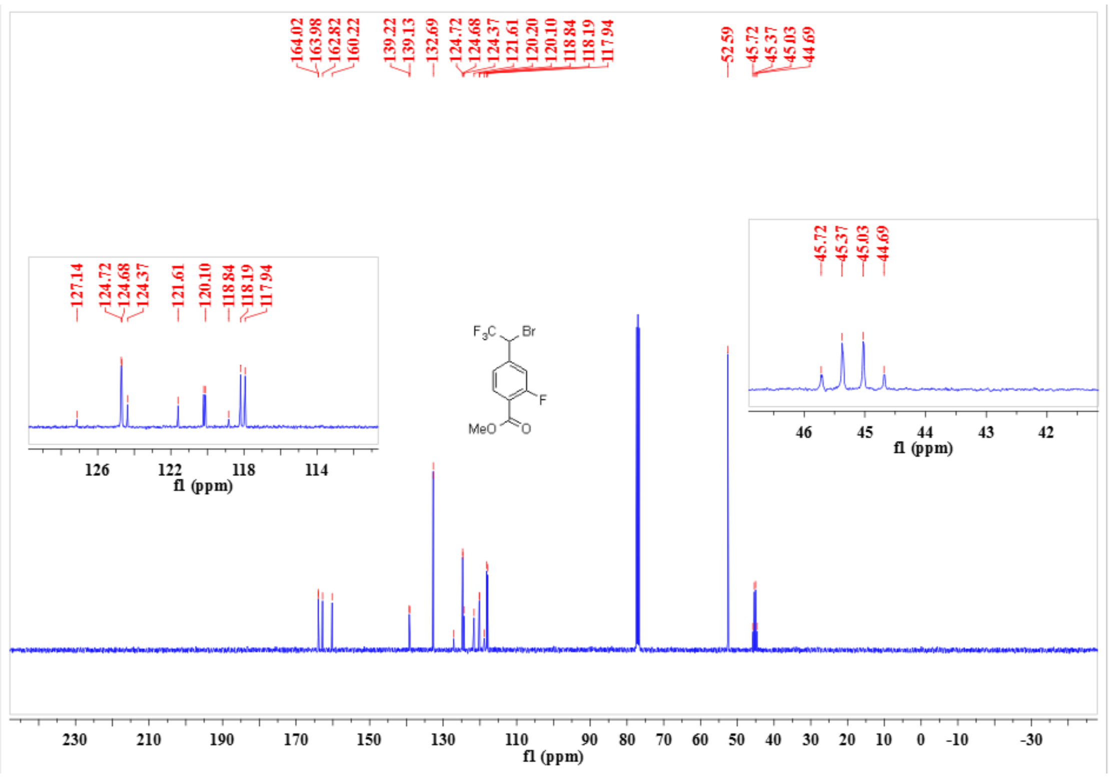

Supplementary Figure 183. $^{13}$C NMR (101 MHz, CDCl$_3$) spectrum of 1r

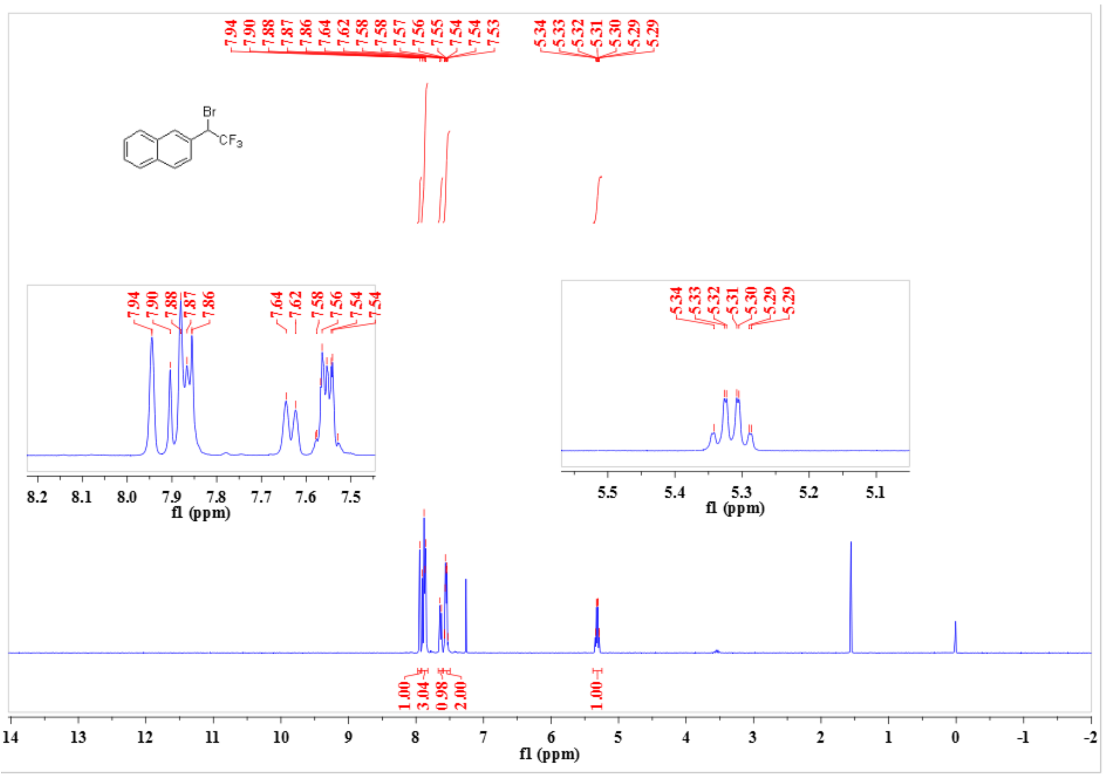

Supplementary Figure 184. $^{1}$H NMR (400 MHz, CDCl$_3$) spectrum of 1s

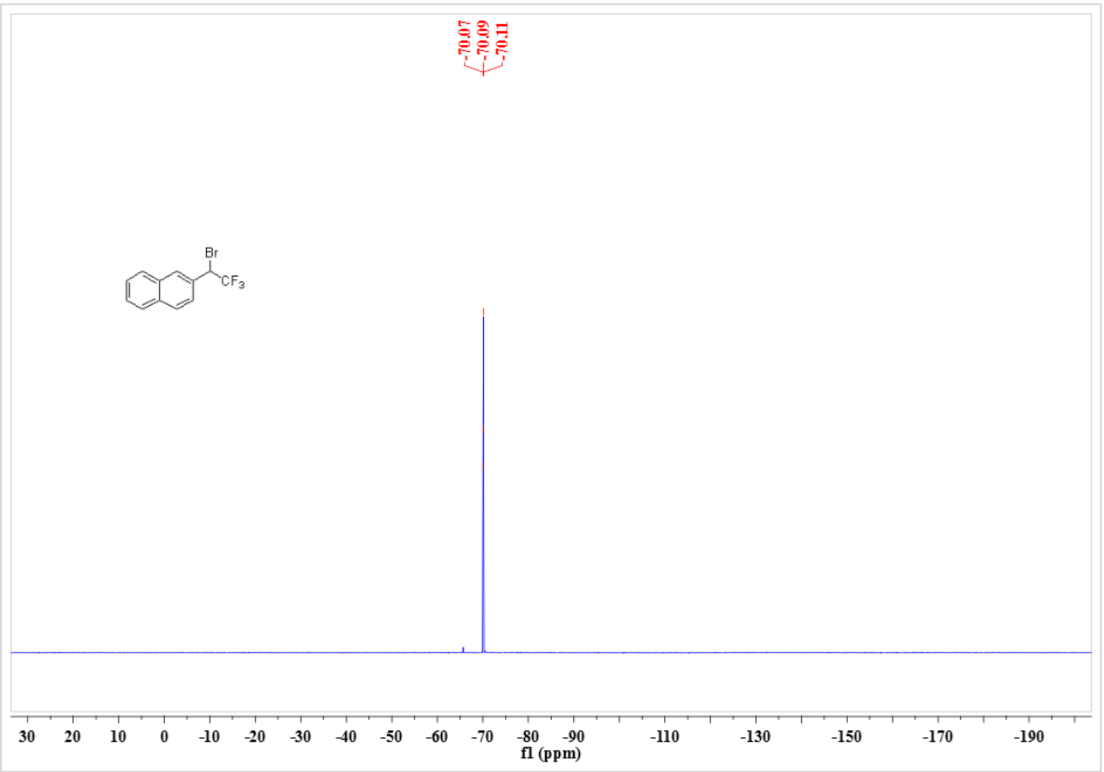


**Supplementary Figure 185.** $^{19}$F NMR (376 MHz, $CDCl_3$) spectrum of 1s

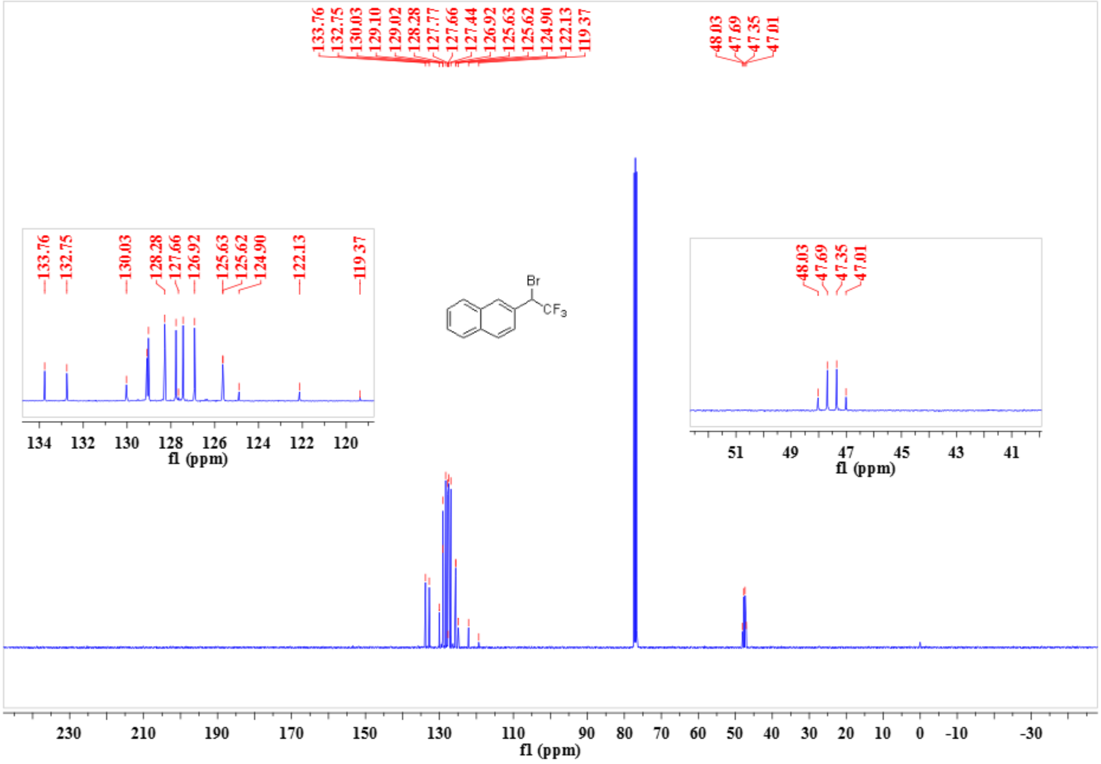


**Supplementary Figure 186.** $^{13}$C NMR (101 MHz, $CDCl_3$) spectrum of 1s

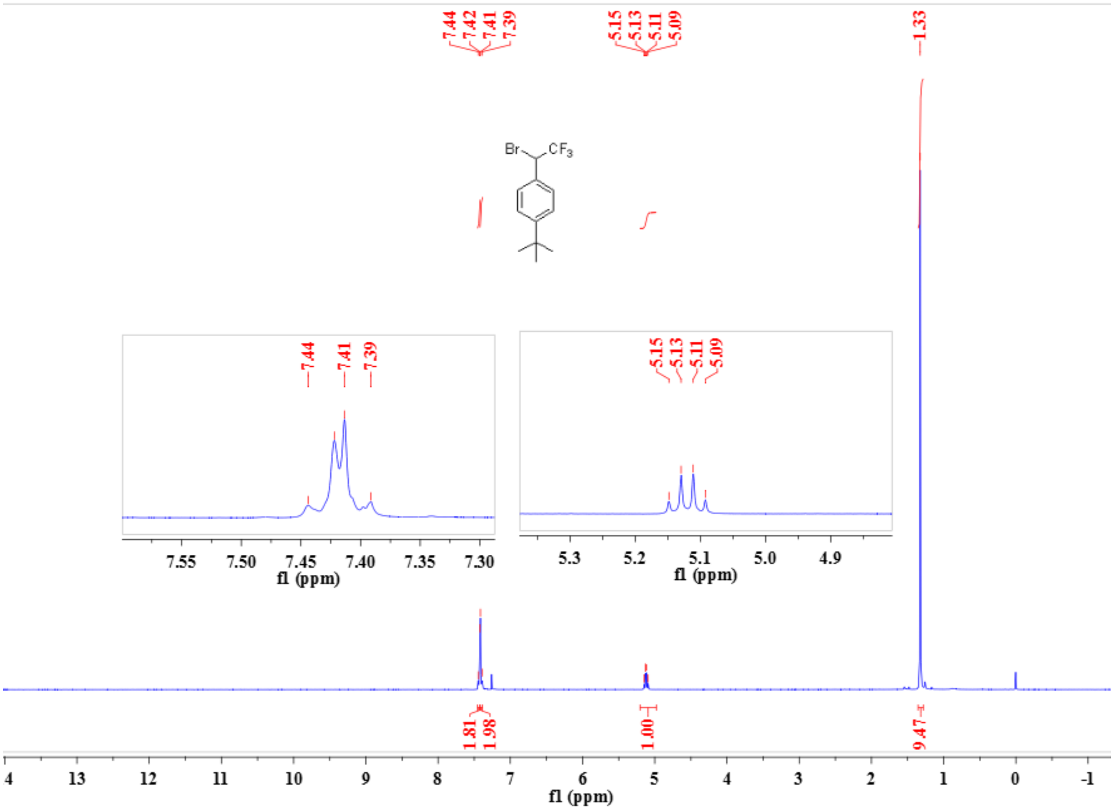

**Supplementary Figure 187.** $^{1}$H NMR (400 MHz, $CDCl_3$) spectrum of 1t

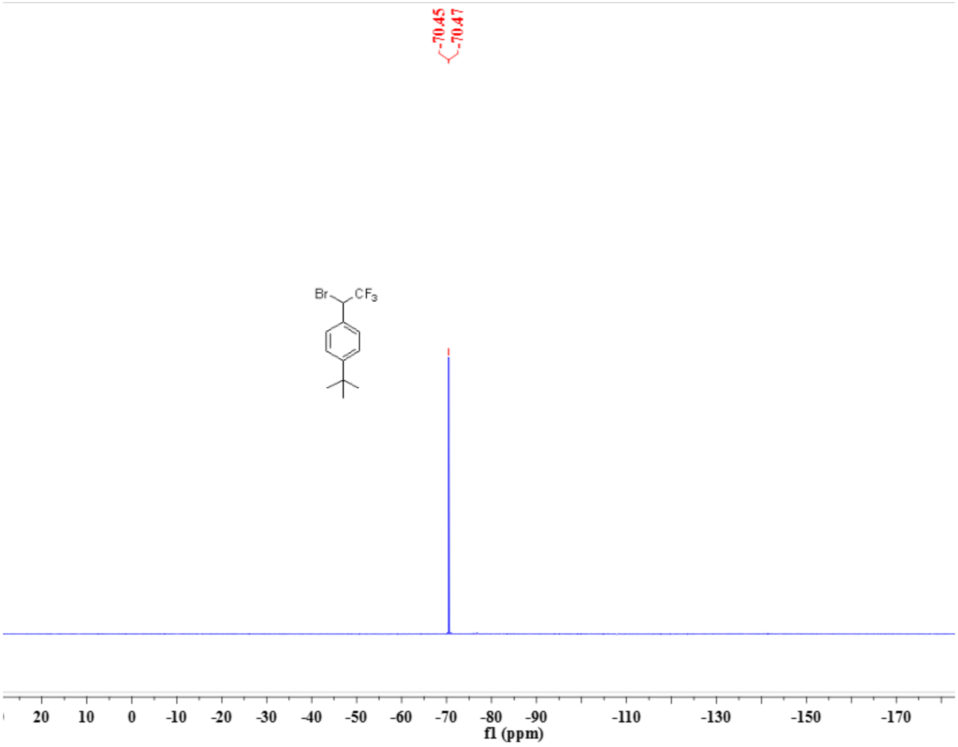

**Supplementary Figure 188.** $^{19}$F NMR (376 MHz, $CDCl_3$) spectrum of 1t

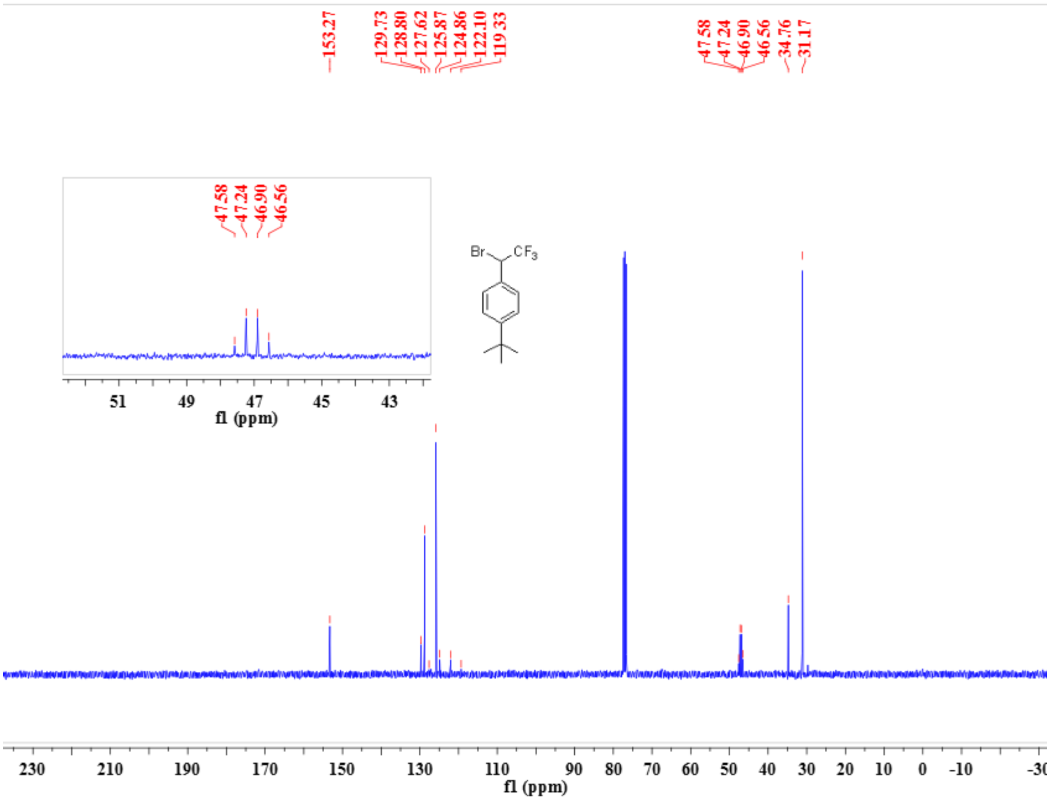


**Supplementary Figure 189.** $^{13}C$ **NMR (101 MHz, $CDCl_3$) spectrum of 1t**

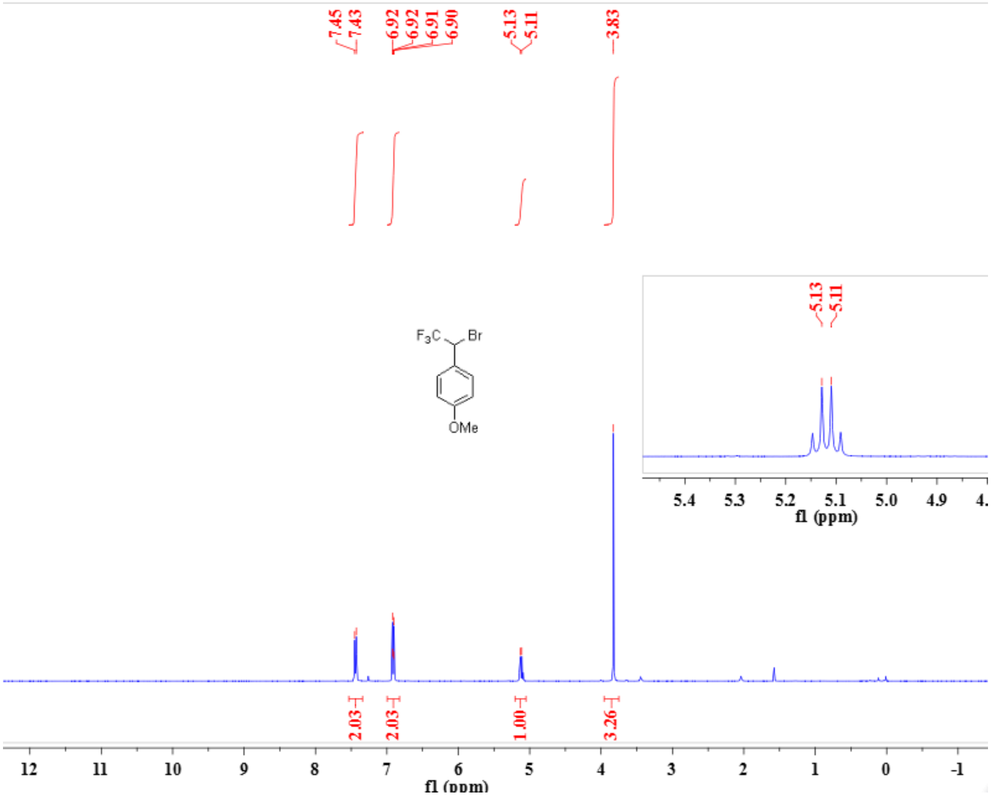


**Supplementary Figure 190.** $^{1}H$ **NMR (400 MHz, $CDCl_3$) spectrum of 1u**

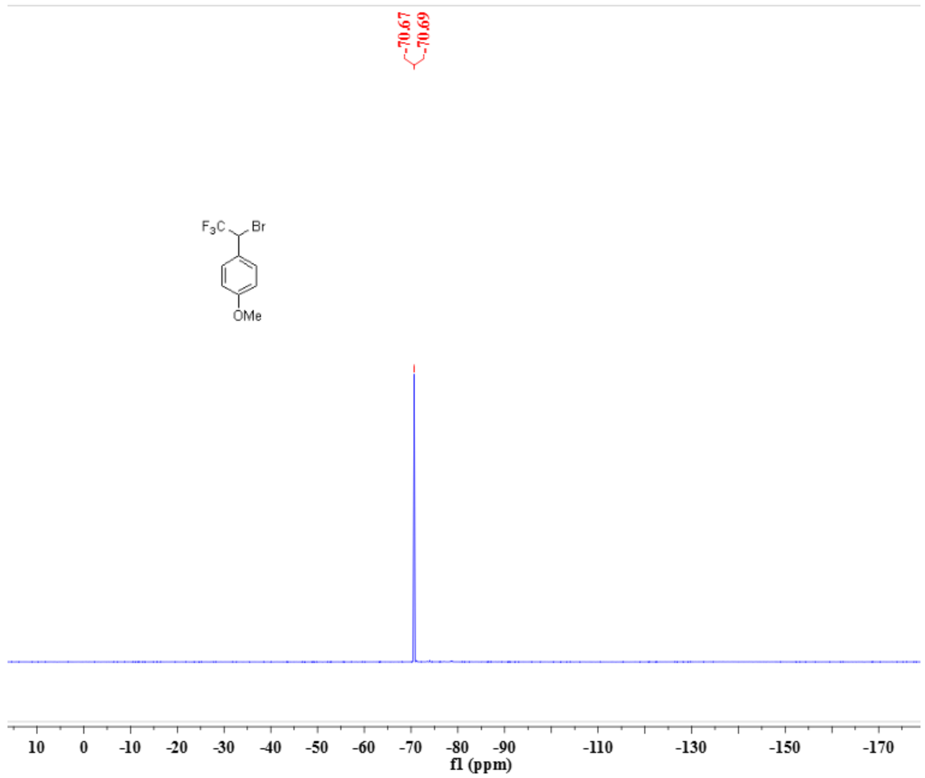


**Supplementary Figure 191.** $^{19}$**F NMR (376 MHz, CDCl$_3$) spectrum of 1u**

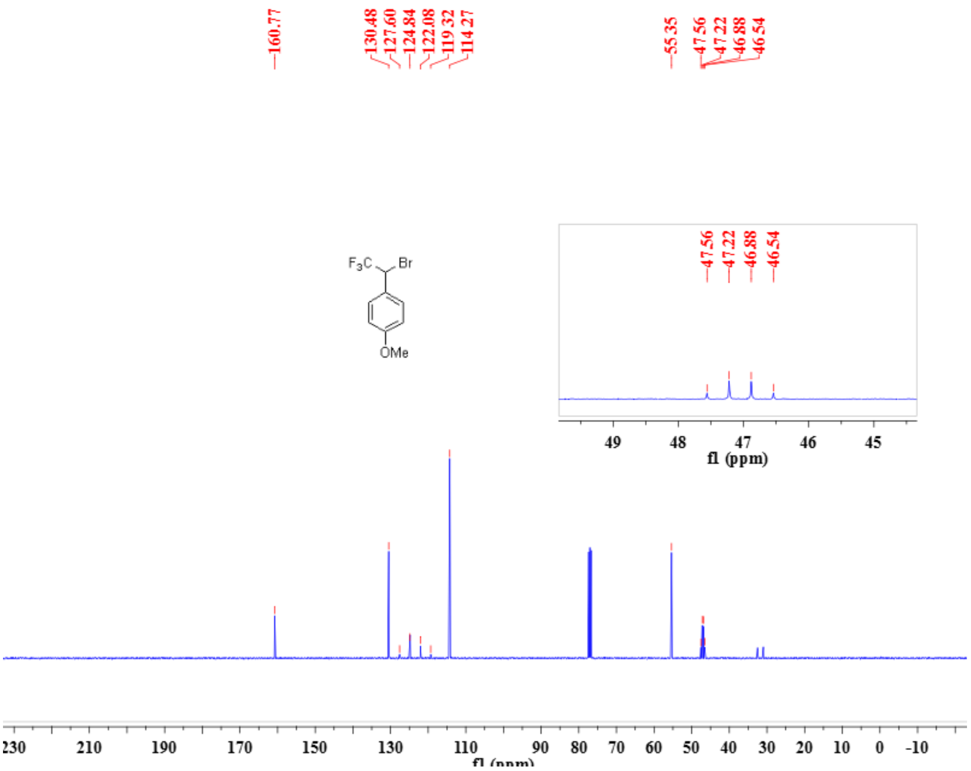


**Supplementary Figure 192.** $^{13}$**C NMR (101 MHz, CDCl$_3$) spectrum of 1u**

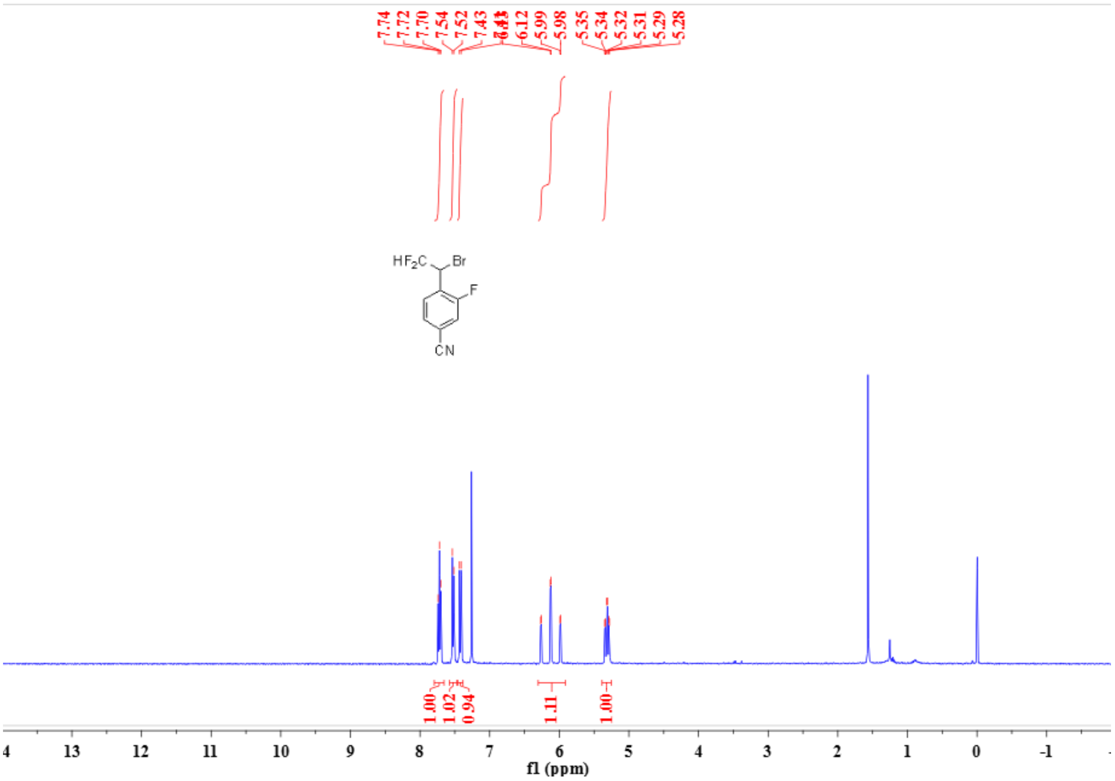

Supplementary Figure 193. $^{1}$H NMR (400 MHz, $CDCl_3$) spectrum of 1v

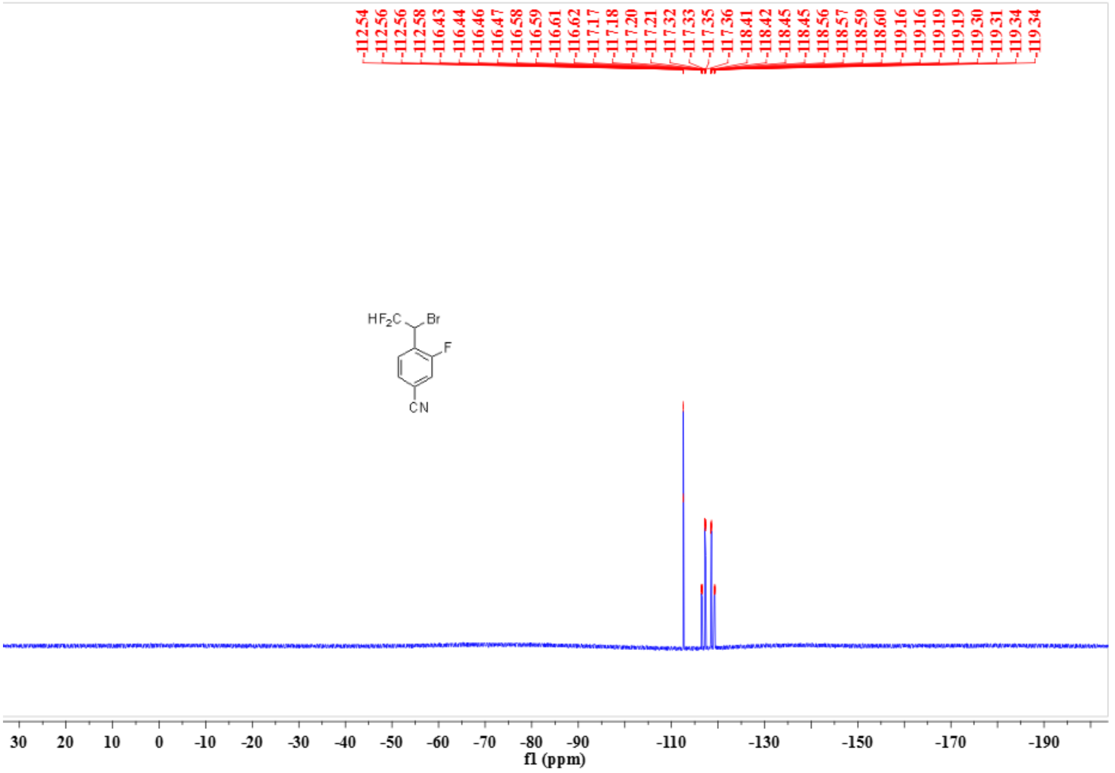

Supplementary Figure 194. $^{19}$F NMR (376 MHz, $CDCl_3$) spectrum of 1v

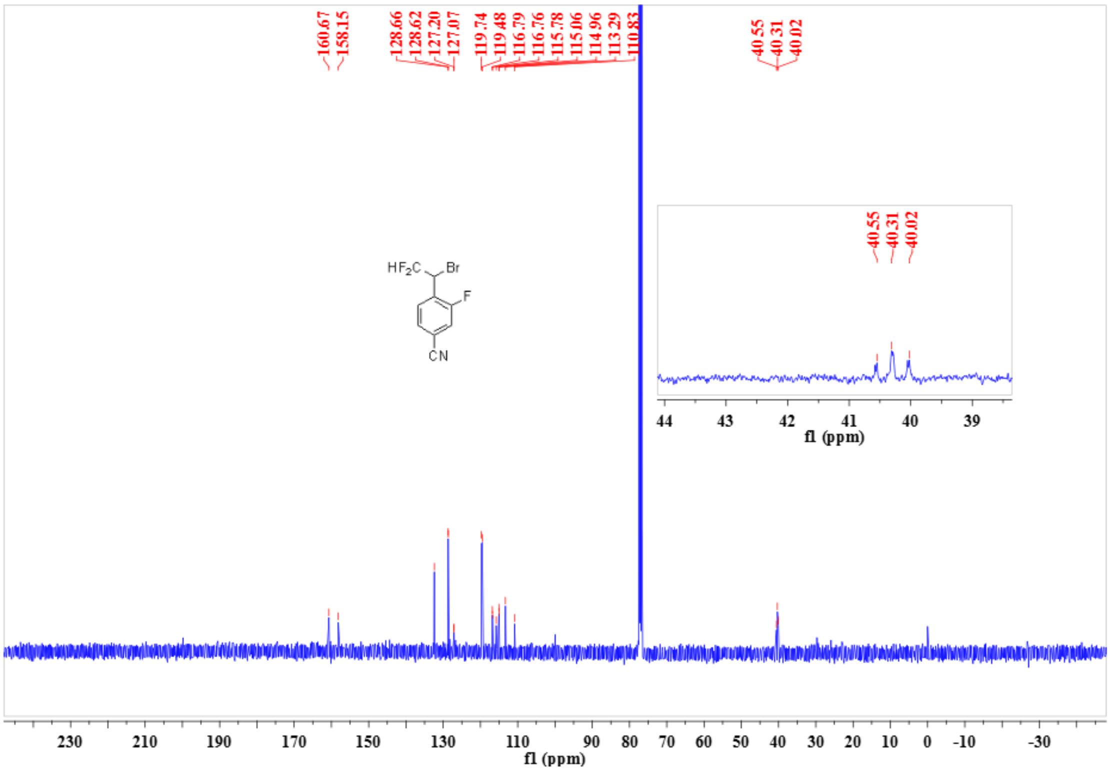

**Supplementary Figure 195.** $^{13}C$ **NMR (101 MHz,** $CDCl_3$**) spectrum of 1v**

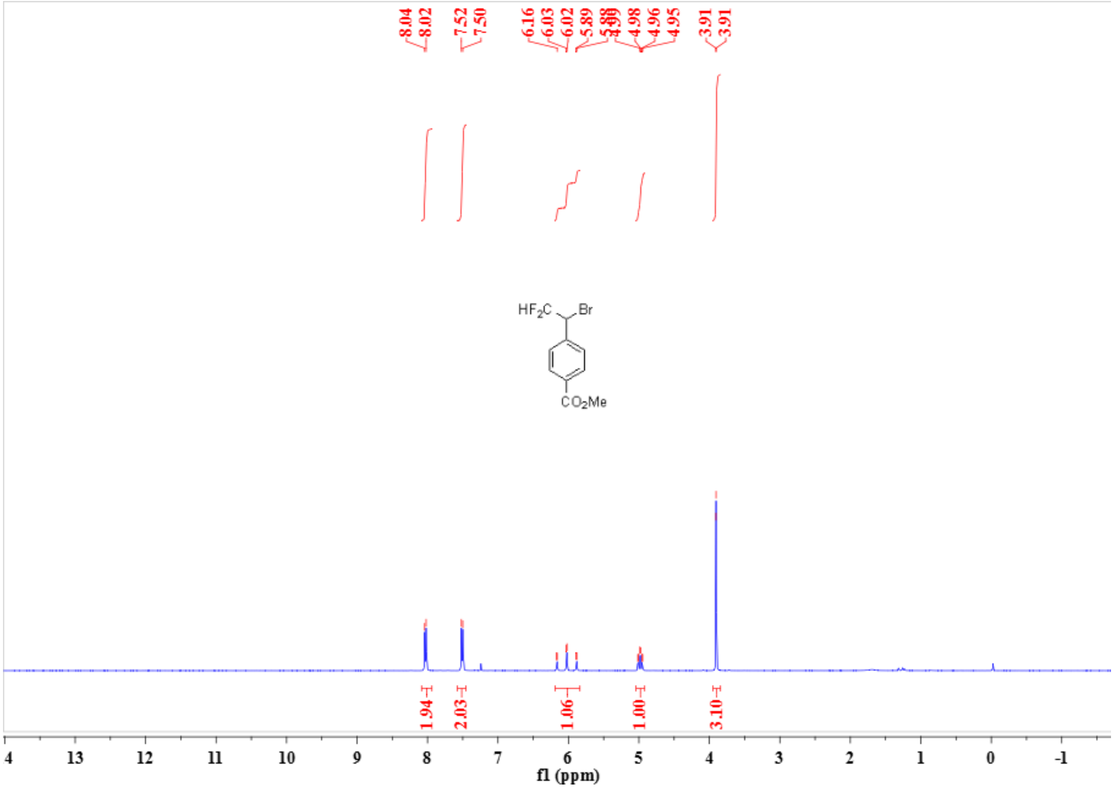

**Supplementary Figure 196.** $^{1}H$ **NMR (400 MHz,** $CDCl_3$**) spectrum of 1w**

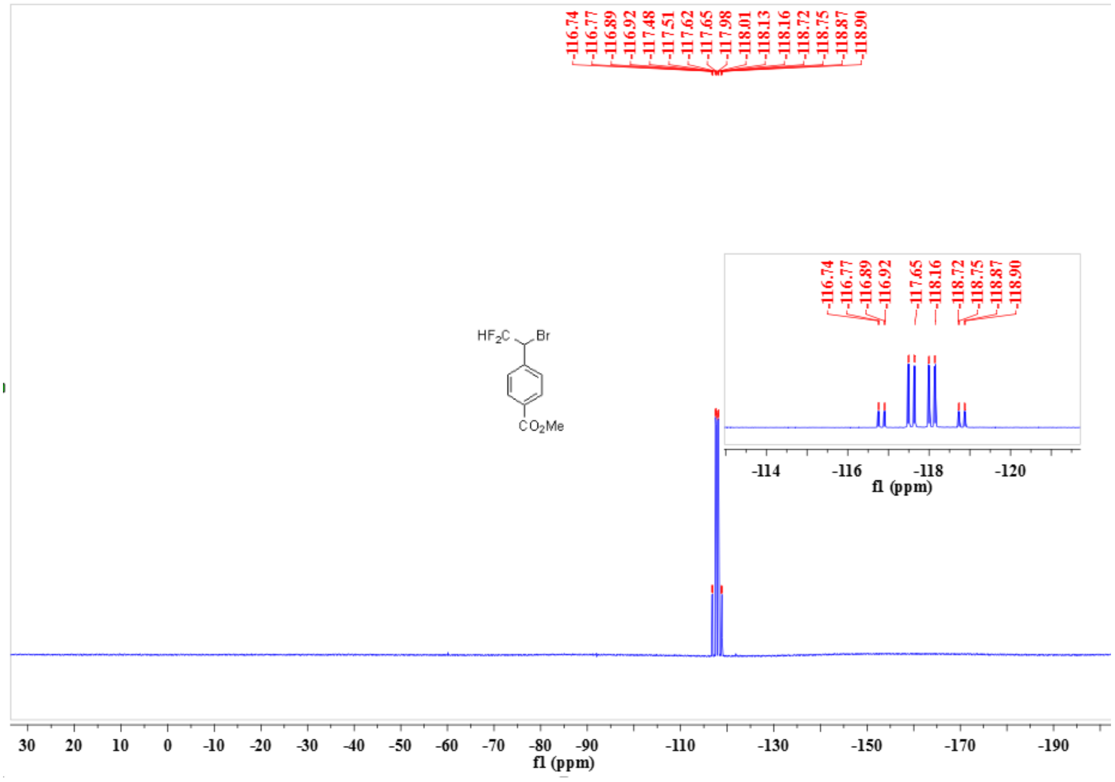


**Supplementary Figure 197.** $^{19}$**F NMR (376 MHz, CDCl$_3$) spectrum of 1w**

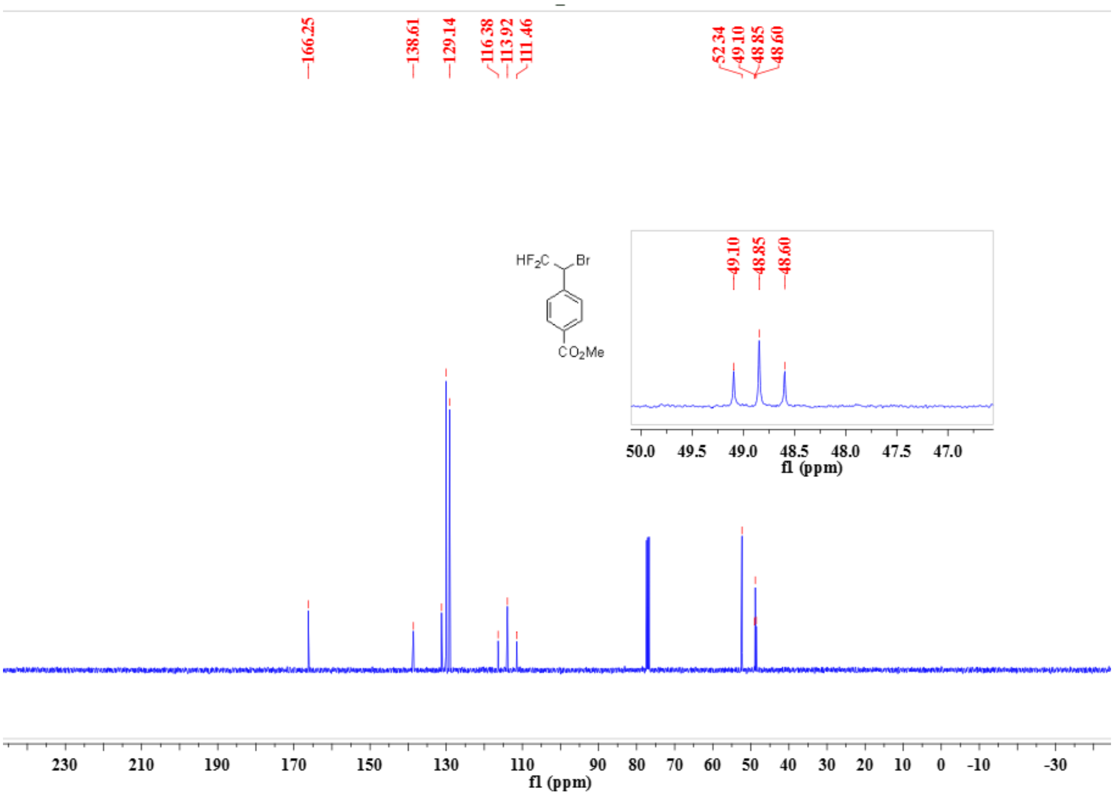


**Supplementary Figure 198.** $^{13}$**C NMR (101 MHz, CDCl$_3$) spectrum of 1w**

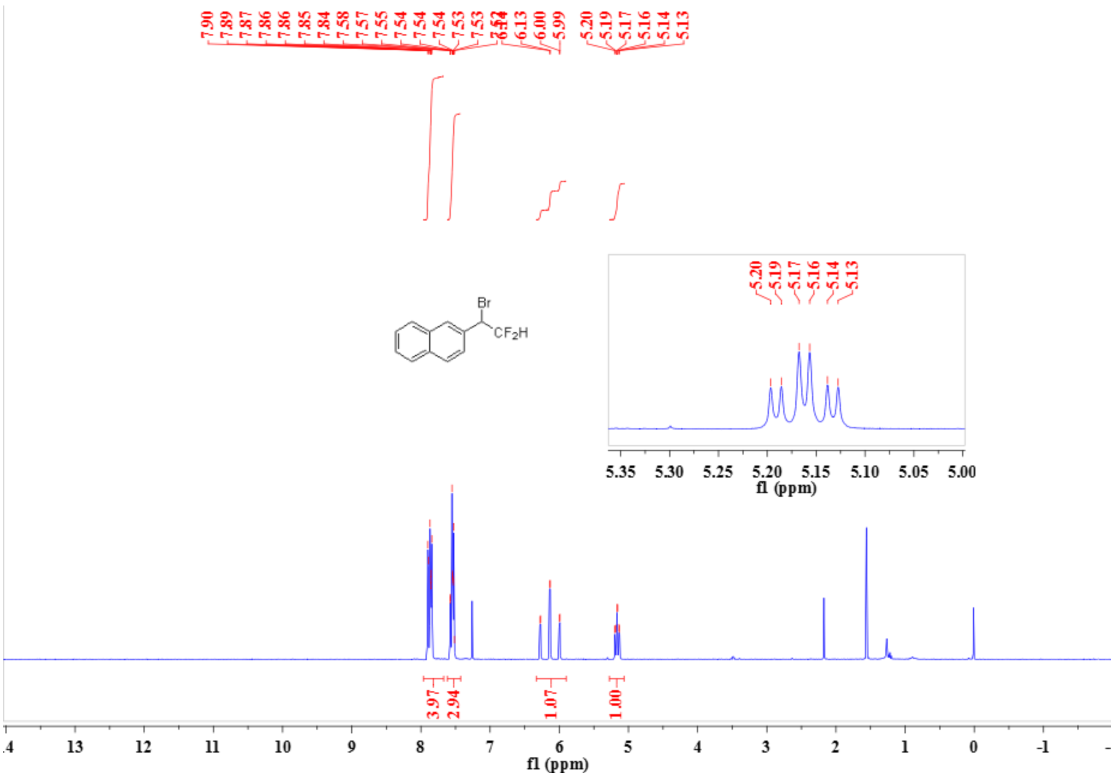

Supplementary Figure 199. $^{1}$H NMR (400 MHz, $CDCl_3$) spectrum of 1x

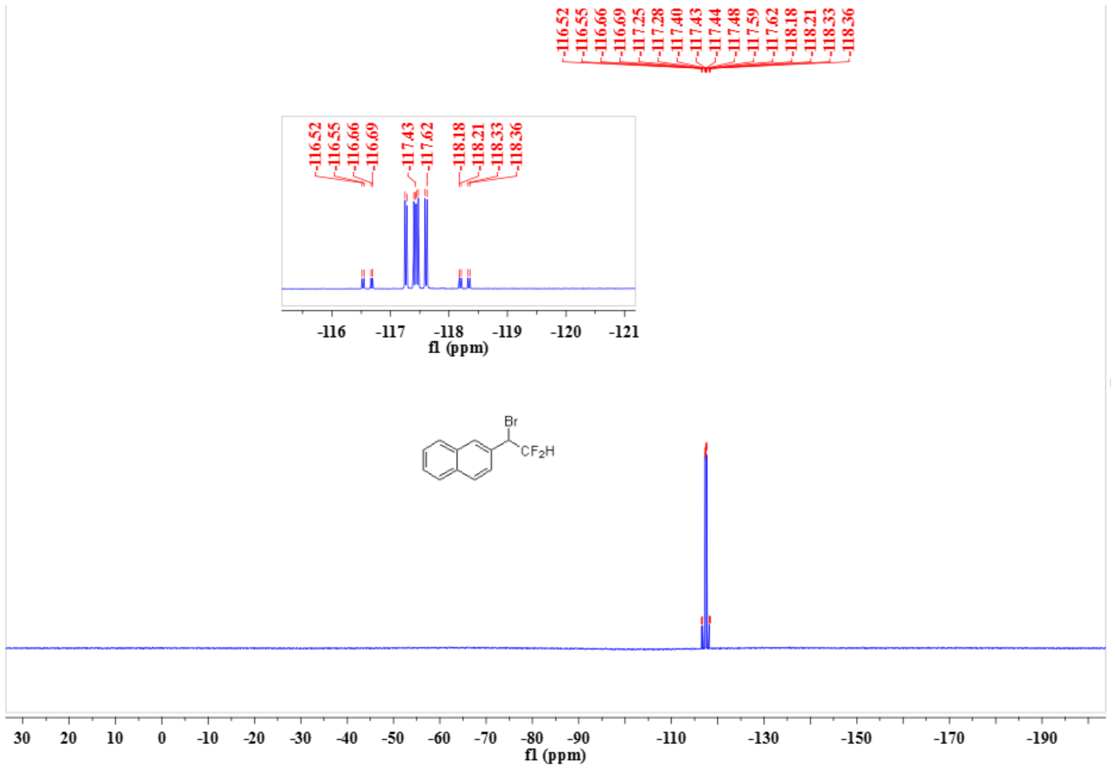

Supplementary Figure 200. $^{19}$F NMR (376 MHz, $CDCl_3$) spectrum of 1x

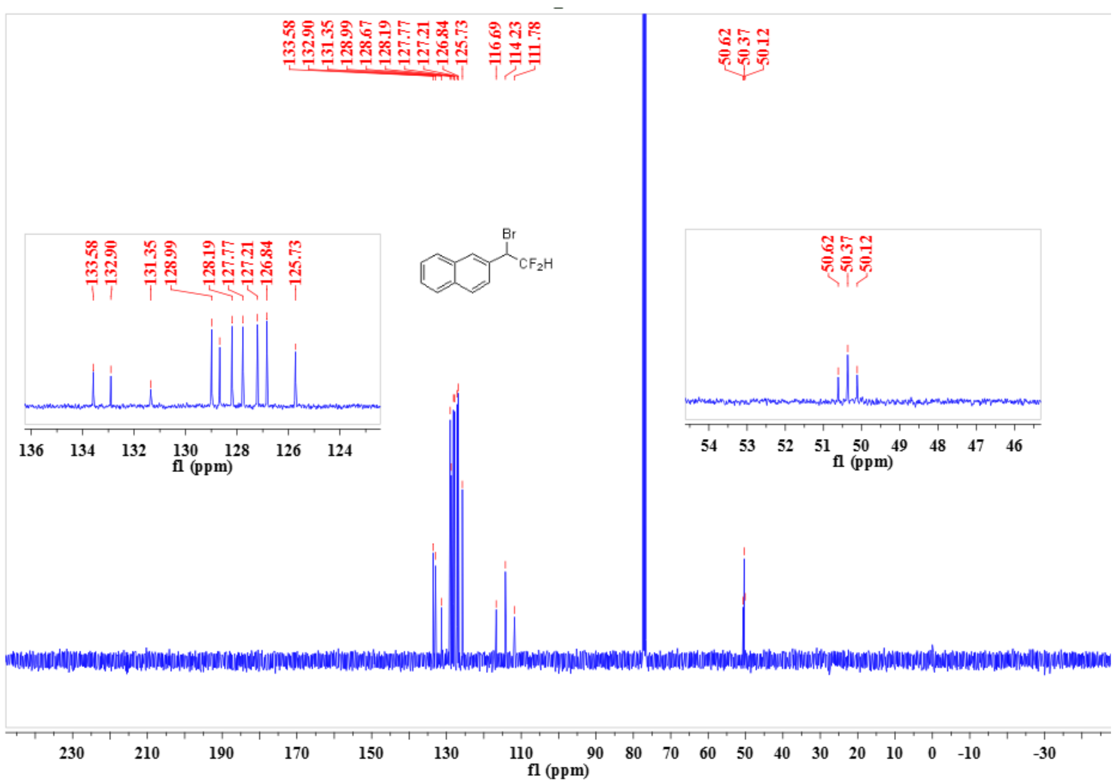

Supplementary Figure 201. $^{13}C$ NMR (101 MHz, $CDCl_3$) spectrum of 1x

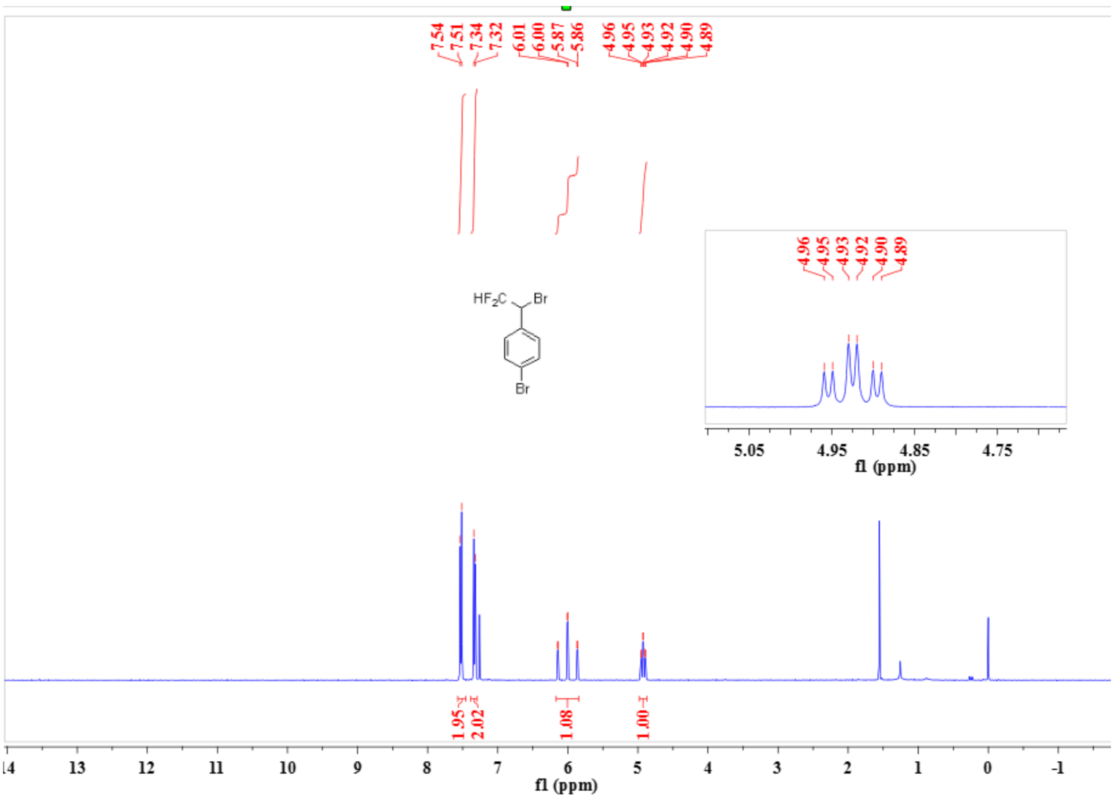

Supplementary Figure 202. $^{1}H$ NMR (400 MHz, $CDCl_3$) spectrum of 1y

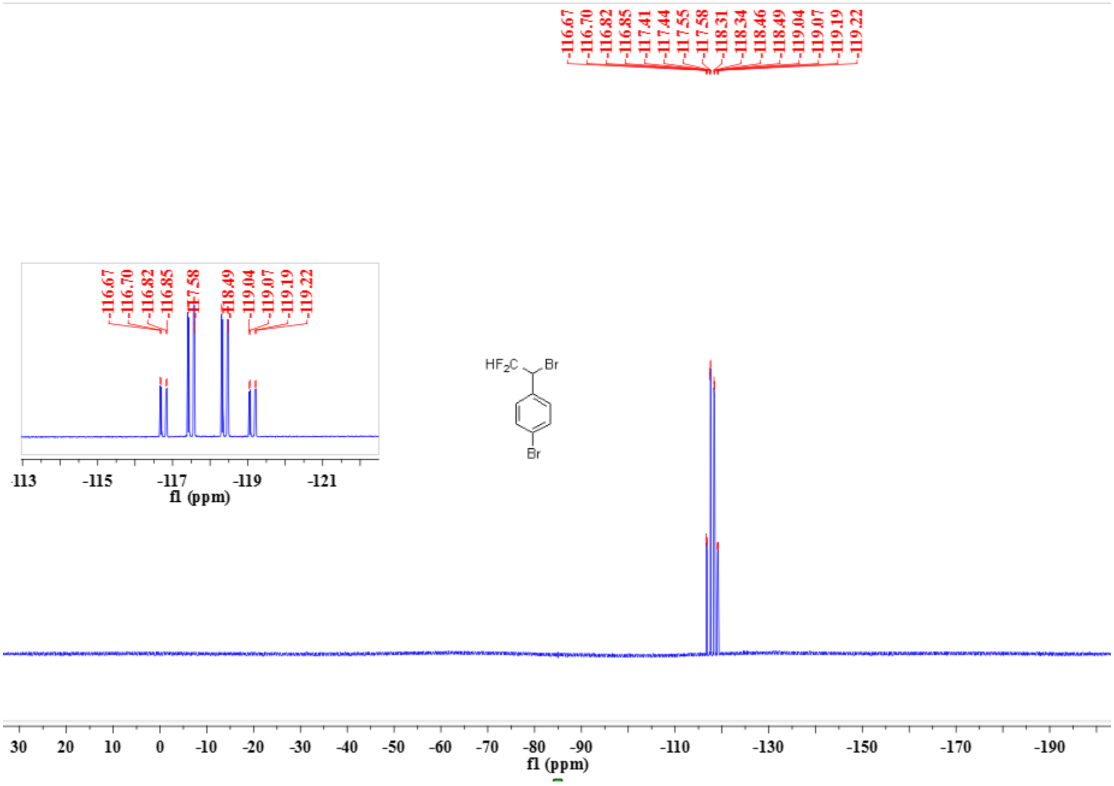


Supplementary Figure 203. $^{19}$F NMR (376 MHz, $CDCl_3$) spectrum of 1y

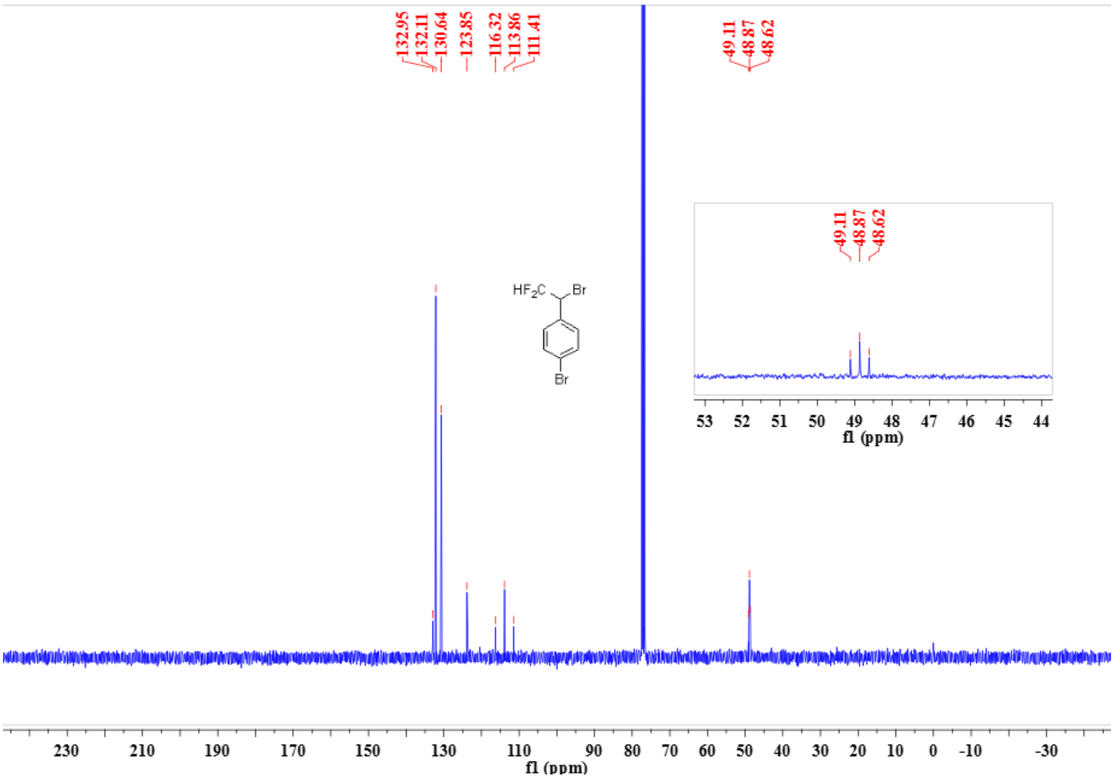


Supplementary Figure 204. $^{13}$C NMR (101 MHz, $CDCl_3$) spectrum of 1y

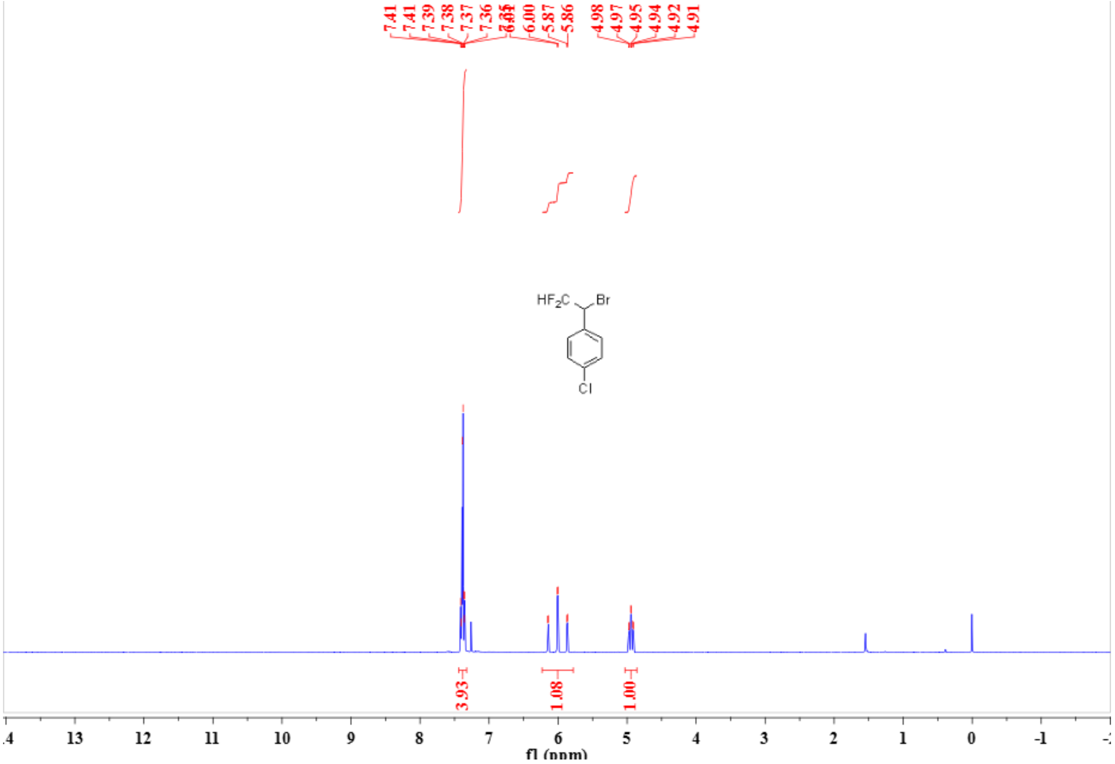


Supplementary Figure 205. $^{1}H$ NMR (400 MHz, $CDCl_3$) spectrum of 1z

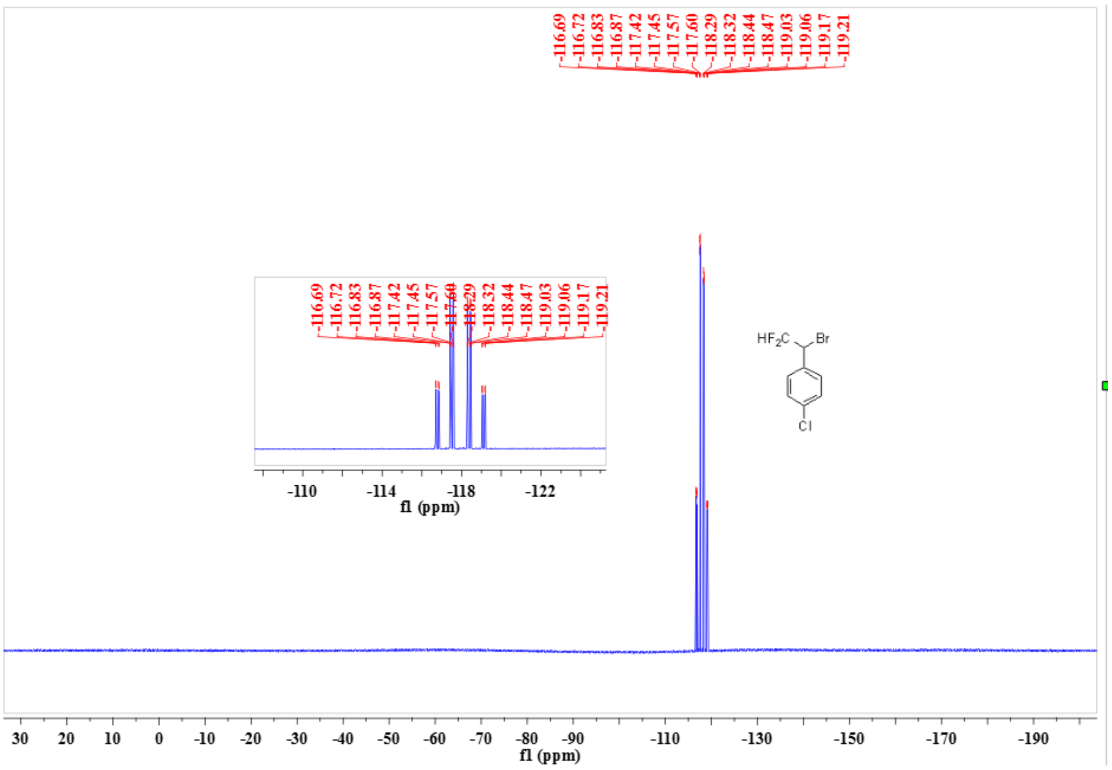


Supplementary Figure 206. $^{19}F$ NMR (376 MHz, $CDCl_3$) spectrum of 1z

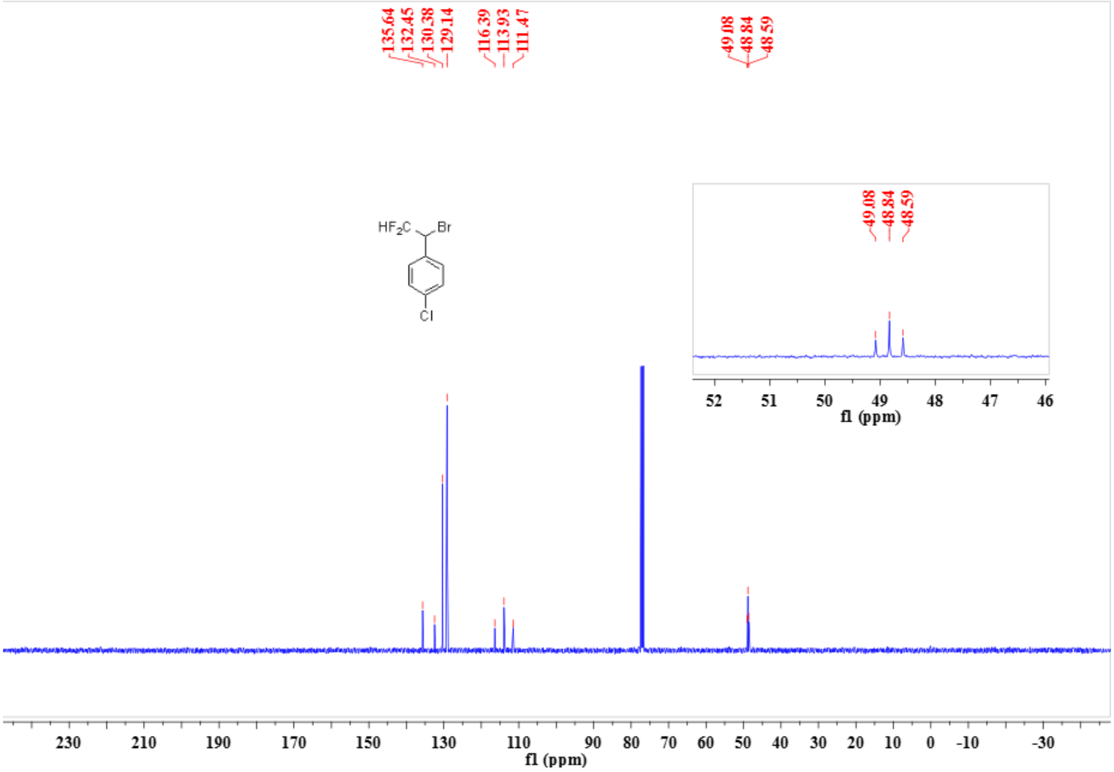

Supplementary Figure 207. $^{13}C$ NMR (101 MHz, $CDCl_3$) spectrum of 1z

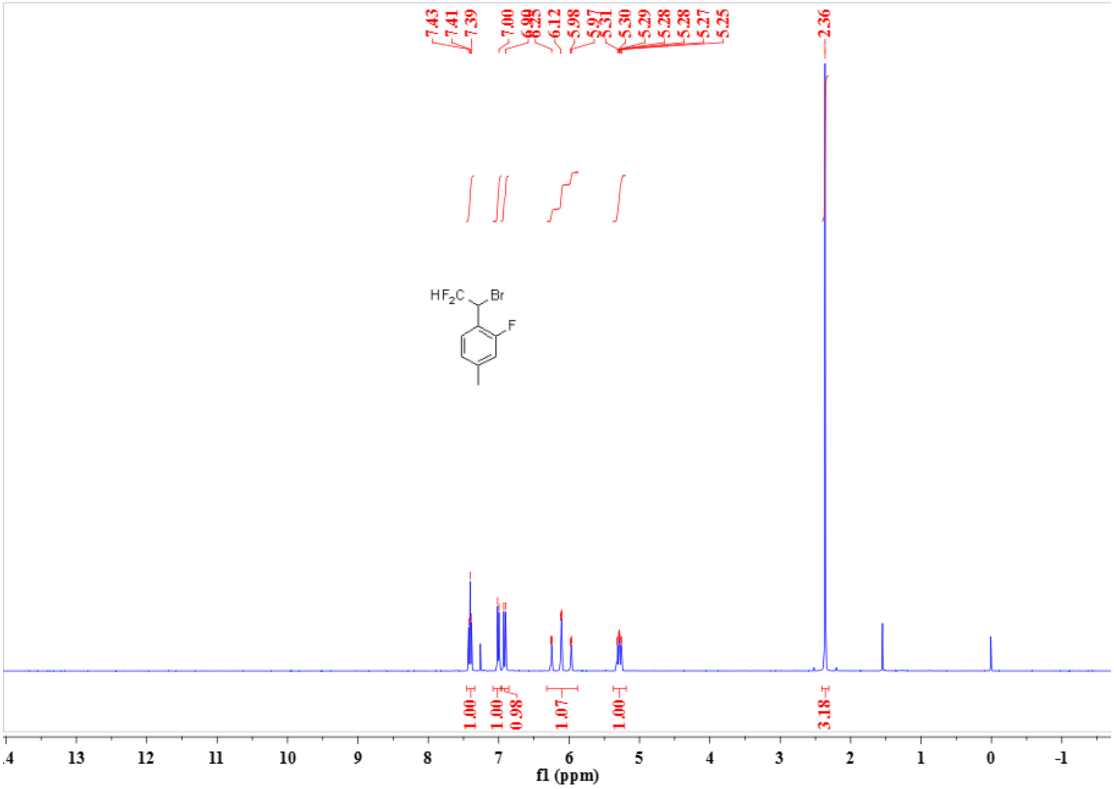

Supplementary Figure 208. $^{1}H$ NMR (400 MHz, $CDCl_3$) spectrum of 1aa

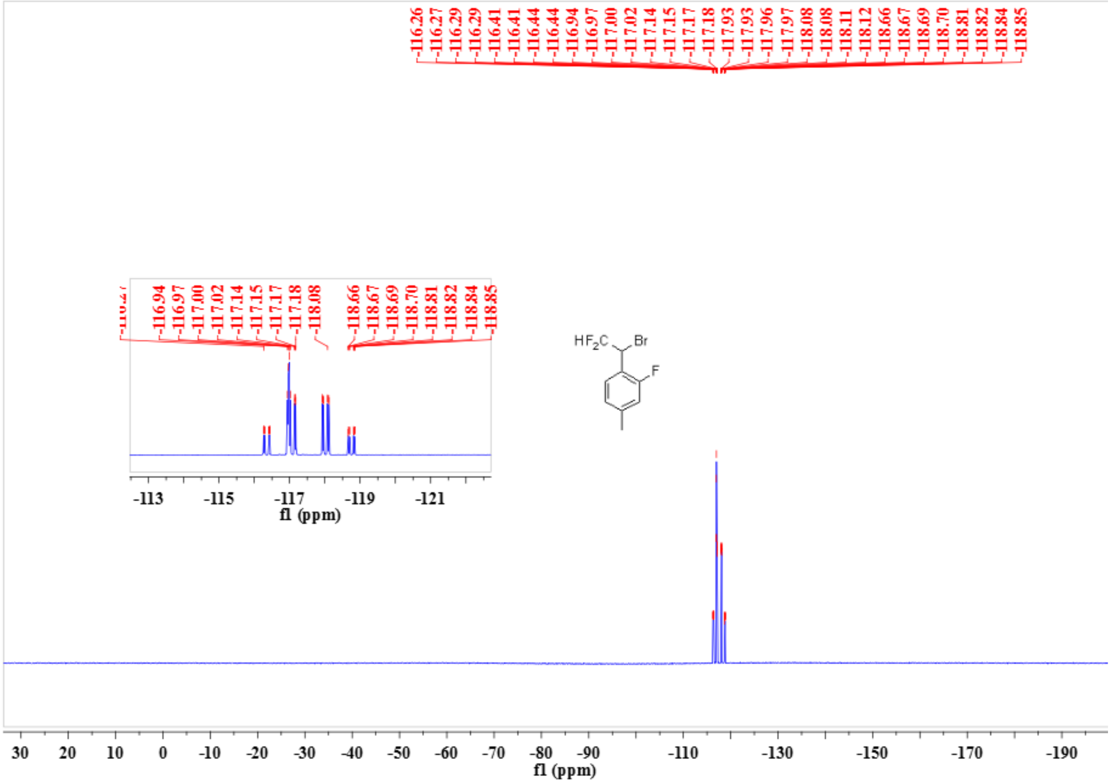

Supplementary Figure 209. $^{19}F$ NMR (376 MHz, $CDCl_3$) spectrum of 1aa

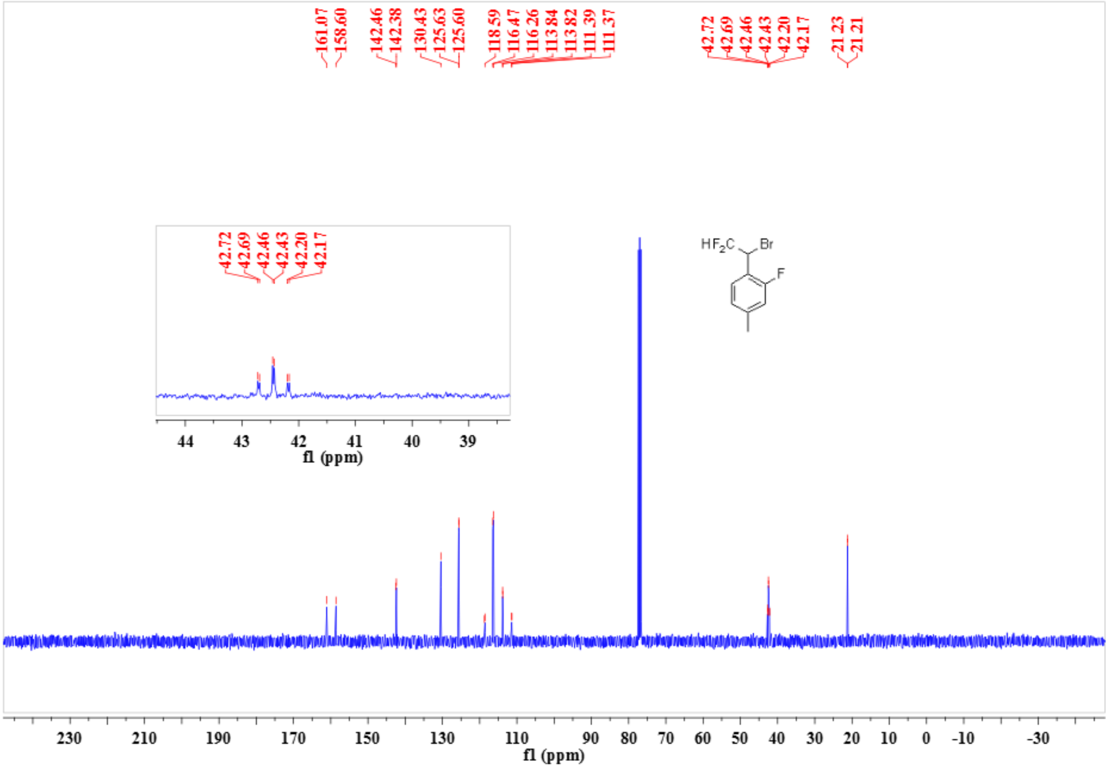

Supplementary Figure 210. $^{13}C$ NMR (101 MHz, $CDCl_3$) spectrum of 1aa

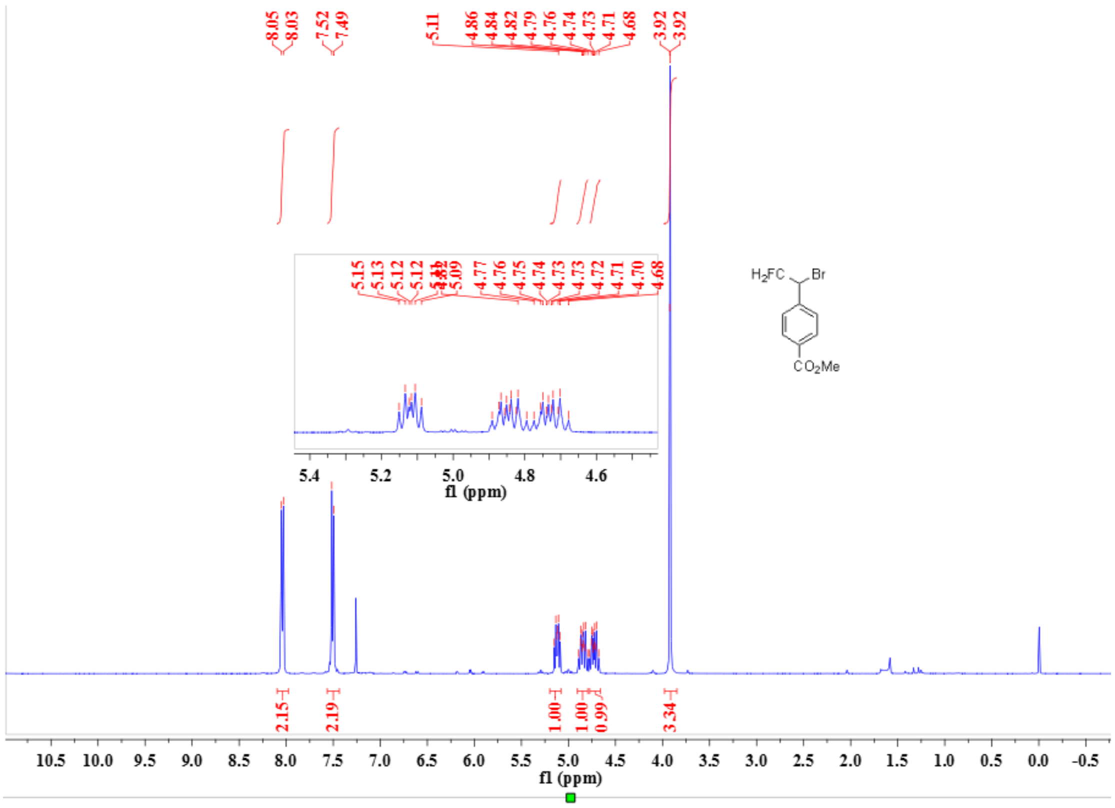

**Supplementary Figure 211.** $^{1}H$ **NMR (400 MHz,** $CDCl_3$**) spectrum of 1ab**

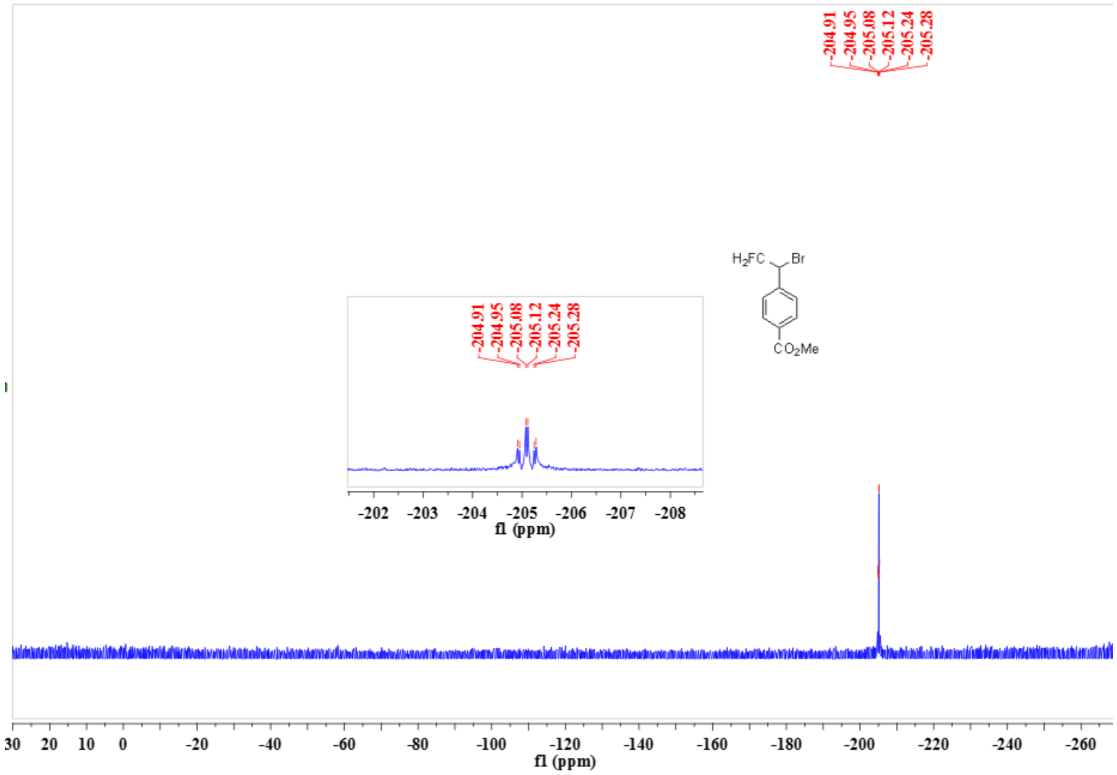

**Supplementary Figure 212.** $^{19}F$ **NMR (376 MHz,** $CDCl_3$**) spectrum of 1ab**

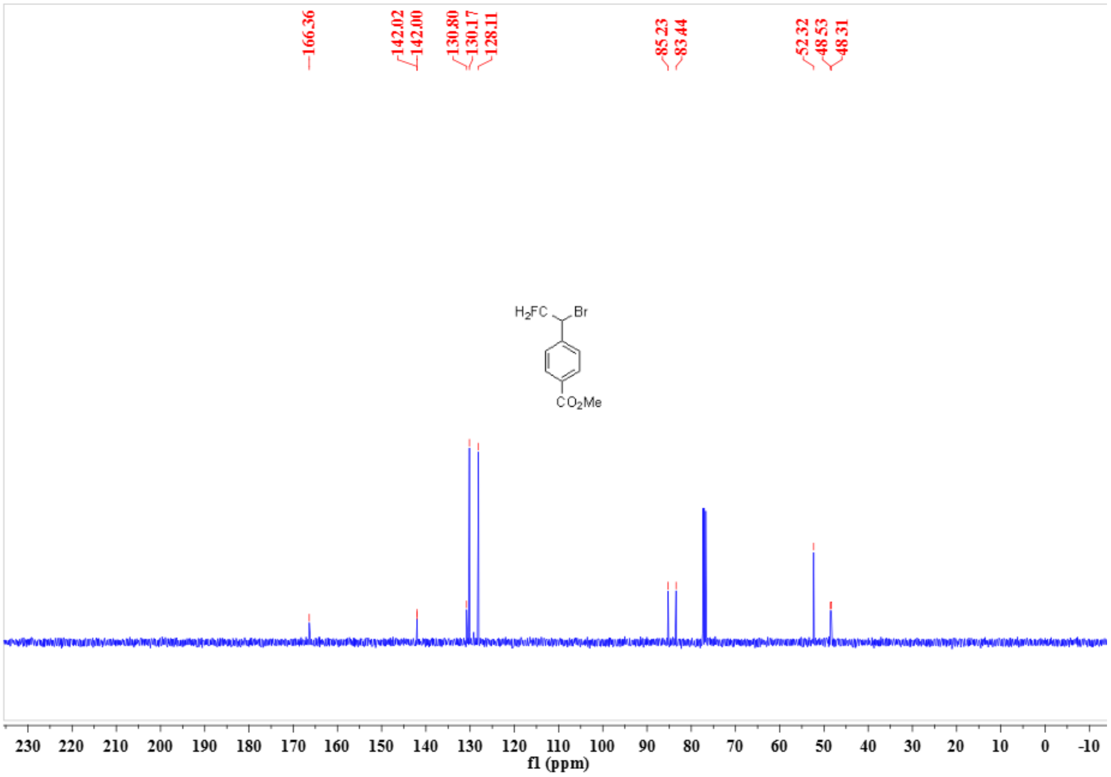

**Supplementary Figure 213. $^{13}$C NMR (101 MHz, $CDCl_3$) spectrum of 1ab**

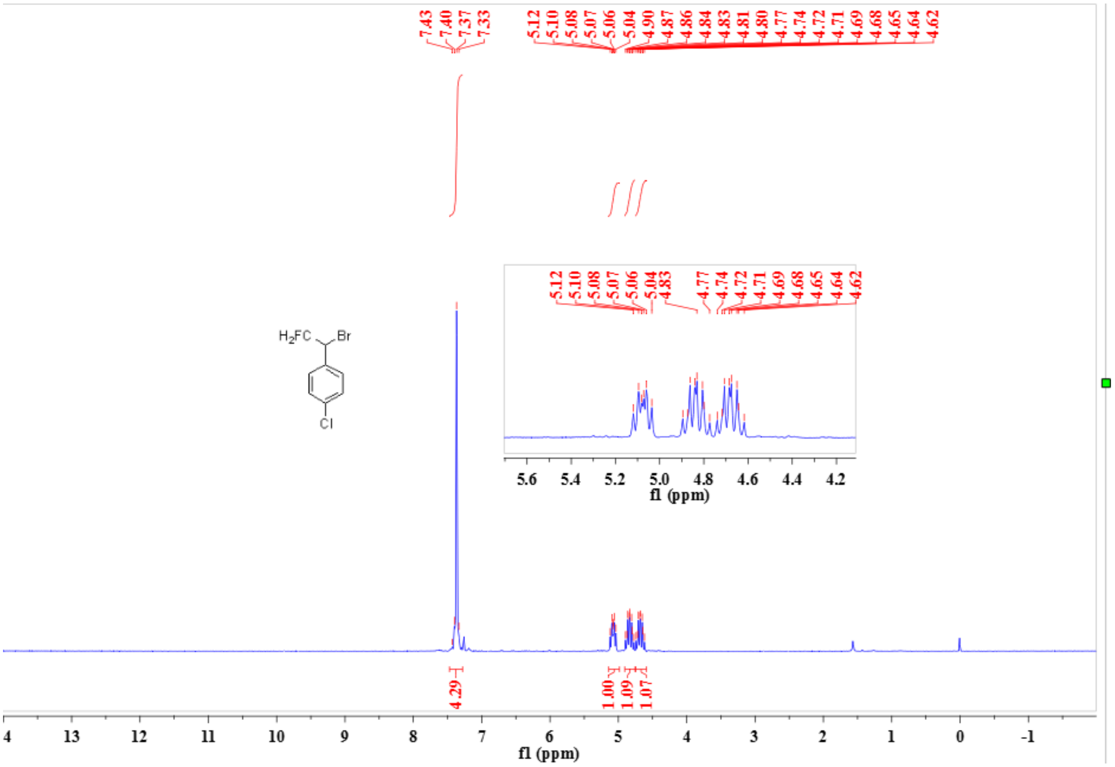

**Supplementary Figure 214. $^{1}$H NMR (400 MHz, $CDCl_3$) spectrum of 1ac**

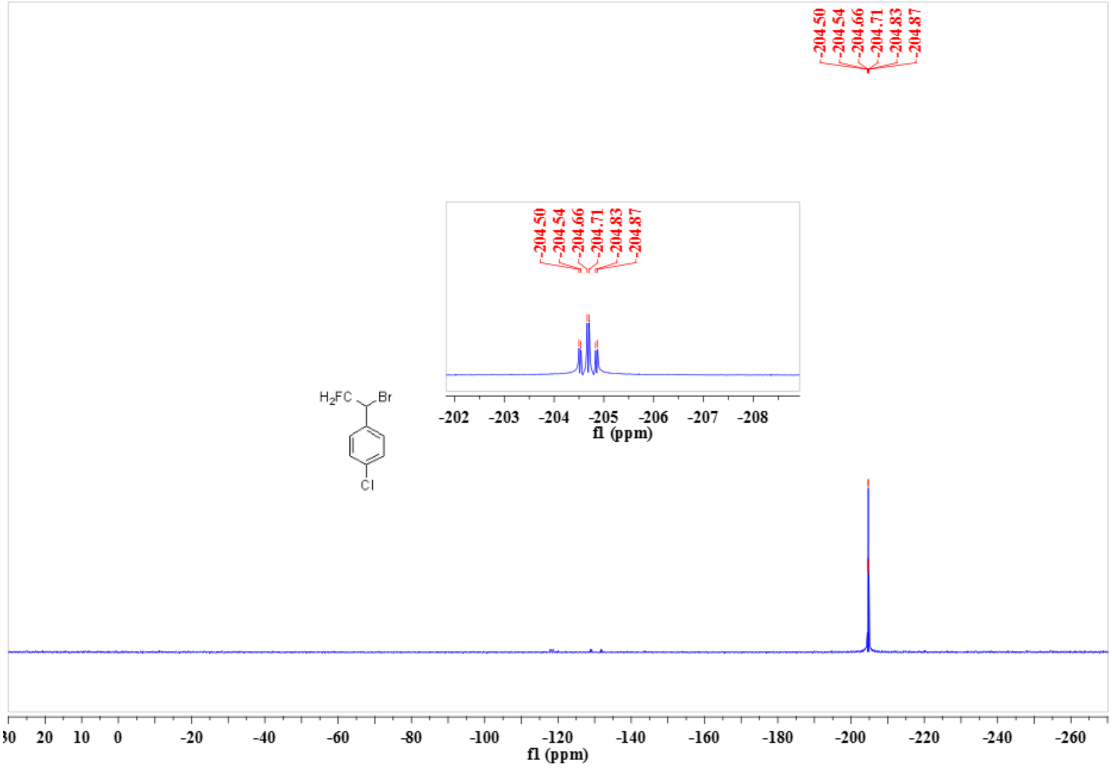


**Supplementary Figure 215.** $^{19}$**F NMR (376 MHz, CDCl$_3$) spectrum of 1ac**

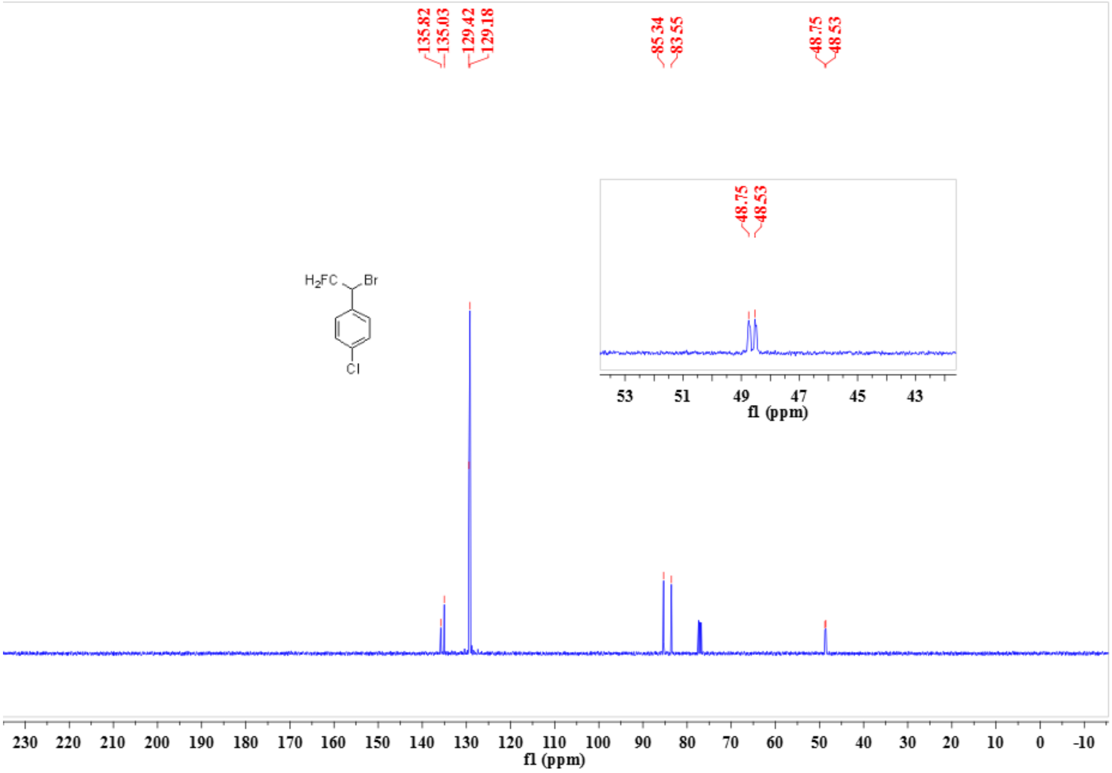


**Supplementary Figure 216.** $^{13}$**C NMR (101 MHz, CDCl$_3$) spectrum of 1ac**

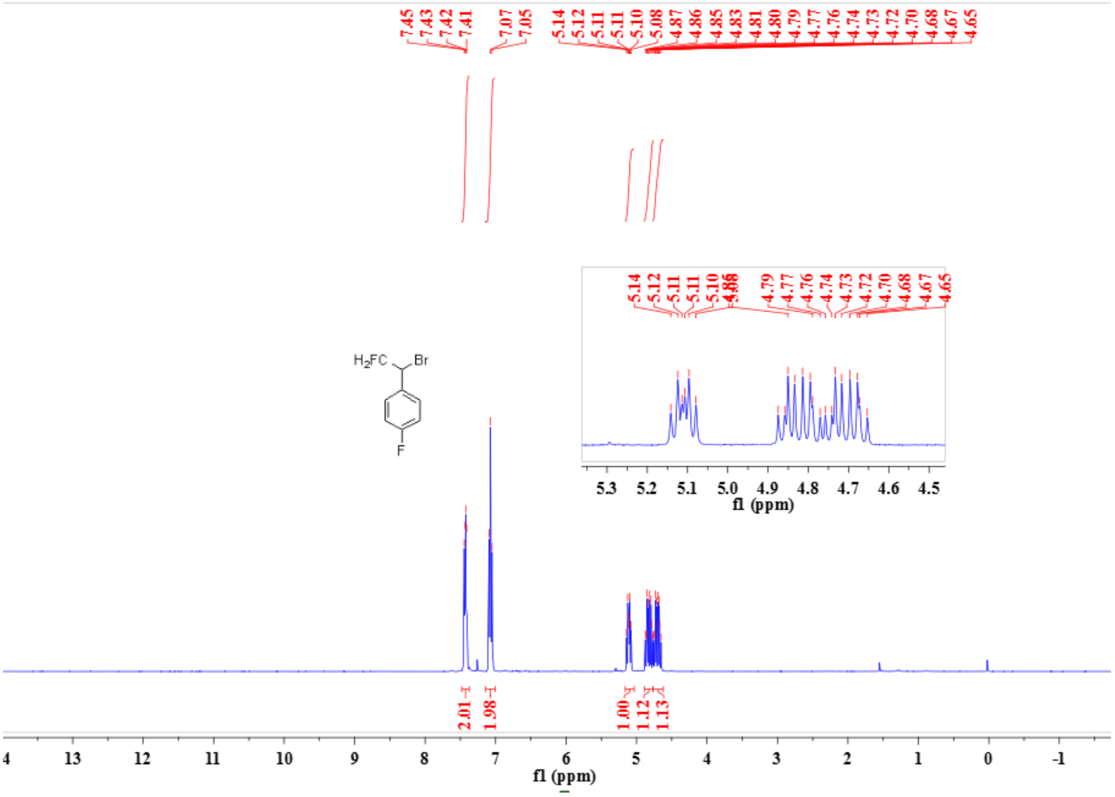

**Supplementary Figure 217. $^{1}$H NMR (400 MHz, $CDCl_3$) spectrum of 1ad**

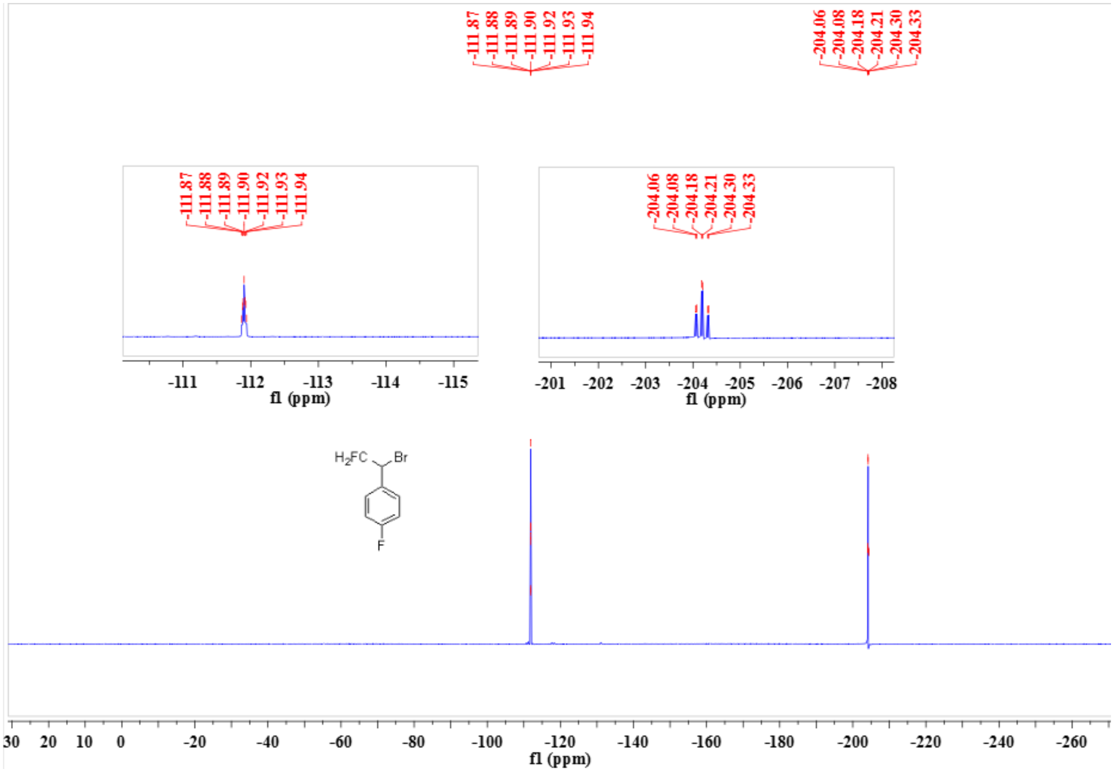

**Supplementary Figure 218. $^{19}$F NMR (376 MHz, $CDCl_3$) spectrum of 1ad**

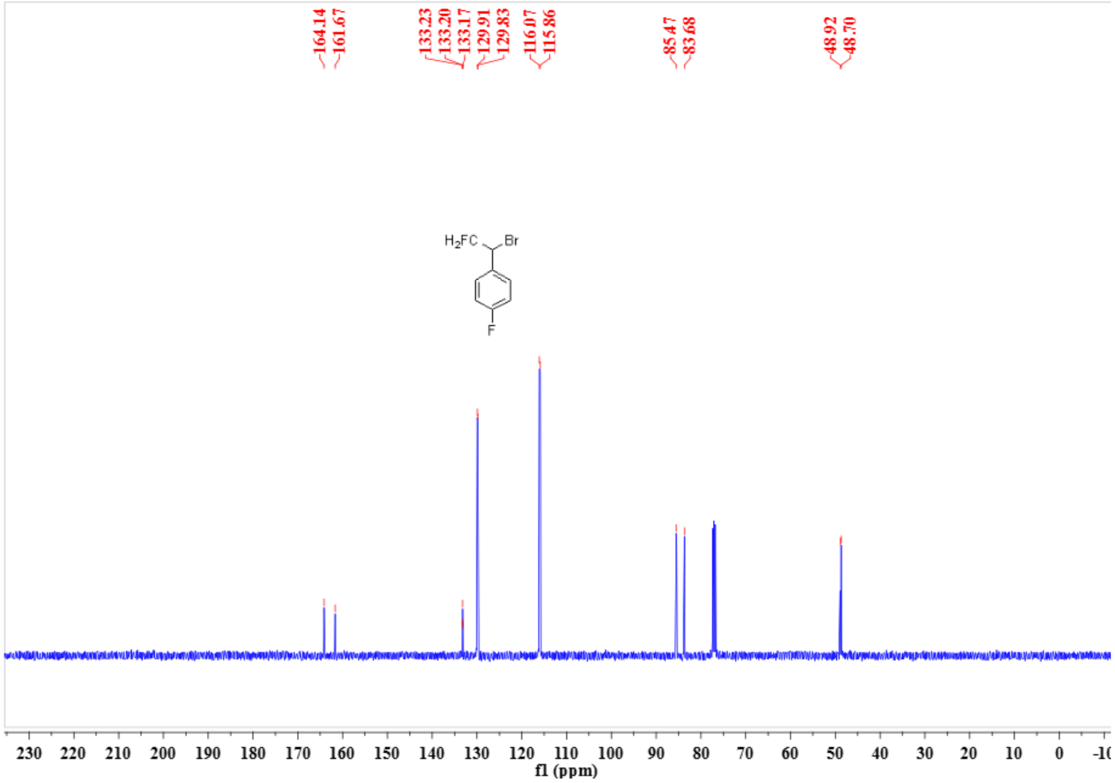


**Supplementary Figure 219.** $^{13}$C NMR (101 MHz, $CDCl_3$) spectrum of 1ad

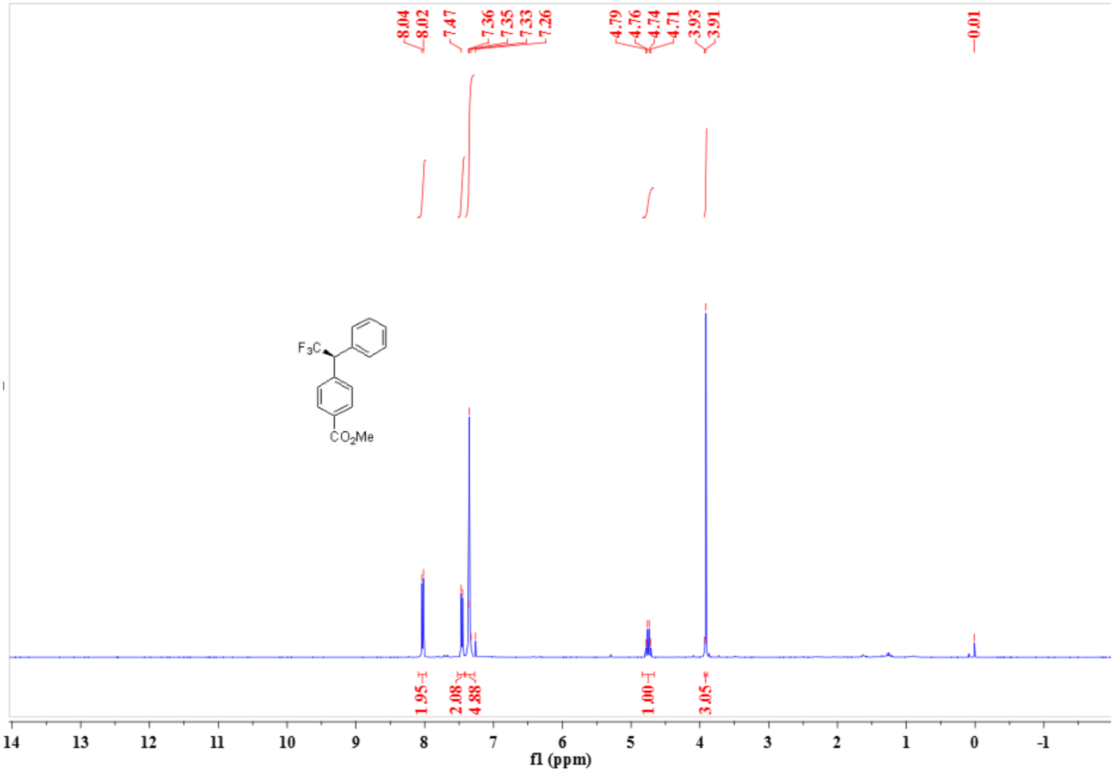


**Supplementary Figure 220.** $^{1}$H NMR (400 MHz, $CDCl_3$) spectrum of 3a

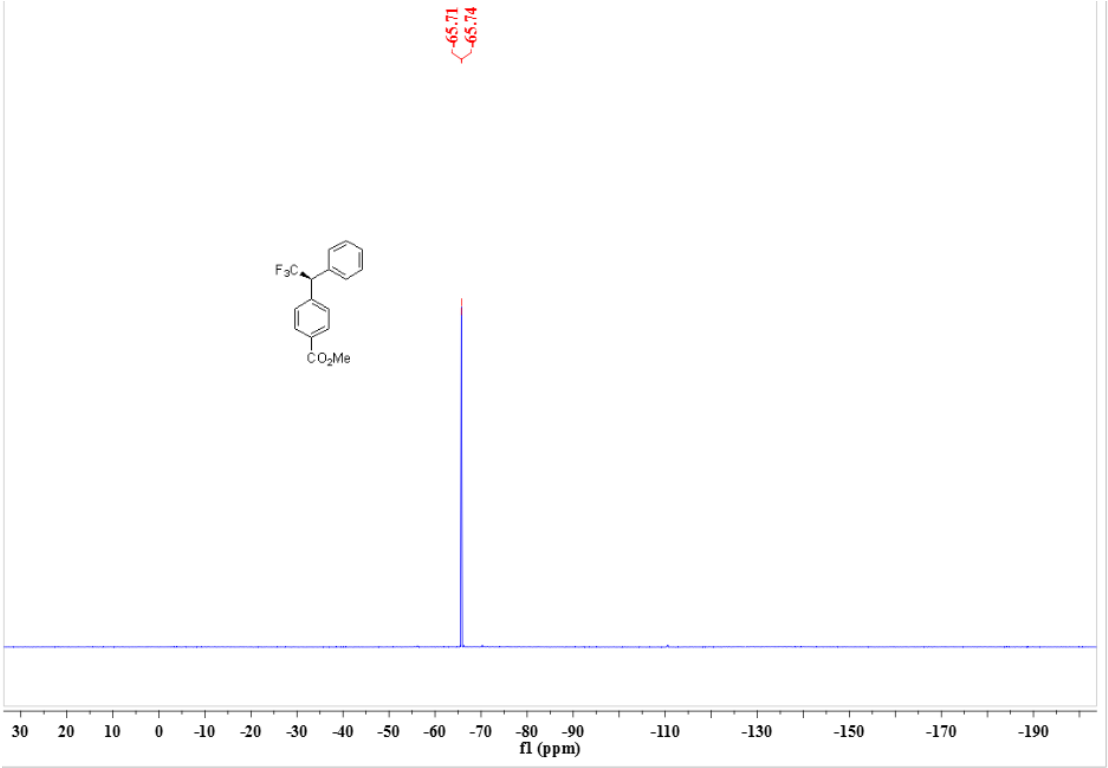


**Supplementary Figure 221.** $^{19}$**F NMR (376 MHz, CDCl$_3$) spectrum of 3a**

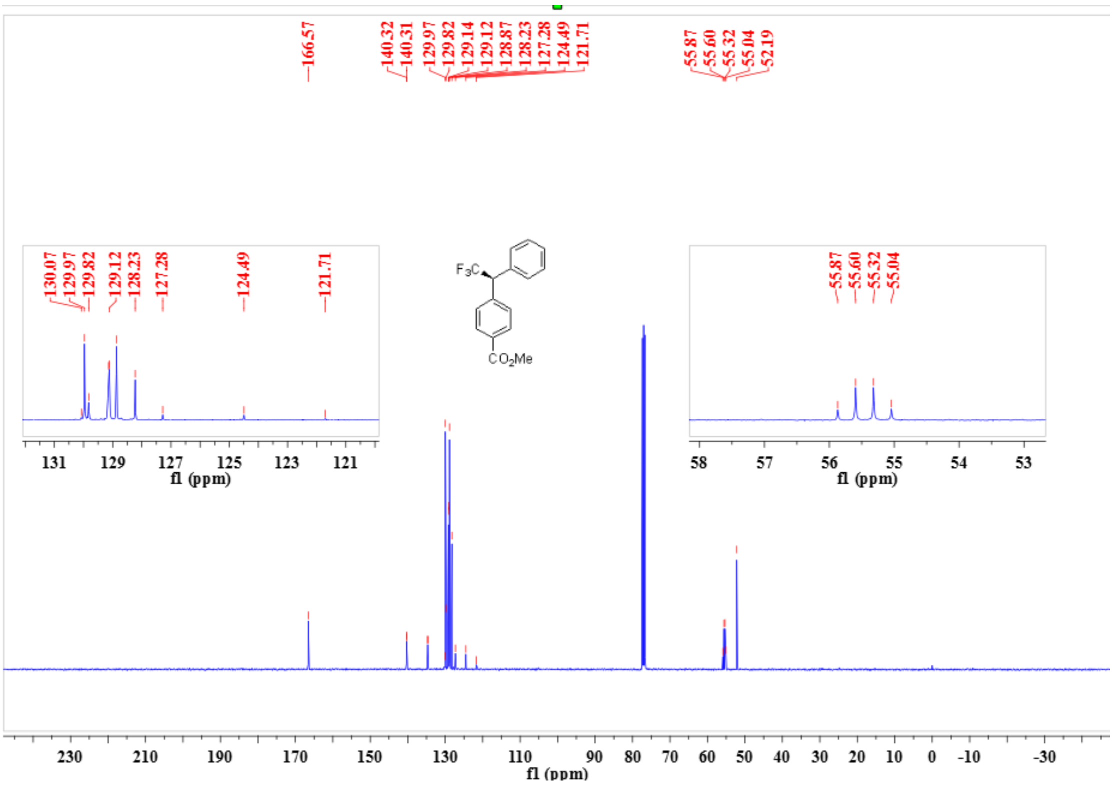


**Supplementary Figure 222.** $^{13}$**C NMR (101 MHz, CDCl$_3$) spectrum of 3a**

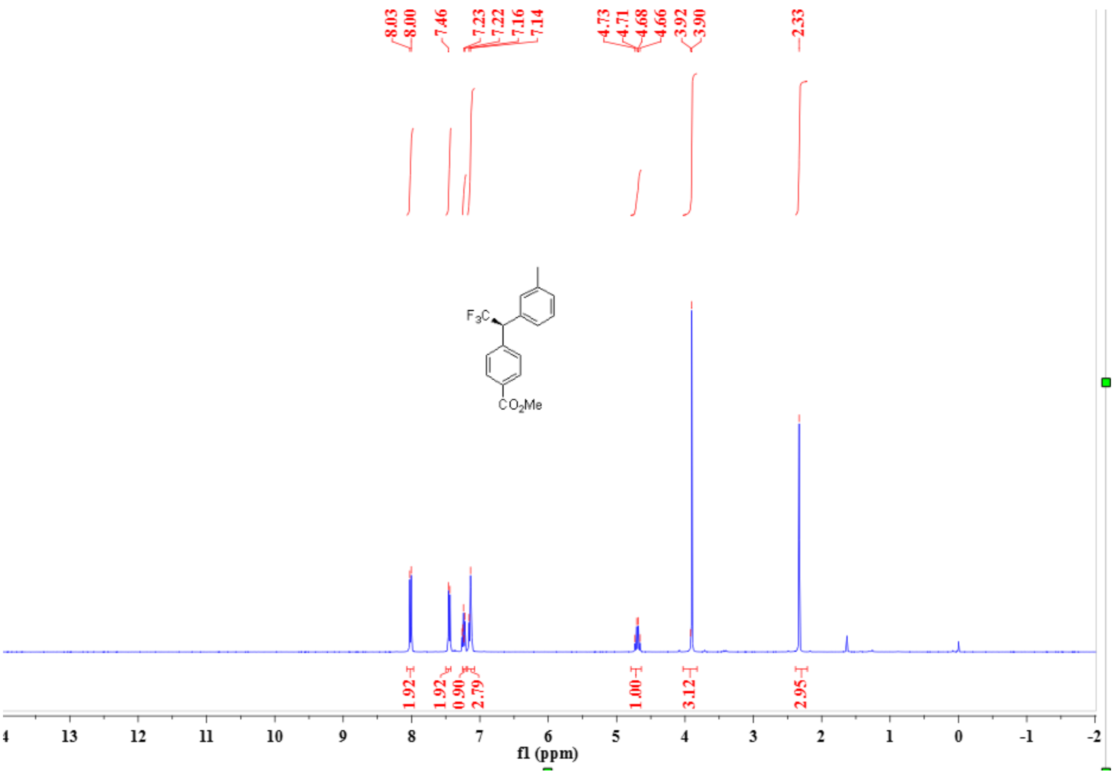


Supplementary Figure 223. $^{1}H$ NMR (400 MHz, $CDCl_3$) spectrum of 3b

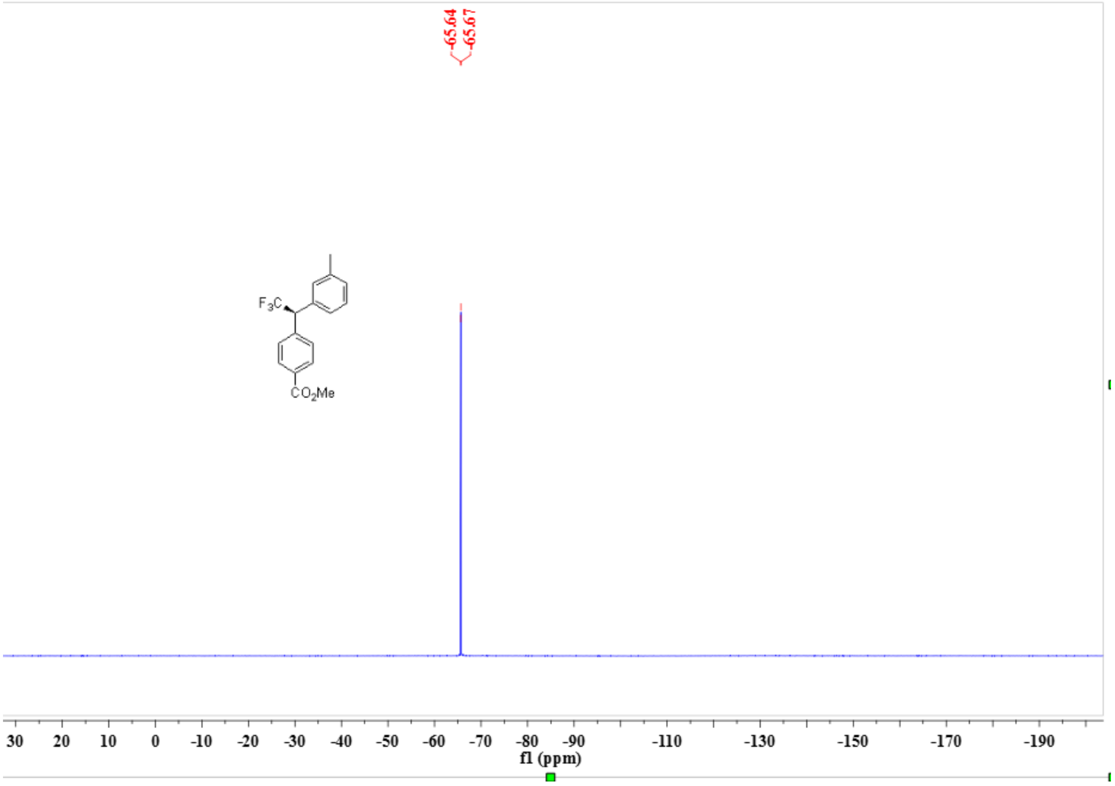


Supplementary Figure 224. $^{19}F$ NMR (376 MHz, $CDCl_3$) spectrum of 3b

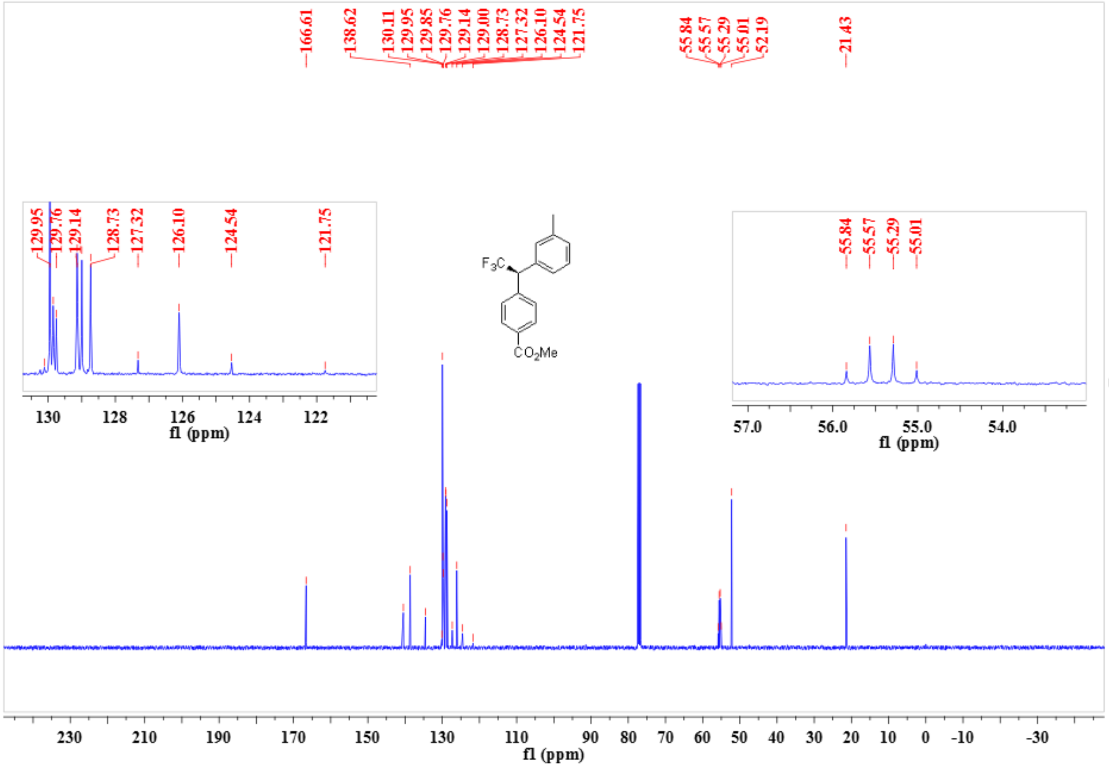

Supplementary Figure 225. $^{13}C$ NMR (101 MHz, $CDCl_3$) spectrum of 3b

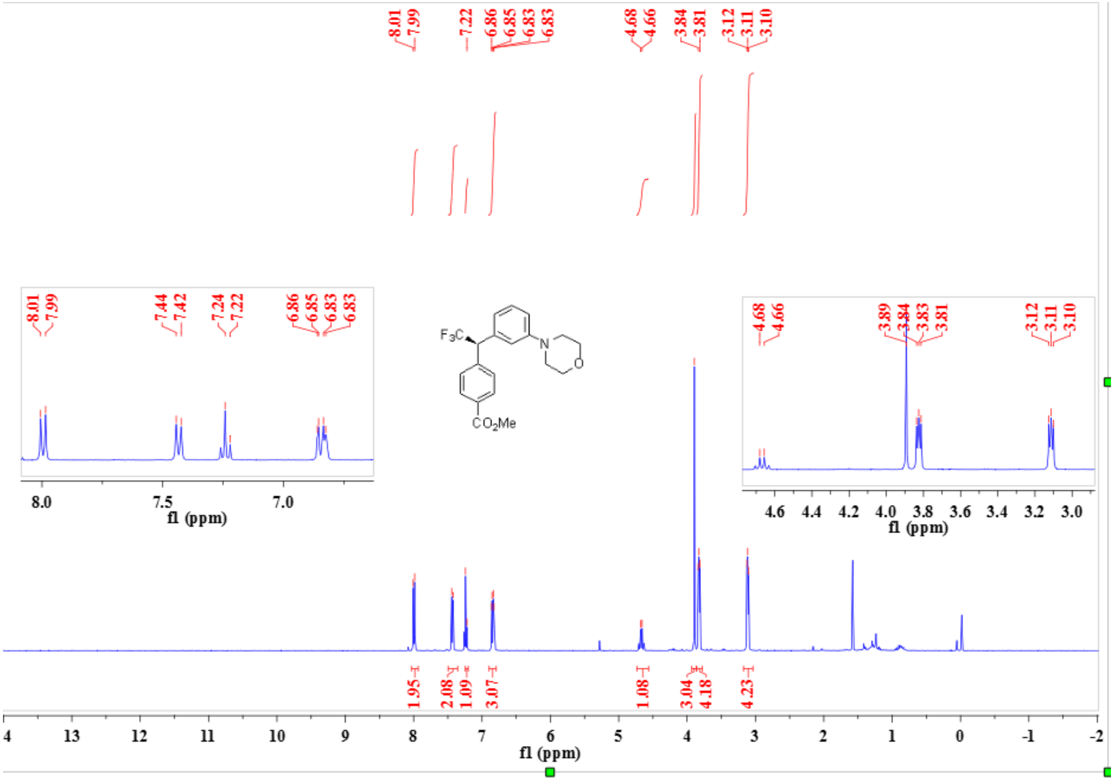

Supplementary Figure 226. $^{1}H$ NMR (400 MHz, $CDCl_3$) spectrum of 3c

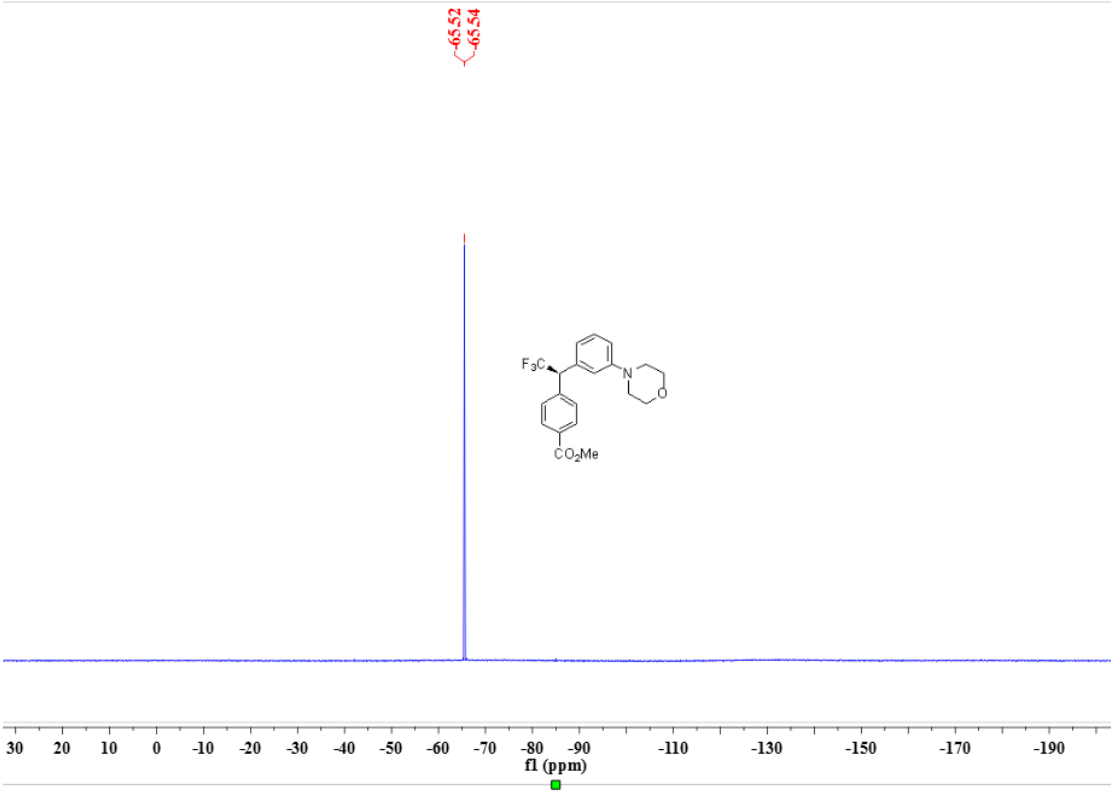


**Supplementary Figure 227.** $^{19}$**F NMR (376 MHz, CDCl$_3$) spectrum of 3c**

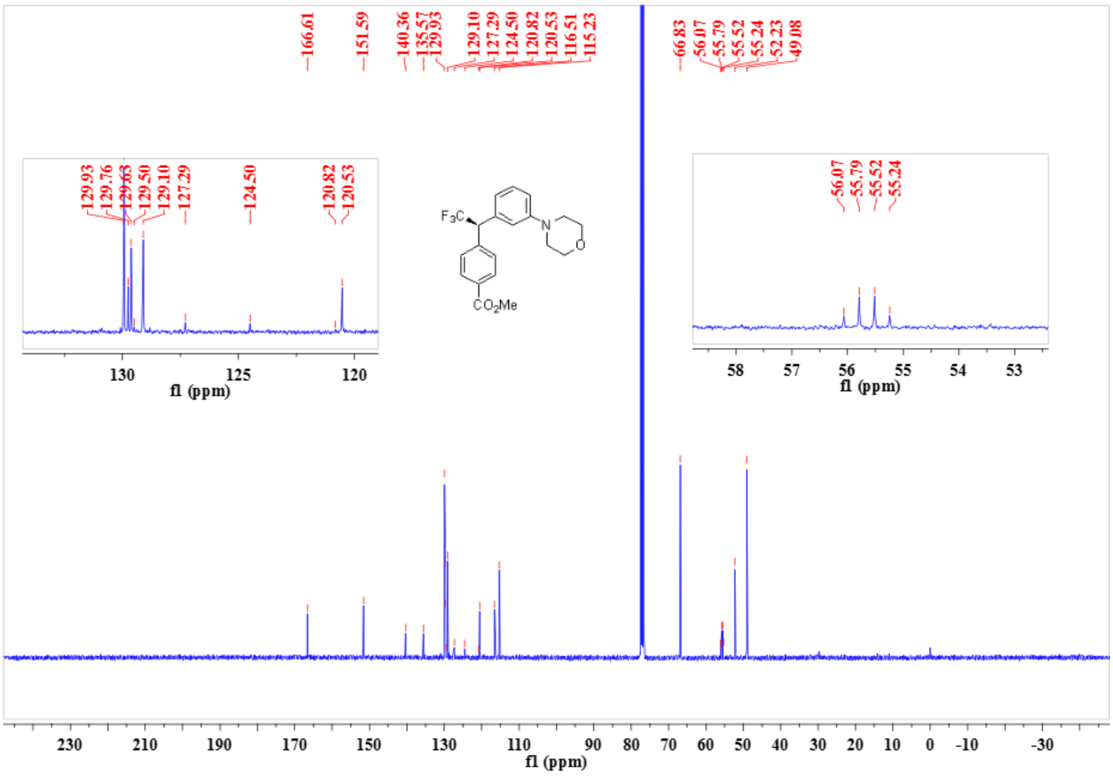


**Supplementary Figure 228.** $^{13}$**C NMR (101 MHz, CDCl$_3$) spectrum of 3c**

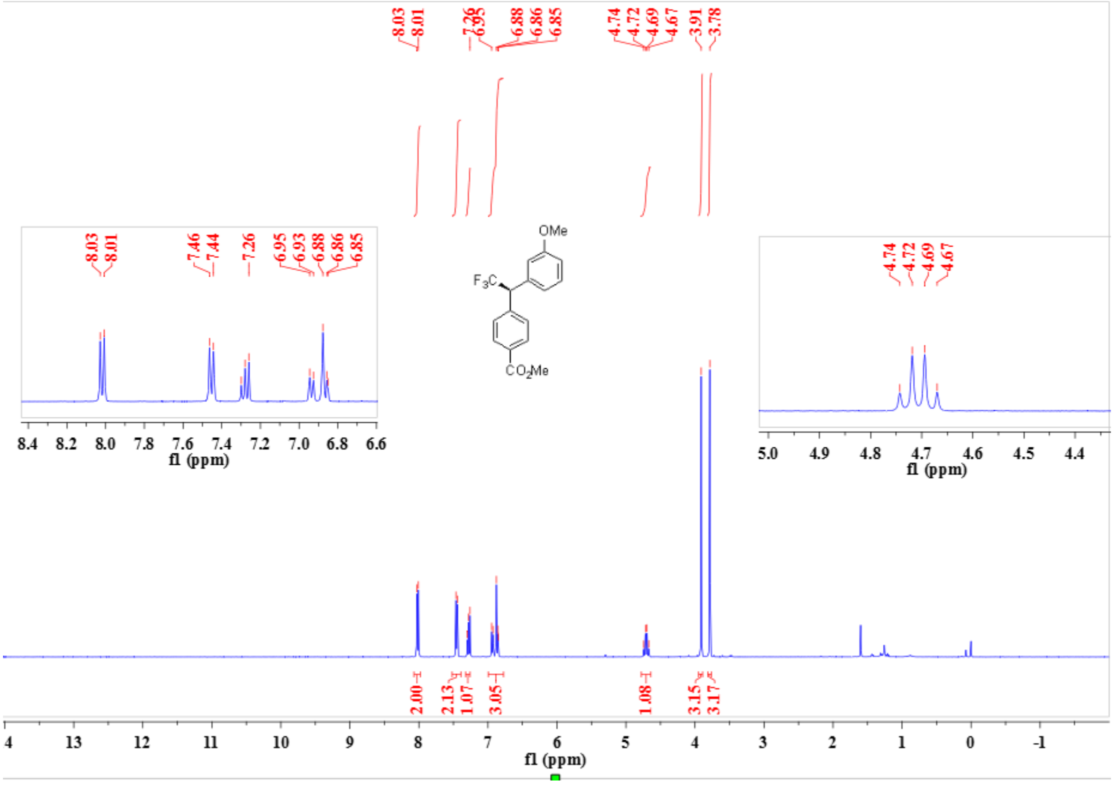

**Supplementary Figure 229.** **$^{1}$H NMR (400 MHz, $CDCl_3$) spectrum of 3d**

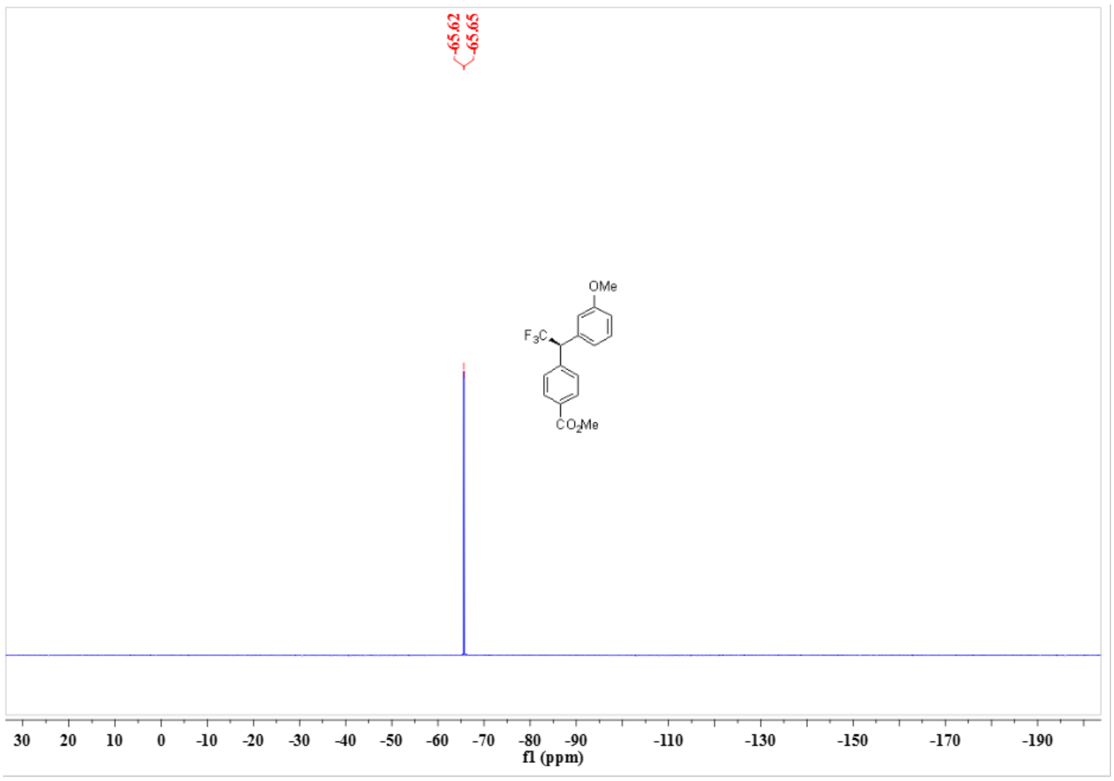

**Supplementary Figure 230.** **$^{19}$F NMR (376 MHz, $CDCl_3$) spectrum of 3d**

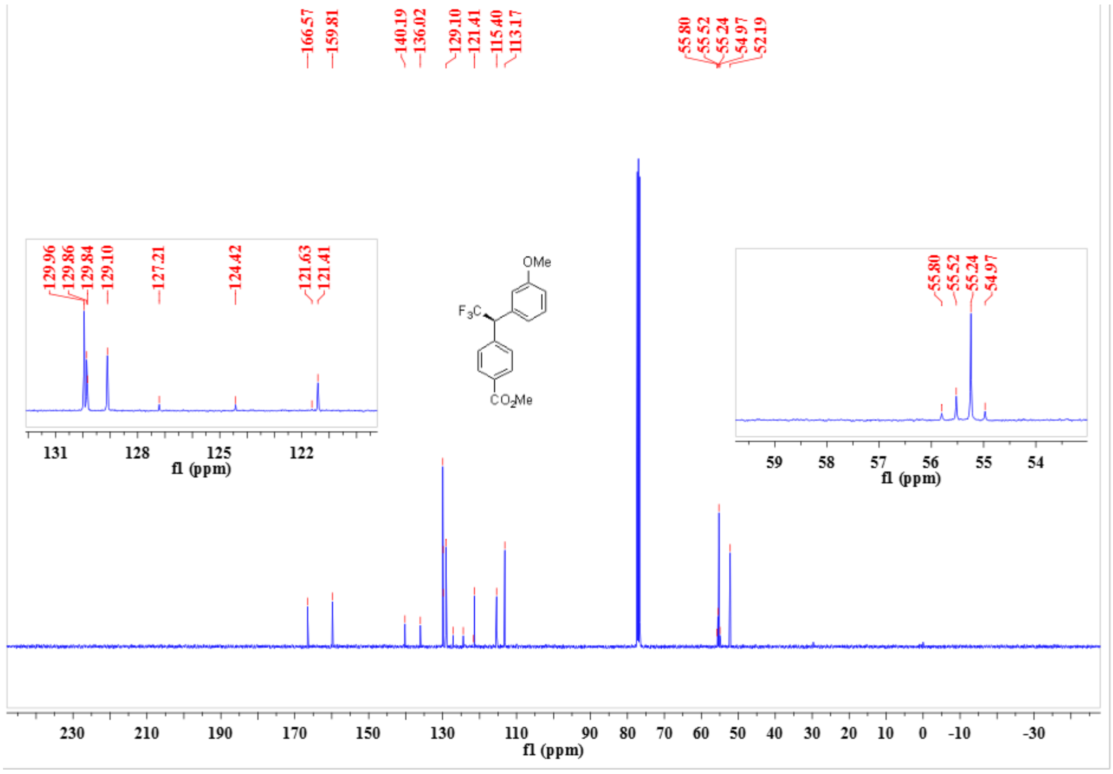


Supplementary Figure 231. $^{13}C$ NMR (101 MHz, $CDCl_3$) spectrum of 3d

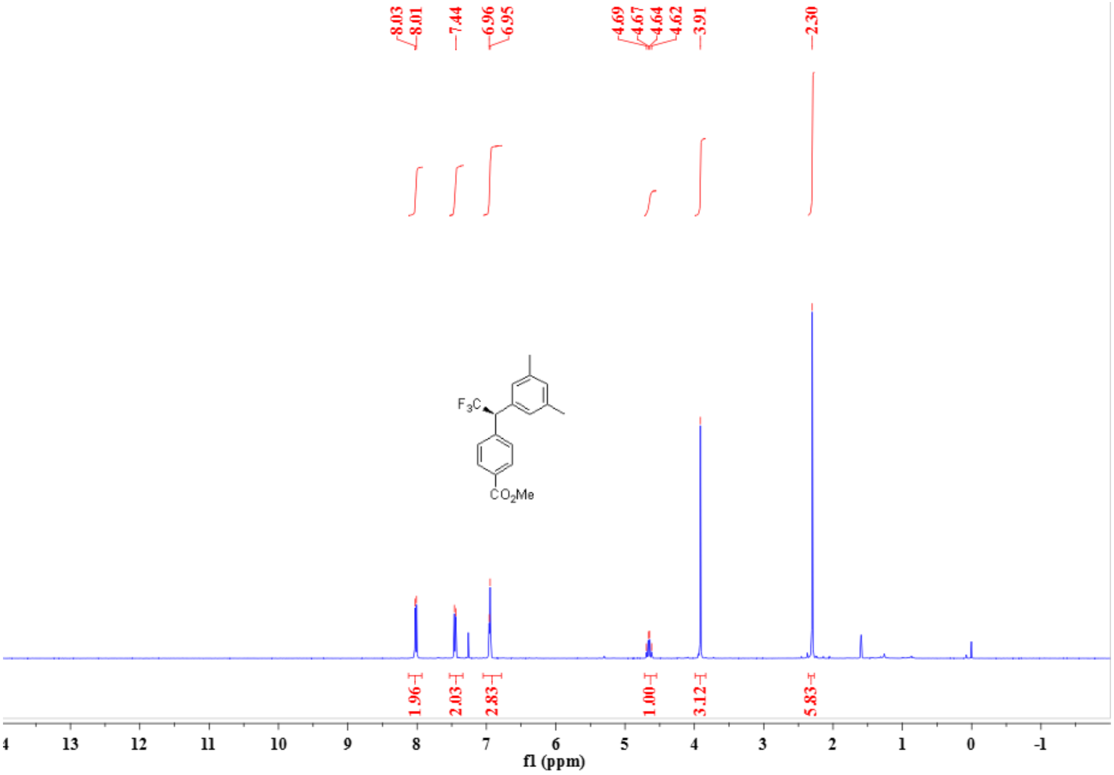


Supplementary Figure 232. $^{1}H$ NMR (400 MHz, $CDCl_3$) spectrum of 3e

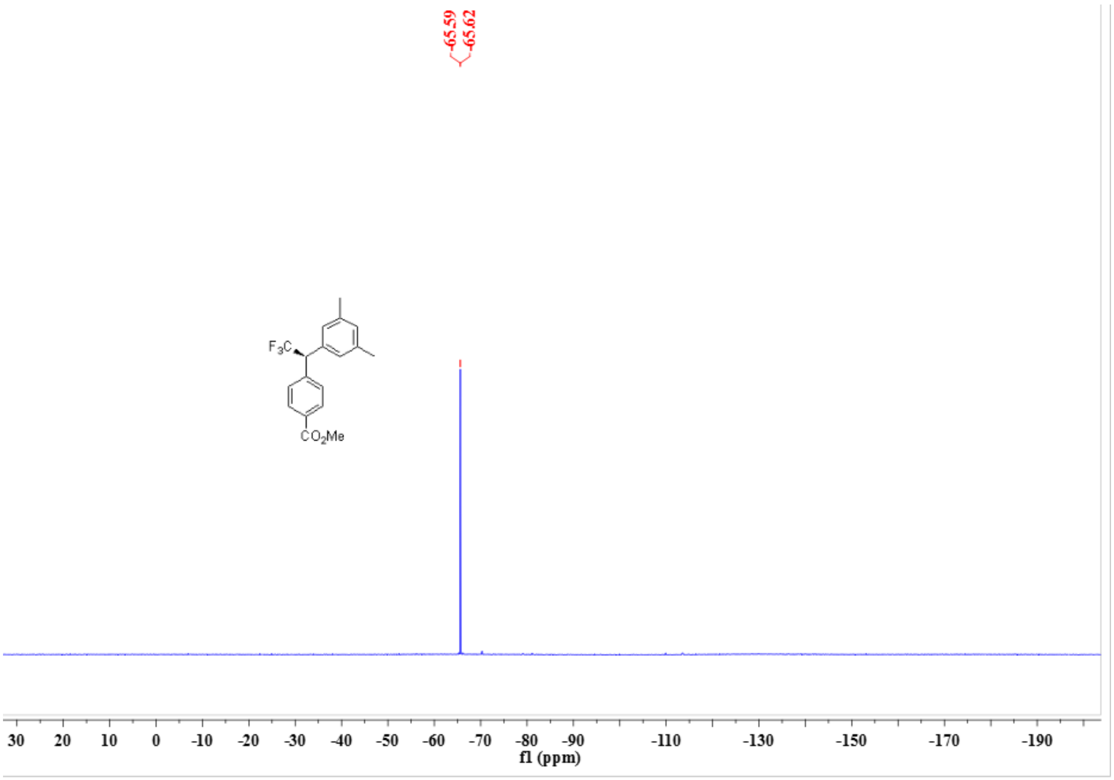


**Supplementary Figure 233.** $^{19}$F NMR (376 MHz, $CDCl_3$) spectrum of 3e

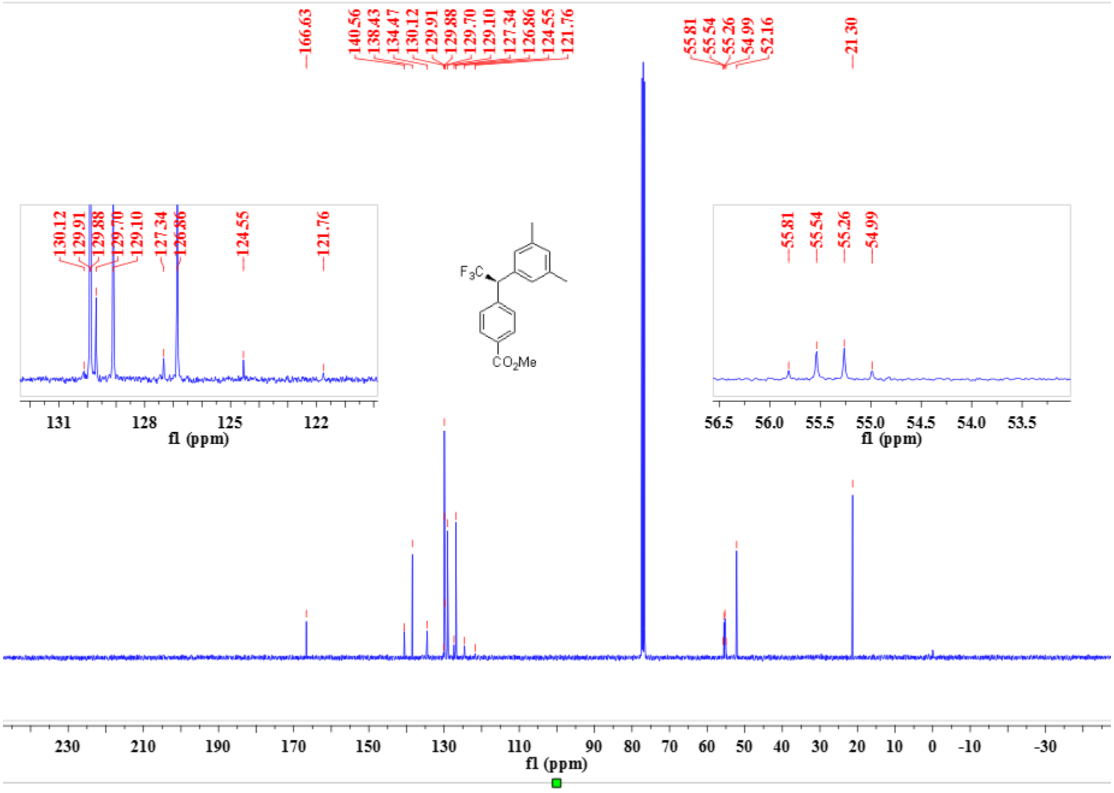


**Supplementary Figure 234.** $^{13}$C NMR (101 MHz, $CDCl_3$) spectrum of 3e

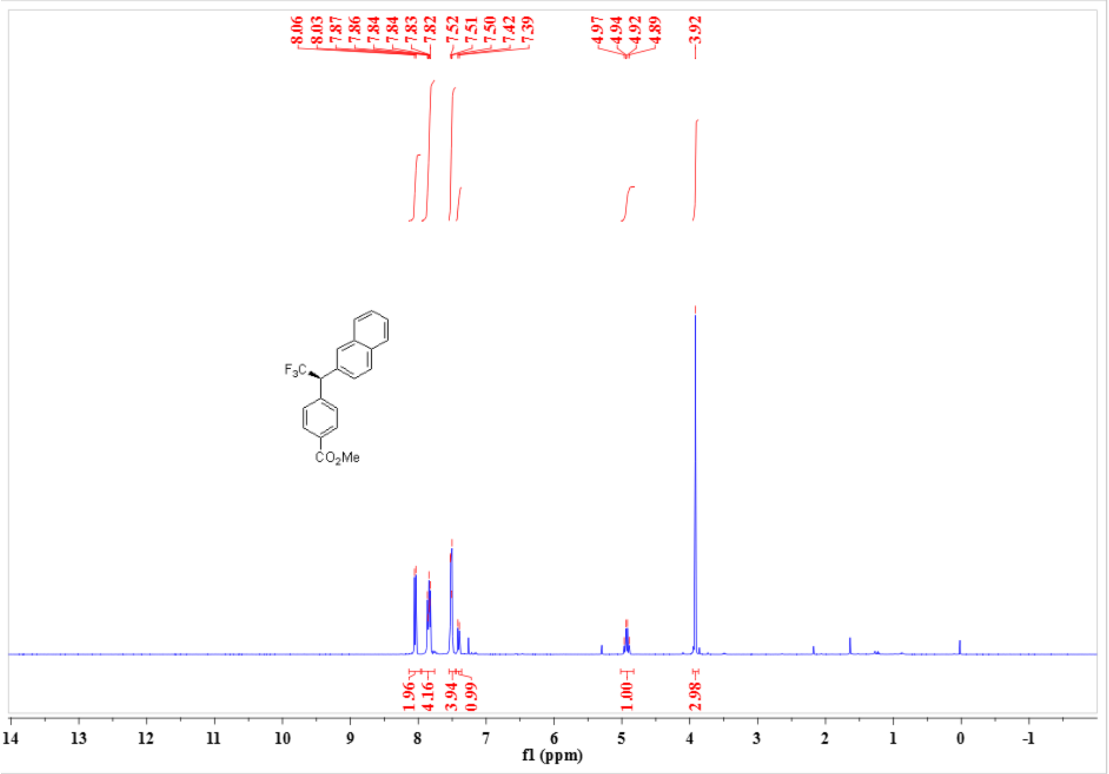


**Supplementary Figure 235.** $^{1}$**H NMR (400 MHz, CDCl₃) spectrum of 3f**

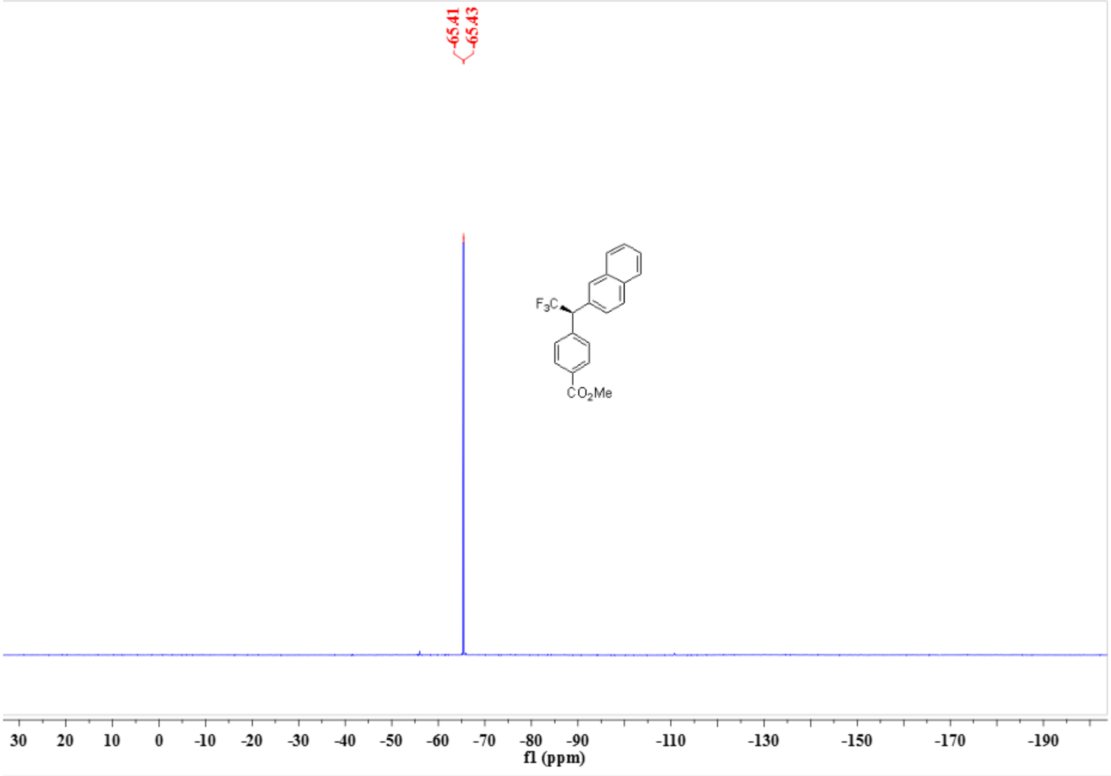


**Supplementary Figure 236.** $^{19}$**F NMR (376 MHz, CDCl₃) spectrum of 3f**

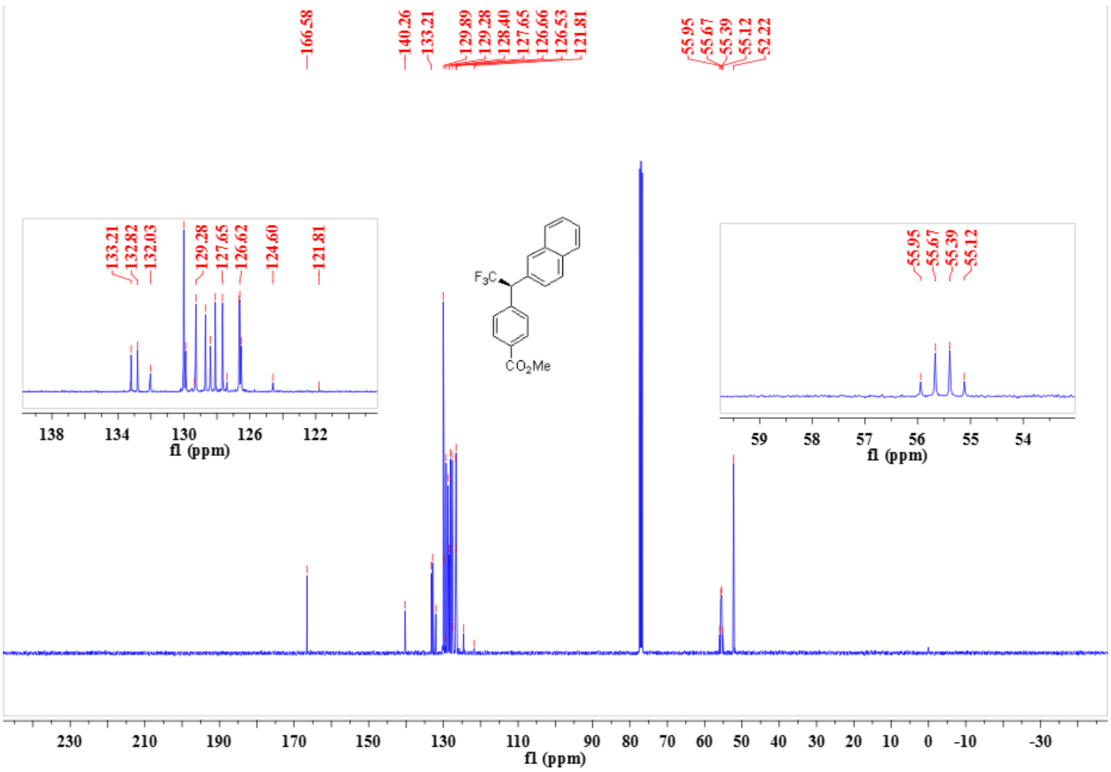


Supplementary Figure 237. $^{13}C$ NMR (101 MHz, $CDCl_3$) spectrum of 3f

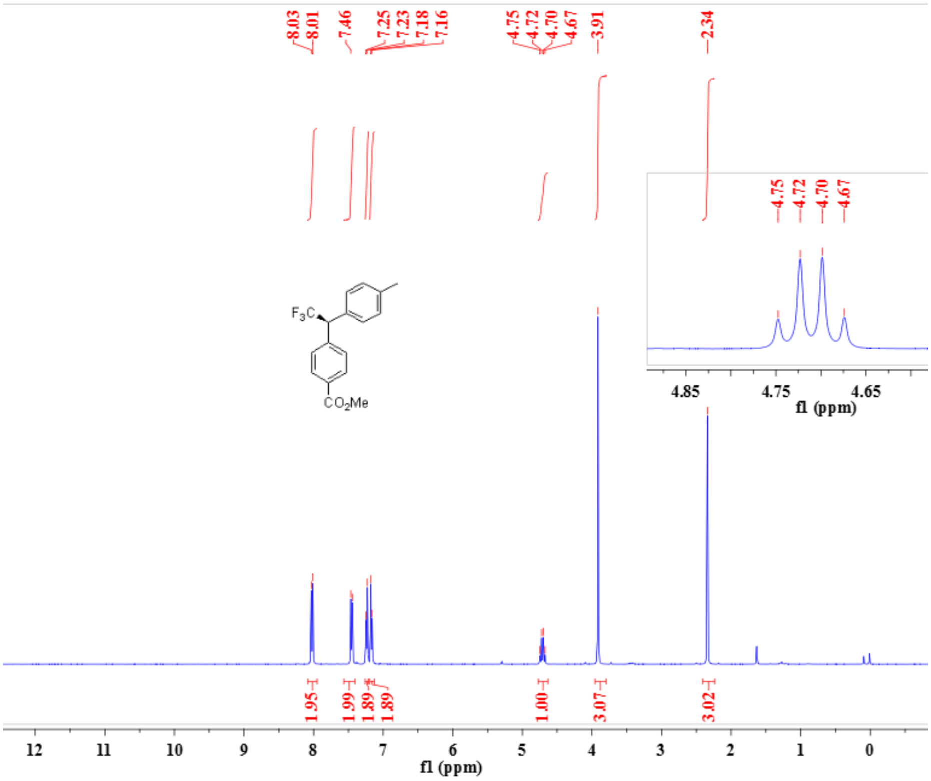


Supplementary Figure 238. $^{1}H$ NMR (400 MHz, $CDCl_3$) spectrum of 3g

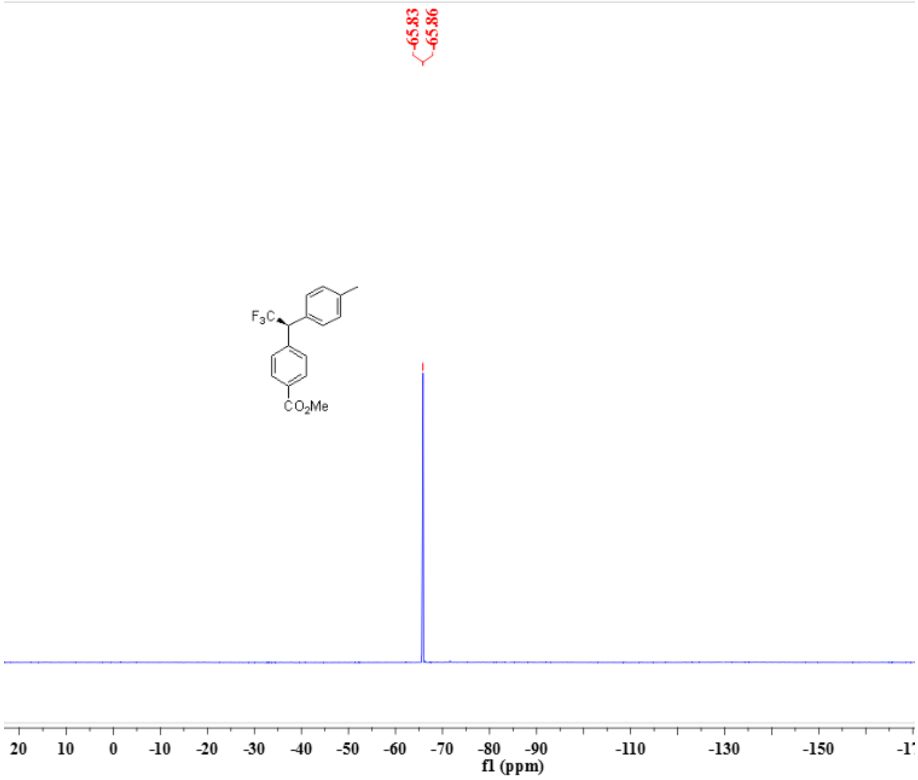


**Supplementary Figure 239.** $^{19}$**F NMR (376 MHz, CDCl**$_3$**) spectrum of 3g**

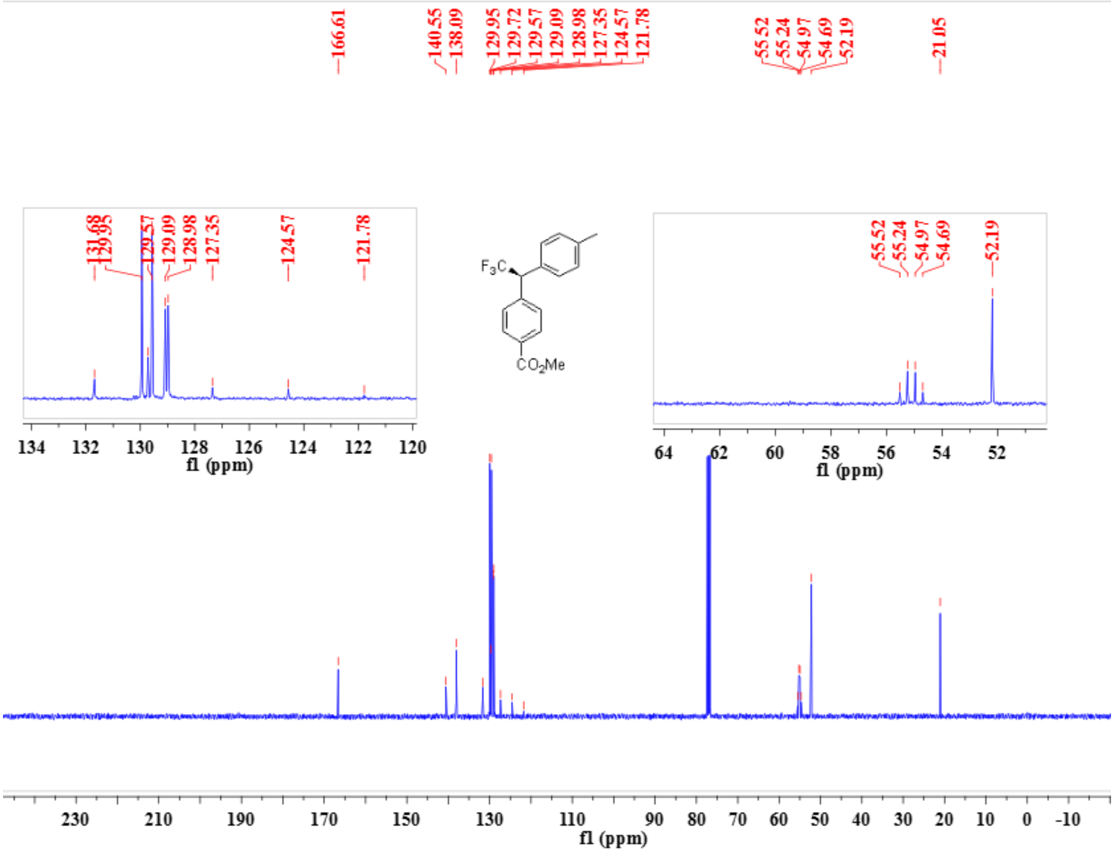


**Supplementary Figure 240.** $^{13}$**C NMR (101 MHz, CDCl**$_3$**) spectrum of 3g**

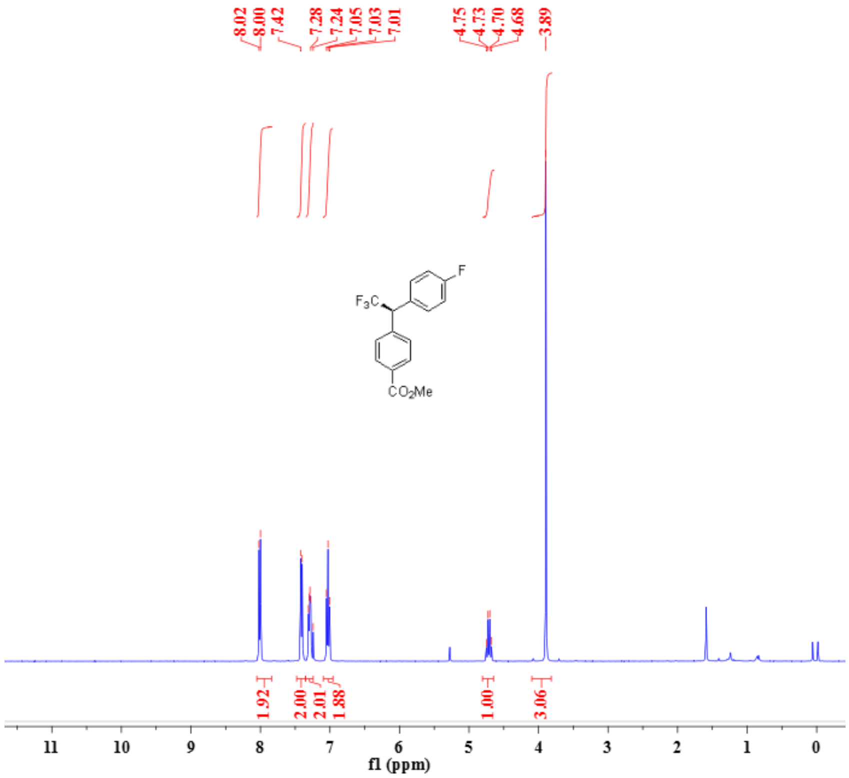

Supplementary Figure 241. $^{1}H$ NMR (400 MHz, $CDCl_3$) spectrum of 3h

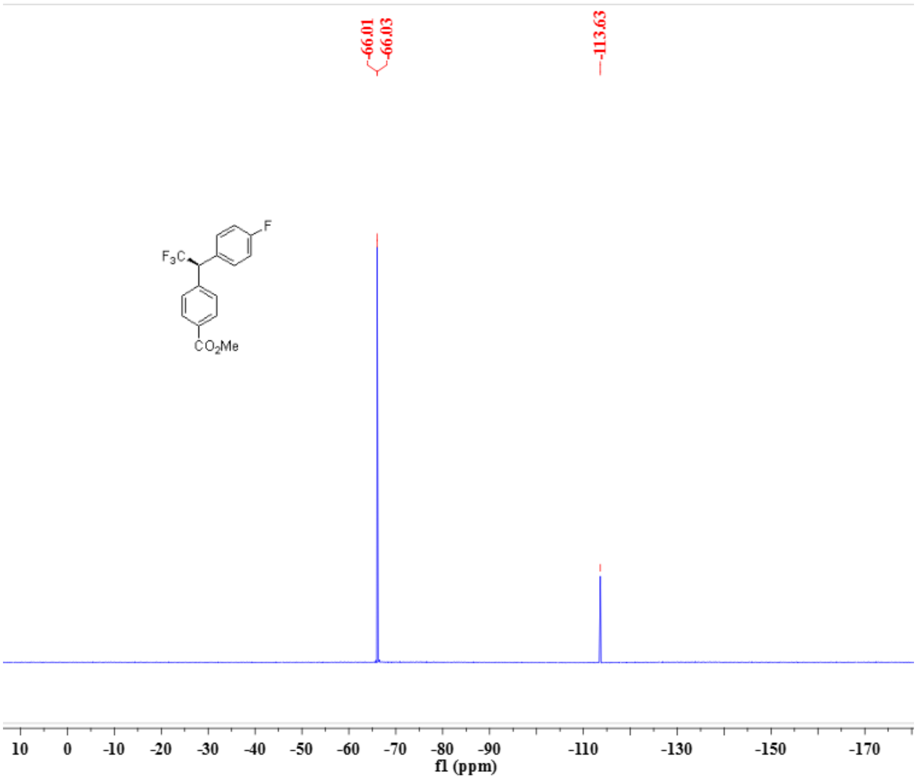

Supplementary Figure 242. $^{19}F$ NMR (376 MHz, $CDCl_3$) spectrum of 3h

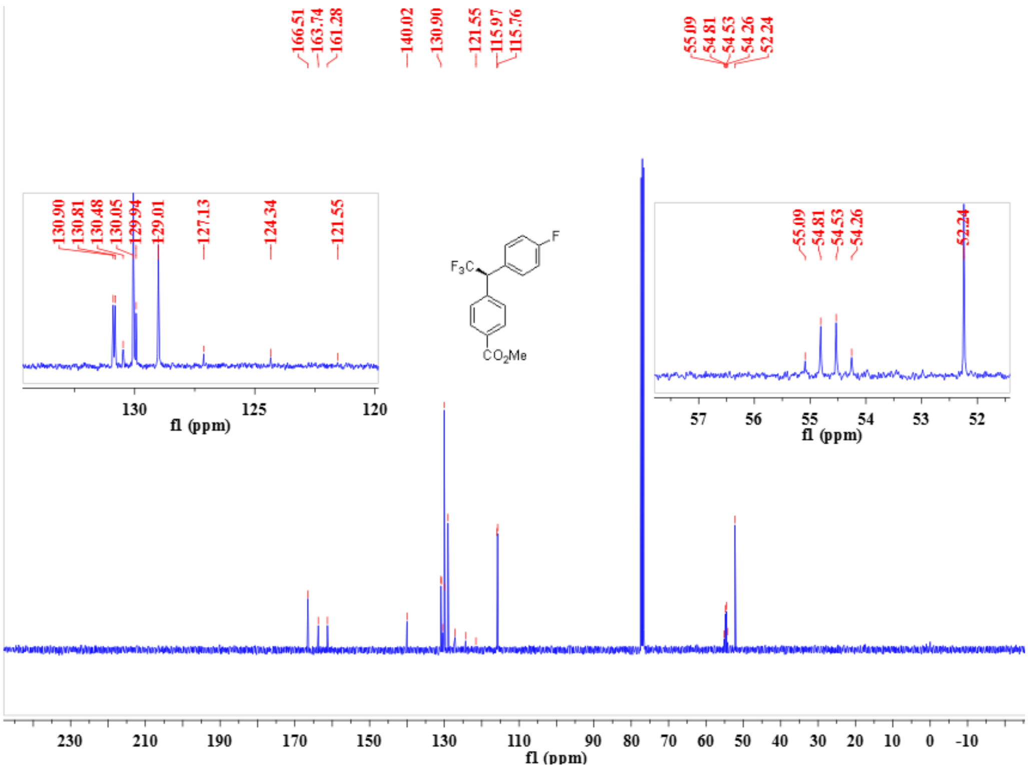

Supplementary Figure 243. $^{13}C$ NMR (101 MHz, $CDCl_3$) spectrum of 3h

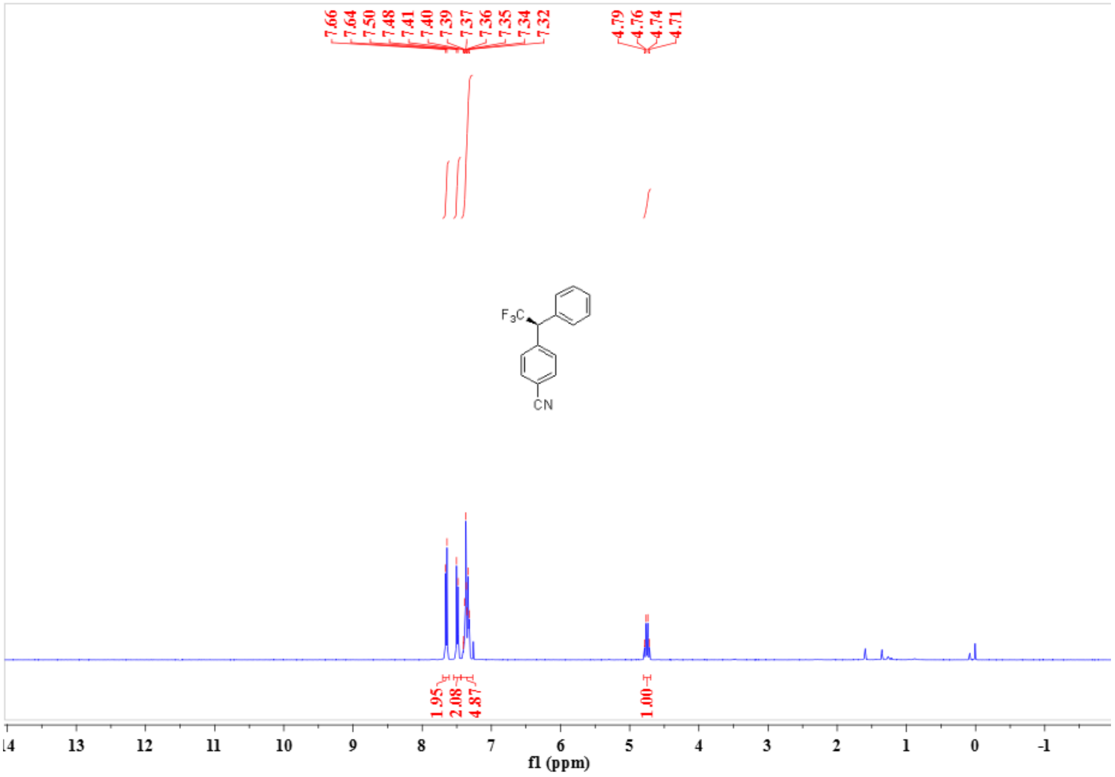

Supplementary Figure 244. $^{1}H$ NMR (400 MHz, $CDCl_3$) spectrum of 3j

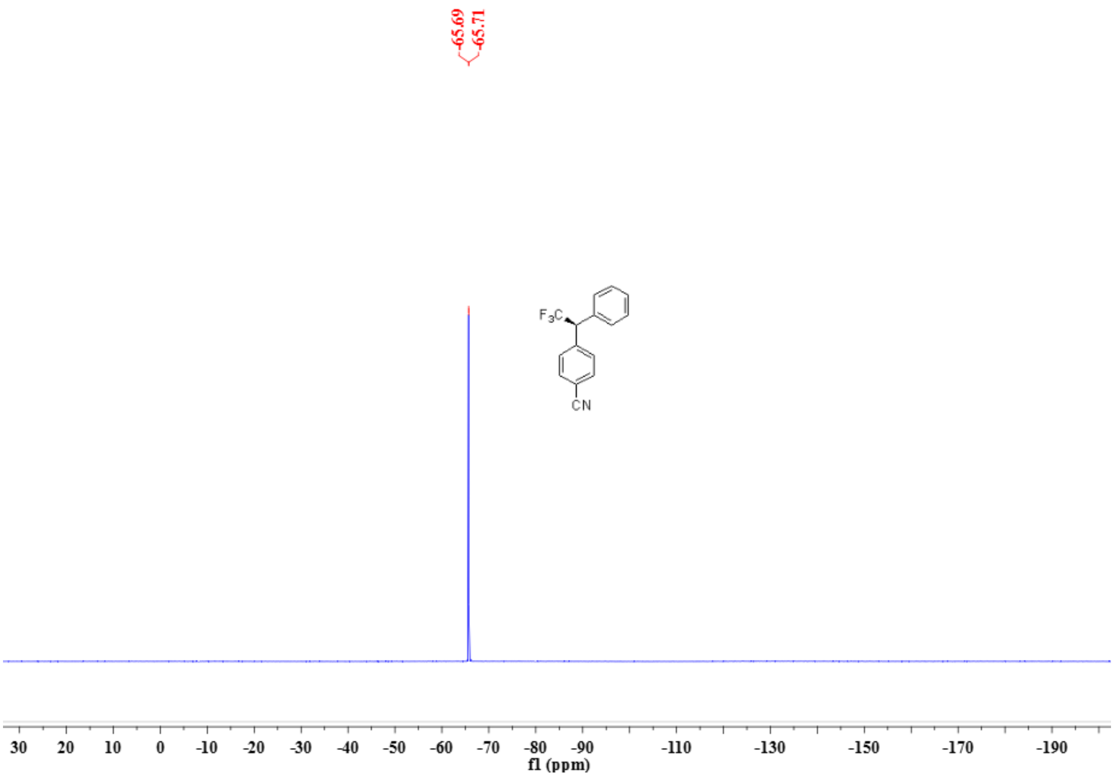


Supplementary Figure 245. $^{19}$F NMR (376 MHz, $CDCl_3$) spectrum of 3j

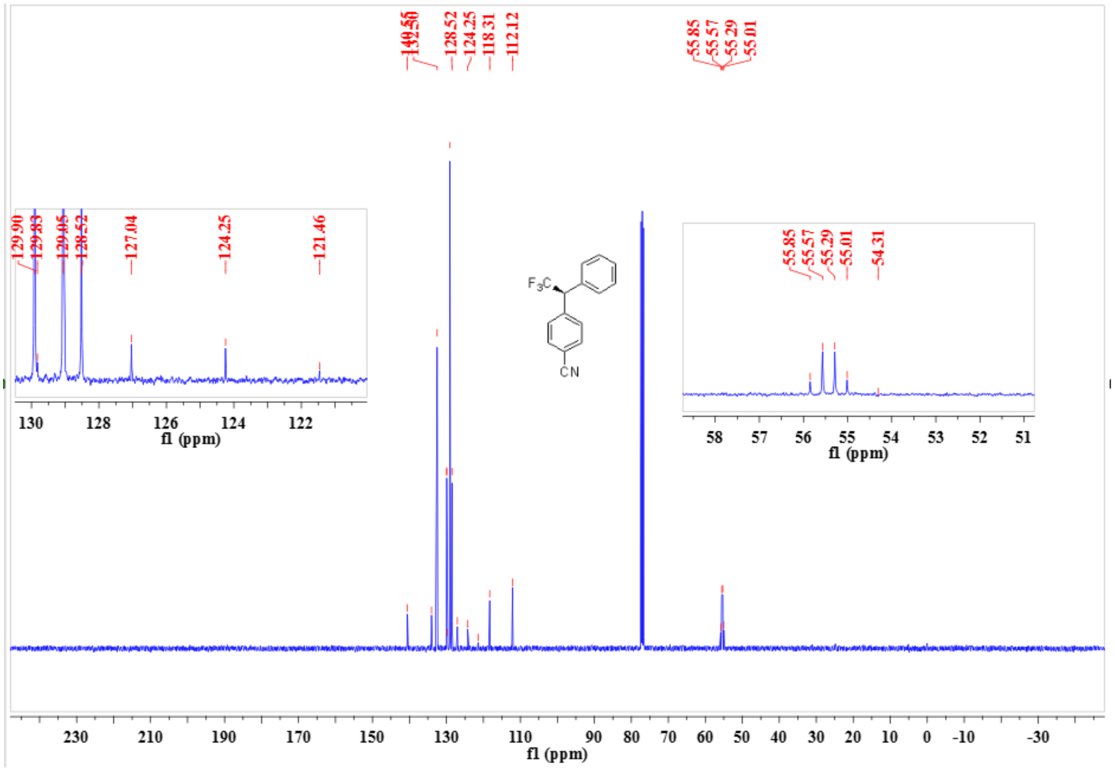


Supplementary Figure 246. $^{13}$C NMR (101 MHz, $CDCl_3$) spectrum of 3j

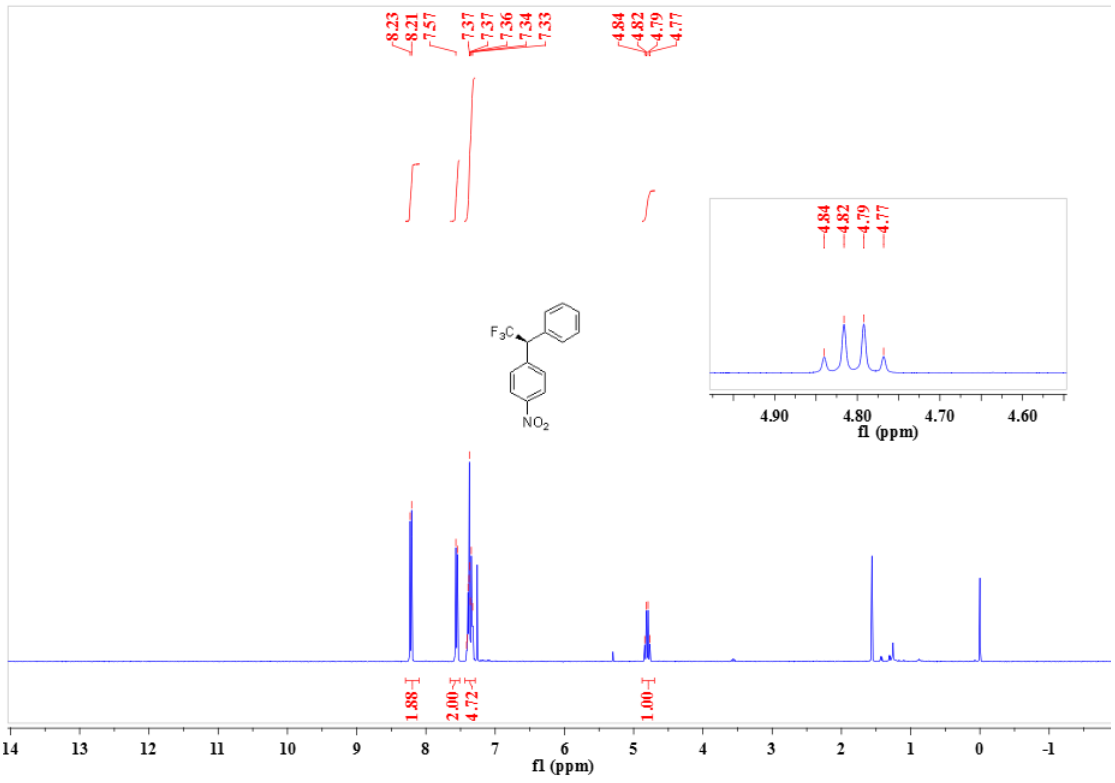


**Supplementary Figure 247.** $^{1}$**H NMR (400 MHz, CDCl$_3$) spectrum of 3k**

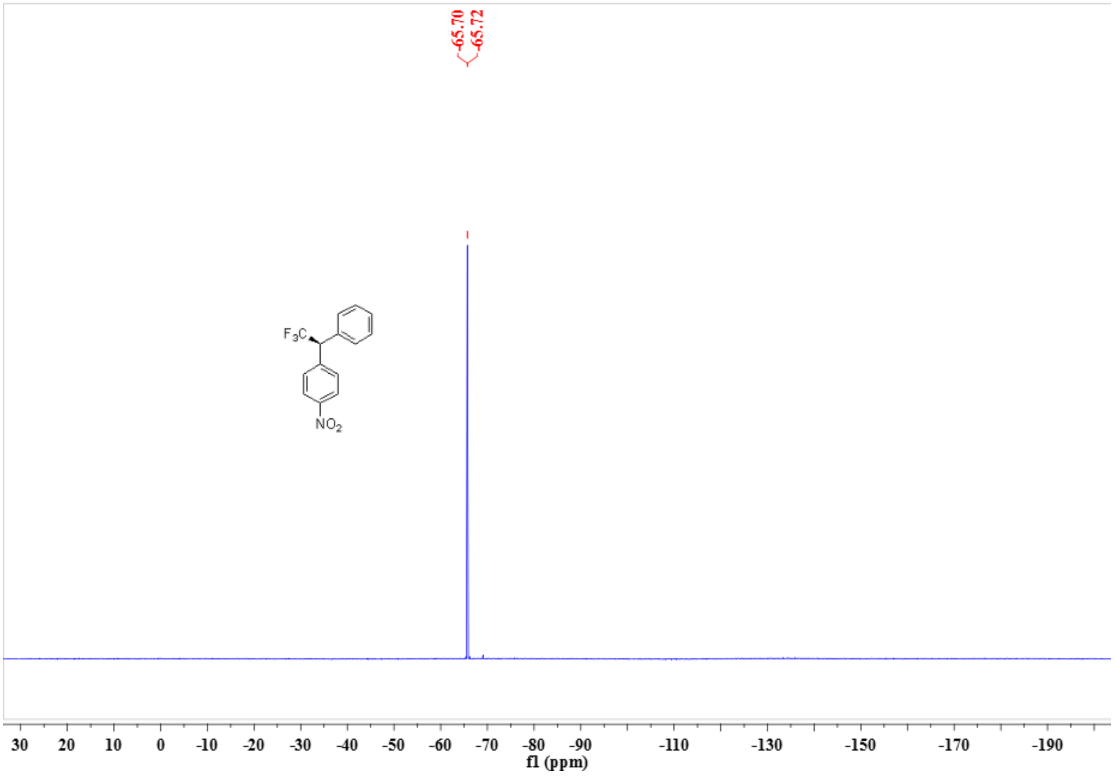


**Supplementary Figure 248.** $^{19}$**F NMR (376 MHz, CDCl$_3$) spectrum of 3k**

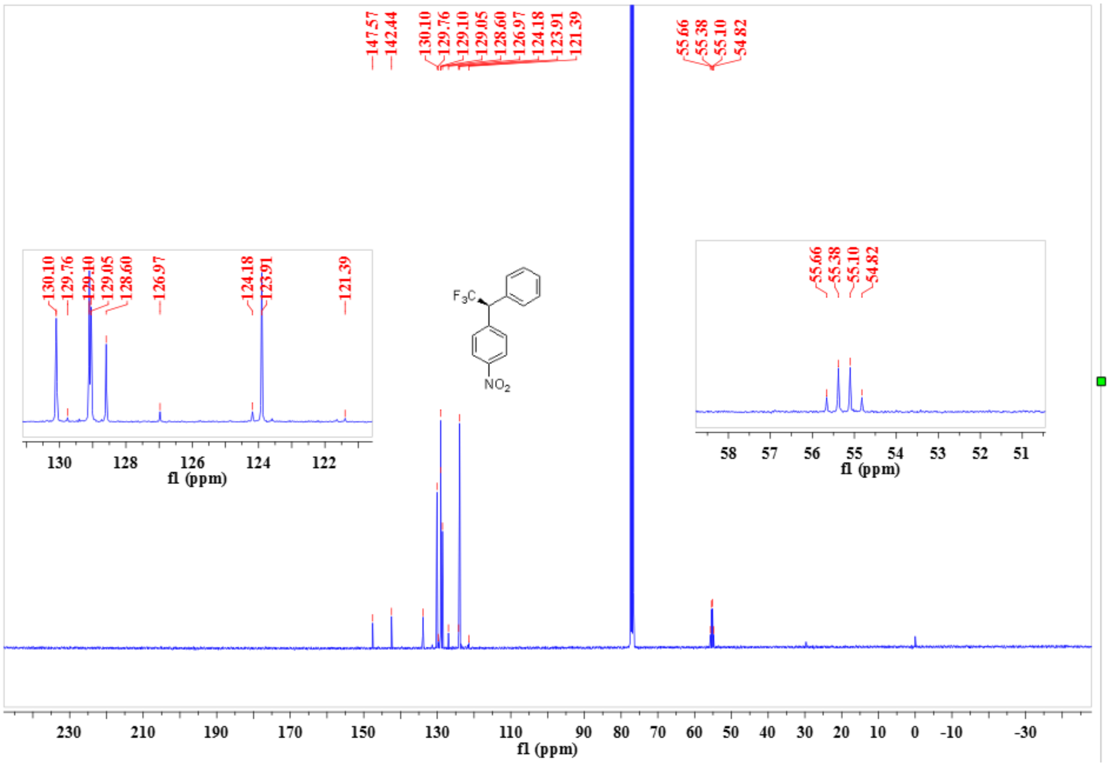


Supplementary Figure 249. $^{13}$C NMR (101 MHz, $CDCl_3$) spectrum of 3k

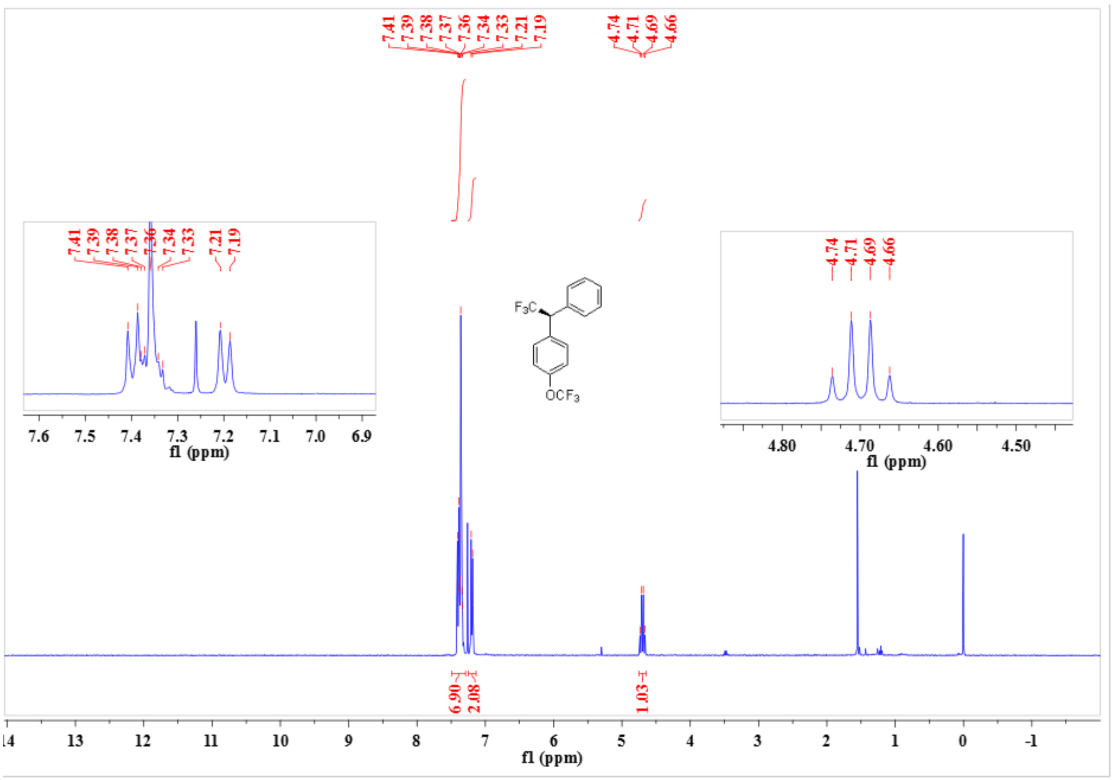


Supplementary Figure 250. $^{1}$H NMR (400 MHz, $CDCl_3$) spectrum of 3l

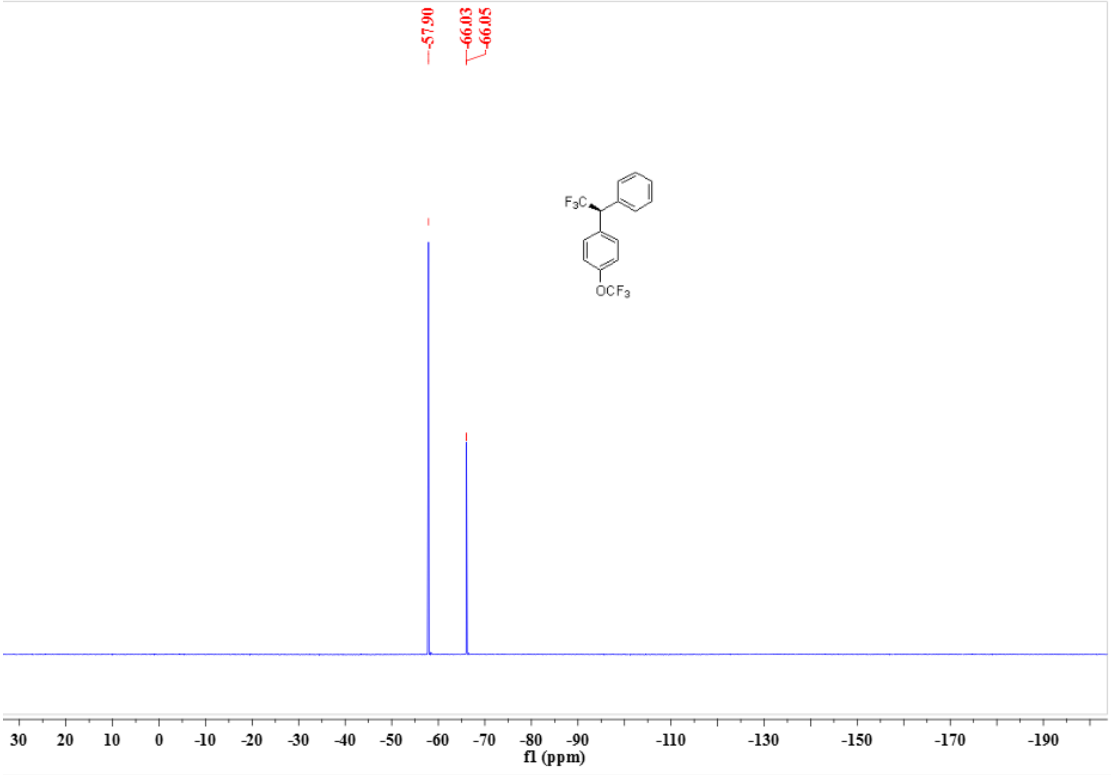


**Supplementary Figure 251.** $^{19}F$ **NMR (376 MHz,** $CDCl_3$**) spectrum of 3l**

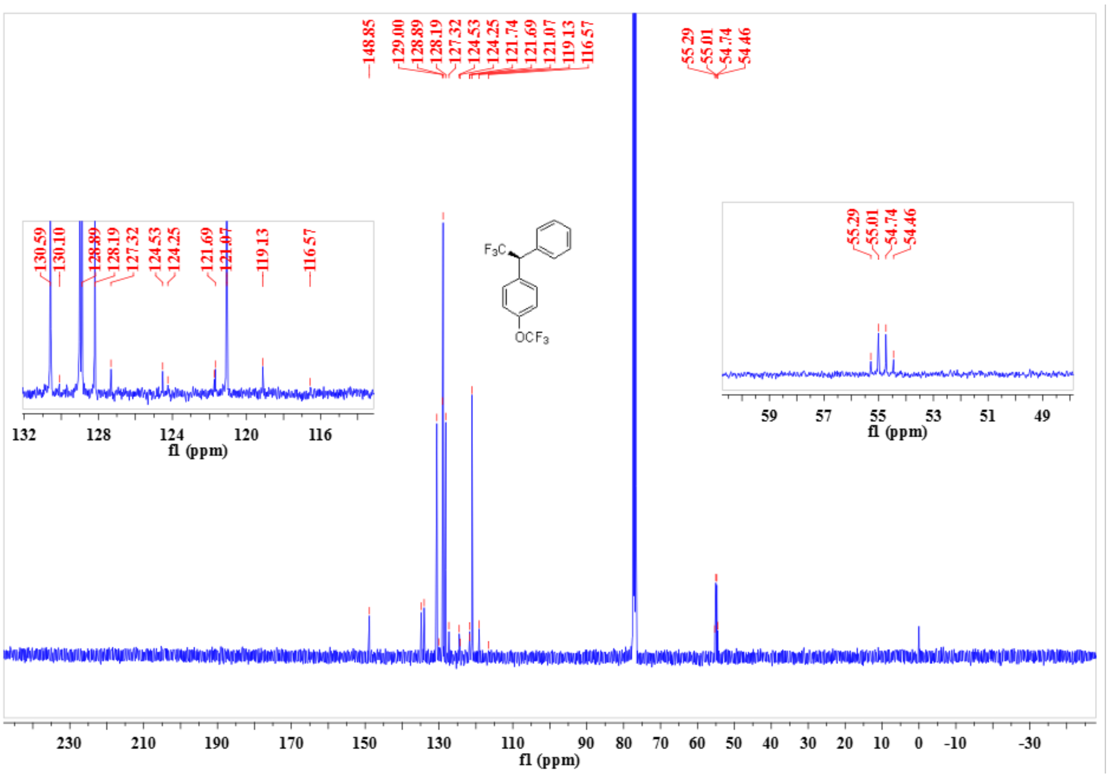


**Supplementary Figure 252.** $^{13}C$ **NMR (101 MHz,** $CDCl_3$**) spectrum of 3l**

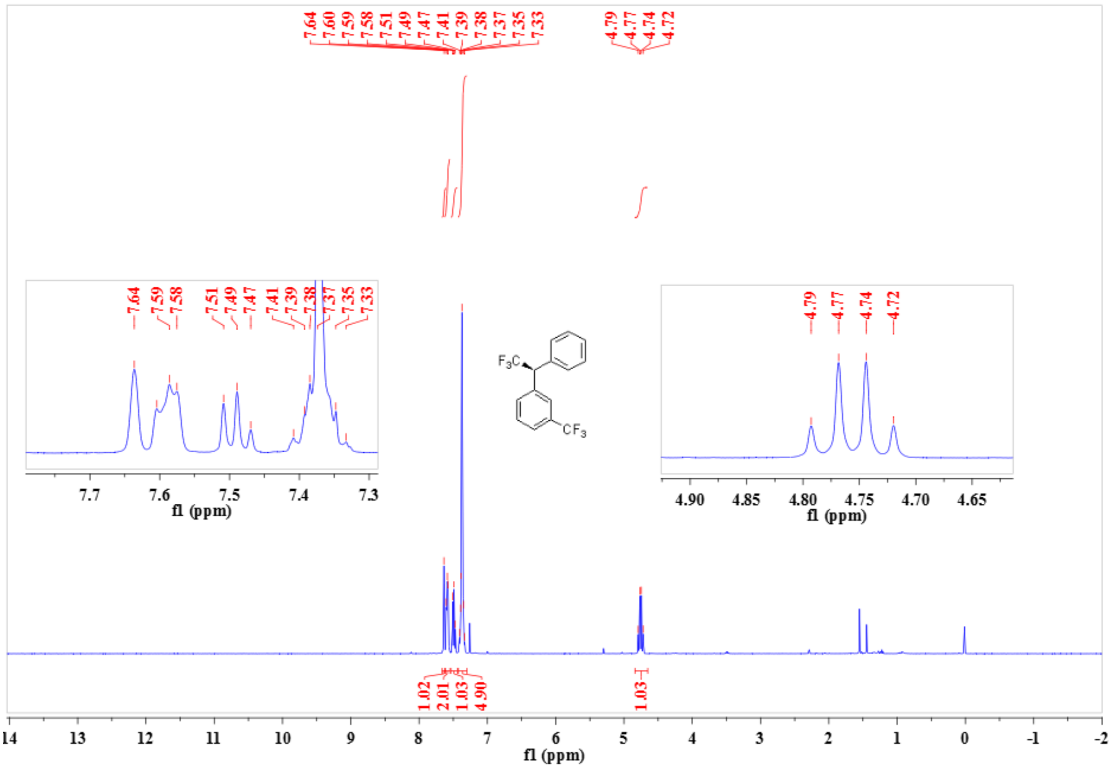

**Supplementary Figure 253.** $^{1}H$ **NMR (400 MHz,** $CDCl_3$**) spectrum of 3m**

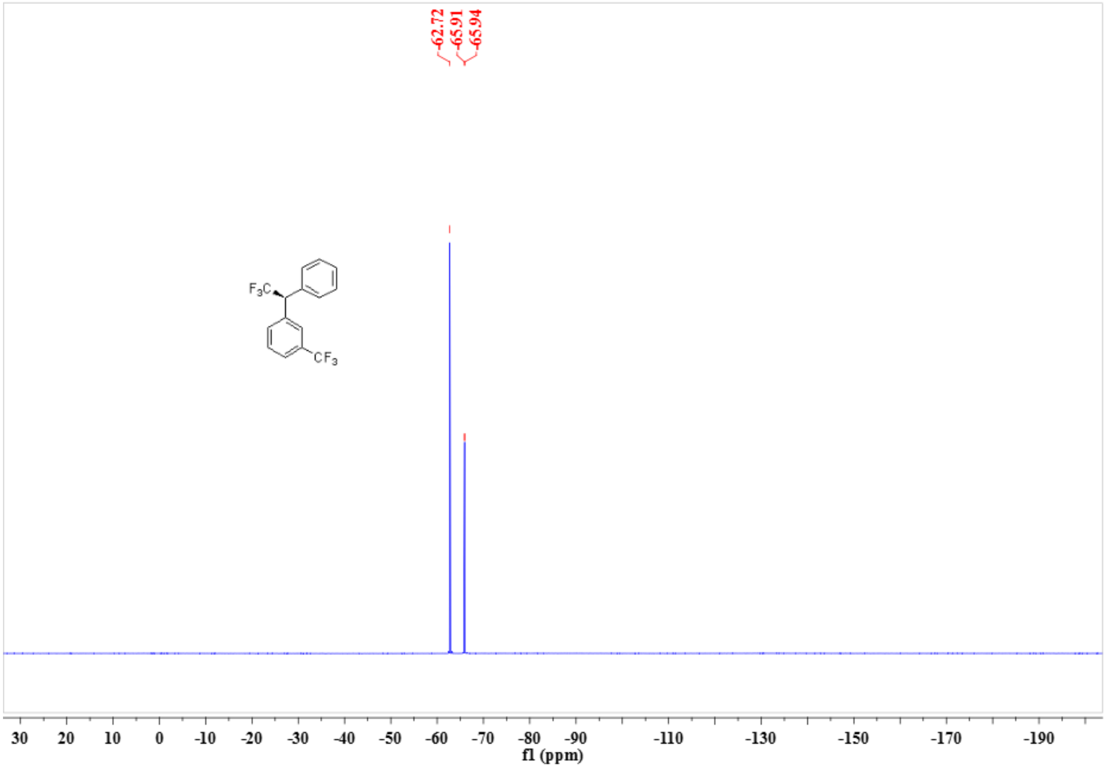

**Supplementary Figure 254.** $^{19}F$ **NMR (376 MHz,** $CDCl_3$**) spectrum of 3m**

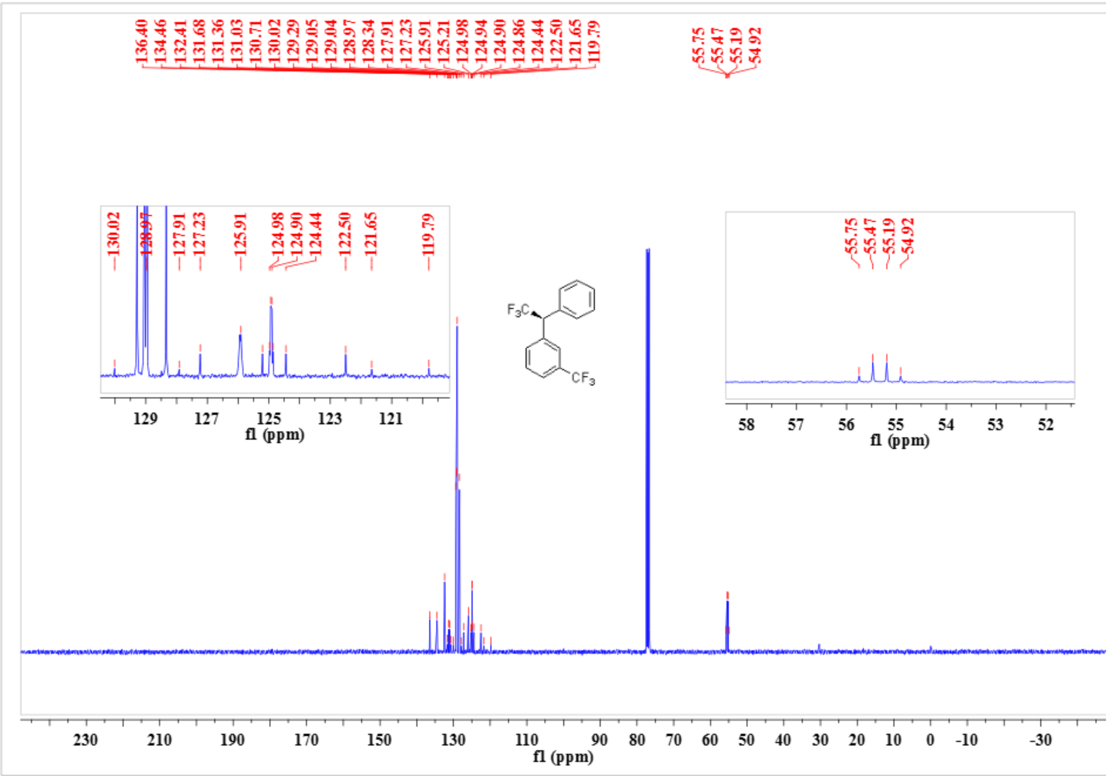


Supplementary Figure 255. $^{13}$C NMR (101 MHz, $CDCl_3$) spectrum of 3m

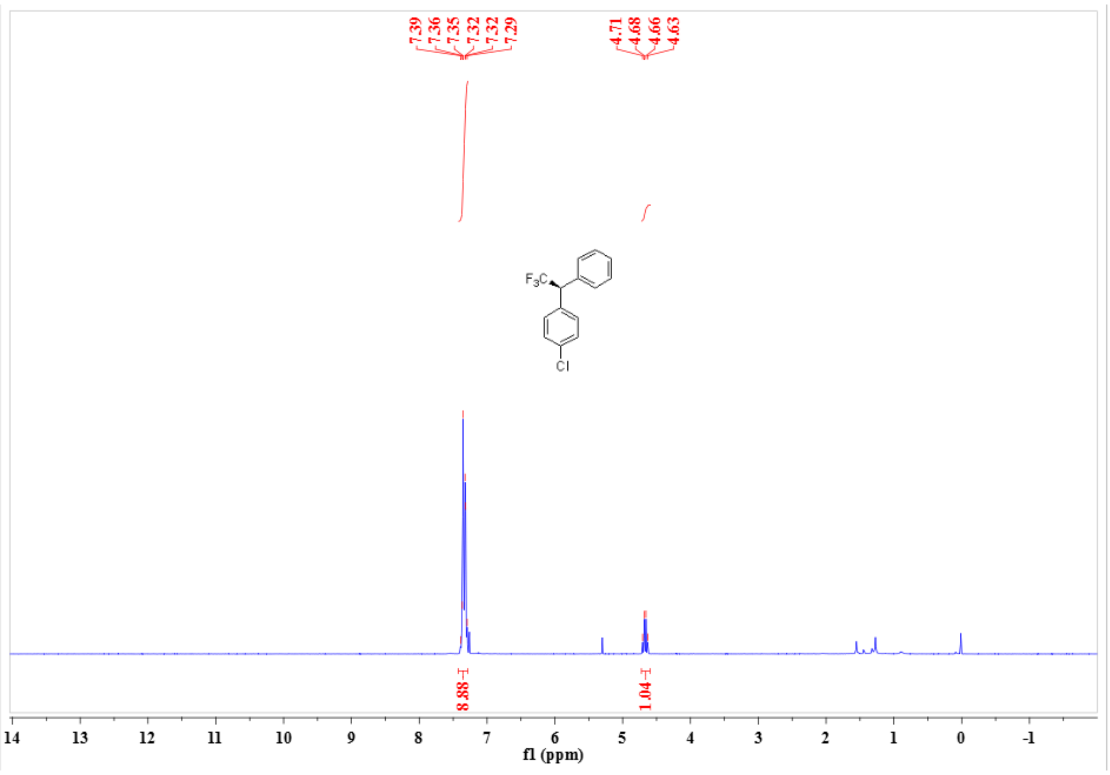


Supplementary Figure 256. $^{1}$H NMR (400 MHz, $CDCl_3$) spectrum of 3n

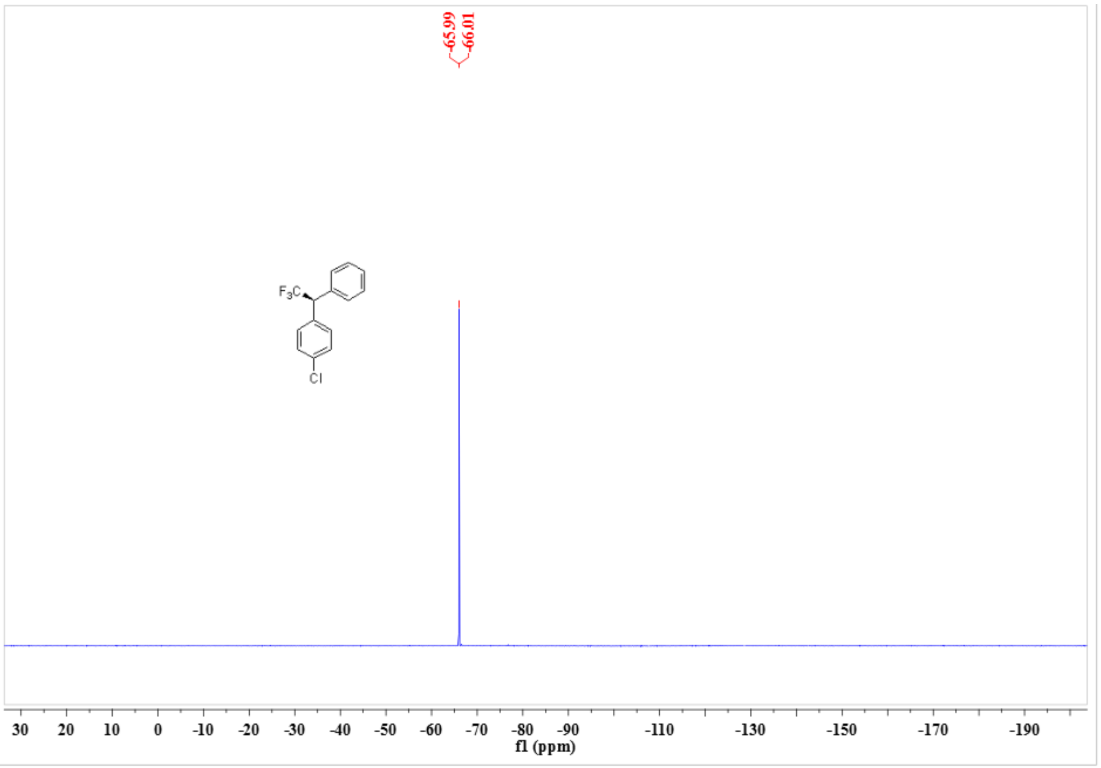

Supplementary Figure 257. $^{19}$F NMR (376 MHz, $CDCl_3$) spectrum of 3n

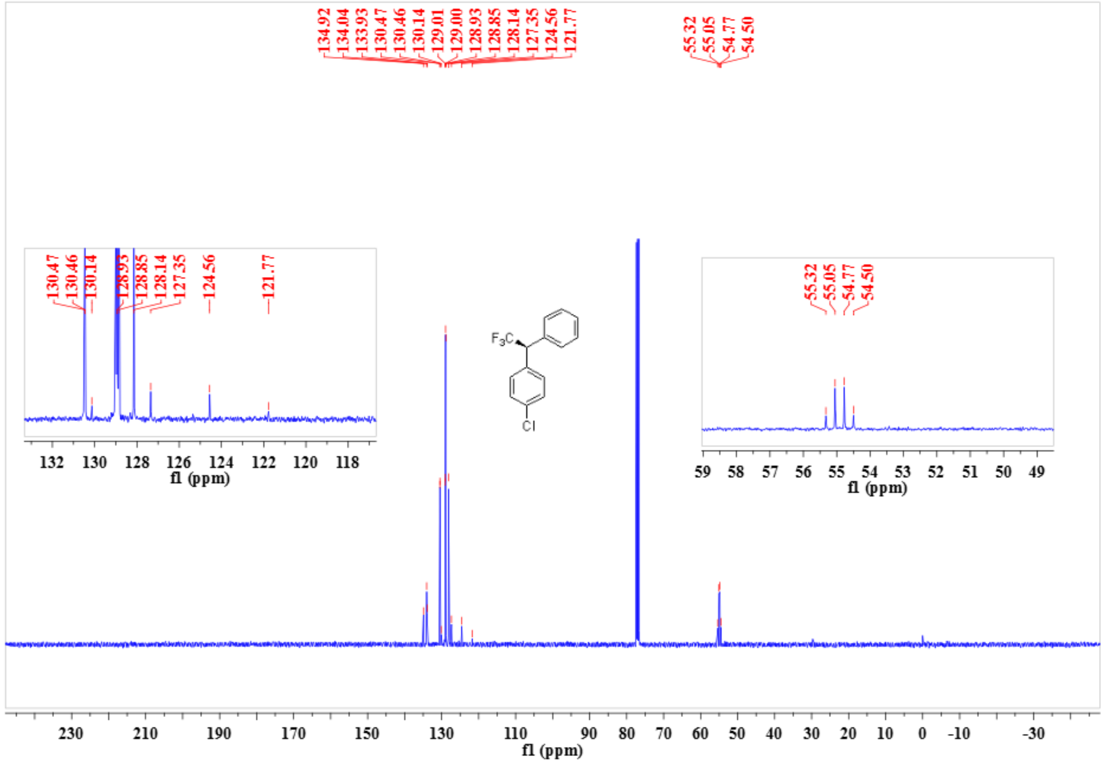

Supplementary Figure 258. $^{13}$C NMR (101 MHz, $CDCl_3$) spectrum of 3n

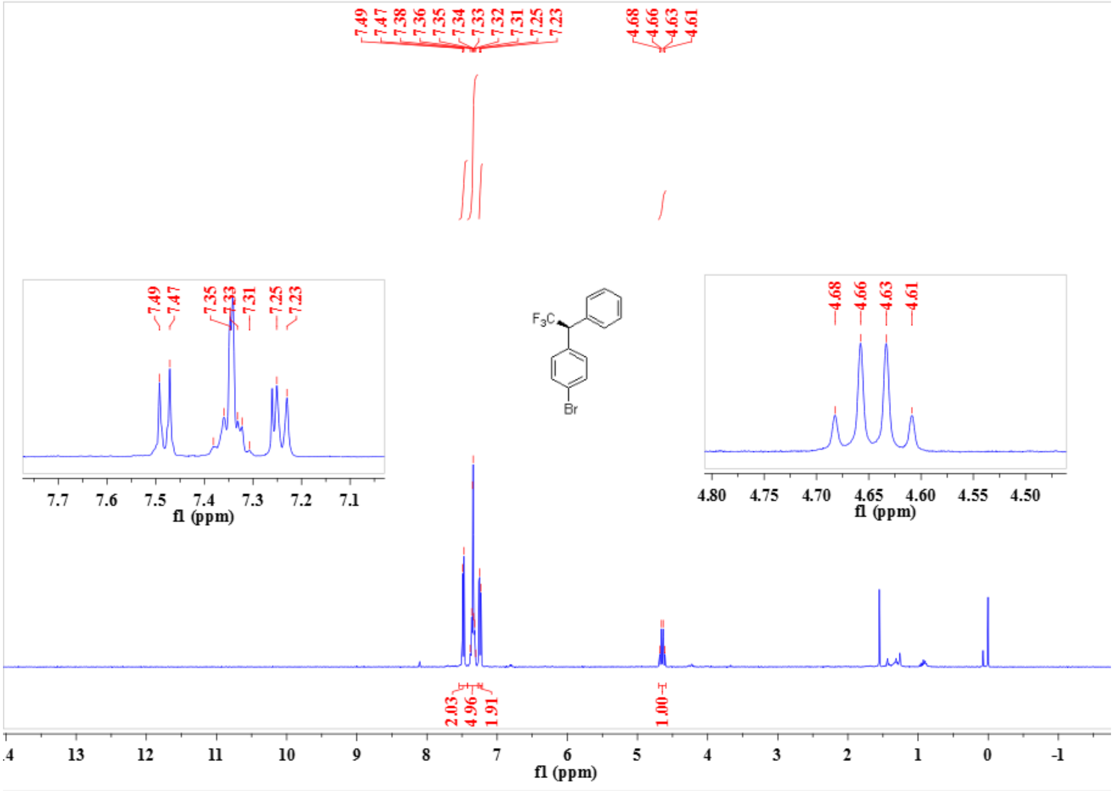


**Supplementary Figure 259.** $^{1}$**H NMR (400 MHz, $CDCl_3$) spectrum of 3o**

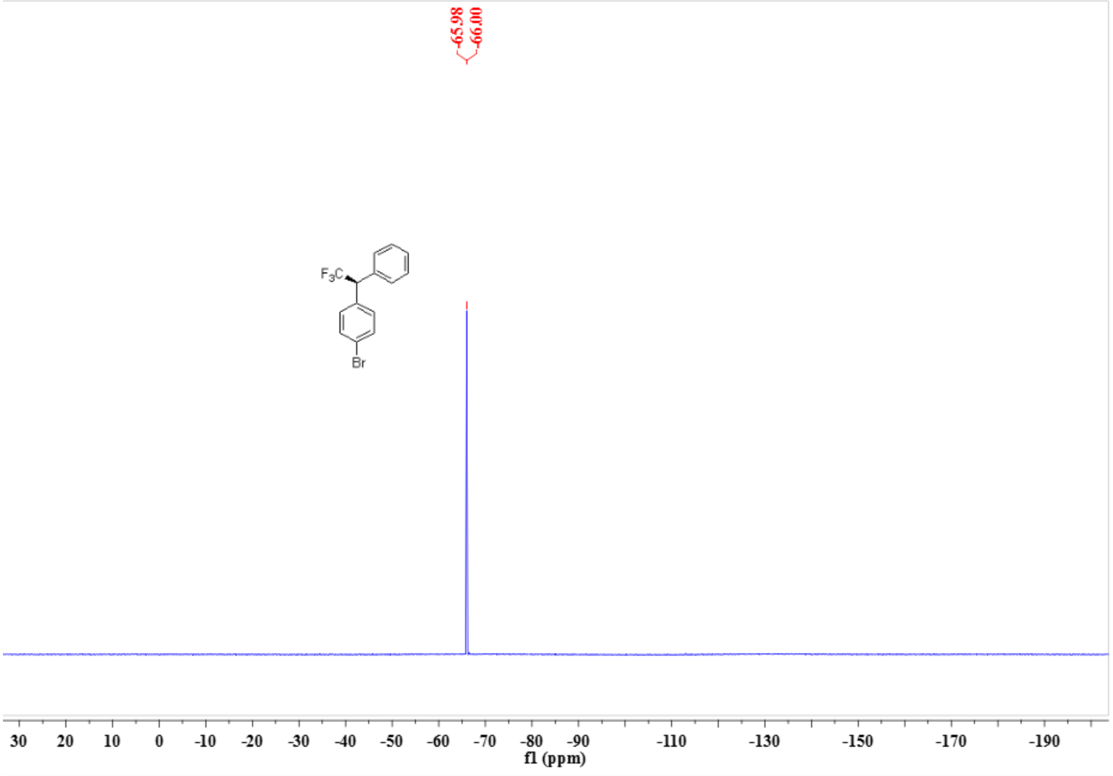


**Supplementary Figure 260.** $^{19}$**F NMR (376 MHz, $CDCl_3$) spectrum of 3o**

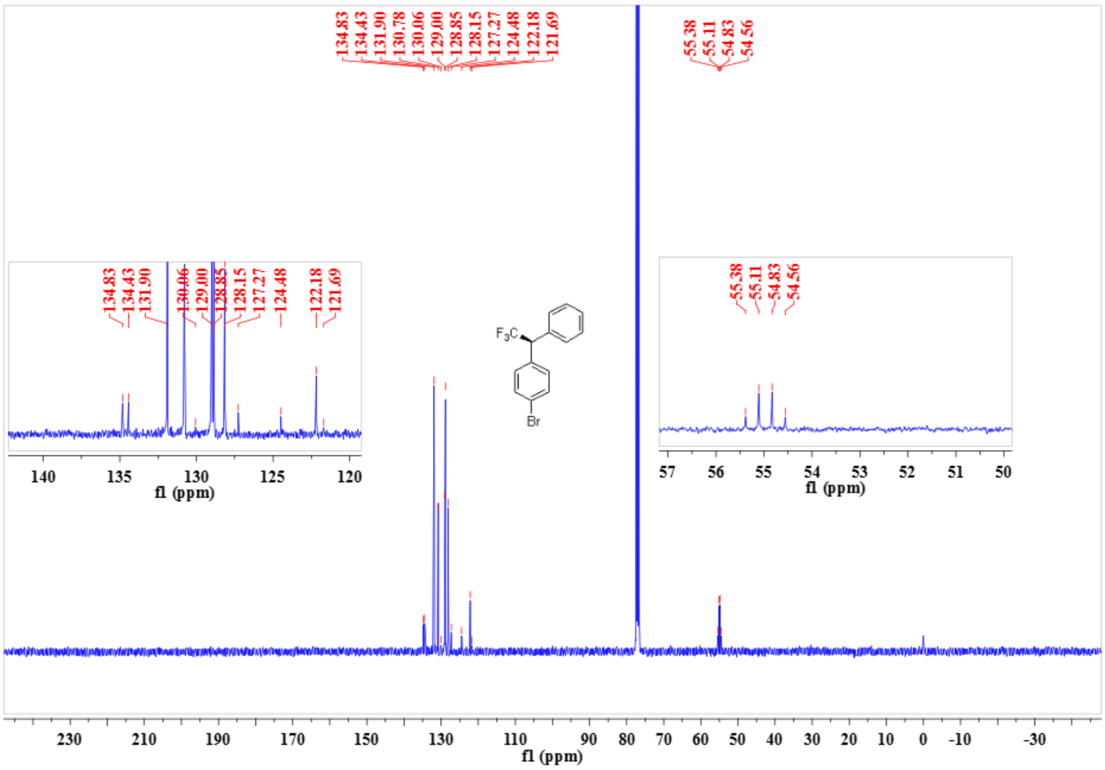


**Supplementary Figure 261.** $^{13}C$ **NMR (101 MHz, CDCl₃) spectrum of 3o**

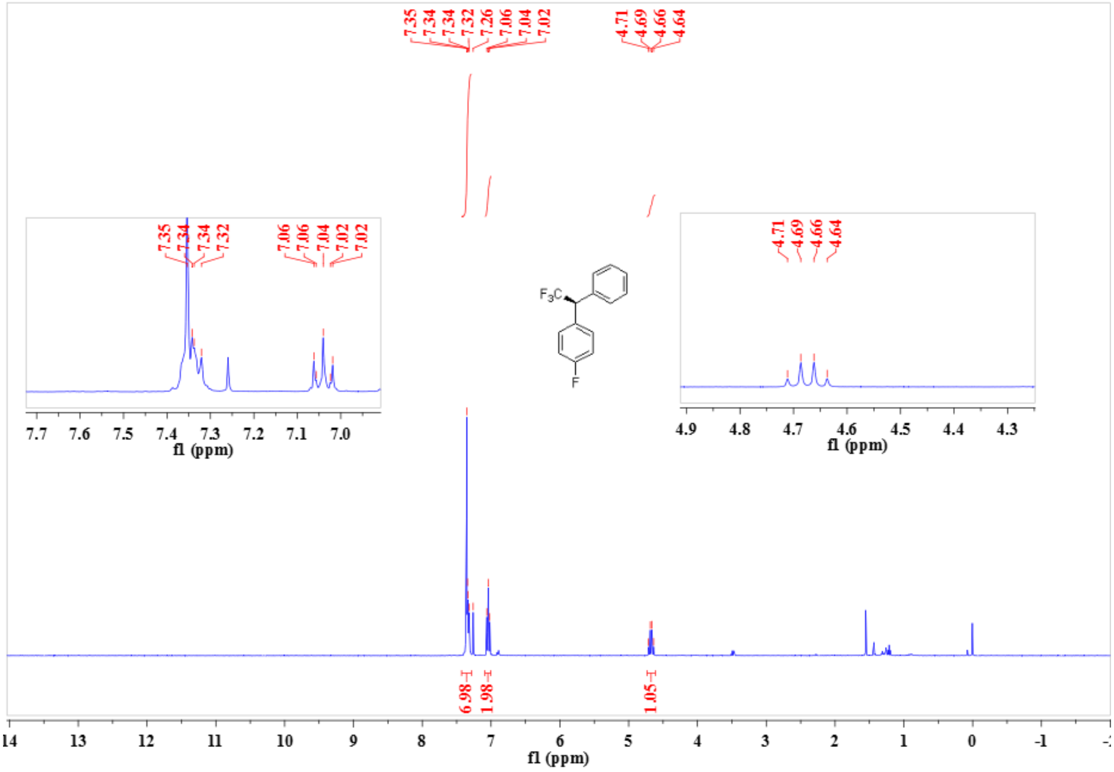


**Supplementary Figure 262.** $^{1}H$ **NMR (400 MHz, CDCl₃) spectrum of 3p**

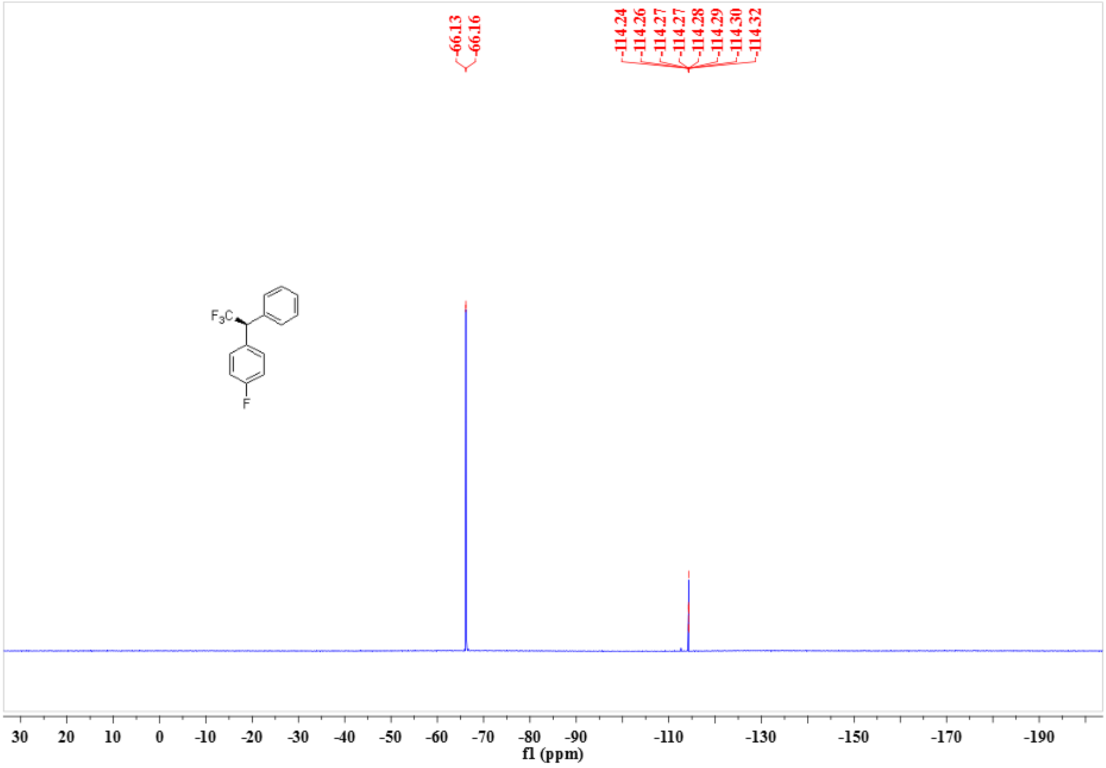


**Supplementary Figure 263.** $^{19}$F NMR (376 MHz, $CDCl_3$) spectrum of 3p

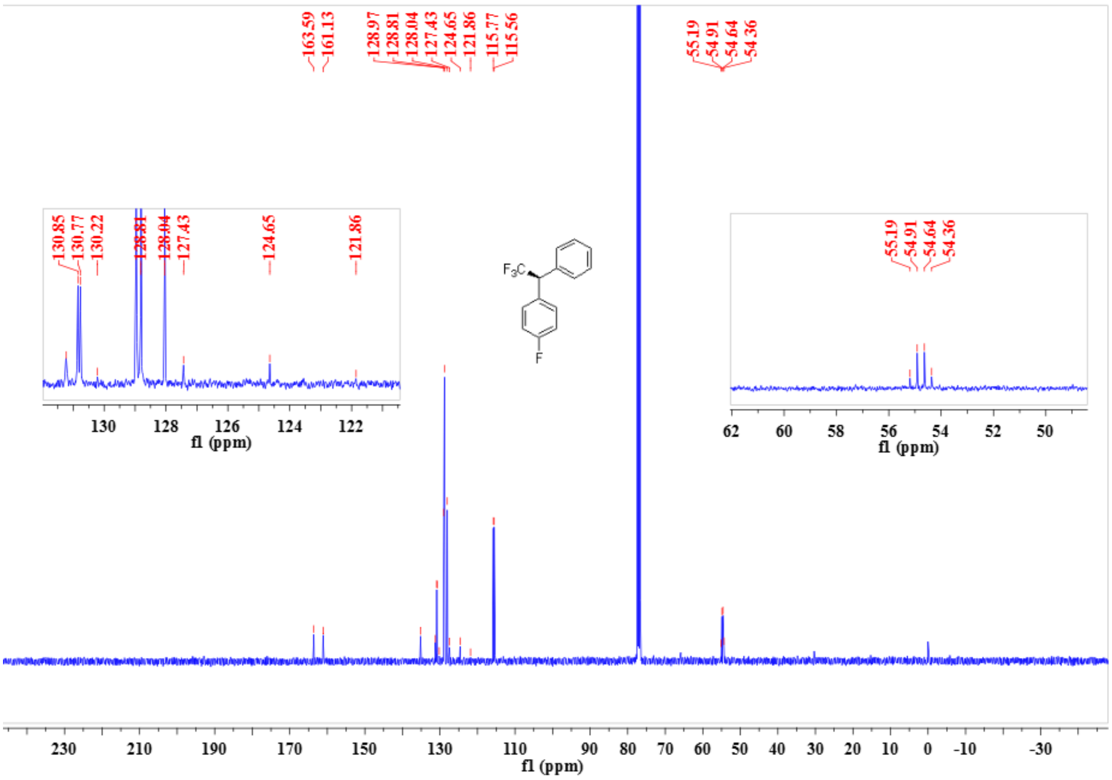


**Supplementary Figure 264.** $^{13}$C NMR (101 MHz, $CDCl_3$) spectrum of 3p

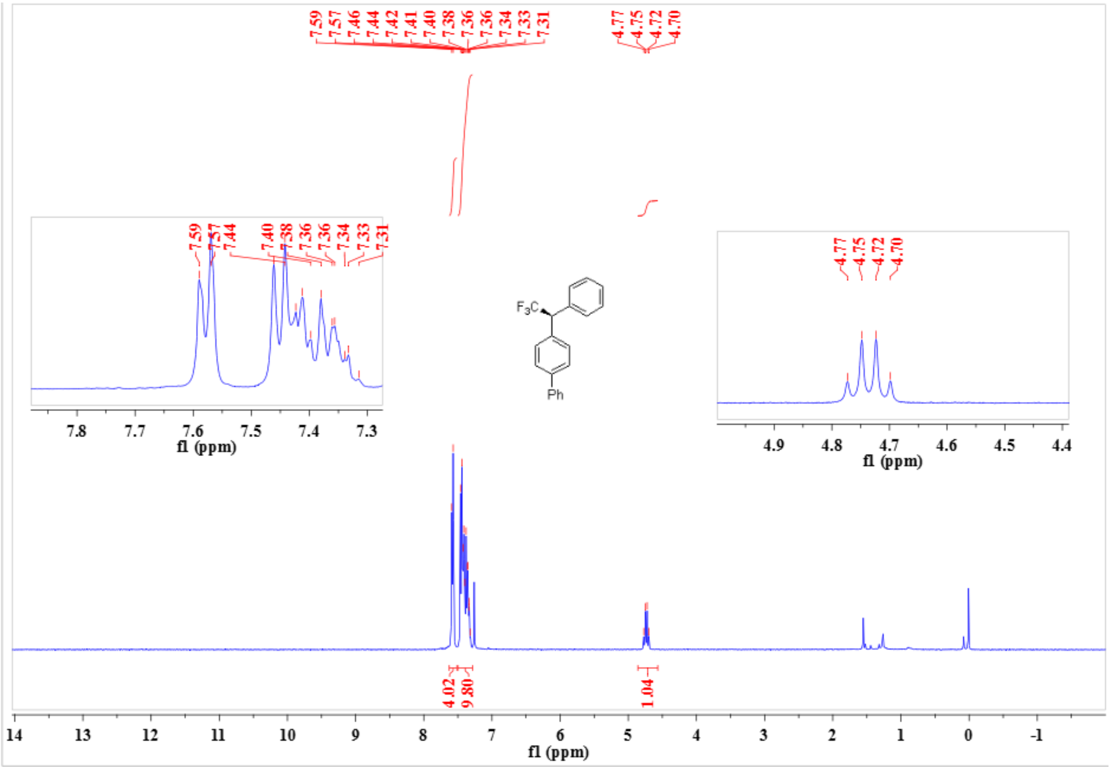

**Supplementary Figure 265.** **$^{1}H$ NMR (400 MHz, $CDCl_3$) spectrum of 3q**

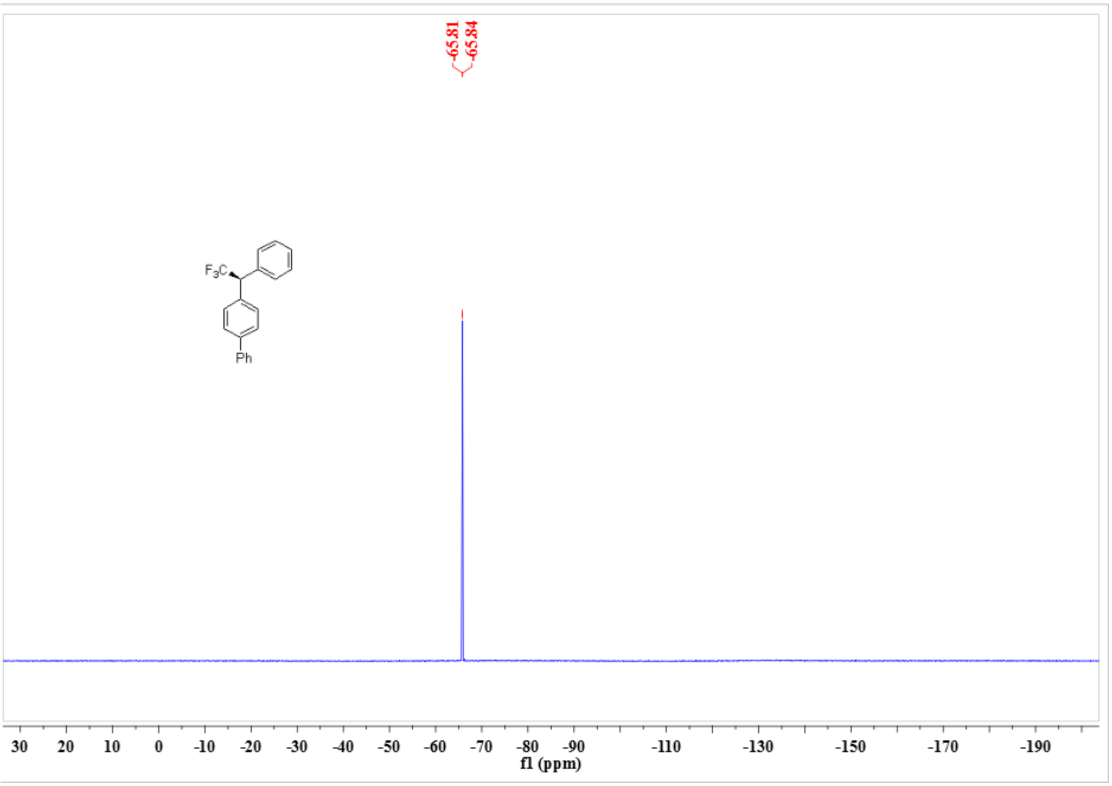

**Supplementary Figure 266.** **$^{19}F$ NMR (376 MHz, $CDCl_3$) spectrum of 3q**

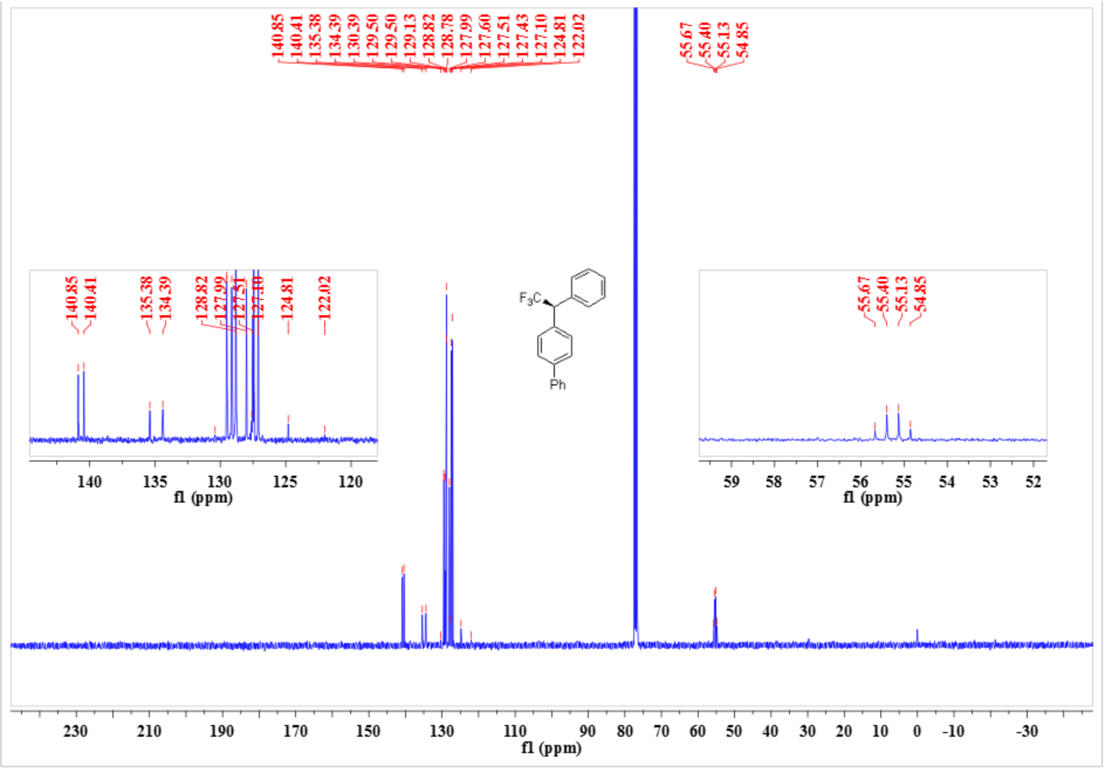

**Supplementary Figure 267.** $^{13}C$ **NMR (101 MHz,** $CDCl_3$**) spectrum of 3q**

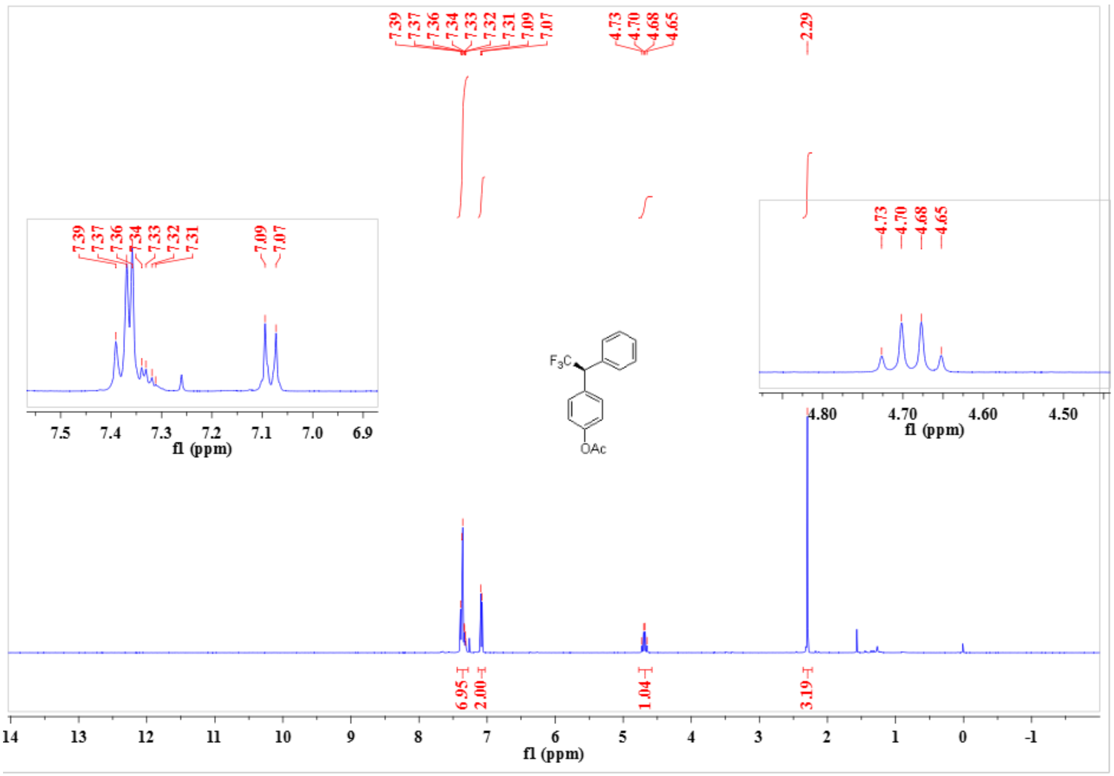

**Supplementary Figure 268.** $^{1}H$ **NMR (400 MHz,** $CDCl_3$**) spectrum of 3r**

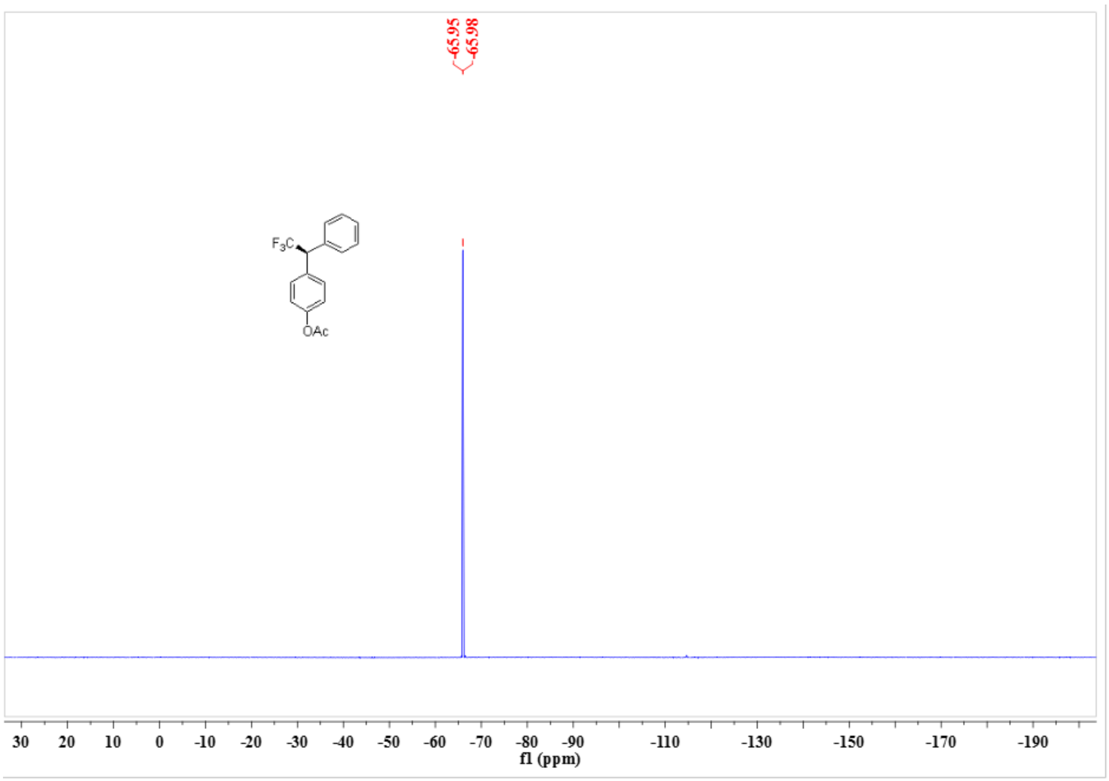


**Supplementary Figure 269.** $^{19}$**F NMR (376 MHz, CDCl$_3$) spectrum of 3r**

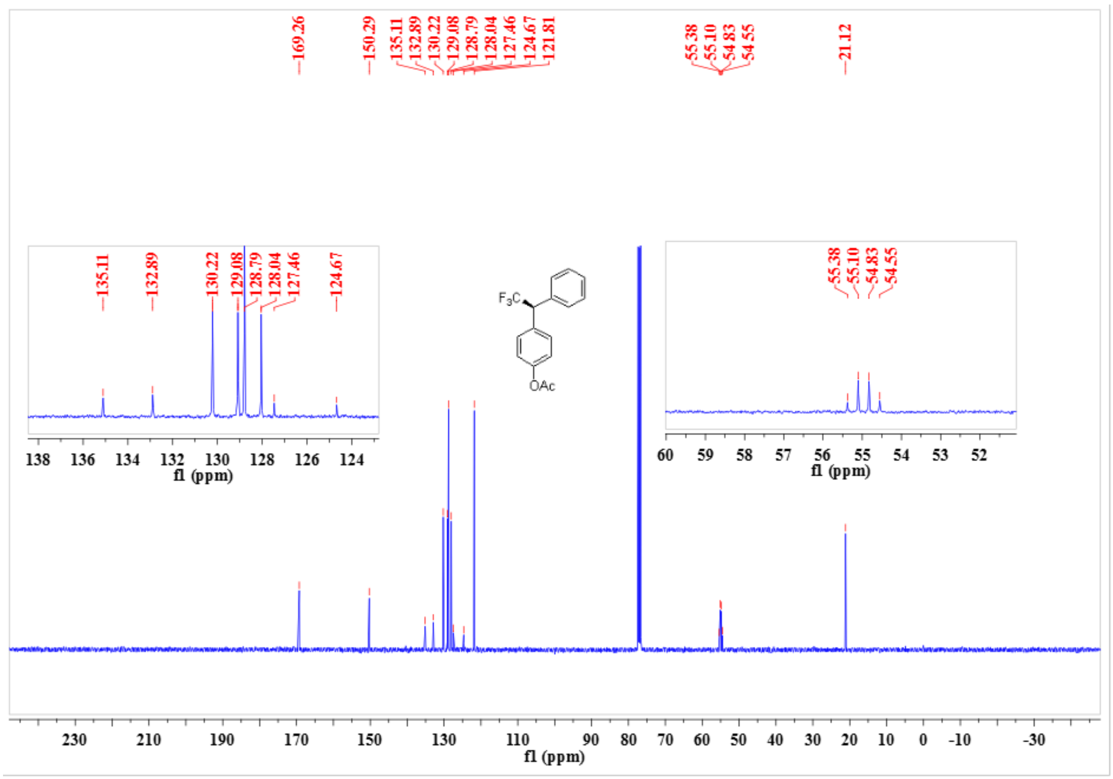


**Supplementary Figure 270.** $^{13}$**C NMR (101 MHz, CDCl$_3$) spectrum of 3r**

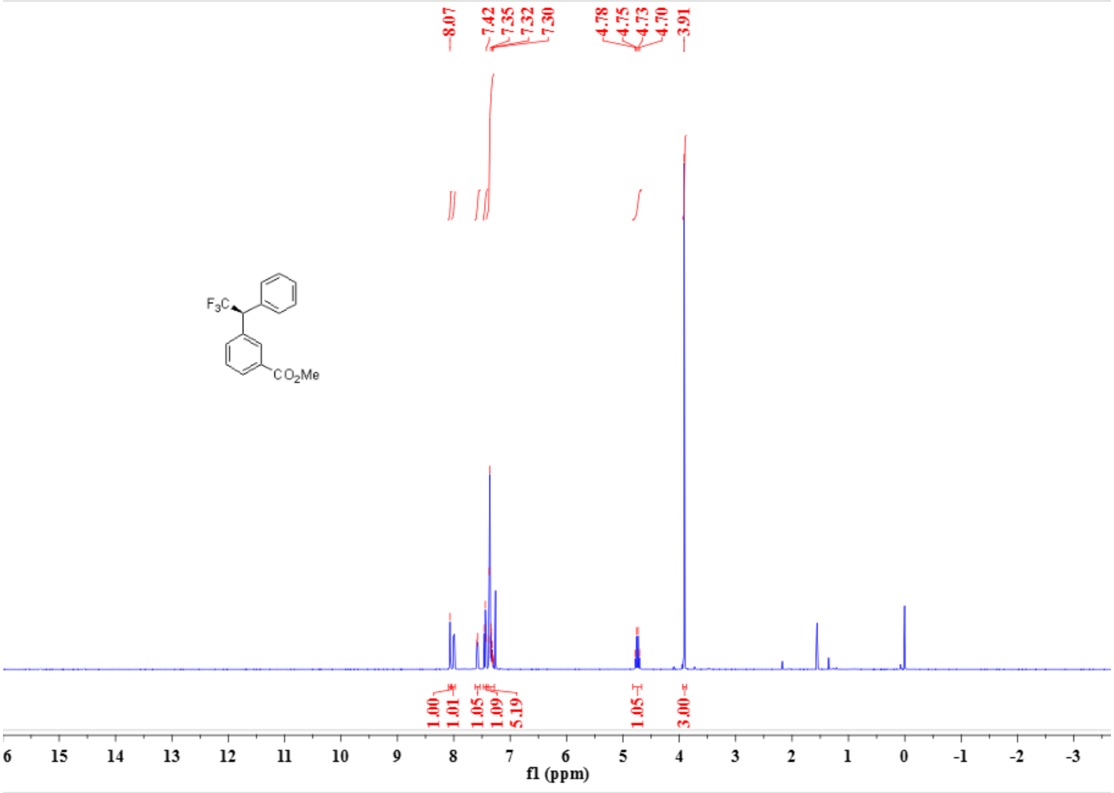


**Supplementary Figure 271.** $^{1}$**H NMR (400 MHz, CDCl**$_3$**) spectrum of 3s**

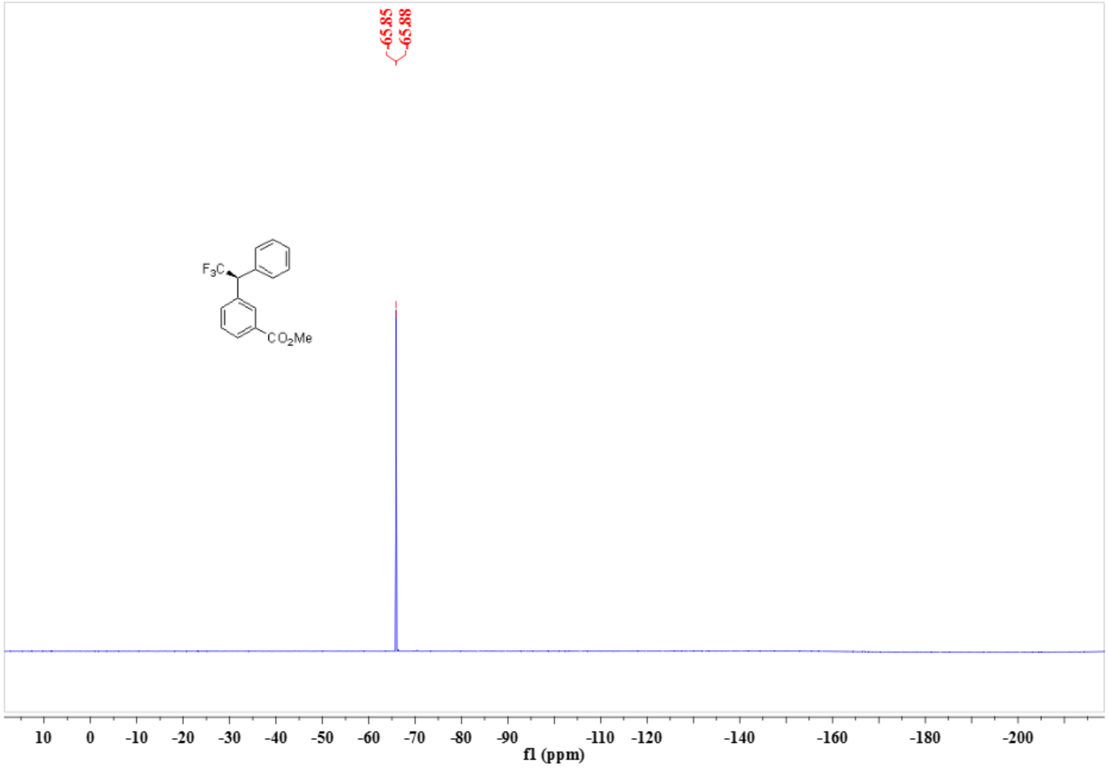


**Supplementary Figure 272.** $^{19}$**F NMR (376 MHz, CDCl**$_3$**) spectrum of 3s**

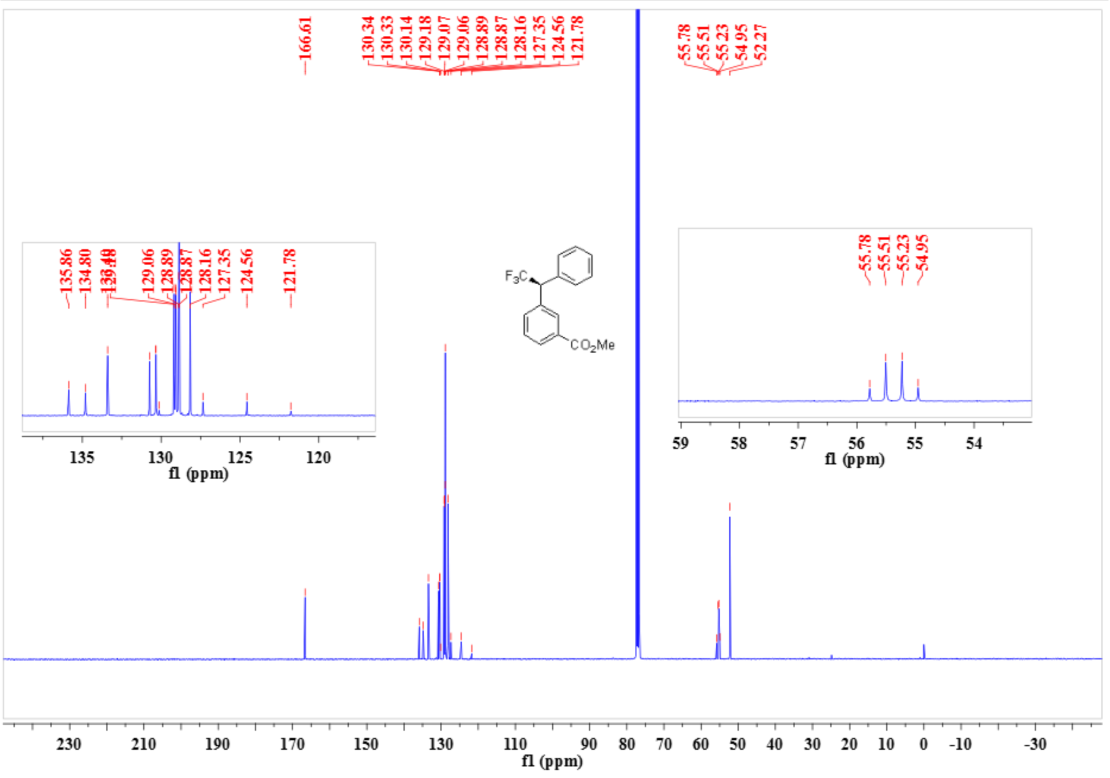


**Supplementary Figure 273.** $^{13}$**C NMR (101 MHz, CDCl$_3$) spectrum of 3s**

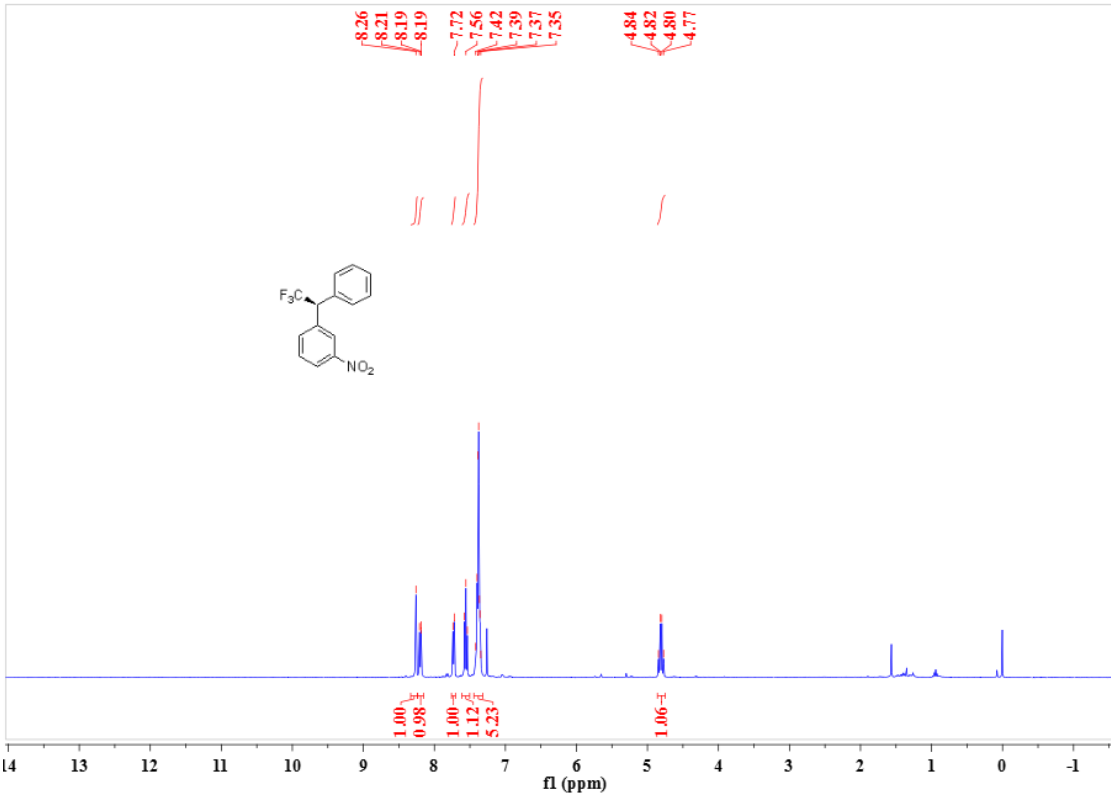


**Supplementary Figure 274.** $^{1}$**H NMR (400 MHz, CDCl$_3$) spectrum of 3t**

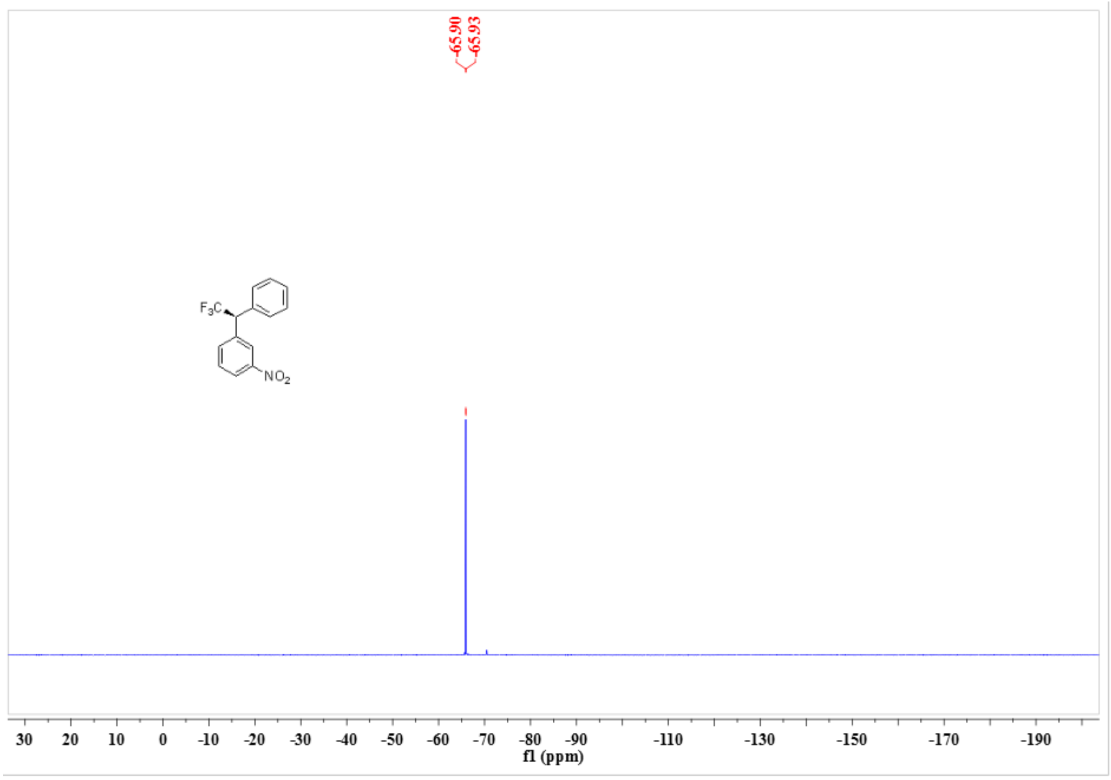


**Supplementary Figure 275.** $^{19}F$ **NMR (376 MHz,** $CDCl_3$**) spectrum of 3t**

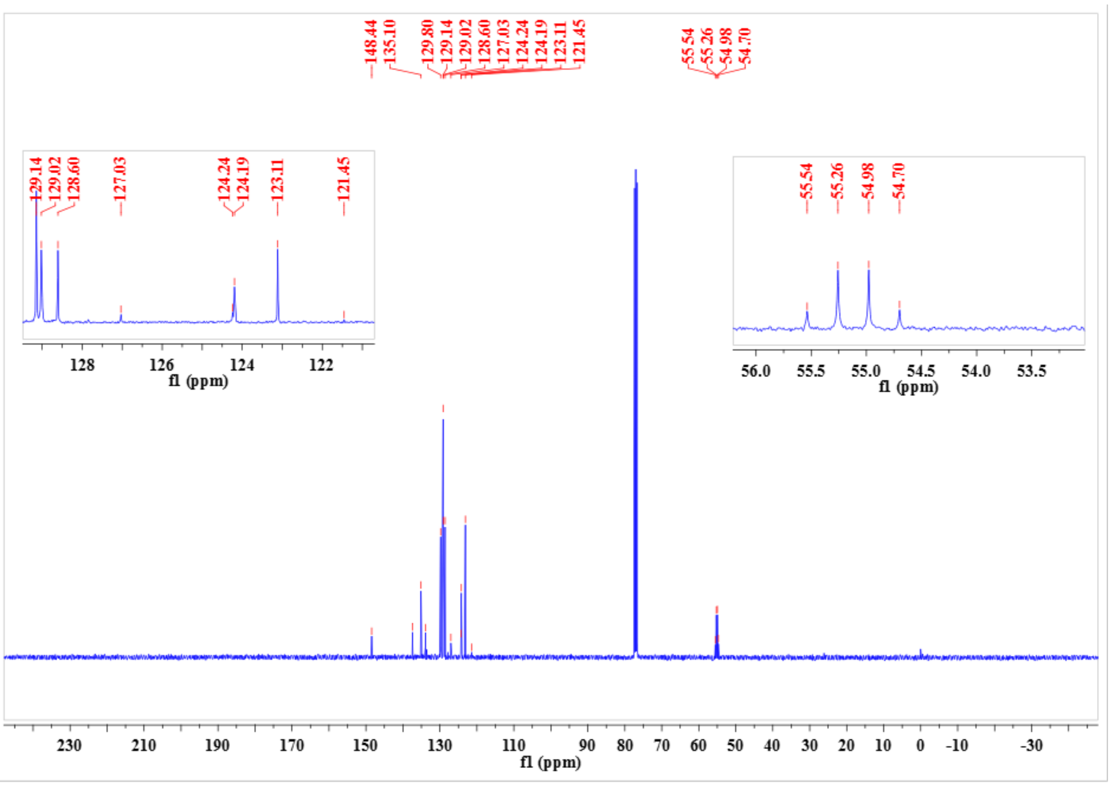


**Supplementary Figure 276.** $^{13}C$ **NMR (101 MHz,** $CDCl_3$**) spectrum of 3t**

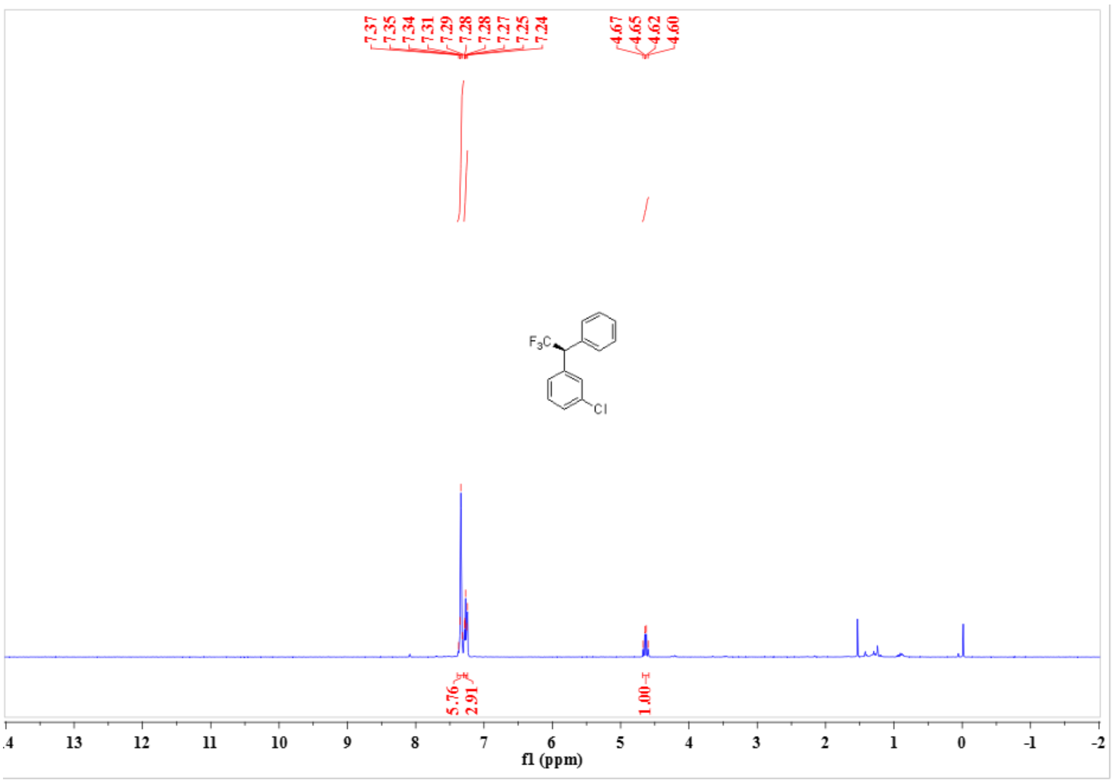


**Supplementary Figure 277.** $^{1}$**H NMR (400 MHz, $CDCl_3$) spectrum of 3u**

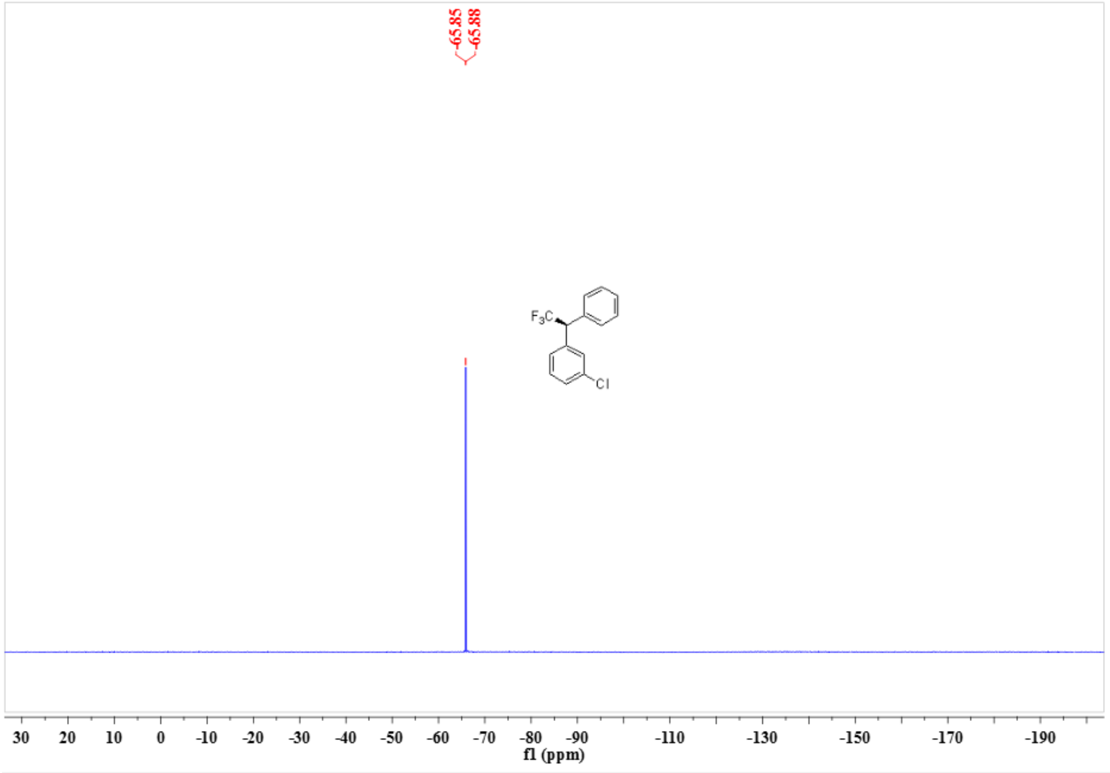


**Supplementary Figure 278.** $^{19}$**F NMR (376 MHz, $CDCl_3$) spectrum of 3u**

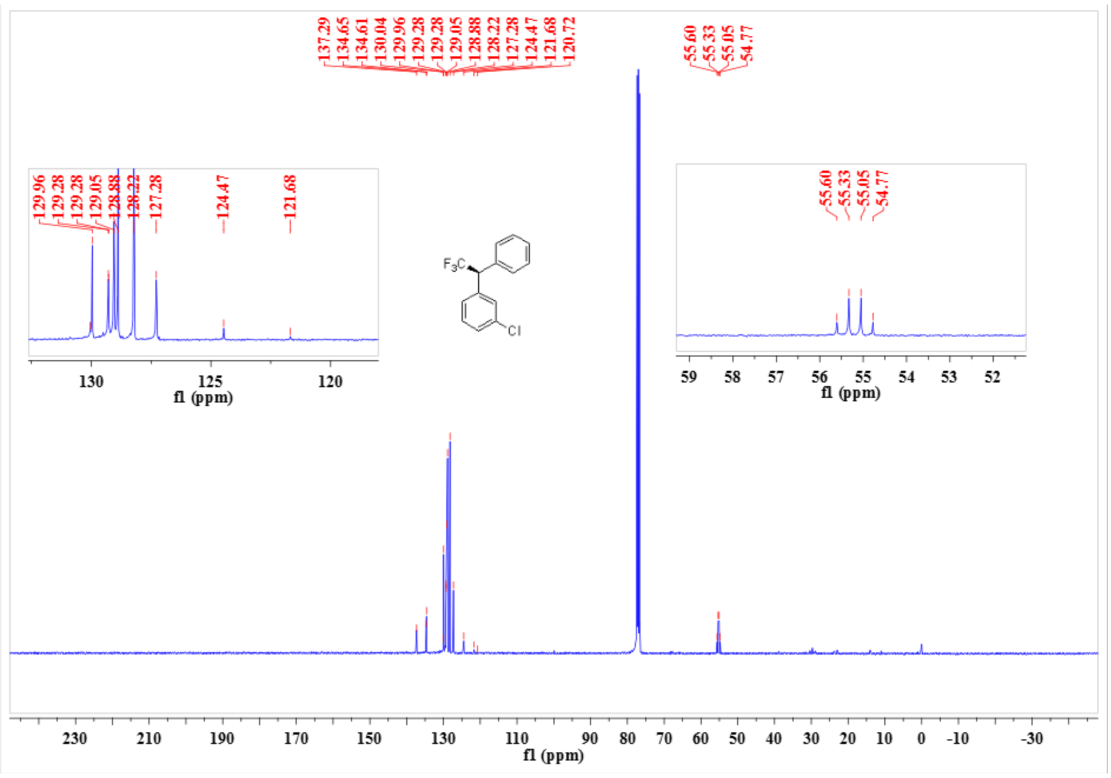


**Supplementary Figure 279.** $^{13}C$ **NMR (101 MHz,** $CDCl_3$**) spectrum of 3u**

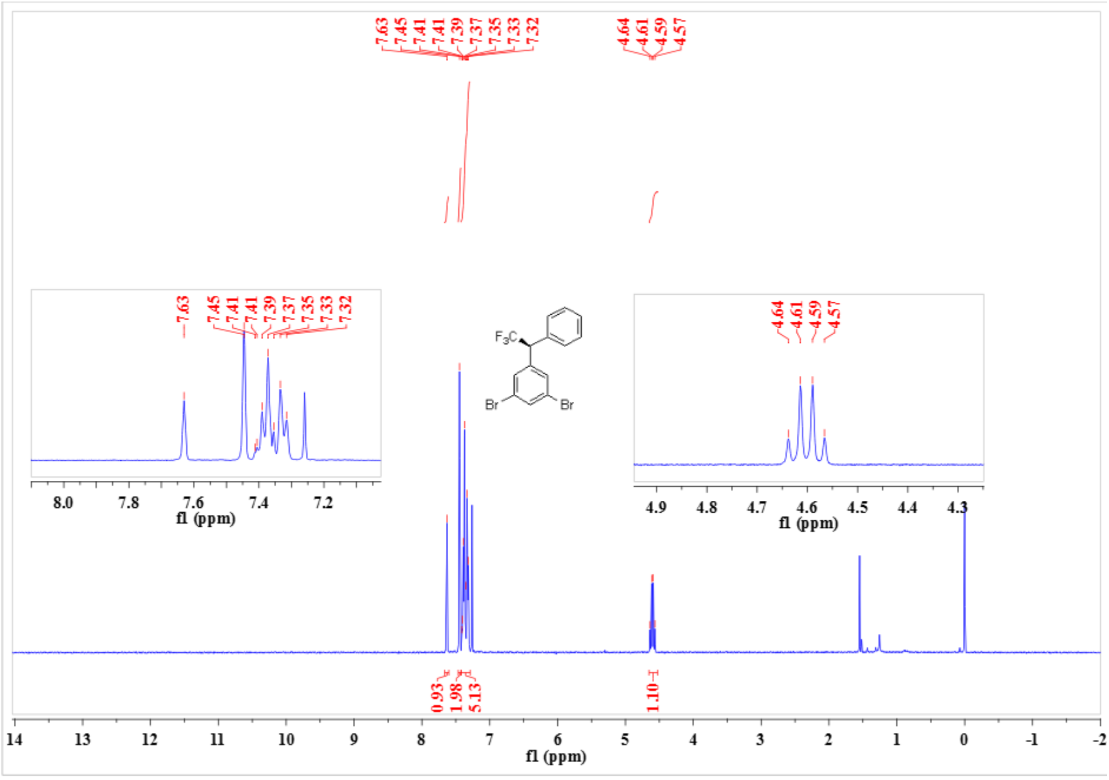


**Supplementary Figure 280.** $^{1}H$ **NMR (400 MHz,** $CDCl_3$**) spectrum of 3v**

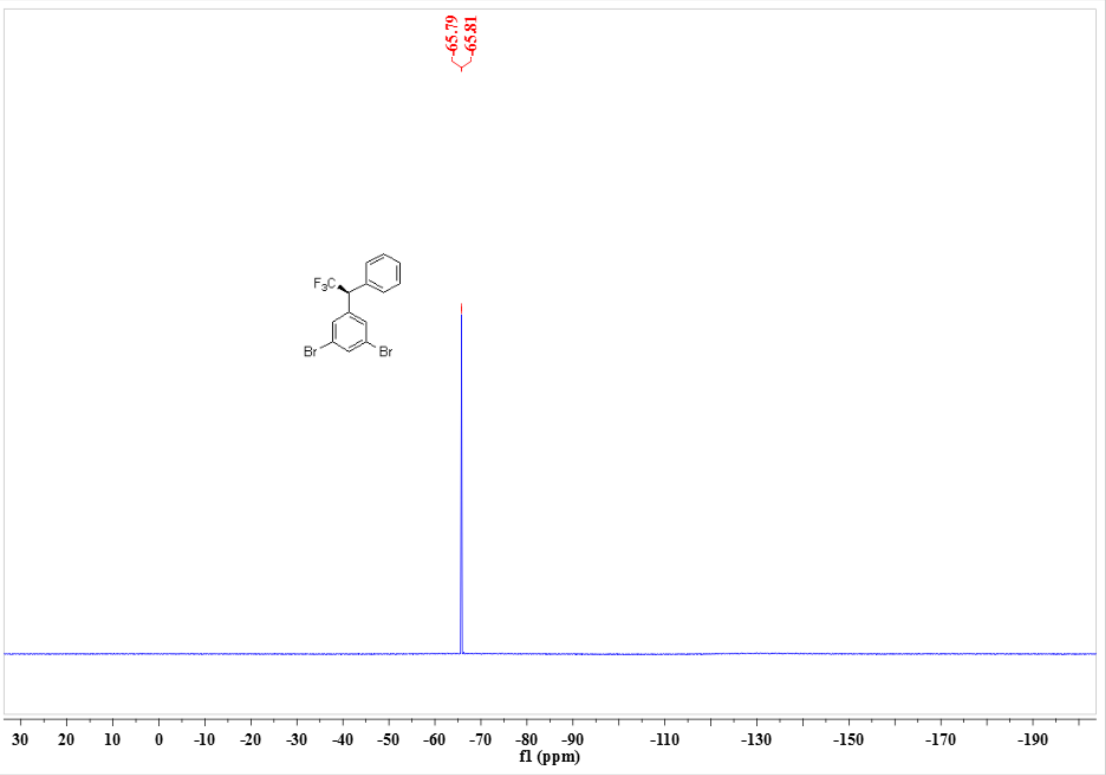



**Supplementary Figure 281.** $^{19}F$ **NMR (376 MHz,** $CDCl_3$**) spectrum of 3v**

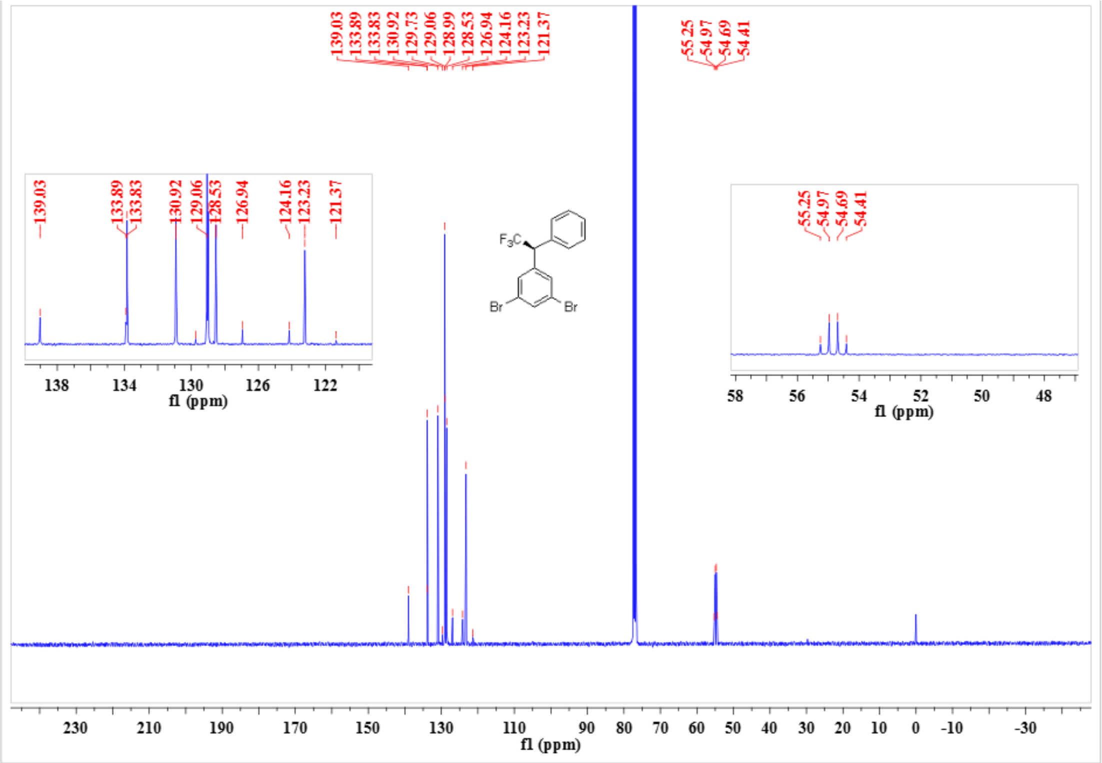



**Supplementary Figure 282.** $^{13}C$ **NMR (101 MHz,** $CDCl_3$**) spectrum of 3v**

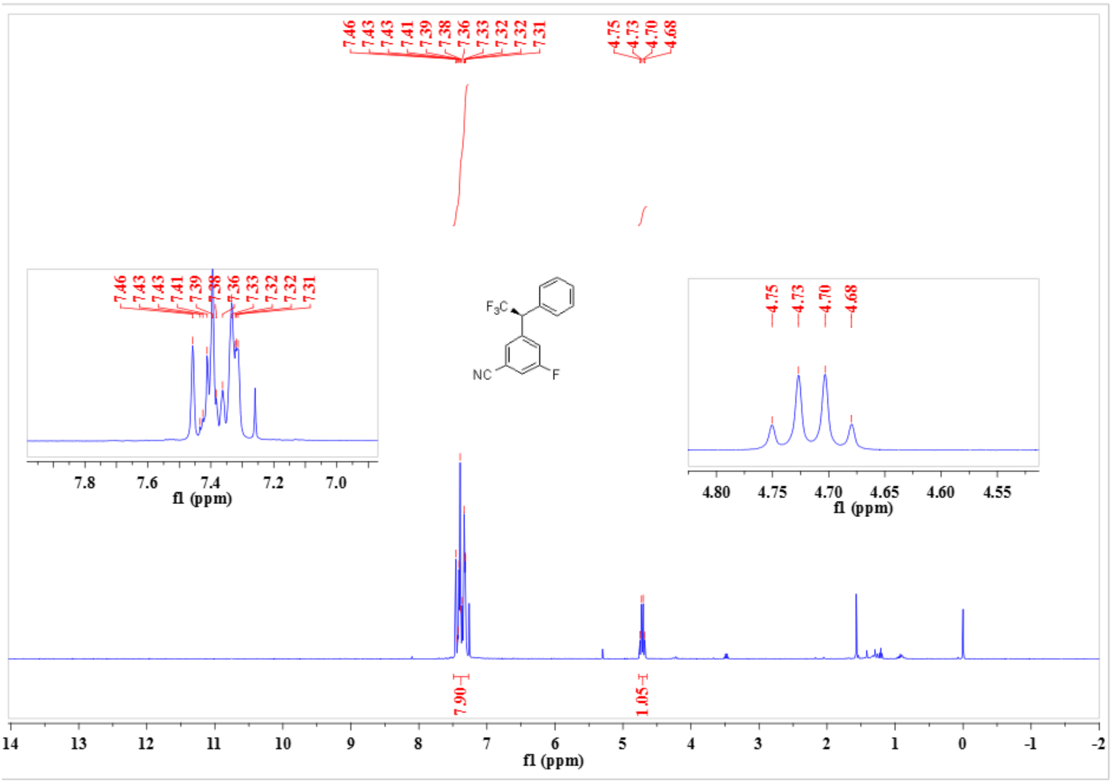

**Supplementary Figure 283.** **$^{1}$H NMR (400 MHz, CDCl$_3$) spectrum of 3w**

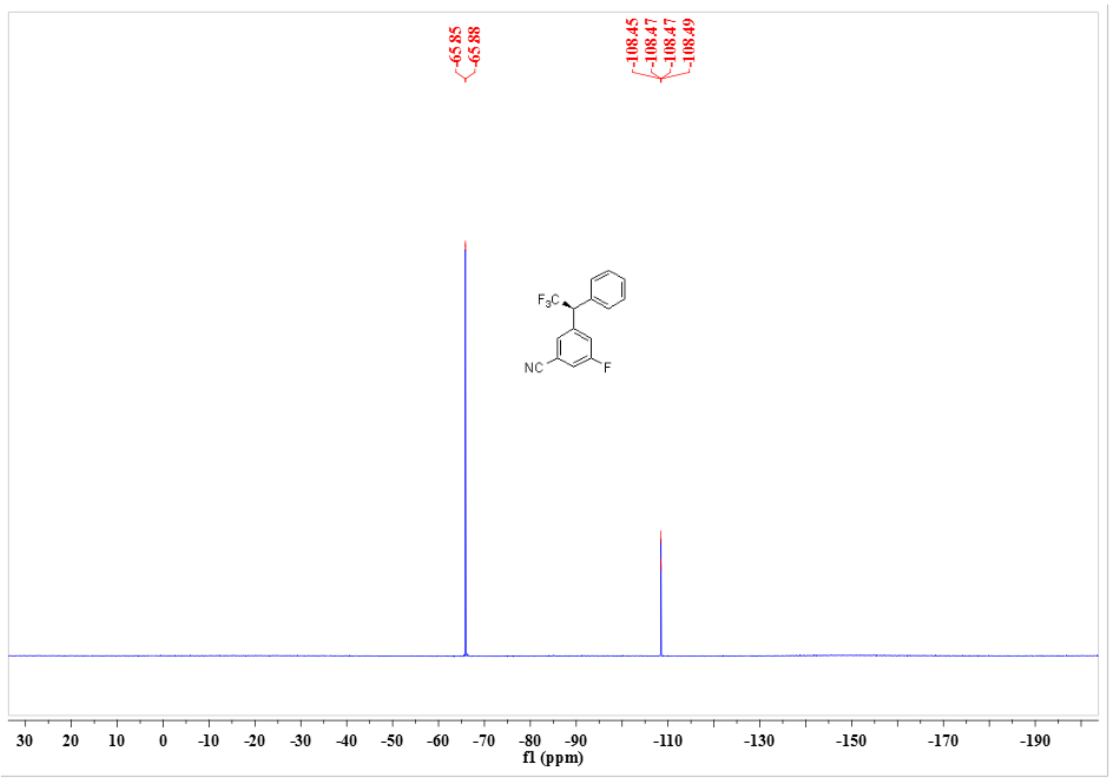

**Supplementary Figure 284.** **$^{19}$F NMR (376 MHz, CDCl$_3$) spectrum of 3w**

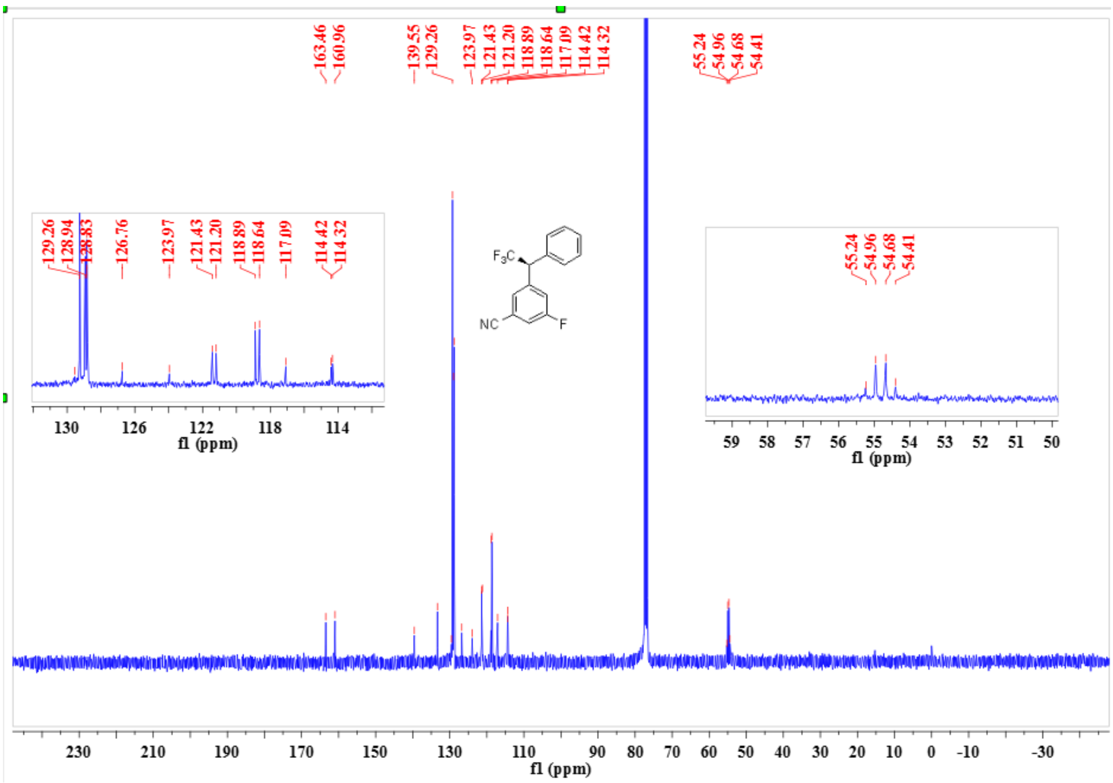


Supplementary Figure 285. $^{13}C$ NMR (101 MHz, $CDCl_3$) spectrum of 3w

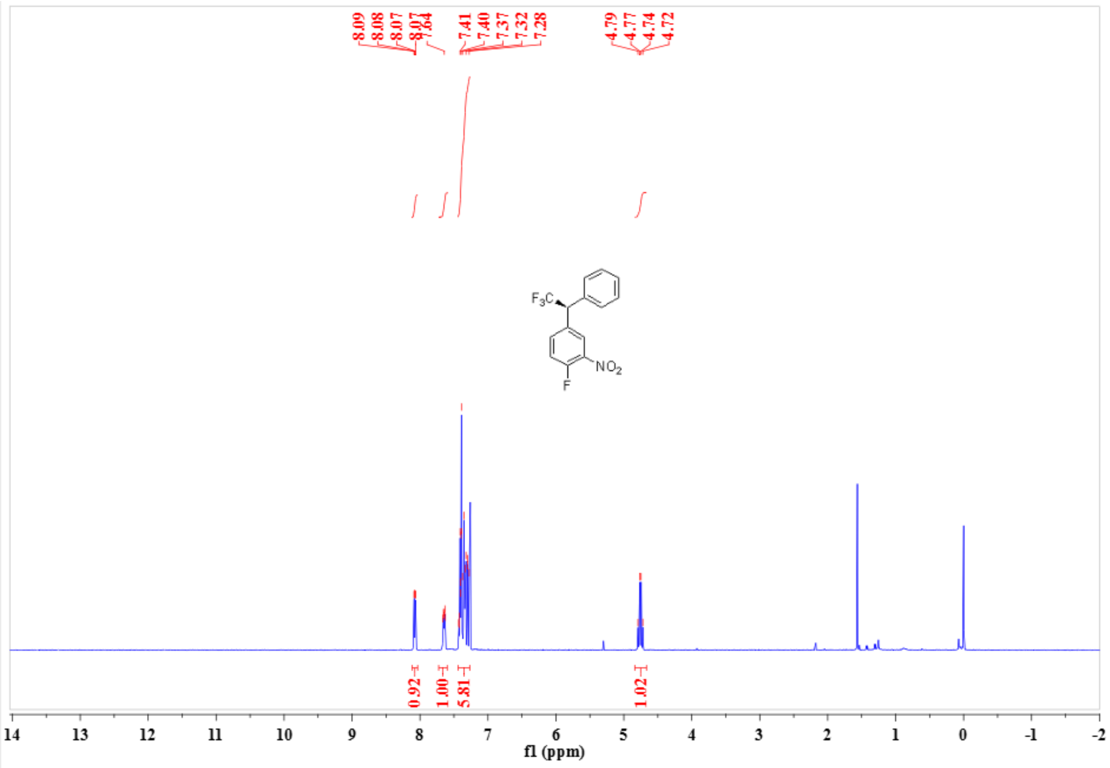


Supplementary Figure 286. $^{1}H$ NMR (400 MHz, $CDCl_3$) spectrum of 3x

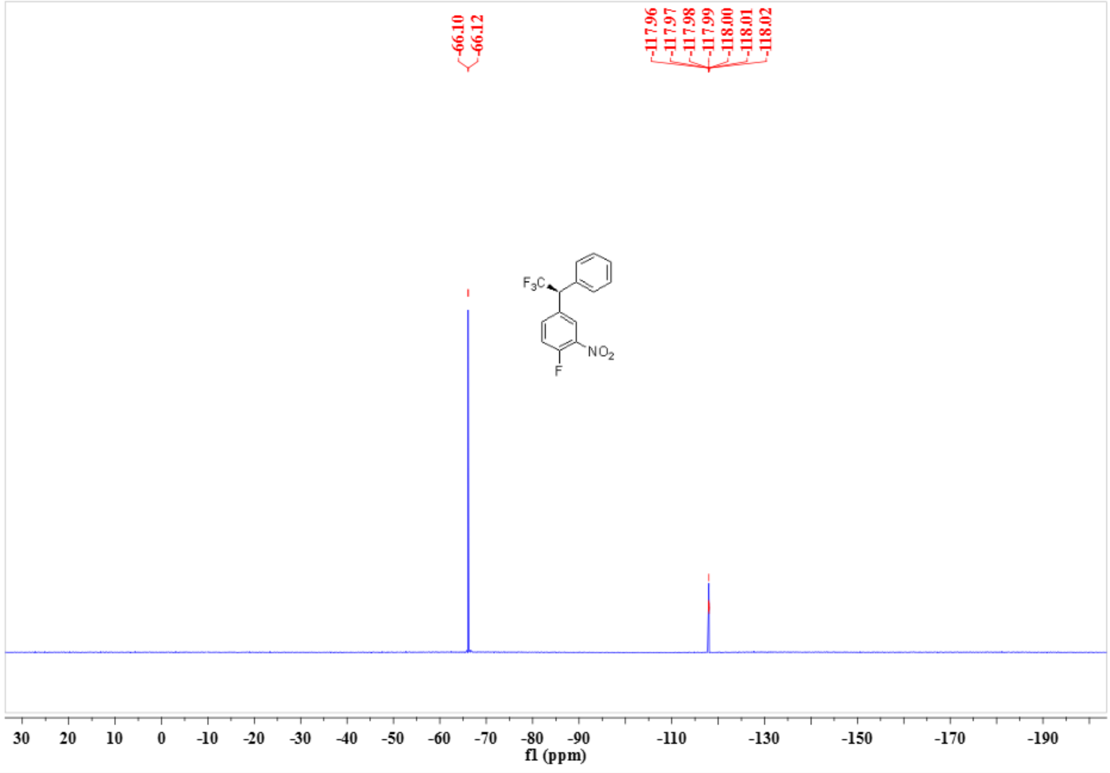

**Supplementary Figure 287.** $^{19}$F NMR (376 MHz, $CDCl_3$) spectrum of 3x

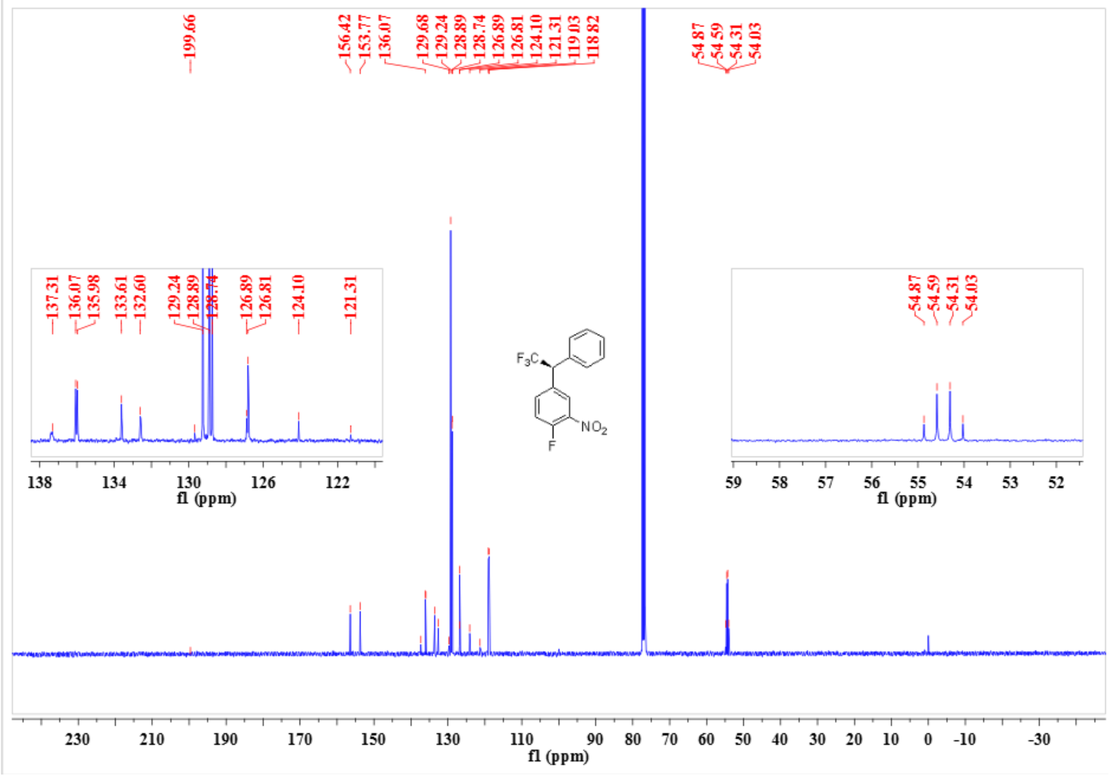

**Supplementary Figure 288.** $^{13}$C NMR (101 MHz, $CDCl_3$) spectrum of 3x

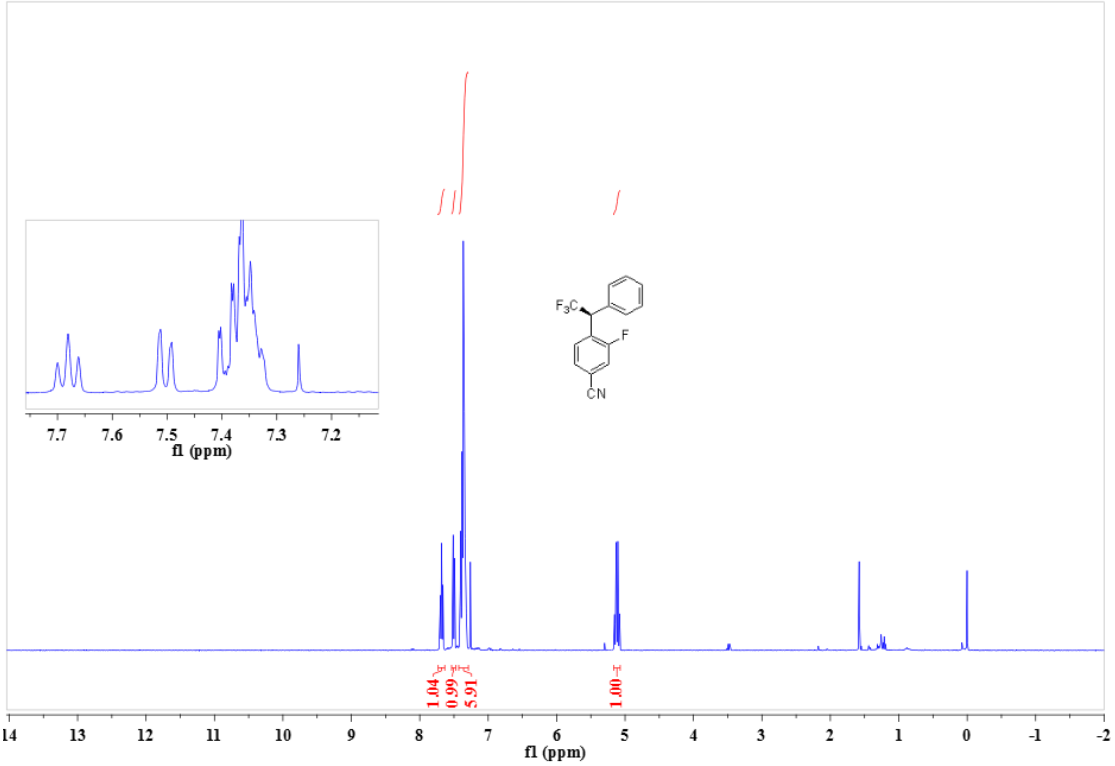


Supplementary Figure 289. $^1$H NMR (400 MHz, $CDCl_3$) spectrum of 3y

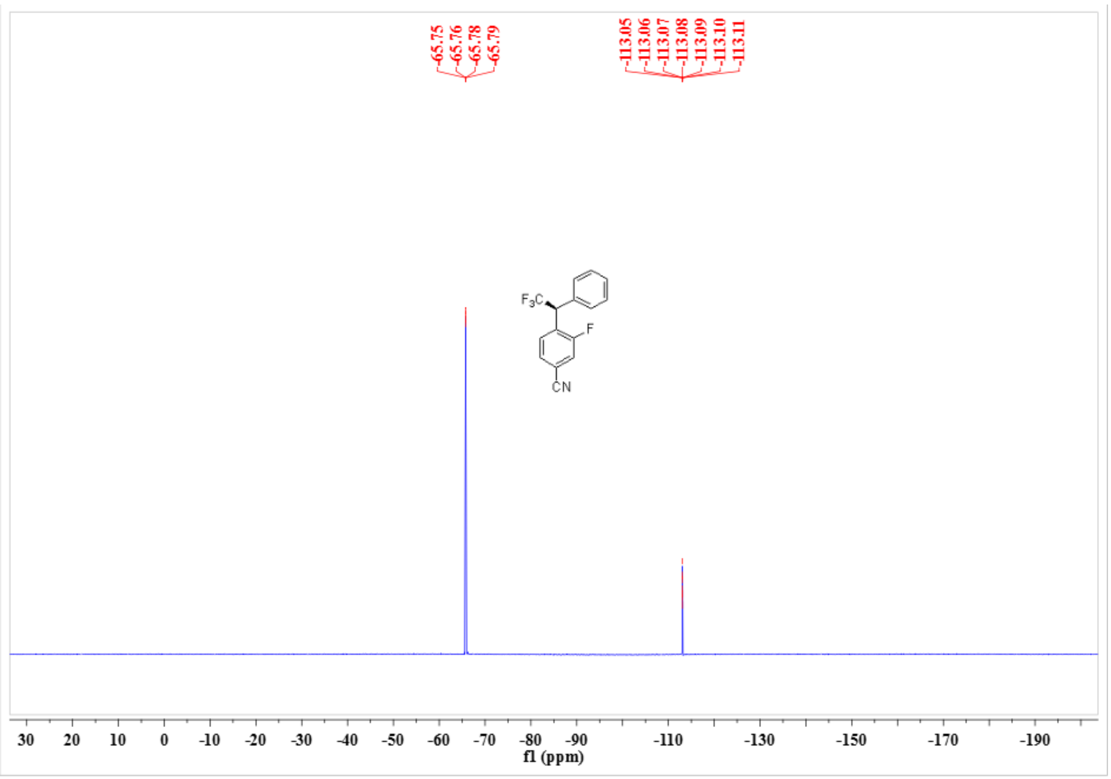


Supplementary Figure 290. $^{19}$F NMR (376 MHz, $CDCl_3$) spectrum of 3y

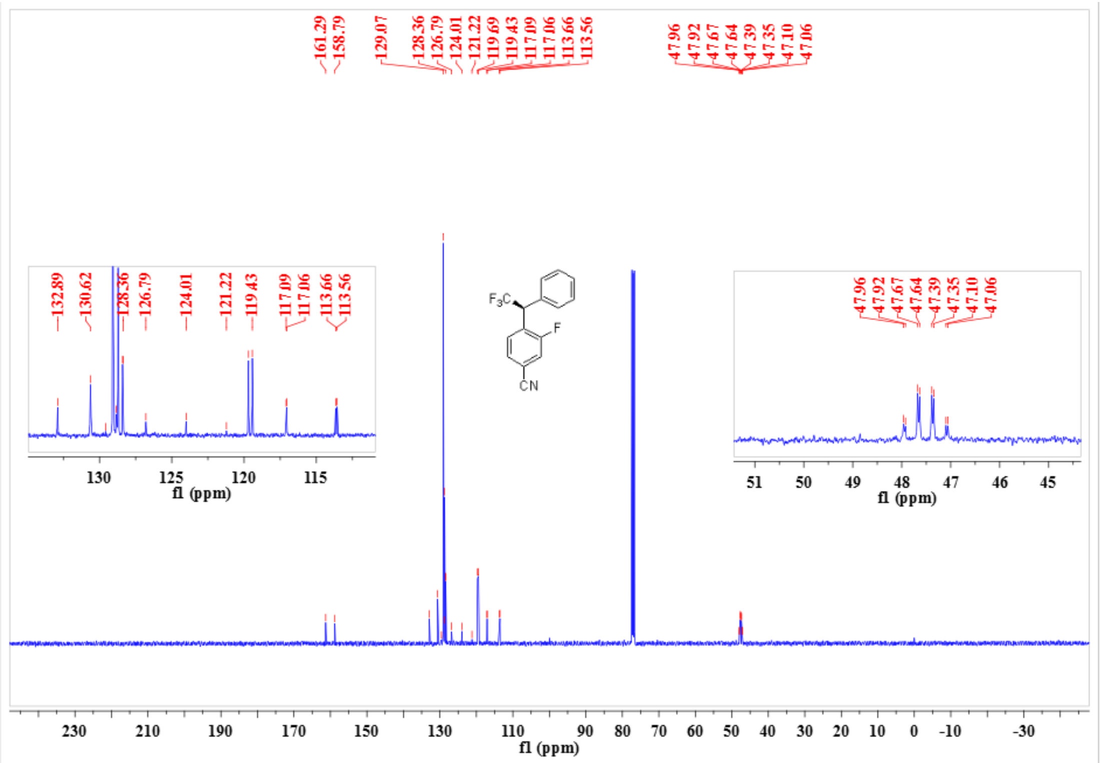

**Supplementary Figure 291.** $^{13}$C NMR (101 MHz, $CDCl_3$) spectrum of 3y

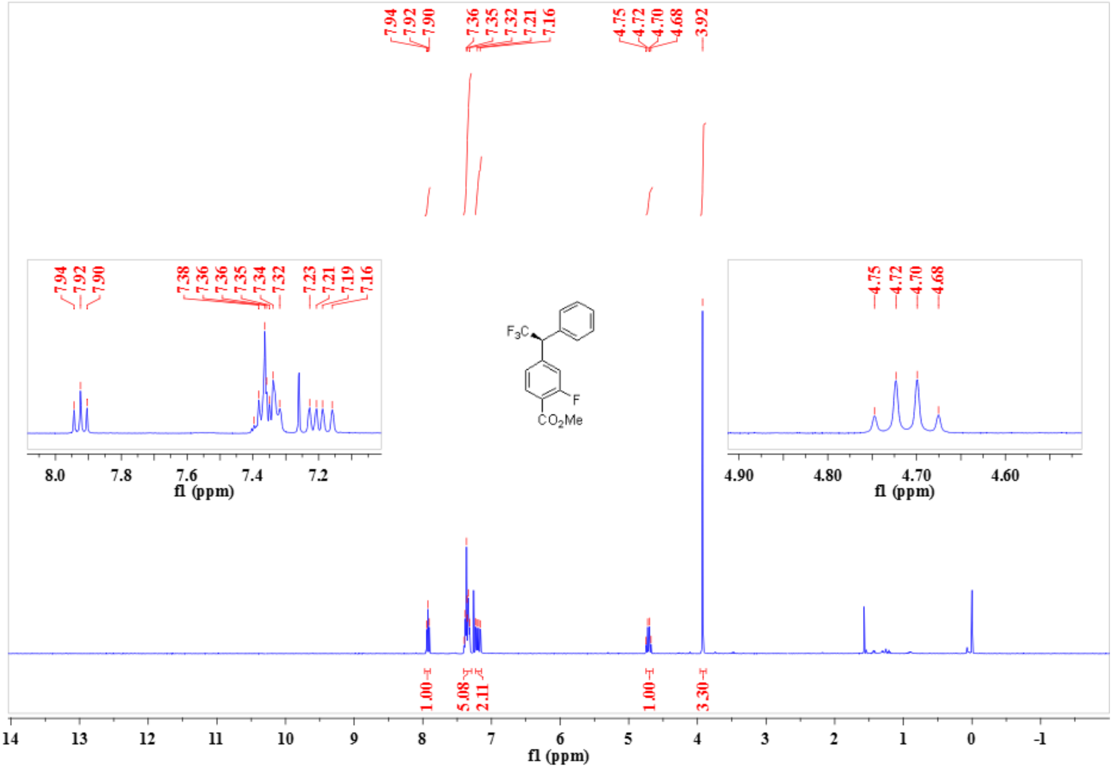

**Supplementary Figure 292.** $^{1}$H NMR (400 MHz, $CDCl_3$) spectrum of 3z

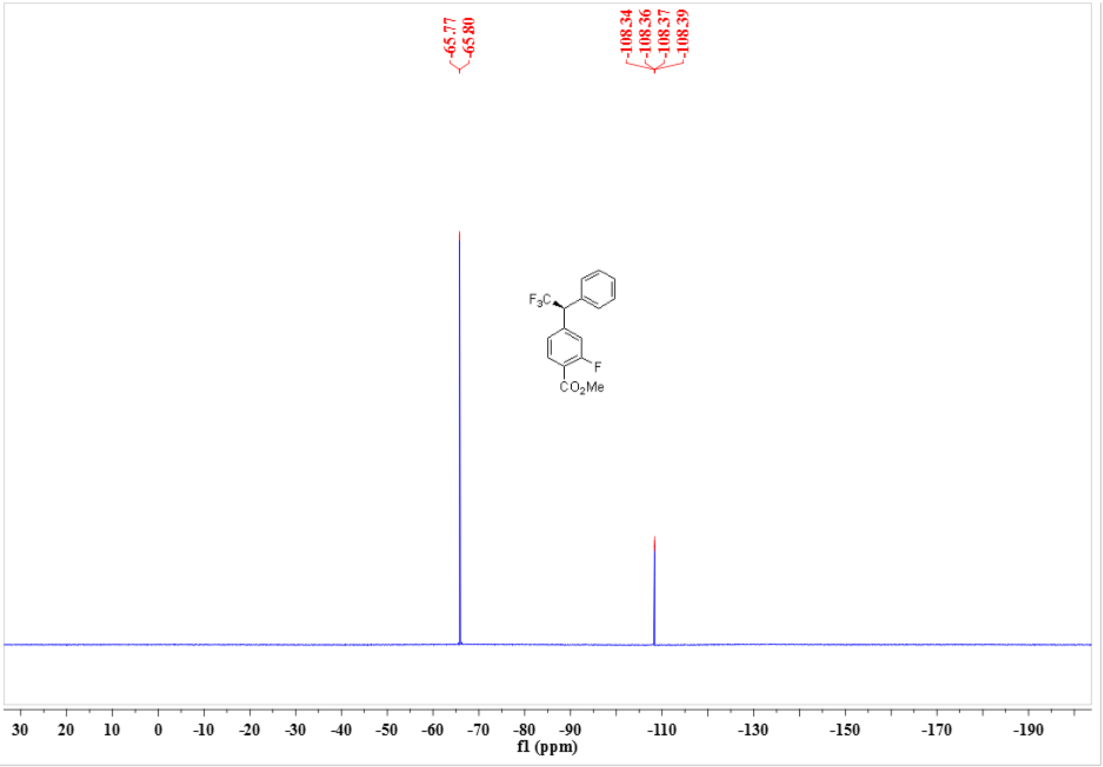


**Supplementary Figure 293.** $^{19}$**F NMR (376 MHz, CDCl**$_3$**) spectrum of 3z**

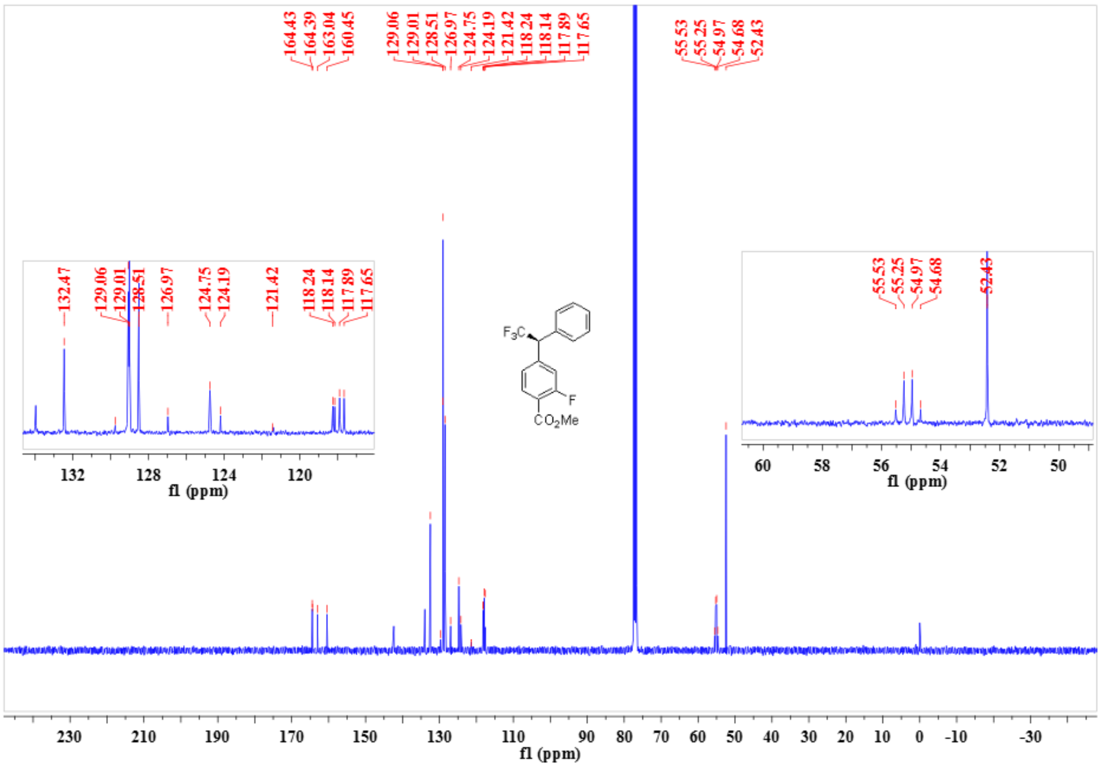


**Supplementary Figure 294.** $^{13}$**C NMR (101 MHz, CDCl**$_3$**) spectrum of 3z**

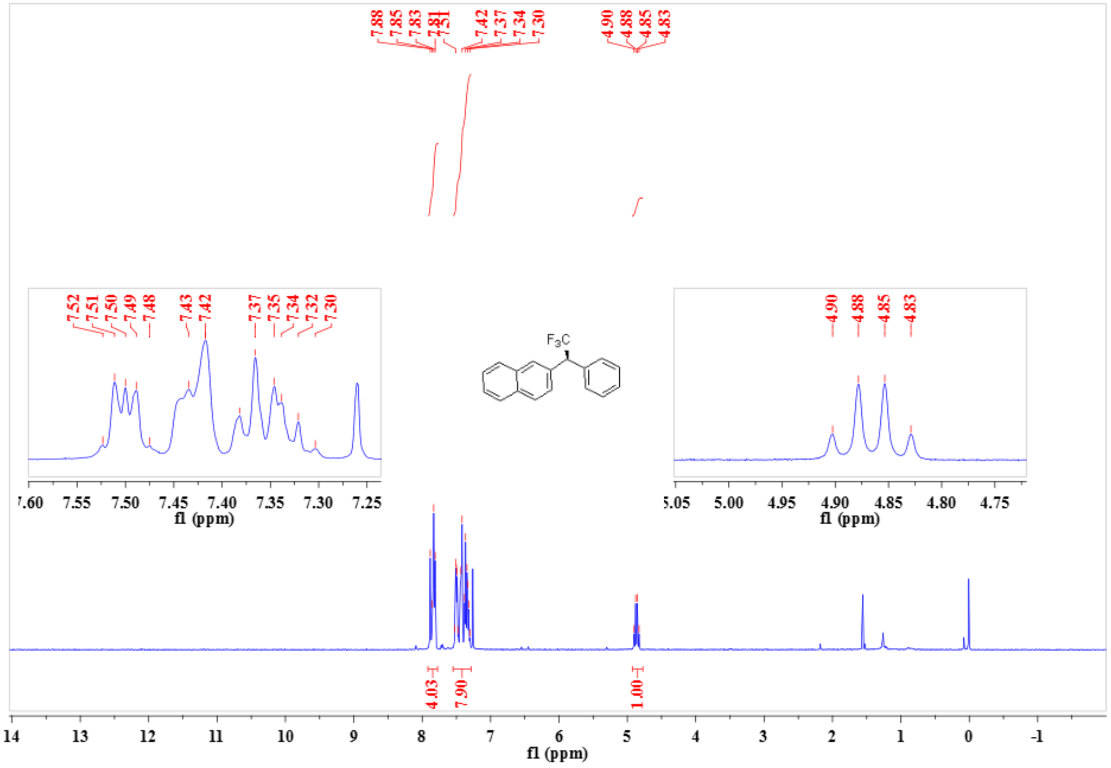

**Supplementary Figure 295.** $^{1}$**H NMR (400 MHz, CDCl$_3$) spectrum of 3aa**

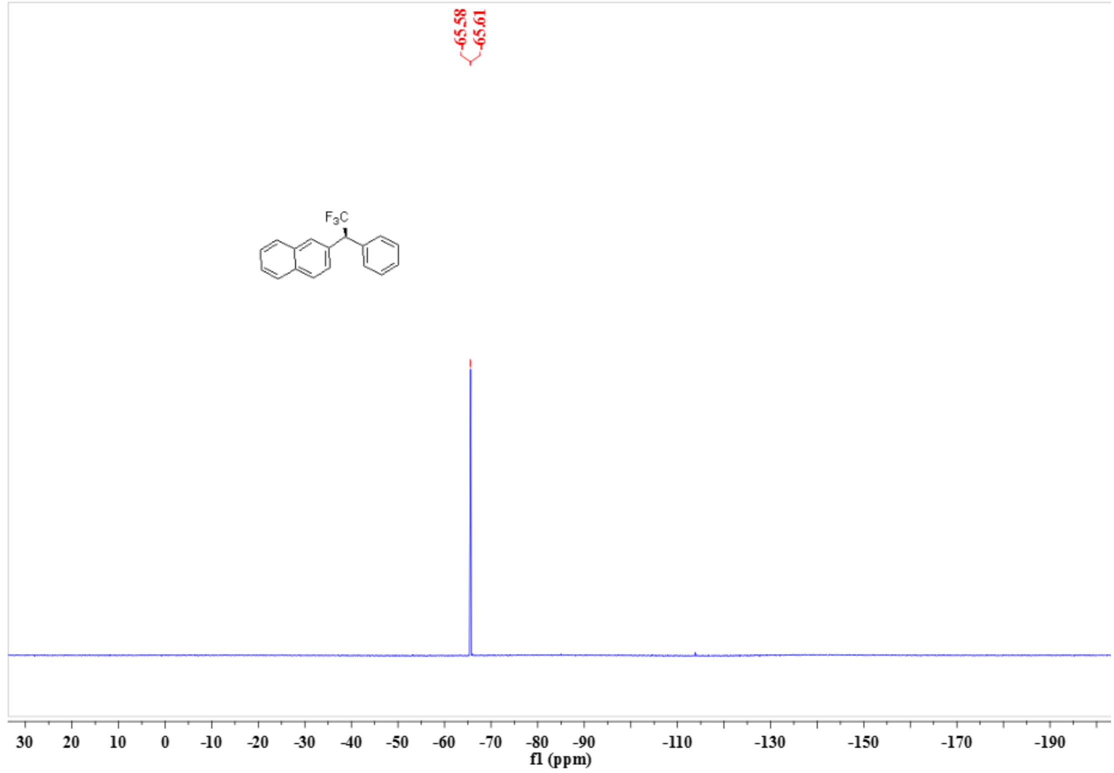

**Supplementary Figure 296.** $^{19}$**F NMR (376 MHz, CDCl$_3$) spectrum of 3aa**

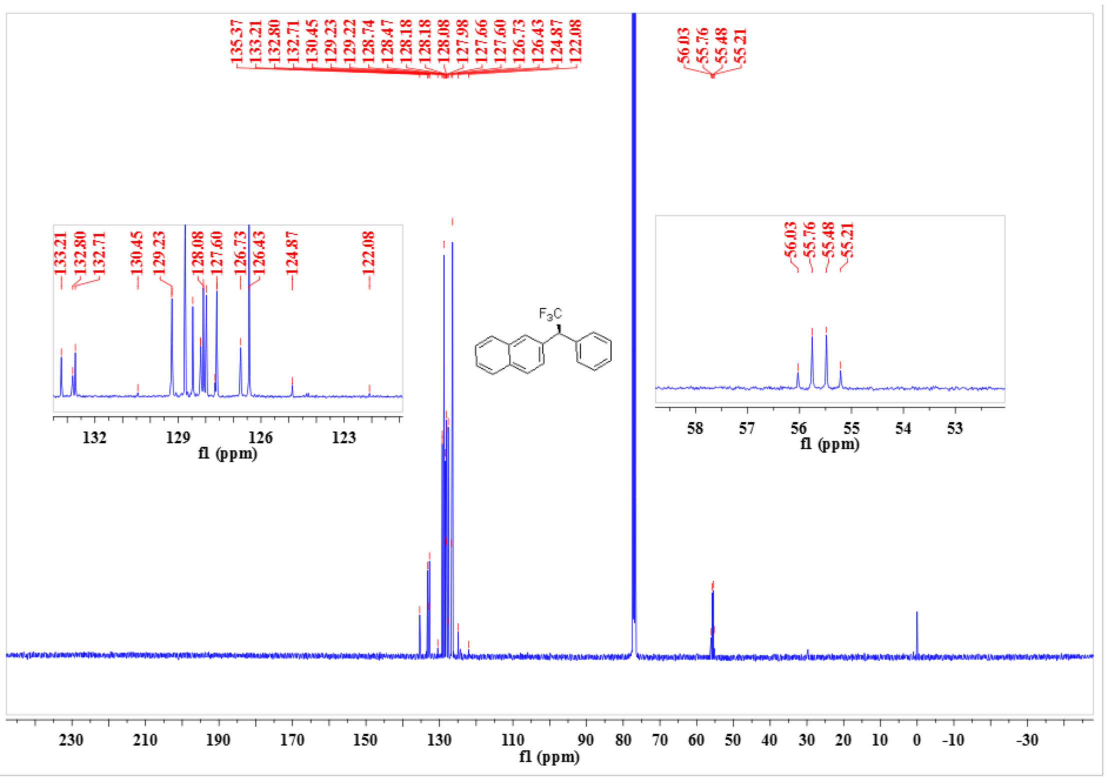


**Supplementary Figure 297.** $^{13}C$ **NMR (101 MHz,** $CDCl_3$**) spectrum of 3aa**

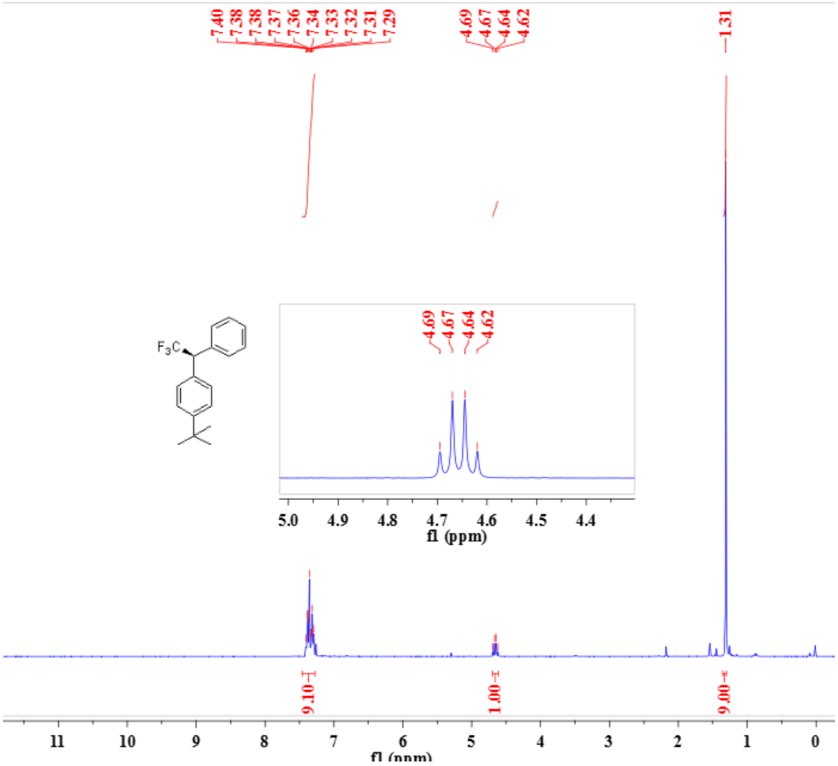


**Supplementary Figure 298.** $^{1}H$ **NMR (400 MHz,** $CDCl_3$**) spectrum of 3ab**

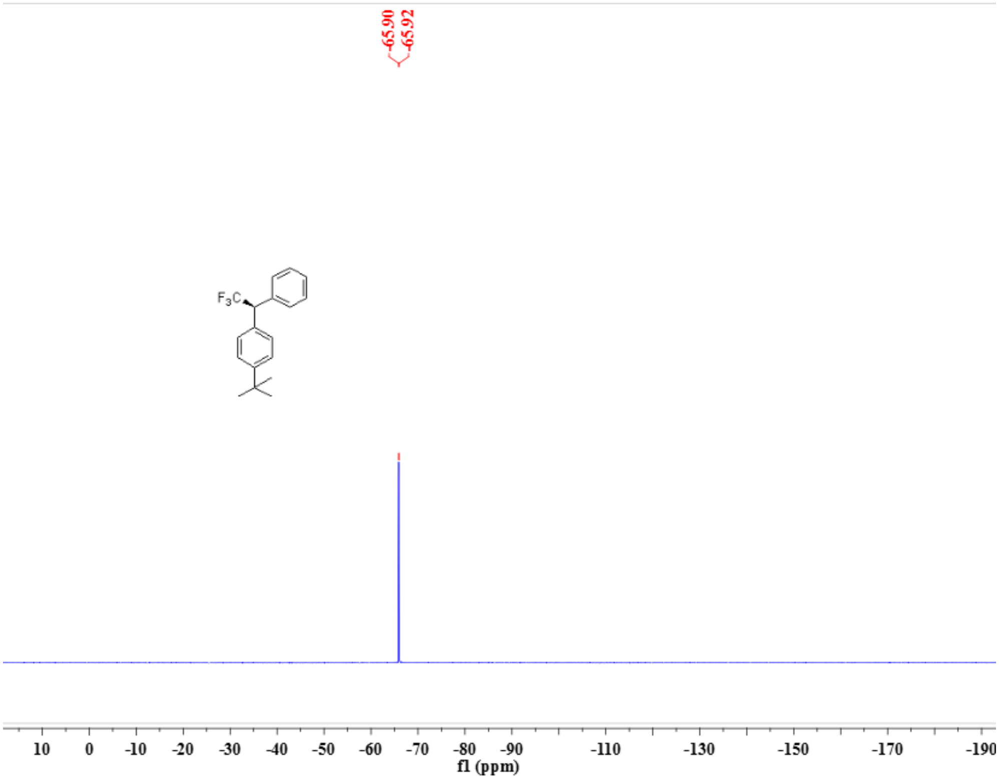


**Supplementary Figure 299. $^{19}$F NMR (376 MHz, CDCl$_3$) spectrum of 3ab**

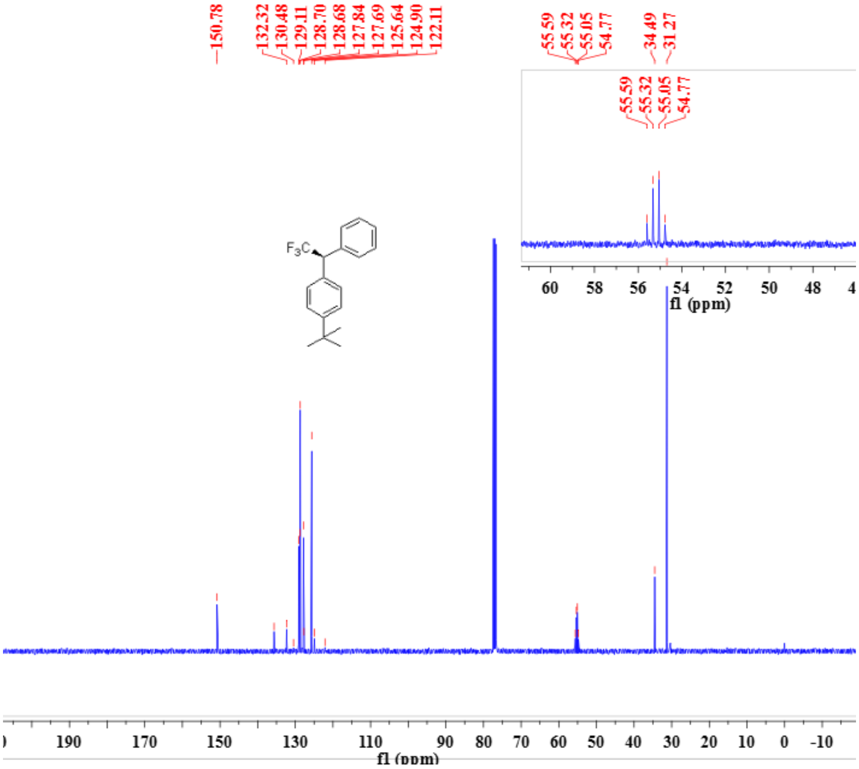


**Supplementary Figure 300. $^{13}$C NMR (101 MHz, CDCl$_3$) spectrum of 3ab**

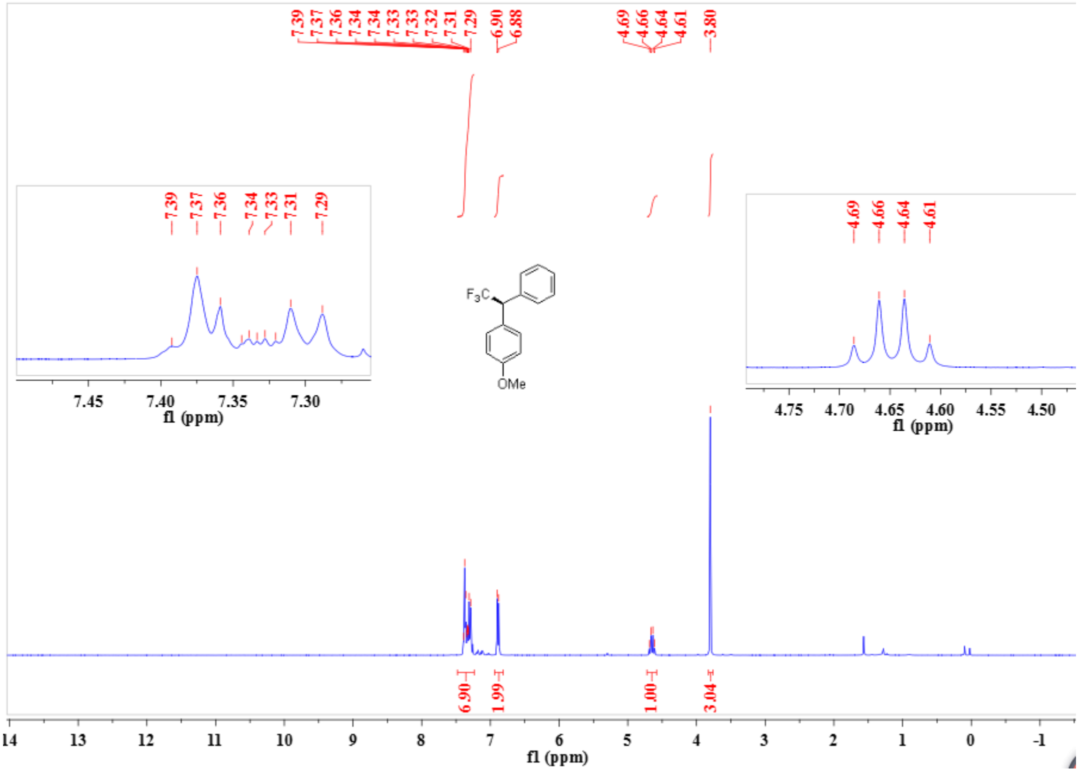


**Supplementary Figure 301.** $^{1}$**H NMR (400 MHz, CDCl$_3$) spectrum of 3ac**

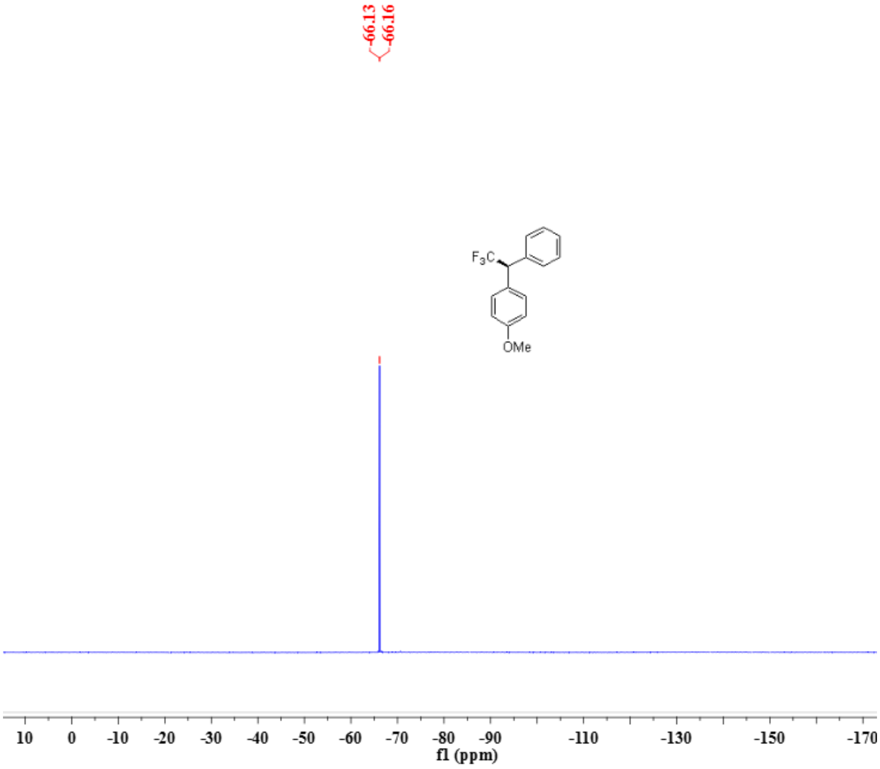


**Supplementary Figure 302.** $^{19}$**F NMR (376 MHz, CDCl$_3$) spectrum of 3ac**

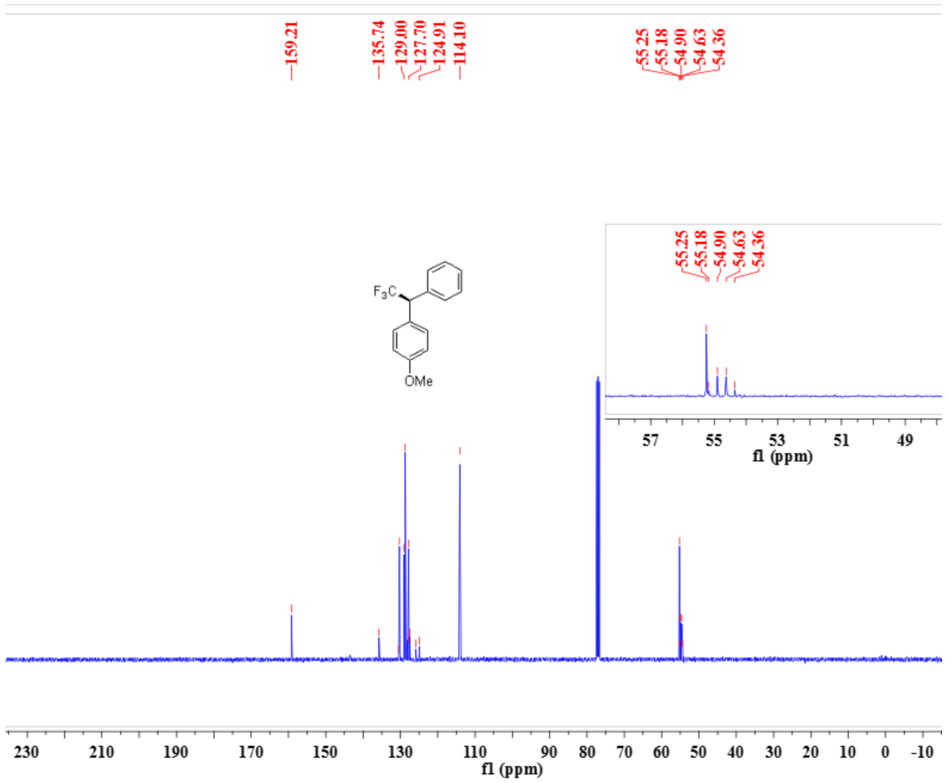


**Supplementary Figure 303.** $^{13}$C NMR (101 MHz, $CDCl_3$) spectrum of 3ac

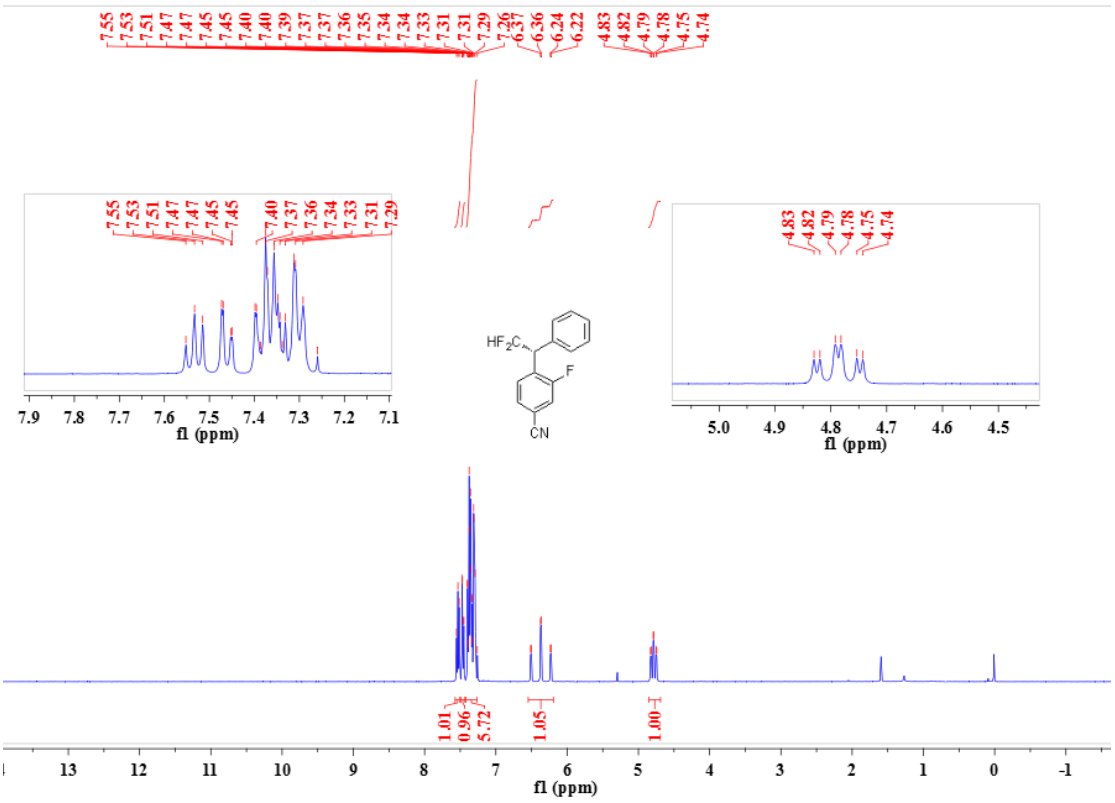


**Supplementary Figure 304.** $^{1}$H NMR (400 MHz, $CDCl_3$) spectrum of 4a

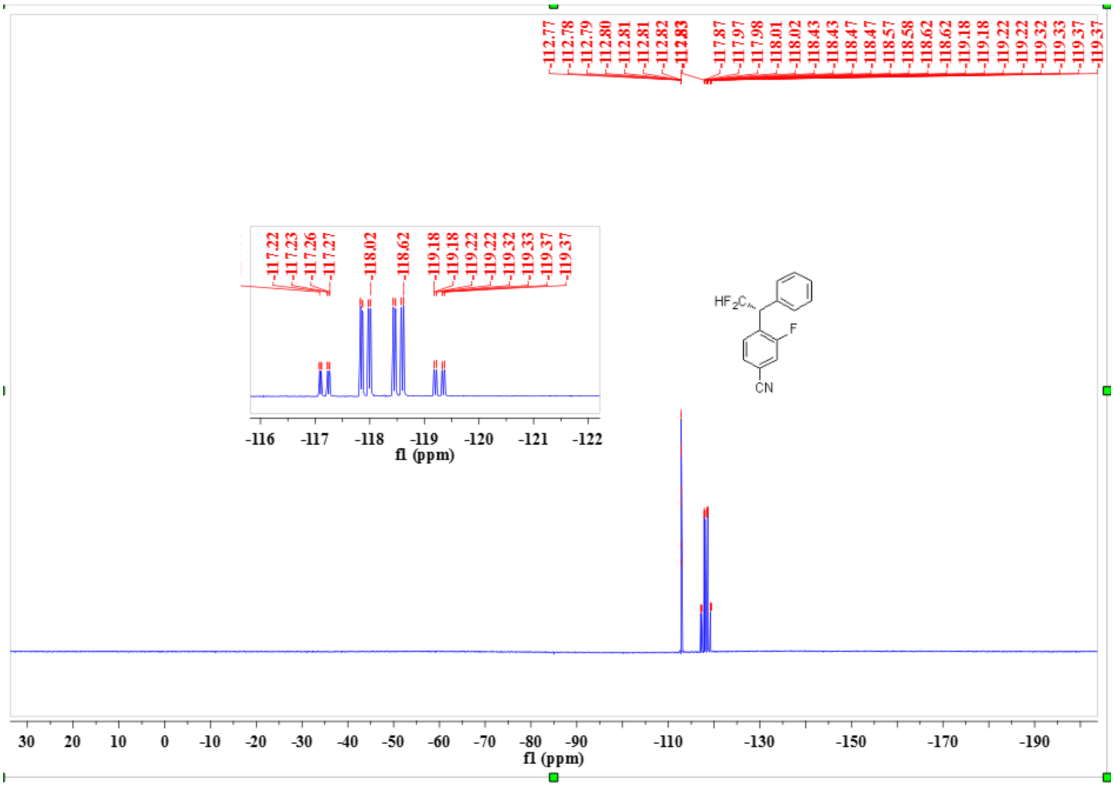

Supplementary Figure 305. $^{19}$F NMR (376 MHz, $CDCl_3$) spectrum of 4a

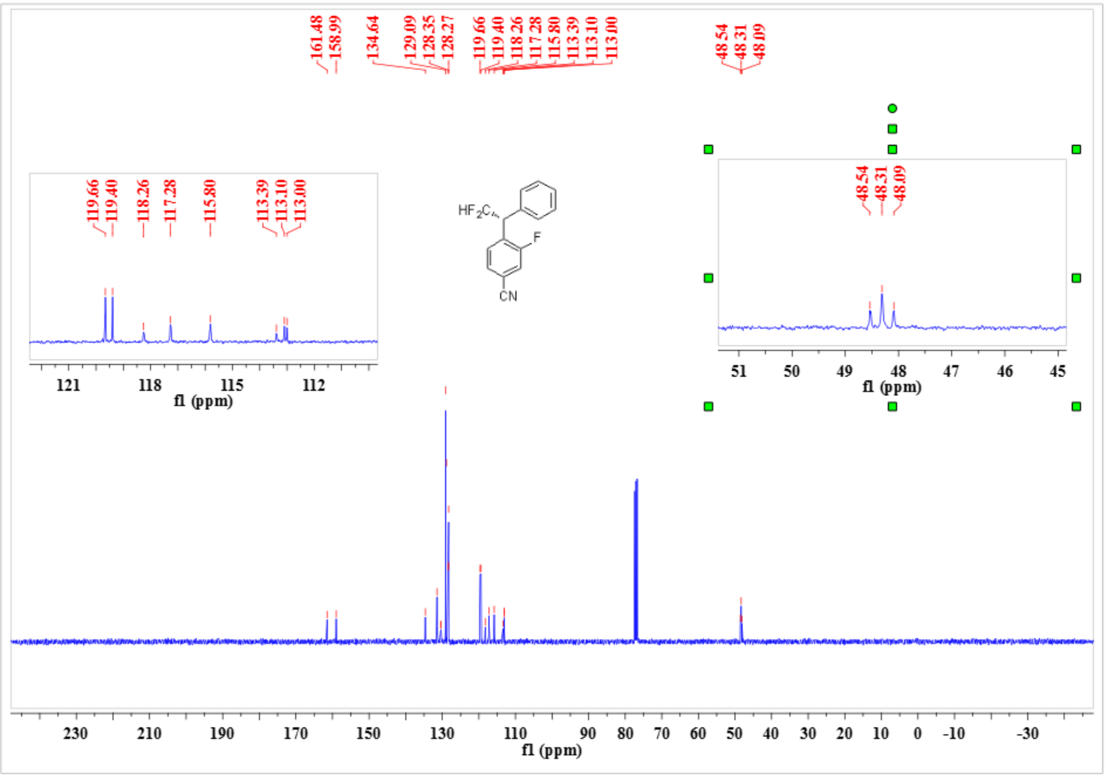

Supplementary Figure 306. $^{13}$C NMR (101 MHz, $CDCl_3$) spectrum of 4a

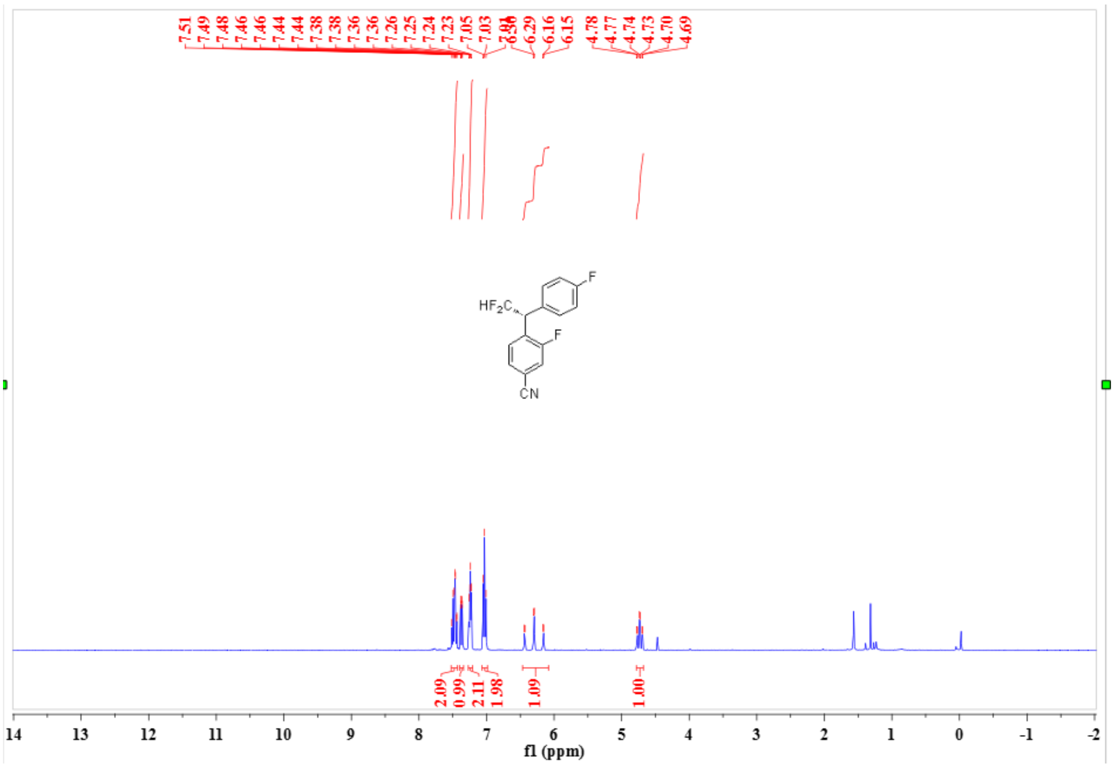

**Supplementary Figure 307.** $^{1}H$ **NMR (400 MHz,** $CDCl_3$**) spectrum of 4b**

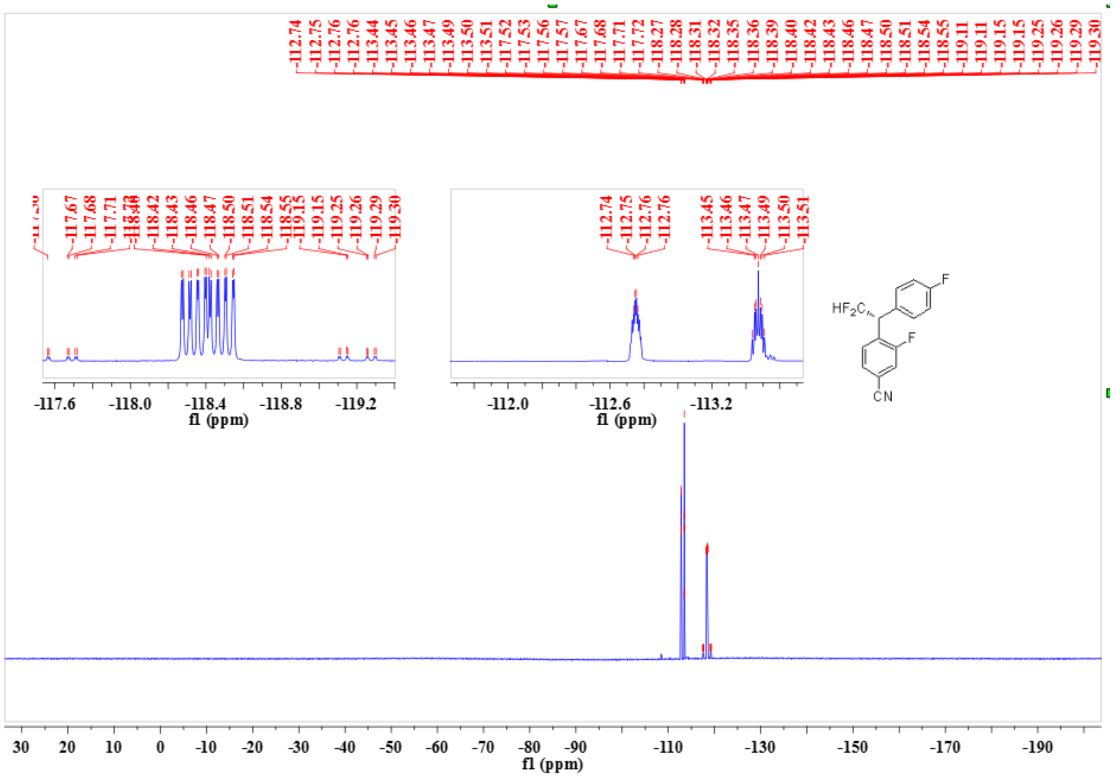

**Supplementary Figure 308.** $^{19}F$ **NMR (376 MHz,** $CDCl_3$**) spectrum of 4b**

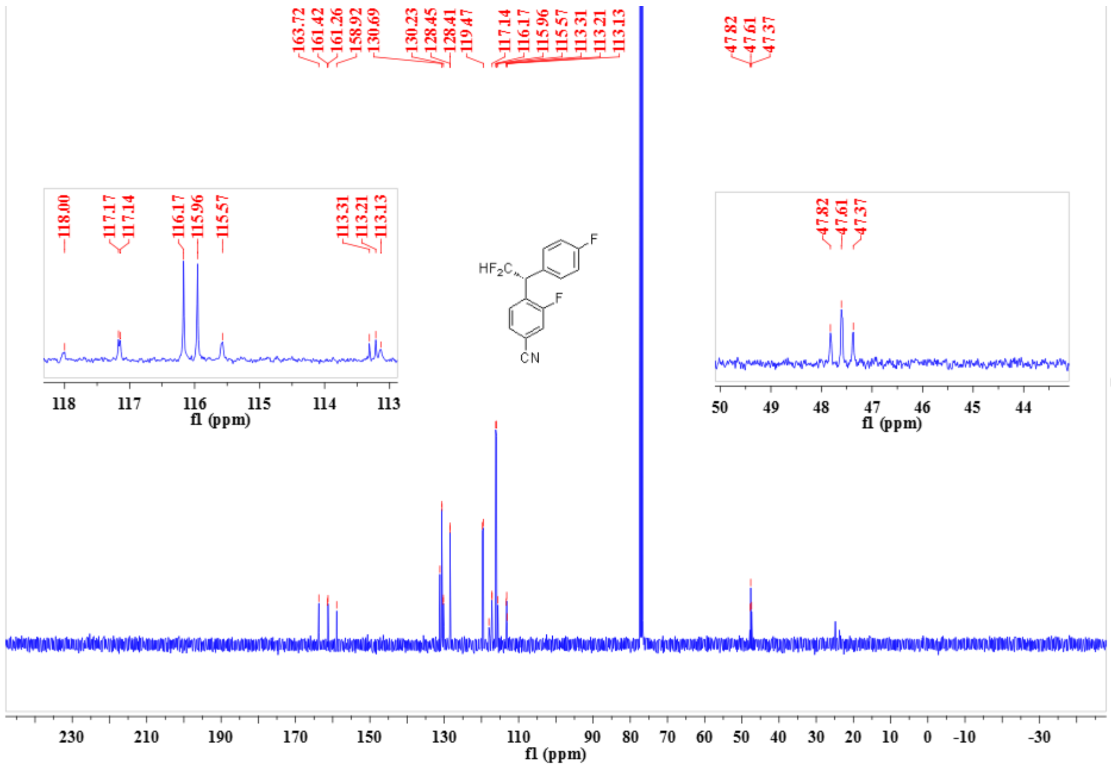


Supplementary Figure 309. $^{13}C$ NMR (101 MHz, $CDCl_3$) spectrum of 4b

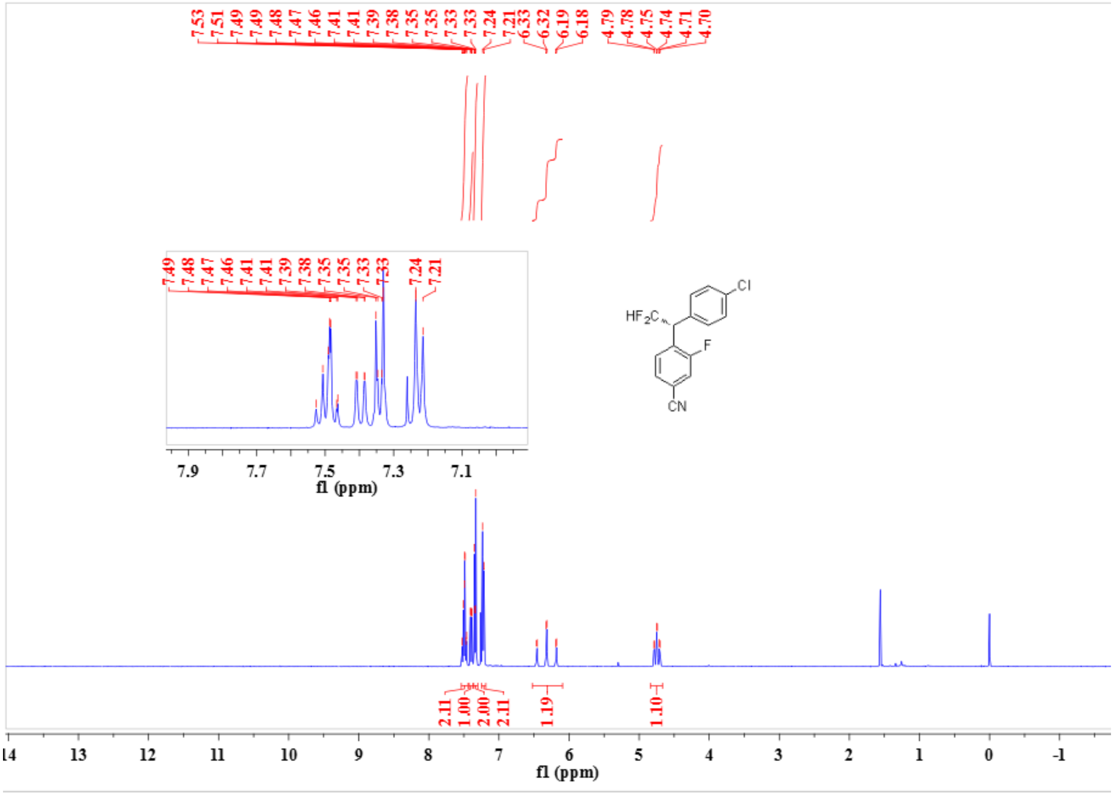


Supplementary Figure 310. $^{1}H$ NMR (400 MHz, $CDCl_3$) spectrum of 4c

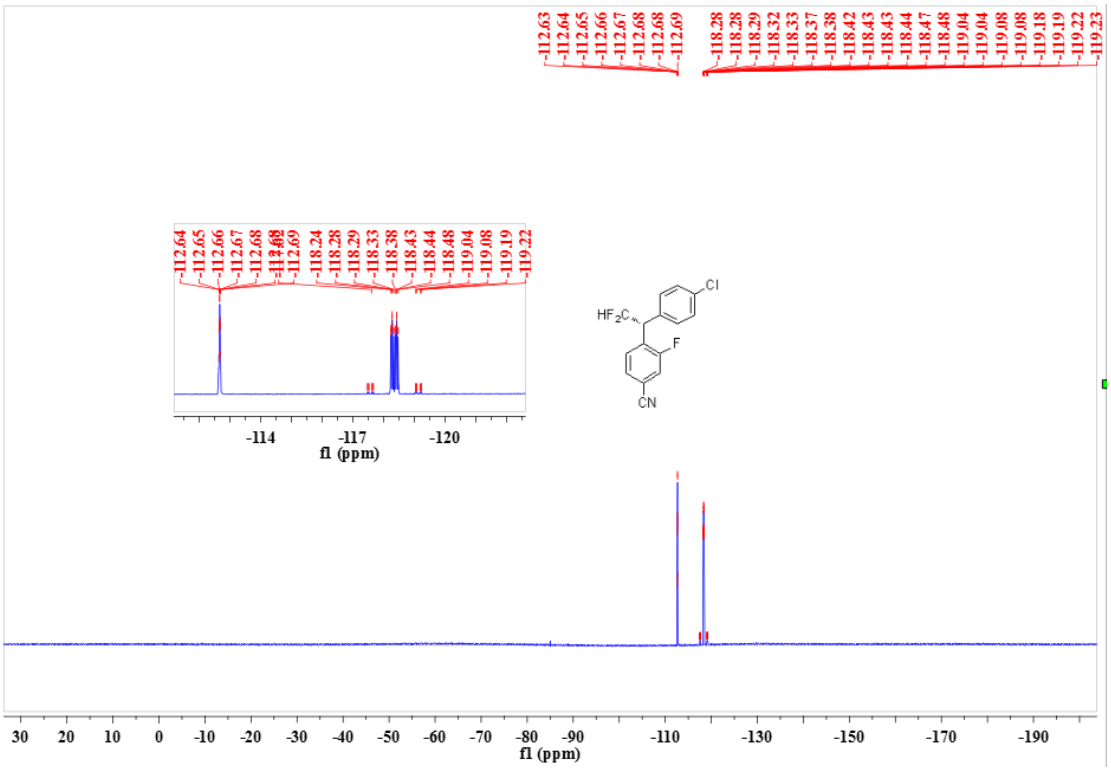

**Supplementary Figure 311.** $^{19}F$ **NMR (376 MHz, $CDCl_3$) spectrum of 4c**

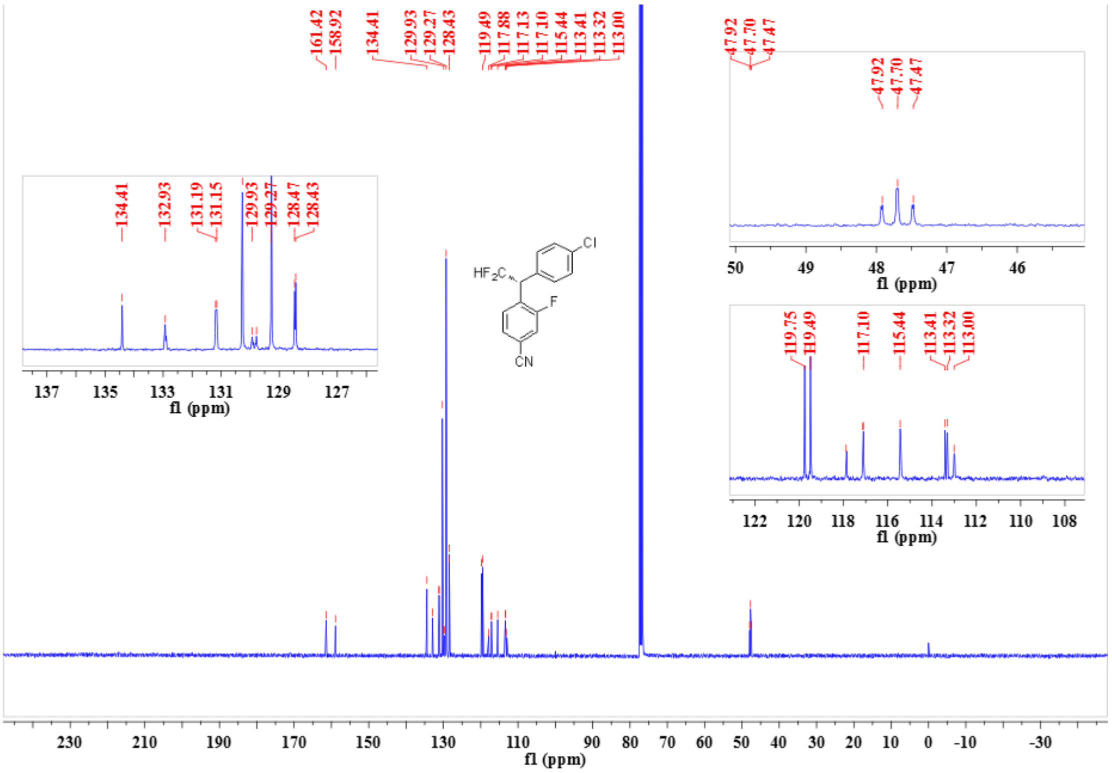

**Supplementary Figure 312.** $^{13}C$ **NMR (101 MHz, $CDCl_3$) spectrum of 4c**

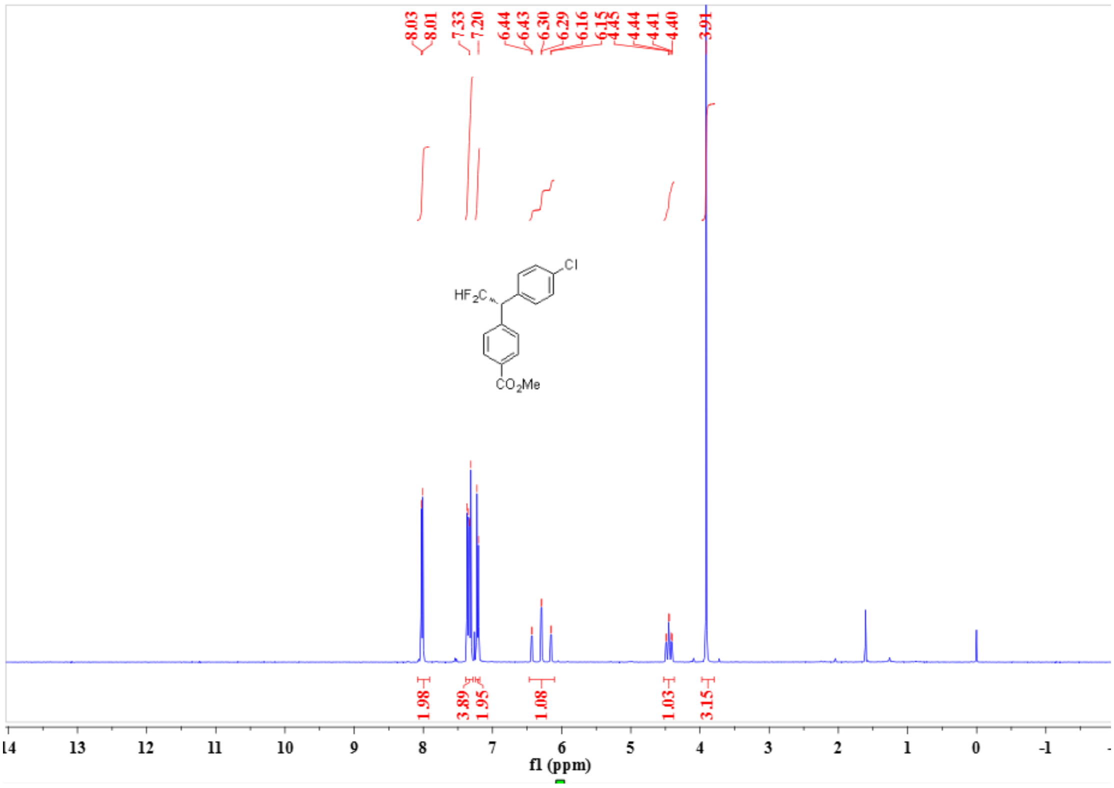

**Supplementary Figure 313.** **$^{1}$H NMR (400 MHz, $CDCl_3$) spectrum of 4d**

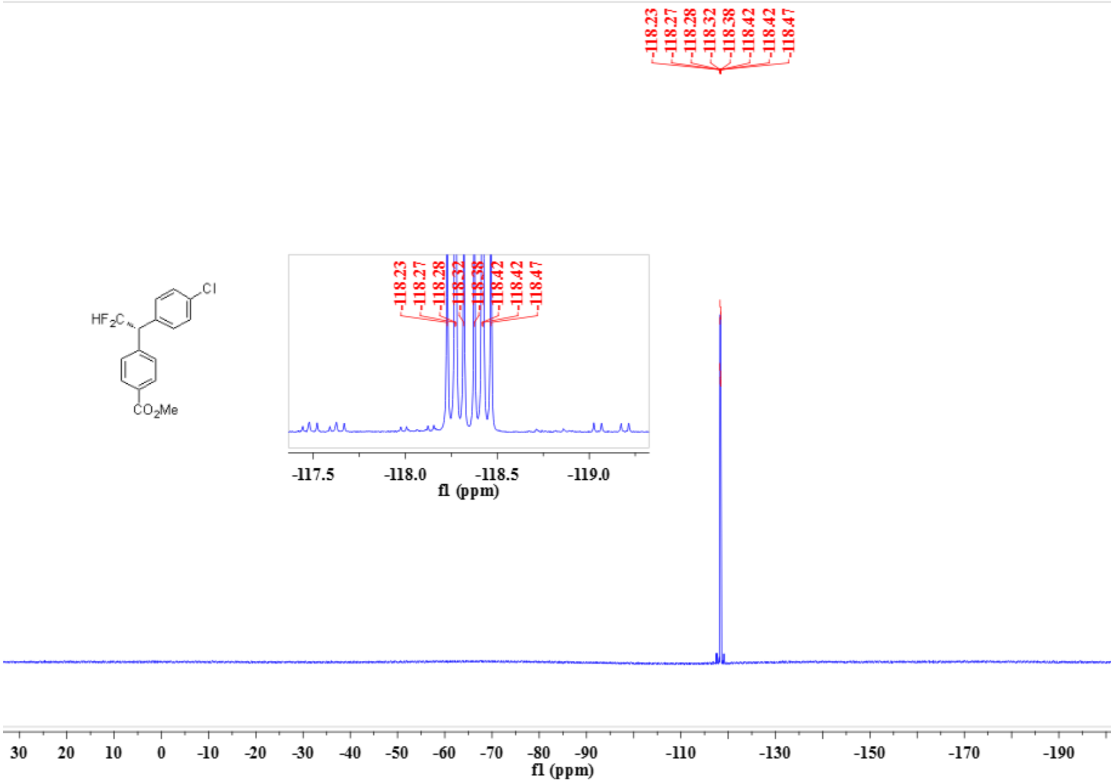

**Supplementary Figure 314.** **$^{19}$F NMR (376 MHz, $CDCl_3$) spectrum of 4d**

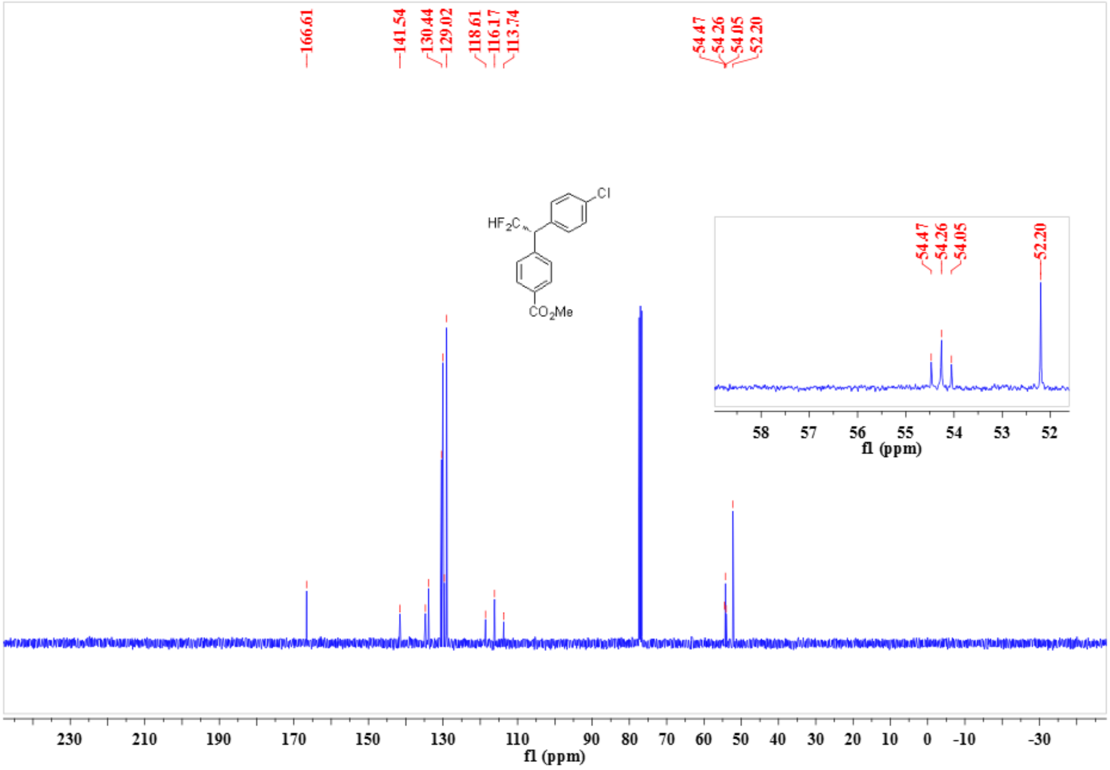


**Supplementary Figure 315.** $^{13}$C NMR (101 MHz, $CDCl_3$) spectrum of 4d

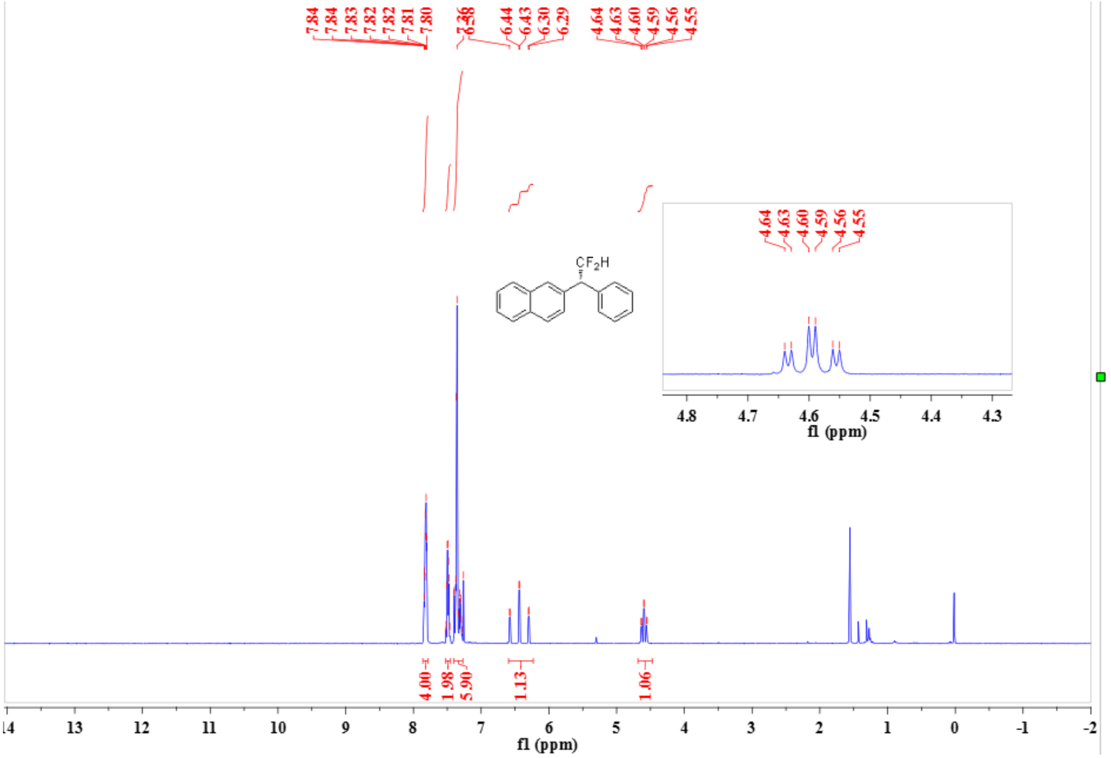


**Supplementary Figure 316.** $^{1}$H NMR (400 MHz, $CDCl_3$) spectrum of 4e

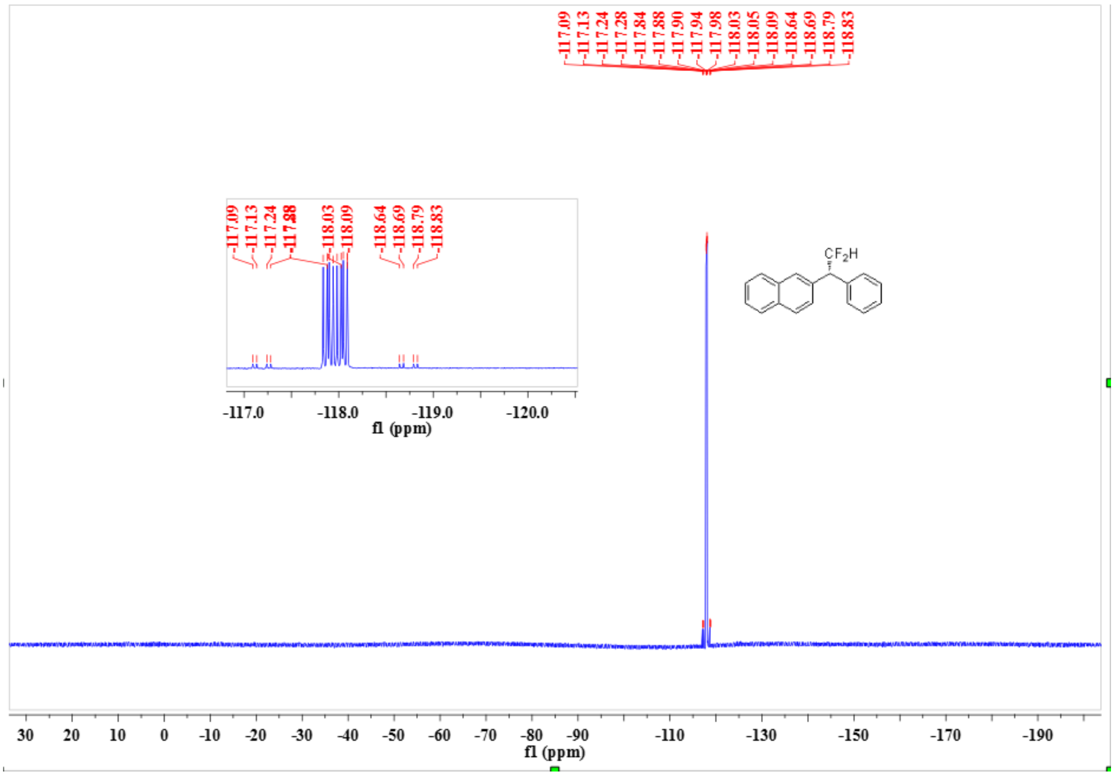

**Supplementary Figure 317. $^{19}F$ NMR (376 MHz, $CDCl_3$) spectrum of 4e**

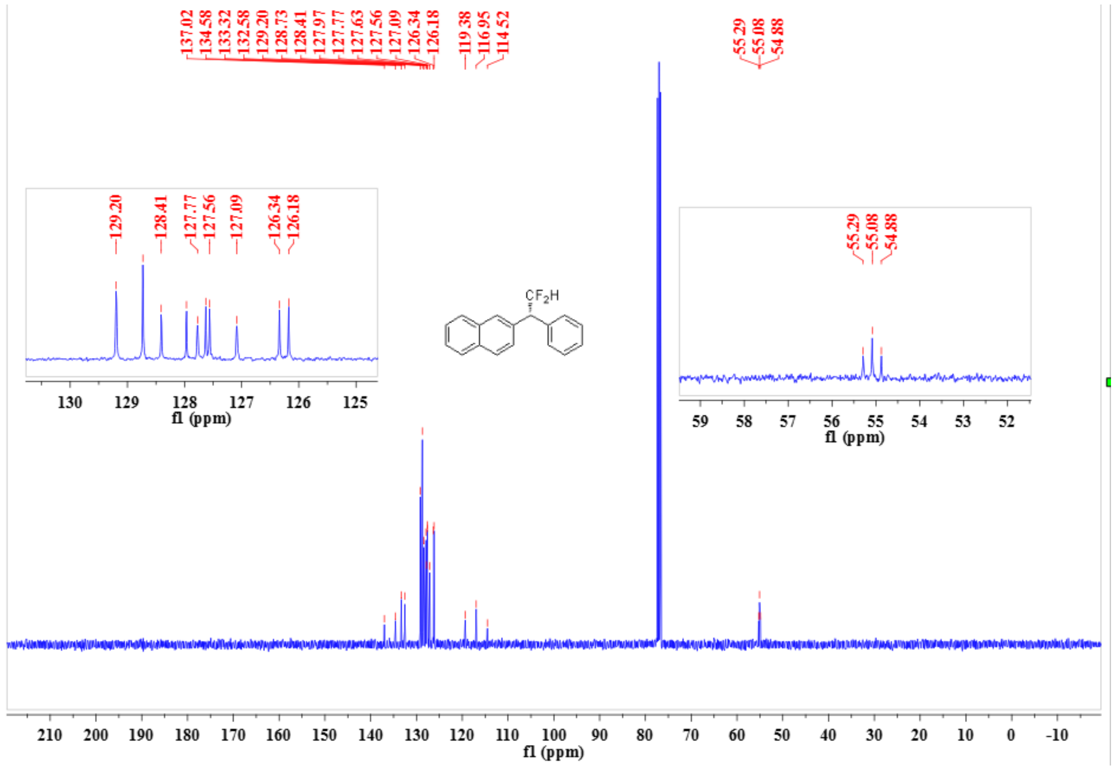

**Supplementary Figure 318. $^{13}C$ NMR (101 MHz, $CDCl_3$) spectrum of 4e**

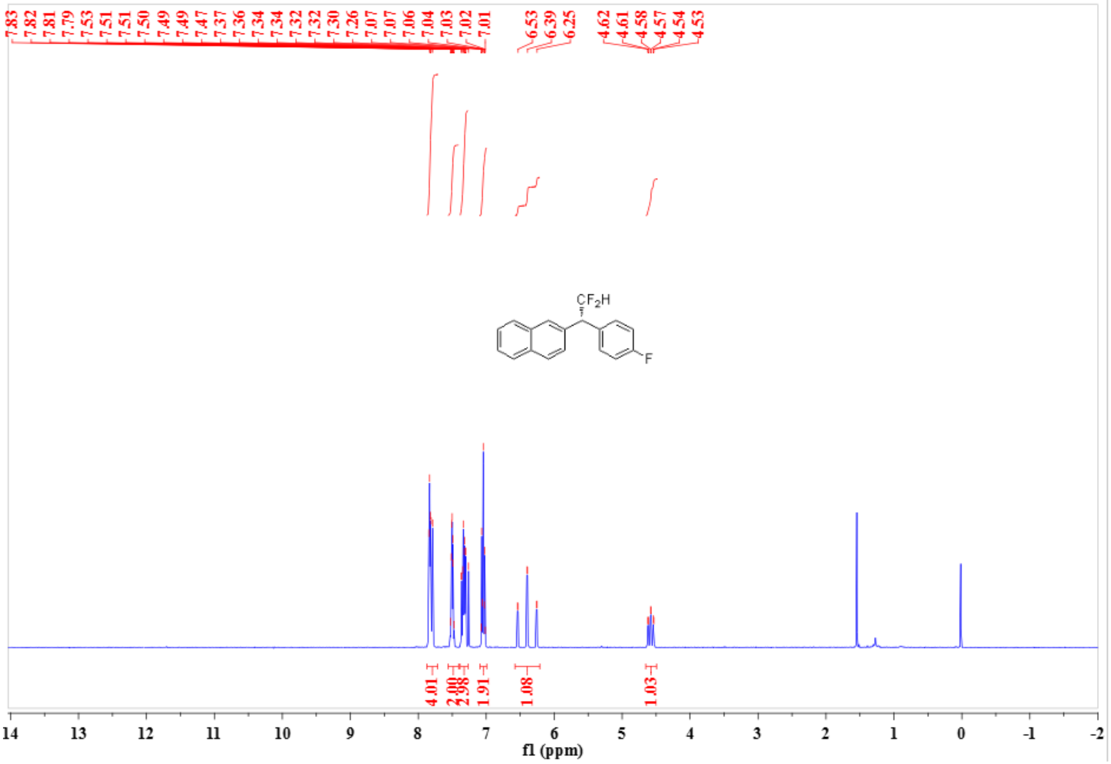

**Supplementary Figure 318.** $^{1}$**H NMR (400 MHz, CDCl$_3$) spectrum of 4f**

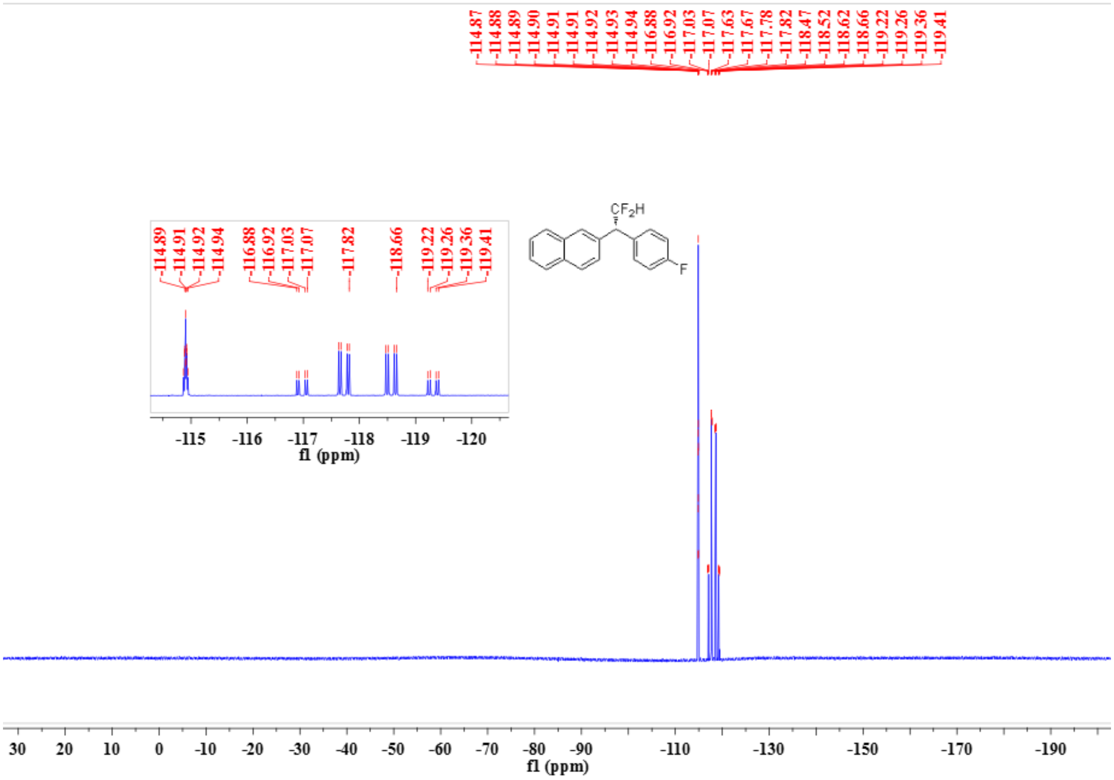

**Supplementary Figure 320.** $^{19}$**F NMR (376 MHz, CDCl$_3$) spectrum of 4f**

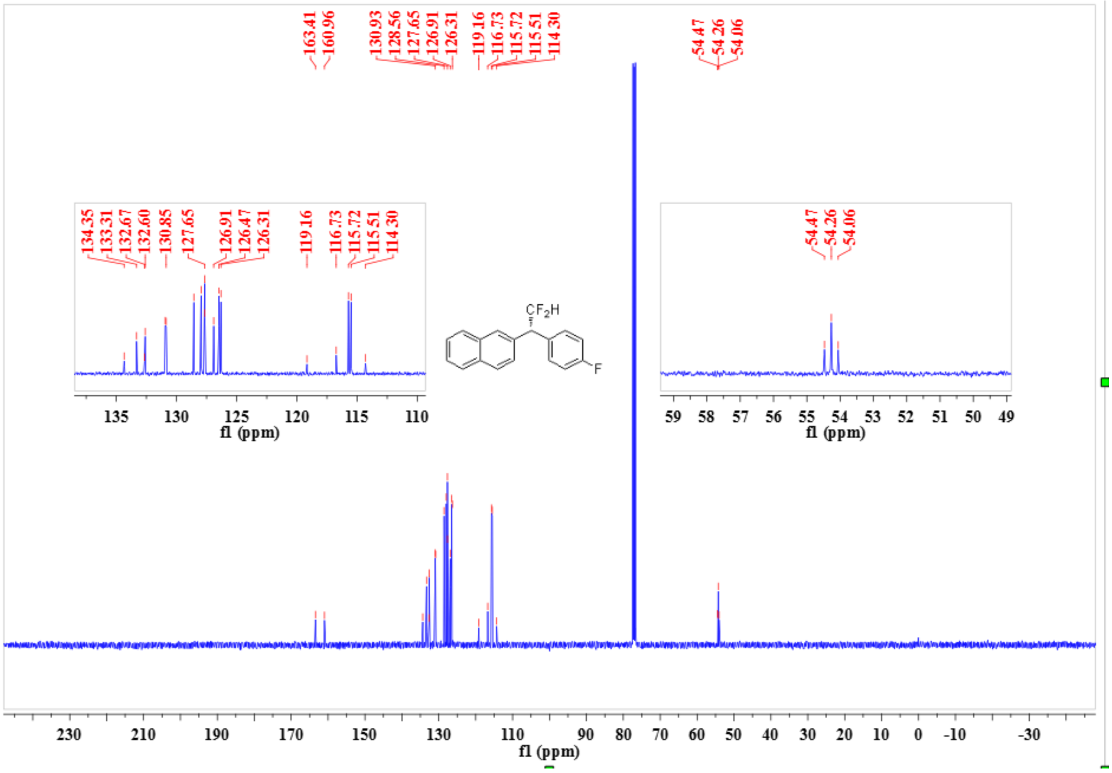


Supplementary Figure 321. $^{13}$C NMR (101 MHz, $CDCl_3$) spectrum of 4f

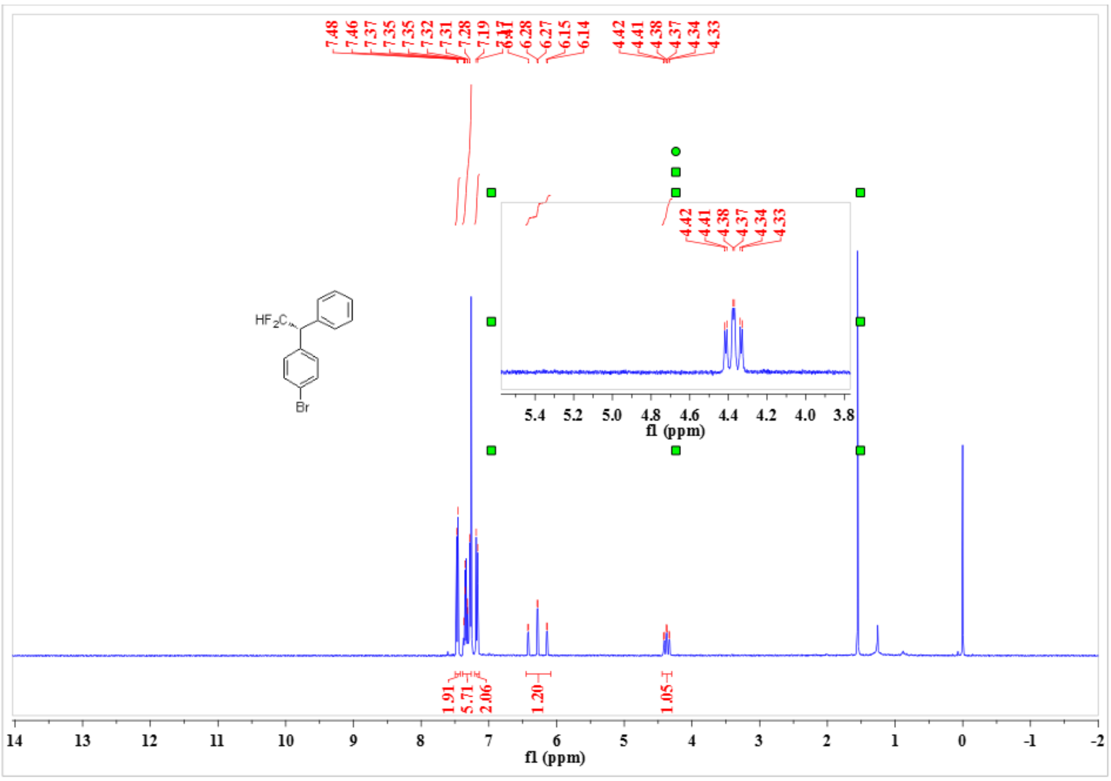


Supplementary Figure 322. $^{1}$H NMR (400 MHz, $CDCl_3$) spectrum of 4g

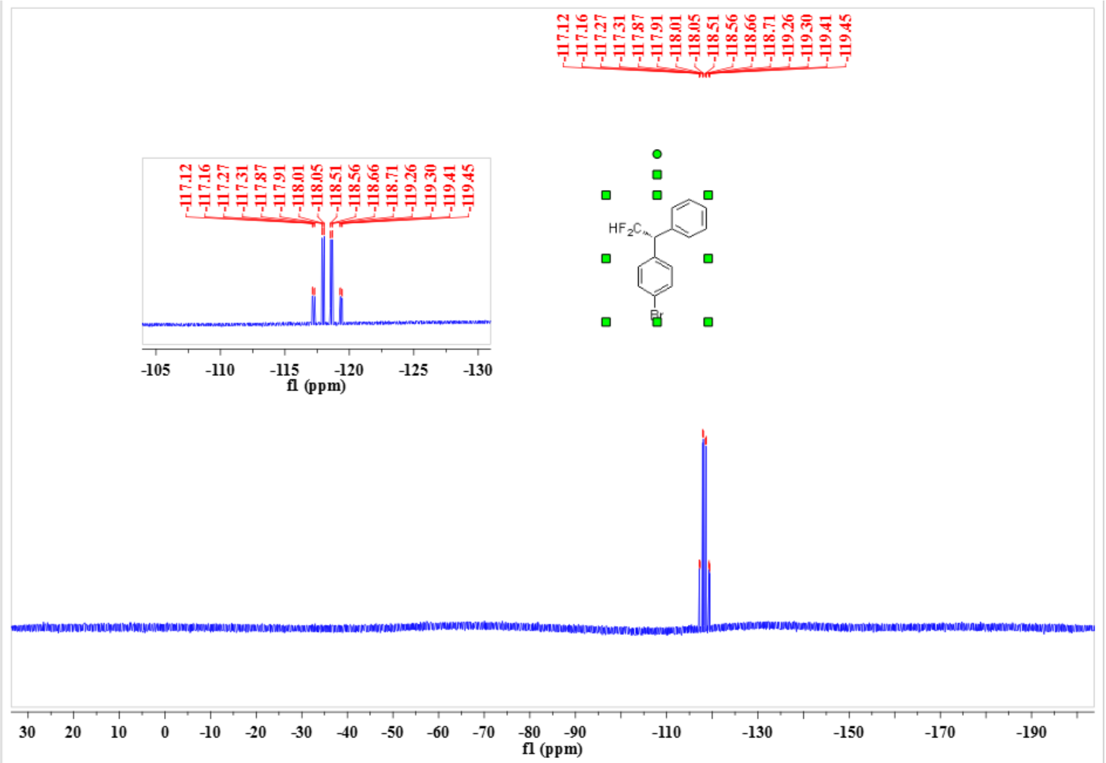


**Supplementary Figure 323.** $^{19}F$ **NMR (376 MHz,** $CDCl_3$**) spectrum of 4g**

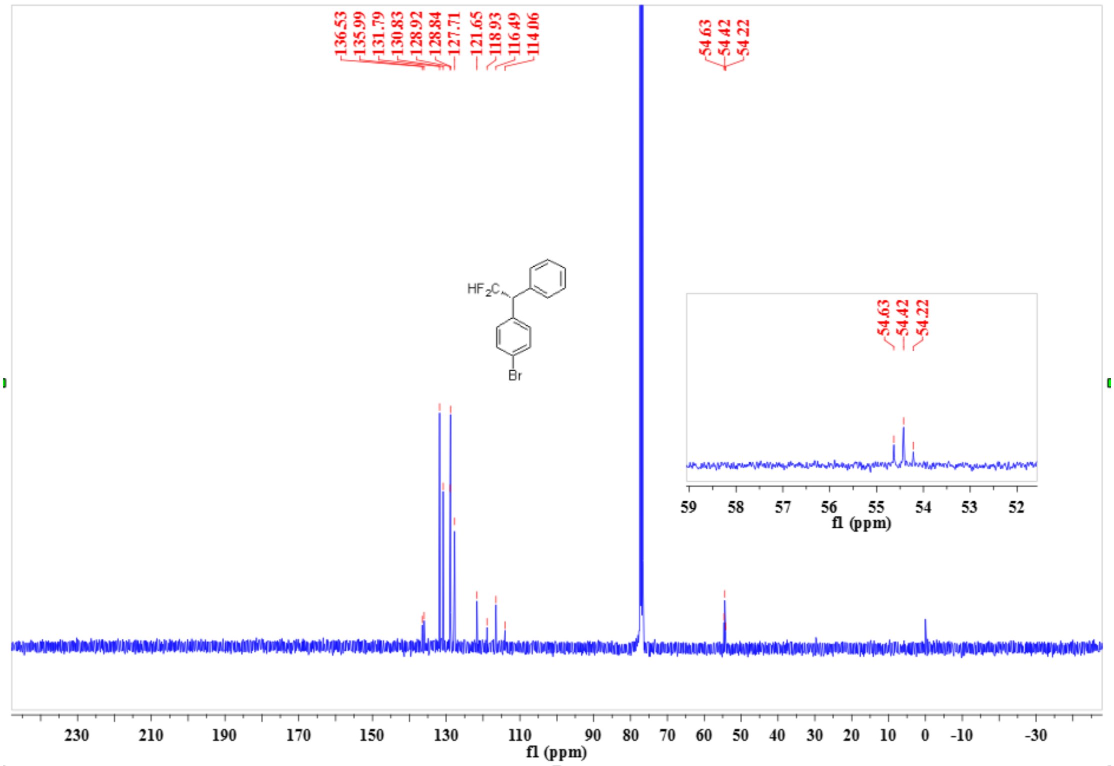


**Supplementary Figure 324.** $^{13}C$ **NMR (101 MHz,** $CDCl_3$**) spectrum of 4g**

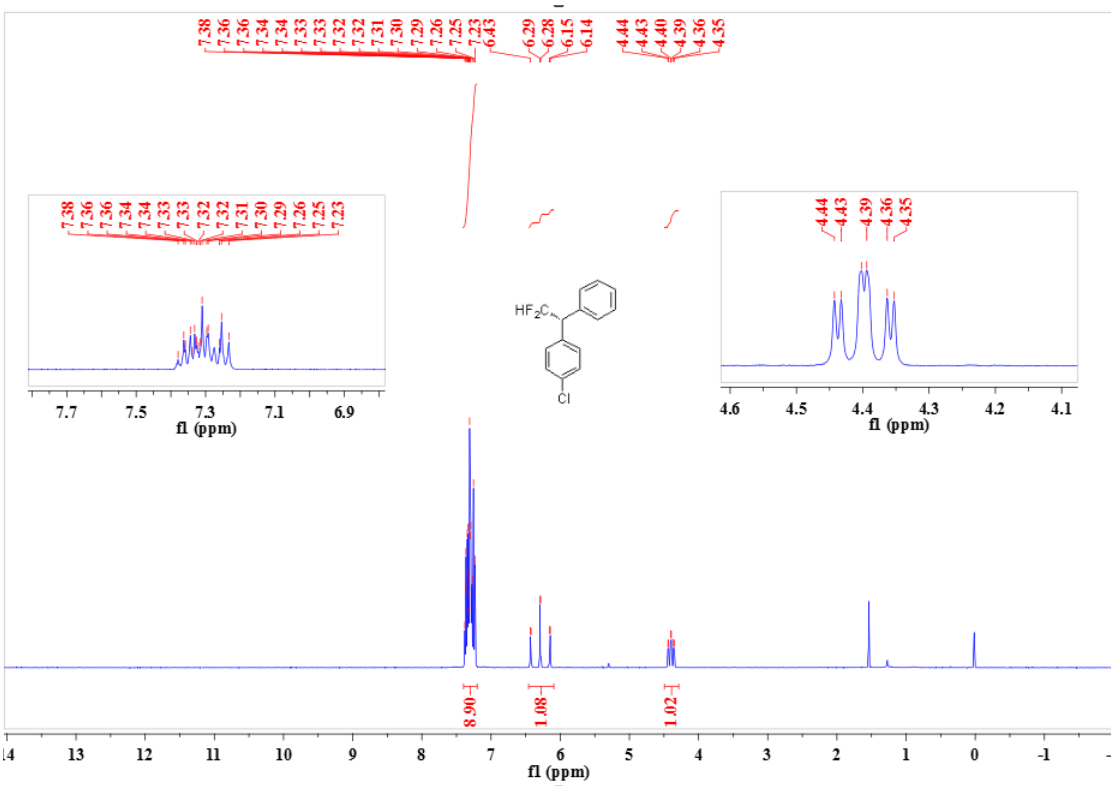

**Supplementary Figure 325.** $^1$H NMR (400 MHz, $CDCl_3$) spectrum of 4h

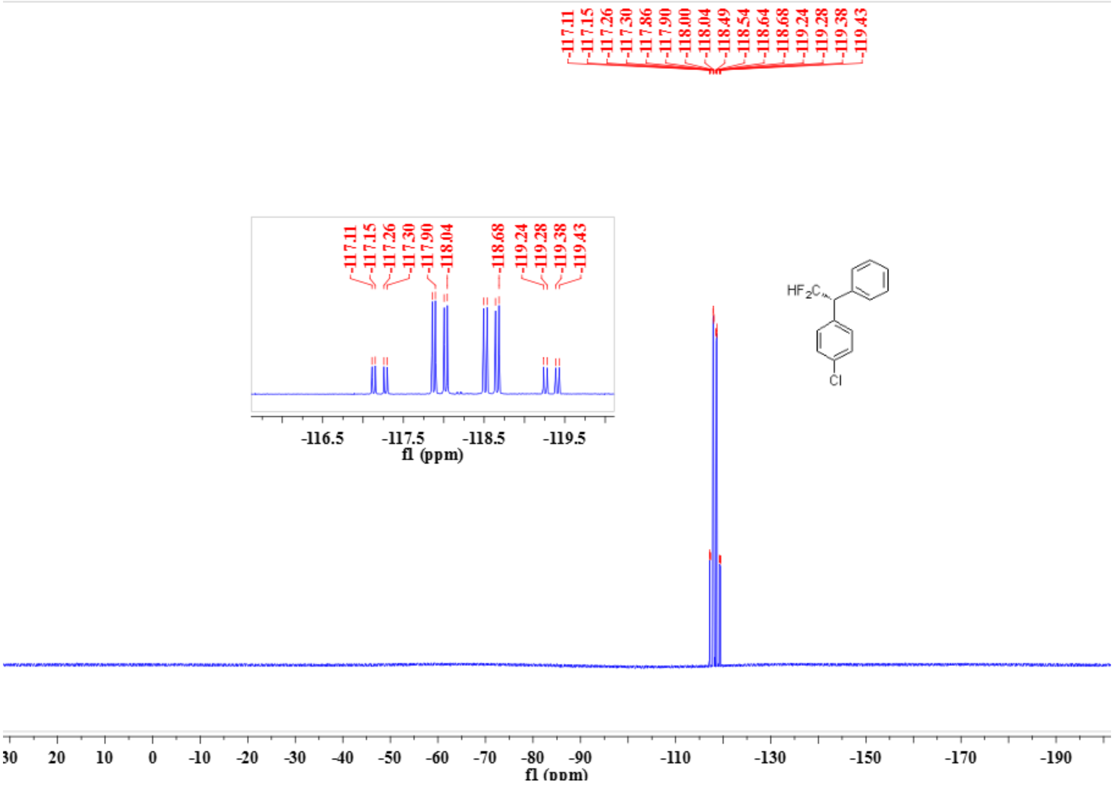

**Supplementary Figure 326.** $^{19}$F NMR (376 MHz, $CDCl_3$) spectrum of 4h

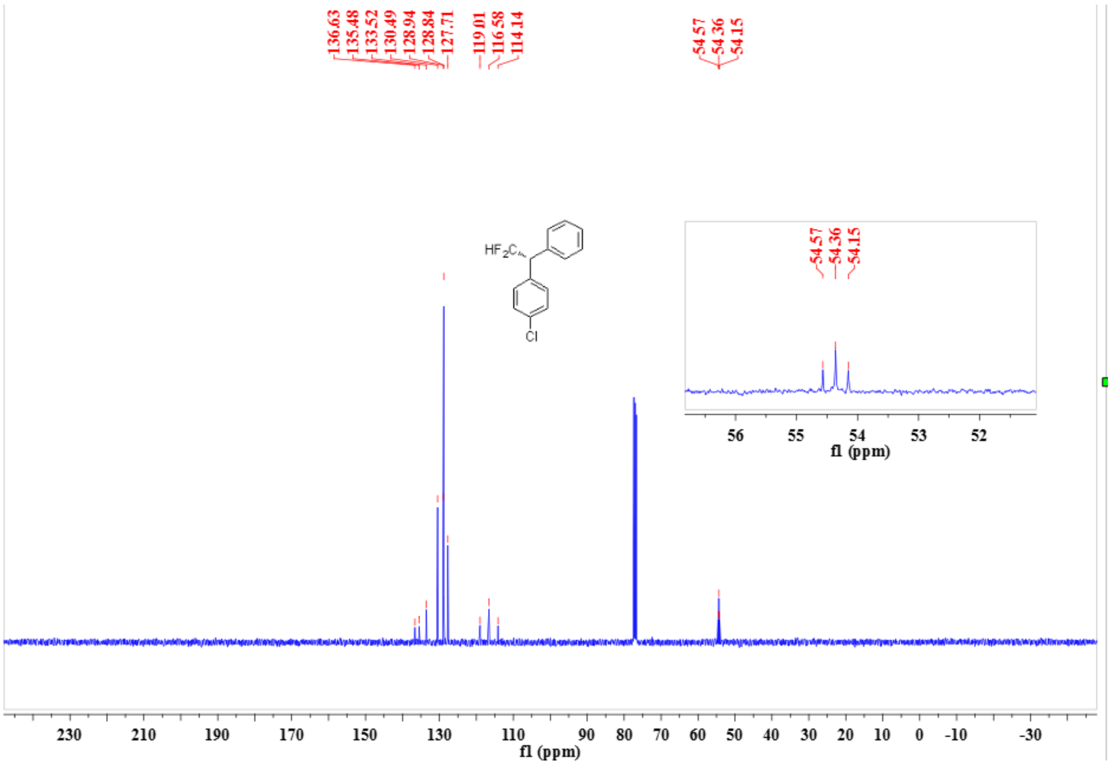

**Supplementary Figure 327.** $^{13}$C NMR (101 MHz, $CDCl_3$) spectrum of 4h

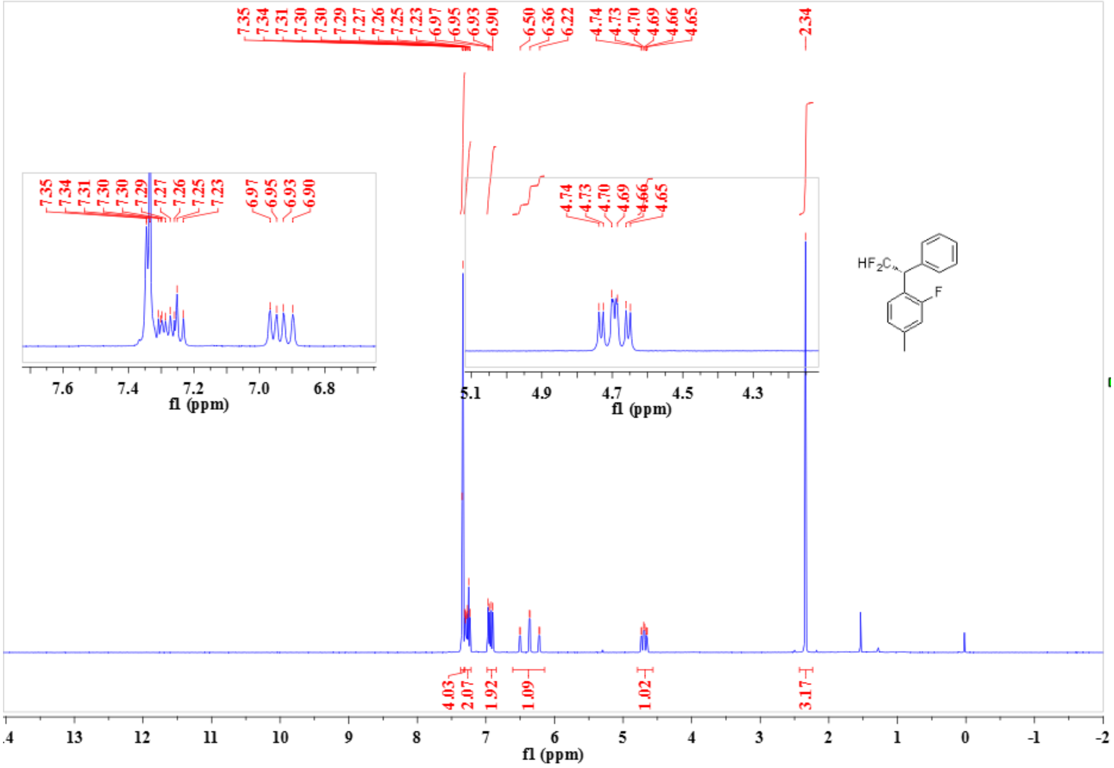

**Supplementary Figure 328.** $^{1}$H NMR (400 MHz, $CDCl_3$) spectrum of 4i

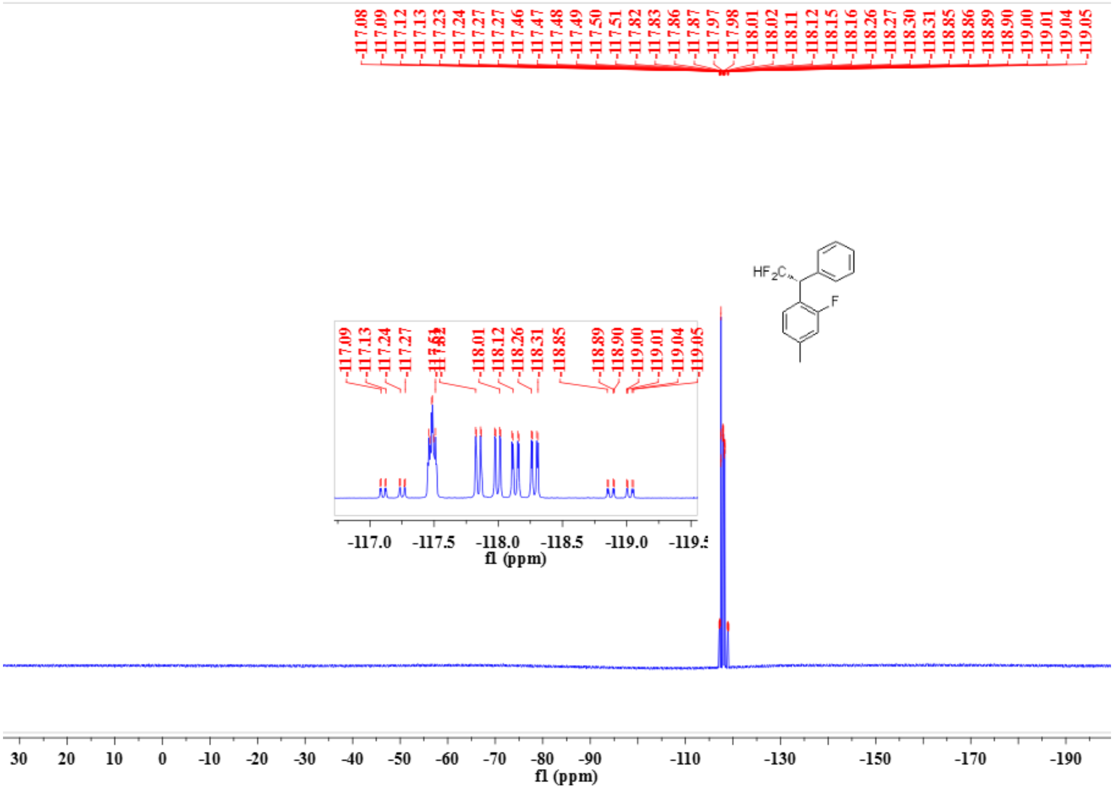

Supplementary Figure 329. $^{19}$F NMR (376 MHz, $CDCl_3$) spectrum of 4i

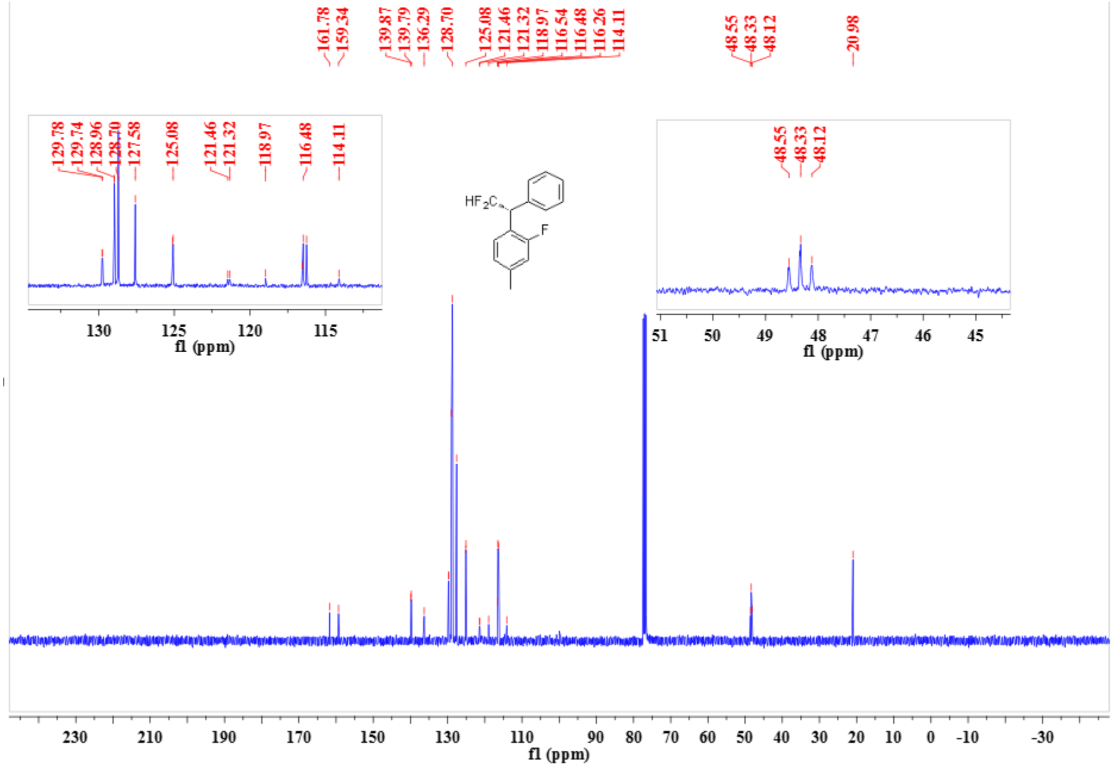

Supplementary Figure 330. $^{13}$C NMR (101 MHz, $CDCl_3$) spectrum of 4i

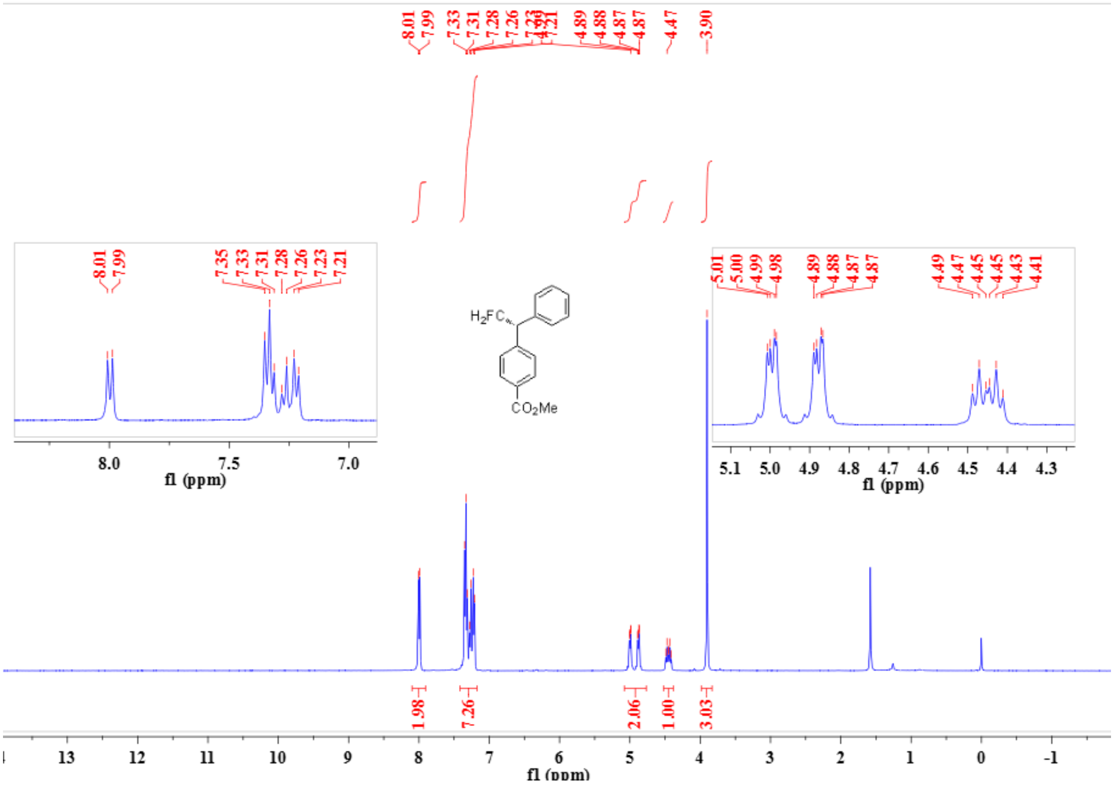

Supplementary Figure 331. $^{1}H$ NMR (400 MHz, $CDCl_3$) spectrum of 4j

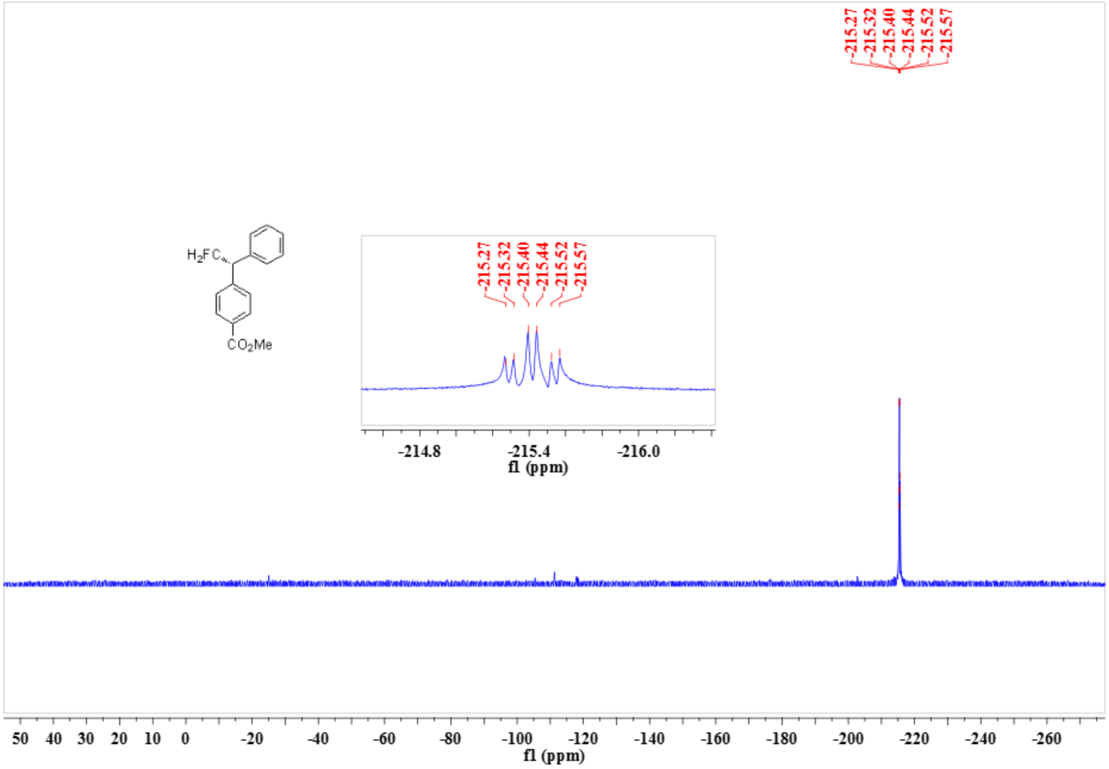

Supplementary Figure 332. $^{19}F$ NMR (376 MHz, $CDCl_3$) spectrum of 4j

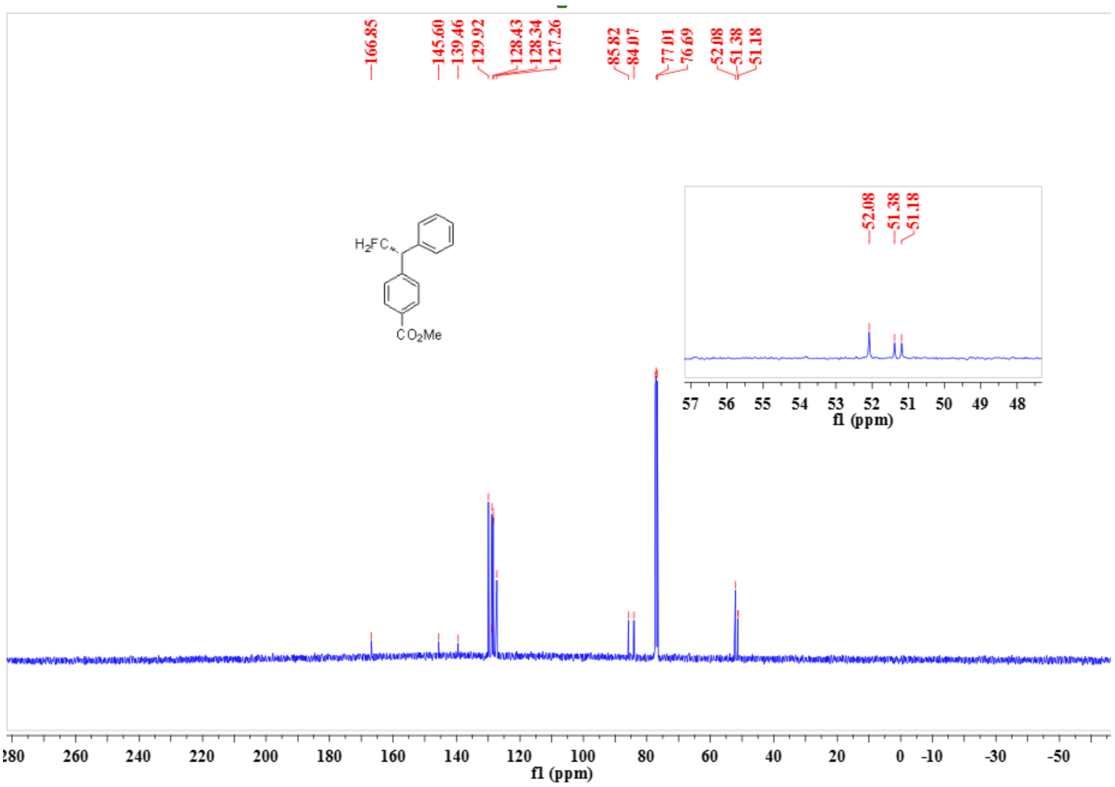

Supplementary Figure 333. $^{13}C$ NMR (101 MHz, $CDCl_3$) spectrum of 4j

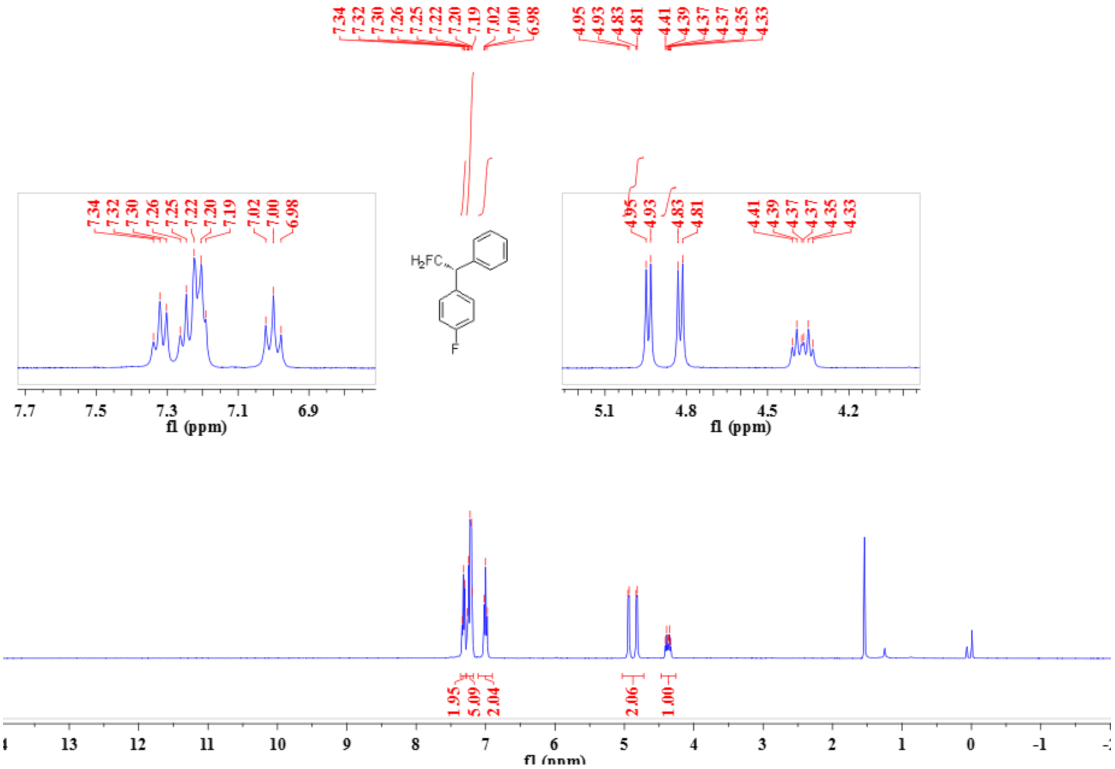

Supplementary Figure 334. $^{1}H$ NMR (400 MHz, $CDCl_3$) spectrum of 4k

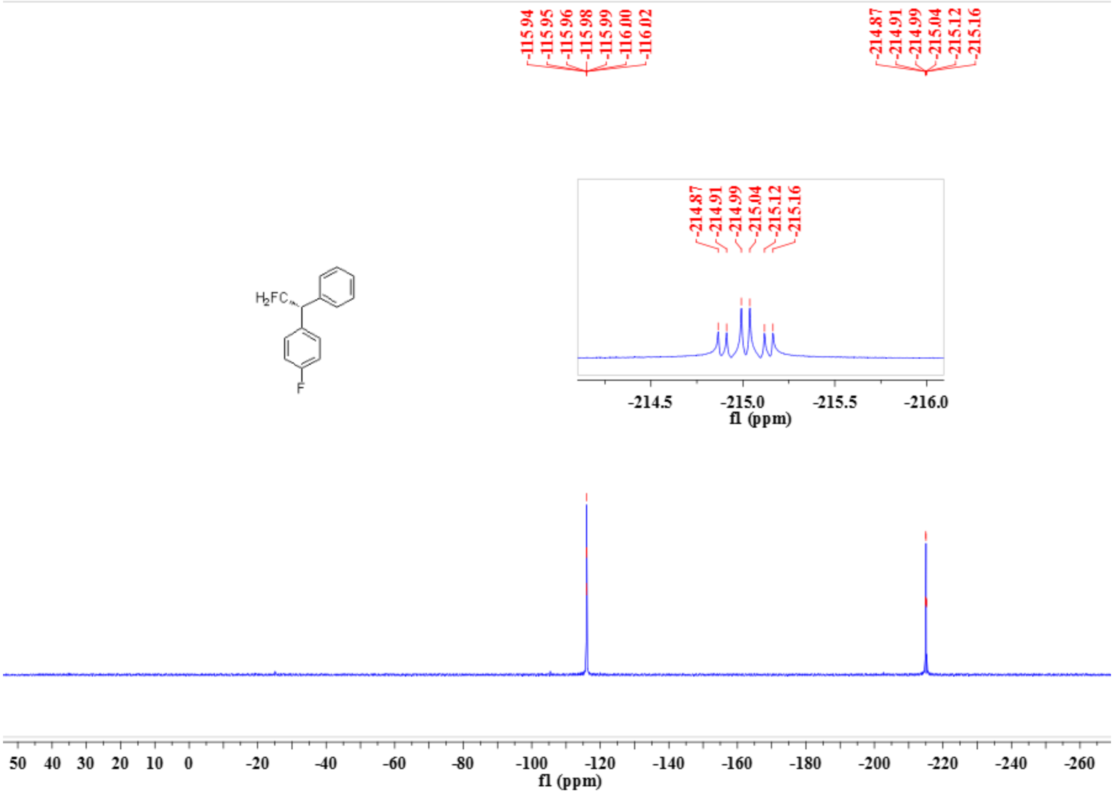

**Supplementary Figure 335.** $^{19}$F NMR (376 MHz, $CDCl_3$) spectrum of 4k

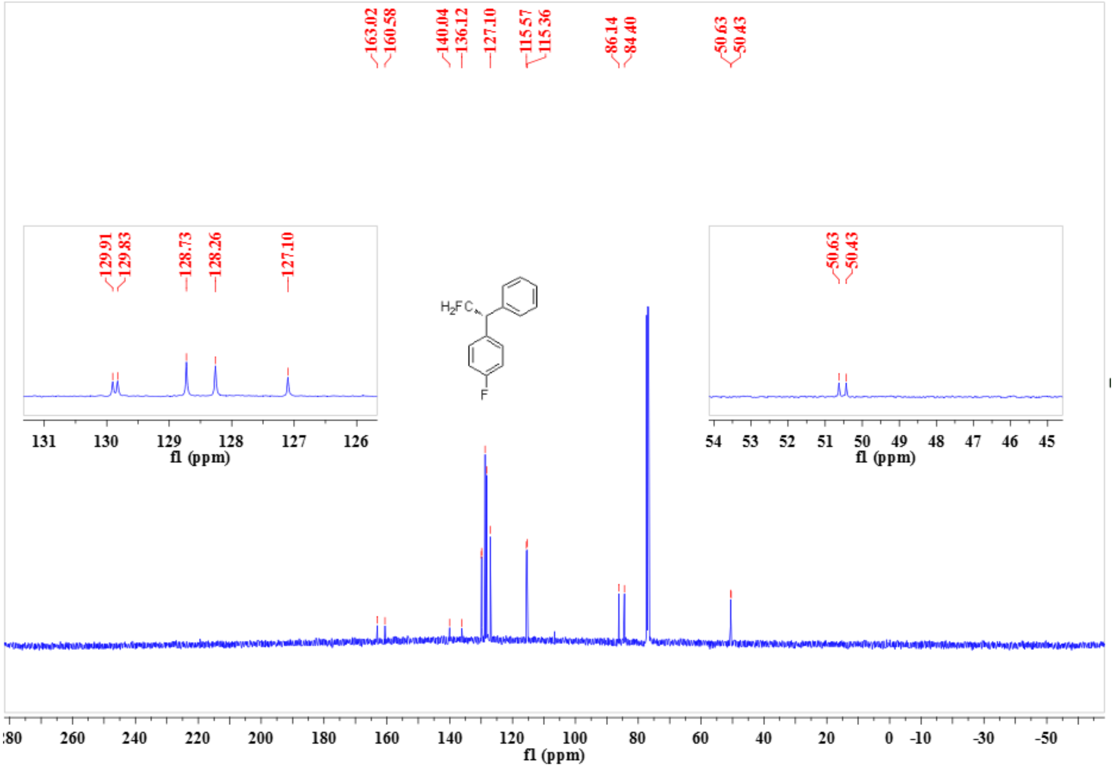

**Supplementary Figure 336.** $^{13}$C NMR (101 MHz, $CDCl_3$) spectrum of 4k

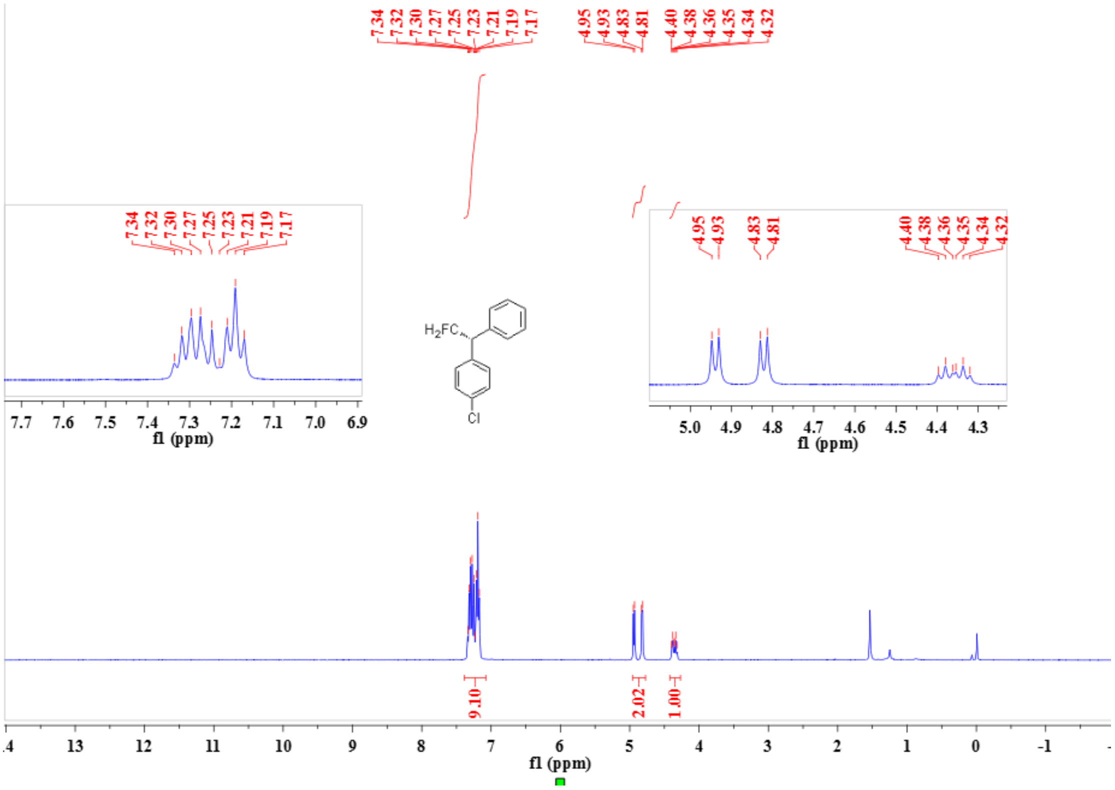

Supplementary Figure 337. $^{1}H$ NMR (400 MHz, $CDCl_3$) spectrum of 4l

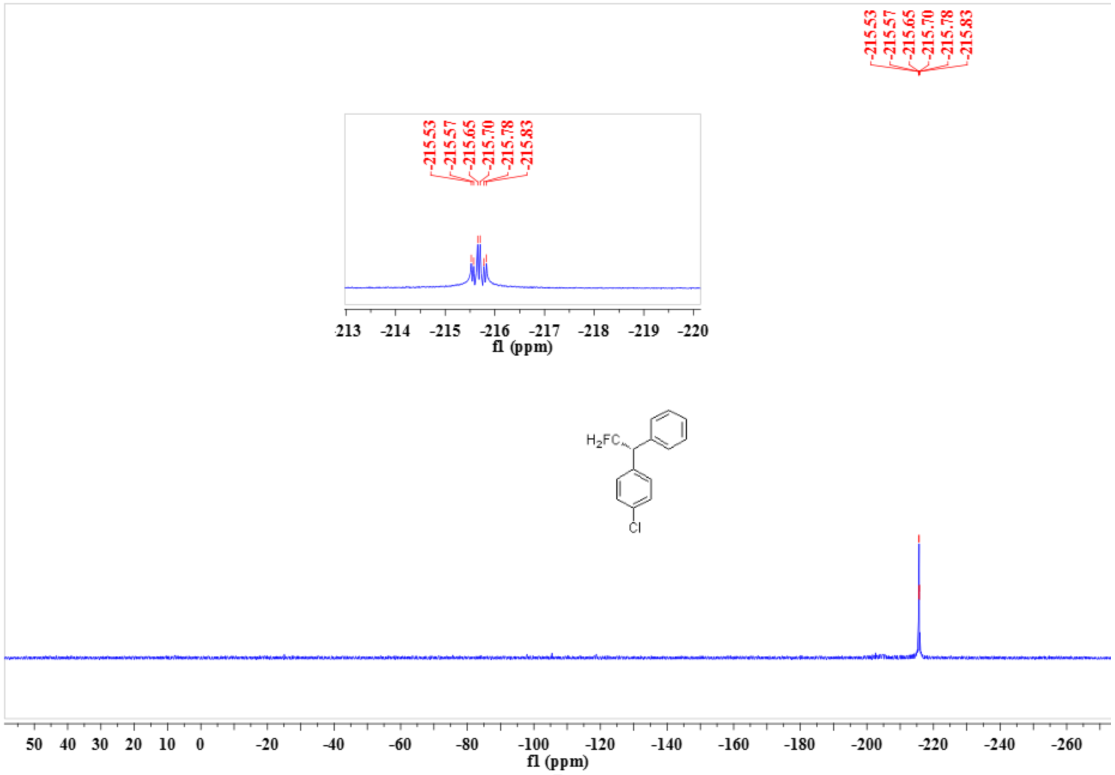

Supplementary Figure 338. $^{19}F$ NMR (376 MHz, $CDCl_3$) spectrum of 4l

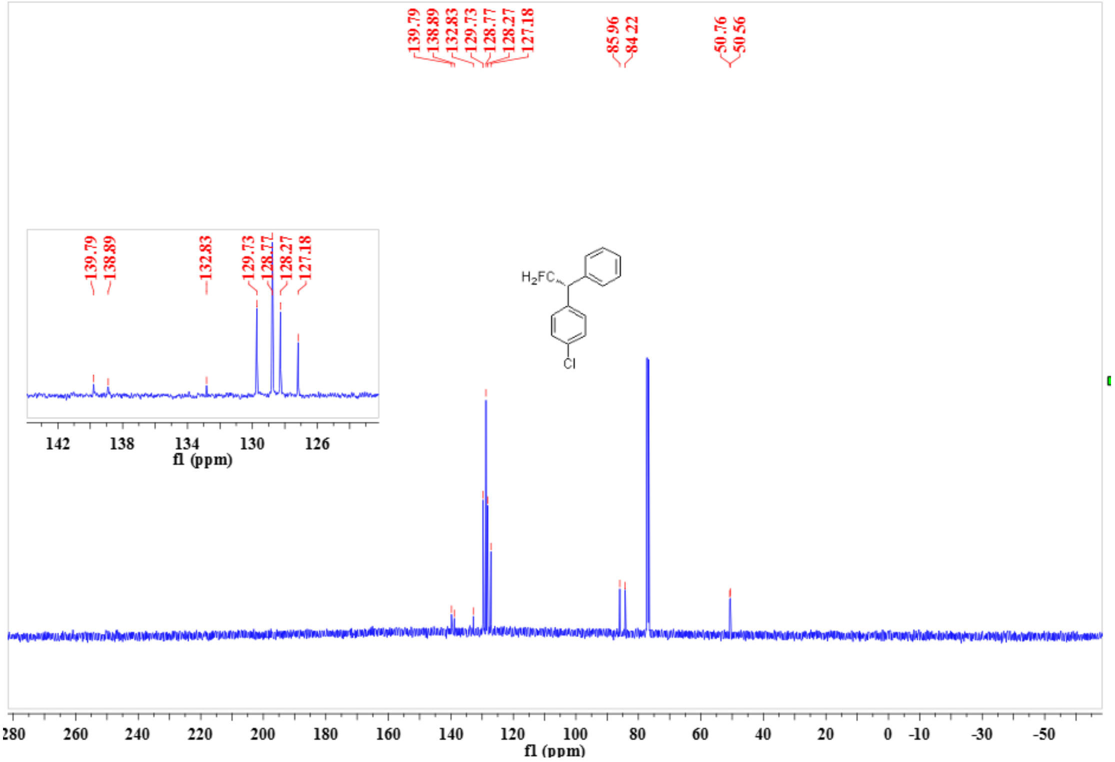


**Supplementary Figure 339.** $^{13}C$ **NMR (101 MHz,** $CDCl_3$**) spectrum of 4l**

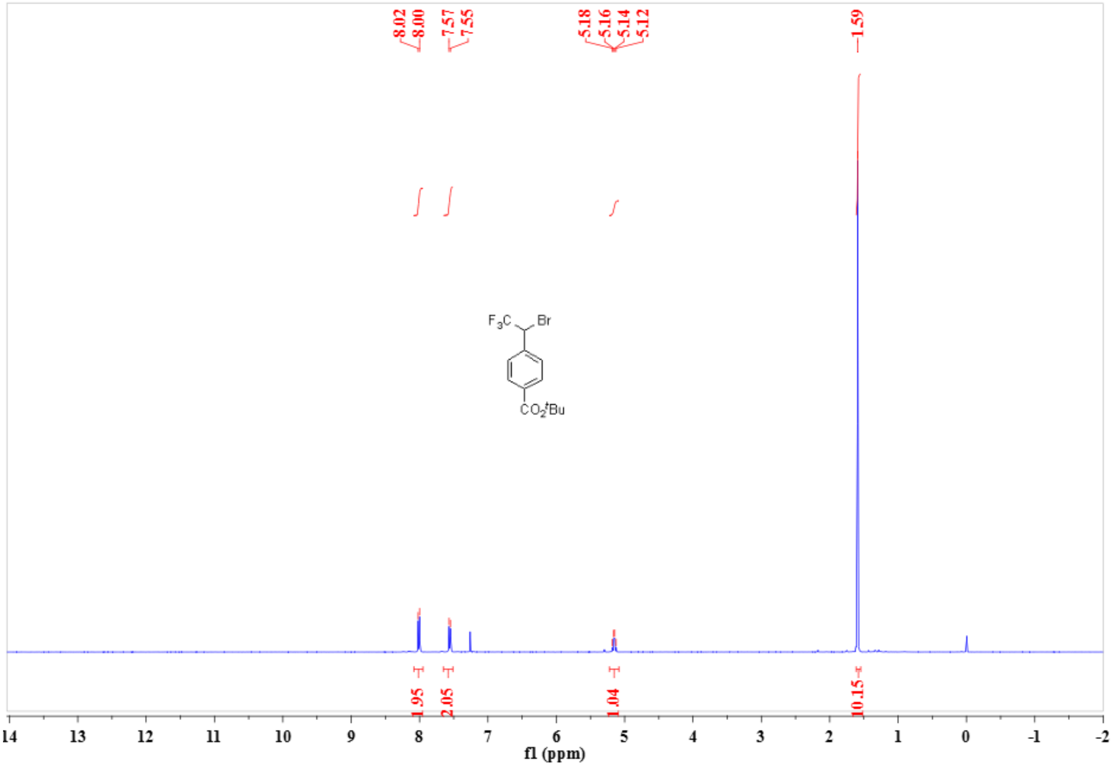


**Supplementary Figure 340.** $^{1}H$ **NMR (400 MHz,** $CDCl_3$**) spectrum of 1ae**

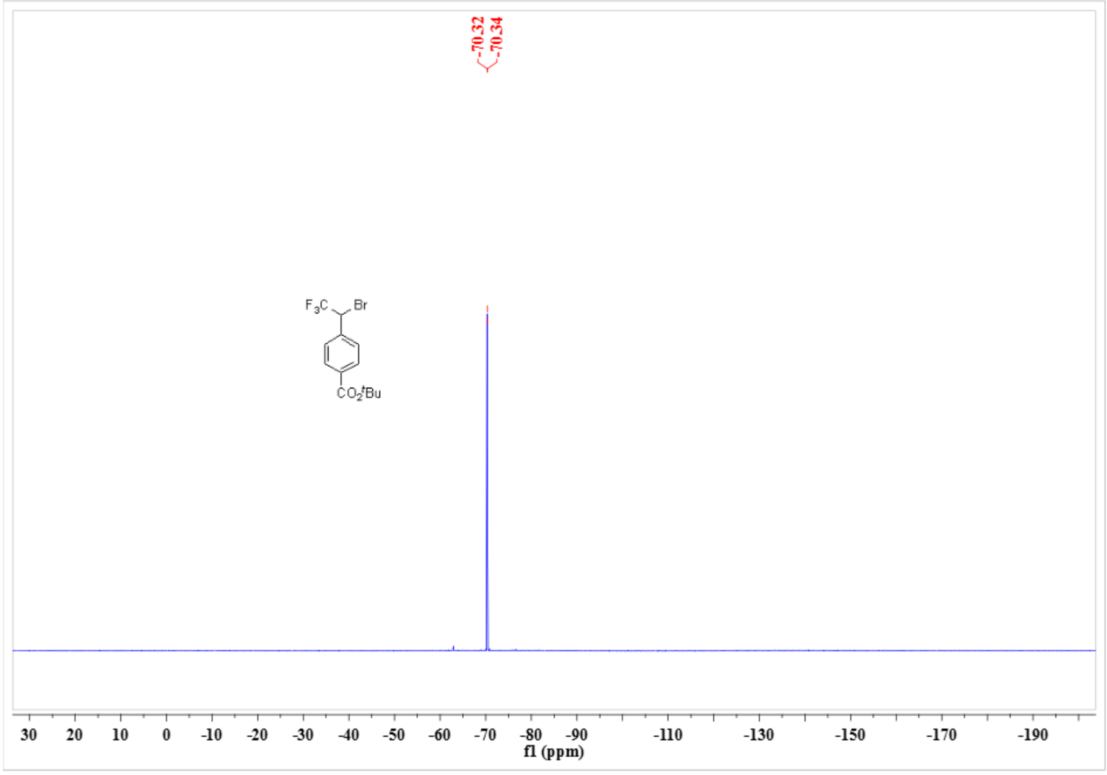


**Supplementary Figure 341.** $^{19}$**F NMR (376 MHz, CDCl$_3$) spectrum of 1ae**

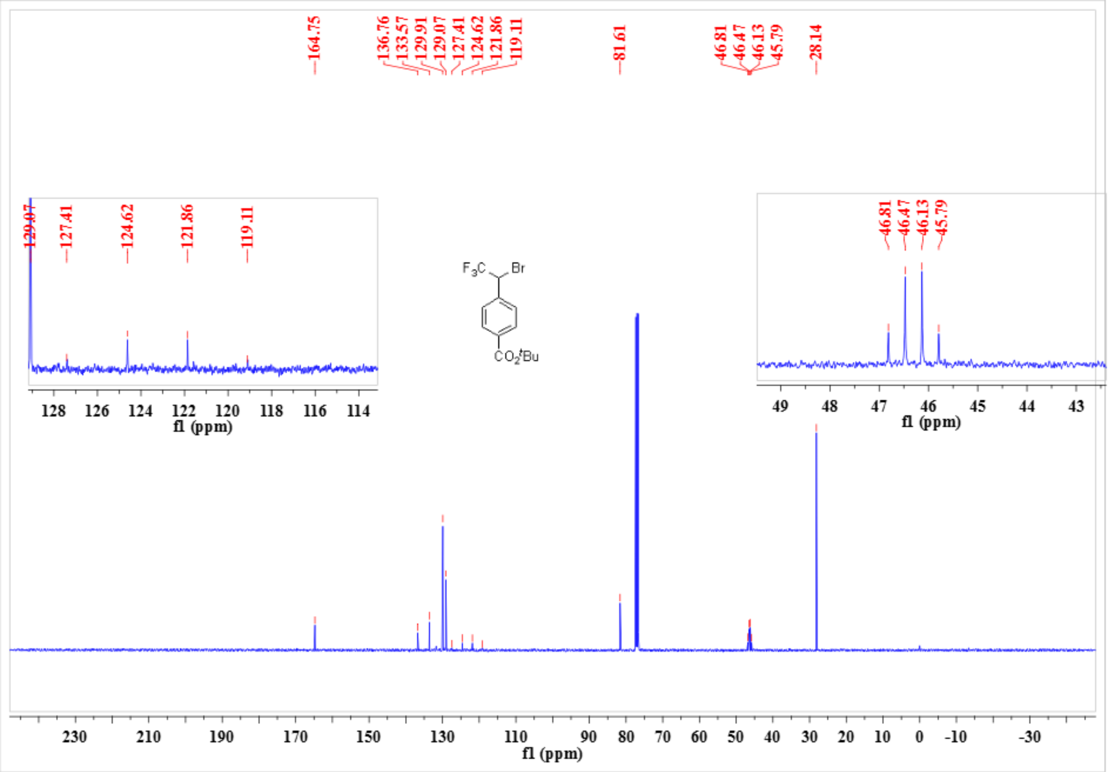


**Supplementary Figure 342.** $^{13}$**C NMR (101 MHz, CDCl$_3$) spectrum of 1ae**

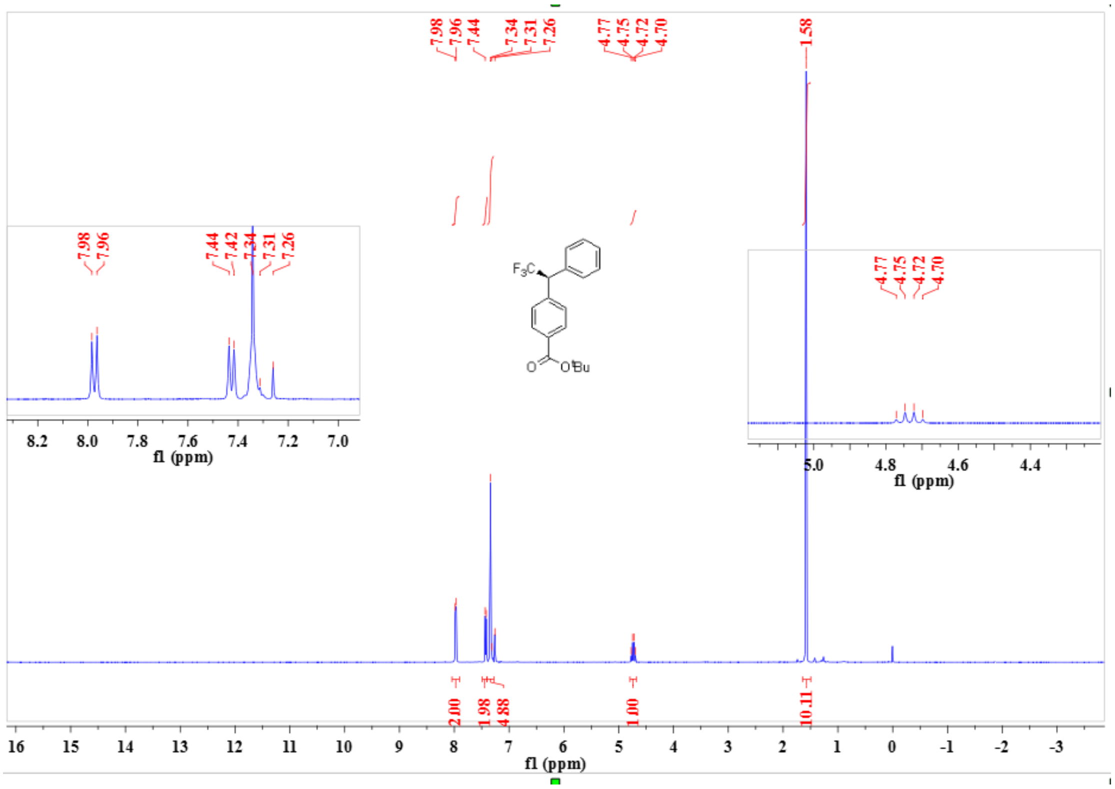

Supplementary Figure 343. $^1$H NMR (400 MHz, $CDCl_3$) spectrum of 5a

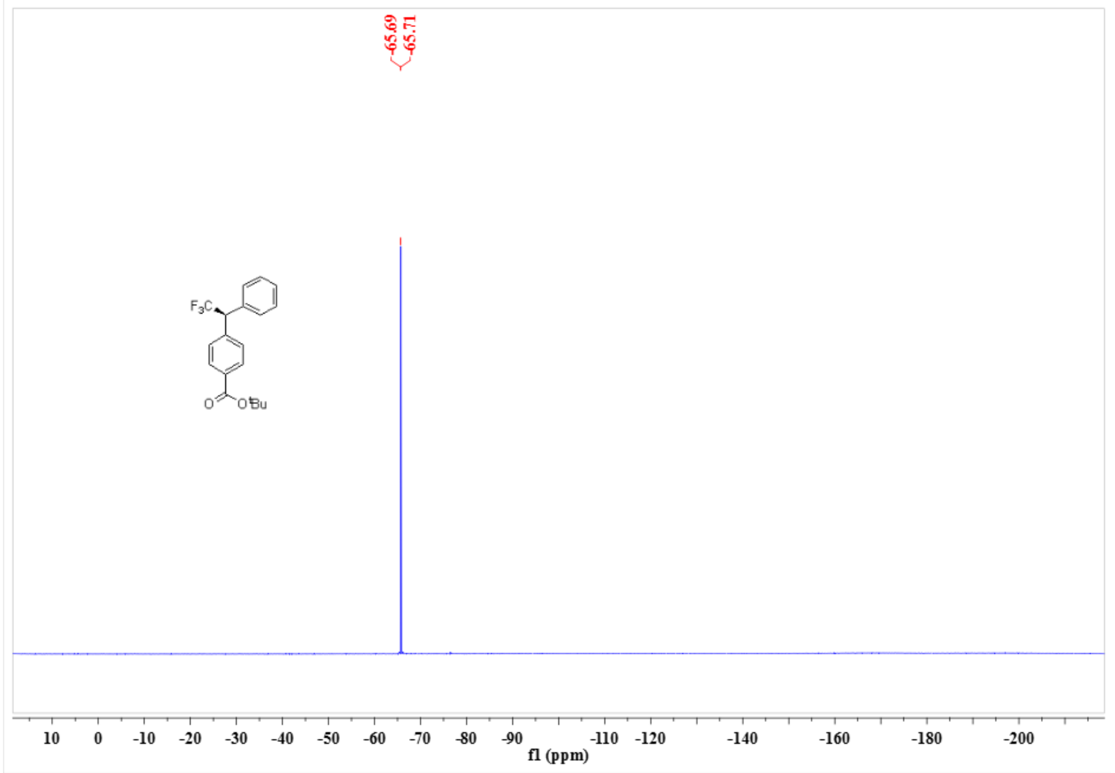

Supplementary Figure 344. $^{19}$F NMR (376 MHz, $CDCl_3$) spectrum of 5a

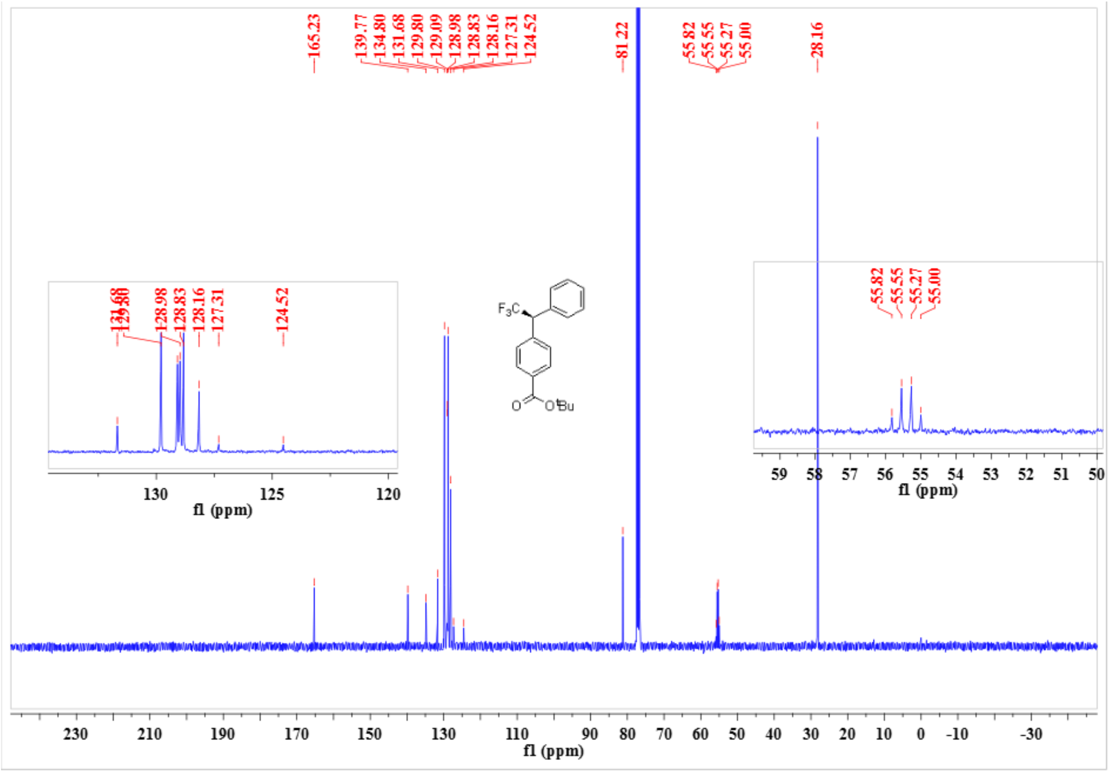

**Supplementary Figure 345.** $^{13}C$ **NMR (101 MHz, CDCl₃) spectrum of 5a**

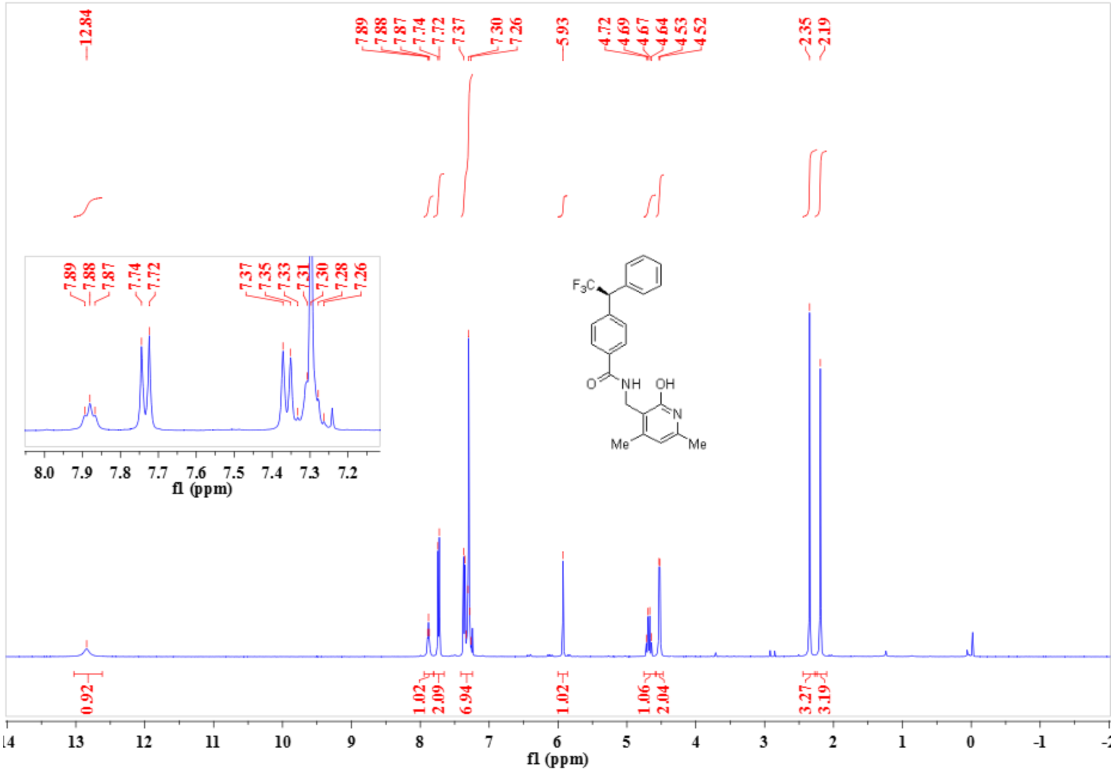

**Supplementary Figure 346.** $^{1}H$ **NMR (400 MHz, CDCl₃) spectrum of 5**

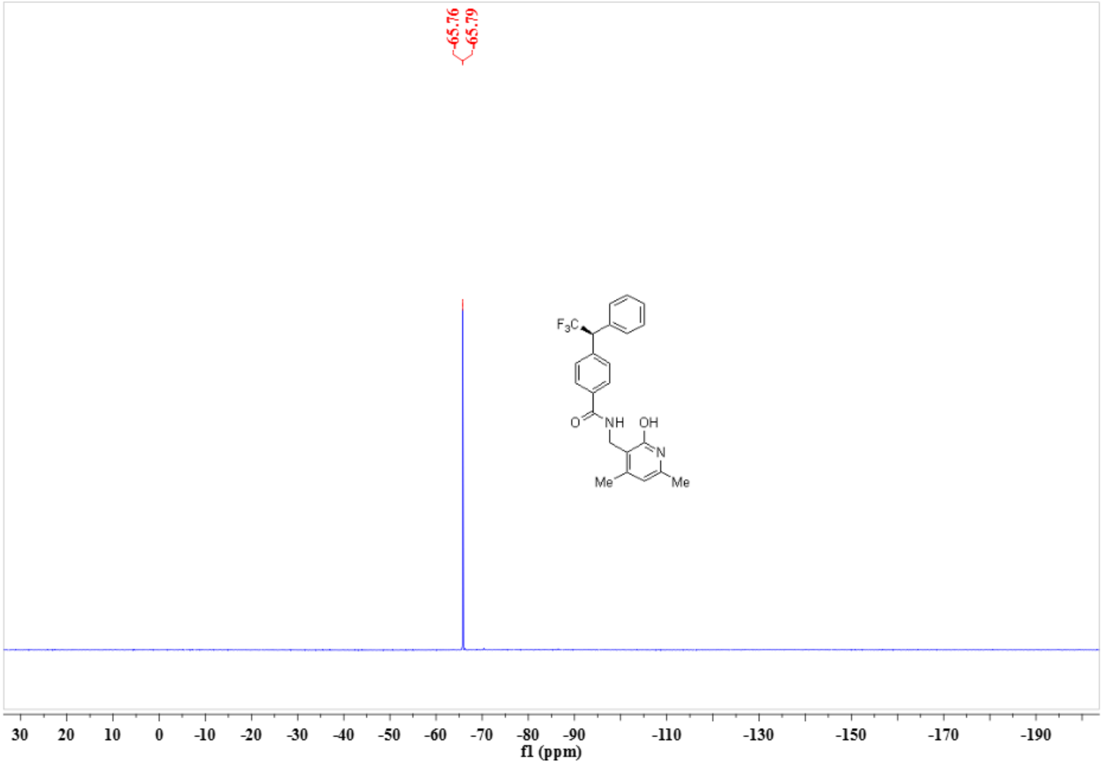

**Supplementary Figure 347.** $^{19}$**F NMR (376 MHz, $CDCl_3$) spectrum of 5**

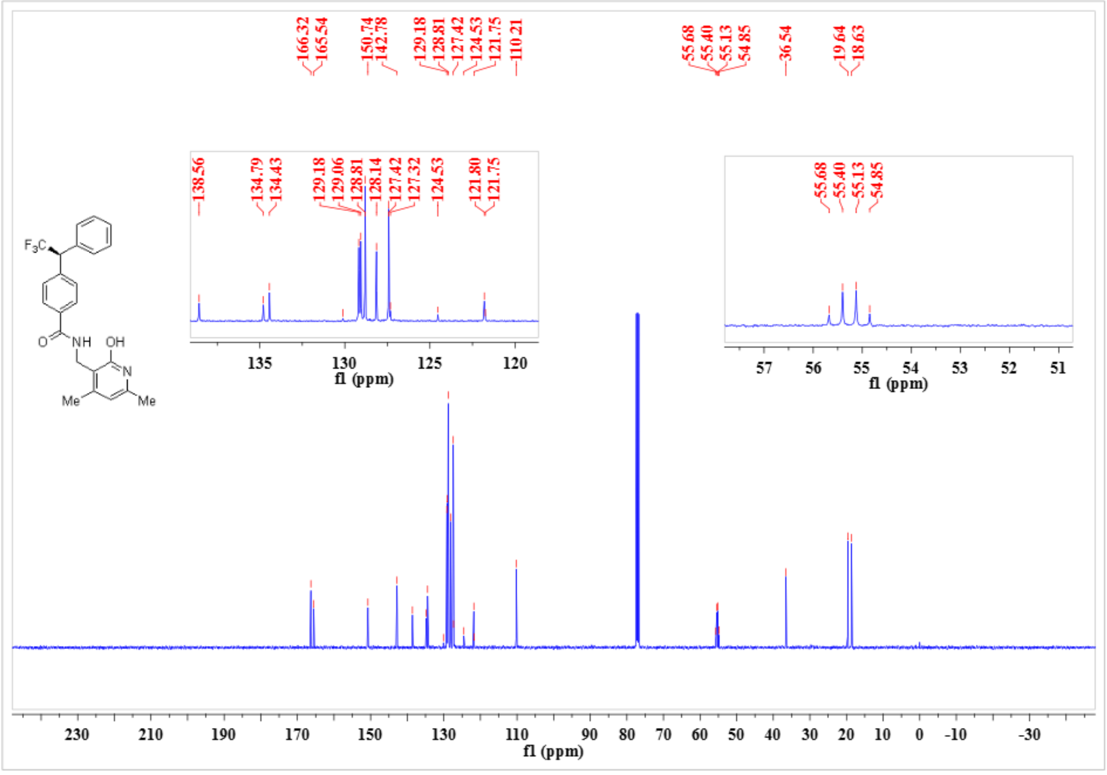

**Supplementary Figure 348.** $^{13}$**C NMR (101 MHz, $CDCl_3$) spectrum of 5**

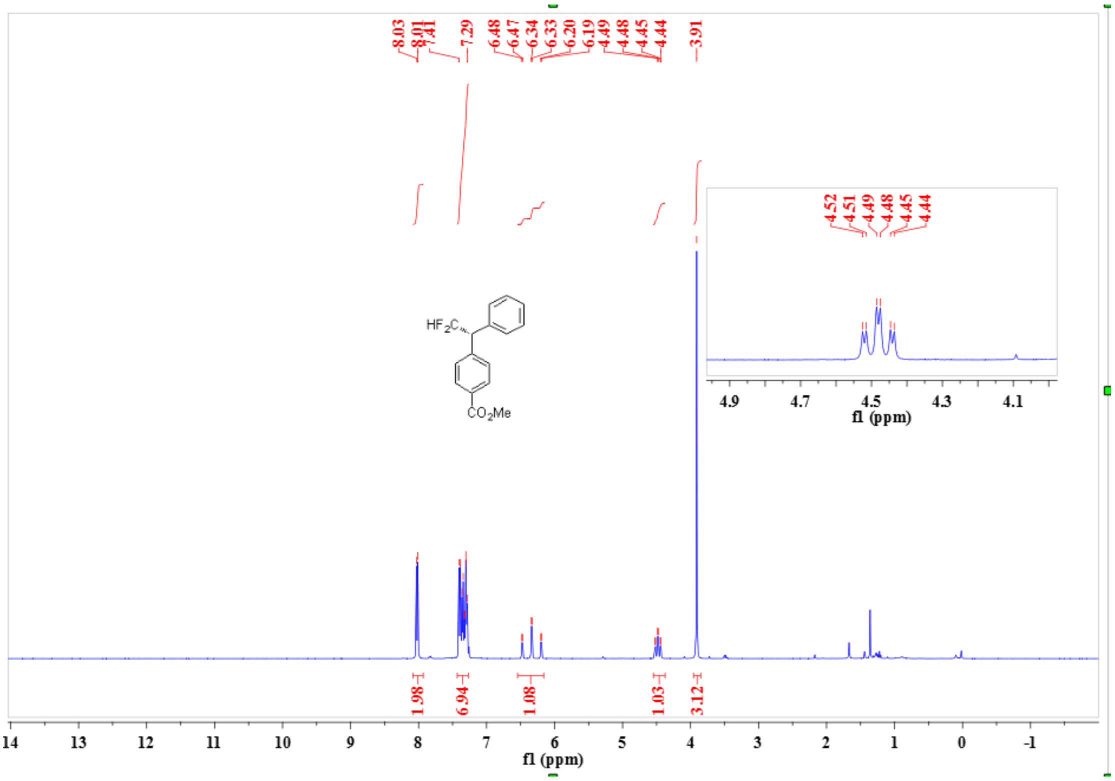

**Supplementary Figure 349.** $^{1}H$ **NMR (400 MHz, CDCl$_3$) spectrum of 6a**

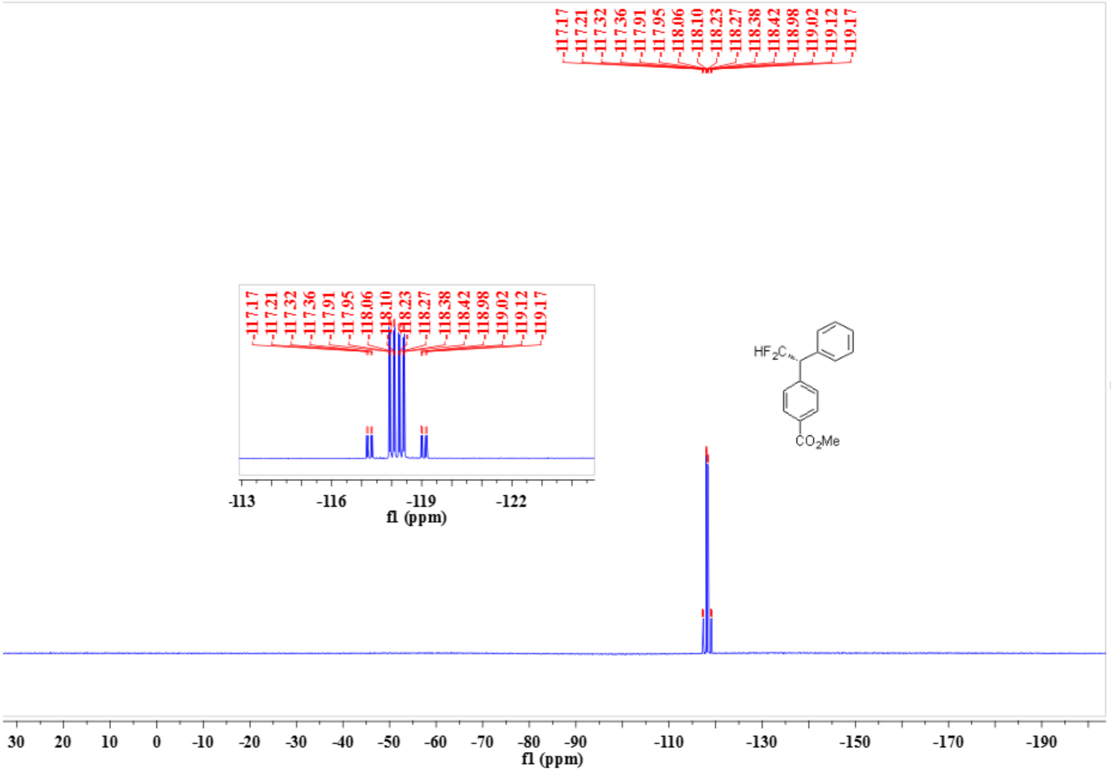

**Supplementary Figure 350.** $^{19}F$ **NMR (376 MHz, CDCl$_3$) spectrum of 6a**

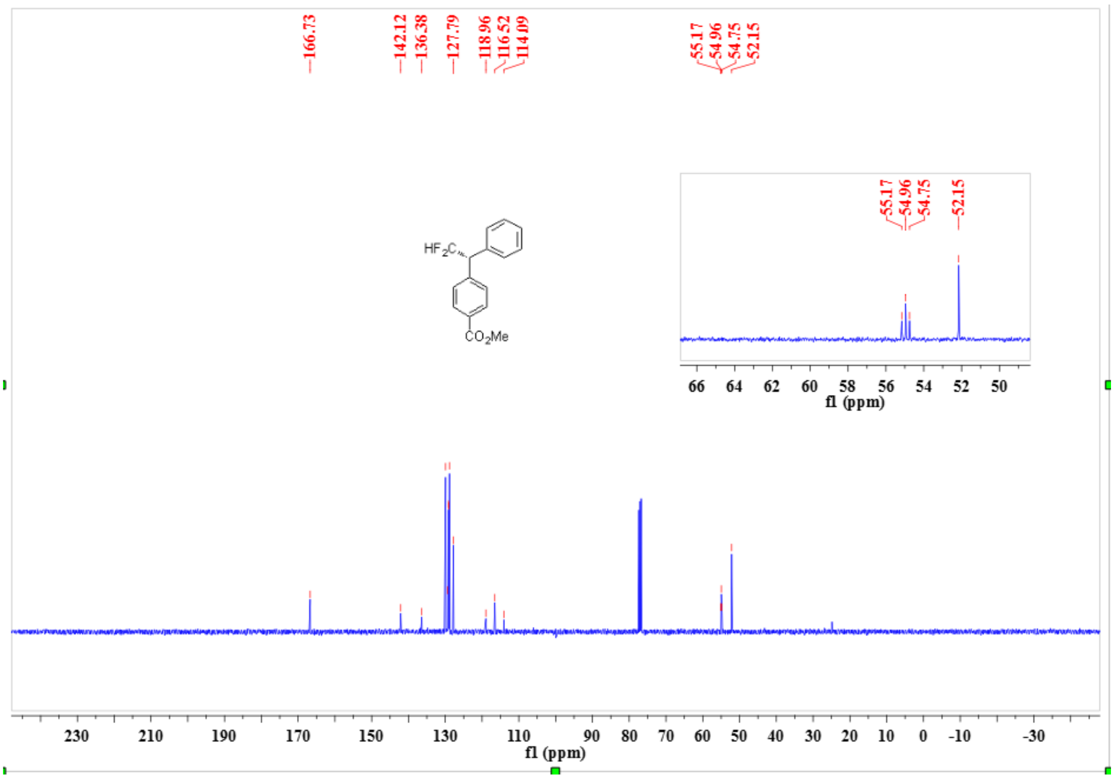

Supplementary Figure 351. $^{13}C$ NMR (101 MHz, $CDCl_3$) spectrum of 6a

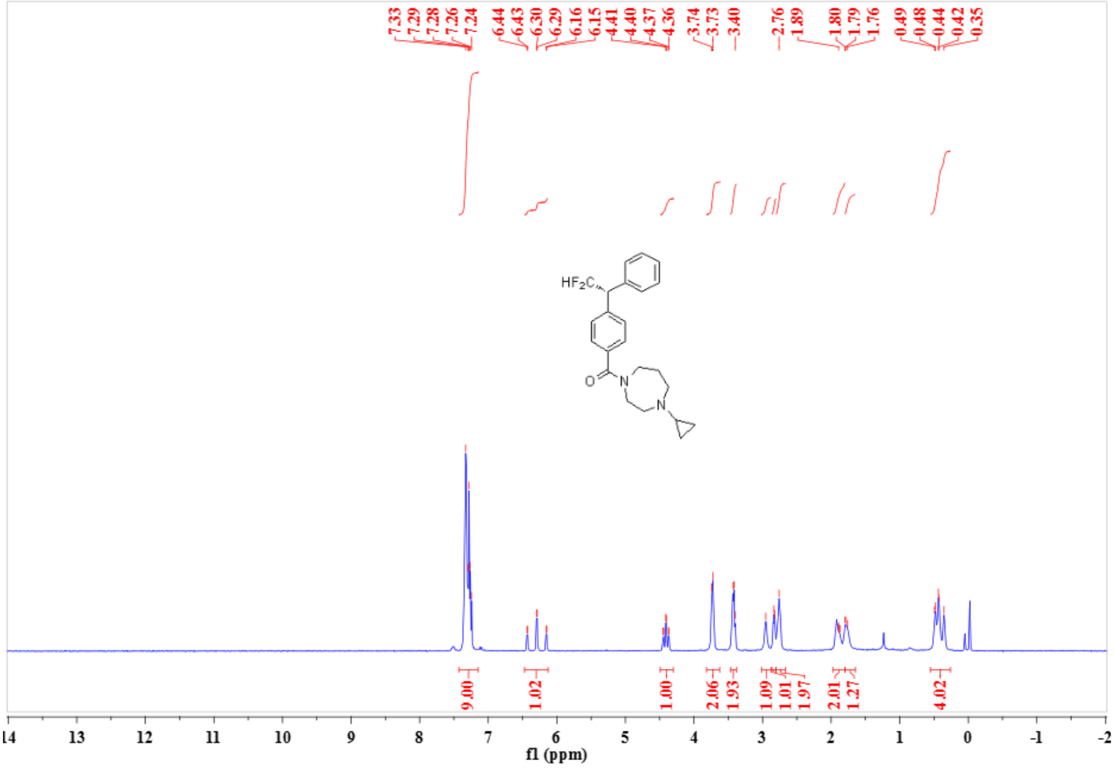

Supplementary Figure 352. $^{1}H$ NMR (400 MHz, $CDCl_3$) spectrum of 6

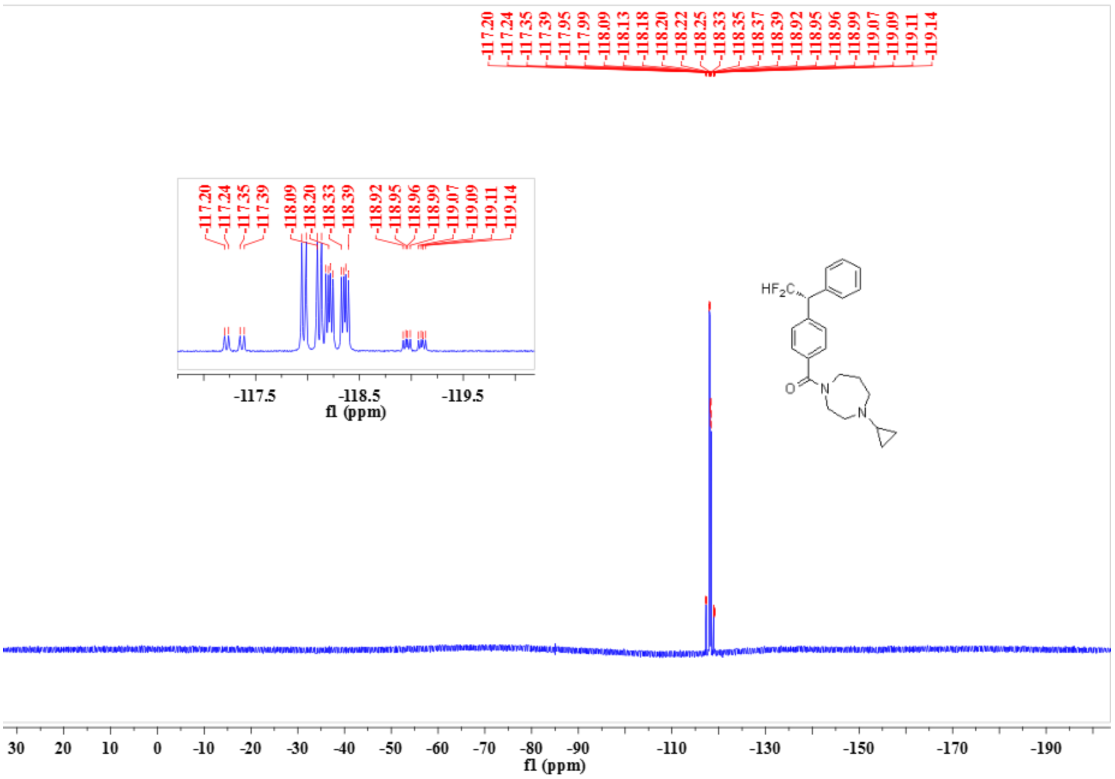

**Supplementary Figure 353.** $^{19}$**F NMR (376 MHz, CDCl$_3$) spectrum of 6**

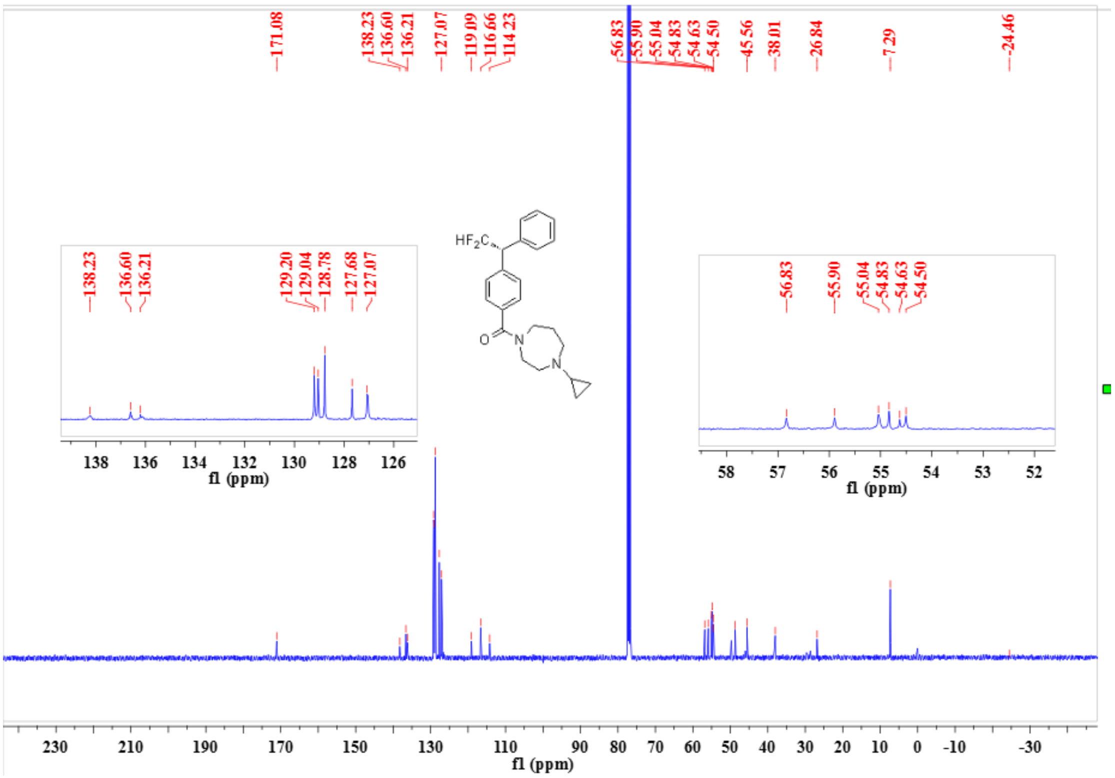

**Supplementary Figure 354.** $^{13}$**C NMR (101 MHz, CDCl$_3$) spectrum of 6**

**Supplementary References**

[1] Zhao, Y.-C.; Huang, W.-Z.; Zheng, J. & Hu, J.-B. Efficient and Direct Nucleophilic Difluoromethylation of Carbonyl Compounds and Imines with $Me_3SiCF_2H$ at Ambient or Low Temperature. *Org. Lett.* **13**, 5342 (2011).

[2] Peng, H,-H; Yuan, Z.-L.; Wang, H.-Y.; Guo, Y.-L. & Liu, G.-S. Palladium-catalyzed intermolecular fluoroesterification of styrenes: exploration and mechanistic insight. *Chem. Sci.,* **4**, 3172 (2013).
