# Supplementary material for: Facilitating the transmetalation step with aryl-zincates in nickel-catalyzed enantioselective arylation of secondary benzylic halides
Source: Nat Commun. 2019 Jul 4;10:2963. doi: 10.1038/s41467-019-10851-4 (PMC6609707; doi:10.1038/s41467-019-10851-4)
Supplement: Supplementary file 2 — Peer Review File [file 41467_2019_10851_MOESM2_ESM.pdf]

Reviewers' comments:

Reviewer #1 (Remarks to the Author):

This work details the nickel-catalyzed enantioselective arylation of secondary benzylic halides bearing fluoroalkyl substituents. The work is important, because it employs air stable boronic acids as the nucleophile and is able to generate stereogenic centers from racemic materials bearing important fluoroalkyl groups.

While the overall reactions worked out by the authors are very nice, I do have some concerns about the mechanistic hypothesis. For instance, the authors do not seem to consider that $ZnBr_2$ could simply abstract a halide from generated nickel halide complexes - thus facilitating a transmetalation step. Precedence is known for such chemistry, see: Organometallics 2004, 23, 623.

That being said, I believe the statements "clearly suggest anionic zincate [Ph_2ZnBr]Li would facilitate the transmetalation step" and "Having identified the role of $ZnBr_2$" may be premature.

I think the authors should consider this possibility, and disclose their thoughts/experiments on the matter before publication.

Minor points:

1) "Transmetalation" is spelled two ways throughout the text.

2) Figure 1 is not referred to in the text of the document. Also, figure 1 doesnt really do justice to related chemistry that is known, i.e. JACS 2015, 137, 9523–9526.

3) I believe for the examples provided, "racemic" is the proper terminology, rather than "prochiral" when referring to the organic halides. This should be corrected.

4) Line 139: should read "might be involved"

Reviewer #2 (Remarks to the Author):

Shen and co-workers report the extension of their enantioselective coupling of racemic substituted benzyl halides where the FG is now CF3/CHF2/CFH2, instead of OCF3 (as documented in their previous work - Angew.Chem.2017,129,12148). Notably, in this work the coupling was ineffective using their previously identified conditions and only was effective using combinations of the [nBu(Ph)BPin]- borate and ZnBr2 which generates zincates in-situ. They then use this procedure to generate an impressive selection of CF3 and CF2H/CFH2 substituted cross-coupled products all with good to excellent e.r. values. Due to the importance of these compounds, the broad scope and the high e.r values observed I believe this paper is suitable for publication in Nat. Comm once the following minor points have been addressed.

Comments:

Are L2 ligated Zinc species able to function as nucleophiles fro cross coupling in the absence of the Ni salt? If the authors have not tried the coupling with stoichiometric ZnBr2 in the absence of Ni salts they must do so.

In the reaction between 3 eq. of 2a and ZnBr2 what is observed in the 11B NMR spectrum? Are both the borate 2a and the expected by-product BuBPin observed? i.e. is the 11B NMR spectrum also consistent with incomplete consumption of 2a, so formation of mixed arly/halo zincates?

Can the ligand/catalyst loading be dropped at all from 20/25 mol%?

Minor comments:

Scheme 1 / footnotes contain no info. on amount of Ni / L, these details should also be shown in the footnote/scheme.

Reviewer #3 (Remarks to the Author):

In this work Shen and co-workers describe C(sp3)-C(sp2) asymmetric Suzuki-Miyaura cross-coupling reaction of fluoroalkyl substituted benzylic bromides with aryl borates. The reaction leads to families of interesting enantioenriched compounds, bearing fluoroalkyl group at the stereogenic center, which are of potential importance to drugs and agrichemicals discovery. The key point is a finding that an addition of ZnBr2 is necessary to enable the reactivity, and an assumption that diaryl zincates [Ar2ZnBr]Li represent a true active trans-metallating spices is made. Since this is a first catalytic asymmetric approach to prepare these interesting compounds, I would recommend a publication of this manuscript in Nat. Commun. However, many questions should be addressed prior to publication:

(1) Quite high catalyst loading (20mol% of Ni salt and 25mol% of the ligand) is used. Why? What happens with a lower loading? This data should be provided in the optimization table – Scheme 1.

(2) Same about Ar-borates: 3(!) equivalents are used. What is the reason? What happens with a lower loading?

(3) All examples, given throughout the text, are based on the benzylic bromides bearing only electron-withdrawing substituents on the aromatic ring. This is definitely a limitation of the approach, which should be clearly stated in the text. If not, then the examples of benzylic electrophiles bearing electron donating substituents should be added.

(4) I have a similar claim regarding a scope of aryl borates: it includes only 6 examples. No examples of ortho- or/and para- substituted aryl borates are provided. Such examples would enrich the work, and should be given. Otherwise, it should be noted that such compounds are not suitable for this transformation.

(5) It is informally agreed in a catalytic chemical community, that ee's above 90% are called "high". In this work most of the ee's are around 88% (some are above, but many are significantly lower). Also, the yields of compounds are in the range of 50-70% - see Schemes 2-3. I would render from calling them "high yields and high enantioselectivity" as authors do throughout the text. Rather, I would modestly say that compounds are obtained in a moderate to good yields and enantioselectivity.

(6) Regarding the mechanistic studies, it is way far from informative. Authors claim that the active species are [Ar2ZnBr]Li, which could be probably prepared independently from Ar2Zn and LiBr. Is there any attempt to isolate these species? Are they known previously? What about their full NMR? XPS might be helpful to say something about Zn and its coordination sphere. All these questions should be addressed.

(7) If authors propose trans-metallation from boron to zinc, is it possible to say something about Keq of this thermodynamically unflavored process? Moreover, according to stoichiometry of this reaction two equiv. per one mole of ZnBr2 is enough. What happens with a third equiv. that is used in the procedure?

(8) Probably, a most important point: if [Ar2ZnBr]Li are truly active nucleophile in the reaction and can be prepared by Ar2Zn + LiBr, why should one use expensive organoboron precursors at all?? The paper would be much more elegant, if the series of Ar2Zn would be prepared and used as the

nucleophiles in the presence of LiBr. In my opinion, it is an important issue that should be done prior to publication.

Reviewer 1

This work details the nickel-catalyzed enantioselective arylation of secondary benzylic halides bearing fluoroalkyl substituents. ***The work is important****, because it employs air stable boronic acids as the nucleophile and is able to generate stereogenic centers from racemic materials bearing important fluoroalkyl groups.*

While the overall reactions worked out by the authors are very nice, I do have some concerns about the mechanistic hypothesis. For instance, the authors do not seem to consider that $ZnBr_2$ could simply abstract a halide from generated nickel halide complexes - thus facilitating a transmetalation step. Precedence is known for such chemistry, see: Organometallics 2004, 23, 623.

That being said, I believe the statements "clearly suggest anionic zincate [Ph2ZnBr]Li would facilitate the transmetalation step" and "Having identified the role of ZnBr2" may be premature.

I think the authors should consider this possibility, and disclose their thoughts/ experiments on the matter before publication.

We thank the reviewer for his constructive suggestion. The proposal of the formation of $[Ph_2ZnBr]Li$ and its role in acceleration the transmetalation step was based on the experimental observations that using the combination of Ph_2Zn + LiBr as the nucleophile gave comparable yield and enantioselectivity with that used lithium phenyl boronate **2a** +$ZnBr_2$ as the nucleophile. Previously, Ingleson and co-workers reported that mixing 2.0 equivalents of lithium aryl boronate with $ZnBr_2$ at room temperature generated an anionic $[Ph_xZnBr_y]^-$ (x + y = 3) (*Chem. Eur. J.*, **2017**, *23*, 15889). We repeated this experiment and confirmed these observations.

Anyway, we think that the reviewer is right that the statement "clearly suggest anionic zincate $[Ph_2ZnBr]Li$ would facilitate the transmetalation step" *and* "Having identified the role of $ZnBr_2$"

in the manuscript is premature. Consequently, we added a sentence in the manuscript, as shown below:

"At the meanwhile, we could not exclude an alternative pathway in which $ZnBr_2$ may abstract a bromide from nickel benzylic bromide complex to generate a cationic η^3-benzyl nickel species[32] that could facilitate the transmetalation process with lithium organoboronate **2a**."

A reference for the abstraction of halide from nickel benzylic bromide complex by ZnBr2 was added as ref. 33, as shown below:

(33) Anderson, T. J. & Vicic, D. A. Direct Observation of Noninnocent Reactivity of $ZnBr_2$ with Alkyl Halide Complexes of Nickel. Organometallics **23**, 623 (2004).

In addition, the sentence "These results clearly suggest anionic zincate $[Ph_2ZnBr]Li$ would facilitate the transmetalation step" was changed to "These results suggest anionic zincate $[Ph_2ZnBr]Li$ might facilitate the transmetalation step".

Furthermore, the phrase "Having identified the role of $ZnBr_2$" was changed to "Having identified the optimized conditions and the likelihood role of $ZnBr_2$".

"Transmetalation" is spelled two ways throughout the text.

We changed "transmetallation" to "transmetalation" in the manuscript, as suggested by the reviewer.

Figure 1 is not referred to in the text of the document. Also, figure 1 doesnt really do justice to related chemistry that is known, i.e. JACS 2015, 137, 9523–9526.

The reviewer is right. Figure 1 was referenced in the manuscript. We added two sentences in the manuscript, as shown below:

"Previously, Fu and coworker reported a highly enantioselective nickel-catalyzed coupling of fluoroalkylated secondary alkyl bromide with aryl zinc chlorides (Figure 1).[31] Thus, the current method represents a complementary method for the construction of fluoromethylated stereogenic centers."

A reference about Fu's work was added as ref. 31.

(31) Liang, Y.; & Fu, G. C. Stereoconvergent Negishi Arylations of Racemic Secondary Alkyl Electrophiles: Differentiating between a CF_3 and an Alkyl Group. *J. Am. Chem. Soc.* **137**, 9523 (2015).

Furthermore, Figure 1 was modified as shown below:

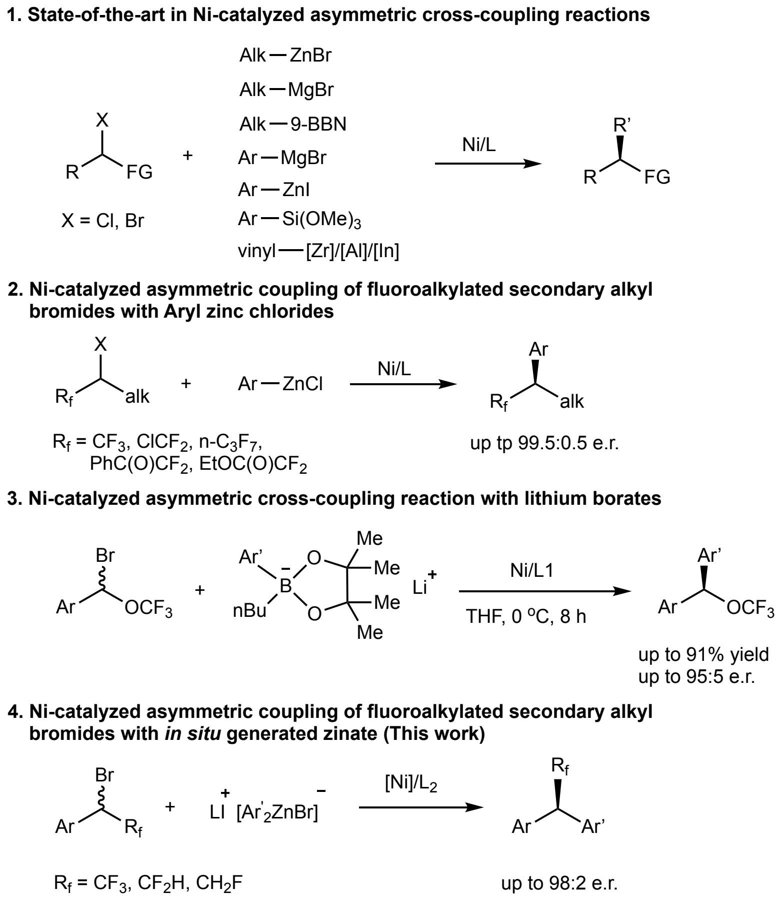

Figure 1. Ni-catalyzed asymmetric cross-coupling of racemic secondary alkyl halides.

I believe for the examples provided, "racemic" is the proper terminology, rather than "prochiral" when referring to the organic halides. This should be corrected.

We thank the reviewer for his suggestion. “prochiral” was changed to “racemic” in the manuscript.

Line 139: should read "might be involved"

The sentence was changed to as below, as suggested by the reviewer.

“These experimental results suggest that an anionic zincate $[Ph_2ZnBr]^-$ might be involved in the reaction”.

Reviewer 2

Shen and co-workers report the extension of their enantioselective coupling of racemic substituted benzyl halides where the FG is now CF3/CHF2/CFH2, instead of OCF3 (as documented in their previous work - Angew.Chem.2017,129,12148). Notably, in this work the coupling was ineffective using their previously identified

conditions and only was effective using combinations of the [nBu(Ph)BPin]-boronate and ZnBr2 which generates zincates in-situ. They then use this procedure to generate an impressive selection of CF3 and CF2H/CFH2 substituted cross-coupled products all with good to excellent e.r. values. ***Due to the importance of these compounds, the broad scope and the high e.r values observed I believe this paper is suitable for publication in Nat. Comm once the following minor points have been addressed.***

We thank the reviewer for his appreciation of our work.

Are L2 ligated Zinc species able to function as nucleophiles fro cross coupling in the absence of the Ni salt? If the authors have not tried the coupling with stoichiometric ZnBr2 in the absence of Ni salts they must do so.

Control experiment in the absence of nickel precursor was conducted, as suggested by the reviewer. It was found that the formation of the desired product **3a** was not observed. Therefore, the possibility that L_2 ligated zinc species function as nucleophiles for cross coupling was excluded. Scheme 1 was modified accordingly, as s shown below (The structures of the ligands were omitted):

Scheme 1. Optimization of the conditions for nickel-catalyzed asymmetric coupling of α-bromo-4-methoxycarbonylbenzyl trifluoromethyl 1a with lithium boronate 2.[a,b]

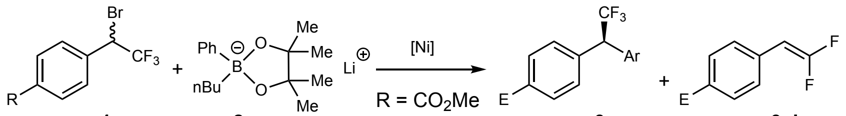

1a **2** **3a** **3a'**

entry	[Ni]	ligand	additive	solvent	temp (°C)	yield (%) 3	yield (%) 3'	e.r.
1	$NiBr_2$•DME	**L2**	$ZnBr_2$	DME	-15	62	5	95.5:4.5
2	$NiBr_2$•DME	**L2**	$ZnCl_2$	DME	-15	54	8	95:5
3	$NiBr_2$•DME	**L2**	$MgBr_2$	DME	-15	0	0	-
4	$NiBr_2$•DME	**L2**	$ZnBr_2$	diglyme	-15	78	0	95:5
5	$NiBr_2$•DME	**L2**	$ZnBr_2$	THF	-15	50	28	94.5:5.5
6	$NiBr_2$•DME	**L2**	$ZnBr_2$	toluene	-15	0	-	-
7	$NiBr_2$•DME	**L2**	$ZnBr_2$	DMA	-15	47	6	93:7
8	$NiBr_2$•DME	**L2**	$ZnBr_2$	DMF	-15	30	6	93:7
9	$NiBr_2$•DME	**L2**	$ZnBr_2$	DME/diglyme	-15	80	0	96:4
10	$NiBr_2$•DME	**L2**	$ZnBr_2$	DME/diglyme	rt	75	15	94:6
11	$NiBr_2$•DME	**L2**	$ZnBr_2$	DME/diglyme	-15	32	5	95.5:4.5[c]
12	$NiBr_2$•DME	**L2**	$ZnBr_2$	DME/diglyme	-15	75	2	95.5:4.5[d]
13	$NiCl_2$•DME	**L2**	$ZnBr_2$	DME/diglyme	-15	70	9	95.5:4.5
14	$Ni(OAc)_2$	**L2**	$ZnBr_2$	DME/diglyme	-15	71	6	96:4
15	$NiBr_2$•DME	**L1**	$ZnBr_2$	DME/diglyme	-15	70	15	88:12
16	$NiBr_2$•DME	**L3**	$ZnBr_2$	DME/diglyme	-15	81	0	94:6
17	$NiBr_2$•DME	**L4**	$ZnBr_2$	DME/diglyme	-15	30	50	90:10
18	$NiBr_2$•DME	**L5**	$ZnBr_2$	DME/diglyme	-15	0	0	-
19	$NiBr_2$•DME	**L6**	$ZnBr_2$	DME/diglyme	-15	24	0	58:42
20	-	**L2**	$ZnBr_2$	DME/diglyme	-15	0	0	-[e]
21	$NiBr_2$•DME	**L2**	$ZnBr_2$	DME/diglyme	-15	70	9	96:4[f]
22	$NiBr_2$•DME	**L2**	$ZnBr_2$	DME/diglyme	-15	50	15	96:4[g]

[a]Reaction conditions: compound **1a** (0.1 mmol), **2** (0.3 mmol), $NiBr_2$•DME (20 mol%), ligand (25 mol%) under conditions indicated in the scheme; [b]Yields were determined by ^{19}F NMR spectroscopy with trifluorotoluene as

an internal standard; [c]0.5 equiv. of $ZnBr_2$ was used; [d]2.0 equiv. of $ZnBr_2$ was used. [e]$NiBr_2$•DME (0 mol%), ligand **L2** (25 mol%); [f]$NiBr_2$•DME (10 mol%), ligand **L2** (12.5 mol%); [g]$NiBr_2$•DME (5 mol%), ligand **L2** (6.25 mol%).

In the reaction between 3 eq. of ***2a*** *and* $ZnBr_2$ *what is observed in the* ^{11}B *NMR spectrum? Are both the borpnate* ***2a*** *and the expected by-product BuBPin observed? i.e. is the* ^{11}B *NMR spectrum also consistent with incomplete consumption of* ***2a****, so formation of mixed arly/halo zincates?*

We thank the reviewer for his constructive suggestion. We studied the ^{11}B NMR spectrum of the reaction between 3.0 equiv. of **2a** and $ZnBr_2$ and the ^{11}B NMR spectrum was shown below. From the ^{11}B NMR spectrum, a peak with a chemical shift at 34.12 ppm was observed, which was corresponding to BuBpin. At the same time, a peak corresponding unreacted lithium organoboronate **3a** with a chemical shift at 7.49 ppm was also observed. These results were consistent with those observed from ^{13}C NMR spectroscopy of the reaction. The ^{11}B NMR spectrum was added to the supporting information.

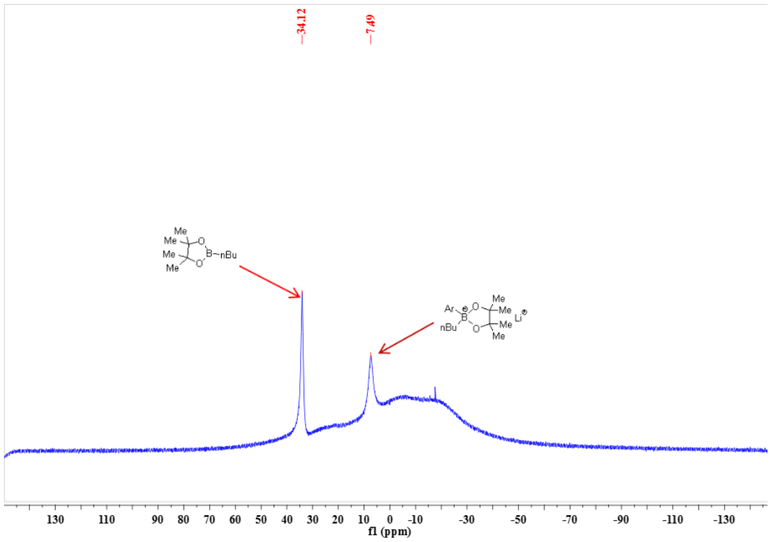

Figure S6. ^{11}B NMR spectra of **2a**+$ZnBr_2$ in THF-*d8*.

Can the ligand/catalyst loading be dropped at all from 20/25 mol%?

Two control experiments with lower catalyst loadings were conducted, as suggested by the reviewer. It was found that the yield decreased to 70% with the same enantioselectivity when a combination of 10 mol% $NiBr_2$•DME and 12.5 mol% **L2** was used as the catalyst. However, the formation of side product **3a'** was observed in 9% yield (Scheme 1, entry 21). Likewise, the yield decreased further to 50% when a combination of 5.0 mol% $NiBr_2$•DME and 6.25 mol% **L2** was used as the catalyst, while the yield of side product **3a'** increased to 15% (Scheme 1, entry 22). These results have been added to Scheme 1, as shown above.

Scheme 1 / footnotes contain no info. on amount of Ni / L, these details should also be shown in the footnote/scheme.

The footnote in Scheme 1 was changed, as suggested, as shown below:

[a]Reaction conditions: compound **1a** (0.1 mmol), **2** (0.3 mmol), $NiBr_2$•DME (20 mol%), ligand (25 mol%) under conditions indicated in the scheme; [b]Yields were determined by ^{19}F NMR spectroscopy with trifluorotoluene as an internal standard; [c]0.5 equiv. of $ZnBr_2$ was used; [d]2.0 equiv. of $ZnBr_2$ was used. [e]$NiBr_2$•DME (0 mol%), ligand **L2** (25 mol%); [f]$NiBr_2$•DME (10 mol%), ligand **L2** (12.5 mol%); [g]$NiBr_2$•DME (5 mol%), ligand **L2** (6.25 mol%).

Reviewer 3

1. Quite high catalyst loading (20mol% of Ni salt and 25mol% of the ligand) is used. Why? What happens with a lower loading? This data should be provided in the optimization table – Scheme 1.

Two control experiments with lower catalyst loadings were conducted, as suggested by the reviewer. It was found that the yield decreased to 70% with the same enantioselectivity when a combination of 10 mol% $NiBr_2$•DME and 12.5 mol% **L2** was used as the catalyst. However, the formation of side product **3a’** was observed in 9% yield (Scheme 1, entry 21). Likewise, the yield decreased further to 50% when a combination of 5.0 mol% $NiBr_2$•DME and 6.25 mol% **L2** was used as the catalyst, while the yield of side product **3a’** increased to 15% (Scheme 1, entry 22). These results have been added to Scheme 1, as shown above.

2. Same about Ar-boronates: 3(!) equivalents are used. What is the reason? What happens with a lower loading?

We appreciate the reviewer for his sharp eye in identifying the amount of the lithium organoboronate. Ideally, 2.0 equivalents of lithium aryl boronates are enough to react with $ZnBr_2$ to generate the reactive species $[Ph_2ZnBr]Li$. However, in the reaction, part of lithium aryl boronate was consumed to reduce $NiBr_2$•DME to generate the reactive nicker species. As a matter of fact, 1,1'-biphenyl was isolated as a side product. In addition, there is always a protonation/deboration side product that also consumed part of the lithium aryl boronate. Therefore, to make sure the full conversion of the starting material, 3.0 equivalents of lithium aryl boronate are used.

3. All examples, given throughout the text, are based on the benzylic bromides bearing only electron-withdrawing substituents on the aromatic ring. This is definitely a limitation of the approach, which should be clearly stated in the text. If not, then the examples of benzylic electrophiles bearing electron donating substituents should be added.

The reviewer was right about substrate scope. We did try the reactions of the benzylic bromides bearing electron-donating substituents such as *tertiary* butyl or methoxy group. The yields were much lower than those bearing electron-withdrawing groups. In these cases, the formations of two side products including the homocoupling side products and the defluorinated side products were observed. A sentence was added in the manuscript, as suggested by the reviewer, as shown below.

"Nevertheless, trifluoromethylated benzylic bromides with electron-donating groups occurred in much less yields and moderate enantioselectivities. In these cases, the formations of two side products including the homocoupling side products and the defluorinated side products were observed."

In addition, two examples of benzylic electrophiles bearing electron donating substituents were added in **Scheme** 2, as shown below.

Scheme 2. Scope of nickel-catalyzed asymmetric coupling of α-bromo-benzyl trifluoromethane with lithium aryl boronates. [a,b]

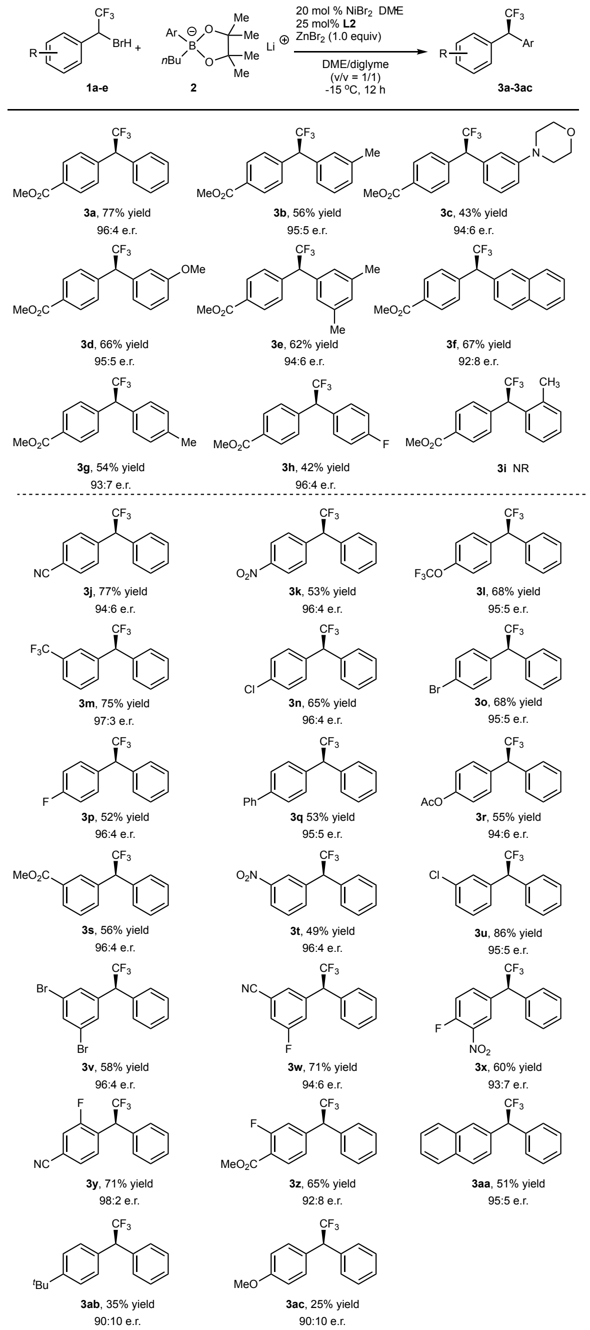

[a]Reaction conditions: compound **1** (0.3 mmol), phenylboronic pinacol ester **2** (0.9 mmol), $NiBr_2$•DME (20 mol%), ligand **L2** (25 mol%) and $ZnBr_2$ (0.3 mmol) in DME/diglyme (v/v = 1/1) at -15 °C for 12 h; [b]Isolated yields.

4. I have a similar claim regarding a scope of aryl boronates: it includes only 6 examples. No examples of ortho- or/and para- substituted aryl boronates are provided. Such examples would enrich the work, and should be given. Otherwise, it should be noted that such compounds are not suitable for this transformation.

We thank the reviewer for his constructive suggestions. Lithium aryl boronates with *ortho*- and *para*- substituted were examined as suggested. Reaction of *para*-methyl or fluoride substituted lithium aryl boronates occurred to generate the corresponding products in moderate yields with good enantioselectivities. However, the formation of the desired coupling product was not observed when lithium aryl boronates with *ortho*- methyl group was subjected to the reaction conditions. A paragraph was added as below:

"Next, we investigated the scope of lithium aryl boronates. It was found that reactions of lithium aryl boronates with *meta*-substituted aryl groups occured in moderate to good yields and enantiolectivities (Scheme 2, **3b**-**e**), while reactions of *para*-methyl or fluoride substituted lithium aryl boronates occurred to generate the corresponding products in moderate yields and enantioselectivities (Scheme 2, **3g**-**h**). However, the formation of the desired coupling product was not observed when lithium aryl boronates with ortho-methyl group was subjected to the reaction conditions (Scheme 2, **3i**).".

Accordingly, part of Scheme 2 was modified as shown below:

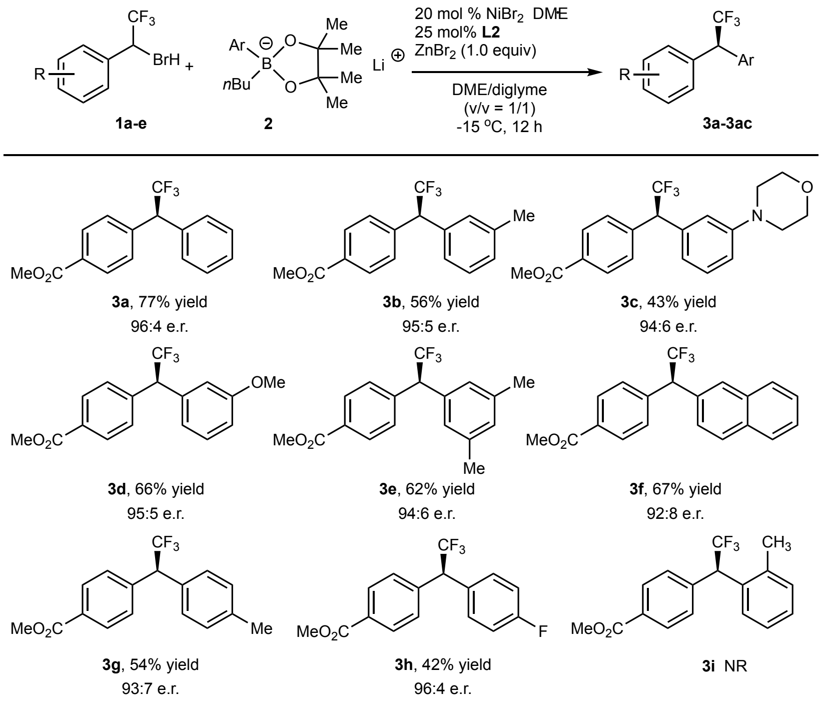

5. It is informally agreed in a catalytic chemical community, that ee's above 90% are called "high". In this work most of the ee's are around 88% (some are above, but many are significantly lower). Also, the yields of compounds are in the range of

50-70% - see Schemes 2-3. I would render from calling them "high yields and high enantioselectivity" as authors do throughout the text. Rather, I would modestly say that compounds are obtained in a moderate to good yields and enantioselectivity.

We changed "high yields and high enantioselectivity" to "moderate yields and good enantioselectivities", as suggested by the reviewer.

6. Regarding the mechanistic studies, it is way far from informative. Authors claim that the active species are [Ar2ZnBr]Li, which could be probably prepared independently from Ar2Zn and LiBr. Is there any attempt to isolate these species? Are they known previously? What about their full NMR? XPS might be helpful to say something about Zn and its coordination sphere. All these questions should be addressed.

Previously, Ingleson and co-workers reported the the formation of $[Ar_xZnBr_y]Li$ (x+y = 3) by the reaction of [^{t}BuB(Pin)Ph]Li with $ZnBr_2$ in THF (*Chem. Eur. J.* **2017**, *23*, 15889). We also tried to isolate $[Ar_2ZnBr]Li$ by layering a THF solution of this compound with pentane and crystallizing at -35 °C. However, these efforts to obtain a pure compound failed.

As suggested by the reviewer, we studied the ^{1}H NMR spectra for the reaction of [^{n}BuB(Pin)Ph]Li with $ZnBr_2$ in THF-d8, as well as the reaction of Ph_2Zn with LiBr. These NMR spectra were added in the supporting information.

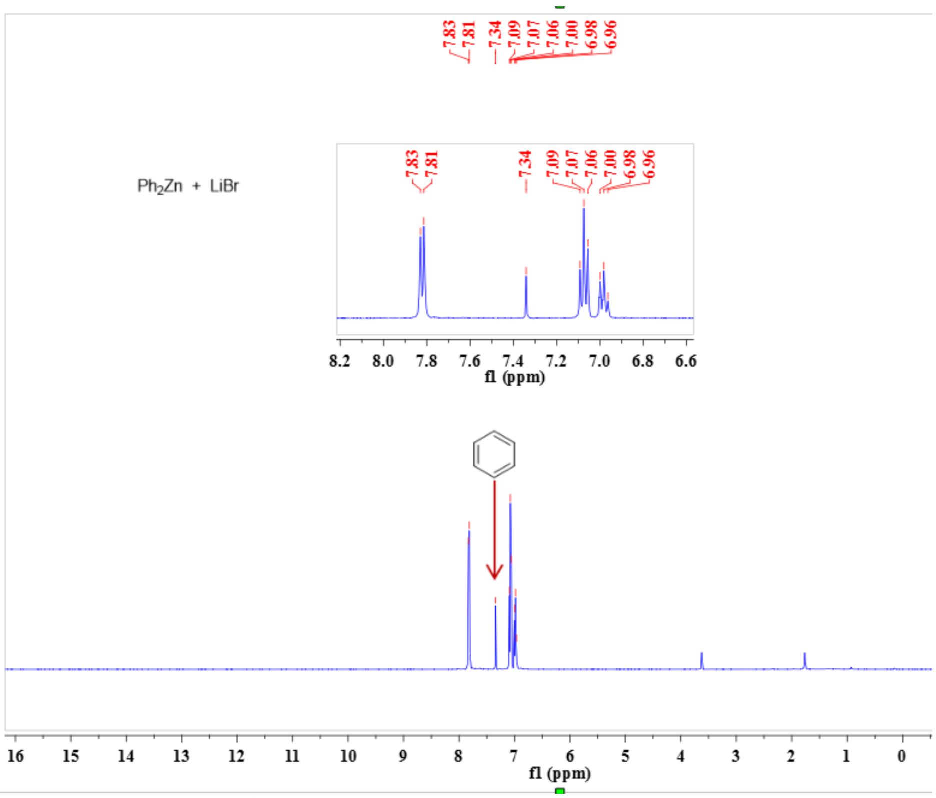

Figure S1. ^{1}H NMR spectra of Ph_2Zn + LiBr in THF-*d8*.

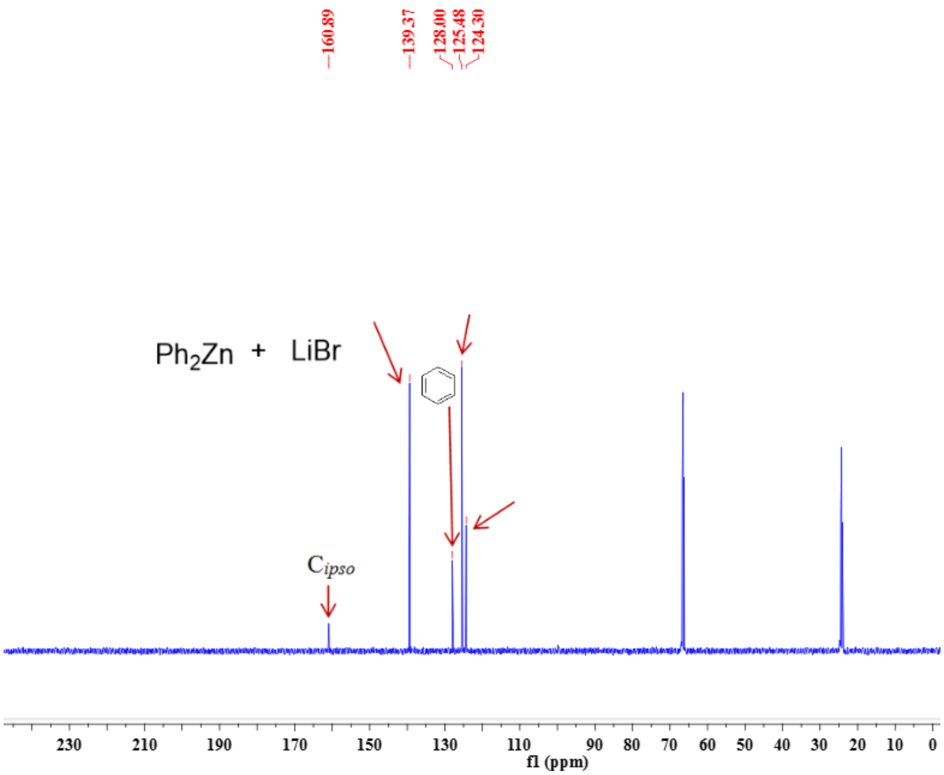

Figure S2. ^{13}C NMR spectra of Ph_2Zn + LiBr in THF-*d8*.

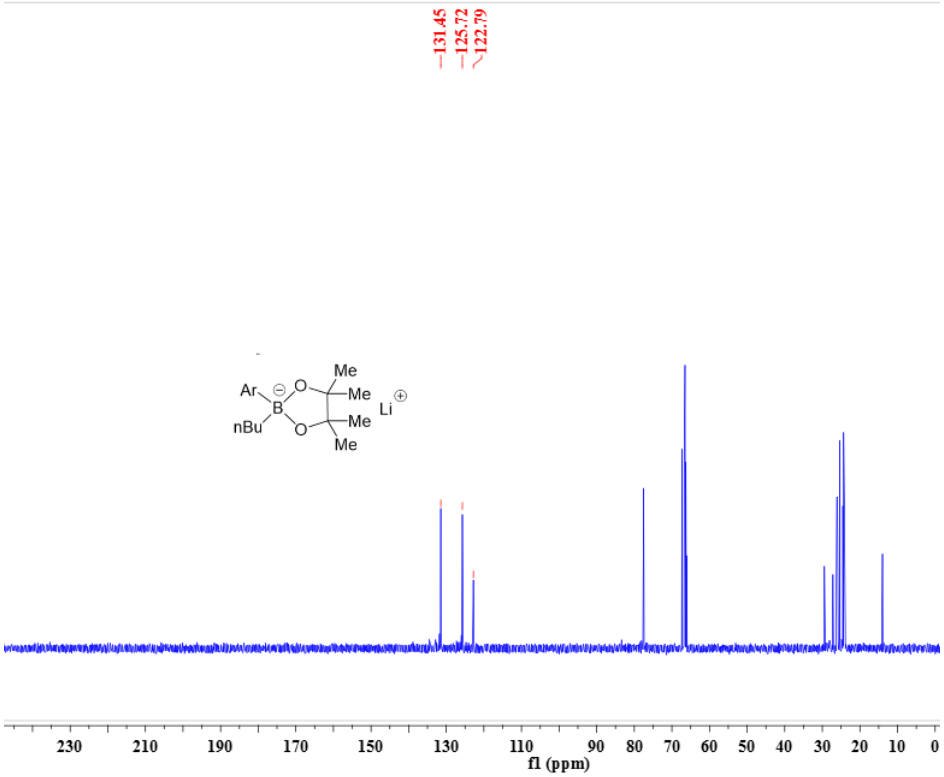

Figure S3. ^{13}C NMR spectra of **2a** in THF-*d8*.

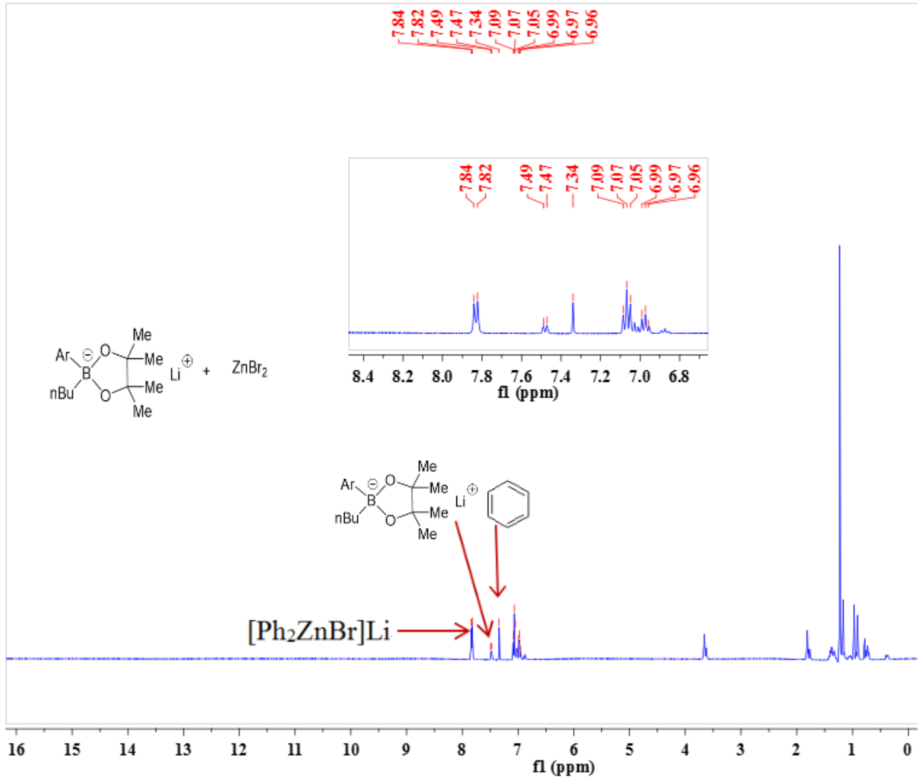

Figure S4. ^{1}H NMR spectra of **2a**+$ZnBr_2$ in THF-*d8*.

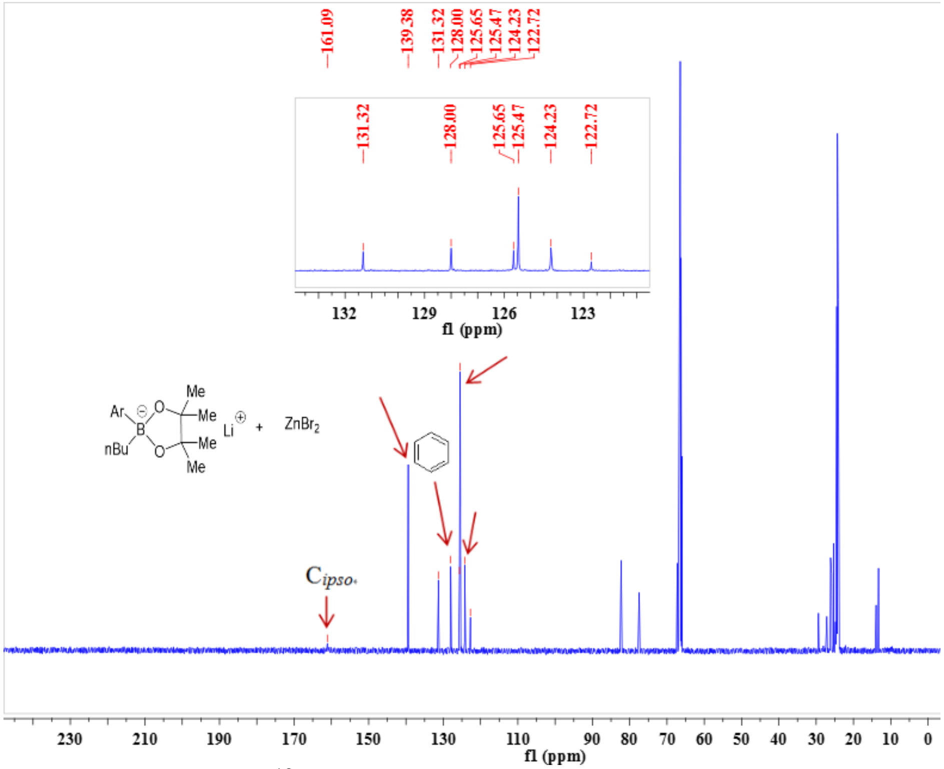

Figure S5. ^{13}C NMR spectra of **2a**+$ZnBr_2$ in THF-*d8*.

We also studied the ^{11}B NMR spectrum of the reaction between 3.0 equiv. of **2a** and $ZnBr_2$ and the ^{11}B NMR spectrum was shown below. From the ^{11}B NMR spectrum, a peak with a chemical shift at 34.12 ppm was observed, which was corresponding to BuBpin. At the same time, a peak corresponding unreacted lithium organoboronate **3a** with a chemical shift at 7.49 ppm was also observed. These results were consistent with those observed from ^{13}C NMR spectroscopy of the reaction. The ^{11}B NMR spectrum was added to the supporting information.

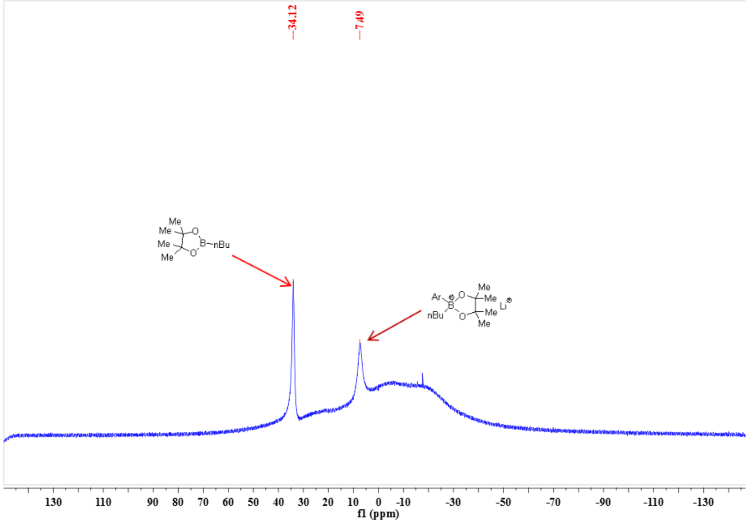

Figure S6. ^{11}B NMR spectra of **2a**+$ZnBr_2$ in THF-*d8*.

These NMR studies supported that the anionic zincate $[Ph_2ZnBr]Li$ was generated when $[^nBuB(Pin)Ph]Li$ reacted with $ZnBr_2$ in THF.

7. If authors propose trans-metallation from boron to zinc, is it possible to say something about Keq of this thermodynamically unflavored process?

We observed the full conversion for the reaction of 3.0 equivalents of $[^nBuB(Pin)Ph]Li$ with $ZnBr_2$ to generate $[Ph_2ZnBr]Li$ and *n*BuBpin, as shown in the ^1H, ^{13}C and ^{11}B NMR spectra of the reaction mixture in THF-d8. It is true that $[Ph_2ZnBr]Li$ is more reactive and likely less stable than $[^nBuB(Pin)Ph]Li$. However, there are four compounds in the equation shown below and it is difficult to determine the thermodynamic stabilities of the reactants and the products.

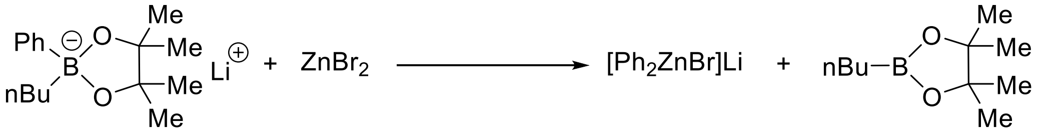

8. Moreover, according to stoichiometry of this reaction two equiv. per one mole of $ZnBr_2$ is enough. What happens with a third equiv. that is used in the procedure?

For the stoichiometric reaction of [^{n}BuB(Pin)Ph]Li with $ZnBr_2$, as can see from the ^{1}H, ^{13}C and ^{11}B NMR spectra of the reaction of 3.0 equivalents of [^{n}BuB(Pin)Ph]Li with $ZnBr_2$, part of the unreacted [^{n}BuB(Pin)Ph]Li was left in the reaction mixture.

9. Probably, a most important point: if [Ar_2ZnBr]Li are truly active nucleophile in the reaction and can be prepared by Ar_2Zn + LiBr, why should one use expensive organoboron precursors at all?? The paper would be much more elegant, if the series of Ar_2Zn would be prepared and used as the nucleophiles in the presence of LiBr. In my opinion, it is an important issue that should be done prior to publication.

We thought that using lithium aryl boronates as the nucleophiles in the cross-coupling reaction is advantageous over the Ar_2Zn. First, very few diarylzinc reagents as well as lithium aryl boronates are commercially available. However, lithium aryl boronates can be easily prepared by mixing *n*BuLi with ArBpin since hundreds of ArBpin can be obtained from the commercial suppliers. On the other hand, Ar_2Zn is generally made from ArMgBr or ArLi. Nevertheless, only a few ArMgBr or ArLi can be obtained from the commercial suppliers. Anyway, we thank the reviewer for his constructive suggestion.

REVIEWERS' COMMENTS:

Reviewer #1 (Remarks to the Author):

The authors now have made revisions that greatly strengthen the manuscript and make it more suitable for publications. The following points need addressing though before publication:

1) In Scheme 1, equation 4, the authors mistakenly write "LI" instead of "Li"

2) A smoother reading of added text should read: "These results suggest that an anionic arylated zincate [Ph2ZnBr]Li could facilitate the transmetalation step and, consequently, accelerate the overall catalytic reaction. However, we could not exclude an alternate pathway in which the ZnBr2 abstracts a bromide from a nickel benzyl bromide complex to generate a cationic nickel species(reference) that could more rapidly participate in a transmetalation reaction with lithium organoborate 2a."

Reviewer #2 (Remarks to the Author):

In this work Shen and co-workers describe C(sp3)-C(sp2) asymmetric Suzuki-Miyaura cross-coupling reaction of fluoroalkyl substituted benzylic bromides with aryl borates. The reaction leads to families of interesting enantioenriched compounds, bearing fluoroalkyl group at the stereogenic center, which are of potential importance to drugs and agrichemicals discovery. The key point is a finding that an addition of ZnBr2 is necessary to enable the reactivity, and an assumption that diaryl zincates [Ar2ZnBr]Li represent a true active trans-metallating spices is made.

I have reviewed the revised manuscript of this paper. I am satisfied with the changes, additions or comments made by authors. Therefore, I recommend a publication of this manuscript in Nat. Commun. as is.

Reviewer #3 (Remarks to the Author):

This revised manuscript from Shen and co-workers has been improved notably by their additional work and clarifications. It is my opinion that they have appropriately addressed all the requests from the reviewers (indeed they have gone further than I would have done to address some of the comments from other reviewers). Thus this revised manuscript is fully suitable for publication essentially as is.

Some minor comments:
Regarding reviewer 1's comment – the presence of ZnBr2 is highly unlikely in the presence of excess anionic organoboronate (i.e. the reaction conditions used herein) – thus including the statement regarding halide abstraction by ZnBr2 is not necessary in my opinion. Though it is correct to be less definitive regarding the exact role of zinc species, as it has not be established 100%, so their modified language is more appropriate e.g. such as "Having identified the optimized conditions and the likely role of ZnBr2" (use likely not likelihood)

Regarding reviewers 3's comment 7 – I agree with the authors response, the transmetalation from boron to zinc in combination with the formation of the other products is exergonic in the case reported herein using these more reactive boronates, this has been demonstrated a number of times now by separate groups. This is different to the use of more classical boron transmetalating agents (e.g. RB(OR)3 anions)

Regarding reviewers 3's comment 9 – I agree with the author's response, furthermore the

formation of “Ar2Zn” as a single species is challenging e.g. from Grignard reagents, the formation of mixed zincates is generally the outcome which may well complicate the reactivity (though if the right zincate is formed it may still work from Grignard reagents/ZnBr2). The addition of nBuLi is simple as the authors state and the transmetalation to zinc appears to give a single product the R2ZnBr monoanionic zincate under these conditions.

Reviewer 1

1) In Scheme 1, equation 4, the authors mistakenly write "LI" instead of "Li"

“LI” was change to “Li” as suggested.

2) A smoother reading of added text should read: "These results suggest that an anionic arylated zincate [Ph2ZnBr]Li could facilitate the transmetalation step and, consequently, accelerate the overall catalytic reaction. However, we could not exclude an alternate pathway in which the ZnBr2 abstracts a bromide from a nickel benzyl bromide complex to generate a cationic nickel species(reference) that could more rapidly participate in a transmetalation reaction with lithium organoborate 2a."

We thank the reviewer’s suggests. The sentences were modified as suggested, as shown below:

“These results suggest an anionic arylated zincate $[Ph_2ZnBr]Li$ could facilitate the transmetalation step, and consequently, accelerates the overall catalytic reaction. However, we could not exclude an alternative pathway in which $ZnBr_2$ may abstract a bromide from nickel benzylic bromide complex to generate a cationic η^3-benzyl nickel species[33] that could more rapidly participate in a transmetalation reaction with lithium organoboronate **2a**.”

With these changes, we believe that we have addressed the reviewers’ comments and thanks to the reviewers’ constructive suggests, the quality of the manuscript did improve. We also would like to thank you for your kind considerations.
